# Revisiting the enigmatic Cheirolepidiaceae: origins, phylogenetic relationships, and a new whole-plant concept

**DOI:** 10.1093/aob/mcag069

**Published:** 2026-03-30

**Authors:** Ana Andruchow-Colombo, Kelly K S Matsunaga

**Affiliations:** Department of Ecology and Evolutionary Biology & Biodiversity Institute, University of Kansas, Lawrence, KS 66045, USA; Department of Ecology and Evolutionary Biology & Biodiversity Institute, University of Kansas, Lawrence, KS 66045, USA

**Keywords:** Cheirolepidiaceae, Early Cretaceous, conifer evolution, Holly Creek Formation, phylogenetic position of Cheirolepidiaceae, total evidence conifer phylogeny, *Arkansia*, *Pseudofrenelopsis*, *Classostrobus*

## Abstract

**Background and Aims:**

The extinct conifer family Cheirolepidiaceae spanned from the Late Triassic to early Palaeocene and was distributed worldwide. While it is known from multiple organs, there are no Cheirolepidiaceae whole-plant reconstructions to date. The relationships of Cheirolepidiaceae with different plant groups have been extensively discussed in the literature, but their phylogenetic position has not been thoroughly explored. To fill these gaps, we studied seed cones associated with other Cheirolepidiaceae organ remains from the Early Cretaceous of Arkansas and investigated their phylogenetic position.

**Methods:**

We studied previously collected fossil remains from the Holly Creek Formation and included them in total evidence phylogenetic analyses with other Cheirolepidiaceae species and representatives of all major living and extinct conifer groups. We performed a time-calibrated Bayesian analysis under the fossilized birth–death model and 60 maximum parsimony analyses with varying search conditions.

**Key Results:**

We describe *Arkansia axsmithii* sp. nov. based on seed cones and isolated ovuliferous complexes associated with previously described wood, leafy shoots, pollen cones and pollen. We separated the associated vegetative material into *Pseudofrenelopsis axsmithii* sp. nov. Our phylogenetic analyses most frequently show the Cheirolepidiaceae as sister to the crown group of conifers, and more rarely as sister to Araucariales, Cupressales + Araucariales, or *Araucaria*. In some cases, *Pararaucaria* is recovered outside of Cheirolepidiaceae. We estimated ages of Carboniferous for the divergence of conifers and Cordaitales, and Late Triassic for the initial diversifications of Cheirolepidiaceae and the conifer crown group.

**Conclusions:**

The *Arkansia* plant constitutes the most completely known Cheirolepidiaceae plant to date. Our phylogenetic analyses support Cheirolepidiaceae as a transitional lineage between voltzialean and living conifers, reflected in their ovuliferous complex morphology, which shows intermediate traits between voltziacean voltzialeans and extant taxa. Age estimates of deep nodes of conifer phylogeny are broadly congruent with the fossil record, suggesting a long-lasting stem group and an origin of crown-group conifers in the Late Triassic.

## INTRODUCTION

The conifers are the most diverse living group of gymnosperms, today represented by three orders, six families, 70 genera and over 600 species (Christenhusz *et al.*, 2011; Farjon, 2017; Page 2019). The fossil record of conifers goes back to the Pennsylvanian (358.9–323.2 million years ago [Ma]; Florin, 1954; Mapes and Rothwell, 1991; Stewart and Rothwell, 1993; Hernandez-Castillo *et al.*, 2001, 2009; Rothwell and Mapes, 2001; Rothwell *et al.*, 2005). These ancient conifers, most of which are classified into the order Voltziales, exhibited complex seed cone morphologies, with cone bracts subtending axillary shoots bearing multiple sterile leaves and fertile sporophylls (Stewart and Rothwell, 1993; Rothwell *et al.*, 2005; Taylor *et al.*, 2009). Between the Permian and the Triassic, a distinctive trend of simplification of seed cones by means of reduction and fusion of cone elements can be observed among voltzialean taxa (Clement-Westerhof, 1984; Mapes and Rothwell, 1984; Schweitzer, 1996; Rothwell *et al.*, 2005; Escapa *et al.*, 2010). Towards the Jurassic, fossil conifers with modern seed cone morphologies begin to appear in the fossil record. These modern-looking conifers belong to the six living families – Araucariaceae, Cupressaceae, Pinaceae, Podocarpaceae, Sciadopityaceae and Taxaceae (Stockey, 1975, 1982; Kunzmann, 2007; Escapa *et al.*, 2008; Rothwell *et al.*, 2012; Spencer *et al.*, 2015; Matsunaga *et al.*, 2021; Andruchow-Colombo *et al.*, 2023) – and the extinct family Cheirolepidiaceae (Alvin, 1982; Watson, 1982, 1988). In these families, seed cone morphology is more compact than that of earlier conifers (i.e. fewer elements closely packed on the cone axis), with bracts subtending a leaf-like ovule-bearing organ (ovuliferous scale), usually interpreted as the highly fused and reduced fertile axillary shoot (Florin, 1951, 1954; Matsunaga, 2026; but see Herting and Stützel, 2022). Among these modern-looking conifer families, the extinct Cheirolepidiaceae are the least understood in terms of their morphology and their relationships to living families (Archangelsky, 1968; Harris, 1979; Alvin, 1983; Clement-Westerhof and Konijnenburg-van Cittert, 1991; Kvaček, 2000; Axsmith and Jacobs, 2005; Escapa and Leslie, 2017).

The fossil record of Cheirolepidiaceae spans from the Upper Triassic (Axsmith *et al.*, 2004*a*) to the Lower Palaeocene (Barreda *et al.*, 2012; Berry, 2022) and includes remains of wood, leaves, pollen cones, seed cones and dispersed pollen grains (Archangelsky, 1968; Clement-Westerhof and Konijnenburg-van Cittert, 1991; Axsmith and Jacobs, 2005). The family was distributed worldwide and was especially diverse during the Jurassic and Early Cretaceous (Steart *et al.*, 2014; Hieger *et al.*, 2015; Thevenard *et al.*, 2022). Species assigned to the family encompass a wide diversity of leaf morphologies (Watson, 1982), of which the forms characterized by sheathing leaf bases assigned to the genera *Frenelopsis* (two or three leaves per node) and *Pseudofrenelopsis* (one leaf per node) are highly characteristic of the family. Other distinctive leaf characters for many Cheirolepidiaceae include the presence of papillae on ordinary epidermal cells and in subsidiary cells (Watson, 1988). Seed cones are variable but characterized by a distally lobed ovuliferous scale that bears one or two inverted ovules apparently embedded in the scale tissues (Escapa *et al.*, 2012). Pollen grains of Cheirolepidiaceae, referred to *Classopollis*, are diagnostic of the family*. Classopollis* pollen grains are spherical with a cryptopore at the distal pole, a triangular depressed area at the proximal pole, an equatorial striated band and a subequatorial furrow or rimula (Clement-Westerhof and Konijnenburg-van Cittert, 1991; Zavialova *et al.*, 2010; Kvaček and Mendes, 2023).

Stanley (1988) described multiple organs of cheirolepidiaceous affinities recovered from the Aptian/Albian of Holy Creek Formation, Arkansas, USA. The remains included leafy shoots that were assigned to *Pseudofrenelopsis parceramosa* (Fontaine) Watson, pollen cones in organic connection with the shoots, dispersed *Classopollis* pollen, seed cones and wood. This record constitutes one of the most complete assemblages of organs known for a Cheirolepidiaceae taxon, but none of its organs were validly published by Stanley. Later, some of the original material and newly collected specimens were formally described in two publications (Axsmith *et al.*, 2004*b*; Axsmith, 2006). These included pollen cones with *in situ Classopollis* pollen, leafy shoots and wood. The pollen cones with *in situ* pollen were assigned to a new species, *Classostrobus arkansensis* (Axsmith *et al.*, 2004*b*), while the leafy shoots and wood fragments were retained in *P. parceramosa* (Axsmith, 2006). Logs at least 2 m in length were reported from the locality but were not collected (Axsmith, 2006). Preliminary descriptions of the seed cones and ovuliferous complexes associated with this species were reported by Creen (2006) and Axsmith and Creen (2005), but the ovulate reproductive structures of this species are yet to be formally published.

Here we revisit the original specimens of Stanley (1988) and the additional specimens collected by Axsmith and collaborators (2004b; see also Axsmith, 2006), including previously undescribed specimens. We formally describe the ovulate cones, modify the taxonomy of the leafy shoots and provide a whole-plant concept for this Cheirolepidiaceae species. This whole-plant concept is based on wood, leafy shoots with cuticle, pollen cones with *in situ Classopollis* pollen, ovulate cones and isolated ovuliferous complexes. To elucidate the evolutionary relationships of this extinct family among conifers, we performed total evidence phylogenetic analyses with other Cheirolepidiaceae species, together with representatives of all other extant and extinct conifer lineages, using both parsimony and Bayesian approaches. In addition, we performed a Bayesian analysis using the fossilized birth–death (FBD) model to coestimate the tree topology and divergence times. We discuss our findings in the context of the broader fossil record of conifers and hypotheses on the evolutionary relationships of Cheirolepidiaceae.

## MATERIALS AND METHODS

### Terminology

We use the term ‘voltzialeans’ to refer to the likely non-monophyletic group of conifers historically assigned to families of the order Voltziales (Matsunaga *et al.*, 2021; Andruchow-Colombo *et al.*, 2023). We use the expression ‘modern-looking’ for conifer families with compact seed cones that show a high degree of fusion of the axillary ovuliferous shoot, such that a distinct ‘ovuliferous scale’ can be recognized. These modern-looking groups include the six living conifer families and the extinct Cheirolepidiaceae.

### Geological setting and age

The Holly Creek Formation forms part of the Trinity Group, which encompasses Lower Cretaceous sediments cropping out in the southern USA (Suarez *et al.*, 2021). The Holly Creek Formation is stratigraphically positioned between the Dierks and De Queen formations (Stanley, 1988). Based on the invertebrate assemblage in the overlying De Queen Formation and on the invertebrate and palynological assemblages of correlative units, the age of the Holly Creek Formation was proposed to be late Aptian to early Albian (Stanley, 1988; Tanrikulu *et al.*, 2017; Suarez *et al.*, 2021). The material described here was collected at an exposure of the Holly Creek Formation found along a stream within the James Hardie Gypsum Quarry in south-west Arkansas, USA (Stanley, 1988; Axsmith *et al.*, 2004*b*). The palaeobotanical diversity of the fossil locality as described by Axsmith *et al.* (2004*b*) is extremely low, almost exclusively comprising remains of a single cheirolepidiaceous taxon, represented by wood, leafy shoots, pollen cones with pollen *in situ*, and seed cones. From another locality of the Holly Creek Formation a vertebrate fauna was described, including fishes, lissamphibians, turtles, crocodyliforms, dinosaurs and squamates (Suarez *et al.*, 2021).

### Fossil preparation and illustration and taxonomic name register

The fossils are preserved as compressions embedded in a grey clay that was bulk-collected, macerated in water and treated with hydrofluoric acid (Axsmith *et al.*, 2004*b*). Additional pollen cone material partially covered in sediment was treated for several days in a saturated solution of sodium hexametaphosphate (at 23 °C) to remove clay that obscured traits, then the remains were rinsed with ethanol 70 % and left to slowly dry in air.

Macro images of the specimens were taken with a Canon EOS 5DS (Canon, Tokyo, Japan) camera with a Canon MP-E 65 mm macro lens under halogen lighting projected at 45°. Photographs were processed with Adobe Photoshop Lightroom Classic 10.2 (Adobe, Mountain View, CA, USA) for white balance and contrast, and with Adobe Photoshop 22.3.1 for focus stacking and assembling plates.

Isolated leaf cuticles were treated with Schulze’s solution (Harris, 1926) to clear them for observation with light microscopy. Additionally, leaf cuticles, microsporophylls and an ovuliferous complex were mounted on scanning electron microscopy (SEM) stubs either with silver paint or with double-sided carbon tape, and covered with 5–15 nm of gold using an EMS/Quorum 150R S sputter coater. Samples were observed and photographed with a Hitachi SU8230 Field Emission Scanning Electron Microscope at the Microscopy and Analytical Imaging Research Resource Core Laboratory (MAI) at the University of Kansas. Some material previously prepared for SEM analysis by Dr Brian Axsmith was also observed at the MAI SEM facility. Those materials included wood sections, pollen cone fragments, a pollen mass and leaf fragments.

One ovuliferous complex and one seed cone specimen were studied using X-ray microcomputed tomography (µCT). The µCT scanning of the ovuliferous complex was performed at the Institute for Bioengineering Research of the University of Kansas with a Zeiss Xradia MicroXCT-400 Scanner using a 0.5× objective, with *XM-Controller* scanning software. The specimen was scanned using 50 kV of voltage and a current of 160 µA, with an exposure time of 2 s per image, taking 2000 projections reconstructed into 1012 stacked images at a voxel size of 22.88 µm. The µCT scanning of the seed cone was performed at California Polytechnic University, Humboldt (CPH, Arcata, CA) using a Nikon XTH 225 X-Ray µCT Scanner equipped with a tungsten reflection target. Specimens were scanned using 63 kV of voltage and a current of 95 µA, with an exposure time of 1.42 s per image. We reconstructed 4477 projections into a stack of 1704 images at a voxel size of 15.00 µm. Three-dimensional reconstructions and still images of µCT scans were produced using Mimics (Materialise, Leuven, Belgium). The two µCT image stacks were uploaded to Morphosource (https://www.morphosource.org) under project number 000818384. The ovuliferous complex scan has the object ID 000818529 (KU specimen number C2591), and the seed cone scan has the object ID 000818466 (KU specimen number C2596).

The fossil specimens are housed permanently in the Paleobotany Collection of the Biodiversity Institute and Natural History Museum of the University of Kansas (repository abbreviation for Cretaceous materials at the collection: C), Lawrence, KS, USA.

New taxon names were registered on the Plant Fossil Names Registry (PFNR) database (https://www.plantfossilnames.org); ID numbers of the new names are provided within the taxonomic sections.

### Phylogenetic analyses

#### Matrix

We used a modified version of the total evidence matrix published by Andruchow-Colombo *et al.* (2023; see also Matsunaga *et al.*, 2021), to which six new taxa were added. Newly added taxa are *Araucaria mirabilis* (Araucariaceae; Calder, 1953; Stockey, 1975) and the following Cheirolepidiaceae: *Tomaxellia biforme* (Early Cretaceous, Argentina; Archangelsky, 1966, 1968; Archangelsky and Gamerro, 1967), *Hirmeriella muensteri* (Early Jurassic, Germany; Clement-Westerhof and Konijnenburg-van Cittert, 1991; Barbacka *et al.*, 2007; Doweld, 2020), *Kachaikestrobus acuminatus* (Early Cretaceous, Argentina; Del Fueyo *et al.*, 2008), the *Alvinia bohemica* plant (Late Cretaceous, Czech Republic; Kvaček, 2000), and the new whole plant from Arkansas (Axsmith *et al*, 2004*b*; Axsmith, 2006; this work). In total, the taxon sampling used here includes *Ginkgo biloba*, 34 extant species from the six extant conifer families and 20 extinct species belonging to both extant and extinct conifer groups (Supplementary Data Appendix S1A).

The combined matrix includes 7372 characters, from DNA (7253 characters from the molecular markers 18S, PHYP, matK and rbcL) and morphology. The molecular block from this matrix is the same as in Andruchow-Colombo *et al.* (2023), but the morphological block was expanded. The 119 morphological characters include 71 seed cone characters (69 discrete and two continuous), 33 leaf characters, 6 pollen cone characters, 6 pollen characters and 3 pollination mechanism characters (Supplementary Data Appendix S1A). The morphological and combined matrixes can be found in Supplementary Data Appendix S1B, and the morphological matrix was also uploaded to MorphoBank as project 4603 (http://morphobank.org/permalink/?P4603).

#### Maximum parsimony

Maximum parsimony searches were conducted under a wide variety of conditions as a sensitivity analysis to test the robustness of phylogenetic topologies to different analytical assumptions. Parsimony analyses were performed in TNT 1.5 (Goloboff *et al.*, 2008; Goloboff and Catalano, 2016; Goloboff and Morales, 2023) with a range of conditions including equal weights and extended implied weights (Goloboff, 1993, 1997, 2014) with four alternative concavity constant values (*k* = 3, 6, 12, 15). All discrete characters were treated as unordered except for characters 23 (number of ovuliferous scale lobes), 61 (degree of fusion of the scale elements), 73 (leaf types), 86 (Florin ring) and, in some analyses, 64 (number of ovuliferous complexes in the seed cone) following Andruchow-Colombo *et al.* (2019; 2023). Following Andruchow-Colombo *et al.* (2023), we have three characters that reflect the variation in the number of ovuliferous complexes per cone. These are character 64, which is discrete with five states; character 0, which is the meristic version; and character 1, which corresponds to the logarithm with base 10 of character 0 (continuous character; see Andruchow-Colombo *et al.*, 2023). Because they all reflect the same aspect of cone morphology, these three alternative versions of the character were never active at the sametime in phylogenetic analyses but were alternatively activated to test their influence on resulting topologies. Furthermore, each of these characters was alternatively analysed under different assumptions: character 64 (discrete multistate) was either treated as ordered or unordered, and characters 0 (meristic) and 1 (continuous) were standardized, as is recommended for non-discrete characters (Thiele, 1993; Goloboff *et al.*, 2006), to either one or five maximum steps in different analyses (see further detail in Andruchow-Colombo *et al.*, 2023). Each analysis was performed alternatively with and without a monophyly constraint of the two main clades of Pinaceae (Abietoideae and Pinoideae–Piceoideae–Laricoideae) allowing for floating fossil taxa (i.e. fossil taxa are not restricted in or out of these clades). The constraint was implemented because analyses under parsimony resulted in relationships among certain extant Pinaceae genera inconsistent with the relationships routinely recovered by larger molecular datasets.

To summarize, the combination of analysis conditions includes five character-weighting schemes (equal weights and extended implied weighting with four alternative concavity constants), six alternative character settings (three alternative characters measuring the number of fertile units in the cone, each with two alternative settings), and the implementation (or non-implementation) of constraints within Pinaceae. The combinations of these conditions resulted in 60 analyses.

Searches were conducted by an initial round of random addition sequences, followed by tree bisection–reconnection (TBR) rearrangements and a combination of sectorial searches and tree fusing (as set by default under the command *xmult*), setting the stop after reaching the minimum length 25 times. An additional round of TBR rearrangements was performed from the trees obtained in the first round of the analysis and then the strict consensus of all the most parsimonious trees was calculated.

Analyses under equal weights gave spurious reconstructions of the deeper conifer nodes (Supplementary Data Appendixes S2A and S2Ai) and so additional analyses were performed under the same conditions with a positive constraint of the living conifer species, leaving all fossils as floaters except for voltzialeans (*Hanskerpia hamiltonensis*, *Emporia lockardii*, *Telemachus aequatus*, *Voltzia hexagona*, *Rissikia media*, *Nothodacrium warrenii*) and Cordaitales (*Cordaixylon dumusum*) (Supplementary Data Appendix S2B). This constraint was based on the topology recovered in other analyses performed here.

To further explore the placing of *Pararaucaria patagonica* within the parsimony trees, which frequently recovered it outside of the core Cheirolepidiaceae clade, we ran the TNT script *PlaceMyFossils* (Catalano *et al.*, 2025). *PlaceMyFossils* explores the parsimony scores of alternative positions of target taxa. We used a backbone tree based on the topology in Fig. 8 (i.e. the metaconsensus of the 48 analyses of extended implied weights) excluding *P. patagonica* (Supplementary Data Appendix S5). This *PlaceMyFossils* analysis was done using only the morphological matrix, as *P. patagonica* is a fossil species, under equal weight parsimony with characters 0, 1 and 64 as inactive.

#### Bayesian

Tree searches using MrBayes 3.2.7 (Huelsenbeck and Ronquist, 2001; Ronquist *et al.*, 2012*b*) were conducted using the version of the total evidence matrix with the discrete version of character referring to the number of ovuliferous complexes (i.e. character 64) and all characters unordered (Supplementary Data Appendix S3). Searches were conducted using two parallel runs of Metropolis-coupled Markov chain Monte Carlo, each with three hot chains and one cold chain. Chain length was set to 30 million generations per run and runs were sampled every 3000 generations, for a total of 10 000 sampled trees for each run. The morphological partition was analysed under the Mk model of morphological evolution corrected for ascertainment bias with γ-distributed between-character rate variation (Lewis, 2001). The molecular partitions were analysed using a GTR + Γ model. The first 25 % of samples were discarded as burn-in and the resulting sampled trees were summarized using an all-compatible majority-rule consensus (a 50 % majority rule consensus with all compatible groups added to the tree; Huelsenbeck *et al.*, 2015). The Bayesian analysis was rooted with *Cordaixylon*.

Convergence of independent runs was checked visually in Tracer v1.7.2 (Rambaut *et al.*, 2018) and using the average standard deviation of split frequencies (ASDSF ≈0.006), potential scale reduction factors (PSRF approximately equal values for all parameters) and effective sample size (ESS >200 for all parameters).

A consensus network was constructed with the trees sampled from the Bayesian analysis (excluding the burn-in, 15 000 trees) using the software SplitsTree 4.18.3 (Holland and Moulton, 2003; Huson and Bryant, 2006), with a threshold of 0.33 (relationships shown in the network appear in at least 33 % of the trees).

#### Divergence time analysis

Total evidence dating (*sensu*Ronquist *et al.*, 2012*a* ) was performed under a relaxed clock model and the FBD model with sampled ancestors (FBD-SA; Ronquist *et al.*, 2012*a*; Gavryushkina *et al.*, 2014; Heath *et al.*, 2014; Zhang *et al.*, 2016; Simões *et al.*, 2020) (Supplementary Data Appendix S4). The analysis was performed in MrBayes 3.2.7 with two independent runs with four chains each for 100 million generations, sampling every 10 000 generations. Following Simões *et al.* (2020), we provided an informative prior on the background clock rate. To obtain this prior (*clockratepr*), the median of the tree height of posterior trees (obtained from a previous strict clock analysis) was divided by the median of the of the root age prior (0.130436/327.1), and then the resulting value was transformed with a lognormal scale: ln(0.00039876) = −7.827, this value is the mean of the prior, while the e exponent of the ratio defined above was set as its standard deviation (e^0.00039876^ = 1.000). Clock rates for morphology and molecular partitions were set to be unlinked. Molecular and morphological models of evolution were the same as for the Bayesian non-clock analysis. The stratigraphic range and/or the uncertainty in the age of the fossil localities were used to inform the uniform prior distribution of fossil species ages. A topological constraint on the position of the fossil *Araucaria mirabilis* was enforced to prevent *A. mirabilis* from being recovered on the Araucariales stem, which occurred in preliminary analyses. This was done because there is compelling evidence that the species is most closely related to Araucariaceae (Stockey, 1975; Kunzmann, 2007), and to prevent its deeper placement in the tree from biasing the placement of Cheirolepidiaceae relative to the Araucariales.

Convergence of independent runs was checked using the same procedure as the non-clock analysis (ASDSF ≈0.003; PSRF ≈1; ESS >200). The first 25 % of samples were removed as burn-in and the resulting sampled trees were summarized using an all-compatible majority-rule consensus. Figures were built with the *MCMCtreeR* package (Puttick, 2019) in R and post-processed in Adobe Illustrator.

## RESULTS

### Systematic palaeontology

*Family –* Cheirolepidiaceae Takhtajan ex Turutanova-Ketova, 1963:249.

*Genus* – *Arkansia* Andruchow-Colombo et Matsunaga gen. nov.

*Etymology –* The name of this genus refers to the state of Arkansas (USA), where the fossil specimens were found.

*Type species* – *Arkansia axsmithii* Andruchow-Colombo et Matsunaga sp. nov.

*Diagnosis* – Megasporangiate cones elliptical, bearing 120 densely packed, helically arranged ovuliferous complexes. Ovuliferous scales bifacially flattened, cordiform to deltoid, distally lobed. One lateral lobe in each side of the scale and multiple medial lobes in multiple layers. Single inverted seed embedded in the ovuliferous scale tissues. Seed round to elliptical.

*Arkansia axsmithii* Andruchow-Colombo et Matsunaga sp. nov.

Figs 1–3.

**Fig. 1.**
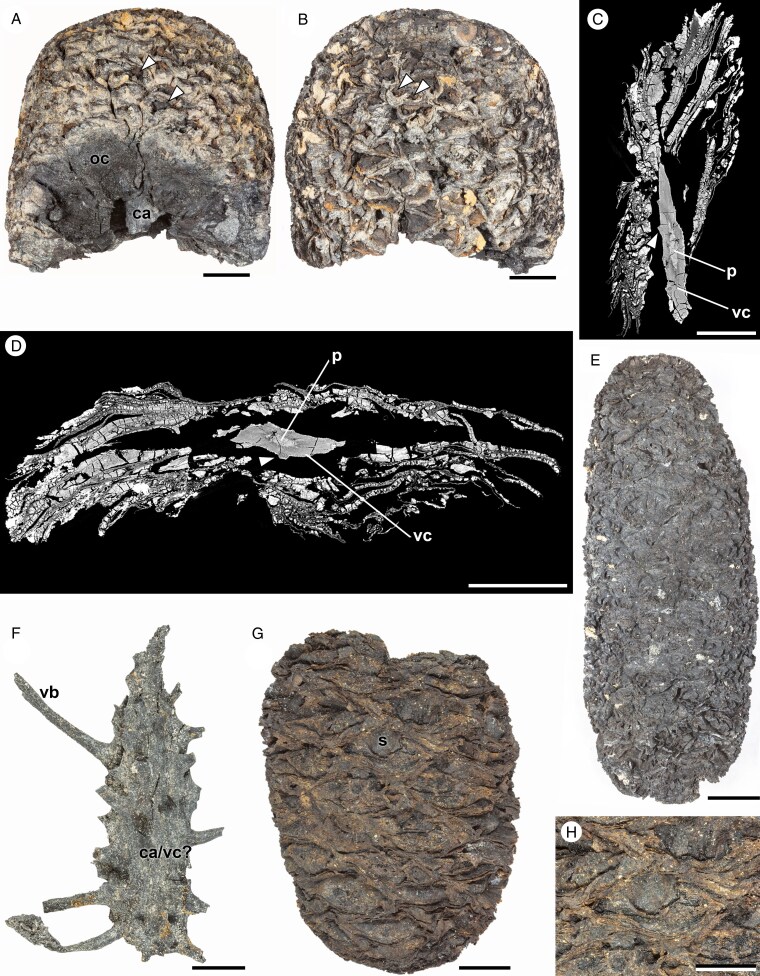
Seed cone morphology of *Arkansia axsmithii* sp. nov. (*Arkansia* plant). (A–D) C2596. (A, B) Two sides of the cone compression partially preserved. Note the fleshy appearance of the ovuliferous scale lobes (arrowheads). (A) Side of the broken cone exposing middle ovuliferous complexes (oc) and cone axis (ca). (C) Longitudinal/oblique section of the cone obtained from a μCT scan showing central axis. The cortex is not visibly preserved. Notice the lignified vascular cylinder (vc), the pith (p) and the base of the vascular bundle of an ovuliferous complex (arrowhead). (D) Cross-section of the cone obtained from a μCT scan showing central axis: cortex not preserved, lignified vascular cylinder (vc), and pith (p). Base of the vascular bundle of an ovuliferous complex is marked by an arrowhead. (E) C2598, seed cone completely preserved. (F) C2602, cone axis with persistent vasculature of the ovuliferous complexes emerging helically. (G) C2593, decorticated, partially preserved seed cone showing the impression of a seed (S) embedded in the ovuliferous scale tissues. (H) Detail of (E) showing the ovuliferous complex bearing an embedded seed. Scale bars (A–D) = 5 mm; (E) = 6 mm; (F), (H) = 3 mm; (G) = 4 mm.

**Fig. 2.**
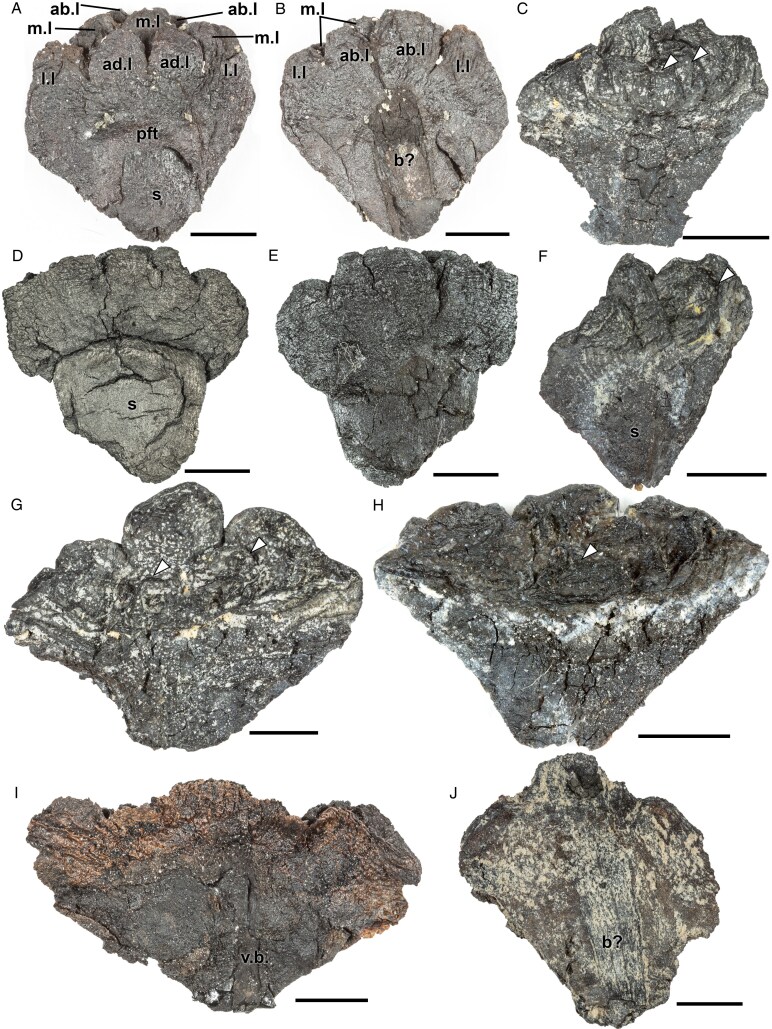
Ovuliferous scale morphology of *Arkansia axsmithii* sp. nov. (*Arkansia* plant). (A) C2591. Adaxial surface of ovuliferous scale showing multiple lobes: two lateral lobes (l.l), two adaxial lobes (ad.l), three middle lobes (m.l) and two abaxial lobes (ab.l), as well as the pocket-forming tissue (pft) partially covering the single inverted seed (s). (B) C2591. Abaxial surface of ovuliferous scale showing multiple lobes and a potential impression of the bract (b?) on the scale tissues. (C) C2607. Ovuliferous scale showing multiple lobes, some of which are preserved more tridimensionally (arrowheads). Note the shrivelled appearance of the distal lobes, suggesting fleshiness. (D) C2597. Partially preserved ovuliferous scale in adaxial view bearing a partially preserved seed (S) and the distal lobed area. (E) C2597. Partially preserved ovuliferous scale in abaxial view showing the distal lobed area. (F) C2600. Ovuliferous scale in adaxial view showing a single inverted seed (S) and a distal lobed area with some of the lobes tridimensionally preserved (arrowhead). Note the shrivelled appearance of the distal lobes, suggesting fleshiness. (G, H) C2595 and C2592. Ovuliferous scales with multiple distal lobes, some of which are tridimensionally preserved (arrowheads). Note the shrivelled appearance of the distal lobes, suggesting fleshiness. (I) C2594. Ovuliferous scale showing distal lobed area and exposing the vasculature (v.b.) towards its base. (J) C2605. Ovuliferous scale in abaxial side showing potential impression of the bract (b?). Scale bars (A–F) = 3 mm; (G–J) = 2 mm.

**Fig. 3.**
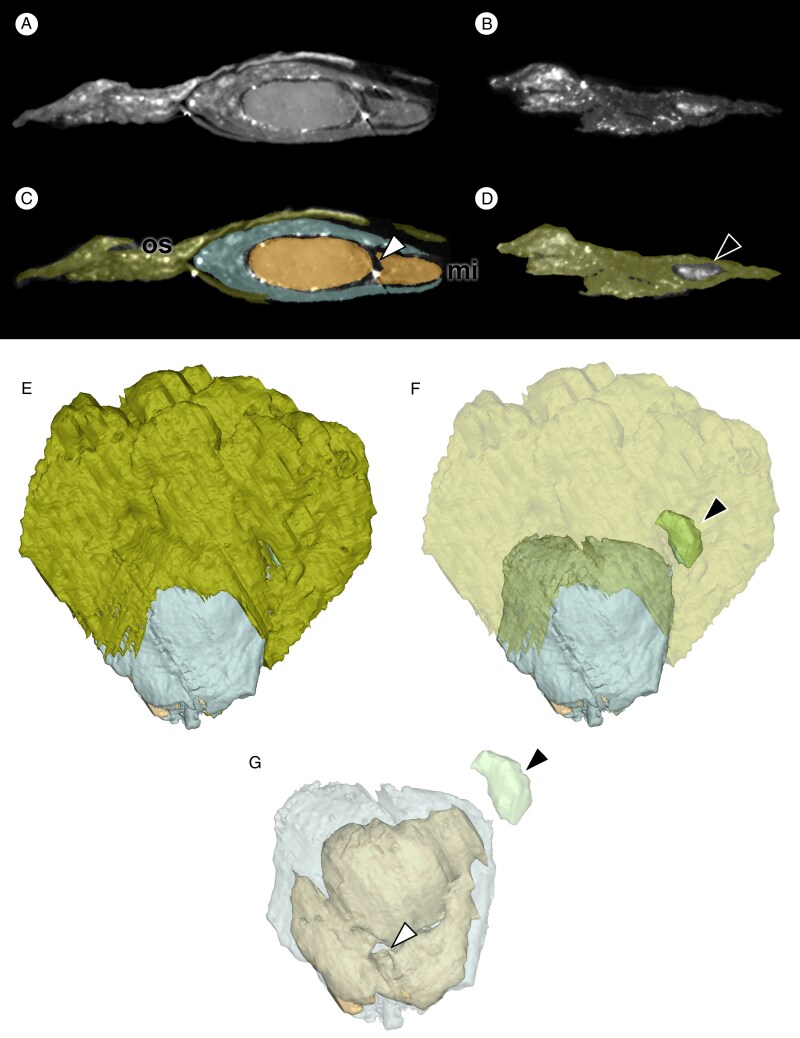
Ovuliferous scale morphology of *Arkansia axsmithii* sp. nov. (*Arkansia* plant) from µCT scan reconstructions of the specimen C2591. (A) µCT-reconstructed image of an oblique longitudinal section of the ovuliferous scale towards its middle showing the seed embedded in the scale tissue. (B) µCT-reconstructed image of an oblique longitudinal section of the ovuliferous scale towards one of its sides showing a second body embedded in the scale tissue, here interpreted as an aborted seed. (C) Image in (A) with the ovuliferous scale tissue tinted in dark yellow green, the seed integument in light blue, and the seed content in light orange; arrowhead shows an interruption of the seed content reconstructed in (G, white arrowhead). os, ovuliferous scale; mi, micropyle. (D) Image in (B) with the ovuliferous scale tissue tinted in dark yellow green; the aborted seed is tinted in a lighter green and marked with a black arrowhead, corresponding to the bean-like structure reconstructed in (F) and (G) (black arrowheads). (E) Reconstruction of the ovuliferous scale from µCT scan showing the scale in dark yellow-green, the seed coat in light blue and part of seed content in light orange. (F) reconstruction from (E) with the ovuliferous scale translucid showing the central, inverted seed in light blue and a bean-like structure (arrowhead) interpreted as an aborted seed. (G) Seed coat translucent showing the seed contents in light orange; white arrowhead marks interruption in seed content also seen in (B); black arrowhead marks the putative aborted seed.

*Etymology* – The name honours the late Dr Brian J. Axsmith for his significant contributions to the collection and study of this fossil taxon, the extinct family Cheirolepidiaceae and the fossil record of plants more broadly.

*Holotype here designated* – C2591, Figs 2A, B and 3.

*Paratypes* – C2592–C2598.

*Referred material* – C2599–C2611, C2612, C2613.

*Plant Fossil Names Registry Number –* PFN003615.

*Diagnosis* – Megasporangiate cones elliptical, bearing ∼120 densely packed, helically arranged ovuliferous complexes. Ovuliferous scales bifacially flattened, cordiform to deltoid, distally lobed. Nine lobes per ovuliferous scale: one lateral lobe in each side of the scale and seven medial lobes in three layers (two abaxial, three medial, two adaxial). Single inverted seed embedded in the ovuliferous scale tissues. Seed round to elliptical.

*Holotype description* – Isolated ovuliferous scale 10.8 mm long, 1.8 mm wide at its base and 10.4 mm wide at its widest point, which is located at 65.7 % of the total length of the scale. Ovuliferous scale cordiform to deltoid, distally lobbed with seven distal lobes (see description for lobe arrangement). The scale bears a single inverted, well-developed seed partially covered by the scale and a reniform body adjacent to the chalaza, smaller than the seed and completely embedded in the scale tissue. The well-developed seed is 5.1 mm long and 3.5 mm wide.

*Description –* The megasporangiate cones are elliptical, 19–33 mm wide when compressed (mean 24.2 mm, s.d.: 5.9 mm, *n* = 5) and ∼51.8 mm long, bearing ∼120 densely packed and helically arranged ovuliferous complexes (Fig. 1). Cone axes measure 1.4–4.9 mm in diameter (mean 3.1 mm, s.d.: 1.8 mm, *n* = 3). Isolated ovuliferous complexes and naked cone axes occur frequently (Figs 1F and 2).

Naked seed cone axes retain attached spirally arranged appendices interpreted to be the vascular bundle of the ovuliferous complexes (Fig. 1F). The cone axis has a central pith surrounded by secondary xylem and the cortex is inconspicuous or not preserved (Fig. 1C, D). Lateral vascular bundles also seem to be lignified and measure 0.3–0.7 mm in diameter and have been observed to measure up to 6.4 mm long (Fig. 1F), although they are usually broken closer to the cone axis. At their divergence from the cone axis, they are more or less circular in cross-section and become wider and flatter distally (Fig. 1D, bottom left). No evidence of branching of the vascular bundle was observed but, due to the fragility of the material, smaller branches are unlikely to be preserved.

In some specimens, an elongated structure or scar is present on the abaxial side of the ovuliferous scale, which could correspond to a fused or partially fused bract (Fig. 2B, J, indicated as ‘b?’). Ovuliferous scales are bifacially flattened, cordiform to deltoid in shape, and lobed distally (Figs 2 and 3E). The ovuliferous scale has a total of approximately nine lobes. Two of the lobes are lateral, one on each side, and occur on the same plane. Between the lateral lobes are approximately seven lobes arranged in three layers: two abaxial, three medial and two adaxial (Figs 2A, B and 3F). Due to their thick but often shrivelled appearance, the distal portion of the lobes may have been fleshy (Fig. 1B, arrowheads; Fig. 2). Ovuliferous scales measure 5.8–11.1 mm long (mean 8.3 mm, s.d. 1.5 mm, *n* = 12), 1–2.8 mm wide at its base (mean 2.1 mm, s.d. 0.6 mm, *n* = 10) and 4.5–11.7 mm at its widest point (mean 9.1 mm, s.d. 1.7 mm, *n* = 18), which is located at 55–81.1 % of the total length of the ovuliferous scale (mean 65.7 %, s.d. 7.9 %, *n* = 12), and 2.6–5.7 mm high (mean 3.8 mm, s.d. 1.1 mm, *n* = 6). *Classopollis* pollen grains were observed in the distal region of an ovuliferous scale between the lobes (Fig. 7D).

Each ovuliferous complex bears one inverted seed that is round to elliptical in shape, embedded in the ovuliferous scale tissues (Figs 1E, F, 2A, D, F and 3A, B, E, F). The seed is 5.1–7 mm long (*n* = 2), 1.6–6.7 mm wide (mean 3.2 mm, s.d. 2.1 mm, *n* = 5), and 1.2–2 mm high (mean 1.6, s.d. 0.4, *n* = 3; Fig. 3F, G). A µCT-scanned ovuliferous complex revealed a reniform structure positioned near the seed chalaza and significantly smaller than the seed (Fig. 3D, F, G, black arrowheads).

*Notes* – The distal lobes of the ovuliferous scales appear to have been thick (Fig. 1B, 2C, F, G, H). Due to their often shrivelled (Figs 1B, arrowhead, and 2), crushed or warped (Fig. 2C, F, G, H) preservation, we interpret this distal area to have been fleshy rather than coriaceous. The reniform structure observed in the µCT scan of an ovuliferous complex (Fig. 3B, D, F, G) is here interpreted as an aborted seed. This interpretation is based on the observed density of the structure under the µCT scan, which was similar to that of the seed (Fig. 3A, B), as well as on its morphology and position adjacent to the chalaza. Other Cheirolepidiaceae species have one or two seeds per ovuliferous complex (Escapa *et al.*, 2012), and therefore the presence of an aborted second ovule is plausible. While all ovuliferous complexes with preserved seeds have only one mature seed, the µCT-scanned specimen suggests the *Arkansia* plant may have had latent or remnant potentiality to produce two seeds.

*Genus – Pseudofrenelopsis* Nathorst, 1893.

*Type species – Pseudofrenelopsis felixii* Nathorst, 1893.

*Species – Pseudofrenelopsis axsmithii* Andruchow-Colombo et Matsunaga sp. nov.

Figs 4 and 5.

**Fig. 4.**
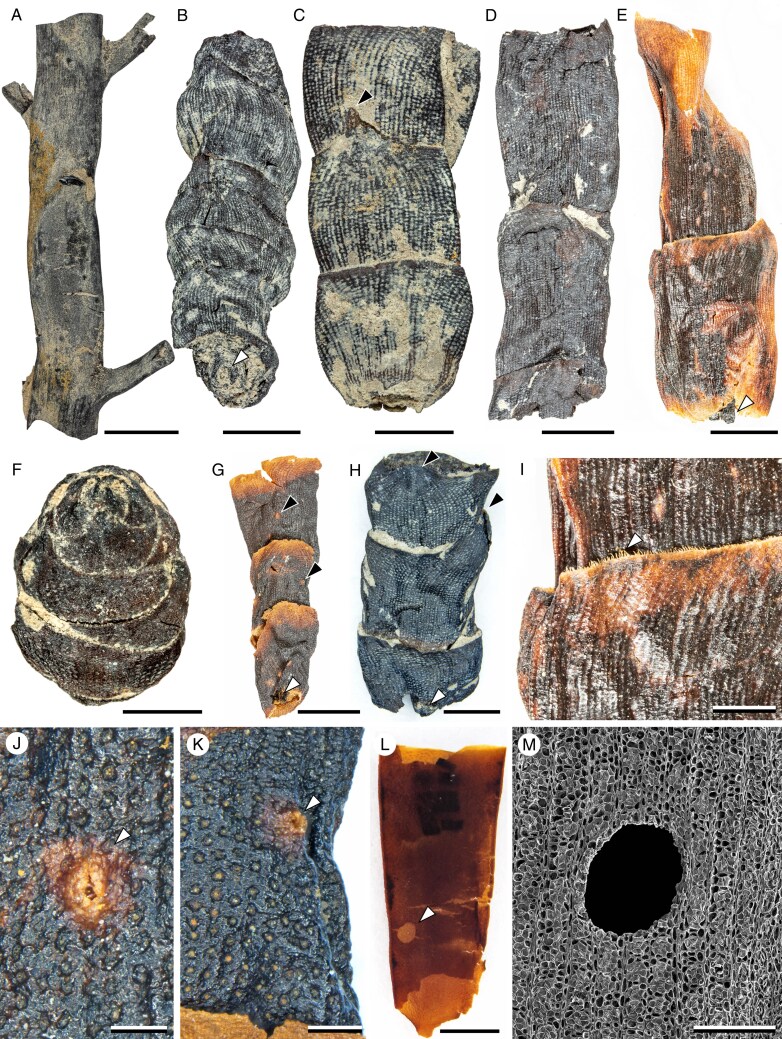
Leafy shoot and leaf morphology of *Pseudofrenelopsis axsmithii* sp. nov. (*Arkansia* plant). (A) C2628. Naked shoot with spirally inserted branches. (B) C2614. Leafy shoot showing three leaves; white arrowhead shows bearing branch. (C) C1232 (holotype). Leafy shoot bearing three leaves. Observe the acute apex of the middle leaf (arrowhead); stomatal rows can be easily distinguished. (D) C2615. Leafy branch showing two complete sheathing leaves and the apical zone of a third (lower) one. (E) C2619. Leafy branch showing two partially preserved sheathing leaves and part of the (much thinner) bearing branch (arrowhead). (F) C1235. Apical portion of a leafy branch showing the helical arrangement of the leaves. (G) C2620. Leafy branch with insect damage (black arrowheads, see details in J, K); white arrowhead shows bearing branch. (H) C2621. Leafy branch with spiral phyllotaxy, apices of two leaves indicated with black arrowheads; white arrowhead shows bearing branch. (I) C2619. Detail of the margin of one of the leaves of the branch in (E); arrowhead indicates a portion preserving marginal hairs. (J, K), C2620. Detail of insect damage on leaves from (G) that could correspond to galling or piercing and sucking types. (L) C2626. Leaf with a circular perforation (DT01) corresponding to hole feeding damage (indicated with a white arrowhead). (M) C2622. Detail of a circular perforation of a leaf (DT01) corresponding to hole feeding damage. Scale bars (A) = 7 mm; (B, D, E, G) = 3 mm; (C, H, L) = 2 mm; (F) = 1.5 mm; (I) = 1 mm; (J) = 0.2 mm; (K, M) = 0.3 mm.

**Fig. 5.**
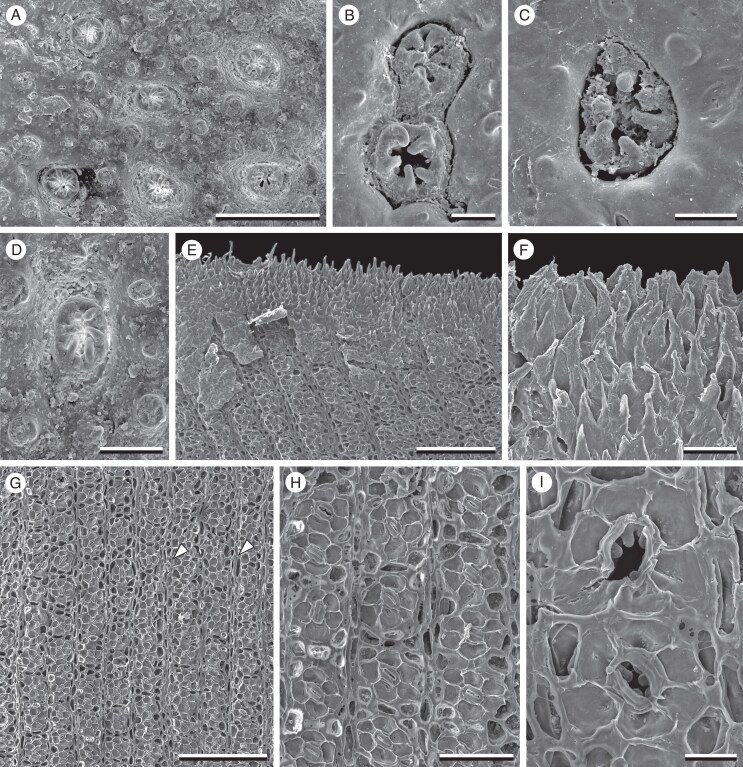
(A–I) Leaf cuticle morphology of *Pseudofrenelopsis axsmithii* sp. nov. (*Arkansia* plant). (A) C2623. External view of a portion of the leaf cuticle showing papillate stomata with well-developed Florin rings and globose papillae in non-stomatal zones. (B) C1245. Detail of two sunken stomata with the elongated papillae oriented towards the stomatal aperture. (C, D) C1245 and C2623 respectively. details of sunken stomata with stomatal papillae surrounded by epidermal papillae. (E) C2622. Cuticle of leaf apical portion showing part of the internal cuticle below and part of the leaf apical margin covered in hairs on the top. (F) C2622. Detail of hairs. (G) C2622. Internal view of a portion of the leaf cuticle showing multiple rows of stomata separated by one or two rows of epidermal cells (arrowheads). (H) C2622. Detail of three stomatal rows showing the randomly oriented stomatal apertures. (I) C2622. Detail of two stomata. Stomatal papillae can be seen through the stomatal apertures. Scale bars (A) = 100 µm; (B–D, I) = 30 µm; (E, G) = 300 µm; (F, H) = 50 µm.

*Holotype here designated* – C1232, Fig. 4C.

*Paratypes –* C1233, C1235, C1242, C1245, C2614–C2616, C2618–C2626,

*Referred material –* C1238,C1239, C1240, C1241,C1244, C2617, C2627, C2628, slide SL-15678.

*Etymology –* The name honours the late Dr Brian J. Axsmith for his significant contributions to the collection and study of this fossil taxon, the extinct family Cheirolepidiaceae, and the fossil record of plants more broadly.

*Plant Fossil Names Registry Number* – PFN003616.

*Diagnosis –* Leafy shoots. Branch arrangement helical. Phyllotaxis helical; single leaf per node with closed sheath and free tip overlapping subsequent node, giving nodes a slightly expanded appearance. Leaf length 3.5–15.4 mm; leaf sheath length 2.7–10.6 mm long, corresponding to most of total leaf length; distal margin and distal portion of adaxial side densely toothed. External side of cuticle papillate. Epidermal papillae globose. Well-developed Florin rings; five to seven stomatal papillae. Stomatal papillae cylindrical. Stomata in continuous rows; apertures randomly oriented. Neighbouring stomata from same row separated by 0–3 ordinary epidermal cells. Contiguous stomatal rows are separated by one or two rows of ordinary epidermal cells. Stomatal apparatuses with four to seven subsidiary cells, most commonly five or six, not forming an even ring.

*Holotype description –* Leafy shoot 9.4 mm long, bearing three consecutive nodes, each bearing a single sheathing leaf 3.54.4 mm long, with acute apex (76°). Stomata arranged in rows on the adaxial (i.e. exposed) leaf side.

*Description –* Shoot fragments are up to 49 mm long. Naked branches are 2.4–8.6 mm wide (mean 5.1 mm, s.d. 2.6 mm, *n* = 10) and specimens with up to five lateral branches arranged helically on a single branch were observed (Fig. 4A). Leafy branches have helical phyllotaxis (Fig. 4F, H). Each leaf has a sheathing and decurrent leaf base that comprises most of the leaf length, extending along the entire internode (Fig. 4B–E, G, H), and a free tip that overlaps with the following node resulting in the appearance of slightly expanded nodes (Fig. 4B, C, H). In all specimens but one, the sheathing leaf base lacks a suture (‘closed’ leaf type *sensu*Watson, 1977; Fig. 4B–E, G, H, L). However, one specimen of a shoot apex seems to have open leaves (i.e. sheaths not completely closed; *sensu*Watson, 1977; Fig. 4F). Leafy branches are 4.3–9.3 mm wide (mean 6 mm, s.d. 1.5 mm, *n* = 17). Total leaf length is 3.5–15.4 mm long (mean 8.5 mm, s.d. 3.2, *n* = 34 leaves from 17 leafy shoots) and leaf sheath is 2.7–10.6 mm long (mean 8.1 mm, s.d. 3 mm, *n* = 13 leaves from 8 leafy shoots), corresponding to 72.6–88.9 % of the total leaf length (mean 79.4 %, s.d. 4.7 %, *n* = 13 leaves from 8 leafy shoots). Leaf apex is acute to slightly obtuse (76–131°, mean 112 °, s.d. 19°, *n* = 8). The distal leaf margin has a row of fine trichomes (Fig. 4I) and the distal portion of the adaxial side is densely toothed (Fig. 5E, F). Leaf sheaths are significantly wider than the stems on which they are borne (Fig. 4B, E, G, F) and become easily detached when not filled with sediment, indicating that they were considerably fleshy.

The external side of the abaxial cuticle is papillate (Fig. 5A–D). The epidermal papillae are globose and 8–26 µm in diameter (Fig. 5A, C, D). Stomata have well-developed Florin rings, which are 36–64 µm in diameter and are surrounded by a depression (Fig. 5A–D). Elongate papillae grow from the Florin ring towards the aperture (Fig. 5B–D). There are five to seven stomatal papillae per stomatal apparatus, which are cylindrical, 8–19 µm long and 3–9 µm in diameter (Fig. 5B–D).

The inner side of the abaxial cuticle shows a high density of stomata, which occur in continuous rows along all the leaf width, with stomatal apertures randomly oriented (Fig. 5G–I). Neighbouring stomata within a single row were never observed to share subsidiary cells; they are separated by 0–3 ordinary epidermal cells that are usually oval with their major axis perpendicular to the leaf longitudinal axis (Fig. 5G–I). Neighbouring stomatal rows are separated by one or two rows of oval, elongated ordinary epidermal cells, of which the long axis is parallel to the leaf longitudinal axis (Fig. 5H). Stomatal rows are 73–102 µm wide; epidermal rows that separate stomatal rows are 16–28 µm wide. Stomatal apparatuses exhibit four to seven subsidiary cells (most commonly five or six) not forming an even ring. The stomatal apparatus is 57–80 µm long (considering the long axis as the one that aligns with the stomatal aperture) and 50–69 µm wide. Subsidiary cells measure 18–34 µm between radial stomatal walls (mean 25.8 µm, s.d. 6 µm; *n* = 9) and 18–30 µm between tangential stomatal walls (mean 22.5 µm; s.d. 3.8 µm, *n* = 9). Ordinary epidermal cells within stomatal rows are 11–28 µm long (mean 17.0 µm; s.d. 6.2 µm; *n* = 7) and 19–28 µm wide (mean 23.7 µm; s.d. 3.7 µm; *n* = 7), while ordinary epidermal cells between stomatal rows are 27–66 µm long (mean 42.3 µm; s.d. 14.2 µm; *n* = 7) and 5–11 µm wide (mean 8.8; s.d. 2.1 µm; *n* = 7).

#### Insect damage

Circular to subcircular perforations measuring 0.5 and 0.9 mm in diameter were observed in two specimens (Fig. 4L, M) and would correspond to the hole-feeding insect damage type DT01 (Labandeira *et al.*, 2007). Additionally, one specimen shows two instances of circular to ellipsoidal damage that are 0.4 and 0.1 mm in diameter, have an elevated rim with a central aperture that is round or elongated, and a discoloration of the cuticle around it (Fig. 4J, K). These are similar in morphology to blister galls illustrated by Donovan *et al.* (2023, Figs 13C and 18O, Q) for extant *Agathis* and somewhat comparable with DT32 (Labandeira *et al.*, 2007). They are also comparable with piercing and sucking damage type DT132 (Labandeira *et al.*, 2007).

### Associated pollen cones and pollen

*Species – Classostrobus arkansensis* Axsmith, *et al.* (2004*b*).

*Holotype (designated by* *Axsmith et al., 2004*b *) –* C1231, SL-15677 (location unknown), SL-15679 (location unknown), SL-20578, C-1230.

*Paratypes (designated by* *Axsmith et al., 2004*b *) –* C1228, C1237.

*Referred material (by* Axsmith *et al.*, 2004*b* *) –* C1229, C1236, C-1237, SL-20577.

*Additional referred material (here designated) –* C2629–C2633, C2634 (slides 1–5).

Plant Fossil Names Registry Number – PFN003130

**Fig. 6.**
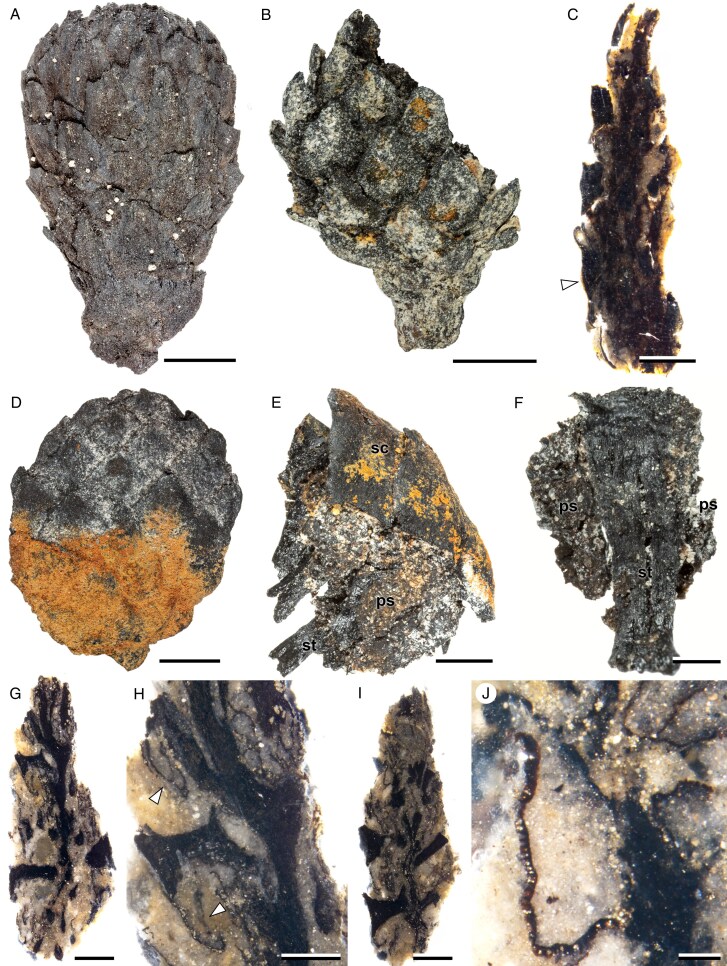
Pollen cone morphology of *Classostrobus arkansensis* (*Arkansia* plant). (A) C-1228. General morphology of pollen cone attached to short axis showing globose outline and spirally arranged microsporophylls. (B) C2631. Partially preserved pollen cone showing its base and spirally arranged microsporophyll. (C) C2634, slide 1. Longitudinal section of pollen cone showing peltate microsporophyll (arrowhead). (D) C2630. Globose pollen cone attached to short axis with spirally arranged microsporophylls. (E) C2633. Detached microsporophylls showing the stalks (st), rhombic scutellum (sc) and possible pollen sacs (ps). (F) C2633. Adaxial view of partially preserved microsporophyll with central stalk (st) and two abaxial elongated pollen sacs (ps). (G, I) C2634, slides 3 and 4. Longitudinal sections of pollen cone. (H) C2634, slide 3. Detail of (G) showing two microsporophylls with abaxial side of scutellum lamina curved inwards (arrowheads), possibly representing part of the pollen sac. (J) C2634, slide 4. Detail of (I); pollen sac wall showing encompassing cells with U-shaped thickenings. Scale bars (A, B, D) = 2 mm; (C, E, G, I) = 1 mm; (F, H) = 500 µm; (J) = 100 µm.

**Fig. 7.**
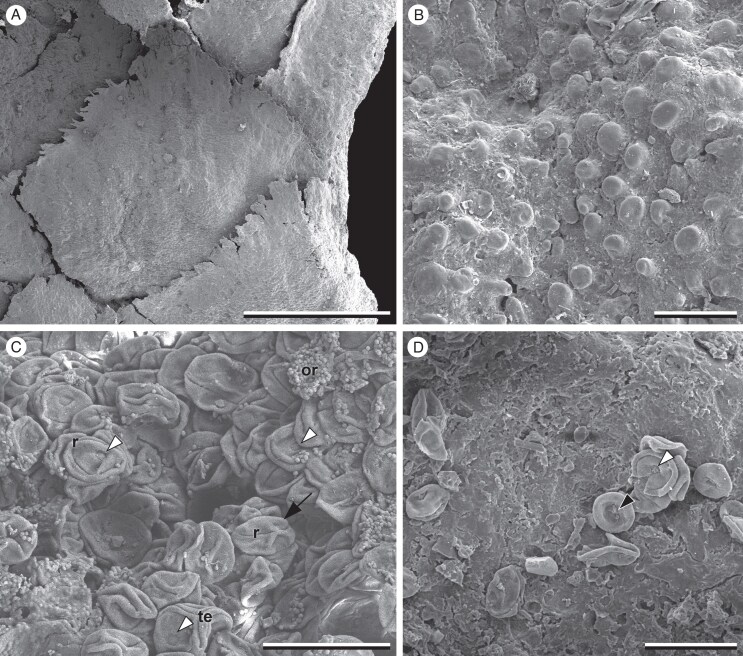
Pollen cone and pollen morphology of *Classostrobus arkansensis* (*Arkansia* plant) under SEM. (A) C-1236. Microsporophyll scutellum showing serrated margin. (B) C-1236. Detail of scutellum abaxial external epidermis showing round papillae. (C) C-1230. Pollen mass of *Classopollis* showing tetrads (te) and isolated pollen grains; notice pollen grain in equatorial view (black arrow) with subequatorial rimula (r); cryptopores of different pollen grains are marked with arrowheads. or, orbicules. (D) C2594. *Classopollis* pollen grains found on the distal region of an ovuliferous scale; one shows a cryptopore at the distal pole (white arrowhead) and the other shows a triangular scar at the proximal pole (black arrowhead). Scale bars (A) = 1 mm, (B–D) = 50 µm.

*Description.* Microsporangiate cones are globose to elliptical, 5.9–13.8 mm long (mean 10.3 mm, s.d. 2.8 mm, *n* = 8) and 4.3–10 mm wide (mean 6.7 mm, s.d. 1.8 mm, *n* = 8), with a length to width ratio of 1–2.4 (mean 1.6, s.d. 0.5, *n* = 8) and ∼55–60 densely packed microsporophylls (Fig. 6A–E). Microsporophylls are peltate (Fig. 6C, E, F) and have a thin stalk and a rhombic scutellum (Fig. 6B, D, E). The scutellum is 1.7–3.4 mm long (mean 2.4 mm, s.d. 0.5 mm, *n* = 15) and 1.4–2.9 mm wide (mean 2.1 mm, s.d. 0.4 mm, *n* = 14), peltate, bearing apparently two elongated abaxial pollen sacs 1.8–1.9 mm long (*n* = 2; Fig. 6E, F) and 0.6–1 mm wide (*n* = 2). The stalk is 2.4–2.5 mm long (*n* = 2) and ∼0.6 mm wide. The walls of the pollen sacs, observed in pollen cone sections, show cells with characteristic U-shaped thickenings (Fig. 6J). The margins of the scutellum are serrated (Fig. 7A) and its epidermis is papillate (Fig. 7B).

Pollen grains of the *Classopollis* type were found in the pollen cones (Fig. 7C) and in the distal region of ovuliferous scales (Fig. 7D). Pollen grains are circular in polar view (Fig. 7C, D) with a diameter of 23.8–28.3 µm (mean 25.6 µm, s.d. 1.7 µm, *n* = 7), they are subcircular in lateral view (Fig. 7C, black arrow), have a circular cryptopore in the distal pole that is 2.9–5.5 µm in diameter (mean 4.4 µm, s.d. 0.9 µm, *n* = 8; Fig. 7C, D, white arrowheads), and a triangular scar with concave sides marking the centre of a trilete mark in the proximal pole (Fig. 7D, black arrowhead). The triangular scar is 6–8 µm high (*n* = 2). Pollen grains show a subequatorial groove on the distal side (rimula, Fig. 7C, D).

*Note –* The epidermal and stomatal morphology of the pollen cones of *Classostrobus arkansensis* was characterized by Axsmith *et al.* (2004b) and is not revisited here.

### Phylogenetic analysis

**Fig. 8.**
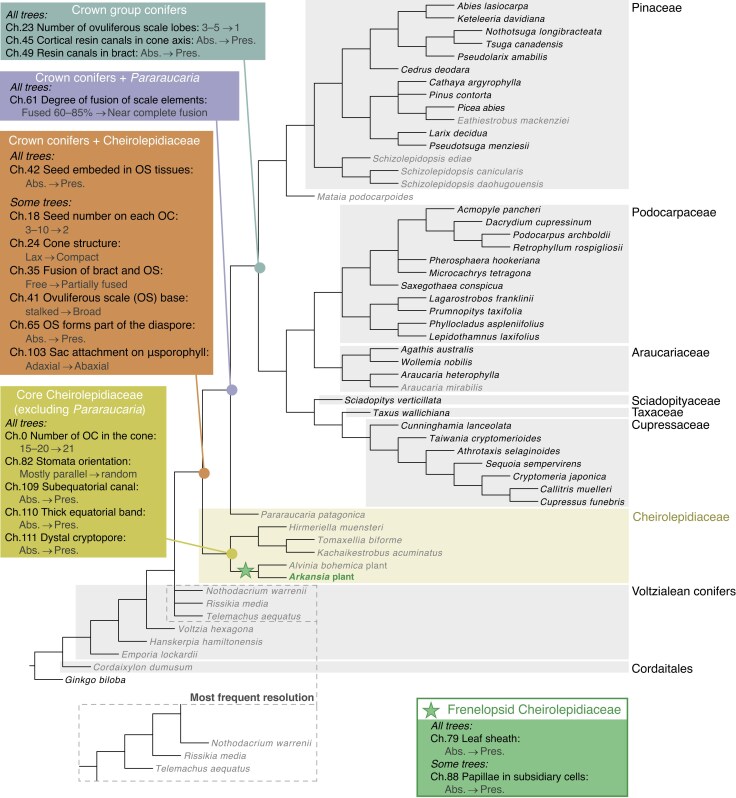
Metaconsensus of the 48 analyses of extended implied weights. Fossil species are shown in grey and the *Arkansia* plant here described is in bold green font. Colour text boxes indicate the synapomorphies of nodes of interest. Ch, character.

**Fig. 9.**
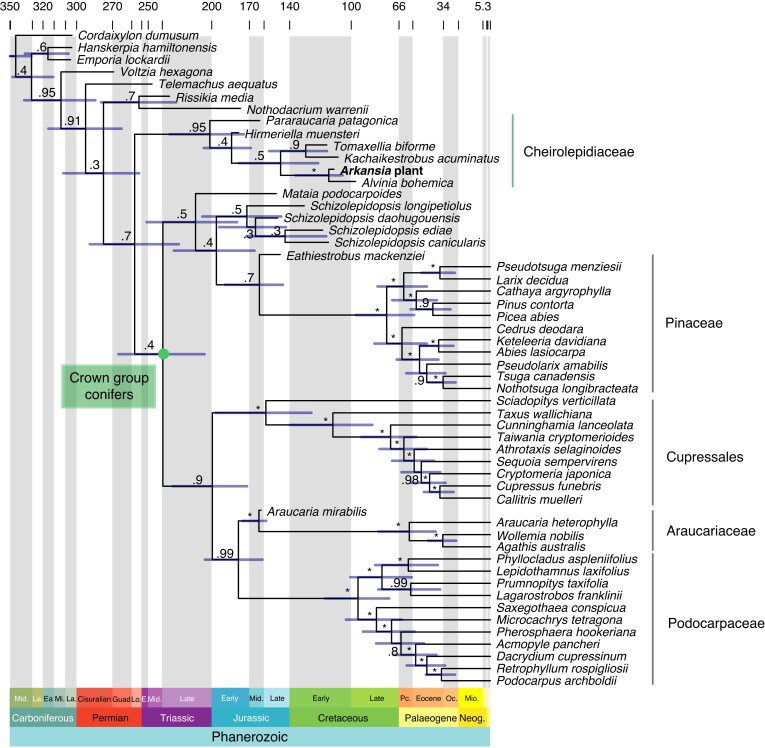
Time-calibrated tree from FBD analysis. Bars at the nodes indicate the age 95 % highest posterior density (HPD) interval. Numbers over the branches indicate posterior probabilities (pp); asterisks mark a posterior probability of 1. The scale on top of the phylogeny corresponds to age in Millions of Years before the present.

Most of our phylogenetic reconstructions recover the new whole plant from Arkansas as sister to *Alvinia bohemica*, a Cheirolepidiaceae species from the Late Cretaceous of the Czech Republic (Figs 8 and 9, Supplementary Data Appendix S2C, but see Supplementary Data Appendix S3A). These two species are always within a clade that also encompasses *Hirmeriella*, *Tomaxellia* and *Kachaikestrobus*, which we will refer to as the ‘core Cheirolepidiaceae’. In some analyses *Pararaucaria* is also part of this clade. When *Pararaucaria* is not recovered within the core Cheirolepidiaceae, it is one node away (Fig. 8, Supplementary Data Appendix S2Ai p. 29). *Pararaucaria patagonica* is the only Cheirolepidiaceae in the matrix that is represented by a single organ (i.e. anatomically preserved seed cones; Table 1). Leaves and pollen, unknown for *Pararaucaria patagonica*, are structures with highly characteristic traits that provide synapomorphies for core Cheirolepidiaceae (Fig. 8). Moreover, *Pararaucaria patagonica* has nearly complete fusion of its ovuliferous scale elements, while the other Cheirolepidiaceae have a degree of fusion of 60–85 % of the total length of the scale (Fig. 8). However, the *PlaceMyFossils* analysis, performed to explore this unexpected positioning of *Pararaucaria*, indicates that while the most parsimonious position of *P. patagonica* is a node away from core Cheirolepidiaceae, its placement within core Cheirolepidiaceae is only one step less parsimonious. Conversely, the placement of *Pararaucaria* anywhere else in the tree involves between 3 and 16 extra steps (see Supplementary Data Appendix S5).

**Table 1.** Fossil records of Cheirolepidiaceae that preserve either multiple organs or seed cone anatomy.

Taxon	Vegetative	Leaf cuticle	Pollen cones	Pollen	Seed cones	Isolated ovuliferous complexes	Region	Age	Citation
*Pseudohirmeriella delawarensis*	T, LS	–	X*	–	X*	X*	New Jersey, USA	Middle Norian	Axsmith *et al.* (2004*a*)
*Hirmeriella muensteri*	LS	X	X	X	X	X	UK; Germany; Poland	Jurassic	Hörhammer (1933); Jung (1968); Clement-Westerhof and Konijnenburg-van Cittert, (1991); Barbacka *et al.* (2007)
*Pararaucaria patagonica*	–	–	–	–	X	–	Patagonia, Argentina	Jurassic	Calder (1953); Stockey (1977); Escapa *et al.* (2012)
*Pararaucaria taquetrensis*	LS	–	–	–	X	X	Patagonia, Argentina	Pleinsbachian to Toarcian	Escapa and Leslie (2017)
*Pararaucaria carrii*	–	–	–	–	X	–	Oregon, USA	Callovian	Stockey and Rothwell (2013)
*Pararaucaria delfueyoi*	*–*	*–*	–	–	X	–	Patagonia, Argentina	Late Jurassic	Escapa *et al.* (2013)
*Pararaucaria collinsonae*	*–*	*–*	–	–	X	–	UK	Tithonian	Steart *et al.* (2014)
*Pseudofrenelopsis* plant (Isle of Wight)	T, LS	X	X	X	–	–	UK	Early Cretaceous	Watson (1977); Alvin *et al.* (1981); Alvin (1983)
*Pararaucaria laiyangensis*	LS	–	–	–	X	–	Shandong, China	Hauterivian–Barremian	Jin *et al.* (2023)
*Tomaxellia biforme*	LS	X	X	X	X	X	Patagonia, Argentina	Aptian	Archangelsky (1966, 1968), Archangelsky and Gamerro (1967)
***Arkansia* plant**	**L, T, F, LS**	**X**	**X**	**X**	**X**	**X**	**Arkansas, USA**	**Aptian/Albian**	Stanley (1988); Axsmith *et al.* (2004*b*); Axsmith (2006), Creen (2006), this work
*Frenelopsis* plant (Texas)	L, F, LS	X*	X	X	X*	X*	Texas, USA	Aptian/Albian	Axsmith and Jacobs (2005)
*Kachaikestrobus acuminatus*	LS	X	–	X	X	–	Patagonia, Argentina	Early Albian	Del Fueyo *et al.* (2008)
*Alvinia* plant	LS	X	X	X	X	X	Czech Republic	Cenomanian	Kvaček (2000)

Notes: Under Vegetative, L refers to leaves, T refers to twigs (no leaves preserved), F refers to fusain and LS refers to leafy shoots.

Under Leaf cuticle, Pollen cones, Pollen, Seed cones, and Isolated ovuliferous complexes,.

X means present/known, – means unknown and * indicates poor preservation.

The position of Cheirolepidiaceae varies between analyses, but they are most commonly recovered as sister to the crown group of conifers. This position was recovered as the single optimal option in all 48 analyses under extended implied weighted parsimony (Fig. 8) and in our FBD analysis (Fig. 9). On the other hand, the equal weight parsimony analyses (12 analyses in total, in which Voltziales and Cordaitales were excluded from the crown group) recovered the Cheirolepidiaceae as an unstable clade that was placed in three alternative positions (Supplementary Data Appendixes S2C and S2Bi). These alternative positions were as sister to Araucariales (12 out of the 12 analyses), as sister to the conifer crown group (8 out of the 12 analyses), as sister to the clade formed by Araucariales and Cupressales (6 out of the 12 analyses) (Supplementary Data Appendixes S2C and S2Bi). The non-clock Bayesian analysis recovered the Cheirolepidiaceae nested within the Araucariaceae crown group, as sister to *Araucaria*, and, in at least a 33 % of the posterior trees, they were positioned either as sister to Araucariaceae or to Araucariales (Supplementary Data Appendix S3A).

Our FBD analysis estimated a mean age of 342.9 million years (Middle Mississipian) for the divergence of Voltziales and Cordaitales and of 331.5 million years (late Middle Mississipian) for diversification of Voltziales, a mean age of 257.0 million years (late Lopingian) for the divergence of the crown group conifers and the Cheirolepidiaceae, of 202.7 million years (late Rhaetian) for the diversification of Cheirolepidiaceae, and of 236.6 million years (Carnian) for the diversification of the crown group of conifers (Fig. 9; Supplementary Data Appendix S4D). Ages within the crown group of conifers are provided in Supplementary Data Appendix S4D, but since most of the living families have few or no fossil members included in our analysis, we have low confidence in the age estimates for these clades and recommend caution in using this information.

## DISCUSSION

### Justification for transferring the Arkansas foliage to the new species *Pseudofrenelopsis axsmithii*

Stanley (1988) and Axsmith (2006) included the Arkansas vegetative material in the previously defined species *Pseudofrenelopsis parceramosa*. We here create a new species for the Arkansas vegetative material, justified in the major morphological and anatomical differences that separate it from previous records of *P. parceramosa.* Although the morphotaxon *Pseudofrenelopsis parceramosa* was reported from several localities across the world (Watson, 1977), we focus here on its three main occurrences: (1) the type material from the Potomac Group (Aptian–Turonian, VA, USA; Fontaine, 1889; Watson, 1977), (2) the material from the Barremian of the English Isle of Wight, which preserves wood, pollen cones and pollen in association with the leafy shoots (Watson, 1977; Alvin *et al.*, 1981; Alvin, 1983), and (3) the Aptian/Albian Arkansas material studied here (Stanley, 1988; Axsmith *et al.*, 2004*b*; Axsmith, 2006; this work).

Axsmith *et al.* (2004*b*) argued that the Arkansas *P. parceramosa* material does not belong to the same biological species as the type material of *P. parceramosa* or to the Isle of Wight occurrence. The Arkansas material can be differentiated based on characters of branch arrangement, epidermal morphology, leaf anatomy, anatomy of associated wood and the morphology of associated pollen cones (Axsmith *et al.*, 2004*b*). Stanley (1988) also recognized some of these morphological differences and even discussed some probable ecological differences between the Arkansas material and the *P. parceramosa* type material, based on differences in the reconstructed palaeohabitat of both plants. This was further discussed by Axsmith (2006), who also noted the long stratigraphic range of the morphospecies and the large intraspecific variation as evidence of the artificial status of the species *P. parceramosa*.

While previous authors recognized the multiple morphological differences between the main occurrences assigned to *P. parceramosa*, the Arkansas material was never separated into a new species. This was due to the lack of knowledge of reproductive organs associated with the type material of *P. parceramosa* and the subsequent uncertainty over whether vegetative characters correlated with differences in reproductive structures (Axsmith *et al.*, 2004*b*). However, we argue that there are enough vegetative differences to separate the Arkansas material from the type material, while the associated pollen cones and wood provided additional lines of evidence to reject conspecificity with the Isle of Wight material.

The branch arrangement in the original material of *P. parceramosa* is either alternate in one plane or whorled (Fontaine, 1889; Watson, 1977), whereas in the Arkansas specimens it is helical (Stanley, 1988; Axsmith, 2006; this work). The fossils from the Isle of Wight share the helical branch arrangement with the Arkansas material but differ in wood anatomy and pollen cone morphology (Watson, 1977; Axsmith *et al.*, 2004*b*; see below). Leaf sheaths in *Pseudofrenelopsis* can be open, sutured or closed (Watson, 1977). Open sheaths have a leaf base extended laterally, but not fully encircling the whole branch circumference; in sutured sheaths the leaf base completely surrounds the branch circumference, but a suture can be observed at the place where the leaf edges meet; and in closed sheaths the leaf base completely encircles the shoot and there is no trace of a suture (Watson, 1977, Text-Fig. 1c–e). *Pseudofrenelopsis parceramosa* occurrences tend to have different proportions of sheath types, but all three are consistently reported as present (Watson, 1977). Open or sutured leaf sheaths are absent in the Arkansas material, with the single exception of a young shoot apparently bearing open sheaths (Fig. 4 F). The emended diagnosis of *P. parceramosa* made by Watson (1977) states that the species has a well-developed cutinized hypodermis, which was not observed for the Arkansas material (Fig. 5E, G–I; Stanley, 1988; Axsmith, 2006). Finally, as noted by Axsmith (2006), the Arkansas material exhibits subsidiary cell papillae in all observed specimens regardless of their size, whereas Watson (1977) observed that in *P. parceramosa* subsidiary cell papillae tend to be absent in leaf sheaths longer than 5 mm.

Differences between the Arkansas and the Isle of Wight materials include the wood anatomy characters described by Axsmith (2006), notably the absence of abietinean pits on the radial tracheid walls and presence of relatively tall rays. Differences in pollen cone morphology between these fossil taxa include microsporophyll shape, as well as the length of marginal trichomes, thickness of cuticle, absence/presence of cutinized hypodermis and papilla shape in the microsporophyll head (Axsmith *et al.*, 2004b). Moreover, while still uncertain, the Isle of Wight pollen cones seem to bear three pollen sacs per microsporophyll (Alvin *et al.*, 1994), whereas the Arkansas species seems to bear two (Axsmith *et al.*, 2004b; this work). Based on these characters, we proposed the new species *Pseudofrenelopsis axsmithii* to encompass the Arkansas vegetative material previously included in Stanley (1988); Axsmith *et al.* (2004*b*) and Axsmith (2006).

Since the first descriptions of the *Pseudofrenelopsis* remains in Arkansas (Stanley, 1988; Axsmith, 2006), several new species have been described for this genus (Mendes *et al.*, 2023). However, none of these taxa exhibit the character combination described for *P. axsmithi*, supporting the decision to transfer the Arkansas foliage from *P. parceramosa* to the new species, *P. axsmithii* (see Supplementary Data Table S6, within Supplementary Data Appendix S6).

### Whole-plant concepts for Cheirolepidiaceae and comparisons with other taxa

There are currently no whole-plant concepts for any Cheirolepidiaceae species, but several taxa have multiple organs described in association (Table 1). The most complete Cheirolepidiaceae known to date, in terms of number of organs preserved, encompasses remains of *Frenelopsis ramosissima* from the Early Cretaceous of Texas (Axsmith and Jacobs, 2005; Table 1). This fossil taxon includes leafy shoots with fragmentary cuticle, associated pollen cones with pollen, ovulate cones, potential isolated ovuliferous complexes, fusinized wood and large logs without anatomical preservation (Axsmith and Jacobs, 2005). However, the seed cones and potential isolated ovuliferous complexes associated with *F. ramosissima* are poorly preserved and therefore do not provide substantial information on this aspect of reproductive morphology.

Other taxa are known from fewer organs, which nevertheless preserve key information on various aspects of Cheirolepidiaceae morphology (Table 1). These include *Tomaxellia biforme*, based on leafy shoots, pollen cones with *in situ* pollen, ovulate cones and isolated ovuliferous complexes from the Aptian–Albian of Argentina (Archangelsky, 1966, 1968; Archangelsky and Gamerro, 1967); *Hirmeriella muensteri*, based on leafy shoots, pollen and ovulate cones, isolated ovuliferous complexes and pollen from the Rhaetian–Toarcian of Germany (Hörhammer, 1933; Jung, 1968; Clement-Westerhof and Konijnenburg-van Cittert, 1991; Barbacka *et al.*, 2007); and *Alvinia bohemica*, based on ovulate cones and isolated ovuliferous complexes associated with leaves and pollen cones with *in situ Classopollis* pollen assigned to *Frenelopsis alata* from the Cenomanian of Czech Republic (Kvaček, 2000).

Other relatively complete Cheirolepidiaceae plants include leafy shoots from the Barremian of the Isle of Wight assigned to *Pseudofrenelopsis parceramosa* that were found in association with anatomically preserved wood and pollen cones with *in situ* pollen (Watson, 1977; Alvin *et al.*, 1981; Alvin, 1983); *Kachaikestrobus acuminatus*, based on leafy shoots with preserved cuticle, organically connected seed cones, and dispersed *Classopollis* adhered to ovuliferous scales, from the early Albian of Argentina (Del Fueyo *et al.*, 2008); and *Pararaucaria taquetrensis* described from impressions of leafy shoots, ovulate cones and isolated ovuliferous complexes from the Early Jurassic of Argentina (Escapa and Leslie, 2017).

In this context, the *Arkansia* plant is the most completely know Cheirolepidiaceae to date, with a similar number of organs known to the *Frenelopsis* plant described from Texas by Axsmith and Jacobs (2005), but with better preservation of its pollen cones, seed cones and isolated ovuliferous complexes. Among the aforementioned fossil taxa, *Arkansia* ovuliferous scales are most similar to those of *Hirmeriella* and *Kachaikestrobus*, all having two lateral lobes, and a series of central lobes in more than one row (Clement-Westerhof and Konijnenburg-van Cittert, 1991; Del Fueyo *et al.*, 2008). *Hirmeriella* and *Kachaikestrobus* have two rows of central lobes. In *Hirmeriella* the abaxial row has one lobe and the adaxial one has three and in *Kachaikestrobus* both rows have six lobes. In contrast, *Arkansia* has three distinct rows of central lobes: two abaxial lobes, three medial lobes and two adaxial lobes, for a total of seven central lobes. *Alvinia* has also multiple central lobes, one adaxial and two abaxial as in *Hirmeriella* (Kvaček, 2000), but does not have clear lateral lobes as do *Arkansia* and *Hirmeriella*. *Tomaxellia* has six lobes in at least two rows, but the details of their arrangement are not clearly preserved (Archangelsky, 1968). Three species of *Pararaucaria* preserve the distal region of the ovuliferous scales: *P. patagonica*, *P. taquetrensis* and *P. laiyangensis*. In all three species, ovuliferous scales have three lobes in a single row (Escapa *et al.*, 2012; Escapa and Leslie, 2017; Jin *et al.*, 2023). Seed number per ovuliferous scale is either invariably two, or variably one or two (Escapa *et al.*, 2013). In *Arkansia* we have only observed single-seeded ovuliferous complexes, although in one specimen a possibly aborted second seed was observed (Fig. 3D, F, G).

Overall, despite similarities in vegetative and reproductive morphology with previously described species, it is clear that the *Arkansia* plant constitutes a distinct fossil taxon, broadening the morphological diversity of the extinct family Cheirolepidiaceae and providing the most completely known plant for the family to date.

### On the nature of the frenelopsid lineage

The leaf morphology of the *Arkansia* plant is of the *Pseudofrenelopsis* type, with shoots bearing a single sheathing leaf per node in a helical phyllotaxis (Watson, 1982; Axsmith, 2006). While fifteen other *Pseudofrenelopsis* records have been previously described (Watson, 1977; Kvaček and Mendes, 2023), none are associated with seed cones and therefore it is unclear if they are all closely related to one another or to the *Arkansia* plant. The genus *Frenelopsis* also has sheathing leaves but, unlike *Pseudofrenelopsis*, is characterized by whorls of two or three leaves per node. *Frenelopsis* and *Pseudofrenelopsis* have been previously classified into an informal morphological group called the ‘frenelopsids’ based on their sheathing leaves and overall resemblance (Watson, 1982). However, since these two genera differ in their phyllotaxy and in how they produce the leaf sheaths (i.e. by lateral fusion of multiple leaves on the same node or by lateral fusion of a single leaf, respectively), it is unclear whether they represent a natural group or if sheathing leaf morphologies independently arose in different Cheirolepidiaceae lineages. Further information on other organs may be necessary to better understand these relationships.

In addition to *Arkansia*, two other frenelopsid taxa have associated foliage and ovulate cones. The *Frenelopsis* plant from Texas described by Axsmith and Jacobs (2005) has both vegetative and ovuliferous remains preserved, but the poor preservation of its ovuliferous structures hinders detailed comparisons with other taxa. The *Alvinia* plant from the Early Cretaceous of the Czech Republic has *Frenelopsis* leaves associated with ovulate cones. While the ovuliferous complexes of *Alvinia* are markedly different from those of *Arkansia* (see above), they were recovered together in our phylogenetic analyses (Figs 8 and 9), suggesting the existence of a frenelopsid clade. However, this clade is supported by leaf characters only (Fig. 8), and so future fossil discoveries that unite vegetative and ovulate structures into organismal concepts will be necessary to better understand the extent to which the frenelopsids truly are a natural group.

### Phylogenetic position of Cheirolepidiaceae and early morphological evolution of modern conifers

The affinities of Cheirolepidiaceae with other conifers have been elusive and contentious. The family as a whole or a subset of their genera have been associated with nearly every major group of conifers, including the families Araucariaceae (Calder, 1953; Jung, 1968; Escapa *et al.*, 2013; Stockey and Rothwell, 2013; Escapa and Leslie, 2017; Jin *et al.*, 2023), Cupressaceae (Calder, 1953; Stockey, 1977), Pinaceae (Calder, 1953; Stockey, 1977; Smith and Stockey, 2001, 2002; Escapa *et al.*, 2013) and Podocarpaceae (Hirmer and Hörhammer, 1934; Jung, 1968; Escapa *et al.*, 2013; Stockey and Rothwell, 2013; Matsunaga *et al.*, 2021); the living order Araucariales (Matsunaga *et al.*, 2021; Andruchow-Colombo *et al.*, 2023) and the extinct order Voltziales (Florin, 1951; Harris, 1957; Archangelsky, 1968; Jung, 1968; Stockey, 1981; Stanley, 1988; Clement-Westerhof and Konijnenburg-van Cittert, 1991; Miller, 1999; Kvaček, 2000; Andruchow-Colombo *et al.*, 2023); as well as the gnetophytes (Krassilov, 1987). These associations were mostly based on qualitative comparisons of morphological features, particularly seed cones, but occasionally also on vegetative and pollen organs. Three of these previous studies included phylogenetic analyses (Matsunaga *et al.*, 2021; Andruchow-Colombo *et al.*, 2023; Jin *et al.*, 2023). Analyses by Matsunaga *et al.* (2021) and Andruchow-Colombo *et al.* (2023) included a single Cheirolepidiaceae species, *Pararaucaria patagonica*, together with members of all major extant and extinct conifer groups, and were based exclusively on seed cone characters. Matsunaga *et al.* (2021) recovered *Pararaucaria* either as sister to Podocarpaceae or to the order Araucariales, while Andruchow-Colombo *et al.* (2023) recovered it either as sister to all conifers or as stem group Araucariales. The phylogenetic analysis of Jin *et al.* (2023) included seven Cheirolepidiaceae taxa together with living and fossil exemplars from all extant conifer families except Taxaceae. They recovered the Cheirolepidiaceae embedded within the total group of Araucariaceae. While this position is interesting with respect to similarities in cone morphology, we treat this result cautiously because the recovered relationships among the living conifer families (even when re-rooting with Pinaceae) differ markedly from the well-supported and commonly accepted conifer topology (e.g. Leslie *et al.*, 2018; Stull *et al.*, 2021). This alternative topological arrangement of the living families affects character reconstructions at internal nodes and therefore might impact the position of Cheirolepidiaceae. Additionally, the taxon sampling in Jin *et al.* (2023) was not broad enough to test possible affinities outside of the crown group of conifers.

In our study we build on these previous works by expanding the matrix of Andruchow-Colombo *et al.* (2023), adding seed cone, vegetative, pollen cone, pollen and pollination characters, as well as five additional Cheirolepidiaceae taxa from Argentina, Europe and the USA. The resulting matrix has a total of six species of Cheirolepidiaceae, which were selected for their relative completeness in terms of organ number or exceptional preservation. These species were analysed together with fossil and living representatives of all major conifer groups (i.e. all living families and voltzialeans) to test previously proposed relationships of Cheirolepidiaceae among conifers. It is important to note here that while we sampled multiple genera of all living conifer families, representing major clades within families and significant axes of morphological diversity, the taxon sampling of fossils belonging to living conifers remains limited, potentially impacting relationships. That said, our analyses recovered a few conflicting positions that reflect the most popular hypotheses on the affinities of this conifer family (Figs 8 and 9, Supplementary Data Appendixes S2C and S3A). However, there is one topology that is most common across all the analyses: the placement of Cheirolepidiaceae as the sister group of the conifer crown group. (Figs 8 and 9). This position was recovered as the single most parsimonious option in all 48 analyses of extended implied weights, as one of the most parsimonious options in 67 % of the 12 equal weight analyses, and in our FBD analysis.

Their position as sister to the conifer crown group suggests that the Cheirolepidiaceae represent a structural intermediate between voltzialean and modern conifers. Similarities between Cheirolepidiaceae and the voltzialean conifers have long been noted by palaeobotanists and this is the relationship most commonly proposed by researchers studying Cheirolepidiaceae fossils. The morphological similarities between these two conifer groups are mainly based on the general morphology of their seed cones. Permian and Triassic voltzialean conifers share with Cheirolepidiaceae the presence of ovuliferous shoots/scales with several lobes, usually in multiple layers (e.g. Florin, 1951; Clement-Westerhof, 1987; Kerp and Clement-Westerhof, 1991; Schweitzer, 1996). However, Cheirolepidiaceae seed cones are more compact than those of most voltzialeans, their ovuliferous complexes have fewer seeds (one or two vs three or many), and they have inverted seeds embedded in ovuliferous scale tissues (Clement-Westerhof, 1988; Escapa *et al.*, 2012). Focusing on these particular traits, the Cheirolepidiaceae seem closer to the modern conifer bauplan, without quite fitting within any particular lineage of extant conifers (Archangelsky, 1968; Jung, 1968). Instead, they make sense as an early modern lineage that, like some living conifers, still retains the traces of voltzialean morphology (i.e. lobed ovuliferous scales). Concordantly, the estimated Late Triassic to Early Jurassic diversification age of Cheirolepidiaceae (Fig. 9) is roughly coeval with the age of occurrence of the first reliable records of living conifer families (Stockey, 1975; Kunzmann, 2007; Escapa *et al.*, 2008; Rothwell *et al.*, 2012; Spencer *et al.*, 2015; Matsunaga *et al.*, 2021; Andruchow-Colombo *et al.*, 2023; see also Table 1). In this sense, our results support the Cheirolepidiaceae as part of Florin’s transformational series as structural intermediates between voltzian voltzialeans and living conifers (Florin, 1951; Clement-Westerhof and Konijnenburg-van Cittert, 1991; Rothwell *et al.*, 2011).

*Arkansia* seed cones, and those of Cheirolepidiaceae more generally, share many similarities with the cones of living conifer families, but some differences hint at ecological and evolutionary changes that have occurred in the evolution of living conifer lineages. In addition to exhibiting prominent ovuliferous scale lobes, the distal regions of *Arkansia* ovuliferous scales were likely fleshy rather than sclerified or coriaceous. Other taxa, such as *Alvinia bohemica*, have been similarly described as soft and only partially sclerified (Kvaček, 2000). While *Arkansia* lacks anatomical preservation of this part of the cone, we note that the distal lobes of *Pararaucaria patagonica* were aerenchymatous (Escapa *et al.*, 2012). It is likely, therefore, that Cheirolepidiaceae cones lacked the extensive lignification that is common among many living conifers. These differences may reflect the varied or changing ecological contexts of Mesozoic conifers and the increasing strength and complexity of biotic interactions, which likely shaped conifer reproductive evolution (Leslie *et al.*, 2012). Among modern conifers, robust and heavily sclerified cones provide protection against seed predators, whereas fleshy and brightly coloured tissues attract seed dispersers. The anatomy of Cheirolepidiaceae seed cones could potentially reflect low abundance of seed predators, adaptation for seed dispersal, or even a strategy for sealing the cone during seed maturation, if in fact cones were truly fleshy.

While the seed cones of the Cheirolepidiaceae appear to be part of a clear trend of simplification in the evolution of conifer seed cones, their pollen and leaf morphological diversity show different patterns. The pollen grains of Cheirolepidiaceae, referred to *Classopollis*, are highly conserved within the family while highly autapomorphic among conifers. These pollen grains are spherical, non-saccate and show a distal cryptopore, a proximal triangular depressed area, an equatorial band and a subequatorial furrow (Clement-Westerhof and Konijnenburg-van Cittert, 1991). The character combination shown by *Classopollis* is unseen in any other plant group, and it was associated with potential adaptations for insect pollination (Labandeira *et al.*, 2007), although other authors argued that the plants producing it would have been wind-pollinated due to the abundance of dispersed pollen and the pollen morphology (Clement-Westerhof and Konijnenburg-van Cittert, 1991). Leaves within the family are markedly more diverse than other organs and usually less systematically informative, encompassing scale-like morphologies that can be either appressed (*Brachyphyllum*, *Watsoniocladus*) or spreading (*Tomaxellia*, *Pagiophyllum*) and sheathing forms with extended decurrent leaf bases that can be arranged helically (*Pseudofrenelopsis*) or in whorls of two to three (*Frenelopsis*) (Watson, 1982). A similar pattern of high leaf variability also occurs in the living families Araucariaceae, Cupressaceae and Podocarpaceae (de Laubenfels, 1953). In these families, it has been proposed that the ancestral condition is that of scale leaves, while other morphologies appeared later in their evolution (Andruchow-Colombo *et al.*, 2018, 2023). This pattern of mosaic evolution in Cheirolepidiaceae seems consistent with observations of modern lineages of conifers, suggesting repeated patterns of morphological diversification across conifers.

### Origin of conifers and the rise and fall of the Cheirolepidiaceae: analysing the congruence of divergence time estimates with the macrofossil and palynological records

Our divergence time analyses under the FBD model provided node age estimates (Fig. 9; Supplementary Data Appendix S4D), which we will only consider here for deep nodes in the phylogeny due to limited fossil sampling for shallower nodes (i.e. limited fossil sampling within living families). We estimated the divergence of conifers from the Cordaitales to be 342.9 Ma (mean age), during the Middle Mississippian (Carboniferous). The divergence of the conifer crown group from the Cheirolepidiaceae was estimated as 257.0 Ma (late Lopingian [latest Permian]). The estimated mean age for the origin (i.e. initial diversification) of Cheirolepidiaceae was 202.7 Ma (late Rhaetian [Late Triassic]); and the estimated mean age for the origin of the crown group of conifers was 236.6 Ma (Carnian [Late Triassic]) (Supplementary Data Appendix S4D).

Our estimated age of origin of the conifer crown group (95 % HPD interval 268.67–206.51 Ma, late Permian to Late Triassic) is significantly younger than those of previous studies, which generally place it in the Carboniferous (i.e. 358.9–298.9 Ma; Leslie *et al.*, 2012, 2018; Stull *et al.*, 2021). These previous dated phylogenies included a much broader taxon sampling among living taxa but did not sample extinct taxa as terminals. These phylogenies are characterized by long internal branches leading to the crown groups of living conifer families and imply significant extinction among early crown group conifers. Our younger estimate may have been influenced by the relatively limited taxon sampling within the conifer crown group. However, it more likely reflects the fact that we have for the first time reconstructed a stratigraphically extensive conifer stem group that pushes the age of the conifer crown group closer to the present. Our analysis suggests an alternative scenario to that of previous node-dating analyses, in which there is a younger conifer crown group derived from a long stem group of conifers.

In this vein, a Triassic origin of the crown group of conifers is broadly in line with observations of the fossil record, notably (1) widespread occurrences of voltzialean conifers from the late Carboniferous to the Triassic, (2) reports of modern-looking taxa of contentious affinities in the Late Triassic (e.g. Delevoryas and Hope, 1973; Axsmith and Ash, 2006), and (3) the appearance of reliable representatives of modern conifer families in the Jurassic (Stockey, 1975; Kunzmann, 2007; Escapa *et al.*, 2008; Rothwell *et al.*, 2012; Spencer *et al.*, 2015; Matsunaga *et al.*, 2021; Andruchow-Colombo *et al.*, 2023). In the scenario supported by our dating analysis, the modern-looking but contentious taxa from the Late Triassic could represent the early diversification of the conifer crown group.

Our dating analysis places the origin of Cheirolepidiaceae between the Carnian (Late Triassic) and the Toarcian (Early Jurassic) (95 % HPD 231.94–178.13 Ma). While these estimates are based exclusively on macrofossils, they are congruent with patterns observed in the global pollen record (see Appendix I at the end of the text for detailed discussion of the global *Classopollis* record). Stratigraphically, palynomorphs morphologically hypothesized as early forms of *Classopollis* appear towards the end of the Middle Triassic in Europe (Alvin, 1982; Kürschner and Herngreen, 2010; Zhang *et al.*, 2020; Appendix I at the end of the text). The *Classopollis* group, which encompasses the synonymous palynomorphs *Circulina*, *Classopollis* and *Corollina* (also known as the *Circulina–Classopollis–Corollina* complex; Traverse, 2004), appears globally in the Upper Triassic, varying in abundance across localities. Above the Triassic–Jurassic boundary, *Classopollis* becomes a widespread and abundant palynomorph (Helby *et al.*, 1987; Volkheimer and Papú, 1993; Marzoli *et al.*, 2004; Cirilli, 2010; de Jersey and McKellar, 2013; Zhang *et al.*, 2020; Appendix I), coeval with the appearance of the first reliable macrofossil records of the family around the world (Srivastava, 1976; Vakhrameyev and Doludenko, 1977; Vakhrameev, 1981; Alvin, 1982; Table 1). As noted by Cirilli (2010), the appearance and dispersion of the *Classopollis* group constituted a global microfloral event that marked the end of Triassic provincialism. After the Cretaceous–Palaeogene (K–Pg) boundary, while no Cheirolepidiaceae macrofossils are recorded anywhere in the world, records of *Classopollis* have been reported in Argentina, China and the USA (Alvin, 1982; Barreda *et al.*, 2012; Wan *et al.*, 2014; Clyde *et al.*, 2021; Smith *et al.*, 2024). While many Palaeogene *Classopollis* records from the USA are likely reworked (Alvin, 1982), Danian records from Argentina (Barreda *et al.*, 2012; Clyde *et al.*, 2021 ), Turkmenistan (Vakhrameev, 1970; Alvin, 1982) and Texas (Smith *et al.*, 2024) seem to be true occurrences, indicating Cheirolepidiaceae persisted after the Cretaceous–Palaeogeneextinction in some regions.

Overall, our dating analysis, which included a wide range of representatives of extinct conifer lineages as terminals, provides age estimates for the origin of the crown group of conifers and the diversification of Cheirolepidiaceae that are congruent with patterns observed in the macrofossil and palynological records. These results help demonstrate the importance of including fossil taxa to populate long internal branches (Nixon, 1996; Mongiardino-Koch *et al.*, 2021), such as those within the conifer phylogeny, which is essential for reconstructing divergence times and exploring aspects of macroevolutionary history.

### Conclusions

We formally described *Arkansia axsmithii* sp. nov. based on seed cones and isolated ovuliferous complexes previously collected by Stanley (1988) and Axsmith and colleagues (Axsmith *et al*., 2004*a*, *b*; Axsmith and Ash, 2006). The ovuliferous complexes are cordiform to deltoid and distally lobed, with two lateral lobes and seven central lobes arranged in three rows; lobes were likely fleshy. Each ovuliferous complex bears a single mature seed, but the µCT scan of the holotype showed evidence of a putative aborted seed, suggesting that *A. axsmithii*, like other members of Cheirolepidiaceae, had potential to produce two seeds. The new species occurs in association with previously described wood, leafy shoots, pollen cones and pollen. We revised the associated organs and separated the vegetative remains, previously referred to the morphotaxon *Pseudofrenelopsis parceramosa*, into the new species *Pseudofrenelopsis axsmithii* based on differences in their branching pattern, leaf anatomy and epidermal morphology. We also documented leaf insect damage for the first time in this taxon. The organ association presented here constitutes the *Arkansia* total plant, which is the most complete cheirolepidiaceous taxon known to date.

Cheirolepidiaceae affinities have been long discussed in literature but rarely explored in a phylogenetic context. To tackle this question, we included the *Arkansia* total plant together with five other Cheirolepidiaceae species in phylogenetic analyses of all major conifer groups using different optimality criteria and varying search conditions. Our phylogenetic results most consistently recover the Cheirolepidiaceae as sister to the crown group of conifers, corroborating the general agreement among researchers that have studied the family. Some analyses resulted in topologies that recover Cheirolepidiaceae in different positions within the conifer crown group. While in the minority, these alternative positions reflect aspects of the long-lasting controversies regarding the affinities of Cheirolepidiaceae. *Pararaucaria patagonica* was occasionally recovered in our analyses outside of Cheirolepidiaceae, between the core Cheirolepidiaceae and the crown group of conifers. However, *P. patagonica* is based solely on seed cone remains and lacks the structures that provide most of the reconstructed synapomorphies of the family (i.e. pollen grains and leaf epidermis). Therefore, its occasional exclusion from the Cheirolepidiaceae clade might result from the lack of information on these organs. Overall, our phylogenetic analyses strongly support the view of Cheirolepidiaceae as a transitional lineage between voltzialean and living conifers. This lineage had an ovuliferous complex morphology with traits intermediate between some voltzialeans (the ‘voltzian Voltziales’) and extant taxa, leaves that were highly diverse and homoplastic (as in several living conifer families), and highly conserved and autapomorphic pollen grains.

Finally, we estimated divergence times of early lineages of conifers using the FBD model, which relies on direct incorporation of fossils as tips in the phylogeny rather than as priors on node ages. Our study therefore provides the first phylogenetic age estimates of these groups based on a tip-dating approach. Our estimated age of origin for the crown group of conifers ranges between the Late Permian and the Late Triassic and is significantly younger than molecular clock estimates in previous studies, which generally place it in the Carboniferous. Our estimates, however, are broadly in line with the conifer palaeobotanical record. Regarding Cheirolepidiaceae, our dating analysis estimates their origin between the Late Triassic and the Early Jurassic, and while these estimates are based on macrofossil data only, they are congruent with patterns of the pollen record across the world.

## Supplementary Material

mcag069_Supplementary_Data

## SUPPLEMENTARY DATA

**Appendix I**: Review of the oldest and youngest palynological records of Cheirolepidiaceae (after the Literature cited section).

**Online:**

Appendix S1: dataset (folder with files). Appendix S1A: character list and fossil calibrations (.docx file). Appendix S1B: morphological matrix in nexus format, combined matrix in TNT (continuous, discrete and DNA characters) and nexus (discrete and DNA characters) formats (folder with files). Appendix S2: parsimony analyses (folder with files). Appendix S2A: parsimony analysis – equal weights (EW) – tree summary – no constraints (folder with files). Appendix S2Ai: summary of equal weights (EW) trees – no constraints (.docx file). Appendix S2B: parsimony analysis – old conifer constraint (i.e. constraint of the conifer crown group with all fossils as floaters except for Voltziales and Cordaitales) – equal weights (EW) (folder with files). Appendix S2Bi: results – old conifer constraints analyses (.docx file; constraint of the conifer crown group with all fossils as floaters except for Voltziales and Cordaitales). Appendix S2Bii: synapomorphies per grouping – equal weights (EW) (.xlsx file). Appendix S2C: summary of equal weights (EW) – table results (.docx file). Summarization of results from Appendixes S2A and S2B. Appendix S2D: common synapomorphies – equal weights (EIW) (.docx file). Appendix S3: Bayesian analysis (folder with files). Appendix S3A: all compatibility consensus tree figure from the Bayesian Analysis (.pdf file). Appendix S4: FBD_analysis_appendix (folder). Appendix S4A: strict clock (folder with files). Appendix S4B: relaxed clock (folder with files). Appendix S4C: figure making (folder with files). Appendix S4D: posterior ages (.docx file). Appendix S5: *PlaceMyFossils* analysis. Raw inputs (morphological matrix and reference tree) and outputs are provided, a figure that summarizes the information retrieved by this analysis (fig. Appendix S5) is also provided. Appendix S6: comparisons of *Pseudofrenelopsis* species. Table S6: comparative table of species of *Pseudofrenelopsis* from Mendes *et al.* (2023) with *P. axsmithii* incorporated in the last row.

## Appendix Review of the oldest and youngest palynological records of Cheirolepidiaceae

*Europe.* Kürschner and Herngreen (2010) studied a succession of Triassic microfloras from central Europe and marked the Ladinian (the second of the two stages of the Middle Triassic ∼242–247 Ma) with the first appearance of the palynomorphs *Partitisporites* spp. and *Praecirculina granifer*, interpreted by these authors as early forms of *Classopollis*. In the Danish basin, the palynomorph *Classopollis* appears by the Norian to low Rhaetian (Upper Triassic; Cirilli, 2010). Peaks of abundance of *Classopollis* (= *Corollina*) are noted for the Rhaetian of Germany, Austria and the UK (Tanner *et al.*, 2004; Bonis and Kürschner, 2012).

*South America. Classopollis* appears in Argentinean sequences in the Norian (Upper Triassic; Volkheimer and Papú, 1993; Zavattieri *et al.*, 1994; Zavattieri and Batten, 1996; Zavattieri and Mego, 2008; Césari *et al.*, 2021) and becomes frequent in the Lower Jurassic (Volkheimer and Papú, 1993). Additionally, starting in the Lower Jurassic, numerous Cheirolepidiaceae macrofossil records were reported from Argentinean localities (Calder, 1953; Stockey, 1977; Falaschi *et al.*, 2011; Escapa *et al.*, 2012, 2013, 2022; Escapa and Leslie, 2017).

*Oceania.* Sedimentary successions from Australia and New Zealand show *Classopollis* (= *Corollina*) appearing in the Norian (Cirilli, 2010) or Rhaetian (Upper Triassic) and becoming abundant to dominant in the Lower Jurassic (Sinemurian to Toarcian stages, Helby *et al.*, 1987; de Jersey and McKellar, 2013).

*North America*. *Classopollis* (= *Corollina*) was reported for the upper Carnian of the Taylorsville basin in the USA (Cornet, 1993) and Rhaetian of the Newark Basin of Pennsylvania in the USA, being a minor component in the Upper Triassic but becoming dominant in the Jurassic (Fowell *et al.*, 1994; Olsen *et al.*, 2002*a*, *b*; Tanner *et al.*, 2004). In the Blomidon Formation, eastern Canada, *Classopollis* becomes a dominant palynomorph at the uppermost Triassic (Tanner *et al.*, 2004). The Upper Triassic of North America also holds the oldest macrofossil record referred to Cheirolepidiaceae, *Pseudohirmerella delawarensis* (Axsmith *et al*, 2004*b*). Its affinities with the family are contentious as it does not show typical Cheirolepidiaceae seed cone morphology and preserves neither pollen nor epidermal characters. However, as was also discussed for Triassic putative members of living conifer families, *Pseudohirmerella* could constitute an early-diverging taxon of the Cheirolepidiaceae lineage.

*Africa*. *Classopollis* was reported at least in the Carnian, Norian and Rhaetian (Upper Triassic) of multiple localities in Northern Africa, as well as in the Lower Jurassic (Lachkar *et al.*, 2000; Marzoli *et al.*, 2004).

*Asia*. There are reports of *Classopollis* in the upper Carnian of Siberia (Upper Triassic; Ilyina and Egorov, 2008) and the Norian–Rhaetian of south and eastern China (Li *et al.*, 2018, 2023). *Classopollis* and *Discisporites* (which has been proposed as related to *Classopollis*; Alvin, 1982) were reported in the Upper Triassic of north-west China, where *Classopollis* is dominant in the Lower Jurassic (Zhang *et al.*, 2020).

*Classopollis* is abundant in Jurassic and Cretaceous sediments across the world, coeval with the macrofossil record of Cheirolepidiaceae (Srivastava, 1976; Vakhrameyev and Doludenko, 1977; Vakhrameev, 1981; Alvin, 1982; Table 1). After the Cretaceous–Palaeogene boundary, which marks the last of the five mass extinctions of Earth’s history, no macrofossil records of Cheirolepidiaceae are recorded anywhere in the world. However, studies show the presence of *Classopollis* right after the K–Pg boundary in different regions of the world; these include Argentina, China, Turkmenistan and the USA (Vakhrameev, 1970; Alvin, 1982; Barreda *et al.*, 2012; Wan *et al.*, 2014; Clyde *et al.*, 2021). Due to their abundance and preservation, Palaeogene *Classopollis* records from the USA have been largely interpreted as reworked pollen from older sediments (Alvin, 1982; Berry, 2022), except for a more recent discovery in Texas (Smith *et al.*, 2024). However, *Classopollis* records from Argentina and Turkmenistan (Vakhrameev, 1970; Alvin, 1982) are accepted as a true signal of the presence of Cheirolepidiaceae after the Late Cretaceous mass extinction event (Quattrocchio 2006; Quattrochio *et al.*, 2011; Barreda *et al.*, 2012; Clyde *et al.*, 2021). A Danian (lowest Palaeogene) *Classopollis* spike (i.e. low abundance to virtual absence in Maastrichtian [uppermost Cretaceous] and a high percentage in the Danian, ranging from ∼23 to 80 %) was reported in two localities in Argentina (Quattrocchio, 2006; Quattrocchio *et al.*, 2011; Barreda *et al.*, 2012). Additionally, a third locality in Argentina showed consistently high abundance of *Classopollis* though the K–Pg boundary (Clyde *et al.*, 2021). *Classopollis* then disappears from the pollen record, marking the final night of this mostly Mesozoic conifer family.
