# Supplementary material for: Revisiting the enigmatic Cheirolepidiaceae: origins, phylogenetic relationships, and a new whole-plant concept
Source: Ann Bot. 2026 Mar 30;137(6):1876–901. doi: 10.1093/aob/mcag069 (PMC13275020; doi:10.1093/aob/mcag069)
Supplement: mcag069_Supplementary_Data [file mcag069_supplementary_data.zip › Appendix_S6_PseudofrenelopsisTable_fromMendesEtAl2023.pdf]

Table S6. Comparative table of species of *Pseudofrenelopsis* from Mendes et al. (2023) with the *P. axsmithii* incorporated in the last row.

Species	Maximum length of leaf tip	Shape of leaf apex	Leaf margin	Nodal areas	Ridges on internode	Adaxial surface	Abaxial surface	Stomata arrangement	Subsidiary cells	Number of subsidiary cells on abaxial (internode) cuticle	Stratigraphy	Selected references
P. felixii = *P. varians*	1.5 mm	Blunt	With trichomes	Not expanded	Absent	With trichomes	With trichomes	Variable irregular to forming ill-defined rows	Florin ring with papillae	(4)5–8(9)	Aptian-Albian	A.G. Nathorst in Felix and Lenk, 1893; Watson, 1977
P. capillata	2 mm	Acutely blunt	With trichomes	Not expanded	Absent	With trichomes	With trichomes	Forming well-defined rows	With papillae	5–6	Aptian	Sucerquia et al., 2015
P. dalatzensis	2 mm	Acutely blunt	With papillae	Not expanded	Present	With trichomes	With papillae	Forming rows	Florin ring with papillae	(6)7–8(10)	Albian-Aptian	Chow and Tsao, 1977; Zhou, 1995
P. gansuensis	1.5 mm	Blunt	Without trichomes or papillae	Not expanded	Absent	With trichomes	Without trichomes or papillae	Forming rows	Without papillae	5–7(8–9)	Albian-Aptian	Deng et al., 2005
P. glabra	1.5 mm	Blunt	Without trichomes or papillae	Not expanded	Absent	Without papillae	Without trichomes or papillae	Forming rows	Without papillae	6–8	Albian	Saiki, 1999
P. guixiensis	2 mm	Acute	With trichomes	Not expanded	Present	With trichomes	Without trichomes or papillae	Forming rows	With papillae	4–6	Lower Cretaceous	Sun et al., 2011
P. heishanensis	2 mm	Blunt	Without trichomes or papillae	Not expanded	Absent	Without trichomes or papillae	Without trichomes or papillae	Forming rows	With papillae	5–6(7)	pre-Albian	Zhou, 1995
P. intermedia	Less than 1 mm	Broadly acute - Blunt	Without trichomes or papillae	Not expanded	Absent	Without trichomes or papillae	Without trichomes or papillae	Forming ill-defined rows	Without papillae	6–11	Lower Cretaceous	Chow and Tsao, 1977
P. liupanshanensis	1 mm	Acute	Without trichomes or papillae	Not expanded	Absent	With papillae	With papillae	Forming well-defined rows	With papillae	(4)5–6	Lower Cretaceous	Du et al., 2014
P. nathorstiana	1 mm	Attenuate	With trichomes	Expanded	Absent	With trichomes	With trichomes	Forming short ill-defined rows	Florin ring with papillae	(4)5–6(7)	Albian	Srinivasan, 1995
P. papillosa	1.5 mm	Bluntly acute	Without trichomes or papillae	Not expanded	Present	With trichomes	Without trichomes or papillae	Forming well-defined rows	Florin ring with papillae	3–6	pre-Aptian	Chow and Tsao, 1977; Zhou, 1995
P. parceramosa	2 mm	Acutely blunt	With trichomes	Not expanded	Absent	With trichomes	Without trichomes or papillae	Forming well-defined rows	Florin ring with papillae	(4)5–6(7)	(Berriasian)-Aptian-(Cenomanian)	Watson, 1977
P. salesii	Very small	Acutely blunt	Unknown	Not expanded	Present	Unknown	With papillae	Forming well-defined rows	Without papillae	4–6	Albian	Batista et al., 2018
P. tholistoma	1–2 mm	Acute	Unknown	Not expanded	Present	Unknown	With or without papillae	Forming rows	With papillae	4–5	Late Early/Early Late Cretaceous	Chow and Tsao, 1977; Cao, 1989
P. zlatkoi	2 mm	Acute	With trichomes	Expanded	Present	With trichomes	With or without papillae	Forming well-defined rows	Florin ring with papillae	(3)4–6(7)	Late Aptian to early Albian	Kvaček and Mendes, 2023
P. dinisii	2.5 mm	Attenuate	With trichomes	Little expanded	Absent	With trichomes	With or without papillae	Forming well-defined rows	Florin ring with papillae	4–6	Hauterivian	Mendes et al. 2023
P. axsmithii	**2.6 mm**	**acute to slightly obtuse**	**With trichomes**	**Little expanded**	**?**	**With trichomes**	**With papillae**	**Forming well-defined rows**	**Florin ring with papillae**	**(4)5–6**	**Aptian/Albian**	**Present paper**

Notes. Cells marked in green indicate a shared character state with *Pseudofrenelopsis axsmithii*.
