# Supplementary material for: Revisiting the enigmatic Cheirolepidiaceae: origins, phylogenetic relationships, and a new whole-plant concept
Source: Ann Bot. 2026 Mar 30;137(6):1876–901. doi: 10.1093/aob/mcag069 (PMC13275020; doi:10.1093/aob/mcag069)
Supplement: mcag069_Supplementary_Data [file mcag069_supplementary_data.zip › AppendixS1-Dataset.zip!/AppendixS1-Dataset/AppendixS1A-CharacterListAndFossilCalibrations.docx]

**Taxon sampling	p. 1–3**

**Character list	p. 3–25**

**Fossil calibrations	p. 25**

**Literature cited	p. 26**

**Taxon sampling**

(54/55)

**Extant taxa**  (35)

Pinaceae  (11)

*Abies lasiocarpa*

*Cathaya argyrophylla*

*Cedrus deodara*

*Keteleeria davidiana*

*Larix decidua*

*Nothotsuga longibracteata*

*Picea abies*

*Pinus contorta*

*Pseudotsuga menziesii*

*Pseudolarix amabilis*

*Tsuga canadensis*

Araucariaceae  (3)

*Agathis australis*

*Araucaria heterophylla*

*Wollemia nobilis*

Podocarpaceae  (11)

*Acmopyle pancheri*

*Dacrydium cupressinum*

*Phyllocladus aspleniifolius*

*Podocarpus archboldii*

*Prumnopitys taxifolia*

*Saxegothaea conspicua*

*Microcachrys tetragona*

*Pherosphaera hookeriana*

*Retrophyllum rospigliosii*

*Lepidothamnus laxifolius*

*Lagarostrobos franklinii*

Cupressaceae  (7)

*Athrotaxis selaginoides*

*Callitris muelleri*

*Cryptomeria japonica*

*Cunninghamia lanceolata*

*Cupressus funebris*

*Sequoia sempervirens*

*Taiwania cryptomerioides*

Sciadopityaceae (1)

*Sciadopitys verticillata*

Taxaceae (1)

*Taxus wallichiana*

Ginkgoaceae (1)

*Ginkgo biloba*

**Fossil taxa**  (19/20) – newly added in bold.

*Schizolepidopsis ediae* (Early Cretaceous, Pinaceae; Matsunaga et al., 2021)

*Pararaucaria patagonica* (Middle Jurassic, Cheirolepidiaceae; Escapa et al. 2012)

*Hanskerpia hamiltonensis* (Pennsylvanian, Voltziales; Rothwell et al. 2005)

*Cordaixylon dumusum* (Pennsylvanian, Cordaitales; Rothwell 1993)

*Emporia lockardii* (Pennsylvanian, Voltziales; Mapes and Rothwell 1984, 2003; Rothwell et al., 2005; Hernández-Castillo et al., 2009)

*Telemachus aequatus* (Middle Triassic, Voltziales; Yao et al. 1997; Bomfleur et al., 2013)

*Eathiestrobus mackenziei* (Late Jurassic, Pinaceae; Rothwell et al. 2012)

*Schizolepidopsis canicularis* (Early Cretaceous, Pinaceae; Leslie et al. 2013)

*Schizolepidopsis daohugouensis* (Middle–Late Jurassic, Pinaceae; Zhang et al. 2011)

*Voltzia hexagona* (Permian, Voltziales; Schweitzer, 1996)

*Rissikia media* (Late Triassic, Voltziales; Townrow, 1967a)

*Nothodacrium warrenii* (Early–Middle Jurassic, Voltziales; Townrow, 1967b)

*Mataia podocarpoides* (Late Jurassic, Coniferophyta; Townrow, 1967a)

***Tomaxellia biforme*** (Early Cretaceous, Argentinean Patagonia, Cheirolepidiaceae; Archanglesky, 1966, 1968; Archangelsky & Gamerro, 1967)

***Himeriella muensteri*** (Early Jurassic, Germany, Cheirolepidiaceae; Clement-Westerhof & Van Konijnenburg-Van Cittert, 1991; Barbacka et al., 2007; Doweld, 2020)

***Kachaikestrobus acuminatus*** (Early Cretaceous, Argentinean Patagonia, Cheirolepidiaceae; Del Fueyo et al., 2008a)

***Alvinia bohemica* plant** (Late Cretaceous, Czech Republic, Cheirolepidiaceae; Kvacek, 2000)

**New total plant from Arkansas** (Early Cretaceous, USA, Cheirolepidiaceae; Axsmith, 2004, 2006; this work)

***Araucaria mirabilis*** (Middle Jurassic, Argentina, Araucariaceae; Calder, 1953; Stockey, 1975)

*Schizolepidopsis longipetiolus* (Lower Cretaceous, Pinaceae, Xu et al. 2013) – only included in	the Bayesian analysis.

*Schizolepidopsis daohugouensis* (Middle–Late Jurassic, Pinaceae; Zhang et al. 2011)

**Character list**

**Seed cones**

**0. Number of ovuliferous complexes in the seed cone.** Meristic (treated as ‘continuous character’ for TNT searches). Only used in parsimony analyses.

Character from Andruchow-Colombo et al. 2023a

This character is intended to represent the same morphological variations as characters 1 (logarithmic transformation) and 66 (discrete character). These three characters were never active at the same time for an analysis, instead they were activated or inactivated alternatively to test the influence of different character definitions on the resulting topology.

This character is standardized to 1 or 5 (command *nstates stand* in TNT), meaning that the maximum possible length of a character transformation (i.e., between 1 and 250, which are the two extremes of the distribution in our sample) is either one step or five (see justification in materials and methods of the publication for these two chosen values).

**1. Logarithm of the number of ovuliferous complexes in the seed cone.** Continuous character. Only used in parsimony analyses.

Character from Andruchow-Colombo et al. 2023a

The number of ovuliferous complexes (OC) in conifers varies between 1 (e.g., *Taxus*) and 250 (e.g., *Araucaria*). Small variations in the OC number are clearly more informative for taxa that have few OCs per cone than in those that have many.

In this sense, the highly diverse genus *Podocarpus* (Podocarpaceae) has more than 150 species that invariably present 1–2 OC per cone; genera as *Pherosphaera* (Podocarpaceae) vary between 3–8 OC per cone; while the genus *Araucaria* (Araucariaceae) has seed cones with 50–250 OC per cone. These examples show that variation of a few units in seed cones with fewer OCs per cone can be more informative, as they can define differences between genera. Whereas in taxa with larger cones (in terms of number of OC), the same difference in a few cone elements tend to be less informative (or not at all) as it is more likely to lie within the range of natural variation of genera and even species.

Therefore, we transformed this variable with a base 10 logarithm, which results in a higher numeric difference among low numbers of OC than in higher number of OCs (se figure below). With this transformation, the range 1–2 becomes 0–0.3, whereas the range 101–102 becomes 2.0043–2.0086. In the first case there is a difference of 0.3 between states, whereas in the second case the difference is of 0.0043, therefore a change of one unit in the lower region of the total range weights more than a change in its middle or higher regions.

The character is then standardized to 1 or 5 (command *nstates stand* in TNT), as it was explained for character 0.

See original (i.e., OC per cone) and logarithmic (i.e., Log_10_ (OC per cone)) values for this character in section D of Appendix I.

**2. Ovules borne in determinate fertile aggregations. 0 = absent; 1 = present.**

Based on Rothwell et al. 2005 character 37, Matsunaga et al 2021 character 1, Andruchow-Colombo et al., 2023a character 2.

We score species as "present" when ovules are produced in cones/strobili, regardless of whether cones are reduced to one or a few fertile units (i.e., as in many Podocarpaceae). Taxa with ovules not borne in cones or strobili, or taxa with fertile regions rather than terminal cones, are scored as "absent".

**3. Ovuliferous scale clearly distinguishable from bract: 0 = absent; 1 = present.**

Character from Andruchow-Colombo et al. 2023a (based on Matsunaga et al 2021 character 2, rescored).

We follow the Florin model of ovulate cone evolution, in which the cones of modern conifers are interpreted as compound. Those cones in which there have been extreme reduction or fusion of the ovuliferous scale, resulting in its apparent absence, are scored as “absent”; examples of this are *Agathis* (lacking any external evidence of a scale), *Cupressus* (the ovuliferous complexes are accepted to be bracts only), *Phyllocladus* and *Pherosphaera* (lacking epimatium), and *Taxus*. Whenever a scale and a bract are distinguishable as independent structures this character is scored as “present” (e.g., *Pinus*, *Podocarpus*, *Araucaria*).

**4. Ultimate ovule-bearing unit.** **0=stem; 1=sporophyll, 2 = axillary structure (can be a scale or a modified ovuliferous shoot), 3=bract**

Character from Andruchow-Colombo et al. 2023a (modified and re-scored from Rothwell et al. 2005 character 34, and Matsunaga et al 2021 character 3, state 2).

In modern-like conifers the ovuliferous complex is present, being the accepted interpretation that they resulted from the reduction and fusion of elements of the ancestral axillary ovuliferous shoot during the evolutionary history of conifers. Whenever the individual elements of the shoot are no longer differentiated as independent structures, we consider that the ultimate bearing unit is an axillary structure formed by variably fused and/or reduced constituent parts (i.e., sterile leaves, fertile leaves, and the bearing shoot). Examples of this are Pararaucaria, Rissikia, and most extant conifers (e.g., *Picea*, *Acmopyle, Araucaria*)

This character then refers to the ultimate structure on which the ovules are borne. “Stem” refers to ovules that terminate axes (e.g., *Taxus*) or that are borne in the axil of bracts (e.g., *Pherosphaera*). Sporophyll refers to ovules that are borne on the sporophylls (fertile leaves) of an ovuliferous shoot (e.g., *Hanskerpia*). Axillary structure refers to dwarf shoots with an evident degree of fusion (e.g., *Voltzia*), ovuliferous complexes where the bract and the scale can be separately distinguished (e.g., *Pinus*), including those cases with modified forms of the scale such as the epimatium (e.g., *Podocarpus*). Bract refers to ovules that are born on the adaxial surface of a bract or of an ovuliferous complex with no differentiation between bract and scale (e.g., *Cupressus*, *Agathis*).

**5. Fusion of ovuliferous shoot. 0 = absent; 1 = present (any degree).**

Character from Andruchow-Colombo et al. 2023a

Fusion of the ovuliferous shoot refers to fusion of elements (foliar and/or axial), irrespective of reduction in the size or number of these elements.

Absent: Individual elements are apparently unfused, even if borne on a reduced axis (see explanation of char. 60, *Axillary shoot reduction*) and appearing to arise from a single point. Taxa should be scored as unfused if, for example, the margins of the individual leaves are visible to their base (e.g., *Voltzia*, *Cordaixylon*).

Present: Individual elements of the axillary shoot are fused for some to their total length. These individual elements can be distinguishable as lobes, but margins of elements are not visible to their base, or all elements are fused into a single structure lacking lobes (i.e., unlobed ovuliferous scale; e.g., *Rissikia*, *Telemachus*, *Pararaucaria*, *Pinus*).

**6. Ovuliferous shoot/scale symmetry.** **0 = helical symmetry; 1 = bilateral symmetry.**

Character from Andruchow-Colombo et al. 2023a (Based on Rothwell et al. 2005 character 43, Gernandt et al. 2018 character 116, Matsunaga et al 2021 character 6. Character modified and rescored, now Podocarpaceae with non-flattened ovuliferous complexes that show a bilateral symmetry are scored with “bilateral symmetry”).

**7. Number of sterile leaves in ovuliferous shoot.** **0 = zero; 1 = 1–5; 2 = 6–20; 3 = >20.**

From Rothwell et al. 2005 character 50, Matsunaga et al 2021 character 7, Andruchow-Colombo et al. 2023a character 7

This refers to the presence and abundance of sterile leaves within the ovuliferous dwarf shoots of fossil conifers. N/A if ovuliferous scales are present or secondarily absent.

**8. Vegetative and fertile leaves intergrade in the dwarf shoot. 0 = absent; 1 = present.**

From Rothwell et al. 2005 character 51, Matsunaga et al 2021 character 8, Andruchow-Colombo et al. 2023a character 8.

Refers to whether there is morphological intergradation between vegetative and fertile scales in an ovuliferous dwarf shoot. N/A if ovuliferous scales are present; applicable only when character 7 is scorable and its state different than zero.

For taxa scored as “present”, fertile and sterile leaves of the dwarf shoot intergrade or are indistinguishable from one another in size and/or shape, except for the presence of a seed. For taxa scored as absent, sterile and fertile leaves are distinct in size and/or shape.

**9. Sterile leaves on adaxial side of shoot. 0 = absent; 1 = present.**

From Rothwell et al. 2005 character 52, Matsunaga et al 2021 character 9, Andruchow-Colombo et al. 2023a character 9.

Describes the distribution of sterile leaves on an ovuliferous dwarf shoot. N/A if ovuliferous scales are present or secondarily absent.

**10. Sterile leaves on abaxial side of shoot.** **0 = absent; 1 = present.**

From Rothwell et al. 2005 character 53, Matsunaga et al 2021 character 10, Andruchow-Colombo et al. 2023a character 10.

Describes the distribution of sterile leaves on an ovuliferous dwarf shoot. N/A if ovuliferous scales are present or secondarily absent.

**11. Fertile leaves aggregated at ovuliferous dwarf shoot apex.** **0 = absent; 1 = present.**

From Rothwell et al. 2005 character 54, Matsunaga et al 2021 character 11, Andruchow-Colombo et al. 2023a character 11.

Describes the distribution of fertile scales within ovuliferous dwarf shoots. N/A if ovuliferous scales are present.

**12. Ovules produced adaxially on dwarf shoot.**  **0 = absent; 1 = present**

Modified from Rothwell et al. 2005 character 44, Matsunaga et al 2021 character 12, Andruchow-Colombo et al. 2023a character 12.

Describes the distribution of fertile leaf within ovuliferous dwarf shoots. N/A if ovuliferous scales are present or secondarily absent.

**13. Ovules produced laterally on dwarf shoot. 0 = absent, 1 = present.**

Modified from Rothwell et al. 2005 character 44, Matsunaga et al 2021 character 13, Andruchow-Colombo et al. 2023a character 13.

Describes the distribution of fertile leaf within ovuliferous dwarf shoots. N/A if ovuliferous scales are present or secondarily absent.

**14. Ovule position on fertile leaf of a dwarf shoot. 0 = marginal/apical; 1 = near tip; 2 = midregion; 3 = basal.**

From Rothwell et al. 2005 character 48, Matsunaga et al 2021 character 14, Andruchow-Colombo et al. 2023a character 14.

Describes the position of the ovule on the fertile leaf of an ovuliferous dwarf shoot. N/A if ovuliferous scales are present or secondarily absent.

**15. Ovules per fertile leaf of a dwarf shoot.** **0 = one; 1 = more than one.**

Modified from Rothwell et al. 2005 character 36, Matsunaga et al 2021 character 15, Andruchow-Colombo et al. 2023a character 15.

Indicates the number of ovules borne on each fertile leaf of an ovuliferous dwarf shoot. N/A if ovuliferous scales are present or secondarily absent.

**16. Ovules with apical projections.** **0 = absent; 1 = present.**

From Rothwell et al. 2005 character 55, Matsunaga et al 2021 character 16, Andruchow-Colombo et al. 2023a character 16.

The ovules of some early conifers can have several types of appendages. This character refers to whether the ovules have two lobes or projections at the apex.

**17. Ovule with chalazal extensions.** **0 = absent; 1 = present.**

Modified from Rothwell et al. 2005 character 55, Matsunaga et al 2021 character 17, Andruchow-Colombo et al. 2023a character 17.

The ovules of some early conifers can have several types of appendages. This character refers to whether the ovules have lobes, horns, or other extensions at the chalazal end.

**18. Seed number on each ovuliferous shoot or ovuliferous complex.** **0 = one; 1 = two; 2 = three to ten; 3 = >10**

Modified from Matsunaga et al 2021 character 18, Andruchow-Colombo et al. 2023a character 18.

This describes the number of seeds produced by each ovuliferous dwarf shoot or ovuliferous complex.

**19. Ovule with nucellar beak.** **0 = absent; 1 = present.**

From Matsunaga et al 2021 character 19, , Andruchow-Colombo et al. 2023a character 19.

In some gymnosperms, the nucellus forms a beak that protrudes up towards or into the micropyle.

**20. Seed number per ovuliferous complex variable in cone.** **0 = absent; 1 = present.**

From Matsunaga et al 2021 character 20, Andruchow-Colombo et al. 2023a character 20.

Describes whether seed number is variable depending on position of the scale within the cone, as is seen in most Cupressaceae.

**21. Ovuliferous complex shape.** **0 = foliate; 1 = pseudopeltate/cuneate; 2 = peltate; 3 = valvate.**

From Andruchow-Colombo et al. 2023a character 21 (based on Farjon 2005 character 41, Matsunaga et al 2021 character 22, with modifications introduced by Andruchow-Colombo et al 2022 character 16).

Describes the shape of the ovuliferous complex. Foliate complexes are dorsiventrally flattened, roughly leaf-like in shape. Pseudopeltate ovuliferous complexes are more tridimensional than the foliate and tend to have a proportionally longer and thinner stalk, with seeds positioned in a distal widened portion of the complex that culminates with a generally lignified cap. Peltate complexes are broadest distally and taper to a narrow base, or umbrella-like with a central stalk-like structure, the peltae become fused together in the post‐pollination cone, protecting the developing seeds. Valvate ovuliferous complexes are shell‐like, lignified, in some genera presenting interlocking cells located on their edges.

**22. Spine/flap-like protrusion from bract. 0 = absent; 1 = present.**

From Matsunaga et al 2021 character 24, Andruchow-Colombo et al. 2023a character 22.

This character refers to a spine-like or laminar protrusion of the bract, such as an umbo, on the external surface of mature or post-pollination stage ovuliferous cones.

**23. Number of ovuliferous scale lobes. 0 = one; 1 = two; 2 = three to five; 3 = more than five.** **Ordered character (in parsimony).**

Modified from Matsunaga et al 2021 character 26 and rescored, Andruchow-Colombo et al. 2023a character 23.

**24. Cone structure.** **0 = lax; 1 = compact.**

From Andruchow-Colombo et al., 2019b character 36; Matsunaga et al 2021 character 28, Andruchow-Colombo et al. 2023a character 24.

This describes the cone structure with respect to the density of cone elements and overall cone habit. Lax cones have long internodes or widely spaced cone elements (e.g., bract-scale complexes). Cones comprised of fertile areas along a shoot rather than being terminal and determine are scored as ‘lax’. Cones with unfused axillary ovuliferous shoots are also scored as ‘lax’. Compact cones are determinate and have tightly imbricated or appressed bract-scale complexes.

**25. Aril. 0 = absent; 1 = present.**

From Andruchow-Colombo et al. 2023a character 26

Refers to whether a fleshy aril surrounding the seed and originating apparently from the integument (Tomlinson et al., 1989a).

**26. Receptacle. 0 = absent; 1 = present.**

From Andruchow-Colombo et al. 2019 character 33, Matsunaga et al 2021 character 31, Andruchow-Colombo et al. 2023a character 27.

Describes whether fertile and sterile bracts are modified into a fleshy receptacle.

**27. Receptacle size. 0 = larger than seed + epimatium; 1 = smaller than seed + epimatium.**

From Andruchow-Colombo et al. 2019 character 35, Matsunaga et al 2021 character 32, Andruchow-Colombo et al. 2023a character 28.

Size of receptacle relative to the epimatium and seed.

**28. Receptacle surface.** **0 = smooth; 1 = warty.**

From Andruchow-Colombo et al. 2019 character 35, Matsunaga et al 2021 character 33, Andruchow-Colombo et al. 2023a character 29.

**29. Mature seed orientation. 0 = erect; 1 = oblique; 2 = inverted.**

From Andruchow-Colombo et al. 2019 character 53, Andruchow-Colombo et al. 2023a character 30.

Final orientation of the seed. Erect refers to the micropyle pointing completely away from the cone axis (e.g., *Pherosphaera*). Oblique refers to seeds that are nearly but not completely erect (e.g., *Acmopyle*). Inverted seeds have the micropyles pointed proximally (i.e., towards the cone axis; e.g., *Araucaria*).

**30. Ovuliferous scale seed wing. 0 = absent; 1 = present.**

From Matsunaga et al 2021 character 35, Andruchow-Colombo et al. 2023a character 31.

Refers to whether mature seeds have a wing that is derived from the adaxial epidermis of the ovuliferous scale (e.g., *Pinus*).

**31. Integumentary seed wing.** **0 = absent; 1 = present.**

From Matsunaga et al 2021 character 36, Andruchow-Colombo et al. 2023a character 32.

Refers to whether mature seeds have a wing that is derived from the integument (e.g., *Agathis*).

**32. Seed wing number per seed. 0 = one; 1 = two; 2 = three.**

Based on Farjon 2005 character 49, Matsunaga et al 2021 character 37, Andruchow-Colombo et al. 2023a character 33.

Number of distinct/recognizable wings on each seed.

**33. Seed wing position. 0 = distal; 1 = lateral; 2 = proximal; 3 = circumferential.**

Based on Farjon 2005 character 49, Matsunaga et al 2021 character 38, Andruchow-Colombo et al. 2023a character 34.

Position of the seed wing relative to the cone base of the ovuliferous complex.

**34. Seed wing symmetry.** **0 = symmetric; 1 = asymmetric.**

Based on Farjon 2005 character 51, Matsunaga et al 2021 character 39, Andruchow-Colombo et al. 2023a character 35.

Applies only to species with multiple wings per seed or circumferential wings.

**35. Fusion of bract and ovuliferous structure.** **0 = completely fused** (i.e., fused for 100% of scale length - boundary between bract and ovuliferous scale cannot be distinguished); **1 = mostly fused** (fused for >50% of scale/shoot length); **2 = partially fused** (fused for <50% of scale/shoot length); **3 = free** (no fusion observed, structures are completely free).

Based on Gernandt et al. 2018 character 111, Andruchow-Colombo et al. 2019 character 43, and Rothwell et al. 2005 character 39; Matsunaga et al 2021 character 40, Andruchow-Colombo et al. 2023a character 36.

Degree of fusion between the bract and any axillary ovuliferous structure, including ovuliferous scales and dwarf shoots.

**36. Bract-ovuliferous scale/shoot relative length.** **0 = bract < scale** (bract is shorter than ovuliferous scale or shoot); **1 = equal** (bract and ovuliferous scale/shoot are approximately of equal length); **2 = bract > scale** (bract is longer than ovuliferous scale or shoot).

Based on Gernandt et al. 2018 character 113, and Andruchow-Colombo et al. 2019 character 44; Matsunaga et al 2021 character 41, Andruchow-Colombo et al. 2023a character 37.

Length of the bract relative to the subtended ovuliferous scale or dwarf shoot.

**37. Dominant structure protecting/bearing the seed.** **0 = ovuliferous scale; 1 = bract; 2 = ovuliferous shoot.**

From Matsunaga et al 2021 character 42, with change of character name, Andruchow-Colombo et al. 2023a character 38.

This describes whether the bract versus the ovuliferous scale is the dominant structure in mature seed cones. By dominant we mean that it is the primary structure that bears or protects the seeds in post-pollination stage cones. While character 37 deals with the relative length of the structures composing the ovuliferous complex, this character deals with the overall dimensions, particularly the width. In this sense, for example the extant *Pseudotsuga* and the extinct *Schizolepidopsis ediae* (Matsunaga et al., 2021) have bracts that are significantly longer than the scales but that are narrow structures that do not provide significant protection to the seed. Whereas the scale is shorter and wider, covering the seeds completely in both these dimensions.

**38. Bract apex.** **0 = simple; 1 = bifid; 2 = trifid.**

Based on Rothwell et al 2005 character 40 and Gernandt et al 2018 character 115, Matsunaga et al 2021 character 43, Andruchow-Colombo et al. 2023a character 39.

Morphology of the bract apex, in some conifers the bract is distally divided in a forked or lobed way.

In Podocarpaceae, the bract is sometimes modified and forms part of the fleshy receptacle. We score this character for such taxa based on the morphology of the bract at earlier stages of ontogeny.

**39. Interseminal ridge.** **0 = absent; 1 = present.**

Based on Gernandt et al. 2018 character 120, and Smith et al. 2016 character 42; Matsunaga et al 2021 character 44, Andruchow-Colombo et al. 2023a character 40.

Refers to whether a prominent ridge is formed on the ovuliferous scale between adjacent seeds (when the ovuliferous complex bears more than one seed), as viewed in transverse section through the ovuliferous scale. N/A if there is one seed per ovuliferous complex.

**40. Scale or seed trichomes/papillae.** **0 = absent; 1 = present.**

Based on Gernandt et al. 2018 character 125, expanded to include trichomes on seeds; Matsunaga et al 2021 character 45, Andruchow-Colombo et al. 2023a character 41.

This character refers to whether there are prominent trichomes or papillae on the ovuliferous complex or seed.

**41. Ovuliferous scale base. 0 = stalked; 1 = broad.**

Based on Gernandt et al 2018 character 119, Smith et al 2016 character 38, Matsunaga et al 2021 character 46, Andruchow-Colombo et al. 2023a character 42.

Only applicable for taxa with foliate ovuliferous complexes (see character 21, Ovuliferous complex shape). Describes the morphology of ovuliferous complex where it diverges from the cone axis. Stalked bases are narrow relative to the rest of the scale (equivalent to “pedicellate” from Gernandt et al. 2018 and Smith et al. 2016), whereas broad bases lack a stalk-like component.

**42. Seed embedded in ovuliferous scale tissue.** **0 = absent; 1 = present.**

Based on character from Escapa and Catalano 2013 character 26 and Andruchow-Colombo et al. 2019 character 46, Matsunaga et al 2021 character 48, Andruchow-Colombo et al. 2023a character 43.

This character describes seeds that are embedded in ovuliferous scale tissue, for instance as a pocket of ovuliferous scale tissue or an epimatium surrounding the seed.

**43. Ovules forming rows.** **0 = absent; 1=present.**

From Matsunaga et al 2021 character 50, Andruchow-Colombo et al. 2023a character 44.

This refers to whether the ovules are arranged in rows and applies only when there are more than two ovules associated with each scale or scale lobule.

**44. Number of ovule rows.** **0 = one; 1 = two; 2 = three.**

Matsunaga et al 2021 character 51, Andruchow-Colombo et al. 2023a character 45.

Scored as N/A for taxa lacking ovule rows (character 44).

**45. Cortical resin canals in cone axis. 0 = absent; 1 = present.**

Based on Gernandt et al 2018, character 137, Matsunaga et al 2021 character 53, Andruchow-Colombo et al. 2023a character 47.

**46. Cortical resin canal diameter.** **0 = uniform; 1 = dilated near branching points.**

Based on character 8 from Smith et al. 2016 and character 138 from Gernandt et al 2018, Matsunaga et al 2021 character 54, Andruchow-Colombo et al. 2023a character 48.

When seen in longitudinal sections through the cone axis, resin canals in some taxa become markedly dilated near their divergence into bract-scale complexes (state=1).

**47. Pith resin canals in cone axis.** **0 = absent; 1 = present.**

From Gernandt et al 2018 character 129, Smith et al 2016 character 2, Matsunaga et al 2021 character 55, Andruchow-Colombo et al. 2023a character 49.

**48. Resin canals in ovuliferous scale.** **0 = absent; 1 = present.**

From Gernandt et al 2018 character 150, Smith et al 2016 character 25, Matsunaga et al 2021 character 56, Andruchow-Colombo et al. 2023a character 50.

**49. Resin canals in bract.** **0 = absent; 1 = present.**

From Gernandt et al 2018 character 146, Smith et al 2016 character 20, Matsunaga et al 2021 character 57, Andruchow-Colombo et al. 2023a character 51.

**50. Secondary xylem of cone axis.** **0 = continuous cylinder; 1 = separate strands.**

Based on character 132 of Gernandt et al 2018, Matsunaga et al 2021 character 58, Andruchow-Colombo et al. 2023a character 52.

This describes the arrangement of secondary xylem in the cone axis. In some taxa it forms a continuous ring around the pith, whereas in others the secondary xylem consists of discrete strands.

**51. Scale and bract trace divergence.** **0 = separate; 1 = united.**

Based on character 135 of Gernandt et al. 2018. See also Miller 1976, Matsunaga et al 2021 character 59, Andruchow-Colombo et al. 2023a character 53.

This character refers to whether the vascular traces to the bract and ovuliferous scale depart from the cone axis as a single strand, which then divides to supply the bract and scale (state 1, united), or if the vascular supply to each structure diverges from the cone axis separately (state 0, separate).

**52. Ovuliferous complex arrangement.** **0 = helical; 1 = opposite; 2 = whorled.**

Based partially on Farjon 2005 character 26, Matsunaga et al 2021 character 60, Andruchow-Colombo et al. 2023a character 54.

Arrangement (phyllotaxis) of ovuliferous complexes in the cone.

**53. Scale trace shape. 0 = abaxially concave; 1 = cylindrical.**

Modified from Gernandt et al 2008 character 142, Matsunaga et al 2021 character 61, Andruchow-Colombo et al. 2023a character 55.

Shape of the vascular trace at the base of the ovuliferous scale, ovuliferous shoot, or ovuliferous complex when bract and scale are fused at that level, as seen in transverse section.

**54. Ovuliferous complex resin canal origin.** **0 = one origin; 1 = two origins.**

From Gernandt et al 2008 character 141, see also Miller 1976, Matsunaga et al 2021 character 63, Andruchow-Colombo et al. 2023a character 56.

Refers to whether the resin canals that the ovuliferous complex originate as a single canal trace that branches, or from multiple traces.

**55. Resin canals at scale base: abaxial to trace. 0 = absent; 1 = present.**

From Gernandt et al 2008 character 152, Matsunaga et al 2021 character 64, Andruchow-Colombo et al. 2023a character 57.

Position of resin canals relative to vascular trace when viewing the scale base in transverse section.

**56. Resin canals at scale base: adaxial to trace.** **0 = absent; 1 = present.**

From Gernandt et al 2008 character 153, Matsunaga et al 2021 character 65, Andruchow-Colombo et al. 2023a character 58.

Position of resin canals relative to vascular trace when viewing the scale base in transverse section.

**57. Bract vascularization. 0 = one bundle; 1 = more than one bundle.**

From Gernandt et al 2008 character 143, Matsunaga et al 2021 character 66.

Number of vascular bundles within the bract, Andruchow-Colombo et al. 2023a character 59.

**58. Bract resin canal number.** **0 = one; 1 = two; 2 = more than two.**

From Gernandt et al 2008 character 147, Matsunaga et al 2021 character 67, Andruchow-Colombo et al. 2023a character 60.

**59. Bract sclerenchyma.** **0 = absent; 1 = present.**

From Gernandt et al 2008 character 148, Matsunaga et al 2021 character 68, Andruchow-Colombo et al. 2023a character 61.

Refers to whether bracts are sclerenchymatous.

**60. Axillary shoot reduction.  0 = absent; 1 = present**

From Andruchow-Colombo et al., 2023a character 62.

Reduction can encompass variation/change in size/length of the axillary shoot, the number of elements, and compactness/density of elements (internode length). This character refers specifically to reduction in the axial (stem) component of the shoot in terms of elongation and internode length.

Absent: axis elongated, usually with both fertile and sterile scales, each of them emerging from different points along the axis (i.e., clear internodes present).

Present: axis not elongated, vegetative scales present or not, all leaves apparently emerge from the same point (i.e., internodes not distinguishable).

**61. Degree of fusion of scale elements. 0 = Barely fused** (less than 10% of total length); **1 = Fused up to mid-region** (11–50%); **2 = Fused 60-85% of total length**; **3 = Nearly completely fused** (bearly lobed); **4 = Completely fused**.

From Andruchow-Colombo et al., 2023a character 63.

**Ordered character (in parsimony).** Only applicable if character 4 (fusion of ovuliferous scales) is scored as “present (any degree)” (1).

The total length is considered as starting at the divergence point of the scale/ovuliferous complex from cone axis and ending at the distalmost point of the scale.

**62. Ovuliferous complex morphology (when it has bilateral symmetry). 0 = mostly flattened; 1 = mostly three-dimensional.**

From Andruchow-Colombo et al., 2023a character 64.

Refers to whether the ovuliferous complex is mostly flattened as in extant Pinaceae or mostly tridimensional as in *Podocarpus* or *Cupressus*. Only applicable if character 5 (Ovuliferous shoot/complex symmetry) is scored as having bilateral symmetry.

**63. Seed cone/ fertile zone symmetry. 0; radial; 1 = asymmetric; 2 = bilateral.**

From Andruchow-Colombo et al., 2023a character 65. Refers to the symmetry of the whole cone/fertile zone.

Most conifers have radially symmetrical cones, some Podocarpaceae as *Podocarpus* have asymmetric cones, and some Cupressaceae sensu stricto have bilaterally symmetrical cones.

**64. Number of ovuliferous complexes in the seed cone. 0 = 1–2; 1 = 3–10; 2 = 11–20; 3 = 21–50; 4 = 50–250.**

From Andruchow-Colombo et al., 2023a character 66. **Ordered in some analyses. Only active if character zero and one** (0. Number of ovuliferous complex in the seed cone – Meristic character; 1. Logarithm of the number of ovuliferous complexes in the seed cone – Continuous character) **are inactive.**

**65. OC scale forms part of the diaspore: 0 = absent; 1 = present.**

From Andruchow-Colombo et al., 2023a character 67 (based on Andruchow-Colombo et al 2019 character 56; Gernandt et al, 2018 character 108, Matsunaga et al 2021 character 47).

*Wollemia*, *Agathis* and most Cupressaceae disperse their seeds only, therefore characters 67, 68, and 69 are scored as absent for them. *Abies* and *Prumnpitys* disperse seed and scale tissues (scale represented by seed wing in *Abies* and epimatium in *Prumnopitys*), for them character 67 is scored as present but 68 and 69 are scored as absent. Most *Araucaria* species disperse ovuliferous complexes (seed-bract-scale) so they are scored as present for characters 67 and 68, and as absent for character 69. *Microcachrys* and *Podocarpus* dispersion unit are their whole cones, therefore they are scored as present for characters 67, 68, and 69.

**66. OC bract forms part of the diaspore: 0 = absent; 1 = present.**

From Andruchow-Colombo et al., 2023a character 68 (based on Andruchow-Colombo et al 2019 character 56; Gernandt et al, 2018 character 108, Matsunaga et al 2021 character 47).

**67. Other bracts form part of the diaspore: 0 = absent; 1 = present.**

From Andruchow-Colombo et al., 2023a character 69 (based on Andruchow-Colombo et al 2019 character 56; Gernandt et al, 2018 character 108, Matsunaga et al 2021 character 47).

**Leaves**

**68. Leaves in short shoots:** 0 = Absent; 1 = Present.

**69. Leaves in long shoots:** 0 = Absent; 1 = Present.

**70. Leaf reorientation.** 0 = Absent; 1 = Present

Describes when leaves are reoriented on the stem such that photosynthetic surfaces face the same direction. Corresponds to “expanded photosynthetic units” of Podocarpaceae

**71. Leaf arrangement.** 0 = Helical; 1 = Whorled; 2 = Opposite to Subopposite; 3 = Decussate; 4 = alternate.

Modified from Andruchow-Colombo et al., 2023b character 0

**72. Mature leaf morphology.** 0 = Reduced leaves; 1 = Scale-like (i.e., non-expanded); 2 = Broad; 3 = Needle-like

Modified from Andruchow-Colombo et al., 2023b character 1; Refers to the primary photosynthetic leaves. For taxa with multiple leaf types, if there is a dominant one (i.e., majority of leaves are of one type), the taxon will be scored for the dominant leaf type. For example in *Acmopyle*, there are two types of scale-like leaves and one type of broad leaves, but the broad leaves are the most common, and so it is scored as “broad”.

**73. Leaf types.** 0 = Bilaterally flattened; 1 = Round to quadrangular in cross-section; 2 = Bifacially flattened.

From Andruchow-Colombo et al., 2023b character 3 – **Ordered character (in parsimony).**

**Bilaterally flattened leaves** **(0)** occur in several Podocarpaceae with expanded leaves (e.g., *Acmopyle*, Sahni, 1920 textfig. 2). These leaves, have two sides that do not correspond to abaxial or adaxial; instead, each leaf side (left and right) is composed of an abaxial and an adaxial region. **Leaves round to quadrangular in cross section (1)** describe both the needle-shaped leaves of Pinaceae (round in cross section) and leaves from Cupressaceae, Araucariaceae, and Podocarpaceae that are rhomboidal in cross section (i.e., they have adaxial and abaxial keels, e.g., *Dacrycarpus imbricatus*; Wilf, 2012, figs. 6, 7 that show dimorphic leaves of the bilateral type, located more terminal on the branches, and of the rhomboidal type, more proximal). **Bifacially flattened leaves (2)** are the most common leaf type in plants, with an abaxial and adaxial side clearly identifiable.

**74. Abaxial keel.** 0 = Absent; 1 = Present.

From Andruchow-Colombo et al., 2023b character 4. Only scored for bifacially flattened leaves, as it is redundant for leaves that are bilaterally flattened or rhomboidal in cross section(see character 73).

**75. Mature leaf margin.** 0 = Entire; 1 = Serrulate; 2 = With marginal frill.

From Andruchow-Colombo et al., 2023b character 5.

Serrulate (1) refers to irregular denticles emerging from leaf margins (e.g., Del Fueyo et al., 2008a, fig. g); in leaves with marginal frills (2) have a series of cells of similar length that emerge more or less perpendicular to the leaf margin forming an even fringe along part of all the leaf margin (e.g., Andruchow-Colombo et al., 2019a, fig. C, F). Entire margins (0) don’t have lateral teeth or frills.

**76. Leaf base.** 0 = Without constriction; 1 = Constricted/(Pseudo)petiolate.

From Andruchow-Colombo et al., 2023b character 7. Most conifer leaves don’t have a constriction towards the leaf base (e.g., *Pinus*, *Wollemia*, *Microcachris*, *Cupressus*). Examples of pseudopetiolate leaves are those of *Agathis*, and *Podocarpus*.

**77. Leaf apex.** 0 = simple; 1 = forked.

New character. Forked/divided leaves are extremely rare among conifers, they have been reported for *Emporia lockardii* (Mapes and Rothwell 1984, 2003; Rothwell et al., 2005; Hernández-Castillo et al., 2009). Leaves were scored as forked/divided also in the outgroup species, *Ginkgo biloba*.

**78. Phylloclades I.** 0 = Absent; 1 = Present.

From Andruchow-Colombo et al., 2023b character 9. Phylloclades are complex branch systems fused into a simple or compound photosynthetic structure, among living conifers the genus *Phyllocladus* is the only one that has them (Keng, 1973, 1977; Andruchow-Colombo et al., 2019b). Cladodes of *Sciadopitys* are not considered as phylloclades as their composition is different (Dörken and Stützel, 2011)

**79. Leaf sheath.** 0 = Absent; 1 = Present.

New character.

We define leaf sheath as the situation in which most of the leaf is leaf base that is appressed to the stem or completely fused to it, and completely surrounding the stem. The sheath covers all the distal internode. May be composed of more than one leaf from the same whorl in taxa with opposite or whorled phyllotaxis (e.g., *Tetraclinis*, *Callitris*, *Frenelopsis*), or from a single leaf per node (e.g., *Pseudofrenelopsis*). The leaf may have a free tip.

**80. Stomata on the adaxial side of the leaf.** 0 = Absent; 1 = Present.

From Andruchow-Colombo et al., 2023b character 10. Refers on whether stomata are present or absent in the adaxial leaf side.

**81. Stomata on the abaxial side of the leaf.** 0 = Absent; 1 = Present.

From Andruchow-Colombo et al., 2023b character 11. Refers on whether stomata are present or absent in the abaxial leaf side.

**82. Stomata orientation.** 0 = Mostly parallel; 1 = Mostly oblique; 2 = Mostly perpendicular; 3 = Random.

From Andruchow-Colombo et al., 2023b character 13. Refers to the orientation of the stomatal aperture with respect to the longitudinal axis of the leaf.

**83. Stomatal organization I.** 0 = Rows; 1 = Scattered.

From Andruchow-Colombo et al., 2023b character 14. Stomata in conifers are generally organized in rows (e.g., *Araucaria*, Stockey & Ko, 1986, fig. 15); scattered stomata are more rare (e.g., Parasitaxus, Wells & Hill, 1989, fig.50).

**84. Stomatal organization II.** 0 = Lateral bands; 1 = Throughout the width of the leaf; 2 = Central band.

From Andruchow-Colombo et al., 2023b character 15. In many taxa stomata are distributed in two bands at each side of the central vein (e.g., Andruchow-Colombo et al., 2019a, fig. 2E); some taxa have a continuous stomatal distribution throughout the leaf width (Fig. 4B, C, H).

**85. Polar subsidiary cells.** 0 = Absent; 1 = Present.

From Andruchow-Colombo et al., 2023b character 16. The position of polar subsidiary cells is sometimes occupied by ordinary epidermal cells (e.g., Stockey, 1990, fig. 30), when this happens this character is scored as polar subsidiary cells absent (0).

**86. Florin ring.** 0 = Absent; 1 = Reduced; 2 = Well developed. **Ordered character (in parsimony).**

From Andruchow-Colombo et al., 2023b character 19.

**87. Papillae in ordinary epidermal cells.** 0 = Absent; 1 = Present.

Modified from Andruchow-Colombo et al., 2023b character 25. See Fig 5A for examples of epidermal papillae.

**88. Papillae in Subsidiary cells.** 0 = Absent; 1 = Present.

New character. See Fig. 5B, C for examples of subsidiary cell papillae

**89. Leaf vasculature.** 0 = Multiple vascular bundles; 1 = Two vascular bundles; 2 = Single vascular bundle.

From Andruchow-Colombo et al., 2023b character 26. Refers to the number of veins in a leaf.

**90. Phylloclades II.** 0 = Simple; 1 = Compound

From Andruchow-Colombo et al., 2023b character 60. Phylloclades (structure explained in character 78) can be simple (lamina non divided) or compound (lamina divided), similarly to leaves in angiosperms and ferns.

**91. Accessory transfusion tissue.** 0 = Absent; 1 = Present.

See Buchholz &Gray (1948), Brodribb & Holbrook (2005), Andruchow-Colombo et al. (2023b) for details on morphology and distribution of accessory transfusion tissue (ATT). Within conifers, ATT was only described for some genera in Podocarpaceae.

**92. Transfusion tissue.** 0 = Absent; 1 = Present.

See Buchholz &Gray (1948), Hu & Yao (1981) for details on morphology and taxonomic distribution of transfusion tissue.

**93. Transfussion Tissue distribution.** 0 = Lateral bands to vascular bundle; 1 = All arround vascular bundle; 2 = Adaxial to the vascular bundle (might extend laterally); 3 = Abaxial to the vascular bundle (might extend laterally).

Modified from Gernandt et al., 2018 character 87 following TT types defined by Hu & Yao, 1981.

0 = Lateral bands to vascular bundle was catalogued as *Cupressus*-type and *Taxus*-type by Hu & Yao, 1981; 1 = All arround vascular bundle was catalogued as *Pinus*-type by Hu & Yao, 1981; 2 = Adaxial to the vascular bundle (might extend laterally) was catalogued as *Araucaria­*-type by Hu & Yao, 1981; 3 = Abaxial to the vascular bundle (might extend laterally) was catalogued as *Pseudotsuga*-type by Hu & Yao, 1981.

**94. Spongy parenchyma relative density.** 0 = Dense; 1 = Lax.

Dense spongy parenchyma show few or no lacunae between cells (e.g., *Abies*, *Araucaria*), lax spongy parenchyma has lacunae between the component cells (e.g., *Lepidothamnus*)

**95. Mesophyll secretory canals.** 0 = Absent; 1 = Present.

From Gernandt et al. 2018 character 81.

**96. Hypodermis in leaves.** 0 = Absent; 1 = Present.

**97. Endoderm Casparian strips.** 0 = Absent; 1 = Present.

From Gernandt et al. 2018 character 85.

**98. Resin canal(s).** 0 = Absent; 1 = Present.

**99. Number of resin canals (if present).** 0 = less than the number of veins; 1 = equal to the number of veins; 2 = more than the number of veins.

**100. Resin canal position.** 0 = External; 1 = Medial; 2 = Interrupting endodermis.

Modified from Gernandt et al., 2018 character 82.

External resin canals refer to their position in contact with the hypodermis/epidermis; while medial refer to those embedded in the spongy mesophyll.

**Pollen cones**

**101. Pollen cone shape.** 0 = Globose; 1 = Elliptical; 2 = Cylindrical.

**102. Microsporophyll with shank (stalk) and distal lamina.** 0 = Absent; 1 = Present.

From Rothwell et al. 2005 character 24.

**103. Sac attachment on microsporophyll.** 0 = Terminal; 1 = Abaxial; 2 = Adaxial.

From Gernandt et al. 2018 character 90, Rothwell et al., 2005 characters 28 and 29.

**104. Microsporophyll sac morphology.** 0 = Globose; 1 = Elongate.

**105. Microsporophyll sac number.** 0 = One; 1 = Two; 2 = Three; 3 = More than four.

**106. Sac dehiscence.** 0 = Longitudinal; 1 = Transversal, 3 = Oblique.

From Gernandt et al. 2018 character 92.

**Pollen**

**107. Pollen sacci.** 0 = Absent; 1 = Present.

**108. Pollen sacci number.** 0 = One; 1 = Two; 2 = Three; 3 = Multiple.

Multiple sacci occur in *Lasiocarpus* according to Singh (1978, p. 65–66).

**109. Subecuatorial canal.** 0 = Absent; 1 = Present.

See Watson (1988, fig. 9.26).

**110. Thickened ecuatorial band:** 0 = Absent; 1 = Present.

See Watson (1988, fig. 9.26) and Del Fueyo et al. (2008b).

**111. Distal cryptopore.** 0 = Absent; 1 = Present.

See Watson (1988, fig. 9.26).

**112. Papilla in center of germinal zone.** 0 = Absent; 1 = Present.

See Ho & Sziklai (1973).

**Pollination**

**113. Pollination drop.** 0 = Absent (nonhydrogamy); 1 = Present (hydrogamy).

Following descriptions by Tomlinson, 1994.

For hydrogamous taxa, the pollen enters via reabsorbed pollination drop and germinates inside the pollen chamber; for nonhydrogamous taxa the pollen might germinate inside or outside the pollen chamber (see characters 115 *Pollen trap in mycropilar zone* and 116 *Extended siphonogamy*).

**114. Orientation of pollination drop:** 0 = Randomly oriented; 1 = Inverted; 2 = Erect.

Following descriptions by Tomlinson, 1994.

Pollination drops can be inverted by extension and inversion of the micropyle (e.g., *Acmopyle*, Mill et al., 2001; *Lepidothamnus*, Andruchow-Colombo et al., 2024), by inversion the ovule (e.g., *Dacrycarpus*, Tomlinson, 1994) or the cone.

**115. Pollen trap in mycropilar zone.** 0 = Abstent; 1 = Present.

In (1) the pollen is captured by the micropyle and germinates inside the pollen chamber. There are two main types of pollen traps (integument modifications) at the micropylar zone referred in literature:

One is a papillate extension that catch the pollen and later degenerate, forming a depression in the surface that engulfs the pollen grains, as seen in *Larix* and *Pseudotsuga* (Powell & Tosh, 1991; Tomlinson, 1994), other is the flange or funnel-like micropyle as seen in *Abies* (Doyle & Kane, 1943).

**116. Extended siphonogamy.** 0 = Abstent; 1 = Present.

Extended siphonogamy refers to the germination of the pollen outside of the pollen chamber reaching for the micropyle as seen in *Saxegothaea* (Podocarpaceae), *Tsuga* (Pinaceae), and Araucariaceae (Tison, 1908; Doyle & Kane, 1943; Tomlinson, 1994).

This character can only be scored for non-hydrogamous taxa (see character 113 *Pollination drop*)

**Fossil callibrations**

| **Taxon** | **Locality** | **Age range (myr)** | **Citation** |
| --- | --- | --- | --- |
| *Schizolepidopsis ediae* | Tevshiin Govi coal mine, Mongolia; and Zhahanaoer locality, Inner Mongolia, China | 145.0–100.5 | Ichinnorov 2003, 2005; Xu et al. 2013; Matsunaga et al. 2021 |
| *Pararaucaria patagonica* | Cerro Cuadrado, Cerro Alto and Cerro Madre e Hija, La Matilde Formation, Santa Cruz, Argentina | 172–162 | Escapa et al. 2012 |
| *Hanskerpia hamiltonensis* | Hartford Limestone, Topeka Limestone Formation, Shawnee Group, Kansas, U.S.A. | 307.1–298.8 | Rothwell et al. 2005 |
| *Cordaixylon dumusum* | Duquesne Coal, Apalachian Basin, Ohio, U.S.A. | 307.1–298.8 | Rothwell 1993 |
| *Emporia lockardii* | Hartford Limestone, Topeka Limestone Formation, Shawnee Group, Kansas, U.S.A. | 307.1–298.8 | Mapes & Rothwell 1984; Hernandez-Castillo et al. 2009 |
| *Telemachus aequatus* | Fremouw Peak, Fremouw Peak, Central Transantarctic Mountains, Antarctica | 247.2–242 | Yao et al. 1997 |
| *Eathiestrobus mackenziei* | Kimmeridge Clay Formation, Black Isle, Scotland | 154.0–149.9 | Rothwell et al. 2012 |
| *Schizolepidopsis canicularis* | Tevshiin Govi coal mine, Mongolia | 125.0–100.5 | Leslie et al. 2013 |
| *Schizolepidopsis longipetiolus* | Huolinhe Formation, Inner Mongolia, China | 139.8–129.4 | Xu et al. 2013 |
| *Schizolepidopsis daohugouensis* | Daohugou, Tiaojishan Formation, Ningcheng, Inner Mongolia, China | 174.2–145.0 | Zhang et al. 2011 |
| *Voltzia hexagona* | Zechstein conglomerate, Wetterau Basin, Germany | 284.1–259.3 | Schweitzer 1996 |
| *Rissikia media* | Ipswich and Burnera Waterfall, Molteno Formation, South Africa | 237–227 | Townrow 1967a; Anderson & Anderson 2003 |
| *Nothodacrium warrenii* | Kirkpatrick Basalt Fromation, Ferrar Group, Carapace Nunatak, Antarctica | 190.9–165.8 | Townrow, 1967b; Ribecai, 2007 |
| *Mataia podocarpoides* | Clent Hills Gr., New Zealand and Australia | 163.6–145.0 | Townrow 1967a; Kamp 2001 |
| *Tomaxellia biforme* | Estancia Bajo Grande, Anfiteatro de Ticó Formation, Baqueró Group, Santa Cruz, Argentina | 118.3–118.1 | Archangelsky 1966; Perez Loinaze et al. 2013; Passalia et al. 2016 |
| *Himeriella muensteri* | Bayreuth, Gümbelsche Sandstein and Sandgrube Lautner, Germany | 201.5–173.1 | Clement-Westerhof & Van Konijnenburg-Van Cittert, 1991 |
| *Kachaikestrobus acuminatus* | Estancia Kachaike, Kachaike Formation, Santa Cruz, Argentina | 113.0–106.8 | Del Fueyo et al. 2008a; Perez Loinaze et al. 2016 |
| *Alvinia bohemica* plant | Podlešín and Lipenec, Peruc-Korycany Formation, Czech Republic | 100.5–93.9 | Kvaček 2000 |
| **New total plant from Arkansas** | James Hardie Gypsum Quarry, Holly Creek Formation, Arkansas, U.S.A. | 119.0–106.8 | Axsmith et al. 2004; Axsmith 2006 |
| *Araucaria mirabilis* | Cerro Cuadrado, Cerro Alto and Cerro Madre e Hija, La Matilde Formation, Santa Cruz, Argentina | 172–162 | Stockey 1975; Zamuner & Falaschi 2005; Falaschi et al. 2009; Escapa et al. 2012 |

**Literature cited**

**Anderson JM and A**. **2003**. *Heyday of the Gymnosperms: systematics and biodiversity of the Late Triassic Molteno fructifications*.

**Andruchow-Colombo A, Escapa IH, Carpenter RJ, *et al.*** **2019a**. Oldest record of the scale-leaved clade of Podocarpaceae, early Paleocene of Patagonia, Argentina. *Alcheringa: An Australasian Journal of Palaeontology* **43**: 127–145.

**Andruchow-Colombo A, Wilf P, Escapa IH**. **2019b**. A South American fossil relative of Phyllocladus: Huncocladus laubenfelsii gen. et sp. nov. (Podocarpaceae), from the early Eocene of Laguna del Hunco, Patagonia, Argentina. *Australian Systematic Botany*.

**Andruchow-Colombo A, Escapa IH, Aagesen L, Matsunaga KKS**. **2023a**. In search of lost time: tracing the fossil diversity of Podocarpaceae through the ages. *Botanical Journal of the Linnean Society* **203**: 315–336.

**Andruchow-Colombo A, Rossetto-Harris G, Brodribb TJ, Gandolfo MA, Wilf P**. **2023b**. A new fossil Acmopyle with accessory transfusion tissue and potential reproductive buds: Direct evidence for ever-wet rainforests in Eocene Patagonia. *American Journal of Botany* **110**: e16221.

**Archangelsky S, Gamerro JC**. **1967**. Pollen grains found in coniferous cones from the lower cretaceous of patagonia (Argentina). *Review of Palaeobotany and Palynology* **5**: 179–182.

**Archangelsky S**. **1966**. New Gymnosperms from the Ticó Flora, Santa Cruz Province, Argentina. *Bulletin of the British Museum (Natural History)* **13**: 259–295.

**Archangelsky S**. **1968**. On the genus *Tomaxellia* (Coniferae) from the Lower Cretaceous of Patagonia (Argentina) and its male and female cones. *Journal of the Linnean Society of London, Botany* **61**: 153–165.

**Axsmith BJ, Krings M, Waselkov K**. **2004**. Conifer pollen cones from the Cretaceous of Arkansas: implications for diversity and reproduction in the Cheirolepidiaceae. *Journal of Paleontology* **78**: 402–409.

**Axsmith BJ**. **2006**. The vegetative structure of a Lower Cretaceous conifer from Arkansas: further implications for morphospecies concepts in the Cheirolepidiaceae. *Cretaceous Research* **27**: 309–317.

**Barbacka M, Ziaja J, Wcisło-Luraniec E**. **2007**. *Hirmeriella muensteri* (Schenk) Jung from Odrowąż (Poland), with female and male cones, and in situ Classopollis pollen grains. *Acta Palaeobotanica* **47**: 339–357.

**Bomfleur B, Decombeix A-L, Escapa IH, Schwendemann AB, Axsmith B**. **2013**. Whole-Plant Concept and Environment Reconstruction of a *Telemachus* Conifer (Voltziales) from the Triassic of Antarctica. *International Journal of Plant Sciences* **174**: 425–444.

**Brodribb TJ, Holbrook NM**. **2005**. Water Stress Deforms Tracheids Peripheral to the Leaf Vein of a Tropical Conifer. *Plant Physiology* **137**: 1139–1146.

**Buchholz JT, Gray NE**. **1948**. A Taxonomic Revision of Podocarpus: I. the Sections of the Genus and Their Subdivisons With Special Reference to Leaf Anatomy. *Journal of the Arnold Arboretum* **29**: 49–63.

**Calder MG**. **1953**. A coniferous forest in Patagonia. *Bulletin of the British Museum (Natural History) Geology* **2**: 97–138.

**Clement-Westerhof JA, an Konijnenburg-vanCittert JHA**. **1991**. Hirmeriella muensteri: New data on the fertile organs leading to a revised concept of the Cheirolepidiaeae. *Review of Palaeobotany and Palynology* **68**: 147–179.

**Del Fueyo GM, Archangelsky S, Llorens M, Cúneo R**. **2008a**. Coniferous Ovulate Cones from the Lower Cretaceous of Santa Cruz Province, Argentina. *International Journal of Plant Sciences* **169**: 799–813.

**Del Fueyo GM, Caccavari M, Dome EA. 2008b.** Morphology and Structure of the Pollen Cone and Pollen Grain of the *Araucaria* Species from Argentina. *BIOCELL* **32**: 49–60. <https://doi.org/10.32604/biocell.2008.32.049>.

**Dörken VM, Stützel T**. **2011**. Morphology and anatomy of anomalous cladodes in Sciadopitys verticillata Siebold & Zucc. (Sciadopityaceae). *Trees* **25**: 199–213.

**Doweld AB**. **2020**. The controversial nomenclature of the fossil plant names *Cheirolepis* , *Cheirolepidium* and *Hirmeriella* (Cheirolepidaceae/Cheirolepidiaceae/Hirmeriellaceae). *TAXON* **69**: 1092–1098.

**Doyle J, Kane A.** 1943. Pollinations in *Tsuga pattoniana* and in species of *Abies* and *Picea*. *Scientific Proceedings of the Royal Dublin Society* **23**: 57–70.

**Escapa IH, Rothwell GW, Stockey RA, Cúneo NR**. **2012**. Seed cone anatomy of Cheirolepidiaceae (Coniferales): Reinterpreting *Pararaucaria patagonica* Wieland. *American Journal of Botany* **99**: 1058–1068.

**Falaschi P, Zamuner AB, Foix N**. **2009**. Una nueva Equisetaceae fértil de la Formación La Matilde, Jurásico Medio, Argentina. *Ameghiniana* **46**: 263–272.

**Hernandez-Castillo GR, Stockey RA, Rothwell GW, Mapes G**. **2009**. Reconstructing *Emporia lockardii* (Voltziales: Emporiaceae) and Initial Thoughts on Paleozoic Conifer Ecology. *International Journal of Plant Sciences* **170**: 1056–1074.

**Ho RH, Sziklai O. 1973.** Fine Structure of the Pollen Surface of Some Taxodiaceae and Cupressaceae Species. *Review of Palaeobotany and Palynology* **15**: 17–26. <https://doi.org/10.1016/0034-6667(73)90013-4>.

**Ichinnorov N. 2003.** Palynocomplex of the Lower Cretaceous sediments of eastern Mongolia. *Mongolian Geoscientist* **22**:12–16.

**Ichinnorov N. 2005.** Pollen and spore assemblages and their stratigraphic significance. *Mongolian Geoscientist* **24**:160–162.

**Kamp PJJ**. **2001**. Possible Jurassic age for part of Rakaia Terrane: Implications for tectonic development of the Torlesse accretionary prism. *New Zealand Journal of Geology and Geophysics* **44**: 185–203.

**Keng H**. **1973**. On the Family Phyllocladaceae. *Taiwania* **18**: 142–145.

**Keng H**. **1977**. Phyllocladus and Its Bearing on the Systematics of Conifers In: Kubitzki K, ed. *Flowering Plants*. Vienna: Springer Vienna, 235–251.

**Kvaček J**. **2000**. Frenelopsis alata and its microsporangiate and ovuliferous reproductive structures from the Cenomanian of Bohemia (Czech Republic, Central Europe). *Review of Palaeobotany and Palynology* **112**: 51–78.

**Leslie AB, Glasspool I, Herendeen PS, *et al.*** **2013**. Pinaceae-like reproductive morphology in *Schizolepidopsis canicularis* sp. nov. from the Early Cretaceous (Aptian-Albian) of Mongolia. *American Journal of Botany* **100**: 2426–2436.

**Mapes G, Rothwell GW**. **1984**. *Lebachia* from late Palaeozoic limestones of Kansas. *Palaeontology* **27**: 69–94.

**Mapes G, Rothwell GW**. **2003**. Validation of the names Emporiaceae, Emporia, and Emporia lockardii. *TAXON* **52**: 327–328.

**Passalia MG**. **2016**. Revised stratigraphic framework of the Cretaceous in the Bajo Grande area (Argentinean Patagonia) inferred from new U-Pb ages and palynology. *Cretaceous Research*: 15.

**Perez Loinaze VS, Barreda VD, Archangelsky S, Archangelsky A**. **2016**. Cretaceous angiosperm pollen from the Kachaike Formation, south-western Santa Cruz Province, Argentina. *Historical Biology* **28**: 941–951.

**Perez Loinaze VS, Vera EI, Passalia MG, *et al.*** **2013**. High-precision U–Pb zircon age from the Anfiteatro de Ticó Formation: Implications for the timing of the early angiosperm diversification in Patagonia. *Journal of South American Earth Sciences* **48**: 97–105.

**Ribecai C**. **2007**. Early Jurassic miospores from Ferrar Group of Carapace Nunatak, South Victoria Land, Antarctica. *Review of Palaeobotany and Palynology* **144**: 3–12.

**Matsunaga KKS, Herendeen PS, Herrera F, Ichinnorov N, Crane PR, Shi G**. **2021**. Ovulate Cones of *Schizolepidopsis ediae* sp. nov. Provide Insights into the Evolution of Pinaceae. *International Journal of Plant Sciences* **182**: 490–507.

**Pankhurst R, Riley T, Fanning C, Kelley S.** 2000. Episodic silicic volcanism in Patagonia and the Antarctic Peninsula: Chronology of magmatism associated with the break-up of Gondwana. *Journal of Petrology* **41**: 605–625 .

**Powell GR, Tosh** **KJ.** 1991. The pollination mechanism and development after bud burst of cones of *Larix laricina*. *Canadian Journal of Botany* **69**: 1179–1187.

**Rothwell GW**. **1993**. Cordaixylon dumusum (Cordaitales). II. Reproductive Biology, Phenology, and Growth Ecology. *International Journal of Plant Sciences* **154**: 572–586.

**Rothwell GW, Mapes G, Hernandez-Castillo GR**. **2005**. *Hanskerpia* gen. nov. and phylogenetic relationships among the most ancient conifers (Voltziales). *TAXON* **54**: 733–750.

**Rothwell GW, Mapes G, Stockey RA, Hilton J**. **2012**. The seed cone *Eathiestrobus* gen. nov.: Fossil evidence for a Jurassic origin of Pinaceae. *American Journal of Botany* **99**: 708–720.

**Sahni B**. **1920**. On the structure and affinities of *Acmopyle pancheri*, Pilger. *Philosophical Transactions of the Royal Society of London, Series B* **210**: 253–310.

**Schweitzer HJ**. **1996**. *Voltzia hexagona* (Bischoff) Geinitz aus dem mittleren Perm Westdeutschlands. *Palaeontographica Abteilung B* **239**: 1–22.

**Stockey RA**. **1975**. Seeds and Embryos of *Araucaria mirabilis*. *American Journal of Botany* **62**: 856–868.

**Stockey RA, Ko H**. **1986**. Cuticle Micromorphology of *Araucaria* de Jussieu. *Botanical Gazette* **147**: 508–548.

**Stockey RA, Ko H**. **1990**. Cuticle Micromorphology of Dacrydium (Podocarpaceae) from New Caledonia. *Botanical Gazette* **151**: 138–149.

**Tomlinson PB**. **1994**. Functional Morphology of Saccate Pollen in Conifers with Special Reference to Podocarpaceae. *International Journal of Plant Sciences* **155**: 699–715.

**Townrow JA**. **1967a**. On *Rissikia* and *Mataia* podocarpaceous conifers from the lower Mesozoic of Southern Lands. *Papers and Proceedings of the Royal Society of Tasmania* **101**: 103–136.

**Townrow JA**. **1967b**. On a conifer from the Jurassic of East Antarctica. *Papers and Proceedings of the Royal Society of Tasmania* **101**: 137–147.

**Wells P, Hill R**. **1989**. Leaf morphology of the imbricate-leaved Podocarpaceae. *Australian Systematic Botany* **2**: 369.

**Wilf P**. **2012**. Rainforest conifers of Eocene Patagonia: Attached cones and foliage of the extant Southeast Asian and Australasian genus *Dacrycarpus* (Podocarpaceae). *American Journal of Botany* **99**: 562–584.

**Xu X, Li R, Dong C, Wang Q, Jin P, Sun B**. **2013**. New *Schizolepis* Fossils from the Early Cretaceous in Inner Mongolia, China and its Phylogenetic Position. *Acta Geologica Sinica - English Edition* **87**: 1250–1263.

**Yao X, Taylor TN, Taylor EL**. **1997**. A Taxodiaceous Seed Cone from the Triassic of Antarctica. *American Journal of Botany* **84**: 343–354.

**Zamuner AB, Falaschi P**. **2005**. Agathoxylon matildense n. sp., leño araucariaceo del Bosque Petrificado del cerro Madre e Hija, Formación La Matilde (Jurásico medio), provincia de Santa Cruz, Argentina. *Ameghiniana* **42**: 339–346.

**Zhang J, D’Rozario A, Yao J, Wu Z, Wang L**. **2011**. A New Species of the Extinct Genus Schizolepis from the Jurassic Daohugou Flora, Inner Mongolia, China with Special Reference to the Fossil Diversity and Evolutionary Implications. *Acta Geologica Sinica - English Edition* **85**: 471–481.
