# Supplementary material for: Revisiting the enigmatic Cheirolepidiaceae: origins, phylogenetic relationships, and a new whole-plant concept
Source: Ann Bot. 2026 Mar 30;137(6):1876–901. doi: 10.1093/aob/mcag069 (PMC13275020; doi:10.1093/aob/mcag069)
Supplement: mcag069_Supplementary_Data [file mcag069_supplementary_data.zip › AppendixS2-ParsimonyAnalyses.zip!/AppendixS2-ParsimonyAnalyses/AppendixS2C-SummarizationEqualWeights-TableResults.docx]

**Results of the Parsimony analyses**

**INDEX**

**A- No Old conifer constraints _________________________________________________p1-3**

**B- Old conifer constraints ____________________________________________________p4-5**

**A- No Old conifer constraint implemented**

**Char 64 – Unordered**

| EW – no constraints | No resolution **– 112 MPT** |
| --- | --- |
| EIW k= 15 – no constraints | Cordait and voltzialeans as stem group conifers; Cheirlepid clade as sister to *Pararaucaria* + crown conifes **– 1 MPT** |
| EIW k= 12 – no constraints | Cordait and voltzialeans as stem group conifers; Cheirlepid clade as sister to *Pararaucaria* + crown conifes **– 1 MPT** |
| EIW k= 6 – no constraints | Cordait and voltzialeans as stem group conifers; Cheirlepid clade as sister to *Pararaucaria* + crown conifes **– 1 MPT** |
| EIW k= 3 – no constraints | Cordait and voltzialeans as stem group conifers; Cheirlepid clade as sister to *Pararaucaria* + crown conifes **– 1 MPT** |
| EW – Pinaceae constraint | Rooting problems with ancient conifers; Cheirolepidiaceae monophyletic **– 6 MPT** |
| EIW k= 15 – Pinaceae constraints | Cordait and voltzialeans as stem group conifers; Cheirlepid clade as sister to *Pararaucaria* + crown conifes **– 1 MPT** |
| EIW k= 12 – Pinaceae constraints | Cordait and voltzialeans as stem group conifers; Cheirlepid clade as sister to *Pararaucaria* + crown conifes **– 1 MPT** |
| EIW k= 6 – Pinaceae constraints | Cordait and voltzialeans as stem group conifers; Cheirlepid clade as sister to *Pararaucaria* + crown conifes **– 1 MPT** |
| EIW k= 3 – Pinaceae constraints | Cordait and voltzialeans as stem group conifers; Cheirlepid clade as sister to *Pararaucaria* + crown conifes **– 1 MPT** |

**Char 64 – Ordered**

| EW – no constraints | No resolution **– 82 MPT** |
| --- | --- |
| EIW k= 15 – no constraints | Cordait and voltzialeans as stem group conifers; Cheirlepid clade as sister to *Pararaucaria* + crown conifes **– 1 MPT** |
| EIW k= 12 – no constraints | Cordait and voltzialeans as stem group conifers; Cheirlepid clade as sister to *Pararaucaria* + crown conifes **– 1 MPT** |
| EIW k= 6 – no constraints | Cordait and voltzialeans as stem group conifers; Cheirlepid clade as sister to *Pararaucaria* + crown conifes **– 1 MPT** |
| EIW k= 3 – no constraints | Cordait and voltzialeans as stem group conifers; Cheirlepid clade as sister to *Pararaucaria* + crown conifes **– 1 MPT** |
| EW – Pinaceae constraint | Rooting problems with ancient conifers; Cheirolepidiaceae monophyletic **– 6 MPT** |
| EIW k= 15 – Pinaceae constraints | Cordait and voltzialeans as stem group conifers; Cheirlepid clade as sister to *Pararaucaria* + crown conifes **– 1 MPT** |
| EIW k= 12 – Pinaceae constraints | Cordait and voltzialeans as stem group conifers; Cheirlepid clade as sister to *Pararaucaria* + crown conifes **– 1 MPT** |
| EIW k= 6 – Pinaceae constraints | Cordait and voltzialeans as stem group conifers; Cheirlepid clade as sister to *Pararaucaria* + crown conifes **– 1 MPT** |
| EIW k= 3 – Pinaceae constraints | Cordait and voltzialeans as stem group conifers; Cheirlepid clade as sister to *Pararaucaria* + crown conifes **– 1 MPT** |

**Char 0 – Stand 1**

| EW – no constraints | Rooting problems with ancient conifers; Cheirolepidiaceae monophyletic **– 20 MPT** |
| --- | --- |
| EIW k= 15 – no constraints | Cordait and voltzialeans as stem group conifers; Cheirlepid clade as sister to *Pararaucaria* + crown conifes **– 1 MPT** |
| EIW k= 12 – no constraints | Cordait and voltzialeans as stem group conifers; Cheirlepid clade as sister to *Pararaucaria* + crown conifes **– 1 MPT** |
| EIW k= 6 – no constraints | Cordait and voltzialeans as stem group conifers; Cheirlepid clade as sister to *Pararaucaria* + crown conifes **– 1 MPT** |
| EIW k= 3 – no constraints | Cordait and voltzialeans as stem group conifers; Cheirlepid clade as sister to *Pararaucaria* + crown conifes **– 1 MPT** |
| EW – Pinaceae constraint | Rooting problems with ancient conifers; Cheirolepidiaceae monophyletic **– 12 MPT** |
| EIW k= 15 – Pinaceae constraints | Cordait and voltzialeans as stem group conifers; Cheirlepid clade as sister to *Pararaucaria* + crown conifes **– 1 MPT** |
| EIW k= 12 – Pinaceae constraints | Cordait and voltzialeans as stem group conifers; Cheirlepid clade as sister to *Pararaucaria* + crown conifes **– 1 MPT** |
| EIW k= 6 – Pinaceae constraints | Cordait and voltzialeans as stem group conifers; Cheirlepid clade as sister to *Pararaucaria* + crown conifes **– 1 MPT** |
| EIW k= 3 – Pinaceae constraints | Cordait and voltzialeans as stem group conifers; Cheirlepid clade as sister to *Pararaucaria* + crown conifes **– 1 MPT** |

**Char 0 – Stand 5**

| EW – no constraints | Rooting problems with ancient conifers; Cheirolepidiaceae monophyletic **– 20 MPT** |
| --- | --- |
| EIW k= 15 – no constraints | Cordait and voltzialeans as stem group conifers; Cheirlepid clade as sister to *Pararaucaria* + crown conifes **– 1 MPT** |
| EIW k= 12 – no constraints | Cordait and voltzialeans as stem group conifers; Cheirlepid clade as sister to *Pararaucaria* + crown conifes **– 1 MPT** |
| EIW k= 6 – no constraints | Cordait and voltzialeans as stem group conifers; Cheirlepid clade as sister to *Pararaucaria* + crown conifes **– 1 MPT** |
| EIW k= 3 – no constraints | Cordait and voltzialeans as stem group conifers; Cheirlepid clade as sister to *Pararaucaria* + crown conifes **– 1 MPT** |
| EW – Pinaceae constraint | Rooting problems with ancient conifers; Cheirolepidiaceae monophyletic **– 12 MPT** |
| EIW k= 15 – Pinaceae constraints | Cordait and voltzialeans as stem group conifers; Cheirlepid clade as sister to *Pararaucaria* + crown conifes **– 1 MPT** |
| EIW k= 12 – Pinaceae constraints | Cordait and voltzialeans as stem group conifers; Cheirlepid clade as sister to *Pararaucaria* + crown conifes **– 1 MPT** |
| EIW k= 6 – Pinaceae constraints | Cordait and voltzialeans as stem group conifers; Cheirlepid clade as sister to *Pararaucaria* + crown conifes **– 1 MPT** |
| EIW k= 3 – Pinaceae constraints | Cordait and voltzialeans as stem group conifers; Cheirlepid clade as sister to *Pararaucaria* + crown conifes **– 1 MPT** |

**Char 1 – Stand 1**

| EW – no constraints | Rooting problems with ancient conifers; Cheirolepidiaceae monophyletic **– 20 MPT** |
| --- | --- |
| EIW k= 15 – no constraints | Cordait and voltzialeans as stem group conifers; Cheirlepid clade as sister to *Pararaucaria* + crown conifes **– 1 MPT** |
| EIW k= 12 – no constraints | Cordait and voltzialeans as stem group conifers; Cheirlepid clade as sister to *Pararaucaria* + crown conifes **– 1 MPT** |
| EIW k= 6 – no constraints | Cordait and voltzialeans as stem group conifers; Cheirlepid clade as sister to *Pararaucaria* + crown conifes **– 1 MPT** |
| EIW k= 3 – no constraints | Cordait and voltzialeans as stem group conifers; Cheirlepid clade as sister to *Pararaucaria* + crown conifes **– 1 MPT** |
| EW – Pinaceae constraint | Rooting problems with ancient conifers; Cheirolepidiaceae monophyletic **– 12 MPT** |
| EIW k= 15 – Pinaceae constraints | Cordait and voltzialeans as stem group conifers; Cheirlepid clade as sister to *Pararaucaria* + crown conifes **– 1 MPT** |
| EIW k= 12 – Pinaceae constraints | Cordait and voltzialeans as stem group conifers; Cheirlepid clade as sister to *Pararaucaria* + crown conifes **– 1 MPT** |
| EIW k= 6 – Pinaceae constraints | Cordait and voltzialeans as stem group conifers; Cheirlepid clade as sister to *Pararaucaria* + crown conifes **– 1 MPT** |
| EIW k= 3 – Pinaceae constraints | Cordait and voltzialeans as stem group conifers; Cheirlepid clade as sister to *Pararaucaria* + crown conifes **– 1 MPT** |

**Char 1 – Stand 5**

| EW – no constraints | Rooting problems with ancient conifers; Cheirolepidiaceae monophyletic **– 20 MPT** |
| --- | --- |
| EIW k= 15 – no constraints | Cordait and voltzialeans as stem group conifers; Cheirlepid clade as sister to *Pararaucaria* + crown conifes **– 1 MPT** |
| EIW k= 12 – no constraints | Cordait and voltzialeans as stem group conifers; Cheirlepid clade as sister to *Pararaucaria* + crown conifes **– 1 MPT** |
| EIW k= 6 – no constraints | Cordait and voltzialeans as stem group conifers; Cheirlepid clade as sister to *Pararaucaria* + crown conifes **– 1 MPT** |
| EIW k= 3 – no constraints | Cordait and voltzialeans as stem group conifers; Cheirlepid clade as sister to *Pararaucaria* + crown conifes **– 1 MPT** |
| EW – Pinaceae constraint | Rooting problems with ancient conifers; Cheirolepidiaceae monophyletic **– 12 MPT** |
| EIW k= 15 – Pinaceae constraints | Cordait and voltzialeans as stem group conifers; Cheirlepid clade as sister to *Pararaucaria* + crown conifes **– 1 MPT** |
| EIW k= 12 – Pinaceae constraints | Cordait and voltzialeans as stem group conifers; Cheirlepid clade as sister to *Pararaucaria* + crown conifes **– 1 MPT** |
| EIW k= 6 – Pinaceae constraints | Cordait and voltzialeans as stem group conifers; Cheirlepid clade as sister to *Pararaucaria* + crown conifes **– 1 MPT** |
| EIW k= 3 – Pinaceae constraints | Cordait and voltzialeans as stem group conifers; Cheirlepid clade as sister to *Pararaucaria* + crown conifes **– 1 MPT** |

**Summary**

Cordait and voltzialeans as stem group conifers; Cheirlepid clade as sister to *Pararaucaria* + crown conifes = 48/60 = 80%

Rooting problems with ancient conifers; Cheirolepidiaceae monophyletic = 10/60 = 16.67%

No resolution = 2/60 = 3.33%

**B- Old conifer constraint Results**

A positive constraint including living conifers, excluding Voltziales and Cordaitales, and treating all other fossils as floaters was implemented. Analyses were run under equal weights with and without the constraints within Pinaceae, and with the different character settings, for a total of 12 analyses.

| 1) Char 64 unord – No Pinaceae Constraints | Low resolution in the strict consensus **– 102 MPT** |
| --- | --- |
| 2) Char 64 unord – With Pinaceae Constraints | Low resolution in the strict consensus **– 97 MPT** |
| 3) Char 64 additive – No Pinaceae Constraints | Low resolution in the strict consensus **– 72 MPT** |
| 4) Char 64 additive – With Pinaceae Constraints | Low resolution in the strict consensus **– 58 MPT** |
| 5) Char 0 stand 1 – No Pinaceae Constraints | Low resolution in the strict consensus **– 14 MPT** |
| 6) Char 0 stand 1 – With Pinaceae Constraints | *Mataia* outside of crown; Cheiro monophyletic sister to Araucariales **–** **5 MPT** |
| 7) Char 0 stand 5 – No Pinaceae Constraints | Low resolution in the strict consensus **– 14 MPT** |
| 8) Char 0 stand 5 – With Pinaceae Constraints | *Mataia* outside of crown; Cheiro monophyletic sister to Araucariales **–** **5 MPT** |
| 9) Char 1 stand 1 – No Pinaceae Constraints | Low resolution in the strict consensus **– 14 MPT** |
| 10) Char 1 stand 1 – With Pinaceae Constraints | *Mataia* outside of crown; Cheiro monophyletic sister to Araucariales **–** **5 MPT** |
| 11) Char 1 stand 5 – No Pinaceae Constraints | Low resolution in the strict consensus **– 14 MPT** |
| 12) Char 1 stand 5 – With Pinaceae Constraints | *Mataia* outside of crown; Cheiro monophyletic sister to Araucariales **–** **5 MPT** |

**Alternative positions of Cheirolepidiaceae in analyses with low resolution (coral colored)**

| 1) Char 64 unord – No Pinaceae Constraints | No good resolution **– 102 MPT** |
| --- | --- |

Cheirolepidiaceae positions: (a) conifer stem group, (b) Cupressales + Araucariales stem group, (c) Araucariales stem group

| 2) Char 64 unord – With Pinaceae Constraints | No good resolution **– 97 MPT** |
| --- | --- |

Cheirolepidiaceae positions: (a) conifer stem group, (b) Araucariales stem group

| 3) Char 64 additive – No Pinaceae Constraints | No good resolution **– 72 MPT** |
| --- | --- |

Cheirolepidiaceae positions: (a) conifer stem group, (b) Cupressales + Araucariales stem group, (c) Araucariales stem group

| 4) Char 64 additive – With Pinaceae Constraints | No good resolution **– 58 MPT** |
| --- | --- |

Cheirolepidiaceae positions: (a) conifer stem group, (b) Araucariales stem group

| 5) Char 0 stand 1 – No Pinaceae Constraints | No good resolution **– 14 MPT** |
| --- | --- |

Cheirolepidiaceae positions: (a) conifer stem group, (b) Cupressales + Araucariales stem group, (c) Araucariales stem group

| 7) Char 0 stand 5 – No Pinaceae Constraints | No good resolution **– 14 MPT** |
| --- | --- |

Cheirolepidiaceae positions: (a) conifer stem group, (b) Cupressales + Araucariales stem group, (c) Araucariales stem group

| 9) Char 1 stand 1 – No Pinaceae Constraints | No good resolution **– 14 MPT** |
| --- | --- |

Cheirolepidiaceae positions: (a) conifer stem group, (b) Cupressales + Araucariales stem group, (c) Araucariales stem group

| 11) Char 1 stand 5 – No Pinaceae Constraints | No good resolution **– 14 MPT** |
| --- | --- |

Cheirolepidiaceae positions: (a) conifer stem group, (b) Cupressales + Araucariales stem group, (c) Araucariales stem group
