# Supplementary material for: Revisiting the enigmatic Cheirolepidiaceae: origins, phylogenetic relationships, and a new whole-plant concept
Source: Ann Bot. 2026 Mar 30;137(6):1876–901. doi: 10.1093/aob/mcag069 (PMC13275020; doi:10.1093/aob/mcag069)
Supplement: mcag069_Supplementary_Data [file mcag069_supplementary_data.zip › AppendixS2-ParsimonyAnalyses.zip!/AppendixS2-ParsimonyAnalyses/AppendixS2D-CommonSinapomorphies-ExtendedImpliedWeights.docx]

**Synapomorphies common to 48 trees – All MPT from EIW analyses**

(Node numbers refer to nodes in meta-consensus)

***Pararaucaria* *patagonica*:**

All trees:

No autapomorphies:

**New total plant from Arkansas:**

All trees:

No autapomorphies:

**Crown Conifers (Node 59):**

All trees:

Char. 23: 2 --> 0

(**Number of ovuliferous scale lobes**: 3–5 🡪 1)

Char. 45: 0 --> 1

(**Cortical resin canals in cone axis**: absent 🡪 present)

Char. 49: 0 --> 1

(**Resin canals in bract**: absent 🡪 present)

**Crown Conifers + *Pararaucaria* (Node 60):**

All trees:

Char. 61: 2 --> 3

(**Degree of fusion of the scale elements**: Nearly completely fused (barely lobed) 🡪 Completely fused)

**Cheirolepidiaceae + Crown Conifers (Node 61):**

**All trees:**

Char. 42: 0 --> 1

(**Seed embedded in ovuliferous scale tissue**: absent 🡪 present)

**Some trees:**

Char. 18: 2 --> 1

(**Seed number on each ovuliferous shoot or ovuliferous complex**: 3–10 🡪 2)

Char. 24: 0 --> 1

(**Cone structure**: lax 🡪 compact)

Char. 35: 3 --> 2

(**Fusion of bract and ovuliferous structure**: free 🡪 partially fused)

Char. 41: 0 --> 1

(**Ovuliferous scale base**: stalked 🡪 broad)

Char. 65: 0 --> 1

(**OC scale forms part of the diaspore**: absent 🡪 present)

Char. 103: 2 --> 1

(**Sac attachment on microsporophyll**: Adaxial 🡪 Abaxial)

**Cheirolepidiaceae without *Pararaucaria* (Node 98):**

All trees:

**Number of ovuliferous complexes in the seed cone**: 15–20 🡪 21)

**Also character 1 is recovered here but not character 67

Char. 82: 0 --> 3

(**Stomata orientation with respect to the leaf longitudinal axis**: Mostly parallel 🡪 Random)

Char. 109: 0 --> 1

(**Subequatorial canal**: Absent 🡪 Present)

Char. 110: 0 --> 1

(**Thickened equatorial band**: Absent 🡪 Present)

Char. 111: 0 --> 1

(**Distal cryptopore**: Absent 🡪 Present)

***Alvinia* + New total plant from Arkansas (Node 99):**

**All trees:**

Char. 79: 0 --> 1

(**Leaf sheath**: Absent 🡪 Present)

**Some trees:**

Char. 88: 0 --> 1

(**Papillae in Subsidiary cells**: Absent 🡪 Present)
