# Supplementary material for: Revisiting the enigmatic Cheirolepidiaceae: origins, phylogenetic relationships, and a new whole-plant concept
Source: Ann Bot. 2026 Mar 30;137(6):1876–901. doi: 10.1093/aob/mcag069 (PMC13275020; doi:10.1093/aob/mcag069)
Supplement: mcag069_Supplementary_Data [file mcag069_supplementary_data.zip › AppendixS2-ParsimonyAnalyses.zip!/AppendixS2-ParsimonyAnalyses/AppendixS2B-OldConiferConstraint-EqWeights/Appendix2Bi-Results-OldConiferConstraints.docx]

**Results of Equal weights analysis with a constraint that excludes the following taxa from the crown group of conifers** (for the constrain the crown group was defined based on extant species only and all fossils -other than the ones listed below- were considered as floaters):

- ***Cordaixylum dumusum***
- ***Emporia lockardii***
- ***Hanskerpia hamiltonensis***
- ***Voltzia hexagona***
- ***Nothodacrium warrenii***
- ***Rissikia media***
- ***Telemachus aequatus***

**In this document you will find results of the 12 equal weight analyses that were performed with the constrain detailed above. For each Analysis there is (i) a strict consensus, (ii) a majority rule consensus to show the most frequent recovered groups, and (iii) a reduced strict consensus, in which unstable taxa were removed, and their alternative positions are indicated.**

**INDEX**

**1) Character 64 unordered - no constrain Pinaceae** (102 trees)**________________p.2-4**

**2) Character 64 unordered - yes constrain Pinaceae** (97 trees)**________________p.5-7**

**3) Character 64 additive - no constrain Pinaceae** (72 trees) **________________p.8-10**

**4) Character 64 additive - yes constrain Pinaceae** (58 trees) **________________p.11-13**

**5) Character 0 standardized to 1 - no constrain Pinaceae** (14 trees)**__________p.14-16**

**6) Character** **0 standardized to 1 - yes constrain Pinaceae** (5 trees)**__________p.17-19**

**7) Character 0 standardized to 5 - no constrain Pinaceae** (14 trees)**__________p.20-22**

**8) Character 0 standardized to 5 - yes constrain Pinaceae** (5 trees)**__________p.23-25**

**9)** **Character 1 standardized to 1 - no constrain Pinaceae** (14 trees)**__________p.26-28**

**10) Character 1 standardized to 1 - yes constrain Pinaceae** (5 trees)**_________p.29-31**

**11) Character 1 standardized to 5 - no constrain Pinaceae** (14 trees)**_________p.32-34**

**12) Character 1 standardized to 5 - yes constrain** **Pinaceae** (5 trees)**_________p.35-37**

**1) Equal Weights - character 64 unordered - yes: constrain Old conifers - no: constrain Pinaceae**

(i) Strict consensus of 102 trees

,-- Ginkgo_biloba

| ,-- Cordaixylon_dumusum

|--| | ,-- Emporia_lockardii

| | |-- Hanskerpia_hamiltonensis

`--| | ,-- Voltzia_hexagona

| | | ,-- Nothodacrium_warrenii

| | | ,----- Rissikia_media

`--| | | ,-- New_total_plant_from_Arkansas

| | | |-- Alvinia_bohemica_plant

| | | |-- Telemachus_aequatus

| | | |-- Pararaucaria_patagonica

`--| | | ,-- Himeriella_muensteri

| | |--| ,-- Kachaikestrobus_acuminatus

| | | `----- Tomaxellia_biforme

| | | ,-- Sciadopitys_verticillata

| | |--| ,-- Taxus_wallichiana

| | | `--| ,-- Cunninghamia_lanceolata

`--| |	`--| ,-- Taiwania_cryptomerioides

| | `--| ,-- Athrotaxis_selaginoides

| | `--| ,-- Sequoia_sempervirens

| | `--| ,-- Cryptomeria_japonica

| | `--| ,-- Cupressus_funebris

| | `----- Callitris_muelleri

| | ,-- Araucaria_mirabilis

| | ,----- Araucaria_heterophylla

| | ,--| ,-- Wollemia_nobilis

| | | `----- Agathis_australis

`--| |	,-- Lagarostrobos_franklinii

|--| ,----- Prumnopitys_taxifolia

| | ,--| ,-- Lepidothamnus_laxifolius

| | | `----- Phyllocladus_aspleniifolius

| `--| ,-- Saxegothaea_conspicua

| | | ,-- Pherosphaera_hookeriana

| `--| |-- Microcachrys_tetragona

| `--| ,-- Acmopyle_pancheri

| `--| ,-- Dacrydium_cupressinum

| `--| ,-- Retrophyllum_rospigliosii

| `----- Podocarpus_archboldii

| ,-- Mataia_podocarpoides

| |-- Eathiestrobus_mackenziei

| |-- Schizolepidopsis_ediae

| |-- Tsuga_canadensis

| |-- Pseudolarix_amabilis

| |-- Pinus_contorta

| |-- Picea_abies

| |-- Nothotsuga_longibracteata

`--+-- Keteleeria_davidiana

|-- Cedrus_deodara

|-- Cathaya_argyrophylla

|-- Abies_lasiocarpa

| ,-- Schizolepidopsis_daohugouensis

|----- Schizolepidopsis_canicularis

| ,-- Pseudotsuga_menziesii

`----- Larix_decidua

(ii) Majority rule tree (from 102 trees, cut 50)

,-- Ginkgo_biloba

| ,-- Cordaixylon_dumusum

|--| | ,-- Emporia_lockardii

| | ,--86--- Hanskerpia_hamiltonensis

`--100|	|	,-- Voltzia_hexagona

| | | ,-- Nothodacrium_warrenii

`--100|	|	,--100--- Rissikia_media

| | | ,-- Pararaucaria_patagonica

`--100|	|	|	,-- New_total_plant_from_Arkansas

| | ,--72+--60--- Alvinia_bohemica_plant

| | | | ,-- Himeriella_muensteri

`--100|	|	`--100|	,-- Kachaikestrobus_acuminatus

| | `--100--- Tomaxellia_biforme

| | ,-- Telemachus_aequatus

| | | ,-- Eathiestrobus_mackenziei

`--100|	|	|	,-- Cedrus_deodara

| | | | ,-- Pinus_contorta

| | ,--100| | |-- Picea_abies

| | | | | |-- Cathaya_argyrophylla

| | | `--58| | ,-- Pseudotsuga_menziesii

| | | | |--100--- Larix_decidua

`--58|	|	|	|	,-- Schizolepidopsis_ediae

| | `--58+--58| ,-- Schizolepidopsis_daohugouensis

| | | `--100--- Schizolepidopsis_canicularis

| | | ,-- Mataia_podocarpoides

| | | |-- Pseudolarix_amabilis

| | | | ,-- Tsuga_canadensis

| | `--76+--70--- Nothotsuga_longibracteata

`--58|	|	,-- Keteleeria_davidiana

| `--70--- Abies_lasiocarpa

| ,-- Sciadopitys_verticillata

| ,--100| ,-- Taxus_wallichiana

| | `--100| ,-- Cunninghamia_lanceolata

| | `--100| ,-- Taiwania_cryptomerioides

| | `--100| ,-- Athrotaxis_selaginoides

| | `--100| ,-- Sequoia_sempervirens

| | `--100| ,-- Cryptomeria_japonica

| | `--100| ,-- Cupressus_funebris

`--86|	`--100--- Callitris_muelleri

| ,-- Araucaria_mirabilis

| ,--100--- Araucaria_heterophylla

| ,--100| ,-- Wollemia_nobilis

| | `--100--- Agathis_australis

| | ,-- Lagarostrobos_franklinii

`--100|	,--100--- Prumnopitys_taxifolia

| ,--100| ,-- Lepidothamnus_laxifolius

| | `--100--- Phyllocladus_aspleniifolius

`--100|	,-- Saxegothaea_conspicua

`--100|	,-- Microcachrys_tetragona

`--100|	,-- Pherosphaera_hookeriana

`--70|	,-- Acmopyle_pancheri

`--100|	,-- Dacrydium_cupressinum

`--100|	,-- Retrophyllum_r

`--100--- Podocarpus_a

(iii) Reduced strict consensus of 102 trees

,-- Ginkgo_biloba

| ,-- Cordaixylon_dumusum

|--| | ,-- Emporia_lockardii

| | |-- Hanskerpia_hamiltonensis

`--| | ,-- Voltzia_hexagona

| | | ,-- Nothodacrium_warrenii

`--| | ,----- Rissikia_media

| | | ,-- Mataia_podocarpoides

| | | |-- Eathiestrobus_mackenziei

| | | |-- Schizolepidopsis_ediae

`--| |	|-- Tsuga_canadensis

| | |-- Pseudolarix_amabilis

| | |-- Pinus_contorta

| | |-- Picea_abies

| | |-- Nothotsuga_longibracteata

`--|	,--+-- Keteleeria_davidiana

| | |-- Cedrus_deodara

| | |-- Cathaya_argyrophylla

| | |-- Abies_lasiocarpa

| | | ,-- Schizolepidopsis_daohugouensis

| | |----- Schizolepidopsis_canicularis

| | | ,-- Pseudotsuga_menziesii

| | `----- Larix_decidua

`--abcde|	,-- Sciadopitys_verticillata

| ,--| ,-- Taxus_wallichiana

| | `--| ,-- Cunninghamia_lanceolata

| | `--| ,-- Taiwania_cryptomerioides

| | `--| ,-- Athrotaxis_selaginoides

| | `--| ,-- Sequoia_sempervirens

| | `--| ,-- Cryptomeria_japonica

| | `--| ,-- Cupressus_funebris

`--acde|	`----- Callitris_muelleri

| ,-- Araucaria_mirabilis

| ,----- Araucaria_heterophylla

| ,--| ,-- Wollemia_nobilis

| | `----- Agathis_australis

| | ,-- Lagarostrobos_franklinii

`--acde|	,----- Prumnopitys_taxifolia

| ,--| ,-- Lepidothamnus_laxifolius

| | `----- Phyllocladus_aspleniifolius

`--| ,-- Saxegothaea_conspicua

| | ,-- Pherosphaera_hookeriana

`--| |-- Microcachrys_tetragona

`--| ,-- Acmopyle_pancheri

`--| ,-- Dacrydium_cupressinum

`--| ,-- Retrophyllum_rospigliosii

**Legends:** `----- Podocarpus_archboldii

a: Pararaucaria_patagonica (36)

b: Telemachus_aequatus (40)

c: Alvinia_bohemica_plant (52)

d: New_total_plant_from_Arkansas (53)

e: node 87 of consensus

,-- Himeriella_muensteri

|--| ,-- Kachaikestrobus_acuminatus

`----- Tomaxellia_biforme

**2) Equal Weights - character 64 unordered - yes: constrain Old conifers - yes: constrain Pinaceae**

(i) Strict consensus of 97 trees

,-- Ginkgo_biloba

| ,-- Cordaixylon_dumusum

|--| | ,-- Emporia_lockardii

| | ,----- Hanskerpia_hamiltonensis

`--| | ,-- Voltzia_hexagona

| | | ,-- Nothodacrium_warrenii

| | | ,----- Rissikia_media

`--| | | ,-- Mataia_podocarpoides

| | | |-- Eathiestrobus_mackenziei

| | | |-- Telemachus_aequatus

`--| | |-- Schizolepidopsis_ediae

| | |-- Tsuga_canadensis

| | |-- Pseudolarix_amabilis

| | |-- Pinus_contorta

| | |-- Picea_abies

| | |-- Nothotsuga_longibracteata

`--| |-- Keteleeria_davidiana

| |-- Cedrus_deodara

| |-- Cathaya_argyrophylla

| |-- Abies_lasiocarpa

| | ,-- Schizolepidopsis_daohugouensis

| |----- Schizolepidopsis_canicularis

| | ,-- Pseudotsuga_menziesii

| |----- Larix_decidua

| | ,-- New_total_plant_from_Arkansas

| | |-- Alvinia_bohemica_plant

`--+--+-- Pararaucaria_patagonica

| | ,-- Himeriella_muensteri

| `--| ,-- Kachaikestrobus_acuminatus

| `----- Tomaxellia_biforme

| ,-- Sciadopitys_verticillata

|--| ,-- Taxus_wallichiana

| `--| ,-- Cunninghamia_lanceolata

| `--| ,-- Taiwania_cryptomerioides

| `--| ,-- Athrotaxis_selaginoides

| `--| ,-- Sequoia_sempervirens

| `--| ,-- Cryptomeria_japonica

| `--| ,-- Cupressus_funebris

| `----- Callitris_muelleri

| ,-- Araucaria_mirabilis

| ,----- Araucaria_heterophylla

| ,--| ,-- Wollemia_nobilis

| | `----- Agathis_australis

| | ,-- Lagarostrobos_franklinii

`--|	,----- Prumnopitys_taxifolia

| ,--| ,-- Lepidothamnus_laxifolius

| | `----- Phyllocladus_aspleniifolius

`--| ,-- Saxegothaea_conspicua

| | ,-- Pherosphaera_hookeriana

`--| |-- Microcachrys_tetragona

`--| ,-- Acmopyle_pancheri

`--| ,-- Dacrydium_cupressinum

`--| ,-- Retrophyllum_rospigliosii

`----- Podocarpus_archboldii

(ii) Majority rule tree (from 97 trees, cut 50)

,-- Ginkgo_biloba

| ,-- Cordaixylon_dumusum

|--| | ,-- Emporia_lockardii

| | ,--100--- Hanskerpia_hamiltonensis

`--100|	|	,-- Voltzia_hexagona

| | | ,-- Nothodacrium_warrenii

`--100|	|	,--100--- Rissikia_media

| | | ,-- New_total_plant_from_Arkansas

`--100|	|	|-- Alvinia_bohemica_plant

| | ,--100+-- Pararaucaria_patagonica

| | | | ,-- Himeriella_muensteri

`--100|	|	`--100|	,-- Kachaikestrobus_acuminatus

| | `--100--- Tomaxellia_biforme

| | ,-- Telemachus_aequatus

| | | ,-- Eathiestrobus_mackenziei

`--100|	|	|	,-- Cedrus_deodara

| | | | ,-- Mataia_podocarpoides

| | | ,--70| |-- Pseudolarix_amabilis

| | ,--94| | | | ,-- Tsuga_canadensis

| | | | | `--51+--77--- Nothotsuga_longibracteata

| | | | | | ,-- Keteleeria_davidiana

`--80|	|	`--68|	`--77--- Abies_lasiocarpa

| | | ,-- Pseudotsuga_menziesii

| | | ,--100--- Larix_decidua

| | `--62| ,-- Cathaya_argyrophylla

| | `--62| ,-- Pinus_contorta

| | `--62| ,-- Picea_abies

| | `--62| ,-- Schizolepidopsis_ediae

`--80|	`--85|	,-- Schizolepidopsis_daohugouensis

| `--100--- Schizolepidopsis_canicularis

| ,-- Sciadopitys_verticillata

| ,--100| ,-- Taxus_wallichiana

| | `--100| ,-- Cunninghamia_lanceolata

| | `--100| ,-- Taiwania_cryptomerioides

| | `--100| ,-- Athrotaxis_selaginoides

| | `--100| ,-- Sequoia_sempervirens

| | `--100| ,-- Cryptomeria_japonica

| | `--100| ,-- Cupressus_funebris

`--80|	`--100--- Callitris_muelleri

| ,-- Araucaria_mirabilis

| ,--100--- Araucaria_heterophylla

| ,--100| ,-- Wollemia_nobilis

| | `--100--- Agathis_australis

| | ,-- Lagarostrobos_franklinii

`--100|	,--100--- Prumnopitys_taxifolia

| ,--100| ,-- Lepidothamnus_laxifolius

| | `--100--- Phyllocladus_aspleniifolius

`--100|	,-- Saxegothaea_conspicua

`--100|	,-- Microcachrys_tetragona

`--100|	,-- Pherosphaera_hookeriana

`--59|	,-- Acmopyle_pancheri

`--100|	,-- Dacrydium_cupressinum

`--100|	,-- Retrophyllum_rospigl

`--100--- Podocarpus_archbold

(iii) Reduced strict consensus of 97 trees

,-- Ginkgo_biloba

| ,-- Cordaixylon_dumusum

|--| | ,-- Emporia_lockardii

| | ,----- Hanskerpia_hamiltonensis

`--| | ,-- Voltzia_hexagona

| | | ,-- Nothodacrium_warrenii

`--| | ,----- Rissikia_media

| | | ,-- Mataia_podocarpoides

| | | |-- Eathiestrobus_mackenziei

`--| |	|-- Schizolepidopsis_ediae

| | |-- Tsuga_canadensis

| | |-- Pseudolarix_amabilis

| | |-- Pinus_contorta

`--|	|-- Picea_abies

| |-- Nothotsuga_longibracteata

| |-- Keteleeria_davidiana

| |-- Cedrus_deodara

| |-- Cathaya_argyrophylla

| |-- Abies_lasiocarpa

`--ab| ,-- Schizolepidopsis_daohugouensis

|----- Schizolepidopsis_canicularis

| ,-- Pseudotsuga_menziesii

|----- Larix_decidua

| ,-- Sciadopitys_verticillata

| ,--| ,-- Taxus_wallichiana

| | `--| ,-- Cunninghamia_lanceolata

| | `--| ,-- Taiwania_cryptomerioides

| | `--| ,-- Athrotaxis_selaginoides

| | `--| ,-- Sequoia_sempervirens

| | `--| ,-- Cryptomeria_japonica

| | `--| ,-- Cupressus_funebris

`--|	`----- Callitris_muelleri

| ,-- Araucaria_mirabilis

| ,----- Araucaria_heterophylla

| ,--| ,-- Wollemia_nobilis

| | `----- Agathis_australis

| | ,-- Lagarostrobos_franklinii

`--b|	,----- Prumnopitys_taxifolia

| ,--| ,-- Lepidothamnus_laxifolius

| | `----- Phyllocladus_aspleniifolius

`--| ,-- Saxegothaea_conspicua

| | ,-- Pherosphaera_hookeriana

`--| |-- Microcachrys_tetragona

`--| ,-- Acmopyle_pancheri

`--| ,-- Dacrydium_cupressinum

`--| ,-- Retrophyllum_rospigliosii

**Legends:** `----- Podocarpus_archboldii

a: Telemachus_aequatus (40)

b: node 83 of consensus

,-- New_total_plant_from_Arkansas

|-- Alvinia_bohemica_plant

|--+-- Pararaucaria_patagonica

| ,-- Himeriella_muensteri

`--| ,-- Kachaikestrobus_acuminatus

`----- Tomaxellia_biforme

**3) Equal Weights - character 64 additive - yes: constrain Old conifers - no: constrain Pinaceae**

(i) Strict consensus of 72 trees

,-- Ginkgo_biloba

| ,-- Cordaixylon_dumusum

|--| | ,-- Emporia_lockardii

| | |-- Hanskerpia_hamiltonensis

`--| | ,-- Voltzia_hexagona

| | | ,-- Nothodacrium_warrenii

| | | ,----- Rissikia_media

`--| | | ,-- New_total_plant_from_Arkansas

| | | |-- Alvinia_bohemica_plant

| | | |-- Telemachus_aequatus

| | | |-- Pararaucaria_patagonica

`--| | | ,-- Himeriella_muensteri

| | |--| ,-- Kachaikestrobus_acuminatus

| | | `----- Tomaxellia_biforme

| | | ,-- Sciadopitys_verticillata

| | |--| ,-- Taxus_wallichiana

| | | `--| ,-- Cunninghamia_lanceolata

`--| |	`--| ,-- Taiwania_cryptomerioides

| | `--| ,-- Athrotaxis_selaginoides

| | `--| ,-- Sequoia_sempervirens

| | `--| ,-- Cryptomeria_japonica

| | `--| ,-- Cupressus_funebris

| | `----- Callitris_muelleri

| | ,-- Araucaria_mirabilis

| | ,----- Araucaria_heterophylla

| | ,--| ,-- Wollemia_nobilis

| | | `----- Agathis_australis

`--| |	,-- Lagarostrobos_franklinii

|--| ,----- Prumnopitys_taxifolia

| | ,--| ,-- Lepidothamnus_laxifolius

| | | `----- Phyllocladus_aspleniifolius

| `--| ,-- Saxegothaea_conspicua

| `--| ,-- Microcachrys_tetragona

| `--| ,-- Pherosphaera_hookeriana

| `--| ,-- Acmopyle_pancheri

| `--| ,-- Dacrydium_cupressinum

| `--| ,-- Retrophyllum_rospigliosii

| `----- Podocarpus_archboldii

| ,-- Mataia_podocarpoides

| |-- Eathiestrobus_mackenziei

| |-- Schizolepidopsis_ediae

| |-- Tsuga_canadensis

| |-- Pseudolarix_amabilis

| |-- Pinus_contorta

| |-- Picea_abies

| |-- Nothotsuga_longibracteata

`--+-- Keteleeria_davidiana

|-- Cedrus_deodara

|-- Cathaya_argyrophylla

|-- Abies_lasiocarpa

| ,-- Schizolepidopsis_daohugouensis

|----- Schizolepidopsis_canicularis

| ,-- Pseudotsuga_menziesii

`----- Larix_decidua

(ii) Majority rule tree (from 72 trees, cut 50)

,-- Ginkgo_biloba

| ,-- Cordaixylon_dumusum

|--| | ,-- Emporia_lockardii

| | ,--80--- Hanskerpia_hamiltonensis

`--100|	|	,-- Voltzia_hexagona

| | | ,-- Nothodacrium_warrenii

`--100|	|	,--100--- Rissikia_media

| | | ,-- Telemachus_aequatus

`--100|	|	|	,-- Schizolepidopsis_ediae

| | | | ,-- Schizolepidopsis_daohugouensis

| | | |--100--- Schizolepidopsis_canicularis

`--100|	|	|	,-- Cedrus_deodara

| | ,--100| | ,-- Mataia_podocarpoides

| | | | | |-- Pseudolarix_amabilis

| | | | | | ,-- Tsuga_canadensis

`--100|	|	`--58|	,--83+--70--- Nothotsuga_longibracteata

| | | | | ,-- Keteleeria_davidiana

| | | | `--70--- Abies_lasiocarpa

| | `--58| ,-- Pseudotsuga_menziesii

| | | ,--100--- Larix_decidua

| | `--58| ,-- Cathaya_argyrophylla

| | `--58| ,-- Eathiestrobus_mackenziei

`--58|	`--58+-- Pinus_contorta

| `-- Picea_abies

| ,-- Pararaucaria_patagonica

| | ,-- New_total_plant_from_Arkansas

| ,--61| ,--72--- Alvinia_bohemica_plant

| | `--58| ,-- Himeriella_muensteri

| | `--100| ,-- Kachaikestrobus_acuminatus

| | `--100--- Tomaxellia_biforme

| | ,-- Sciadopitys_verticillata

| | ,--100| ,-- Taxus_wallichiana

`--58|	|	`--100|	,-- Cunninghamia_lanceolata

| | `--100| ,-- Taiwania_cryptomerioides

| | `--100| ,-- Athrotaxis_selaginoides

| | `--100| ,-- Sequoia_sempervirens

| | `--100| ,-- Cryptomeria_japonica

| | `--100| ,-- Cupressus_funebris

`--80|	`--100--- Callitris_muelleri

| ,-- Araucaria_mirabilis

| ,--100--- Araucaria_heterophylla

| ,--100| ,-- Wollemia_nobilis

| | `--100--- Agathis_australis

| | ,-- Lagarostrobos_franklinii

`--100|	,--100--- Prumnopitys_taxifolia

| ,--100| ,-- Lepidothamnus_laxifolius

| | `--100--- Phyllocladus_aspleniifolius

`--100|	,-- Saxegothaea_conspicua

`--100|	,-- Microcachrys_tetragona

`--100|	,-- Pherosphaera_hookeriana

`--100|	,-- Acmopyle_pancheri

`--100|	,-- Dacrydium_cupressinum

`--100|	,-- Retrophyllum_rospig

`--100--- Podocarpus_archbol

(iii) Reduced strict consensus of 72 trees

,-- Ginkgo_biloba

| ,-- Cordaixylon_dumusum

|--| | ,-- Emporia_lockardii

| | |-- Hanskerpia_hamiltonensis

`--| | ,-- Voltzia_hexagona

| | | ,-- Nothodacrium_warrenii

`--| | ,----- Rissikia_media

| | | ,-- Mataia_podocarpoides

| | | |-- Eathiestrobus_mackenziei

| | | |-- Schizolepidopsis_ediae

`--| |	|-- Tsuga_canadensis

| | |-- Pseudolarix_amabilis

| | |-- Pinus_contorta

| | |-- Picea_abies

| | |-- Nothotsuga_longibracteata

`--|	,--+-- Keteleeria_davidiana

| | |-- Cedrus_deodara

| | |-- Cathaya_argyrophylla

| | |-- Abies_lasiocarpa

| | | ,-- Schizolepidopsis_daohugouensis

| | |----- Schizolepidopsis_canicularis

| | | ,-- Pseudotsuga_menziesii

| | `----- Larix_decidua

`--abcde|	,-- Sciadopitys_verticillata

| ,--| ,-- Taxus_wallichiana

| | `--| ,-- Cunninghamia_lanceolata

| | `--| ,-- Taiwania_cryptomerioides

| | `--| ,-- Athrotaxis_selaginoides

| | `--| ,-- Sequoia_sempervirens

| | `--| ,-- Cryptomeria_japonica

| | `--| ,-- Cupressus_funebris

`--acde|	`----- Callitris_muelleri

| ,-- Araucaria_mirabilis

| ,----- Araucaria_heterophylla

| ,--| ,-- Wollemia_nobilis

| | `----- Agathis_australis

| | ,-- Lagarostrobos_franklinii

`--acde|	,----- Prumnopitys_taxifolia

| ,--| ,-- Lepidothamnus_laxifolius

| | `----- Phyllocladus_aspleniifolius

`--| ,-- Saxegothaea_conspicua

`--| ,-- Microcachrys_tetragona

`--| ,-- Pherosphaera_hookeriana

`--| ,-- Acmopyle_pancheri

`--| ,-- Dacrydium_cupressinum

`--| ,-- Retrophyll_rospigliosii

**Legends:** `----- Podocarpus_archboldii

a: Pararaucaria_patagonica (36)

b: Telemachus_aequatus (40)

c: Alvinia_bohemica_plant (52)

d: New_total_plant_from_Arkansas (53)

e: node 88 of consensus

,-- Himeriella_muensteri

|--| ,-- Kachaikestrobus_acuminatus

`----- Tomaxellia_biforme

**4) Equal Weights - character 64 additive - yes: constrain Old conifers - yes: constrain Pinaceae**

(i) Strict consensus of 58 trees

,-- Ginkgo_biloba

| ,-- Cordaixylon_dumusum

|--| | ,-- Emporia_lockardii

| | ,----- Hanskerpia_hamiltonensis

`--| | ,-- Voltzia_hexagona

| | | ,-- Nothodacrium_warrenii

| | | ,----- Rissikia_media

`--| | | ,-- Mataia_podocarpoides

| | | |-- Eathiestrobus_mackenziei

| | | |-- Telemachus_aequatus

`--| | |-- Schizolepidopsis_ediae

| | |-- Tsuga_canadensis

| | |-- Pseudolarix_amabilis

| | |-- Pinus_contorta

| | |-- Picea_abies

| | |-- Nothotsuga_longibracteata

`--| |-- Keteleeria_davidiana

| |-- Cedrus_deodara

| |-- Cathaya_argyrophylla

| |-- Abies_lasiocarpa

| | ,-- Schizolepidopsis_daohugouensis

| |----- Schizolepidopsis_canicularis

| | ,-- Pseudotsuga_menziesii

| |----- Larix_decidua

| | ,-- New_total_plant_from_Arkansas

| | |-- Alvinia_bohemica_plant

`--+--+-- Pararaucaria_patagonica

| | ,-- Himeriella_muensteri

| `--| ,-- Kachaikestrobus_acuminatus

| `----- Tomaxellia_biforme

| ,-- Sciadopitys_verticillata

|--| ,-- Taxus_wallichiana

| `--| ,-- Cunninghamia_lanceolata

| `--| ,-- Taiwania_cryptomerioides

| `--| ,-- Athrotaxis_selaginoides

| `--| ,-- Sequoia_sempervirens

| `--| ,-- Cryptomeria_japonica

| `--| ,-- Cupressus_funebris

| `----- Callitris_muelleri

| ,-- Araucaria_mirabilis

| ,----- Araucaria_heterophylla

| ,--| ,-- Wollemia_nobilis

| | `----- Agathis_australis

| | ,-- Lagarostrobos_franklinii

`--|	,----- Prumnopitys_taxifolia

| ,--| ,-- Lepidothamnus_laxifolius

| | `----- Phyllocladus_aspleniifolius

`--| ,-- Saxegothaea_conspicua

`--| ,-- Microcachrys_tetragona

`--| ,-- Pherosphaera_hookeriana

`--| ,-- Acmopyle_pancheri

`--| ,-- Dacrydium_cupressinum

`--| ,-- Retrophyllum_rospigliosii

`----- Podocarpus_archboldii

(ii) Majority rule tree (from 58 trees, cut 50)

,-- Ginkgo_biloba

| ,-- Cordaixylon_dumusum

|--| | ,-- Emporia_lockardii

| | ,--100--- Hanskerpia_hamiltonensis

`--100|	|	,-- Voltzia_hexagona

| | | ,-- Nothodacrium_warrenii

`--100|	|	,--100--- Rissikia_media

| | | ,-- Pararaucaria_patagonica

`--100|	|	|	,-- New_total_plant_from_Arkansas

| | ,--100+--55--- Alvinia_bohemica_plant

| | | | ,-- Himeriella_muensteri

`--100|	|	`--100|	,-- Kachaikestrobus_acuminatus

| | `--100--- Tomaxellia_biforme

| | ,-- Telemachus_aequatus

| | | ,-- Eathiestrobus_mackenziei

`--100|	|	|	,-- Cedrus_deodara

| | | | ,-- Mataia_podocarpoides

| | | ,--70| |-- Pseudolarix_amabilis

| | ,--91| | | | ,-- Tsuga_canadensis

| | | | | `--55+--77--- Nothotsuga_longibracteata

| | | | | | ,-- Keteleeria_davidiana

`--67|	|	`--56|	`--77--- Abies_lasiocarpa

| | | ,-- Pseudotsuga_menziesii

| | | ,--100--- Larix_decidua

| | `--53| ,-- Cathaya_argyrophylla

| | `--53| ,-- Pinus_contorta

| | `--53| ,-- Picea_abies

| | `--53| ,-- Schizolepidopsis_ediae

`--67|	`--75|	,-- Schizolepidopsis_daohugouensis

| `--100--- Schizolepidopsis_canicularis

| ,-- Sciadopitys_verticillata

| ,--100| ,-- Taxus_wallichiana

| | `--100| ,-- Cunninghamia_lanceolata

| | `--100| ,-- Taiwania_cryptomerioides

| | `--100| ,-- Athrotaxis_selaginoides

| | `--100| ,-- Sequoia_sempervirens

| | `--100| ,-- Cryptomeria_japonica

| | `--100| ,-- Cupressus_funebris

`--67|	`--100--- Callitris_muelleri

| ,-- Araucaria_mirabilis

| ,--100--- Araucaria_heterophylla

| ,--100| ,-- Wollemia_nobilis

| | `--100--- Agathis_australis

| | ,-- Lagarostrobos_franklinii

`--100|	,--100--- Prumnopitys_taxifolia

| ,--100| ,-- Lepidothamnus_laxifolius

| | `--100--- Phyllocladus_aspleniifolius

`--100|	,-- Saxegothaea_conspicua

`--100|	,-- Microcachrys_tetragona

`--100|	,-- Pherosphaera_hookeriana

`--100|	,-- Acmopyle_pancheri

`--100|	,-- Dacrydium_cupressinum

`--100|	,-- Retrophyllum_rospig

`--100--- Podocarpus_archbold

(iii) Reduced strict consensus of 58 trees

,-- Ginkgo_biloba

| ,-- Cordaixylon_dumusum

|--| | ,-- Emporia_lockardii

| | ,----- Hanskerpia_hamiltonensis

`--| | ,-- Voltzia_hexagona

| | | ,-- Nothodacrium_warrenii

`--| | ,----- Rissikia_media

| | | ,-- Mataia_podocarpoides

| | | |-- Eathiestrobus_mackenziei

`--| |	|-- Schizolepidopsis_ediae

| | |-- Tsuga_canadensis

| | |-- Pseudolarix_amabilis

| | |-- Pinus_contorta

`--|	|-- Picea_abies

| |-- Nothotsuga_longibracteata

| |-- Keteleeria_davidiana

| |-- Cedrus_deodara

| |-- Cathaya_argyrophylla

| |-- Abies_lasiocarpa

`--ab| ,-- Schizolepidopsis_daohugouensis

|----- Schizolepidopsis_canicularis

| ,-- Pseudotsuga_menziesii

|----- Larix_decidua

| ,-- Sciadopitys_verticillata

| ,--| ,-- Taxus_wallichiana

| | `--| ,-- Cunninghamia_lanceolata

| | `--| ,-- Taiwania_cryptomerioides

| | `--| ,-- Athrotaxis_selaginoides

| | `--| ,-- Sequoia_sempervirens

| | `--| ,-- Cryptomeria_japonica

| | `--| ,-- Cupressus_funebris

`--|	`----- Callitris_muelleri

| ,-- Araucaria_mirabilis

| ,----- Araucaria_heterophylla

| ,--| ,-- Wollemia_nobilis

| | `----- Agathis_australis

| | ,-- Lagarostrobos_franklinii

`--b|	,----- Prumnopitys_taxifolia

| ,--| ,-- Lepidothamnus_laxifolius

| | `----- Phyllocladus_aspleniifolius

`--| ,-- Saxegothaea_conspicua

`--| ,-- Microcachrys_tetragona

`--| ,-- Pherosphaera_hookeriana

`--| ,-- Acmopyle_pancheri

`--| ,-- Dacrydium_cupressinum

`--| ,-- Retrophyllum_rospigliosii

**Legends:** `----- Podocarpus_archboldii

a: Telemachus_aequatus (40)

b: node 84 of consensus

,-- New_total_plant_from_Arkansas

|-- Alvinia_bohemica_plant

|--+-- Pararaucaria_patagonica

| ,-- Himeriella_muensteri

`--| ,-- Kachaikestrobus_acuminatus

`----- Tomaxellia_biforme

**5) Equal Weights - character 0 standardized to 1 - yes: constrain Old conifers - no: constrain Pinaceae**

(i) Strict consensus of 14 trees

,-- Ginkgo_biloba

| ,-- Cordaixylon_dumusum

|--| | ,-- Emporia_lockardii

| | |-- Hanskerpia_hamiltonensis

`--| | ,-- Voltzia_hexagona

| | | ,-- Nothodacrium_warrenii

`--| | |-- Rissikia_media

| | | ,-- New_total_plant_from_Arkansas

| | | |-- Alvinia_bohemica_plant

| | | |-- Telemachus_aequatus

`--| | |-- Pararaucaria_patagonica

| | | ,-- Himeriella_muensteri

| | |--| ,-- Kachaikestrobus_acuminatus

| | | `----- Tomaxellia_biforme

| | | ,-- Sciadopitys_verticillata

| | |--| ,-- Taxus_wallichiana

`--| | `--| ,-- Cunninghamia_lanceolata

| | `--| ,-- Taiwania_cryptomerioides

| | `--| ,-- Athrotaxis_selaginoides

| | `--| ,-- Sequoia_sempervirens

| | `--| ,-- Cryptomeria_japonica

| | `--| ,-- Cupressus_funebris

| | `----- Callitris_muelleri

| | ,-- Araucaria_mirabilis

| | ,----- Araucaria_heterophylla

| | ,--| ,-- Wollemia_nobilis

`--| | `----- Agathis_australis

| | ,-- Lagarostrobos_franklinii

|--| ,----- Prumnopitys_taxifolia

| | ,--| ,-- Lepidothamnus_laxifolius

| | | `----- Phyllocladus_aspleniifolius

| `--| ,-- Saxegothaea_conspicua

| | | ,-- Pherosphaera_hookeriana

| `--| |-- Microcachrys_tetragona

| `--| ,-- Acmopyle_pancheri

| `--| ,-- Dacrydium_cupressinum

| `--| ,-- Retrophyllum_rospigliosii

| `----- Podocarpus_archboldii

| ,-- Eathiestrobus_mackenziei

| |-- Schizolepidopsis_ediae

| |-- Pinus_contorta

| |-- Picea_abies

| |-- Cedrus_deodara

| |-- Cathaya_argyrophylla

| | ,-- Schizolepidopsis_daohugouensis

`--+----- Schizolepidopsis_canicularis

| ,-- Pseudotsuga_menziesii

|----- Larix_decidua

| ,-- Keteleeria_davidiana

| ,----- Abies_lasiocarpa

| | ,-- Mataia_podocarpoides

`--| ,----- Pseudolarix_amabilis

`--| ,-- Tsuga_canadensis

`----- Nothotsuga_longibracteata

(ii) Majority rule tree (from 14 trees, cut 50)

,-- Ginkgo_biloba

| ,-- Cordaixylon_dumusum

|--| | ,-- Emporia_lockardii

| | ,--71--- Hanskerpia_hamiltonensis

`--100|	|	,-- Voltzia_hexagona

| | | ,-- Nothodacrium_warrenii

`--100|	|	,--85--- Rissikia_media

| | | ,-- Telemachus_aequatus

`--100|	|	|	,-- Schizolepidopsis_ediae

| | | | ,-- Schizolepidopsis_daohugouensis

| | | |--100--- Schizolepidopsis_canicularis

`--100|	|	|	,-- Cedrus_deodara

| | ,--100| | ,-- Keteleeria_davidiana

| | | | | ,--100--- Abies_lasiocarpa

| | | | | | ,-- Mataia_podocarpoides

`--100|	|	`--57|	,--100|	|-- Pseudolarix_amabilis

| | | | `--100| ,-- Tsuga_canadensis

| | | | `--100--- Nothotsuga_longibracteata

| | `--57| ,-- Pseudotsuga_menziesii

| | | ,--100--- Larix_decidua

| | `--57| ,-- Cathaya_argyrophylla

| | `--57| ,-- Eathiestrobus_mackenziei

`--57|	`--57+-- Pinus_contorta

| `-- Picea_abies

| ,-- Pararaucaria_patagonica

| | ,-- New_total_plant_from_Arkansas

| ,--57| ,--71--- Alvinia_bohemica_plant

| | `--57| ,-- Himeriella_muensteri

| | `--100| ,-- Kachaikestrobus_acuminatus

| | `--100--- Tomaxellia_biforme

| | ,-- Sciadopitys_verticillata

| | ,--100| ,-- Taxus_wallichiana

`--57|	|	`--100|	,-- Cunninghamia_lanceolata

| | `--100| ,-- Taiwania_cryptomerioides

| | `--100| ,-- Athrotaxis_selaginoides

| | `--100| ,-- Sequoia_sempervirens

| | `--100| ,-- Cryptomeria_japonica

| | `--100| ,-- Cupressus_funebris

`--85|	`--100--- Callitris_muelleri

| ,-- Araucaria_mirabilis

| ,--100--- Araucaria_heterophylla

| ,--100| ,-- Wollemia_nobilis

| | `--100--- Agathis_australis

| | ,-- Lagarostrobos_franklinii

`--100|	,--100--- Prumnopitys_taxifolia

| ,--100| ,-- Lepidothamnus_laxifolius

| | `--100--- Phyllocladus_aspleniifolius

`--100|	,-- Saxegothaea_conspicua

`--100|	,-- Microcachrys_tetragona

`--100|	,-- Pherosphaera_hookeriana

`--78|	,-- Acmopyle_pancheri

`--100|	,-- Dacrydium_cupressinum

`--100|	,-- Retrophyllum_rospigl

`--100--- Podocarpus_archbol

(iii) Reduced strict consensus of 14 trees

,-- Ginkgo_biloba

| ,-- Cordaixylon_dumusum

|--| | ,-- Emporia_lockardii

| | |-- Hanskerpia_hamiltonensis

`--| | ,-- Voltzia_hexagona

| | | ,-- Nothodacrium_warrenii

`--| | |-- Rissikia_media

| | | ,-- Eathiestrobus_mackenziei

| | | |-- Schizolepidopsis_ediae

`--| |	|-- Pinus_contorta

| | |-- Picea_abies

| | |-- Cedrus_deodara

| | |-- Cathaya_argyrophylla

| | | ,-- Schizolepidopsis_daohugouensis

`--|	,--+----- Schizolepidopsis_canicularis

| | | ,-- Pseudotsuga_menziesii

| | |----- Larix_decidua

| | | ,-- Keteleeria_davidiana

| | | ,----- Abies_lasiocarpa

| | | | ,-- Mataia_podocarpoides

| | `--| |-- Pseudolarix_amabilis

| | `--| ,-- Tsuga_canadensis

| | `----- Nothotsuga_longibracteata

`--abcde|	,-- Sciadopitys_verticillata

| ,--| ,-- Taxus_wallichiana

| | `--| ,-- Cunninghamia_lanceolata

| | `--| ,-- Taiwania_cryptomerioides

| | `--| ,-- Athrotaxis_selaginoides

| | `--| ,-- Sequoia_sempervirens

| | `--| ,-- Cryptomeria_japonica

| | `--| ,-- Cupressus_funebris

`--acde|	`----- Callitris_muelleri

| ,-- Araucaria_mirabilis

| ,----- Araucaria_heterophylla

| ,--| ,-- Wollemia_nobilis

| | `----- Agathis_australis

| | ,-- Lagarostrobos_franklinii

`--acde|	,----- Prumnopitys_taxifolia

| ,--| ,-- Lepidothamnus_laxifolius

| | `----- Phyllocladus_aspleniifolius

`--| ,-- Saxegothaea_conspicua

| | ,-- Pherosphaera_hookeriana

`--| |-- Microcachrys_tetragona

`--| ,-- Acmopyle_pancheri

`--| ,-- Dacrydium_cupressinum

`--| ,-- Retrophyllum_rospigliosii

**Legends:** `----- Podocarpus_archboldii

a: Pararaucaria_patagonica (36)

b: Telemachus_aequatus (40)

c: Alvinia_bohemica_plant (52)

d: New_total_plant_from_Arkansas (53)

e: node 90 of consensus

,-- Himeriella_muensteri

|--| ,-- Kachaikestrobus_acuminatus

`----- Tomaxellia_biforme

**6) Equal Weights - character 0 standardized to 1 - yes: constrain Old conifers - yes: constrain Pinaceae**

(i) Strict consensus of 5 trees

,-- Ginkgo_biloba

| ,-- Cordaixylon_dumusum

|--| | ,-- Emporia_lockardii

`--| ,----- Hanskerpia_hamiltonensis

| | ,-- Voltzia_hexagona

`--| |	,-- Nothodacrium_warrenii

| | ,----- Rissikia_media

`--| | ,-- Telemachus_aequatus

| | | ,-- Mataia_podocarpoides

`--| | |	,-- Eathiestrobus_mackenziei

| | | |-- Pseudolarix_amabilis

| | | |-- Pinus_contorta

`--| |	|-- Picea_abies

| | |-- Cedrus_deodara

| | |-- Cathaya_argyrophylla

| | | ,-- Tsuga_canadensis

| | ,--+----- Nothotsuga_longibracteata

`--| | | ,-- Pseudotsuga_menziesii

| | |----- Larix_decidua

| | | ,-- Keteleeria_davidiana

| | |----- Abies_lasiocarpa

| | | ,-- Schizolepidopsis_ediae

| | `--| ,-- Schizolepidopsis_daohugouensis

| | `----- Schizolepidopsis_canicularis

| | ,-- Sciadopitys_verticillata

`--| ,--| ,-- Taxus_wallichiana

| | `--| ,-- Cunninghamia_lanceolata

| | `--| ,-- Taiwania_cryptomerioides

| | `--| ,-- Athrotaxis_selaginoides

| | `--| ,-- Sequoia_sempervirens

| | `--| ,-- Cryptomeria_japonica

| | `--| ,-- Cupressus_funebris

| | `----- Callitris_muelleri

`--|	,-- Pararaucaria_patagonica

| | ,-- New_total_plant_from_Arkansas

| ,--| ,----- Alvinia_bohemica_plant

| | `--| ,-- Himeriella_muensteri

| | `--| ,-- Kachaikestrobus_acuminatus

| | `----- Tomaxellia_biforme

| | ,-- Araucaria_mirabilis

`--|	,----- Araucaria_heterophylla

| ,--| ,-- Wollemia_nobilis

| | `----- Agathis_australis

| | ,-- Lagarostrobos_franklinii

`--|	,----- Prumnopitys_taxifolia

| ,--| ,-- Lepidothamnus_laxifolius

| | `----- Phyllocladus_aspleniifolius

`--| ,-- Saxegothaea_conspicua

`--| ,-- Microcachrys_tetragona

`--| ,-- Pherosphaera_hookeriana

`--| ,-- Acmopyle_pancheri

`--| ,-- Dacrydium_cupressinum

`--| ,-- Retrophyllum_rospigliosii

`----- Podocarpus_archboldii

(ii) Majority rule tree (from 5 trees, cut 50)

,-- Ginkgo_biloba

| ,-- Cordaixylon_dumusum

|--| | ,-- Emporia_lockardii

`--100|	,--100--- Hanskerpia_hamiltonensis

| | ,-- Voltzia_hexagona

`--100|	|	,-- Nothodacrium_warrenii

| | ,--100--- Rissikia_media

`--100|	|	,-- Telemachus_aequatus

| | | ,-- Mataia_podocarpoides

`--100|	|	|	,-- Eathiestrobus_mackenziei

| | | | ,-- Pinus_contorta

| | | | |-- Picea_abies

`--100|	|	,--100|	|-- Cathaya_argyrophylla

| | | | | ,-- Pseudotsuga_menziesii

| | | | |--100--- Larix_decidua

| | | `--60| ,-- Schizolepidopsis_ediae

`--100|	|	|--100|	,-- Schizolepidopsis_daohugouensis

| | | `--100--- Schizolepidopsis_canicularis

| | | ,-- Cedrus_deodara

| | | | ,-- Keteleeria_davidiana

| | `--60| ,--100--- Abies_lasiocarpa

| | `--80| ,-- Pseudolarix_amabilis

| | `--80| ,-- Tsuga_canadensis

`--100|	`--100--- Nothotsuga_longibracteata

| ,-- Sciadopitys_verticillata

| ,--100| ,-- Taxus_wallichiana

| | `--100| ,-- Cunninghamia_lanceolata

| | `--100| ,-- Taiwania_cryptomerioides

| | `--100| ,-- Athrotaxis_selaginoides

| | `--100| ,-- Sequoia_sempervirens

| | `--100| ,-- Cryptomeria_japonica

| | `--100| ,-- Cupressus_funebr

| | `--100--- Callitris_muell

`--100|	,-- Pararaucaria_patagonica

| | ,-- New_total_plant_from_Arkansas

| ,--100| ,--100--- Alvinia_bohemica_plant

| | `--100| ,-- Himeriella_muensteri

| | `--100| ,-- Kachaikestrobus_acuminatus

| | `--100--- Tomaxellia_biforme

| | ,-- Araucaria_mirabilis

`--100|	,--100--- Araucaria_heterophylla

| ,--100| ,-- Wollemia_nobilis

| | `--100--- Agathis_australis

| | ,-- Lagarostrobos_franklinii

`--100|	,--100--- Prumnopitys_taxifolia

| ,--100| ,-- Lepidothamnus_laxifolius

| | `--100--- Phyllocladus_aspleniifolius

`--100|	,-- Saxegothaea_conspicua

`--100|	,-- Microcachrys_tetragona

`--100|	,-- Pherosphaera_hookeriana

`--100|	,-- Acmopyle_pancheri

`--100|	,-- Dacrydium_cupres

`--100|	,-- Retroph_ro

`--100--- Podoc_arch

(iii) Reduced strict consensus of 5 trees

,-- Ginkgo_biloba

| ,-- Cordaixylon_dumusum

|--| | ,-- Emporia_lockardii

| | ,----- Hanskerpia_hamiltonensis

`--| | ,-- Voltzia_hexagona

| | | ,-- Nothodacrium_warrenii

`--| | ,----- Rissikia_media

| | | ,-- Mataia_podocarpoides

`--| |	|	,-- Eathiestrobus_mackenziei

| | | |-- Pseudolarix_amabilis

| | | |-- Pinus_contorta

`--|	|	|-- Picea_abies

| | |-- Cedrus_deodara

| | |-- Cathaya_argyrophylla

| | | ,-- Tsuga_canadensis

`--a| ,--+----- Nothotsuga_longibracteata

| | | ,-- Pseudotsuga_menziesii

| | |----- Larix_decidua

| | | ,-- Keteleeria_davidiana

| | |----- Abies_lasiocarpa

| | | ,-- Schizolepidopsis_ediae

| | `--| ,-- Schizolepidopsis_daohugouensis

| | `----- Schizolepidopsis_canicularis

`--|	,-- Sciadopitys_verticillata

| ,--| ,-- Taxus_wallichiana

| | `--| ,-- Cunninghamia_lanceolata

| | `--| ,-- Taiwania_cryptomerioides

| | `--| ,-- Athrotaxis_selaginoides

| | `--| ,-- Sequoia_sempervirens

| | `--| ,-- Cryptomeria_japonica

| | `--| ,-- Cupressus_funebris

`--|	`----- Callitris_muelleri

| ,-- Araucaria_mirabilis

| ,----- Araucaria_heterophylla

| ,--| ,-- Wollemia_nobilis

| | `----- Agathis_australis

| | ,-- Lagarostrobos_franklinii

`--b|	,----- Prumnopitys_taxifolia

| ,--| ,-- Lepidothamnus_laxifolius

| | `----- Phyllocladus_aspleniifolius

`--| ,-- Saxegothaea_conspicua

`--| ,-- Microcachrys_tetragona

`--| ,-- Pherosphaera_hookeriana

`--| ,-- Acmopyle_pancheri

`--| ,-- Dacrydium_cupressinum

`--| ,-- Retrophyllum_rospigliosii

**Legends:** `----- Podocarpus_archboldii

a: Telemachus_aequatus (40)

b: node 92 of consensus

,-- Pararaucaria_patagonica

| ,-- New_total_plant_from_Arkansas

|--| ,----- Alvinia_bohemica_plant

`--| ,-- Himeriella_muensteri

`--| ,-- Kachaikestrobus_acuminatus

`----- Tomaxellia_biforme

**7) Equal Weights - character 0 standardized to 5 - yes: constrain Old conifers - no: constrain Pinaceae**

(i) Strict consensus of 14 trees

,-- Ginkgo_biloba

| ,-- Cordaixylon_dumusum

|--| | ,-- Emporia_lockardii

| | |-- Hanskerpia_hamiltonensis

`--| | ,-- Voltzia_hexagona

| | | ,-- Nothodacrium_warrenii

`--| | |-- Rissikia_media

| | | ,-- New_total_plant_from_Arkansas

| | | |-- Alvinia_bohemica_plant

| | | |-- Telemachus_aequatus

`--| | |-- Pararaucaria_patagonica

| | | ,-- Himeriella_muensteri

| | |--| ,-- Kachaikestrobus_acuminatus

| | | `----- Tomaxellia_biforme

| | | ,-- Sciadopitys_verticillata

| | |--| ,-- Taxus_wallichiana

`--| | `--| ,-- Cunninghamia_lanceolata

| | `--| ,-- Taiwania_cryptomerioides

| | `--| ,-- Athrotaxis_selaginoides

| | `--| ,-- Sequoia_sempervirens

| | `--| ,-- Cryptomeria_japonica

| | `--| ,-- Cupressus_funebris

| | `----- Callitris_muelleri

| | ,-- Araucaria_mirabilis

| | ,----- Araucaria_heterophylla

| | ,--| ,-- Wollemia_nobilis

`--| | `----- Agathis_australis

| | ,-- Lagarostrobos_franklinii

|--| ,----- Prumnopitys_taxifolia

| | ,--| ,-- Lepidothamnus_laxifolius

| | | `----- Phyllocladus_aspleniifolius

| `--| ,-- Saxegothaea_conspicua

| | | ,-- Pherosphaera_hookeriana

| `--| |-- Microcachrys_tetragona

| `--| ,-- Acmopyle_pancheri

| `--| ,-- Dacrydium_cupressinum

| `--| ,-- Retrophyllum_rospigliosii

| `----- Podocarpus_archboldii

| ,-- Eathiestrobus_mackenziei

| |-- Schizolepidopsis_ediae

| |-- Pinus_contorta

| |-- Picea_abies

| |-- Cedrus_deodara

| |-- Cathaya_argyrophylla

| | ,-- Schizolepidopsis_daohugouensis

`--+----- Schizolepidopsis_canicularis

| ,-- Pseudotsuga_menziesii

|----- Larix_decidua

| ,-- Keteleeria_davidiana

| ,----- Abies_lasiocarpa

| | ,-- Mataia_podocarpoides

`--| ,----- Pseudolarix_amabilis

`--| ,-- Tsuga_canadensis

`----- Nothotsuga_longibracteata

(ii) Majority rule tree (from 14 trees, cut 50)

,-- Ginkgo_biloba

| ,-- Cordaixylon_dumusum

|--| | ,-- Emporia_lockardii

| | ,--71--- Hanskerpia_hamiltonensis

`--100|	|	,-- Voltzia_hexagona

| | | ,-- Nothodacrium_warrenii

`--100|	|	,--85--- Rissikia_media

| | | ,-- Telemachus_aequatus

`--100|	|	|	,-- Schizolepidopsis_ediae

| | | | ,-- Schizolepidopsis_daohugouensis

| | | |--100--- Schizolepidopsis_canicularis

`--100|	|	|	,-- Cedrus_deodara

| | ,--100| | ,-- Keteleeria_davidiana

| | | | | ,--100--- Abies_lasiocarpa

| | | | | | ,-- Mataia_podocarpoides

`--100|	|	`--57|	,--100|	|-- Pseudolarix_amabilis

| | | | `--100| ,-- Tsuga_canadensis

| | | | `--100--- Nothotsuga_longibracteata

| | `--57| ,-- Pseudotsuga_menziesii

| | | ,--100--- Larix_decidua

| | `--57| ,-- Cathaya_argyrophylla

| | `--57| ,-- Eathiestrobus_mackenziei

`--57|	`--57+-- Pinus_contorta

| `-- Picea_abies

| ,-- Pararaucaria_patagonica

| | ,-- New_total_plant_from_Arkansas

| ,--57| ,--71--- Alvinia_bohemica_plant

| | `--57| ,-- Himeriella_muensteri

| | `--100| ,-- Kachaikestrobus_acuminatus

| | `--100--- Tomaxellia_biforme

| | ,-- Sciadopitys_verticillata

| | ,--100| ,-- Taxus_wallichiana

`--57|	|	`--100|	,-- Cunninghamia_lanceolata

| | `--100| ,-- Taiwania_cryptomerioides

| | `--100| ,-- Athrotaxis_selaginoides

| | `--100| ,-- Sequoia_sempervirens

| | `--100| ,-- Cryptomeria_japonica

| | `--100| ,-- Cupressus_funebris

`--85|	`--100--- Callitris_muelleri

| ,-- Araucaria_mirabilis

| ,--100--- Araucaria_heterophylla

| ,--100| ,-- Wollemia_nobilis

| | `--100--- Agathis_australis

| | ,-- Lagarostrobos_franklinii

`--100|	,--100--- Prumnopitys_taxifolia

| ,--100| ,-- Lepidothamnus_laxifolius

| | `--100--- Phyllocladus_aspleniifolius

`--100|	,-- Saxegothaea_conspicua

`--100|	,-- Microcachrys_tetragona

`--100|	,-- Pherosphaera_hookeriana

`--78|	,-- Acmopyle_pancheri

`--100|	,-- Dacrydium_cupressinum

`--100|	,-- Retrophyllum_rospig

`--100--- Podocarpus_archbold

(iii) Reduced strict consensus of 14 trees

,-- Ginkgo_biloba

| ,-- Cordaixylon_dumusum

|--| | ,-- Emporia_lockardii

| | |-- Hanskerpia_hamiltonensis

`--| | ,-- Voltzia_hexagona

| | | ,-- Nothodacrium_warrenii

`--| | |-- Rissikia_media

| | | ,-- Eathiestrobus_mackenziei

| | | |-- Schizolepidopsis_ediae

`--| |	|-- Pinus_contorta

| | |-- Picea_abies

| | |-- Cedrus_deodara

| | |-- Cathaya_argyrophylla

| | | ,-- Schizolepidopsis_daohugouensis

`--|	,--+----- Schizolepidopsis_canicularis

| | | ,-- Pseudotsuga_menziesii

| | |----- Larix_decidua

| | | ,-- Keteleeria_davidiana

| | | ,----- Abies_lasiocarpa

| | | | ,-- Mataia_podocarpoides

| | `--| |-- Pseudolarix_amabilis

| | `--| ,-- Tsuga_canadensis

| | `----- Nothotsuga_longibracteata

`--abcde|	,-- Sciadopitys_verticillata

| ,--| ,-- Taxus_wallichiana

| | `--| ,-- Cunninghamia_lanceolata

| | `--| ,-- Taiwania_cryptomerioides

| | `--| ,-- Athrotaxis_selaginoides

| | `--| ,-- Sequoia_sempervirens

| | `--| ,-- Cryptomeria_japonica

| | `--| ,-- Cupressus_funebris

`--acde|	`----- Callitris_muelleri

| ,-- Araucaria_mirabilis

| ,----- Araucaria_heterophylla

| ,--| ,-- Wollemia_nobilis

| | `----- Agathis_australis

| | ,-- Lagarostrobos_franklinii

`--acde|	,----- Prumnopitys_taxifolia

| ,--| ,-- Lepidothamnus_laxifolius

| | `----- Phyllocladus_aspleniifolius

`--| ,-- Saxegothaea_conspicua

| | ,-- Pherosphaera_hookeriana

`--| |-- Microcachrys_tetragona

`--| ,-- Acmopyle_pancheri

`--| ,-- Dacrydium_cupressinum

`--| ,-- Retrophyllum_rospigliosii

**Legends:** `----- Podocarpus_archboldii

a: Pararaucaria_patagonica (36)

b: Telemachus_aequatus (40)

c: Alvinia_bohemica_plant (52)

d: New_total_plant_from_Arkansas (53)

e: node 90 of consensus

,-- Himeriella_muensteri

|--| ,-- Kachaikestrobus_acuminatus

`----- Tomaxellia_biforme

**8) Equal Weights - character 0 standardized to 5 - yes: constrain Old conifers - yes: constrain Pinaceae**

(i) Strict consensus of 5 trees

,-- Ginkgo_biloba

| ,-- Cordaixylon_dumusum

|--| | ,-- Emporia_lockardii

`--| ,----- Hanskerpia_hamiltonensis

| | ,-- Voltzia_hexagona

`--| |	,-- Nothodacrium_warrenii

| | ,----- Rissikia_media

`--| | ,-- Telemachus_aequatus

| | | ,-- Mataia_podocarpoides

`--| | |	,-- Eathiestrobus_mackenziei

| | | |-- Pseudolarix_amabilis

| | | |-- Pinus_contorta

`--| |	|-- Picea_abies

| | |-- Cedrus_deodara

| | |-- Cathaya_argyrophylla

| | | ,-- Tsuga_canadensis

| | ,--+----- Nothotsuga_longibracteata

`--| | | ,-- Pseudotsuga_menziesii

| | |----- Larix_decidua

| | | ,-- Keteleeria_davidiana

| | |----- Abies_lasiocarpa

| | | ,-- Schizolepidopsis_ediae

| | `--| ,-- Schizolepidopsis_daohugouensis

| | `----- Schizolepidopsis_canicularis

| | ,-- Sciadopitys_verticillata

`--| ,--| ,-- Taxus_wallichiana

| | `--| ,-- Cunninghamia_lanceolata

| | `--| ,-- Taiwania_cryptomerioides

| | `--| ,-- Athrotaxis_selaginoides

| | `--| ,-- Sequoia_sempervirens

| | `--| ,-- Cryptomeria_japonica

| | `--| ,-- Cupressus_funebris

| | `----- Callitris_muelleri

`--|	,-- Pararaucaria_patagonica

| | ,-- New_total_plant_from_Arkansas

| ,--| ,----- Alvinia_bohemica_plant

| | `--| ,-- Himeriella_muensteri

| | `--| ,-- Kachaikestrobus_acuminatus

| | `----- Tomaxellia_biforme

| | ,-- Araucaria_mirabilis

`--|	,----- Araucaria_heterophylla

| ,--| ,-- Wollemia_nobilis

| | `----- Agathis_australis

| | ,-- Lagarostrobos_franklinii

`--|	,----- Prumnopitys_taxifolia

| ,--| ,-- Lepidothamnus_laxifolius

| | `----- Phyllocladus_aspleniifolius

`--| ,-- Saxegothaea_conspicua

`--| ,-- Microcachrys_tetragona

`--| ,-- Pherosphaera_hookeriana

`--| ,-- Acmopyle_pancheri

`--| ,-- Dacrydium_cupressinum

`--| ,-- Retrophyllum_rospigliosii

`----- Podocarpus_archboldii

(ii) Majority rule tree (from 5 trees, cut 50)

,-- Ginkgo_biloba

| ,-- Cordaixylon_dumusum

|--| | ,-- Emporia_lockardii

`--100|	,--100--- Hanskerpia_hamiltonensis

| | ,-- Voltzia_hexagona

`--100|	|	,-- Nothodacrium_warrenii

| | ,--100--- Rissikia_media

`--100|	|	,-- Telemachus_aequatus

| | | ,-- Mataia_podocarpoides

`--100|	|	|	,-- Eathiestrobus_mackenziei

| | | | ,-- Pinus_contorta

| | | | |-- Picea_abies

`--100|	|	,--100|	|-- Cathaya_argyrophylla

| | | | | ,-- Pseudotsuga_menziesii

| | | | |--100--- Larix_decidua

| | | `--60| ,-- Schizolepidopsis_ediae

`--100|	|	|--100|	,-- Schizolepidopsis_daohugouensis

| | | `--100--- Schizolepidopsis_canicularis

| | | ,-- Cedrus_deodara

| | | | ,-- Keteleeria_davidiana

| | `--60| ,--100--- Abies_lasiocarpa

| | `--80| ,-- Pseudolarix_amabilis

| | `--80| ,-- Tsuga_canadensis

`--100|	`--100--- Nothotsuga_longibracteata

| ,-- Sciadopitys_verticillata

| ,--100| ,-- Taxus_wallichiana

| | `--100| ,-- Cunninghamia_lanceolata

| | `--100| ,-- Taiwania_cryptomerioides

| | `--100| ,-- Athrotaxis_selaginoides

| | `--100| ,-- Sequoia_sempervirens

| | `--100| ,-- Cryptomeria_japonica

| | `--100| ,-- Cupressus_funebr

| | `--100--- Callitris_muell

`--100|	,-- Pararaucaria_patagonica

| | ,-- New_total_plant_from_Arkansas

| ,--100| ,--100--- Alvinia_bohemica_plant

| | `--100| ,-- Himeriella_muensteri

| | `--100| ,-- Kachaikestrobus_acuminatus

| | `--100--- Tomaxellia_biforme

| | ,-- Araucaria_mirabilis

`--100|	,--100--- Araucaria_heterophylla

| ,--100| ,-- Wollemia_nobilis

| | `--100--- Agathis_australis

| | ,-- Lagarostrobos_franklinii

`--100|	,--100--- Prumnopitys_taxifolia

| ,--100| ,-- Lepidothamnus_laxifolius

| | `--100--- Phyllocladus_aspleniifolius

`--100|	,-- Saxegothaea_conspicua

`--100|	,-- Microcachrys_tetragona

`--100|	,-- Pherosphaera_hookeriana

`--100|	,-- Acmopyle_pancheri

`--100|	,-- Dacrydium_cupres

`--100|	,-- Retroph_ro

`--100--- Podoc_arch

(iii) Reduced strict consensus of 5 trees

,-- Ginkgo_biloba

| ,-- Cordaixylon_dumusum

|--| | ,-- Emporia_lockardii

| | ,----- Hanskerpia_hamiltonensis

`--| | ,-- Voltzia_hexagona

| | | ,-- Nothodacrium_warrenii

`--| | ,----- Rissikia_media

| | | ,-- Mataia_podocarpoides

`--| |	|	,-- Eathiestrobus_mackenziei

| | | |-- Pseudolarix_amabilis

| | | |-- Pinus_contorta

`--|	|	|-- Picea_abies

| | |-- Cedrus_deodara

| | |-- Cathaya_argyrophylla

| | | ,-- Tsuga_canadensis

`--a| ,--+----- Nothotsuga_longibracteata

| | | ,-- Pseudotsuga_menziesii

| | |----- Larix_decidua

| | | ,-- Keteleeria_davidiana

| | |----- Abies_lasiocarpa

| | | ,-- Schizolepidopsis_ediae

| | `--| ,-- Schizolepidopsis_daohugouensis

| | `----- Schizolepidopsis_canicularis

`--|	,-- Sciadopitys_verticillata

| ,--| ,-- Taxus_wallichiana

| | `--| ,-- Cunninghamia_lanceolata

| | `--| ,-- Taiwania_cryptomerioides

| | `--| ,-- Athrotaxis_selaginoides

| | `--| ,-- Sequoia_sempervirens

| | `--| ,-- Cryptomeria_japonica

| | `--| ,-- Cupressus_funebris

`--|	`----- Callitris_muelleri

| ,-- Araucaria_mirabilis

| ,----- Araucaria_heterophylla

| ,--| ,-- Wollemia_nobilis

| | `----- Agathis_australis

| | ,-- Lagarostrobos_franklinii

`--b|	,----- Prumnopitys_taxifolia

| ,--| ,-- Lepidothamnus_laxifolius

| | `----- Phyllocladus_aspleniifolius

`--| ,-- Saxegothaea_conspicua

`--| ,-- Microcachrys_tetragona

`--| ,-- Pherosphaera_hookeriana

`--| ,-- Acmopyle_pancheri

`--| ,-- Dacrydium_cupressinum

`--| ,-- Retrophyllum_rospigliosii

**Legends:** `----- Podocarpus_archboldii

a: Telemachus_aequatus (40)

b: node 92 of consensus

,-- Pararaucaria_patagonica

| ,-- New_total_plant_from_Arkansas

|--| ,----- Alvinia_bohemica_plant

`--| ,-- Himeriella_muensteri

`--| ,-- Kachaikestrobus_acuminatus

`----- Tomaxellia_biforme

**9) Equal Weights - character 1 standardized to 1 - yes: constrain Old conifers - no: constrain Pinaceae**

(i) Strict consensus of 14 trees

,-- Ginkgo_biloba

| ,-- Cordaixylon_dumusum

|--| | ,-- Emporia_lockardii

| | |-- Hanskerpia_hamiltonensis

`--| | ,-- Voltzia_hexagona

| | | ,-- Nothodacrium_warrenii

`--| | |-- Rissikia_media

| | | ,-- New_total_plant_from_Arkansas

| | | |-- Alvinia_bohemica_plant

| | | |-- Telemachus_aequatus

`--| | |-- Pararaucaria_patagonica

| | | ,-- Himeriella_muensteri

| | |--| ,-- Kachaikestrobus_acuminatus

| | | `----- Tomaxellia_biforme

| | | ,-- Sciadopitys_verticillata

| | |--| ,-- Taxus_wallichiana

`--| | `--| ,-- Cunninghamia_lanceolata

| | `--| ,-- Taiwania_cryptomerioides

| | `--| ,-- Athrotaxis_selaginoides

| | `--| ,-- Sequoia_sempervirens

| | `--| ,-- Cryptomeria_japonica

| | `--| ,-- Cupressus_funebris

| | `----- Callitris_muelleri

| | ,-- Araucaria_mirabilis

| | ,----- Araucaria_heterophylla

| | ,--| ,-- Wollemia_nobilis

`--| | `----- Agathis_australis

| | ,-- Lagarostrobos_franklinii

|--| ,----- Prumnopitys_taxifolia

| | ,--| ,-- Lepidothamnus_laxifolius

| | | `----- Phyllocladus_aspleniifolius

| `--| ,-- Saxegothaea_conspicua

| | | ,-- Pherosphaera_hookeriana

| `--| |-- Microcachrys_tetragona

| `--| ,-- Acmopyle_pancheri

| `--| ,-- Dacrydium_cupressinum

| `--| ,-- Retrophyllum_rospigliosii

| `----- Podocarpus_archboldii

| ,-- Eathiestrobus_mackenziei

| |-- Schizolepidopsis_ediae

| |-- Pinus_contorta

| |-- Picea_abies

| |-- Cedrus_deodara

| |-- Cathaya_argyrophylla

| | ,-- Schizolepidopsis_daohugouensis

`--+----- Schizolepidopsis_canicularis

| ,-- Pseudotsuga_menziesii

|----- Larix_decidua

| ,-- Keteleeria_davidiana

| ,----- Abies_lasiocarpa

| | ,-- Mataia_podocarpoides

`--| ,----- Pseudolarix_amabilis

`--| ,-- Tsuga_canadensis

`----- Nothotsuga_longibracteata

(ii) Majority rule tree (from 14 trees, cut 50)

,-- Ginkgo_biloba

| ,-- Cordaixylon_dumusum

|--| | ,-- Emporia_lockardii

| | ,--71--- Hanskerpia_hamiltonensis

`--100|	|	,-- Voltzia_hexagona

| | | ,-- Nothodacrium_warrenii

`--100|	|	,--85--- Rissikia_media

| | | ,-- Telemachus_aequatus

`--100|	|	|	,-- Schizolepidopsis_ediae

| | | | ,-- Schizolepidopsis_daohugouensis

| | | |--100--- Schizolepidopsis_canicularis

`--100|	|	|	,-- Cedrus_deodara

| | ,--100| | ,-- Keteleeria_davidiana

| | | | | ,--100--- Abies_lasiocarpa

| | | | | | ,-- Mataia_podocarpoides

`--100|	|	`--57|	,--100|	|-- Pseudolarix_amabilis

| | | | `--100| ,-- Tsuga_canadensis

| | | | `--100--- Nothotsuga_longibracteata

| | `--57| ,-- Pseudotsuga_menziesii

| | | ,--100--- Larix_decidua

| | `--57| ,-- Cathaya_argyrophylla

| | `--57| ,-- Eathiestrobus_mackenziei

`--57|	`--57+-- Pinus_contorta

| `-- Picea_abies

| ,-- Pararaucaria_patagonica

| | ,-- New_total_plant_from_Arkansas

| ,--57| ,--71--- Alvinia_bohemica_plant

| | `--57| ,-- Himeriella_muensteri

| | `--100| ,-- Kachaikestrobus_acuminatus

| | `--100--- Tomaxellia_biforme

| | ,-- Sciadopitys_verticillata

| | ,--100| ,-- Taxus_wallichiana

`--57|	|	`--100|	,-- Cunninghamia_lanceolata

| | `--100| ,-- Taiwania_cryptomerioides

| | `--100| ,-- Athrotaxis_selaginoides

| | `--100| ,-- Sequoia_sempervirens

| | `--100| ,-- Cryptomeria_japonica

| | `--100| ,-- Cupressus_funebris

`--85|	`--100--- Callitris_muelleri

| ,-- Araucaria_mirabilis

| ,--100--- Araucaria_heterophylla

| ,--100| ,-- Wollemia_nobilis

| | `--100--- Agathis_australis

| | ,-- Lagarostrobos_franklinii

`--100|	,--100--- Prumnopitys_taxifolia

| ,--100| ,-- Lepidothamnus_laxifolius

| | `--100--- Phyllocladus_aspleniifolius

`--100|	,-- Saxegothaea_conspicua

`--100|	,-- Microcachrys_tetragona

`--100|	,-- Pherosphaera_hookeriana

`--78|	,-- Acmopyle_pancheri

`--100|	,-- Dacrydium_cupressinum

`--100|	,-- Retrophyllum_rospig

`--100--- Podocarpus_archbold

(iii) Reduced strict consensus of 14 trees

,-- Ginkgo_biloba

| ,-- Cordaixylon_dumusum

|--| | ,-- Emporia_lockardii

| | |-- Hanskerpia_hamiltonensis

`--| | ,-- Voltzia_hexagona

| | | ,-- Nothodacrium_warrenii

`--| | |-- Rissikia_media

| | | ,-- Eathiestrobus_mackenziei

| | | |-- Schizolepidopsis_ediae

`--| |	|-- Pinus_contorta

| | |-- Picea_abies

| | |-- Cedrus_deodara

| | |-- Cathaya_argyrophylla

| | | ,-- Schizolepidopsis_daohugouensis

`--|	,--+----- Schizolepidopsis_canicularis

| | | ,-- Pseudotsuga_menziesii

| | |----- Larix_decidua

| | | ,-- Keteleeria_davidiana

| | | ,----- Abies_lasiocarpa

| | | | ,-- Mataia_podocarpoides

| | `--| |-- Pseudolarix_amabilis

| | `--| ,-- Tsuga_canadensis

| | `----- Nothotsuga_longibracteata

`--abcde|	,-- Sciadopitys_verticillata

| ,--| ,-- Taxus_wallichiana

| | `--| ,-- Cunninghamia_lanceolata

| | `--| ,-- Taiwania_cryptomerioides

| | `--| ,-- Athrotaxis_selaginoides

| | `--| ,-- Sequoia_sempervirens

| | `--| ,-- Cryptomeria_japonica

| | `--| ,-- Cupressus_funebris

`--acde|	`----- Callitris_muelleri

| ,-- Araucaria_mirabilis

| ,----- Araucaria_heterophylla

| ,--| ,-- Wollemia_nobilis

| | `----- Agathis_australis

| | ,-- Lagarostrobos_franklinii

`--acde|	,----- Prumnopitys_taxifolia

| ,--| ,-- Lepidothamnus_laxifolius

| | `----- Phyllocladus_aspleniifolius

`--| ,-- Saxegothaea_conspicua

| | ,-- Pherosphaera_hookeriana

`--| |-- Microcachrys_tetragona

`--| ,-- Acmopyle_pancheri

`--| ,-- Dacrydium_cupressinum

`--| ,-- Retrophyllum_rospigliosii

**Legends:** `----- Podocarpus_archboldii

a: Pararaucaria_patagonica (36)

b: Telemachus_aequatus (40)

c: Alvinia_bohemica_plant (52)

d: New_total_plant_from_Arkansas (53)

e: node 90 of consensus

,-- Himeriella_muensteri

|--| ,-- Kachaikestrobus_acuminatus

`----- Tomaxellia_biforme

**10) Equal Weights - character 1 standardized to 1 - yes: constrain Old conifers - yes: constrain Pinaceae**

(i) Strict consensus of 5 trees

,-- Ginkgo_biloba

| ,-- Cordaixylon_dumusum

|--| | ,-- Emporia_lockardii

`--| ,----- Hanskerpia_hamiltonensis

| | ,-- Voltzia_hexagona

`--| |	,-- Nothodacrium_warrenii

| | ,----- Rissikia_media

`--| | ,-- Telemachus_aequatus

| | | ,-- Mataia_podocarpoides

`--| | |	,-- Eathiestrobus_mackenziei

| | | |-- Pseudolarix_amabilis

| | | |-- Pinus_contorta

`--| |	|-- Picea_abies

| | |-- Cedrus_deodara

| | |-- Cathaya_argyrophylla

| | | ,-- Tsuga_canadensis

| | ,--+----- Nothotsuga_longibracteata

`--| | | ,-- Pseudotsuga_menziesii

| | |----- Larix_decidua

| | | ,-- Keteleeria_davidiana

| | |----- Abies_lasiocarpa

| | | ,-- Schizolepidopsis_ediae

| | `--| ,-- Schizolepidopsis_daohugouensis

| | `----- Schizolepidopsis_canicularis

| | ,-- Sciadopitys_verticillata

`--| ,--| ,-- Taxus_wallichiana

| | `--| ,-- Cunninghamia_lanceolata

| | `--| ,-- Taiwania_cryptomerioides

| | `--| ,-- Athrotaxis_selaginoides

| | `--| ,-- Sequoia_sempervirens

| | `--| ,-- Cryptomeria_japonica

| | `--| ,-- Cupressus_funebris

| | `----- Callitris_muelleri

`--|	,-- Pararaucaria_patagonica

| | ,-- New_total_plant_from_Arkansas

| ,--| ,----- Alvinia_bohemica_plant

| | `--| ,-- Himeriella_muensteri

| | `--| ,-- Kachaikestrobus_acuminatus

| | `----- Tomaxellia_biforme

| | ,-- Araucaria_mirabilis

`--|	,----- Araucaria_heterophylla

| ,--| ,-- Wollemia_nobilis

| | `----- Agathis_australis

| | ,-- Lagarostrobos_franklinii

`--|	,----- Prumnopitys_taxifolia

| ,--| ,-- Lepidothamnus_laxifolius

| | `----- Phyllocladus_aspleniifolius

`--| ,-- Saxegothaea_conspicua

`--| ,-- Microcachrys_tetragona

`--| ,-- Pherosphaera_hookeriana

`--| ,-- Acmopyle_pancheri

`--| ,-- Dacrydium_cupressinum

`--| ,-- Retrophyllum_rospigliosii

`----- Podocarpus_archboldii

(ii) Majority rule tree (from 5 trees, cut 50)

,-- Ginkgo_biloba

| ,-- Cordaixylon_dumusum

|--| | ,-- Emporia_lockardii

`--100|	,--100--- Hanskerpia_hamiltonensis

| | ,-- Voltzia_hexagona

`--100|	|	,-- Nothodacrium_warrenii

| | ,--100--- Rissikia_media

`--100|	|	,-- Telemachus_aequatus

| | | ,-- Mataia_podocarpoides

`--100|	|	|	,-- Eathiestrobus_mackenziei

| | | | ,-- Pinus_contorta

| | | | |-- Picea_abies

`--100|	|	,--100|	|-- Cathaya_argyrophylla

| | | | | ,-- Pseudotsuga_menziesii

| | | | |--100--- Larix_decidua

| | | `--60| ,-- Schizolepidopsis_ediae

`--100|	|	|--100|	,-- Schizolepidopsis_daohugouensis

| | | `--100--- Schizolepidopsis_canicularis

| | | ,-- Cedrus_deodara

| | | | ,-- Keteleeria_davidiana

| | `--60| ,--100--- Abies_lasiocarpa

| | `--80| ,-- Pseudolarix_amabilis

| | `--80| ,-- Tsuga_canadensis

`--100|	`--100--- Nothotsuga_longibracteata

| ,-- Sciadopitys_verticillata

| ,--100| ,-- Taxus_wallichiana

| | `--100| ,-- Cunninghamia_lanceolata

| | `--100| ,-- Taiwania_cryptomerioides

| | `--100| ,-- Athrotaxis_selaginoides

| | `--100| ,-- Sequoia_sempervirens

| | `--100| ,-- Cryptomeria_japonica

| | `--100| ,-- Cupressus_funebr

| | `--100--- Callitris_muelle

`--100|	,-- Pararaucaria_patagonica

| | ,-- New_total_plant_from_Arkansas

| ,--100| ,--100--- Alvinia_bohemica_plant

| | `--100| ,-- Himeriella_muensteri

| | `--100| ,-- Kachaikestrobus_acuminatus

| | `--100--- Tomaxellia_biforme

| | ,-- Araucaria_mirabilis

`--100|	,--100--- Araucaria_heterophylla

| ,--100| ,-- Wollemia_nobilis

| | `--100--- Agathis_australis

| | ,-- Lagarostrobos_franklinii

`--100|	,--100--- Prumnopitys_taxifolia

| ,--100| ,-- Lepidothamnus_laxifolius

| | `--100--- Phyllocladus_aspleniifolius

`--100|	,-- Saxegothaea_conspicua

`--100|	,-- Microcachrys_tetragona

`--100|	,-- Pherosphaera_hookeriana

`--100|	,-- Acmopyle_pancheri

`--100|	,-- Dacrydium_cupres

`--100|	,-- Retroph_ro

`--100--- Podoc_arch

(iii) Reduced strict consensus of 5 trees

,-- Ginkgo_biloba

| ,-- Cordaixylon_dumusum

|--| | ,-- Emporia_lockardii

| | ,----- Hanskerpia_hamiltonensis

`--| | ,-- Voltzia_hexagona

| | | ,-- Nothodacrium_warrenii

`--| | ,----- Rissikia_media

| | | ,-- Mataia_podocarpoides

`--| |	|	,-- Eathiestrobus_mackenziei

| | | |-- Pseudolarix_amabilis

| | | |-- Pinus_contorta

`--|	|	|-- Picea_abies

| | |-- Cedrus_deodara

| | |-- Cathaya_argyrophylla

| | | ,-- Tsuga_canadensis

`--a| ,--+----- Nothotsuga_longibracteata

| | | ,-- Pseudotsuga_menziesii

| | |----- Larix_decidua

| | | ,-- Keteleeria_davidiana

| | |----- Abies_lasiocarpa

| | | ,-- Schizolepidopsis_ediae

| | `--| ,-- Schizolepidopsis_daohugouensis

| | `----- Schizolepidopsis_canicularis

`--|	,-- Sciadopitys_verticillata

| ,--| ,-- Taxus_wallichiana

| | `--| ,-- Cunninghamia_lanceolata

| | `--| ,-- Taiwania_cryptomerioides

| | `--| ,-- Athrotaxis_selaginoides

| | `--| ,-- Sequoia_sempervirens

| | `--| ,-- Cryptomeria_japonica

| | `--| ,-- Cupressus_funebris

`--|	`----- Callitris_muelleri

| ,-- Araucaria_mirabilis

| ,----- Araucaria_heterophylla

| ,--| ,-- Wollemia_nobilis

| | `----- Agathis_australis

| | ,-- Lagarostrobos_franklinii

`--b|	,----- Prumnopitys_taxifolia

| ,--| ,-- Lepidothamnus_laxifolius

| | `----- Phyllocladus_aspleniifolius

`--| ,-- Saxegothaea_conspicua

`--| ,-- Microcachrys_tetragona

`--| ,-- Pherosphaera_hookeriana

`--| ,-- Acmopyle_pancheri

`--| ,-- Dacrydium_cupressinum

`--| ,-- Retrophyllum_rospigliosii

**Legends:** `----- Podocarpus_archboldii

a: Telemachus_aequatus (40)

b: node 92 of consensus

,-- Pararaucaria_patagonica

| ,-- New_total_plant_from_Arkansas

|--| ,----- Alvinia_bohemica_plant

`--| ,-- Himeriella_muensteri

`--| ,-- Kachaikestrobus_acuminatus

`----- Tomaxellia_biforme

**11) Equal Weights - character 1 standardized to 5 - yes: constrain Old conifers - no: constrain Pinaceae**

(i) Strict consensus of 14 trees

,-- Ginkgo_biloba

| ,-- Cordaixylon_dumusum

|--| | ,-- Emporia_lockardii

| | |-- Hanskerpia_hamiltonensis

`--| | ,-- Voltzia_hexagona

| | | ,-- Nothodacrium_warrenii

`--| | |-- Rissikia_media

| | | ,-- New_total_plant_from_Arkansas

| | | |-- Alvinia_bohemica_plant

| | | |-- Telemachus_aequatus

`--| | |-- Pararaucaria_patagonica

| | | ,-- Himeriella_muensteri

| | |--| ,-- Kachaikestrobus_acuminatus

| | | `----- Tomaxellia_biforme

| | | ,-- Sciadopitys_verticillata

| | |--| ,-- Taxus_wallichiana

`--| | `--| ,-- Cunninghamia_lanceolata

| | `--| ,-- Taiwania_cryptomerioides

| | `--| ,-- Athrotaxis_selaginoides

| | `--| ,-- Sequoia_sempervirens

| | `--| ,-- Cryptomeria_japonica

| | `--| ,-- Cupressus_funebris

| | `----- Callitris_muelleri

| | ,-- Araucaria_mirabilis

| | ,----- Araucaria_heterophylla

| | ,--| ,-- Wollemia_nobilis

`--| | `----- Agathis_australis

| | ,-- Lagarostrobos_franklinii

|--| ,----- Prumnopitys_taxifolia

| | ,--| ,-- Lepidothamnus_laxifolius

| | | `----- Phyllocladus_aspleniifolius

| `--| ,-- Saxegothaea_conspicua

| | | ,-- Pherosphaera_hookeriana

| `--| |-- Microcachrys_tetragona

| `--| ,-- Acmopyle_pancheri

| `--| ,-- Dacrydium_cupressinum

| `--| ,-- Retrophyllum_rospigliosii

| `----- Podocarpus_archboldii

| ,-- Eathiestrobus_mackenziei

| |-- Schizolepidopsis_ediae

| |-- Pinus_contorta

| |-- Picea_abies

| |-- Cedrus_deodara

| |-- Cathaya_argyrophylla

| | ,-- Schizolepidopsis_daohugouensis

`--+----- Schizolepidopsis_canicularis

| ,-- Pseudotsuga_menziesii

|----- Larix_decidua

| ,-- Keteleeria_davidiana

| ,----- Abies_lasiocarpa

| | ,-- Mataia_podocarpoides

`--| ,----- Pseudolarix_amabilis

`--| ,-- Tsuga_canadensis

`----- Nothotsuga_longibracteata

(ii) Majority rule tree (from 14 trees, cut 50)

,-- Ginkgo_biloba

| ,-- Cordaixylon_dumusum

|--| | ,-- Emporia_lockardii

| | ,--71--- Hanskerpia_hamiltonensis

`--100|	|	,-- Voltzia_hexagona

| | | ,-- Nothodacrium_warrenii

`--100|	|	,--85--- Rissikia_media

| | | ,-- Telemachus_aequatus

`--100|	|	|	,-- Schizolepidopsis_ediae

| | | | ,-- Schizolepidopsis_daohugouensis

| | | |--100--- Schizolepidopsis_canicularis

`--100|	|	|	,-- Cedrus_deodara

| | ,--100| | ,-- Keteleeria_davidiana

| | | | | ,--100--- Abies_lasiocarpa

| | | | | | ,-- Mataia_podocarpoides

`--100|	|	`--57|	,--100|	|-- Pseudolarix_amabilis

| | | | `--100| ,-- Tsuga_canadensis

| | | | `--100--- Nothotsuga_longibracteata

| | `--57| ,-- Pseudotsuga_menziesii

| | | ,--100--- Larix_decidua

| | `--57| ,-- Cathaya_argyrophylla

| | `--57| ,-- Eathiestrobus_mackenziei

`--57|	`--57+-- Pinus_contorta

| `-- Picea_abies

| ,-- Pararaucaria_patagonica

| | ,-- New_total_plant_from_Arkansas

| ,--57| ,--71--- Alvinia_bohemica_plant

| | `--57| ,-- Himeriella_muensteri

| | `--100| ,-- Kachaikestrobus_acuminatus

| | `--100--- Tomaxellia_biforme

| | ,-- Sciadopitys_verticillata

| | ,--100| ,-- Taxus_wallichiana

`--57|	|	`--100|	,-- Cunninghamia_lanceolata

| | `--100| ,-- Taiwania_cryptomerioides

| | `--100| ,-- Athrotaxis_selaginoides

| | `--100| ,-- Sequoia_sempervirens

| | `--100| ,-- Cryptomeria_japonica

| | `--100| ,-- Cupressus_funebris

`--85|	`--100--- Callitris_muelleri

| ,-- Araucaria_mirabilis

| ,--100--- Araucaria_heterophylla

| ,--100| ,-- Wollemia_nobilis

| | `--100--- Agathis_australis

| | ,-- Lagarostrobos_franklinii

`--100|	,--100--- Prumnopitys_taxifolia

| ,--100| ,-- Lepidothamnus_laxifolius

| | `--100--- Phyllocladus_aspleniifolius

`--100|	,-- Saxegothaea_conspicua

`--100|	,-- Microcachrys_tetragona

`--100|	,-- Pherosphaera_hookeriana

`--78|	,-- Acmopyle_pancheri

`--100|	,-- Dacrydium_cupressinum

`--100|	,-- Retrophyllum_rospigl

`--100--- Podocarpus_archbold

(iii) Reduced strict consensus of 14 trees

,-- Ginkgo_biloba

| ,-- Cordaixylon_dumusum

|--| | ,-- Emporia_lockardii

| | |-- Hanskerpia_hamiltonensis

`--| | ,-- Voltzia_hexagona

| | | ,-- Nothodacrium_warrenii

`--| | |-- Rissikia_media

| | | ,-- Eathiestrobus_mackenziei

| | | |-- Schizolepidopsis_ediae

`--| |	|-- Pinus_contorta

| | |-- Picea_abies

| | |-- Cedrus_deodara

| | |-- Cathaya_argyrophylla

| | | ,-- Schizolepidopsis_daohugouensis

`--|	,--+----- Schizolepidopsis_canicularis

| | | ,-- Pseudotsuga_menziesii

| | |----- Larix_decidua

| | | ,-- Keteleeria_davidiana

| | | ,----- Abies_lasiocarpa

| | | | ,-- Mataia_podocarpoides

| | `--| |-- Pseudolarix_amabilis

| | `--| ,-- Tsuga_canadensis

| | `----- Nothotsuga_longibracteata

`--abcde|	,-- Sciadopitys_verticillata

| ,--| ,-- Taxus_wallichiana

| | `--| ,-- Cunninghamia_lanceolata

| | `--| ,-- Taiwania_cryptomerioides

| | `--| ,-- Athrotaxis_selaginoides

| | `--| ,-- Sequoia_sempervirens

| | `--| ,-- Cryptomeria_japonica

| | `--| ,-- Cupressus_funebris

`--acde|	`----- Callitris_muelleri

| ,-- Araucaria_mirabilis

| ,----- Araucaria_heterophylla

| ,--| ,-- Wollemia_nobilis

| | `----- Agathis_australis

| | ,-- Lagarostrobos_franklinii

`--acde|	,----- Prumnopitys_taxifolia

| ,--| ,-- Lepidothamnus_laxifolius

| | `----- Phyllocladus_aspleniifolius

`--| ,-- Saxegothaea_conspicua

| | ,-- Pherosphaera_hookeriana

`--| |-- Microcachrys_tetragona

`--| ,-- Acmopyle_pancheri

`--| ,-- Dacrydium_cupressinum

`--| ,-- Retrophyllum_rospigliosii

**Legends:** `----- Podocarpus_archboldii

a: Pararaucaria_patagonica (36)

b: Telemachus_aequatus (40)

c: Alvinia_bohemica_plant (52)

d: New_total_plant_from_Arkansas (53)

e: node 90 of consensus

,-- Himeriella_muensteri

|--| ,-- Kachaikestrobus_acuminatus

`----- Tomaxellia_biforme

**12) Equal Weights - character 1 standardized to 5 - yes: constrain Old conifers - yes: constrain Pinaceae**

(i) Strict consensus of 5 trees

,-- Ginkgo_biloba

| ,-- Cordaixylon_dumusum

|--| | ,-- Emporia_lockardii

`--| ,----- Hanskerpia_hamiltonensis

| | ,-- Voltzia_hexagona

`--| |	,-- Nothodacrium_warrenii

| | ,----- Rissikia_media

`--| | ,-- Telemachus_aequatus

| | | ,-- Mataia_podocarpoides

`--| | |	,-- Eathiestrobus_mackenziei

| | | |-- Pseudolarix_amabilis

| | | |-- Pinus_contorta

`--| |	|-- Picea_abies

| | |-- Cedrus_deodara

| | |-- Cathaya_argyrophylla

| | | ,-- Tsuga_canadensis

| | ,--+----- Nothotsuga_longibracteata

`--| | | ,-- Pseudotsuga_menziesii

| | |----- Larix_decidua

| | | ,-- Keteleeria_davidiana

| | |----- Abies_lasiocarpa

| | | ,-- Schizolepidopsis_ediae

| | `--| ,-- Schizolepidopsis_daohugouensis

| | `----- Schizolepidopsis_canicularis

| | ,-- Sciadopitys_verticillata

`--| ,--| ,-- Taxus_wallichiana

| | `--| ,-- Cunninghamia_lanceolata

| | `--| ,-- Taiwania_cryptomerioides

| | `--| ,-- Athrotaxis_selaginoides

| | `--| ,-- Sequoia_sempervirens

| | `--| ,-- Cryptomeria_japonica

| | `--| ,-- Cupressus_funebris

| | `----- Callitris_muelleri

`--|	,-- Pararaucaria_patagonica

| | ,-- New_total_plant_from_Arkansas

| ,--| ,----- Alvinia_bohemica_plant

| | `--| ,-- Himeriella_muensteri

| | `--| ,-- Kachaikestrobus_acuminatus

| | `----- Tomaxellia_biforme

| | ,-- Araucaria_mirabilis

`--|	,----- Araucaria_heterophylla

| ,--| ,-- Wollemia_nobilis

| | `----- Agathis_australis

| | ,-- Lagarostrobos_franklinii

`--|	,----- Prumnopitys_taxifolia

| ,--| ,-- Lepidothamnus_laxifolius

| | `----- Phyllocladus_aspleniifolius

`--| ,-- Saxegothaea_conspicua

`--| ,-- Microcachrys_tetragona

`--| ,-- Pherosphaera_hookeriana

`--| ,-- Acmopyle_pancheri

`--| ,-- Dacrydium_cupressinum

`--| ,-- Retrophyllum_rospigliosii

`----- Podocarpus_archboldii

(ii) Majority rule tree (from 5 trees, cut 50)

,-- Ginkgo_biloba

| ,-- Cordaixylon_dumusum

|--| | ,-- Emporia_lockardii

`--100|	,--100--- Hanskerpia_hamiltonensis

| | ,-- Voltzia_hexagona

`--100|	|	,-- Nothodacrium_warrenii

| | ,--100--- Rissikia_media

`--100|	|	,-- Telemachus_aequatus

| | | ,-- Mataia_podocarpoides

`--100|	|	|	,-- Eathiestrobus_mackenziei

| | | | ,-- Pinus_contorta

| | | | |-- Picea_abies

`--100|	|	,--100|	|-- Cathaya_argyrophylla

| | | | | ,-- Pseudotsuga_menziesii

| | | | |--100--- Larix_decidua

| | | `--60| ,-- Schizolepidopsis_ediae

`--100|	|	|--100|	,-- Schizolepidopsis_daohugouensis

| | | `--100--- Schizolepidopsis_canicularis

| | | ,-- Cedrus_deodara

| | | | ,-- Keteleeria_davidiana

| | `--60| ,--100--- Abies_lasiocarpa

| | `--80| ,-- Pseudolarix_amabilis

| | `--80| ,-- Tsuga_canadensis

`--100|	`--100--- Nothotsuga_longibracteata

| ,-- Sciadopitys_verticillata

| ,--100| ,-- Taxus_wallichiana

| | `--100| ,-- Cunninghamia_lanceolata

| | `--100| ,-- Taiwania_cryptomerioides

| | `--100| ,-- Athrotaxis_selaginoides

| | `--100| ,-- Sequoia_sempervirens

| | `--100| ,-- Cryptomeria_japonica

| | `--100| ,-- Cupressus_funeb

| | `--100--- Callitris_muelle

`--100|	,-- Pararaucaria_patagonica

| | ,-- New_total_plant_from_Arkansas

| ,--100| ,--100--- Alvinia_bohemica_plant

| | `--100| ,-- Himeriella_muensteri

| | `--100| ,-- Kachaikestrobus_acuminatus

| | `--100--- Tomaxellia_biforme

| | ,-- Araucaria_mirabilis

`--100|	,--100--- Araucaria_heterophylla

| ,--100| ,-- Wollemia_nobilis

| | `--100--- Agathis_australis

| | ,-- Lagarostrobos_franklinii

`--100|	,--100--- Prumnopitys_taxifolia

| ,--100| ,-- Lepidothamnus_laxifolius

| | `--100--- Phyllocladus_aspleniifolius

`--100|	,-- Saxegothaea_conspicua

`--100|	,-- Microcachrys_tetragona

`--100|	,-- Pherosphaera_hookeriana

`--100|	,-- Acmopyle_pancheri

`--100|	,-- Dacrydium_cupres

`--100|	,-- Retroph_ro

`--100--- Podoc_arch

(iii) Reduced strict consensus of 5 trees

,-- Ginkgo_biloba

| ,-- Cordaixylon_dumusum

|--| | ,-- Emporia_lockardii

| | ,----- Hanskerpia_hamiltonensis

`--| | ,-- Voltzia_hexagona

| | | ,-- Nothodacrium_warrenii

`--| | ,----- Rissikia_media

| | | ,-- Mataia_podocarpoides

`--| |	|	,-- Eathiestrobus_mackenziei

| | | |-- Pseudolarix_amabilis

| | | |-- Pinus_contorta

`--|	|	|-- Picea_abies

| | |-- Cedrus_deodara

| | |-- Cathaya_argyrophylla

| | | ,-- Tsuga_canadensis

`--a| ,--+----- Nothotsuga_longibracteata

| | | ,-- Pseudotsuga_menziesii

| | |----- Larix_decidua

| | | ,-- Keteleeria_davidiana

| | |----- Abies_lasiocarpa

| | | ,-- Schizolepidopsis_ediae

| | `--| ,-- Schizolepidopsis_daohugouensis

| | `----- Schizolepidopsis_canicularis

`--|	,-- Sciadopitys_verticillata

| ,--| ,-- Taxus_wallichiana

| | `--| ,-- Cunninghamia_lanceolata

| | `--| ,-- Taiwania_cryptomerioides

| | `--| ,-- Athrotaxis_selaginoides

| | `--| ,-- Sequoia_sempervirens

| | `--| ,-- Cryptomeria_japonica

| | `--| ,-- Cupressus_funebris

`--|	`----- Callitris_muelleri

| ,-- Araucaria_mirabilis

| ,----- Araucaria_heterophylla

| ,--| ,-- Wollemia_nobilis

| | `----- Agathis_australis

| | ,-- Lagarostrobos_franklinii

`--b|	,----- Prumnopitys_taxifolia

| ,--| ,-- Lepidothamnus_laxifolius

| | `----- Phyllocladus_aspleniifolius

`--| ,-- Saxegothaea_conspicua

`--| ,-- Microcachrys_tetragona

`--| ,-- Pherosphaera_hookeriana

`--| ,-- Acmopyle_pancheri

`--| ,-- Dacrydium_cupressinum

`--| ,-- Retrophyllum_rospigliosii

**Legends:** `----- Podocarpus_archboldii

a: Telemachus_aequatus (40)

b: node 92 of consensus

,-- Pararaucaria_patagonica

| ,-- New_total_plant_from_Arkansas

|--| ,----- Alvinia_bohemica_plant

`--| ,-- Himeriella_muensteri

`--| ,-- Kachaikestrobus_acuminatus

`----- Tomaxellia_biform
