# Supplementary material for: Revisiting the enigmatic Cheirolepidiaceae: origins, phylogenetic relationships, and a new whole-plant concept
Source: Ann Bot. 2026 Mar 30;137(6):1876–901. doi: 10.1093/aob/mcag069 (PMC13275020; doi:10.1093/aob/mcag069)
Supplement: mcag069_Supplementary_Data [file mcag069_supplementary_data.zip › AppendixS2-ParsimonyAnalyses.zip!/AppendixS2-ParsimonyAnalyses/AppendixS2A-EqWeights_TreeSummarization_NoConstraints/Appendix2Ai-sumarizationEWtrees_NoConstrains.docx]

**Summarization of trees from equal weights searches**

**These trees do not have a constraint excluding older voltzialean from the crown group.**

**For half of these tree searches a constrain for the two main clades of Pinaceaea was implemented (with all fossils considered as floaters).**

**INDEX**

**Metaconsensus of the 10 equal weight tree searches detailed below with alternative positions of *Mataia podocarpoides* and synapomorphies of all nodes of interest______________________p2–5**

**Character 64 active and treated as unordered, constrain of Pinaceae main clades on** (tree file: MPT-EW-cons-64unord.tre; 6 trees read).

**Character 64 active and treated as additive, constrain of Pinaceae main clades on** (tree file: MPT-EW-cons-64ad.tre; 6 trees read).

**Character 0 active and standardized to 1, constrain of Pinaceae main clades off** (tree file: MPT-EW-c0s1.tre; 20 trees read).

**Character 0 active and standardized to 1, constrain of Pinaceae main clades on**

(tree file: MPT-EW-cons-c0s1.tre; 12 trees read).

**Character 0 active and standardized to 5, constrain of Pinaceae main clades off** (tree file: MPT-EW-c0s5.tre; 20 trees read).

**Character 0 active and standardized to 5, constrain of Pinaceae main clades on**

(tree file: MPT-EW-cons-c0s5.tre; 12 trees read).

**Character 1 active and standardized to 1, constrain of Pinaceae main clades off** (tree file: MPT-EW-c1s1.tre: 20 trees read).

**Character 1 active and standardized to 1, constrain of Pinaceae main clades on**

(tree file: MPT-EW-cons-c1s1.tre; 12 trees read).

**Character 1 active and standardized to 5, constrain of Pinaceae main clades on**

(tree file: MPT-EW-c1s5.tre; 20 trees read).

**Character 1 active and standardized to 5, constrain of Pinaceae main clades on**

(tree file: MPT-EW-cons-c1s5.tre; 12 trees read).

**All most parsimonious trees for the analysis detailed below**___p6–117

**Character 64 active and treated as unordered, constrain of Pinaceae main clades off** (tree file: MPT-EW-64unord.tre; 112 trees read). Voltziales and Cordaitales in pink; Cheirolepidiaceae in orange; new species in bold orange.

**All most parsimonious trees for the analysis detailed below**_p118–199

**Character 64 active and treated as additive, constrain of Pinaceae main clades off** (tree file: MPT-EW-64ad.tre; 82 trees read). Voltziales and Cordaitales in pink; Cheirolepidiaceae in orange; new species in bold orange.

**Strict meta-consensus of 140 trees (showing alternstive positions of *Mataia*)**

,-- *Ginkgo* *biloba*

| ,-- *Eathiestrobus* *mackenziei*

| |-- *Pseudolarix* *amabilis*

| |-- *Pinus* *contorta*

| |-- *Picea* *abies*

| |-- *Cedrus* *deodara*

| |-- *Cathaya* *argyrophylla*

| | ,-- *Tsuga canadensis*

| ,--+----- *Nothotsuga longibracteata*

| | | ,-- *Pseudotsuga menziesii*

|--| | |----- *Larix decidua*

| | | ,-- *Keteleeria davidiana*

| | |----- *Abies lasiocarpa*

| | | ,-- *Schizolepidopsis ediae*

| | `--| ,-- *Schizolepidopsis daohugouensis*

| | `----- *Schizolepidopsis canicularis*

| | ,-- *Araucaria mirabilis*

| | ,----- *Araucaria heterophylla*

| | ,--| ,-- *Wollemia nobilis*

`--**a**|	| `----- *Agathis australis*

| | ,-- *Lagarostrobos* *franklinii*

| ,--| ,----- *Prumnopitys taxifolia*

| | | ,--| ,-- *Lepidothamnus laxifolius*

| | | | `----- *Phyllocladus aspleniifolius*

| | `--| ,-- *Saxegothaea conspicua*

| | `--| ,-- *Microcachrys tetragona*

| | `--| ,-- *Pherosphaera hookeriana*

| | `--| ,-- *Acmopyle pancheri*

| | `--| ,-- *Dacrydium cupressinum*

`--**a**|	`--| ,-- *Retrophyllum rospigliosii*

| `----- *Podocarpus archboldii*

| ,-- *Pararaucaria patagonica*

| | ,-- **New total plant from Arkansas**

| ,--| ,----- *Alvinia bohemica* plant

| | `--| ,-- *Himeriella muensteri*

| | `--| ,-- *Kachaikestrobus acuminatus*

| | `----- *Tomaxellia biforme*

`--| ,-- *Sciadopitys verticillata*

| | ,-- *Cunninghamia lanceolata*

| | ,--| ,-- *Taiwania cryptomerioides*

`--| | `--| ,-- *Athrotaxis selaginoides*

| | `--| ,-- *Sequoia sempervirens*

| | `--| ,-- *Cryptomeria* *japonica*

`--|	`--| ,-- *Cupressus funebris*

| `----- *Callitris muelleri*

| ,-- *Telemachus aequatus*

| | ,-- *Nothodacrium warrenii*

`--| |-- *Rissikia media*

`--| ,-- *Voltzia hexagona*

| | ,-- *Emporia lockardii*

`--| ,----- *Hanskerpia hamiltonensis*

`--| ,-- *Cordaixylon* *dumusum*

`----- *Taxus wallichiana*

Legends:

**a: *Mataia podocarpoides* (47)**

**Strict meta-consensus of 140 trees (number corresponds to node number)**

,--34 *Ginkgo biloba*

| ,--47 *Mataia podocarpoides*

| | ,--41 *Eathiestrobus mackenziei*

| | |--9 *Pseudolarix* *amabilis*

| | |--7 *Pinus contorta*

| | |--6 *Picea abies*

| | |--2 *Cedrus deodara*

| | |--1 *Cathaya argyrophylla*

|--55| | | ,--10 *Tsuga canadensis*

| |--57+--60---5 *Nothotsuga longibracteata*

| | | ,--8 *Pseudotsuga menziesii*

| | |--59---4 *Larix decidua*

| | | ,--3 *Keteleeria davidiana*

| | |--56---0 *Abies lasiocarpa*

| | | ,--35 *Schizolepidopsis ediae*

| | `--90| ,--43 *Schizolepidopsis daohugouensis*

`--58|	`--93---42 *Schizolepidopsis canicularis*

| ,--54 *Araucaria mirabilis*

| ,--65---12 *Araucaria heterophylla*

| ,--62| ,--13 *Wollemia nobilis*

| | `--61---11 *Agathis australis*

| | ,--24 *Lagarostrobos franklinii*

| ,--63| ,--75---18 *Prumnopitys taxifolia*

| | | ,--73| ,--23 *Lepidothamnus* *laxifolius*

| | | | `--72---16 *Phyllocladus* *aspleniifolius*

| | `--70| ,--19 *Saxegothaea conspicua*

| | `--69| ,--20 *Microcachrys tetragona*

| | `--68| ,--21 *Pherosphaera hookeriana*

| | `--67| ,--14 *Acmopyle pancheri*

| | `--66| ,--15 *Dacrydium cupressinum*

`--64|	`--71|	,--22 *Retrophyllum rospigliosii*

| `--74---17 *Podocarpus archboldii*

| ,--36 *Pararaucaria* *patagonica*

| | ,--53 **New total plant from Arkansas**

| ,--**91**| ,--97---52 *Alvinia bohemica* plant

| | `--96| ,--50 *Himeriella muensteri*

| | `--95| ,--51 *Kachaikestrobus acuminatus*

| | `--94---49 *Tomaxellia biforme*

`--**77**|	,--25 *Sciadopitys verticillata*

| | ,--29 *Cunninghamia lanceolata*

| | ,--**80**| ,--32 *Taiwania cryptomerioides*

`--76|	|	`--79|	,--26 *Athrotaxis selaginoides*

| | `--78| ,--31 *Sequoia sempervirens*

| | `--84| ,--28 *Cryptomeria japonica*

`--**81**|	`--83|	,--30 *Cupressus funebris*

| `--82---27 *Callitris muelleri*

| ,--40 *Telemachus aequatus*

| | ,--46 *Nothodacrium* *warrenii*

`--**89**|	|--45 *Rissikia media*

`--88|	,--44 *Voltzia hexagona*

| | ,--39 *Emporia lockardii*

`--87|	,--92---37 *Hanskerpia hamiltonensis*

`--86|	,--38 *Cordaixylon dumusum*

`--85---33 *Taxus wallichiana*

**Synapomorphies common to 140 trees**

(Node numbers refer to nodes in consensus)

**Node 77** (Cheiro + Cupressales + Weirdly rooted Voltzialeans & Cordait & Taxus):

All trees:

Char. 23: 0 --> 2

*Number of ovuliferous scale lobes*: one 🡪 three to five

Char. 61: 4 --> 3

*Degree of fusion of the scale elements*: Completely fused 🡪 Nearly completely fused (bearly lobed)

**Node 80**(Cupressaceae):

All trees:

Char. 20: 0 --> 1

*Seed number per ovuliferous complex variable in cone*: absent 🡪 present

Char. 36: 0 --> 2

*Bract-ovuliferous scale/shoot relative length*: bract < scale 🡪 bract > scale

Char. 37: 0 --> 1

*Dominant structure protecting/bearing the seed*: ovuliferous scale 🡪 bract

90 DNA sites [**5 sites of** ***18s*** (252, 355, 764, 837, 1637), **35 sites of** **PHYP** (2477, 2507, 2710, 2801, 2810, 2875, 2903, 2906, 2919, 2927, 2960, 3113, 3194, 3209, 3236, 3242, 3302, 3404, 3488, 3494, 3623, 3665, 3701, 3719, 3731, 3791, 3822, 3852, 3854, 3884, 3902, 3921, 3929, 3935, 3963), **39 sites of** **matK** (4392, 4462, 4477, 4490, 4530, 4547, 4571, 4598, 4615, 4616, 4629, 4637, 4726, 4807, 4842, 4880, 4914, 4928, 4929, 4965, 5011, 5026, 5042, 5064, 5279, 5293, 5310, 5331, 5378, 5379, 5458, 5461, 5491, 5508, 5512, 5651, 5752, 5764, 5812), **11 sites of** **rbcL** (6085, 6094, 6202, 6253, 6329, 6574, 6659, 6799, 6871, 7135, 7195)]

Some trees:

2 DNA sites: PHYP (2876), and rbcL (6490)

**Node 81**(Cupressaceae + weirdly rooted Voltzialeans & Cordait & Taxus):

All trees:

97 DNA sites [**6 sites of** **18s** (403, 1629, 1646, 1647, 1843, 1848), **45 sites of** **PHYP** 2398, 2410, 2411, 2414, 2419, 2420, 2423, 2437, 2438, 2442, 2444, 2451, 2453, 2454, 2460, 2606, 2613, 2645, 2651, 2678, 2711, 2766, 2780, 2789, 2841, 2854, 2855, 2856, 2900, 2918, 2957, 2986, 3009, 3024, 3066, 3119, 3224, 3269, 3285, 3566, 3650, 3750, 3881, 3887, 3955), **28 sites of** **matK** (4416, 4495, 4499, 4500, 4572, 4663, 4682, 4703, 4715, 4730, 4783, 4923, 4980, 5027, 5052, 5233, 5275, 5311, 5352, 5363, 5485, 5486, 5490, 5546, 5561, 5630, 5852, 5900), **18 sites of rbcL** (6199, 6205, 6225, 6355, 6391, 6397, 6412, 6553, 6595, 6643, 6754, 6986, 7048, 7057, 7081, 7153, 7279, 7287)]

Some trees:

1 DNA site: matK (4963)

**Node 89** (clade of weirdly rooted Voltzialeans & Cordait & Taxus):

All trees:

Char. 49: 1 --> 0

*Resin canals in bract*: present 🡪 absent

Char. 59: 0 --> 1

*Bract sclerenchyma*: absent 🡪 present

Char. 94: 1 --> 0

*Spongy parenchyma*: relative density: Lax 🡪 Dense

Char. 107: 0 --> 1

*Pollen sacci*: Absent 🡪 Present

**Node 91** (Cheirolepidiaceae):

All trees:

Char. 40: 0 --> 1

*Scale or seed trichomes/papillae*: absent 🡪 present

**Node 96** (Core Cheirolepidiaceae):

All trees:

Char. 61: 3 --> 2

Degree of fusion of the scale elements: Nearly completely fused (bearly lobed) 🡪 Fused 60-85% of total length

**Character 64 active and treated as unordered, constrain of Pinaceae main clades off (All most parsimonious trees)**

Tree 0:

,-- Ginkgo_biloba

| ,-- Cordaixylon_dumusum

|--| | ,-- Emporia_lockardii

| | ,----- Hanskerpia_hamiltonensis

`--| | ,-- Voltzia_hexagona

| | | ,-- Nothodacrium_warrenii

`--| | ,----- Rissikia_media

| | | ,-- Pararaucaria_patagonica

`--| |	|	,-- **New_total_plant_from_Arkansas**

| | ,--| ,----- Alvinia_bohemica_plant

| | | `--| ,-- Himeriella_muensteri

`--| |	`--| ,-- Kachaikestrobus_acuminatus

| | `----- Tomaxellia_biforme

| | ,-- Telemachus_aequatus

| | | ,-- Eathiestrobus_mackenziei

`--| |	| ,-- Cedrus_deodara

| | ,--| | ,-- Pseudotsuga_menziesii

| | | | | ,----- Larix_decidua

| | | `--| ,--| ,-- Cathaya_argyrophylla

| | | | | `--| ,-- Pinus_contorta

`--| |	| |	`----- Picea_abies

| | `--| ,-- Mataia_podocarpoides

| | | ,--| ,-- Keteleeria_davidiana

| | | | `----- Abies_lasiocarpa

| | `--| ,-- Pseudolarix_amabilis

| | | | ,-- Tsuga_canadensis

| | `--| ,----- Nothotsuga_longibracteata

`--|	`--| ,-- Schizolepidopsis_ediae

| `--| ,-- Schizolepidopsis_daohugouensis

| `----- Schizolepidopsis_canicularis

| ,-- Sciadopitys_verticillata

| ,--| ,-- Taxus_wallichiana

| | `--| ,-- Cunninghamia_lanceolata

| | `--| ,-- Taiwania_cryptomerioides

| | `--| ,-- Athrotaxis_selaginoides

| | `--| ,-- Sequoia_sempervirens

| | `--| ,-- Cryptomeria_japonica

| | `--| ,-- Cupressus_funebris

`--|	`----- Callitris_muelleri

| ,-- Araucaria_mirabilis

| ,----- Araucaria_heterophylla

| ,--| ,-- Wollemia_nobilis

| | `----- Agathis_australis

| | ,-- Lagarostrobos_franklinii

`--|	,----- Prumnopitys_taxifolia

| ,--| ,-- Lepidothamnus_laxifolius

| | `----- Phyllocladus_aspleniifolius

`--| ,-- Saxegothaea_conspicua

`--| ,-- Microcachrys_tetragona

`--| ,-- Pherosphaera_hookeriana

`--| ,-- Acmopyle_pancheri

`--| ,-- Dacrydium_cupressinum

`--| ,-- Retrophyllum_rospigliosii

`----- Podocarpus_archboldii

Tree 1:

,-- Ginkgo_biloba

| ,-- Cordaixylon_dumusum

|--| | ,-- Emporia_lockardii

| | ,----- Hanskerpia_hamiltonensis

`--| | ,-- Voltzia_hexagona

| | | ,-- Nothodacrium_warrenii

`--| | ,----- Rissikia_media

| | | ,-- Alvinia_bohemica_plant

`--| |	,--| ,-- **New_total_plant_from_Arkansas**

| | | `----- Pararaucaria_patagonica

| | ,--| ,-- Himeriella_muensteri

`--| | `--| ,-- Kachaikestrobus_acuminatus

| | `----- Tomaxellia_biforme

| | ,-- Telemachus_aequatus

| | | ,-- Eathiestrobus_mackenziei

`--| |	| ,-- Cedrus_deodara

| | ,--| | ,-- Pseudotsuga_menziesii

| | | | | ,----- Larix_decidua

| | | `--| ,--| ,-- Cathaya_argyrophylla

| | | | | `--| ,-- Pinus_contorta

`--| |	| |	`----- Picea_abies

| | `--| ,-- Mataia_podocarpoides

| | | ,--| ,-- Keteleeria_davidiana

| | | | `----- Abies_lasiocarpa

| | `--| ,-- Pseudolarix_amabilis

| | | | ,-- Tsuga_canadensis

| | `--| ,----- Nothotsuga_longibracteata

`--|	`--| ,-- Schizolepidopsis_ediae

| `--| ,-- Schizolepidopsis_daohugouensis

| `----- Schizolepidopsis_canicularis

| ,-- Sciadopitys_verticillata

| ,--| ,-- Taxus_wallichiana

| | `--| ,-- Cunninghamia_lanceolata

| | `--| ,-- Taiwania_cryptomerioides

| | `--| ,-- Athrotaxis_selaginoides

| | `--| ,-- Sequoia_sempervirens

| | `--| ,-- Cryptomeria_japonica

| | `--| ,-- Cupressus_funebris

`--|	`----- Callitris_muelleri

| ,-- Araucaria_mirabilis

| ,----- Araucaria_heterophylla

| ,--| ,-- Wollemia_nobilis

| | `----- Agathis_australis

| | ,-- Lagarostrobos_franklinii

`--|	,----- Prumnopitys_taxifolia

| ,--| ,-- Lepidothamnus_laxifolius

| | `----- Phyllocladus_aspleniifolius

`--| ,-- Saxegothaea_conspicua

`--| ,-- Microcachrys_tetragona

`--| ,-- Pherosphaera_hookeriana

`--| ,-- Acmopyle_pancheri

`--| ,-- Dacrydium_cupressinum

`--| ,-- Retrophyllum_rospigliosii

`----- Podocarpus_archboldii

Tree 2:

,-- Ginkgo_biloba

| ,-- Mataia_podocarpoides

| | ,-- Cedrus_deodara

| | | ,-- Pseudotsuga_menziesii

| | | ,----- Larix_decidua

|--| | | ,--| ,-- Cathaya_argyrophylla

| | ,--| | `--| ,-- Pinus_contorta

| | | | | `--| ,-- Eathiestrobus_mackenziei

| | | | | `----- Picea_abies

| | | `--| ,-- Keteleeria_davidiana

`--| |	| ,----- Abies_lasiocarpa

| | | | ,-- Pseudolarix_amabilis

| | `--| | ,-- Tsuga_canadensis

| | `--| ,----- Nothotsuga_longibracteata

| | `--| ,-- Schizolepidopsis_ediae

| | `--| ,-- Schizolepidopsis_daohugouensis

| | `----- Schizolepidopsis_canicularis

| | ,-- Araucaria_mirabilis

`--|	,----- Araucaria_heterophylla

| ,--| ,-- Wollemia_nobilis

| | `----- Agathis_australis

| | ,-- Lagarostrobos_franklinii

| ,--| ,----- Prumnopitys_taxifolia

| | | ,--| ,-- Lepidothamnus_laxifolius

| | | | `----- Phyllocladus_aspleniifolius

| | `--| ,-- Saxegothaea_conspicua

| | `--| ,-- Microcachrys_tetragona

| | `--| ,-- Pherosphaera_hookeriana

| | `--| ,-- Acmopyle_pancheri

| | `--| ,-- Dacrydium_cupressinum

`--|	`--| ,-- Retrophyllum_rospigliosii

| `----- Podocarpus_archboldii

| ,-- Pararaucaria_patagonica

| | ,-- **New_total_plant_from_Arkansas**

| ,--| ,----- Alvinia_bohemica_plant

| | `--| ,-- Himeriella_muensteri

| | `--| ,-- Kachaikestrobus_acuminatus

| | `----- Tomaxellia_biforme

`--| ,-- Sciadopitys_verticillata

| | ,-- Cunninghamia_lanceolata

| | ,--| ,-- Taiwania_cryptomerioides

`--| | `--| ,-- Athrotaxis_selaginoides

| | `--| ,-- Sequoia_sempervirens

| | `--| ,-- Cryptomeria_japonica

`--|	`--| ,-- Cupressus_funebris

| `----- Callitris_muelleri

| ,-- Telemachus_aequatus

| | ,-- Nothodacrium_warrenii

`--| ,----- Rissikia_media

| | ,-- Voltzia_hexagona

`--| |	,-- Emporia_lockardii

`--| ,----- Hanskerpia_hamiltonensis

`--| ,-- Cordaixylon_dumusum

`----- Taxus_wallichiana

Tree 3:

,-- Ginkgo_biloba

| ,-- Cordaixylon_dumusum

|--| | ,-- Emporia_lockardii

| | ,----- Hanskerpia_hamiltonensis

`--| | ,-- Voltzia_hexagona

| | | ,-- Nothodacrium_warrenii

`--| | ,----- Rissikia_media

| | | ,-- Telemachus_aequatus

`--| | |	,-- Schizolepidopsis_daohugouensis

| | | ,----- Schizolepidopsis_canicularis

| | | | ,-- Schizolepidopsis_ediae

`--| | ,--| | ,-- Cedrus_deodara

| | | | | | ,-- Keteleeria_davidiana

| | | `--| | ,----- Abies_lasiocarpa

| | | | | | ,-- Mataia_podocarpoides

`--| |	`--| ,--| ,----- Pseudolarix_amabilis

| | | | `--| ,-- Tsuga_canadensis

| | | | `----- Nothotsuga_longibracteata

| | `--| ,-- Pseudotsuga_menziesii

| | | ,----- Larix_decidua

| | `--| ,-- Cathaya_argyrophylla

| | `--| ,-- Picea_abies

`--|	`--| ,-- Eathiestrobus_mackenziei

| `----- Pinus_contorta

| ,-- Sciadopitys_verticillata

| ,--| ,-- Taxus_wallichiana

| | `--| ,-- Cunninghamia_lanceolata

| | `--| ,-- Taiwania_cryptomerioides

| | `--| ,-- Athrotaxis_selaginoides

| | `--| ,-- Sequoia_sempervirens

| | `--| ,-- Cryptomeria_japonica

| | `--| ,-- Cupressus_funebris

| | `----- Callitris_muelleri

`--|	,-- Pararaucaria_patagonica

| | ,-- **New_total_plant_from_Arkansas**

| ,--| ,----- Alvinia_bohemica_plant

| | `--| ,-- Himeriella_muensteri

| | `--| ,-- Kachaikestrobus_acuminatus

| | `----- Tomaxellia_biforme

| | ,-- Araucaria_mirabilis

`--|	,----- Araucaria_heterophylla

| ,--| ,-- Wollemia_nobilis

| | `----- Agathis_australis

| | ,-- Lagarostrobos_franklinii

`--|	,----- Prumnopitys_taxifolia

| ,--| ,-- Lepidothamnus_laxifolius

| | `----- Phyllocladus_aspleniifolius

`--| ,-- Saxegothaea_conspicua

`--| ,-- Microcachrys_tetragona

`--| ,-- Pherosphaera_hookeriana

`--| ,-- Acmopyle_pancheri

`--| ,-- Dacrydium_cupressinum

`--| ,-- Retrophyllum_rospigliosii

`----- Podocarpus_archboldii

Tree 4:

,-- Ginkgo_biloba

| ,-- Cordaixylon_dumusum

|--| | ,-- Emporia_lockardii

| | ,----- Hanskerpia_hamiltonensis

`--| | ,-- Voltzia_hexagona

| | | ,-- Nothodacrium_warrenii

`--| | ,----- Rissikia_media

| | | ,-- Alvinia_bohemica_plant

`--| |	,--| ,-- **New_total_plant_from_Arkansas**

| | | `----- Pararaucaria_patagonica

| | ,--| ,-- Himeriella_muensteri

`--| | `--| ,-- Kachaikestrobus_acuminatus

| | `----- Tomaxellia_biforme

| | ,-- Telemachus_aequatus

| | | ,-- Eathiestrobus_mackenziei

`--| |	| ,-- Cedrus_deodara

| | ,--| | ,-- Pseudotsuga_menziesii

| | | | | ,----- Larix_decidua

| | | `--| ,--| ,-- Cathaya_argyrophylla

| | | | | `--| ,-- Pinus_contorta

`--| |	| |	`----- Picea_abies

| | `--| ,-- Mataia_podocarpoides

| | | ,--| ,-- Keteleeria_davidiana

| | | | `----- Abies_lasiocarpa

| | `--| ,-- Pseudolarix_amabilis

| | | | ,-- Tsuga_canadensis

| | `--| ,----- Nothotsuga_longibracteata

`--|	`--| ,-- Schizolepidopsis_ediae

| `--| ,-- Schizolepidopsis_daohugouensis

| `----- Schizolepidopsis_canicularis

| ,-- Sciadopitys_verticillata

| ,--| ,-- Taxus_wallichiana

| | `--| ,-- Cunninghamia_lanceolata

| | `--| ,-- Taiwania_cryptomerioides

| | `--| ,-- Athrotaxis_selaginoides

| | `--| ,-- Sequoia_sempervirens

| | `--| ,-- Cryptomeria_japonica

| | `--| ,-- Cupressus_funebris

`--|	`----- Callitris_muelleri

| ,-- Araucaria_mirabilis

| ,----- Araucaria_heterophylla

| ,--| ,-- Wollemia_nobilis

| | `----- Agathis_australis

| | ,-- Lagarostrobos_franklinii

`--|	,----- Prumnopitys_taxifolia

| ,--| ,-- Lepidothamnus_laxifolius

| | `----- Phyllocladus_aspleniifolius

`--| ,-- Saxegothaea_conspicua

| | ,-- Pherosphaera_hookeriana

`--| ,----- Microcachrys_tetragona

`--| ,-- Acmopyle_pancheri

`--| ,-- Dacrydium_cupressinum

`--| ,-- Retrophyllum_rospigliosii

`----- Podocarpus_archboldii

Tree 5:

,-- Ginkgo_biloba

| ,-- Mataia_podocarpoides

| | ,-- Cedrus_deodara

| | | ,-- Pseudotsuga_menziesii

| | | ,----- Larix_decidua

|--| | | ,--| ,-- Cathaya_argyrophylla

| | ,--| | `--| ,-- Picea_abies

| | | | | `--| ,-- Eathiestrobus_mackenziei

| | | | | `----- Pinus_contorta

| | | `--| ,-- Keteleeria_davidiana

`--| |	| ,----- Abies_lasiocarpa

| | | | ,-- Pseudolarix_amabilis

| | `--| | ,-- Tsuga_canadensis

| | `--| ,----- Nothotsuga_longibracteata

| | `--| ,-- Schizolepidopsis_ediae

| | `--| ,-- Schizolepidopsis_daohugouensis

| | `----- Schizolepidopsis_canicularis

| | ,-- Araucaria_mirabilis

`--|	,----- Araucaria_heterophylla

| ,--| ,-- Wollemia_nobilis

| | `----- Agathis_australis

| | ,-- Lagarostrobos_franklinii

| ,--| ,----- Prumnopitys_taxifolia

| | | ,--| ,-- Lepidothamnus_laxifolius

| | | | `----- Phyllocladus_aspleniifolius

| | `--| ,-- Saxegothaea_conspicua

| | `--| ,-- Microcachrys_tetragona

| | `--| ,-- Pherosphaera_hookeriana

| | `--| ,-- Acmopyle_pancheri

| | `--| ,-- Dacrydium_cupressinum

`--|	`--| ,-- Retrophyllum_rospigliosii

| `----- Podocarpus_archboldii

| ,-- Pararaucaria_patagonica

| | ,-- **New_total_plant_from_Arkansas**

| ,--| ,----- Alvinia_bohemica_plant

| | `--| ,-- Himeriella_muensteri

| | `--| ,-- Kachaikestrobus_acuminatus

| | `----- Tomaxellia_biforme

`--| ,-- Sciadopitys_verticillata

| | ,-- Cunninghamia_lanceolata

| | ,--| ,-- Taiwania_cryptomerioides

`--| | `--| ,-- Athrotaxis_selaginoides

| | `--| ,-- Sequoia_sempervirens

| | `--| ,-- Cryptomeria_japonica

`--|	`--| ,-- Cupressus_funebris

| `----- Callitris_muelleri

| ,-- Telemachus_aequatus

| | ,-- Nothodacrium_warrenii

`--| ,----- Rissikia_media

| | ,-- Voltzia_hexagona

`--| |	,-- Emporia_lockardii

`--| ,----- Hanskerpia_hamiltonensis

`--| ,-- Cordaixylon_dumusum

`----- Taxus_wallichiana

Tree 6:

,-- Ginkgo_biloba

| ,-- Cordaixylon_dumusum

|--| | ,-- Emporia_lockardii

| | ,----- Hanskerpia_hamiltonensis

`--| | ,-- Voltzia_hexagona

| | | ,-- Nothodacrium_warrenii

`--| | ,----- Rissikia_media

| | | ,-- Telemachus_aequatus

`--| | |	,-- Schizolepidopsis_ediae

| | | ,--| ,-- Schizolepidopsis_daohugouensis

| | | | `----- Schizolepidopsis_canicularis

`--| |	| ,-- Cedrus_deodara

| | ,--| | ,-- Mataia_podocarpoides

| | | | | | ,-- Keteleeria_davidiana

| | | `--| ,--| ,----- Abies_lasiocarpa

`--| |	| | `--| ,-- Pseudolarix_amabilis

| | | | `--| ,-- Tsuga_canadensis

| | `--| `----- Nothotsuga_longibracteata

| | | ,-- Pseudotsuga_menziesii

| | | ,----- Larix_decidua

| | `--| ,-- Cathaya_argyrophylla

| | `--| ,-- Pinus_contorta

| | `--| ,-- Eathiestrobus_mackenziei

`--|	`----- Picea_abies

| ,-- Sciadopitys_verticillata

| ,--| ,-- Taxus_wallichiana

| | `--| ,-- Cunninghamia_lanceolata

| | `--| ,-- Taiwania_cryptomerioides

| | `--| ,-- Athrotaxis_selaginoides

| | `--| ,-- Sequoia_sempervirens

| | `--| ,-- Cryptomeria_japonica

| | `--| ,-- Cupressus_funebris

| | `----- Callitris_muelleri

`--|	,-- Pararaucaria_patagonica

| | ,-- **New_total_plant_from_Arkansas**

| ,--| ,----- Alvinia_bohemica_plant

| | `--| ,-- Himeriella_muensteri

| | `--| ,-- Kachaikestrobus_acuminatus

| | `----- Tomaxellia_biforme

| | ,-- Araucaria_mirabilis

`--|	,----- Araucaria_heterophylla

| ,--| ,-- Wollemia_nobilis

| | `----- Agathis_australis

| | ,-- Lagarostrobos_franklinii

`--|	,----- Prumnopitys_taxifolia

| ,--| ,-- Lepidothamnus_laxifolius

| | `----- Phyllocladus_aspleniifolius

`--| ,-- Saxegothaea_conspicua

`--| ,-- Microcachrys_tetragona

`--| ,-- Pherosphaera_hookeriana

`--| ,-- Acmopyle_pancheri

`--| ,-- Dacrydium_cupressinum

`--| ,-- Retrophyllum_rospigliosii

`----- Podocarpus_archboldii

Tree 7:

,-- Ginkgo_biloba

| ,-- Mataia_podocarpoides

| | ,-- Eathiestrobus_mackenziei

| | | ,-- Cedrus_deodara

| | ,--| | ,-- Pseudotsuga_menziesii

|--| | | | | ,----- Larix_decidua

| | | `--| ,--| ,-- Cathaya_argyrophylla

| | | | | `--| ,-- Pinus_contorta

| | | | | `----- Picea_abies

`--| |	`--|	,-- Keteleeria_davidiana

| | | ,----- Abies_lasiocarpa

| | | | ,-- Pseudolarix_amabilis

| | `--| | ,-- Tsuga_canadensis

| | `--| ,----- Nothotsuga_longibracteata

| | `--| ,-- Schizolepidopsis_ediae

| | `--| ,-- Schizolepidopsis_daohugouensis

| | `----- Schizolepidopsis_canicularis

`--|	,-- Araucaria_mirabilis

| ,----- Araucaria_heterophylla

| ,--| ,-- Wollemia_nobilis

| | `----- Agathis_australis

| | ,-- Lagarostrobos_franklinii

| ,--| ,----- Prumnopitys_taxifolia

| | | ,--| ,-- Lepidothamnus_laxifolius

| | | | `----- Phyllocladus_aspleniifolius

| | `--| ,-- Saxegothaea_conspicua

| | `--| ,-- Microcachrys_tetragona

| | `--| ,-- Pherosphaera_hookeriana

| | `--| ,-- Acmopyle_pancheri

| | `--| ,-- Dacrydium_cupressinum

`--|	`--| ,-- Retrophyllum_rospigliosii

| `----- Podocarpus_archboldii

| ,-- Pararaucaria_patagonica

| | ,-- **New_total_plant_from_Arkansas**

| ,--| ,----- Alvinia_bohemica_plant

| | `--| ,-- Himeriella_muensteri

| | `--| ,-- Kachaikestrobus_acuminatus

| | `----- Tomaxellia_biforme

`--| ,-- Sciadopitys_verticillata

| | ,-- Cunninghamia_lanceolata

| | ,--| ,-- Taiwania_cryptomerioides

`--| | `--| ,-- Athrotaxis_selaginoides

| | `--| ,-- Sequoia_sempervirens

| | `--| ,-- Cryptomeria_japonica

`--|	`--| ,-- Cupressus_funebris

| `----- Callitris_muelleri

| ,-- Telemachus_aequatus

| | ,-- Nothodacrium_warrenii

`--| ,----- Rissikia_media

| | ,-- Voltzia_hexagona

`--| |	,-- Emporia_lockardii

`--| ,----- Hanskerpia_hamiltonensis

`--| ,-- Cordaixylon_dumusum

`----- Taxus_wallichiana

Tree 8:

,-- Ginkgo_biloba

| ,-- Cordaixylon_dumusum

|--| | ,-- Emporia_lockardii

| | ,----- Hanskerpia_hamiltonensis

`--| | ,-- Voltzia_hexagona

| | | ,-- Nothodacrium_warrenii

`--| | ,----- Rissikia_media

| | | ,-- Pararaucaria_patagonica

`--| |	|	,-- **New_total_plant_from_Arkansas**

| | ,--| ,----- Alvinia_bohemica_plant

| | | `--| ,-- Himeriella_muensteri

`--| |	`--| ,-- Kachaikestrobus_acuminatus

| | `----- Tomaxellia_biforme

| | ,-- Telemachus_aequatus

| | | ,-- Eathiestrobus_mackenziei

`--| |	| ,-- Cedrus_deodara

| | ,--| | ,-- Pseudotsuga_menziesii

| | | | | ,----- Larix_decidua

| | | `--| ,--| ,-- Cathaya_argyrophylla

| | | | | `--| ,-- Pinus_contorta

`--| |	| |	`----- Picea_abies

| | `--| ,-- Keteleeria_davidiana

| | | ,----- Abies_lasiocarpa

| | | | ,-- Pseudolarix_amabilis

| | `--| | ,-- Mataia_podocarpoides

| | `--| ,--| ,-- Tsuga_canadensis

| | | | `----- Nothotsuga_longibracteata

`--|	`--| ,-- Schizolepidopsis_ediae

| `--| ,-- Schizolepidopsis_daohugouensis

| `----- Schizolepidopsis_canicularis

| ,-- Sciadopitys_verticillata

| ,--| ,-- Taxus_wallichiana

| | `--| ,-- Cunninghamia_lanceolata

| | `--| ,-- Taiwania_cryptomerioides

| | `--| ,-- Athrotaxis_selaginoides

| | `--| ,-- Sequoia_sempervirens

| | `--| ,-- Cryptomeria_japonica

| | `--| ,-- Cupressus_funebris

`--|	`----- Callitris_muelleri

| ,-- Araucaria_mirabilis

| ,----- Araucaria_heterophylla

| ,--| ,-- Wollemia_nobilis

| | `----- Agathis_australis

| | ,-- Lagarostrobos_franklinii

`--|	,----- Prumnopitys_taxifolia

| ,--| ,-- Lepidothamnus_laxifolius

| | `----- Phyllocladus_aspleniifolius

`--| ,-- Saxegothaea_conspicua

`--| ,-- Microcachrys_tetragona

`--| ,-- Pherosphaera_hookeriana

`--| ,-- Acmopyle_pancheri

`--| ,-- Dacrydium_cupressinum

`--| ,-- Retrophyllum_rospigliosii

`----- Podocarpus_archboldii

Tree 9:

,-- Ginkgo_biloba

| ,-- Eathiestrobus_mackenziei

| | ,-- Cedrus_deodara

| ,--| | ,-- Pseudotsuga_menziesii

| | | | ,----- Larix_decidua

| | `--| ,--| ,-- Cathaya_argyrophylla

| | | | `--| ,-- Pinus_contorta

|--| | | | `----- Picea_abies

| | `--| ,-- Keteleeria_davidiana

| | | ,----- Abies_lasiocarpa

| | | | ,-- Pseudolarix_amabilis

| | `--| | ,-- Tsuga_canadensis

| | `--| ,----- Nothotsuga_longibracteata

`--|	`--| ,-- Schizolepidopsis_ediae

| `--| ,-- Schizolepidopsis_daohugouensis

| `----- Schizolepidopsis_canicularis

| ,-- Mataia_podocarpoides

| | ,-- Araucaria_mirabilis

| | ,----- Araucaria_heterophylla

| | ,--| ,-- Wollemia_nobilis

| | | `----- Agathis_australis

| | | ,-- Lagarostrobos_franklinii

| | ,--| ,----- Prumnopitys_taxifolia

`--| | | ,--| ,-- Lepidothamnus_laxifolius

| | | | `----- Phyllocladus_aspleniifolius

| | `--| ,-- Saxegothaea_conspicua

| | `--| ,-- Microcachrys_tetragona

| | `--| ,-- Pherosphaera_hookeriana

| | `--| ,-- Acmopyle_pancheri

| | `--| ,-- Dacrydium_cupressinum

`--|	`--| ,-- Retrophyllum_rospigliosii

| `----- Podocarpus_archboldii

| ,-- Pararaucaria_patagonica

| | ,-- **New_total_plant_from_Arkansas**

| ,--| ,----- Alvinia_bohemica_plant

| | `--| ,-- Himeriella_muensteri

| | `--| ,-- Kachaikestrobus_acuminatus

| | `----- Tomaxellia_biforme

`--| ,-- Sciadopitys_verticillata

| | ,-- Cunninghamia_lanceolata

| | ,--| ,-- Taiwania_cryptomerioides

`--| | `--| ,-- Athrotaxis_selaginoides

| | `--| ,-- Sequoia_sempervirens

| | `--| ,-- Cryptomeria_japonica

`--|	`--| ,-- Cupressus_funebris

| `----- Callitris_muelleri

| ,-- Telemachus_aequatus

| | ,-- Nothodacrium_warrenii

`--| ,----- Rissikia_media

| | ,-- Voltzia_hexagona

`--| |	,-- Emporia_lockardii

`--| ,----- Hanskerpia_hamiltonensis

`--| ,-- Cordaixylon_dumusum

`----- Taxus_wallichiana

Tree 10:

,-- Ginkgo_biloba

| ,-- Cordaixylon_dumusum

|--| | ,-- Emporia_lockardii

| | ,----- Hanskerpia_hamiltonensis

`--| | ,-- Voltzia_hexagona

| | | ,-- Nothodacrium_warrenii

`--| | ,----- Rissikia_media

| | | ,-- Pararaucaria_patagonica

`--| |	,--| ,-- **New_total_plant_from_Arkansas**

| | | `----- Alvinia_bohemica_plant

| | ,--| ,-- Himeriella_muensteri

`--| | `--| ,-- Kachaikestrobus_acuminatus

| | `----- Tomaxellia_biforme

| | ,-- Telemachus_aequatus

| | | ,-- Eathiestrobus_mackenziei

`--| |	| ,-- Cedrus_deodara

| | ,--| | ,-- Pseudotsuga_menziesii

| | | | | ,----- Larix_decidua

| | | `--| ,--| ,-- Cathaya_argyrophylla

| | | | | `--| ,-- Pinus_contorta

`--| |	| |	`----- Picea_abies

| | `--| ,-- Mataia_podocarpoides

| | | ,--| ,-- Keteleeria_davidiana

| | | | `----- Abies_lasiocarpa

| | `--| ,-- Pseudolarix_amabilis

| | | | ,-- Tsuga_canadensis

| | `--| ,----- Nothotsuga_longibracteata

`--|	`--| ,-- Schizolepidopsis_ediae

| `--| ,-- Schizolepidopsis_daohugouensis

| `----- Schizolepidopsis_canicularis

| ,-- Sciadopitys_verticillata

| ,--| ,-- Taxus_wallichiana

| | `--| ,-- Cunninghamia_lanceolata

| | `--| ,-- Taiwania_cryptomerioides

| | `--| ,-- Athrotaxis_selaginoides

| | `--| ,-- Sequoia_sempervirens

| | `--| ,-- Cryptomeria_japonica

| | `--| ,-- Cupressus_funebris

`--|	`----- Callitris_muelleri

| ,-- Araucaria_mirabilis

| ,----- Araucaria_heterophylla

| ,--| ,-- Wollemia_nobilis

| | `----- Agathis_australis

| | ,-- Lagarostrobos_franklinii

`--|	,----- Prumnopitys_taxifolia

| ,--| ,-- Lepidothamnus_laxifolius

| | `----- Phyllocladus_aspleniifolius

`--| ,-- Saxegothaea_conspicua

`--| ,-- Microcachrys_tetragona

`--| ,-- Pherosphaera_hookeriana

`--| ,-- Acmopyle_pancheri

`--| ,-- Dacrydium_cupressinum

`--| ,-- Retrophyllum_rospigliosii

`----- Podocarpus_archboldii

Tree 11:

,-- Ginkgo_biloba

| ,-- Mataia_podocarpoides

| | ,-- Cedrus_deodara

| | | ,-- Keteleeria_davidiana

| | | ,----- Abies_lasiocarpa

|--| | ,--| ,--| ,-- Pseudolarix_amabilis

| | | | | `--| ,-- Tsuga_canadensis

| | | | | `----- Nothotsuga_longibracteata

| | | `--| ,-- Pseudotsuga_menziesii

| | | | ,----- Larix_decidua

`--| |	| | ,-- Cathaya_argyrophylla

| | `--| | ,-- Eathiestrobus_mackenziei

| | `--| ,----- Pinus_contorta

| | `--| ,-- Picea_abies

| | `--| ,-- Schizolepidopsis_ediae

| | `--| ,-- Schizolepidopsis_daohugouensis

| | `----- Schizolepidopsis_canicularis

| | ,-- Araucaria_mirabilis

`--|	,----- Araucaria_heterophylla

| ,--| ,-- Wollemia_nobilis

| | `----- Agathis_australis

| | ,-- Lagarostrobos_franklinii

| ,--| ,----- Prumnopitys_taxifolia

| | | ,--| ,-- Lepidothamnus_laxifolius

| | | | `----- Phyllocladus_aspleniifolius

| | `--| ,-- Saxegothaea_conspicua

| | `--| ,-- Microcachrys_tetragona

| | `--| ,-- Pherosphaera_hookeriana

| | `--| ,-- Acmopyle_pancheri

| | `--| ,-- Dacrydium_cupressinum

`--|	`--| ,-- Retrophyllum_rospigliosii

| `----- Podocarpus_archboldii

| ,-- Pararaucaria_patagonica

| | ,-- **New_total_plant_from_Arkansas**

| ,--| ,----- Alvinia_bohemica_plant

| | `--| ,-- Himeriella_muensteri

| | `--| ,-- Kachaikestrobus_acuminatus

| | `----- Tomaxellia_biforme

`--| ,-- Sciadopitys_verticillata

| | ,-- Cunninghamia_lanceolata

| | ,--| ,-- Taiwania_cryptomerioides

`--| | `--| ,-- Athrotaxis_selaginoides

| | `--| ,-- Sequoia_sempervirens

| | `--| ,-- Cryptomeria_japonica

`--|	`--| ,-- Cupressus_funebris

| `----- Callitris_muelleri

| ,-- Telemachus_aequatus

| | ,-- Nothodacrium_warrenii

`--| ,----- Rissikia_media

| | ,-- Voltzia_hexagona

`--| |	,-- Emporia_lockardii

`--| ,----- Hanskerpia_hamiltonensis

`--| ,-- Cordaixylon_dumusum

`----- Taxus_wallichiana

Tree 12:

,-- Ginkgo_biloba

| ,-- Cordaixylon_dumusum

|--| | ,-- Emporia_lockardii

| | ,----- Hanskerpia_hamiltonensis

`--| | ,-- Voltzia_hexagona

| | | ,-- Nothodacrium_warrenii

`--| | ,----- Rissikia_media

| | | ,-- Telemachus_aequatus

`--| | |	,-- Schizolepidopsis_ediae

| | | | ,-- Schizolepidopsis_daohugouensis

| | | ,--| ,----- Schizolepidopsis_canicularis

`--| | | | | ,-- Cedrus_deodara

| | | `--| | ,-- Mataia_podocarpoides

| | | | | | ,-- Keteleeria_davidiana

| | | `--| ,--| ,----- Abies_lasiocarpa

`--| |	| | `--| ,-- Pseudolarix_amabilis

| | | | `--| ,-- Tsuga_canadensis

| | `--| `----- Nothotsuga_longibracteata

| | | ,-- Pseudotsuga_menziesii

| | | ,----- Larix_decidua

| | `--| ,-- Cathaya_argyrophylla

| | `--| ,-- Picea_abies

`--|	`--| ,-- Eathiestrobus_mackenziei

| `----- Pinus_contorta

| ,-- Sciadopitys_verticillata

| ,--| ,-- Taxus_wallichiana

| | `--| ,-- Cunninghamia_lanceolata

| | `--| ,-- Taiwania_cryptomerioides

| | `--| ,-- Athrotaxis_selaginoides

| | `--| ,-- Sequoia_sempervirens

| | `--| ,-- Cryptomeria_japonica

| | `--| ,-- Cupressus_funebris

| | `----- Callitris_muelleri

`--|	,-- Pararaucaria_patagonica

| | ,-- **New_total_plant_from_Arkansas**

| ,--| ,----- Alvinia_bohemica_plant

| | `--| ,-- Himeriella_muensteri

| | `--| ,-- Kachaikestrobus_acuminatus

| | `----- Tomaxellia_biforme

| | ,-- Araucaria_mirabilis

`--|	,----- Araucaria_heterophylla

| ,--| ,-- Wollemia_nobilis

| | `----- Agathis_australis

| | ,-- Lagarostrobos_franklinii

`--|	,----- Prumnopitys_taxifolia

| ,--| ,-- Lepidothamnus_laxifolius

| | `----- Phyllocladus_aspleniifolius

`--| ,-- Saxegothaea_conspicua

`--| ,-- Microcachrys_tetragona

`--| ,-- Pherosphaera_hookeriana

`--| ,-- Acmopyle_pancheri

`--| ,-- Dacrydium_cupressinum

`--| ,-- Retrophyllum_rospigliosii

`----- Podocarpus_archboldii

Tree 13:

,-- Ginkgo_biloba

| ,-- Cordaixylon_dumusum

|--| | ,-- Emporia_lockardii

| | ,----- Hanskerpia_hamiltonensis

`--| | ,-- Voltzia_hexagona

| | | ,-- Nothodacrium_warrenii

`--| | ,----- Rissikia_media

| | | ,-- Telemachus_aequatus

`--| | |	,-- Schizolepidopsis_daohugouensis

| | | ,----- Schizolepidopsis_canicularis

| | | | ,-- Schizolepidopsis_ediae

`--| | ,--| | ,-- Cedrus_deodara

| | | | | | ,-- Mataia_podocarpoides

| | | `--| | ,--| ,-- Keteleeria_davidiana

| | | | | | `----- Abies_lasiocarpa

`--| |	`--| ,--| ,-- Pseudolarix_amabilis

| | | | `--| ,-- Tsuga_canadensis

| | | | `----- Nothotsuga_longibracteata

| | `--| ,-- Pseudotsuga_menziesii

| | | ,----- Larix_decidua

| | `--| ,-- Cathaya_argyrophylla

| | `--| ,-- Pinus_contorta

`--|	`--| ,-- Eathiestrobus_mackenziei

| `----- Picea_abies

| ,-- Sciadopitys_verticillata

| ,--| ,-- Taxus_wallichiana

| | `--| ,-- Cunninghamia_lanceolata

| | `--| ,-- Taiwania_cryptomerioides

| | `--| ,-- Athrotaxis_selaginoides

| | `--| ,-- Sequoia_sempervirens

| | `--| ,-- Cryptomeria_japonica

| | `--| ,-- Cupressus_funebris

| | `----- Callitris_muelleri

`--|	,-- Pararaucaria_patagonica

| | ,-- **New_total_plant_from_Arkansas**

| ,--| ,----- Alvinia_bohemica_plant

| | `--| ,-- Himeriella_muensteri

| | `--| ,-- Kachaikestrobus_acuminatus

| | `----- Tomaxellia_biforme

| | ,-- Araucaria_mirabilis

`--|	,----- Araucaria_heterophylla

| ,--| ,-- Wollemia_nobilis

| | `----- Agathis_australis

| | ,-- Lagarostrobos_franklinii

`--|	,----- Prumnopitys_taxifolia

| ,--| ,-- Lepidothamnus_laxifolius

| | `----- Phyllocladus_aspleniifolius

`--| ,-- Saxegothaea_conspicua

`--| ,-- Microcachrys_tetragona

`--| ,-- Pherosphaera_hookeriana

`--| ,-- Acmopyle_pancheri

`--| ,-- Dacrydium_cupressinum

`--| ,-- Retrophyllum_rospigliosii

`----- Podocarpus_archboldii

Tree 14:

,-- Ginkgo_biloba

| ,-- Cordaixylon_dumusum

|--| | ,-- Emporia_lockardii

| | ,----- Hanskerpia_hamiltonensis

`--| | ,-- Voltzia_hexagona

| | | ,-- Nothodacrium_warrenii

`--| | ,----- Rissikia_media

| | | ,-- Pararaucaria_patagonica

`--| |	,--| ,-- **New_total_plant_from_Arkansas**

| | | `----- Alvinia_bohemica_plant

| | ,--| ,-- Himeriella_muensteri

`--| | `--| ,-- Kachaikestrobus_acuminatus

| | `----- Tomaxellia_biforme

| | ,-- Telemachus_aequatus

| | | ,-- Eathiestrobus_mackenziei

`--| |	| ,-- Cedrus_deodara

| | ,--| | ,-- Pseudotsuga_menziesii

| | | | | ,----- Larix_decidua

| | | `--| ,--| ,-- Cathaya_argyrophylla

| | | | | `--| ,-- Pinus_contorta

`--| |	| |	`----- Picea_abies

| | `--| ,-- Keteleeria_davidiana

| | | ,----- Abies_lasiocarpa

| | | | ,-- Pseudolarix_amabilis

| | `--| | ,-- Mataia_podocarpoides

| | `--| ,--| ,-- Tsuga_canadensis

| | | | `----- Nothotsuga_longibracteata

`--|	`--| ,-- Schizolepidopsis_ediae

| `--| ,-- Schizolepidopsis_daohugouensis

| `----- Schizolepidopsis_canicularis

| ,-- Sciadopitys_verticillata

| ,--| ,-- Taxus_wallichiana

| | `--| ,-- Cunninghamia_lanceolata

| | `--| ,-- Taiwania_cryptomerioides

| | `--| ,-- Athrotaxis_selaginoides

| | `--| ,-- Sequoia_sempervirens

| | `--| ,-- Cryptomeria_japonica

| | `--| ,-- Cupressus_funebris

`--|	`----- Callitris_muelleri

| ,-- Araucaria_mirabilis

| ,----- Araucaria_heterophylla

| ,--| ,-- Wollemia_nobilis

| | `----- Agathis_australis

| | ,-- Lagarostrobos_franklinii

`--|	,----- Prumnopitys_taxifolia

| ,--| ,-- Lepidothamnus_laxifolius

| | `----- Phyllocladus_aspleniifolius

`--| ,-- Saxegothaea_conspicua

| | ,-- Pherosphaera_hookeriana

`--| ,----- Microcachrys_tetragona

`--| ,-- Acmopyle_pancheri

`--| ,-- Dacrydium_cupressinum

`--| ,-- Retrophyllum_rospigliosii

`----- Podocarpus_archboldii

Tree 15:

,-- Ginkgo_biloba

| ,-- Cordaixylon_dumusum

|--| | ,-- Emporia_lockardii

| | ,----- Hanskerpia_hamiltonensis

`--| | ,-- Voltzia_hexagona

| | | ,-- Nothodacrium_warrenii

`--| | ,----- Rissikia_media

| | | ,-- Pararaucaria_patagonica

`--| |	|	,-- **New_total_plant_from_Arkansas**

| | ,--| ,----- Alvinia_bohemica_plant

| | | `--| ,-- Himeriella_muensteri

`--| |	`--| ,-- Kachaikestrobus_acuminatus

| | `----- Tomaxellia_biforme

| | ,-- Telemachus_aequatus

| | | ,-- Eathiestrobus_mackenziei

`--| |	| ,-- Cedrus_deodara

| | ,--| | ,-- Pseudotsuga_menziesii

| | | | | ,----- Larix_decidua

| | | `--| ,--| ,-- Cathaya_argyrophylla

| | | | | `--| ,-- Pinus_contorta

`--| |	| |	`----- Picea_abies

| | `--| ,-- Keteleeria_davidiana

| | | ,--| ,-- Mataia_podocarpoides

| | | | `----- Abies_lasiocarpa

| | `--| ,-- Pseudolarix_amabilis

| | | | ,-- Tsuga_canadensis

| | `--| ,----- Nothotsuga_longibracteata

`--|	`--| ,-- Schizolepidopsis_ediae

| `--| ,-- Schizolepidopsis_daohugouensis

| `----- Schizolepidopsis_canicularis

| ,-- Sciadopitys_verticillata

| ,--| ,-- Taxus_wallichiana

| | `--| ,-- Cunninghamia_lanceolata

| | `--| ,-- Taiwania_cryptomerioides

| | `--| ,-- Athrotaxis_selaginoides

| | `--| ,-- Sequoia_sempervirens

| | `--| ,-- Cryptomeria_japonica

| | `--| ,-- Cupressus_funebris

`--|	`----- Callitris_muelleri

| ,-- Araucaria_mirabilis

| ,----- Araucaria_heterophylla

| ,--| ,-- Wollemia_nobilis

| | `----- Agathis_australis

| | ,-- Lagarostrobos_franklinii

`--|	,----- Prumnopitys_taxifolia

| ,--| ,-- Lepidothamnus_laxifolius

| | `----- Phyllocladus_aspleniifolius

`--| ,-- Saxegothaea_conspicua

| | ,-- Pherosphaera_hookeriana

`--| ,----- Microcachrys_tetragona

`--| ,-- Acmopyle_pancheri

`--| ,-- Dacrydium_cupressinum

`--| ,-- Retrophyllum_rospigliosii

`----- Podocarpus_archboldii

Tree 16:

,-- Ginkgo_biloba

| ,-- Cordaixylon_dumusum

|--| | ,-- Emporia_lockardii

| | ,----- Hanskerpia_hamiltonensis

`--| | ,-- Voltzia_hexagona

| | | ,-- Nothodacrium_warrenii

`--| | ,----- Rissikia_media

| | | ,-- Pararaucaria_patagonica

`--| |	|	,-- **New_total_plant_from_Arkansas**

| | ,--| ,----- Alvinia_bohemica_plant

| | | `--| ,-- Himeriella_muensteri

`--| |	`--| ,-- Kachaikestrobus_acuminatus

| | `----- Tomaxellia_biforme

| | ,-- Telemachus_aequatus

| | | ,-- Eathiestrobus_mackenziei

`--| |	| ,-- Cedrus_deodara

| | | | ,-- Keteleeria_davidiana

| | ,--| | ,----- Abies_lasiocarpa

| | | | | | ,-- Mataia_podocarpoides

| | | `--| ,--| ,----- Pseudolarix_amabilis

| | | | | `--| ,-- Tsuga_canadensis

`--| |	| |	`----- Nothotsuga_longibracteata

| | `--| ,-- Pseudotsuga_menziesii

| | | ,----- Larix_decidua

| | `--| ,-- Cathaya_argyrophylla

| | `--| ,-- Pinus_contorta

| | `--| ,-- Picea_abies

| | `--| ,-- Schizolepidopsis_ediae

`--|	`--| ,-- Schizolepidopsis_daohugouensis

| `----- Schizolepidopsis_canicularis

| ,-- Sciadopitys_verticillata

| ,--| ,-- Taxus_wallichiana

| | `--| ,-- Cunninghamia_lanceolata

| | `--| ,-- Taiwania_cryptomerioides

| | `--| ,-- Athrotaxis_selaginoides

| | `--| ,-- Sequoia_sempervirens

| | `--| ,-- Cryptomeria_japonica

| | `--| ,-- Cupressus_funebris

`--|	`----- Callitris_muelleri

| ,-- Araucaria_mirabilis

| ,----- Araucaria_heterophylla

| ,--| ,-- Wollemia_nobilis

| | `----- Agathis_australis

| | ,-- Lagarostrobos_franklinii

`--|	,----- Prumnopitys_taxifolia

| ,--| ,-- Lepidothamnus_laxifolius

| | `----- Phyllocladus_aspleniifolius

`--| ,-- Saxegothaea_conspicua

| | ,-- Pherosphaera_hookeriana

`--| ,----- Microcachrys_tetragona

`--| ,-- Acmopyle_pancheri

`--| ,-- Dacrydium_cupressinum

`--| ,-- Retrophyllum_rospigliosii

`----- Podocarpus_archboldii

Tree 17:

,-- Ginkgo_biloba

| ,-- Cordaixylon_dumusum

|--| | ,-- Emporia_lockardii

| | ,----- Hanskerpia_hamiltonensis

`--| | ,-- Voltzia_hexagona

| | | ,-- Nothodacrium_warrenii

`--| | ,----- Rissikia_media

| | | ,-- Pararaucaria_patagonica

`--| |	|	,-- **New_total_plant_from_Arkansas**

| | ,--| ,----- Alvinia_bohemica_plant

| | | `--| ,-- Himeriella_muensteri

`--| |	`--| ,-- Kachaikestrobus_acuminatus

| | `----- Tomaxellia_biforme

| | ,-- Telemachus_aequatus

| | | ,-- Eathiestrobus_mackenziei

`--| |	| ,-- Cedrus_deodara

| | ,--| | ,-- Mataia_podocarpoides

| | | `--| | ,-- Keteleeria_davidiana

| | | | | ,----- Abies_lasiocarpa

| | | `--| ,--| ,-- Pseudolarix_amabilis

`--| |	| | `--| ,-- Tsuga_canadensis

| | | | `----- Nothotsuga_longibracteata

| | `--| ,-- Pseudotsuga_menziesii

| | | ,----- Larix_decidua

| | `--| ,-- Cathaya_argyrophylla

| | `--| ,-- Pinus_contorta

| | `--| ,-- Picea_abies

`--|	`--| ,-- Schizolepidopsis_ediae

| `--| ,-- Schizolepidopsis_daohugouensis

| `----- Schizolepidopsis_canicularis

| ,-- Sciadopitys_verticillata

| ,--| ,-- Taxus_wallichiana

| | `--| ,-- Cunninghamia_lanceolata

| | `--| ,-- Taiwania_cryptomerioides

| | `--| ,-- Athrotaxis_selaginoides

| | `--| ,-- Sequoia_sempervirens

| | `--| ,-- Cryptomeria_japonica

| | `--| ,-- Cupressus_funebris

`--|	`----- Callitris_muelleri

| ,-- Araucaria_mirabilis

| ,----- Araucaria_heterophylla

| ,--| ,-- Wollemia_nobilis

| | `----- Agathis_australis

| | ,-- Lagarostrobos_franklinii

`--|	,----- Prumnopitys_taxifolia

| ,--| ,-- Lepidothamnus_laxifolius

| | `----- Phyllocladus_aspleniifolius

`--| ,-- Saxegothaea_conspicua

| | ,-- Pherosphaera_hookeriana

`--| ,----- Microcachrys_tetragona

`--| ,-- Acmopyle_pancheri

`--| ,-- Dacrydium_cupressinum

`--| ,-- Retrophyllum_rospigliosii

`----- Podocarpus_archboldii

Tree 18:

,-- Ginkgo_biloba

| ,-- Cordaixylon_dumusum

|--| | ,-- Emporia_lockardii

| | ,----- Hanskerpia_hamiltonensis

`--| | ,-- Voltzia_hexagona

| | | ,-- Nothodacrium_warrenii

`--| | ,----- Rissikia_media

| | | ,-- **New_total_plant_from_Arkansas**

`--| |	,--| ,-- Alvinia_bohemica_plant

| | | `----- Pararaucaria_patagonica

| | ,--| ,-- Himeriella_muensteri

`--| | `--| ,-- Kachaikestrobus_acuminatus

| | `----- Tomaxellia_biforme

| | ,-- Telemachus_aequatus

| | | ,-- Eathiestrobus_mackenziei

`--| |	| ,-- Cedrus_deodara

| | ,--| | ,-- Mataia_podocarpoides

| | | `--| | ,-- Keteleeria_davidiana

| | | | | ,----- Abies_lasiocarpa

| | | `--| ,--| ,-- Pseudolarix_amabilis

`--| |	| | `--| ,-- Tsuga_canadensis

| | | | `----- Nothotsuga_longibracteata

| | `--| ,-- Pseudotsuga_menziesii

| | | ,----- Larix_decidua

| | `--| ,-- Cathaya_argyrophylla

| | `--| ,-- Pinus_contorta

| | `--| ,-- Picea_abies

`--|	`--| ,-- Schizolepidopsis_ediae

| `--| ,-- Schizolepidopsis_daohugouensis

| `----- Schizolepidopsis_canicularis

| ,-- Sciadopitys_verticillata

| ,--| ,-- Taxus_wallichiana

| | `--| ,-- Cunninghamia_lanceolata

| | `--| ,-- Taiwania_cryptomerioides

| | `--| ,-- Athrotaxis_selaginoides

| | `--| ,-- Sequoia_sempervirens

| | `--| ,-- Cryptomeria_japonica

| | `--| ,-- Cupressus_funebris

`--|	`----- Callitris_muelleri

| ,-- Araucaria_mirabilis

| ,----- Araucaria_heterophylla

| ,--| ,-- Wollemia_nobilis

| | `----- Agathis_australis

| | ,-- Lagarostrobos_franklinii

`--|	,----- Prumnopitys_taxifolia

| ,--| ,-- Lepidothamnus_laxifolius

| | `----- Phyllocladus_aspleniifolius

`--| ,-- Saxegothaea_conspicua

`--| ,-- Microcachrys_tetragona

`--| ,-- Pherosphaera_hookeriana

`--| ,-- Acmopyle_pancheri

`--| ,-- Dacrydium_cupressinum

`--| ,-- Retrophyllum_rospigliosii

`----- Podocarpus_archboldii

Tree 19:

,-- Ginkgo_biloba

| ,-- Cordaixylon_dumusum

|--| | ,-- Emporia_lockardii

| | ,----- Hanskerpia_hamiltonensis

`--| | ,-- Voltzia_hexagona

| | | ,-- Nothodacrium_warrenii

`--| | ,----- Rissikia_media

| | | ,-- Pararaucaria_patagonica

`--| |	|	,-- **New_total_plant_from_Arkansas**

| | ,--| ,----- Alvinia_bohemica_plant

| | | `--| ,-- Himeriella_muensteri

`--| |	`--| ,-- Kachaikestrobus_acuminatus

| | `----- Tomaxellia_biforme

| | ,-- Telemachus_aequatus

| | | ,-- Eathiestrobus_mackenziei

`--| |	| ,-- Cedrus_deodara

| | ,--| | ,-- Mataia_podocarpoides

| | | `--| | ,-- Keteleeria_davidiana

| | | | | ,----- Abies_lasiocarpa

| | | `--| ,--| ,-- Pseudolarix_amabilis

`--| |	| | `--| ,-- Tsuga_canadensis

| | | | `----- Nothotsuga_longibracteata

| | `--| ,-- Pseudotsuga_menziesii

| | | ,----- Larix_decidua

| | `--| ,-- Cathaya_argyrophylla

| | `--| ,-- Pinus_contorta

| | `--| ,-- Picea_abies

`--|	`--| ,-- Schizolepidopsis_ediae

| `--| ,-- Schizolepidopsis_daohugouensis

| `----- Schizolepidopsis_canicularis

| ,-- Sciadopitys_verticillata

| ,--| ,-- Taxus_wallichiana

| | `--| ,-- Cunninghamia_lanceolata

| | `--| ,-- Taiwania_cryptomerioides

| | `--| ,-- Athrotaxis_selaginoides

| | `--| ,-- Sequoia_sempervirens

| | `--| ,-- Cryptomeria_japonica

| | `--| ,-- Cupressus_funebris

`--|	`----- Callitris_muelleri

| ,-- Araucaria_mirabilis

| ,----- Araucaria_heterophylla

| ,--| ,-- Wollemia_nobilis

| | `----- Agathis_australis

| | ,-- Lagarostrobos_franklinii

`--|	,----- Prumnopitys_taxifolia

| ,--| ,-- Lepidothamnus_laxifolius

| | `----- Phyllocladus_aspleniifolius

`--| ,-- Saxegothaea_conspicua

`--| ,-- Microcachrys_tetragona

`--| ,-- Pherosphaera_hookeriana

`--| ,-- Acmopyle_pancheri

`--| ,-- Dacrydium_cupressinum

`--| ,-- Retrophyllum_rospigliosii

`----- Podocarpus_archboldii

Tree 20:

,-- Ginkgo_biloba

| ,-- Cordaixylon_dumusum

|--| | ,-- Emporia_lockardii

| | ,----- Hanskerpia_hamiltonensis

`--| | ,-- Voltzia_hexagona

| | | ,-- Nothodacrium_warrenii

`--| | ,----- Rissikia_media

| | | ,-- **New_total_plant_from_Arkansas**

`--| |	,--| ,-- Alvinia_bohemica_plant

| | | `----- Pararaucaria_patagonica

| | ,--| ,-- Himeriella_muensteri

`--| | `--| ,-- Kachaikestrobus_acuminatus

| | `----- Tomaxellia_biforme

| | ,-- Telemachus_aequatus

| | | ,-- Eathiestrobus_mackenziei

`--| |	| ,-- Cedrus_deodara

| | ,--| | ,-- Mataia_podocarpoides

| | | | | | ,-- Keteleeria_davidiana

| | | `--| ,--| ,----- Abies_lasiocarpa

| | | | | `--| ,-- Pseudolarix_amabilis

`--| |	| |	`--| ,-- Tsuga_canadensis

| | `--| `----- Nothotsuga_longibracteata

| | | ,-- Pseudotsuga_menziesii

| | | ,----- Larix_decidua

| | `--| ,-- Cathaya_argyrophylla

| | `--| ,-- Pinus_contorta

| | `--| ,-- Picea_abies

`--|	`--| ,-- Schizolepidopsis_ediae

| `--| ,-- Schizolepidopsis_daohugouensis

| `----- Schizolepidopsis_canicularis

| ,-- Sciadopitys_verticillata

| ,--| ,-- Taxus_wallichiana

| | `--| ,-- Cunninghamia_lanceolata

| | `--| ,-- Taiwania_cryptomerioides

| | `--| ,-- Athrotaxis_selaginoides

| | `--| ,-- Sequoia_sempervirens

| | `--| ,-- Cryptomeria_japonica

| | `--| ,-- Cupressus_funebris

`--|	`----- Callitris_muelleri

| ,-- Araucaria_mirabilis

| ,----- Araucaria_heterophylla

| ,--| ,-- Wollemia_nobilis

| | `----- Agathis_australis

| | ,-- Lagarostrobos_franklinii

`--|	,----- Prumnopitys_taxifolia

| ,--| ,-- Lepidothamnus_laxifolius

| | `----- Phyllocladus_aspleniifolius

`--| ,-- Saxegothaea_conspicua

| | ,-- Pherosphaera_hookeriana

`--| ,----- Microcachrys_tetragona

`--| ,-- Acmopyle_pancheri

`--| ,-- Dacrydium_cupressinum

`--| ,-- Retrophyllum_rospigliosii

`----- Podocarpus_archboldii

Tree 21:

,-- Ginkgo_biloba

| ,-- Cordaixylon_dumusum

|--| | ,-- Emporia_lockardii

| | ,----- Hanskerpia_hamiltonensis

`--| | ,-- Voltzia_hexagona

| | | ,-- Nothodacrium_warrenii

`--| | ,----- Rissikia_media

| | | ,-- Pararaucaria_patagonica

`--| |	,--| ,-- **New_total_plant_from_Arkansas**

| | | `----- Alvinia_bohemica_plant

| | ,--| ,-- Himeriella_muensteri

`--| | `--| ,-- Kachaikestrobus_acuminatus

| | `----- Tomaxellia_biforme

| | ,-- Telemachus_aequatus

| | | ,-- Eathiestrobus_mackenziei

`--| |	| ,-- Cedrus_deodara

| | ,--| | ,-- Mataia_podocarpoides

| | | | | | ,-- Keteleeria_davidiana

| | | `--| ,--| ,----- Abies_lasiocarpa

| | | | | `--| ,-- Pseudolarix_amabilis

`--| |	| |	`--| ,-- Tsuga_canadensis

| | `--| `----- Nothotsuga_longibracteata

| | | ,-- Pseudotsuga_menziesii

| | | ,----- Larix_decidua

| | `--| ,-- Cathaya_argyrophylla

| | `--| ,-- Pinus_contorta

| | `--| ,-- Picea_abies

`--|	`--| ,-- Schizolepidopsis_ediae

| `--| ,-- Schizolepidopsis_daohugouensis

| `----- Schizolepidopsis_canicularis

| ,-- Sciadopitys_verticillata

| ,--| ,-- Taxus_wallichiana

| | `--| ,-- Cunninghamia_lanceolata

| | `--| ,-- Taiwania_cryptomerioides

| | `--| ,-- Athrotaxis_selaginoides

| | `--| ,-- Sequoia_sempervirens

| | `--| ,-- Cryptomeria_japonica

| | `--| ,-- Cupressus_funebris

`--|	`----- Callitris_muelleri

| ,-- Araucaria_mirabilis

| ,----- Araucaria_heterophylla

| ,--| ,-- Wollemia_nobilis

| | `----- Agathis_australis

| | ,-- Lagarostrobos_franklinii

`--|	,----- Prumnopitys_taxifolia

| ,--| ,-- Lepidothamnus_laxifolius

| | `----- Phyllocladus_aspleniifolius

`--| ,-- Saxegothaea_conspicua

`--| ,-- Microcachrys_tetragona

`--| ,-- Pherosphaera_hookeriana

`--| ,-- Acmopyle_pancheri

`--| ,-- Dacrydium_cupressinum

`--| ,-- Retrophyllum_rospigliosii

`----- Podocarpus_archboldii

Tree 22:

,-- Ginkgo_biloba

| ,-- Cordaixylon_dumusum

|--| | ,-- Emporia_lockardii

`--| | ,-- Hanskerpia_hamiltonensis

`--| | ,-- Voltzia_hexagona

`--| |	,-- Nothodacrium_warrenii

| | ,----- Rissikia_media

`--| | ,-- Telemachus_aequatus

| | | ,-- Schizolepidopsis_ediae

| | | | ,-- Schizolepidopsis_daohugouensis

`--| | ,--| ,----- Schizolepidopsis_canicularis

| | | | | ,-- Cedrus_deodara

| | | `--| | ,-- Mataia_podocarpoides

| | | | | | ,-- Keteleeria_davidiana

`--| |	`--| ,--| ,----- Abies_lasiocarpa

| | | | `--| ,-- Pseudolarix_amabilis

| | | | `--| ,-- Tsuga_canadensis

| | `--| `----- Nothotsuga_longibracteata

| | | ,-- Pseudotsuga_menziesii

| | | ,----- Larix_decidua

`--|	`--| ,-- Cathaya_argyrophylla

| `--| ,-- Pinus_contorta

| `--| ,-- Eathiestrobus_mackenziei

| `----- Picea_abies

| ,-- **New_total_plant_from_Arkansas**

| ,----- Alvinia_bohemica_plant

| ,--| ,-- Himeriella_muensteri

| | `--| ,-- Kachaikestrobus_acuminatus

| | `----- Tomaxellia_biforme

| | ,-- Pararaucaria_patagonica

`--| |	,-- Sciadopitys_verticillata

| | ,--| ,-- Taxus_wallichiana

| | | `--| ,-- Cunninghamia_lanceolata

| | | `--| ,-- Taiwania_cryptomerioides

`--| |	`--| ,-- Athrotaxis_selaginoides

| | `--| ,-- Sequoia_sempervirens

| | `--| ,-- Cryptomeria_japonica

| | `--| ,-- Cupressus_funebris

`--|	`----- Callitris_muelleri

| ,-- Araucaria_mirabilis

| ,----- Araucaria_heterophylla

| ,--| ,-- Wollemia_nobilis

| | `----- Agathis_australis

| | ,-- Lagarostrobos_franklinii

`--|	,----- Prumnopitys_taxifolia

| ,--| ,-- Lepidothamnus_laxifolius

| | `----- Phyllocladus_aspleniifolius

`--| ,-- Saxegothaea_conspicua

`--| ,-- Microcachrys_tetragona

`--| ,-- Pherosphaera_hookeriana

`--| ,-- Acmopyle_pancheri

`--| ,-- Dacrydium_cupressinum

`--| ,-- Retrophyllum_rospigliosii

`----- Podocarpus_archboldii

Tree 23:

,-- Ginkgo_biloba

| ,-- Cordaixylon_dumusum

|--| | ,-- Emporia_lockardii

`--| ,----- Hanskerpia_hamiltonensis

| | ,-- Voltzia_hexagona

`--| |	,-- Nothodacrium_warrenii

| | ,----- Rissikia_media

`--| | ,-- Telemachus_aequatus

| | | ,-- Schizolepidopsis_ediae

| | | | ,-- Schizolepidopsis_daohugouensis

`--| | ,--| ,----- Schizolepidopsis_canicularis

| | | | | ,-- Cedrus_deodara

| | | `--| | ,-- Mataia_podocarpoides

| | | | | | ,-- Keteleeria_davidiana

`--| |	`--| ,--| ,----- Abies_lasiocarpa

| | | | `--| ,-- Pseudolarix_amabilis

| | | | `--| ,-- Tsuga_canadensis

| | `--| `----- Nothotsuga_longibracteata

| | | ,-- Pseudotsuga_menziesii

| | | ,----- Larix_decidua

`--|	`--| ,-- Cathaya_argyrophylla

| `--| ,-- Pinus_contorta

| `--| ,-- Eathiestrobus_mackenziei

| `----- Picea_abies

| ,-- **New_total_plant_from_Arkansas**

| ,----- Alvinia_bohemica_plant

| ,--| ,-- Himeriella_muensteri

| | `--| ,-- Kachaikestrobus_acuminatus

| | `----- Tomaxellia_biforme

| | ,-- Pararaucaria_patagonica

`--| |	,-- Sciadopitys_verticillata

| | ,--| ,-- Taxus_wallichiana

| | | `--| ,-- Cunninghamia_lanceolata

| | | `--| ,-- Taiwania_cryptomerioides

`--| |	`--| ,-- Athrotaxis_selaginoides

| | `--| ,-- Sequoia_sempervirens

| | `--| ,-- Cryptomeria_japonica

| | `--| ,-- Cupressus_funebris

`--|	`----- Callitris_muelleri

| ,-- Araucaria_mirabilis

| ,----- Araucaria_heterophylla

| ,--| ,-- Wollemia_nobilis

| | `----- Agathis_australis

| | ,-- Lagarostrobos_franklinii

`--|	,----- Prumnopitys_taxifolia

| ,--| ,-- Lepidothamnus_laxifolius

| | `----- Phyllocladus_aspleniifolius

`--| ,-- Saxegothaea_conspicua

`--| ,-- Microcachrys_tetragona

`--| ,-- Pherosphaera_hookeriana

`--| ,-- Acmopyle_pancheri

`--| ,-- Dacrydium_cupressinum

`--| ,-- Retrophyllum_rospigliosii

`----- Podocarpus_archboldii

Tree 24:

,-- Ginkgo_biloba

| ,-- Cordaixylon_dumusum

|--| | ,-- Emporia_lockardii

| | ,----- Hanskerpia_hamiltonensis

`--| | ,-- Voltzia_hexagona

| | | ,-- Nothodacrium_warrenii

`--| | ,----- Rissikia_media

| | | ,-- Pararaucaria_patagonica

`--| |	|	,-- **New_total_plant_from_Arkansas**

| | ,--| ,----- Alvinia_bohemica_plant

| | | `--| ,-- Himeriella_muensteri

`--| |	`--| ,-- Kachaikestrobus_acuminatus

| | `----- Tomaxellia_biforme

| | ,-- Telemachus_aequatus

| | | ,-- Eathiestrobus_mackenziei

`--| |	| ,-- Cedrus_deodara

| | ,--| | ,-- Mataia_podocarpoides

| | | | | | ,-- Keteleeria_davidiana

| | | `--| ,--| ,----- Abies_lasiocarpa

| | | | | `--| ,-- Pseudolarix_amabilis

`--| |	| |	`--| ,-- Tsuga_canadensis

| | `--| `----- Nothotsuga_longibracteata

| | | ,-- Pseudotsuga_menziesii

| | | ,----- Larix_decidua

| | `--| ,-- Cathaya_argyrophylla

| | `--| ,-- Pinus_contorta

| | `--| ,-- Picea_abies

`--|	`--| ,-- Schizolepidopsis_ediae

| `--| ,-- Schizolepidopsis_daohugouensis

| `----- Schizolepidopsis_canicularis

| ,-- Sciadopitys_verticillata

| ,--| ,-- Taxus_wallichiana

| | `--| ,-- Cunninghamia_lanceolata

| | `--| ,-- Taiwania_cryptomerioides

| | `--| ,-- Athrotaxis_selaginoides

| | `--| ,-- Sequoia_sempervirens

| | `--| ,-- Cryptomeria_japonica

| | `--| ,-- Cupressus_funebris

`--|	`----- Callitris_muelleri

| ,-- Araucaria_mirabilis

| ,----- Araucaria_heterophylla

| ,--| ,-- Wollemia_nobilis

| | `----- Agathis_australis

| | ,-- Lagarostrobos_franklinii

`--|	,----- Prumnopitys_taxifolia

| ,--| ,-- Lepidothamnus_laxifolius

| | `----- Phyllocladus_aspleniifolius

`--| ,-- Saxegothaea_conspicua

`--| ,-- Microcachrys_tetragona

`--| ,-- Pherosphaera_hookeriana

`--| ,-- Acmopyle_pancheri

`--| ,-- Dacrydium_cupressinum

`--| ,-- Retrophyllum_rospigliosii

`----- Podocarpus_archboldii

Tree 25:

,-- Ginkgo_biloba

| ,-- Cordaixylon_dumusum

|--| | ,-- Emporia_lockardii

| | ,----- Hanskerpia_hamiltonensis

`--| | ,-- Voltzia_hexagona

| | | ,-- Nothodacrium_warrenii

`--| | ,----- Rissikia_media

| | | ,-- Pararaucaria_patagonica

`--| |	|	,-- **New_total_plant_from_Arkansas**

| | ,--| ,----- Alvinia_bohemica_plant

| | | `--| ,-- Himeriella_muensteri

`--| |	`--| ,-- Kachaikestrobus_acuminatus

| | `----- Tomaxellia_biforme

| | ,-- Telemachus_aequatus

| | | ,-- Eathiestrobus_mackenziei

`--| |	| ,-- Cedrus_deodara

| | ,--| | ,-- Pseudotsuga_menziesii

| | | | | ,----- Larix_decidua

| | | `--| ,--| ,-- Cathaya_argyrophylla

| | | | | `--| ,-- Pinus_contorta

`--| |	| |	`----- Picea_abies

| | `--| ,-- Keteleeria_davidiana

| | | ,----- Abies_lasiocarpa

| | | | ,-- Pseudolarix_amabilis

| | `--| | ,-- Nothotsuga_longibracteata

| | `--| ,--| ,-- Mataia_podocarpoides

| | | | `----- Tsuga_canadensis

`--|	`--| ,-- Schizolepidopsis_ediae

| `--| ,-- Schizolepidopsis_daohugouensis

| `----- Schizolepidopsis_canicularis

| ,-- Sciadopitys_verticillata

| ,--| ,-- Taxus_wallichiana

| | `--| ,-- Cunninghamia_lanceolata

| | `--| ,-- Taiwania_cryptomerioides

| | `--| ,-- Athrotaxis_selaginoides

| | `--| ,-- Sequoia_sempervirens

| | `--| ,-- Cryptomeria_japonica

| | `--| ,-- Cupressus_funebris

`--|	`----- Callitris_muelleri

| ,-- Araucaria_mirabilis

| ,----- Araucaria_heterophylla

| ,--| ,-- Wollemia_nobilis

| | `----- Agathis_australis

| | ,-- Lagarostrobos_franklinii

`--|	,----- Prumnopitys_taxifolia

| ,--| ,-- Lepidothamnus_laxifolius

| | `----- Phyllocladus_aspleniifolius

`--| ,-- Saxegothaea_conspicua

| | ,-- Pherosphaera_hookeriana

`--| ,----- Microcachrys_tetragona

`--| ,-- Acmopyle_pancheri

`--| ,-- Dacrydium_cupressinum

`--| ,-- Retrophyllum_rospigliosii

`----- Podocarpus_archboldii

Tree 26:

,-- Ginkgo_biloba

| ,-- Cedrus_deodara

| | ,-- Pseudotsuga_menziesii

| | ,----- Larix_decidua

| | ,--| ,-- Cathaya_argyrophylla

| ,--| | `--| ,-- Pinus_contorta

| | | | `--| ,-- Eathiestrobus_mackenziei

|--| | | | `----- Picea_abies

| | `--| ,-- Keteleeria_davidiana

| | | ,----- Abies_lasiocarpa

| | | | ,-- Pseudolarix_amabilis

| | `--| | ,-- Tsuga_canadensis

| | `--| ,----- Nothotsuga_longibracteata

| | `--| ,-- Schizolepidopsis_ediae

`--|	`--| ,-- Schizolepidopsis_daohugouensis

| `----- Schizolepidopsis_canicularis

| ,-- Mataia_podocarpoides

| | ,-- Araucaria_mirabilis

| | ,----- Araucaria_heterophylla

| | ,--| ,-- Wollemia_nobilis

| | | `----- Agathis_australis

| | | ,-- Lagarostrobos_franklinii

| | ,--| ,----- Prumnopitys_taxifolia

`--| | | ,--| ,-- Lepidothamnus_laxifolius

| | | | `----- Phyllocladus_aspleniifolius

| | `--| ,-- Saxegothaea_conspicua

| | `--| ,-- Microcachrys_tetragona

| | `--| ,-- Pherosphaera_hookeriana

| | `--| ,-- Acmopyle_pancheri

| | `--| ,-- Dacrydium_cupressinum

`--|	`--| ,-- Retrophyllum_rospigliosii

| `----- Podocarpus_archboldii

| ,-- Pararaucaria_patagonica

| | ,-- **New_total_plant_from_Arkansas**

| ,--| ,----- Alvinia_bohemica_plant

| | `--| ,-- Himeriella_muensteri

| | `--| ,-- Kachaikestrobus_acuminatus

| | `----- Tomaxellia_biforme

`--| ,-- Sciadopitys_verticillata

| | ,-- Cunninghamia_lanceolata

| | ,--| ,-- Taiwania_cryptomerioides

`--| | `--| ,-- Athrotaxis_selaginoides

| | `--| ,-- Sequoia_sempervirens

| | `--| ,-- Cryptomeria_japonica

`--|	`--| ,-- Cupressus_funebris

| `----- Callitris_muelleri

| ,-- Telemachus_aequatus

| | ,-- Nothodacrium_warrenii

`--| ,----- Rissikia_media

| | ,-- Voltzia_hexagona

`--| |	,-- Emporia_lockardii

`--| ,----- Hanskerpia_hamiltonensis

`--| ,-- Cordaixylon_dumusum

`----- Taxus_wallichiana

Tree 27:

,-- Ginkgo_biloba

| ,-- Eathiestrobus_mackenziei

| | ,-- Cedrus_deodara

| ,--| | ,-- Keteleeria_davidiana

| | | | ,----- Abies_lasiocarpa

| | `--| ,--| ,-- Pseudolarix_amabilis

| | | | `--| ,-- Tsuga_canadensis

|--| | | | `----- Nothotsuga_longibracteata

| | `--| ,-- Pseudotsuga_menziesii

| | | ,----- Larix_decidua

| | `--| ,-- Cathaya_argyrophylla

| | `--| ,-- Pinus_contorta

| | `--| ,-- Picea_abies

`--|	`--| ,-- Schizolepidopsis_ediae

| `--| ,-- Schizolepidopsis_daohugouensis

| `----- Schizolepidopsis_canicularis

| ,-- Mataia_podocarpoides

| | ,-- Araucaria_mirabilis

| | ,----- Araucaria_heterophylla

| | ,--| ,-- Wollemia_nobilis

| | | `----- Agathis_australis

| | | ,-- Lagarostrobos_franklinii

| | ,--| ,----- Prumnopitys_taxifolia

`--| | | ,--| ,-- Lepidothamnus_laxifolius

| | | | `----- Phyllocladus_aspleniifolius

| | `--| ,-- Saxegothaea_conspicua

| | `--| ,-- Microcachrys_tetragona

| | `--| ,-- Pherosphaera_hookeriana

| | `--| ,-- Acmopyle_pancheri

| | `--| ,-- Dacrydium_cupressinum

`--|	`--| ,-- Retrophyllum_rospigliosii

| `----- Podocarpus_archboldii

| ,-- Pararaucaria_patagonica

| | ,-- **New_total_plant_from_Arkansas**

| ,--| ,----- Alvinia_bohemica_plant

| | `--| ,-- Himeriella_muensteri

| | `--| ,-- Kachaikestrobus_acuminatus

| | `----- Tomaxellia_biforme

`--| ,-- Sciadopitys_verticillata

| | ,-- Cunninghamia_lanceolata

| | ,--| ,-- Taiwania_cryptomerioides

`--| | `--| ,-- Athrotaxis_selaginoides

| | `--| ,-- Sequoia_sempervirens

| | `--| ,-- Cryptomeria_japonica

`--|	`--| ,-- Cupressus_funebris

| `----- Callitris_muelleri

| ,-- Telemachus_aequatus

| | ,-- Nothodacrium_warrenii

`--| ,----- Rissikia_media

| | ,-- Voltzia_hexagona

`--| |	,-- Emporia_lockardii

`--| ,----- Hanskerpia_hamiltonensis

`--| ,-- Cordaixylon_dumusum

`----- Taxus_wallichiana

Tree 28:

,-- Ginkgo_biloba

| ,-- Mataia_podocarpoides

| | ,-- Eathiestrobus_mackenziei

| | | ,-- Cedrus_deodara

| | ,--| | ,-- Keteleeria_davidiana

|--| | | | | ,----- Abies_lasiocarpa

| | | `--| ,--| ,-- Pseudolarix_amabilis

| | | | | `--| ,-- Tsuga_canadensis

| | | | | `----- Nothotsuga_longibracteata

`--| |	`--|	,-- Pseudotsuga_menziesii

| | | ,----- Larix_decidua

| | `--| ,-- Cathaya_argyrophylla

| | `--| ,-- Pinus_contorta

| | `--| ,-- Picea_abies

| | `--| ,-- Schizolepidopsis_ediae

| | `--| ,-- Schizolepidopsis_daohugouensis

| | `----- Schizolepidopsis_canicularis

`--|	,-- Araucaria_mirabilis

| ,----- Araucaria_heterophylla

| ,--| ,-- Wollemia_nobilis

| | `----- Agathis_australis

| | ,-- Lagarostrobos_franklinii

| ,--| ,----- Prumnopitys_taxifolia

| | | ,--| ,-- Lepidothamnus_laxifolius

| | | | `----- Phyllocladus_aspleniifolius

| | `--| ,-- Saxegothaea_conspicua

| | `--| ,-- Microcachrys_tetragona

| | `--| ,-- Pherosphaera_hookeriana

| | `--| ,-- Acmopyle_pancheri

| | `--| ,-- Dacrydium_cupressinum

`--|	`--| ,-- Retrophyllum_rospigliosii

| `----- Podocarpus_archboldii

| ,-- Pararaucaria_patagonica

| | ,-- **New_total_plant_from_Arkansas**

| ,--| ,----- Alvinia_bohemica_plant

| | `--| ,-- Himeriella_muensteri

| | `--| ,-- Kachaikestrobus_acuminatus

| | `----- Tomaxellia_biforme

`--| ,-- Sciadopitys_verticillata

| | ,-- Cunninghamia_lanceolata

| | ,--| ,-- Taiwania_cryptomerioides

`--| | `--| ,-- Athrotaxis_selaginoides

| | `--| ,-- Sequoia_sempervirens

| | `--| ,-- Cryptomeria_japonica

`--|	`--| ,-- Cupressus_funebris

| `----- Callitris_muelleri

| ,-- Telemachus_aequatus

| | ,-- Nothodacrium_warrenii

`--| ,----- Rissikia_media

| | ,-- Voltzia_hexagona

`--| |	,-- Emporia_lockardii

`--| ,----- Hanskerpia_hamiltonensis

`--| ,-- Cordaixylon_dumusum

`----- Taxus_wallichiana

Tree 29:

,-- Ginkgo_biloba

| ,-- Cedrus_deodara

| | ,-- Keteleeria_davidiana

| | ,----- Abies_lasiocarpa

| ,--| ,--| ,-- Pseudolarix_amabilis

| | | | `--| ,-- Tsuga_canadensis

| | | | `----- Nothotsuga_longibracteata

|--| | `--| ,-- Pseudotsuga_menziesii

| | | ,----- Larix_decidua

| | | | ,-- Cathaya_argyrophylla

| | `--| | ,-- Eathiestrobus_mackenziei

| | `--| ,----- Pinus_contorta

| | `--| ,-- Picea_abies

| | `--| ,-- Schizolepidopsis_ediae

`--|	`--| ,-- Schizolepidopsis_daohugouensis

| `----- Schizolepidopsis_canicularis

| ,-- Mataia_podocarpoides

| | ,-- Araucaria_mirabilis

| | ,----- Araucaria_heterophylla

| | ,--| ,-- Wollemia_nobilis

| | | `----- Agathis_australis

| | | ,-- Lagarostrobos_franklinii

| | ,--| ,----- Prumnopitys_taxifolia

`--| | | ,--| ,-- Lepidothamnus_laxifolius

| | | | `----- Phyllocladus_aspleniifolius

| | `--| ,-- Saxegothaea_conspicua

| | `--| ,-- Microcachrys_tetragona

| | `--| ,-- Pherosphaera_hookeriana

| | `--| ,-- Acmopyle_pancheri

| | `--| ,-- Dacrydium_cupressinum

`--|	`--| ,-- Retrophyllum_rospigliosii

| `----- Podocarpus_archboldii

| ,-- Pararaucaria_patagonica

| | ,-- **New_total_plant_from_Arkansas**

| ,--| ,----- Alvinia_bohemica_plant

| | `--| ,-- Himeriella_muensteri

| | `--| ,-- Kachaikestrobus_acuminatus

| | `----- Tomaxellia_biforme

`--| ,-- Sciadopitys_verticillata

| | ,-- Cunninghamia_lanceolata

| | ,--| ,-- Taiwania_cryptomerioides

`--| | `--| ,-- Athrotaxis_selaginoides

| | `--| ,-- Sequoia_sempervirens

| | `--| ,-- Cryptomeria_japonica

`--|	`--| ,-- Cupressus_funebris

| `----- Callitris_muelleri

| ,-- Telemachus_aequatus

| | ,-- Nothodacrium_warrenii

`--| ,----- Rissikia_media

| | ,-- Voltzia_hexagona

`--| |	,-- Emporia_lockardii

`--| ,----- Hanskerpia_hamiltonensis

`--| ,-- Cordaixylon_dumusum

`----- Taxus_wallichiana

Tree 30:

,-- Ginkgo_biloba

| ,-- Cordaixylon_dumusum

|--| | ,-- Emporia_lockardii

| | ,----- Hanskerpia_hamiltonensis

`--| | ,-- Voltzia_hexagona

| | | ,-- Nothodacrium_warrenii

`--| | ,----- Rissikia_media

| | | ,-- Telemachus_aequatus

`--| | |	,-- Schizolepidopsis_ediae

| | | ,--| ,-- Schizolepidopsis_daohugouensis

| | | | `----- Schizolepidopsis_canicularis

`--| |	| ,-- Cedrus_deodara

| | ,--| | ,-- Abies_lasiocarpa

| | | | | ,--| ,-- Mataia_podocarpoides

| | | | | | `----- Keteleeria_davidiana

`--| | `--| ,--| ,-- Pseudolarix_amabilis

| | | | `--| ,-- Tsuga_canadensis

| | | | `----- Nothotsuga_longibracteata

| | `--| ,-- Pseudotsuga_menziesii

| | | ,----- Larix_decidua

| | `--| ,-- Cathaya_argyrophylla

| | `--| ,-- Pinus_contorta

| | `--| ,-- Eathiestrobus_mackenziei

`--|	`----- Picea_abies

| ,-- Sciadopitys_verticillata

| ,--| ,-- Taxus_wallichiana

| | `--| ,-- Cunninghamia_lanceolata

| | `--| ,-- Taiwania_cryptomerioides

| | `--| ,-- Athrotaxis_selaginoides

| | `--| ,-- Sequoia_sempervirens

| | `--| ,-- Cryptomeria_japonica

| | `--| ,-- Cupressus_funebris

| | `----- Callitris_muelleri

`--|	,-- Pararaucaria_patagonica

| | ,-- **New_total_plant_from_Arkansas**

| ,--| ,----- Alvinia_bohemica_plant

| | `--| ,-- Himeriella_muensteri

| | `--| ,-- Kachaikestrobus_acuminatus

| | `----- Tomaxellia_biforme

| | ,-- Araucaria_mirabilis

`--|	,----- Araucaria_heterophylla

| ,--| ,-- Wollemia_nobilis

| | `----- Agathis_australis

| | ,-- Lagarostrobos_franklinii

`--|	,----- Prumnopitys_taxifolia

| ,--| ,-- Lepidothamnus_laxifolius

| | `----- Phyllocladus_aspleniifolius

`--| ,-- Saxegothaea_conspicua

`--| ,-- Microcachrys_tetragona

`--| ,-- Pherosphaera_hookeriana

`--| ,-- Acmopyle_pancheri

`--| ,-- Dacrydium_cupressinum

`--| ,-- Retrophyllum_rospigliosii

`----- Podocarpus_archboldii

Tree 31:

,-- Ginkgo_biloba

| ,-- Cordaixylon_dumusum

|--| | ,-- Emporia_lockardii

| | ,----- Hanskerpia_hamiltonensis

`--| | ,-- Voltzia_hexagona

| | | ,-- Nothodacrium_warrenii

`--| | ,----- Rissikia_media

| | | ,-- **New_total_plant_from_Arkansas**

`--| |	,--| ,-- Alvinia_bohemica_plant

| | | `----- Pararaucaria_patagonica

| | ,--| ,-- Himeriella_muensteri

`--| | `--| ,-- Kachaikestrobus_acuminatus

| | `----- Tomaxellia_biforme

| | ,-- Telemachus_aequatus

| | | ,-- Eathiestrobus_mackenziei

`--| |	| ,-- Cedrus_deodara

| | ,--| | ,-- Pseudotsuga_menziesii

| | | | | ,----- Larix_decidua

| | | `--| ,--| ,-- Cathaya_argyrophylla

| | | | | `--| ,-- Pinus_contorta

`--| |	| |	`----- Picea_abies

| | `--| ,-- Keteleeria_davidiana

| | | ,----- Abies_lasiocarpa

| | | | ,-- Pseudolarix_amabilis

| | `--| | ,-- Nothotsuga_longibracteata

| | `--| ,--| ,-- Mataia_podocarpoides

| | | | `----- Tsuga_canadensis

`--|	`--| ,-- Schizolepidopsis_ediae

| `--| ,-- Schizolepidopsis_daohugouensis

| `----- Schizolepidopsis_canicularis

| ,-- Sciadopitys_verticillata

| ,--| ,-- Taxus_wallichiana

| | `--| ,-- Cunninghamia_lanceolata

| | `--| ,-- Taiwania_cryptomerioides

| | `--| ,-- Athrotaxis_selaginoides

| | `--| ,-- Sequoia_sempervirens

| | `--| ,-- Cryptomeria_japonica

| | `--| ,-- Cupressus_funebris

`--|	`----- Callitris_muelleri

| ,-- Araucaria_mirabilis

| ,----- Araucaria_heterophylla

| ,--| ,-- Wollemia_nobilis

| | `----- Agathis_australis

| | ,-- Lagarostrobos_franklinii

`--|	,----- Prumnopitys_taxifolia

| ,--| ,-- Lepidothamnus_laxifolius

| | `----- Phyllocladus_aspleniifolius

`--| ,-- Saxegothaea_conspicua

`--| ,-- Microcachrys_tetragona

`--| ,-- Pherosphaera_hookeriana

`--| ,-- Acmopyle_pancheri

`--| ,-- Dacrydium_cupressinum

`--| ,-- Retrophyllum_rospigliosii

`----- Podocarpus_archboldii

Tree 32:

,-- Ginkgo_biloba

| ,-- Cordaixylon_dumusum

|--| | ,-- Emporia_lockardii

| | ,----- Hanskerpia_hamiltonensis

`--| | ,-- Voltzia_hexagona

| | | ,-- Nothodacrium_warrenii

`--| | ,----- Rissikia_media

| | | ,-- Pararaucaria_patagonica

`--| |	|	,-- **New_total_plant_from_Arkansas**

| | ,--| ,----- Alvinia_bohemica_plant

| | | `--| ,-- Himeriella_muensteri

`--| |	`--| ,-- Kachaikestrobus_acuminatus

| | `----- Tomaxellia_biforme

| | ,-- Telemachus_aequatus

| | | ,-- Eathiestrobus_mackenziei

`--| |	| ,-- Cedrus_deodara

| | | | ,-- Mataia_podocarpoides

| | ,--| | ,--| ,-- Keteleeria_davidiana

| | | | | | `----- Abies_lasiocarpa

| | | `--| ,--| ,-- Pseudolarix_amabilis

| | | | | `--| ,-- Tsuga_canadensis

`--| |	| |	`----- Nothotsuga_longibracteata

| | `--| ,-- Pseudotsuga_menziesii

| | | ,----- Larix_decidua

| | `--| ,-- Cathaya_argyrophylla

| | `--| ,-- Pinus_contorta

| | `--| ,-- Picea_abies

| | `--| ,-- Schizolepidopsis_ediae

`--|	`--| ,-- Schizolepidopsis_daohugouensis

| `----- Schizolepidopsis_canicularis

| ,-- Sciadopitys_verticillata

| ,--| ,-- Taxus_wallichiana

| | `--| ,-- Cunninghamia_lanceolata

| | `--| ,-- Taiwania_cryptomerioides

| | `--| ,-- Athrotaxis_selaginoides

| | `--| ,-- Sequoia_sempervirens

| | `--| ,-- Cryptomeria_japonica

| | `--| ,-- Cupressus_funebris

`--|	`----- Callitris_muelleri

| ,-- Araucaria_mirabilis

| ,----- Araucaria_heterophylla

| ,--| ,-- Wollemia_nobilis

| | `----- Agathis_australis

| | ,-- Lagarostrobos_franklinii

`--|	,----- Prumnopitys_taxifolia

| ,--| ,-- Lepidothamnus_laxifolius

| | `----- Phyllocladus_aspleniifolius

`--| ,-- Saxegothaea_conspicua

`--| ,-- Microcachrys_tetragona

`--| ,-- Pherosphaera_hookeriana

`--| ,-- Acmopyle_pancheri

`--| ,-- Dacrydium_cupressinum

`--| ,-- Retrophyllum_rospigliosii

`----- Podocarpus_archboldii

Tree 33:

,-- Ginkgo_biloba

| ,-- Cordaixylon_dumusum

|--| | ,-- Emporia_lockardii

| | ,----- Hanskerpia_hamiltonensis

`--| | ,-- Voltzia_hexagona

| | | ,-- Nothodacrium_warrenii

`--| | ,----- Rissikia_media

| | | ,-- Alvinia_bohemica_plant

`--| |	,--| ,-- **New_total_plant_from_Arkansas**

| | | `----- Pararaucaria_patagonica

| | ,--| ,-- Himeriella_muensteri

`--| | `--| ,-- Kachaikestrobus_acuminatus

| | `----- Tomaxellia_biforme

| | ,-- Telemachus_aequatus

| | | ,-- Eathiestrobus_mackenziei

| | | | ,-- Cedrus_deodara

`--| |	| |	,-- Mataia_podocarpoides

| | ,--| | ,--| ,-- Keteleeria_davidiana

| | | | | | `----- Abies_lasiocarpa

| | | `--| ,--| ,-- Pseudolarix_amabilis

| | | | | `--| ,-- Tsuga_canadensis

`--| |	| |	`----- Nothotsuga_longibracteata

| | `--| ,-- Pseudotsuga_menziesii

| | | ,----- Larix_decidua

| | `--| ,-- Cathaya_argyrophylla

| | `--| ,-- Pinus_contorta

| | `--| ,-- Picea_abies

| | `--| ,-- Schizolepidopsis_ediae

`--|	`--| ,-- Schizolepidopsis_daohugouensis

| `----- Schizolepidopsis_canicularis

| ,-- Sciadopitys_verticillata

| ,--| ,-- Taxus_wallichiana

| | `--| ,-- Cunninghamia_lanceolata

| | `--| ,-- Taiwania_cryptomerioides

| | `--| ,-- Athrotaxis_selaginoides

| | `--| ,-- Sequoia_sempervirens

| | `--| ,-- Cryptomeria_japonica

| | `--| ,-- Cupressus_funebris

`--|	`----- Callitris_muelleri

| ,-- Araucaria_mirabilis

| ,----- Araucaria_heterophylla

| ,--| ,-- Wollemia_nobilis

| | `----- Agathis_australis

| | ,-- Lagarostrobos_franklinii

`--|	,----- Prumnopitys_taxifolia

| ,--| ,-- Lepidothamnus_laxifolius

| | `----- Phyllocladus_aspleniifolius

`--| ,-- Saxegothaea_conspicua

`--| ,-- Microcachrys_tetragona

`--| ,-- Pherosphaera_hookeriana

`--| ,-- Acmopyle_pancheri

`--| ,-- Dacrydium_cupressinum

`--| ,-- Retrophyllum_rospigliosii

`----- Podocarpus_archboldii

Tree 34:

,-- Ginkgo_biloba

| ,-- Cordaixylon_dumusum

|--| | ,-- Emporia_lockardii

| | ,----- Hanskerpia_hamiltonensis

`--| | ,-- Voltzia_hexagona

| | | ,-- Nothodacrium_warrenii

`--| | ,----- Rissikia_media

| | | ,-- Telemachus_aequatus

`--| | |	,-- Schizolepidopsis_daohugouensis

| | | ,----- Schizolepidopsis_canicularis

| | | | ,-- Schizolepidopsis_ediae

`--| | ,--| | ,-- Cedrus_deodara

| | | | | | ,-- Keteleeria_davidiana

| | | `--| | ,----- Abies_lasiocarpa

| | | | | | ,-- Mataia_podocarpoides

`--| |	`--| ,--| ,----- Pseudolarix_amabilis

| | | | `--| ,-- Tsuga_canadensis

| | | | `----- Nothotsuga_longibracteata

| | `--| ,-- Pseudotsuga_menziesii

| | | ,----- Larix_decidua

| | `--| ,-- Cathaya_argyrophylla

| | `--| ,-- Pinus_contorta

`--|	`--| ,-- Eathiestrobus_mackenziei

| `----- Picea_abies

| ,-- Sciadopitys_verticillata

| ,--| ,-- Taxus_wallichiana

| | `--| ,-- Cunninghamia_lanceolata

| | `--| ,-- Taiwania_cryptomerioides

| | `--| ,-- Athrotaxis_selaginoides

| | `--| ,-- Sequoia_sempervirens

| | `--| ,-- Cryptomeria_japonica

| | `--| ,-- Cupressus_funebris

| | `----- Callitris_muelleri

`--|	,-- Pararaucaria_patagonica

| | ,-- **New_total_plant_from_Arkansas**

| ,--| ,----- Alvinia_bohemica_plant

| | `--| ,-- Himeriella_muensteri

| | `--| ,-- Kachaikestrobus_acuminatus

| | `----- Tomaxellia_biforme

| | ,-- Araucaria_mirabilis

`--|	,----- Araucaria_heterophylla

| ,--| ,-- Wollemia_nobilis

| | `----- Agathis_australis

| | ,-- Lagarostrobos_franklinii

`--|	,----- Prumnopitys_taxifolia

| ,--| ,-- Lepidothamnus_laxifolius

| | `----- Phyllocladus_aspleniifolius

`--| ,-- Saxegothaea_conspicua

`--| ,-- Microcachrys_tetragona

`--| ,-- Pherosphaera_hookeriana

`--| ,-- Acmopyle_pancheri

`--| ,-- Dacrydium_cupressinum

`--| ,-- Retrophyllum_rospigliosii

`----- Podocarpus_archboldii

Tree 35:

,-- Ginkgo_biloba

| ,-- Cordaixylon_dumusum

|--| | ,-- Emporia_lockardii

| | ,----- Hanskerpia_hamiltonensis

`--| | ,-- Voltzia_hexagona

| | | ,-- Nothodacrium_warrenii

`--| | ,----- Rissikia_media

| | | ,-- Telemachus_aequatus

`--| | |	,-- Schizolepidopsis_daohugouensis

| | | ,----- Schizolepidopsis_canicularis

| | | | ,-- Schizolepidopsis_ediae

`--| | ,--| | ,-- Cedrus_deodara

| | | `--| | ,-- Keteleeria_davidiana

| | | | | ,----- Abies_lasiocarpa

| | | `--| ,--| ,-- Pseudolarix_amabilis

`--| |	| | `--| ,-- Mataia_podocarpoides

| | | | `--| ,-- Tsuga_canadensis

| | `--| `----- Nothotsuga_longibracteata

| | | ,-- Pseudotsuga_menziesii

| | | ,----- Larix_decidua

| | `--| ,-- Cathaya_argyrophylla

| | `--| ,-- Picea_abies

`--|	`--| ,-- Eathiestrobus_mackenziei

| `----- Pinus_contorta

| ,-- Sciadopitys_verticillata

| ,--| ,-- Taxus_wallichiana

| | `--| ,-- Cunninghamia_lanceolata

| | `--| ,-- Taiwania_cryptomerioides

| | `--| ,-- Athrotaxis_selaginoides

| | `--| ,-- Sequoia_sempervirens

| | `--| ,-- Cryptomeria_japonica

| | `--| ,-- Cupressus_funebris

| | `----- Callitris_muelleri

`--|	,-- Pararaucaria_patagonica

| | ,-- **New_total_plant_from_Arkansas**

| ,--| ,----- Alvinia_bohemica_plant

| | `--| ,-- Himeriella_muensteri

| | `--| ,-- Kachaikestrobus_acuminatus

| | `----- Tomaxellia_biforme

| | ,-- Araucaria_mirabilis

`--|	,----- Araucaria_heterophylla

| ,--| ,-- Wollemia_nobilis

| | `----- Agathis_australis

| | ,-- Lagarostrobos_franklinii

`--|	,----- Prumnopitys_taxifolia

| ,--| ,-- Lepidothamnus_laxifolius

| | `----- Phyllocladus_aspleniifolius

`--| ,-- Saxegothaea_conspicua

`--| ,-- Microcachrys_tetragona

`--| ,-- Pherosphaera_hookeriana

`--| ,-- Acmopyle_pancheri

`--| ,-- Dacrydium_cupressinum

`--| ,-- Retrophyllum_rospigliosii

`----- Podocarpus_archboldii

Tree 36:

,-- Ginkgo_biloba

| ,-- Cordaixylon_dumusum

|--| | ,-- Emporia_lockardii

| | ,----- Hanskerpia_hamiltonensis

`--| | ,-- Voltzia_hexagona

| | | ,-- Nothodacrium_warrenii

`--| | ,----- Rissikia_media

| | | ,-- Telemachus_aequatus

`--| | |	,-- Schizolepidopsis_daohugouensis

| | | ,----- Schizolepidopsis_canicularis

| | | | ,-- Schizolepidopsis_ediae

`--| | ,--| | ,-- Cedrus_deodara

| | | `--| | ,-- Keteleeria_davidiana

| | | | | ,----- Abies_lasiocarpa

| | | `--| ,--| ,-- Mataia_podocarpoides

`--| |	| | `--| ,-- Pseudolarix_amabilis

| | | | `--| ,-- Tsuga_canadensis

| | `--| `----- Nothotsuga_longibracteata

| | | ,-- Pseudotsuga_menziesii

| | | ,----- Larix_decidua

| | `--| ,-- Cathaya_argyrophylla

| | `--| ,-- Picea_abies

`--|	`--| ,-- Eathiestrobus_mackenziei

| `----- Pinus_contorta

| ,-- Sciadopitys_verticillata

| ,--| ,-- Taxus_wallichiana

| | `--| ,-- Cunninghamia_lanceolata

| | `--| ,-- Taiwania_cryptomerioides

| | `--| ,-- Athrotaxis_selaginoides

| | `--| ,-- Sequoia_sempervirens

| | `--| ,-- Cryptomeria_japonica

| | `--| ,-- Cupressus_funebris

| | `----- Callitris_muelleri

`--|	,-- Pararaucaria_patagonica

| | ,-- **New_total_plant_from_Arkansas**

| ,--| ,----- Alvinia_bohemica_plant

| | `--| ,-- Himeriella_muensteri

| | `--| ,-- Kachaikestrobus_acuminatus

| | `----- Tomaxellia_biforme

| | ,-- Araucaria_mirabilis

`--|	,----- Araucaria_heterophylla

| ,--| ,-- Wollemia_nobilis

| | `----- Agathis_australis

| | ,-- Lagarostrobos_franklinii

`--|	,----- Prumnopitys_taxifolia

| ,--| ,-- Lepidothamnus_laxifolius

| | `----- Phyllocladus_aspleniifolius

`--| ,-- Saxegothaea_conspicua

`--| ,-- Microcachrys_tetragona

`--| ,-- Pherosphaera_hookeriana

`--| ,-- Acmopyle_pancheri

`--| ,-- Dacrydium_cupressinum

`--| ,-- Retrophyllum_rospigliosii

`----- Podocarpus_archboldii

Tree 37:

,-- Ginkgo_biloba

| ,-- Cordaixylon_dumusum

|--| | ,-- Emporia_lockardii

| | ,----- Hanskerpia_hamiltonensis

`--| | ,-- Voltzia_hexagona

| | | ,-- Nothodacrium_warrenii

`--| | ,----- Rissikia_media

| | | ,-- Telemachus_aequatus

`--| | |	,-- Schizolepidopsis_daohugouensis

| | | ,----- Schizolepidopsis_canicularis

| | | | ,-- Schizolepidopsis_ediae

`--| | ,--| | ,-- Cedrus_deodara

| | | | | | ,-- Mataia_podocarpoides

| | | `--| | ,--| ,-- Keteleeria_davidiana

| | | | | | `----- Abies_lasiocarpa

`--| |	`--| ,--| ,-- Pseudolarix_amabilis

| | | | `--| ,-- Tsuga_canadensis

| | | | `----- Nothotsuga_longibracteata

| | `--| ,-- Pseudotsuga_menziesii

| | | ,----- Larix_decidua

| | `--| ,-- Cathaya_argyrophylla

| | `--| ,-- Picea_abies

`--|	`--| ,-- Eathiestrobus_mackenziei

| `----- Pinus_contorta

| ,-- Sciadopitys_verticillata

| ,--| ,-- Taxus_wallichiana

| | `--| ,-- Cunninghamia_lanceolata

| | `--| ,-- Taiwania_cryptomerioides

| | `--| ,-- Athrotaxis_selaginoides

| | `--| ,-- Sequoia_sempervirens

| | `--| ,-- Cryptomeria_japonica

| | `--| ,-- Cupressus_funebris

| | `----- Callitris_muelleri

`--|	,-- Pararaucaria_patagonica

| | ,-- **New_total_plant_from_Arkansas**

| ,--| ,----- Alvinia_bohemica_plant

| | `--| ,-- Himeriella_muensteri

| | `--| ,-- Kachaikestrobus_acuminatus

| | `----- Tomaxellia_biforme

| | ,-- Araucaria_mirabilis

`--|	,----- Araucaria_heterophylla

| ,--| ,-- Wollemia_nobilis

| | `----- Agathis_australis

| | ,-- Lagarostrobos_franklinii

`--|	,----- Prumnopitys_taxifolia

| ,--| ,-- Lepidothamnus_laxifolius

| | `----- Phyllocladus_aspleniifolius

`--| ,-- Saxegothaea_conspicua

`--| ,-- Microcachrys_tetragona

`--| ,-- Pherosphaera_hookeriana

`--| ,-- Acmopyle_pancheri

`--| ,-- Dacrydium_cupressinum

`--| ,-- Retrophyllum_rospigliosii

`----- Podocarpus_archboldii

Tree 38:

,-- Ginkgo_biloba

| ,-- Cordaixylon_dumusum

|--| | ,-- Emporia_lockardii

| | ,----- Hanskerpia_hamiltonensis

`--| | ,-- Voltzia_hexagona

| | | ,-- Nothodacrium_warrenii

`--| | ,----- Rissikia_media

| | | ,-- Telemachus_aequatus

`--| | |	,-- Schizolepidopsis_daohugouensis

| | | ,----- Schizolepidopsis_canicularis

| | | | ,-- Schizolepidopsis_ediae

`--| | ,--| | ,-- Cedrus_deodara

| | | | | | ,-- Keteleeria_davidiana

| | | `--| | ,--| ,-- Mataia_podocarpoides

| | | | | | `----- Abies_lasiocarpa

`--| |	`--| ,--| ,-- Pseudolarix_amabilis

| | | | `--| ,-- Tsuga_canadensis

| | | | `----- Nothotsuga_longibracteata

| | `--| ,-- Pseudotsuga_menziesii

| | | ,----- Larix_decidua

| | `--| ,-- Cathaya_argyrophylla

| | `--| ,-- Picea_abies

`--|	`--| ,-- Eathiestrobus_mackenziei

| `----- Pinus_contorta

| ,-- Sciadopitys_verticillata

| ,--| ,-- Taxus_wallichiana

| | `--| ,-- Cunninghamia_lanceolata

| | `--| ,-- Taiwania_cryptomerioides

| | `--| ,-- Athrotaxis_selaginoides

| | `--| ,-- Sequoia_sempervirens

| | `--| ,-- Cryptomeria_japonica

| | `--| ,-- Cupressus_funebris

| | `----- Callitris_muelleri

`--|	,-- Pararaucaria_patagonica

| | ,-- **New_total_plant_from_Arkansas**

| ,--| ,----- Alvinia_bohemica_plant

| | `--| ,-- Himeriella_muensteri

| | `--| ,-- Kachaikestrobus_acuminatus

| | `----- Tomaxellia_biforme

| | ,-- Araucaria_mirabilis

`--|	,----- Araucaria_heterophylla

| ,--| ,-- Wollemia_nobilis

| | `----- Agathis_australis

| | ,-- Lagarostrobos_franklinii

`--|	,----- Prumnopitys_taxifolia

| ,--| ,-- Lepidothamnus_laxifolius

| | `----- Phyllocladus_aspleniifolius

`--| ,-- Saxegothaea_conspicua

`--| ,-- Microcachrys_tetragona

`--| ,-- Pherosphaera_hookeriana

`--| ,-- Acmopyle_pancheri

`--| ,-- Dacrydium_cupressinum

`--| ,-- Retrophyllum_rospigliosii

`----- Podocarpus_archboldii

Tree 39:

,-- Ginkgo_biloba

| ,-- Cordaixylon_dumusum

|--| | ,-- Emporia_lockardii

| | ,----- Hanskerpia_hamiltonensis

`--| | ,-- Voltzia_hexagona

| | | ,-- Nothodacrium_warrenii

`--| | ,----- Rissikia_media

| | | ,-- Telemachus_aequatus

`--| | |	,-- Schizolepidopsis_daohugouensis

| | | ,----- Schizolepidopsis_canicularis

| | | | ,-- Schizolepidopsis_ediae

`--| | ,--| | ,-- Cedrus_deodara

| | | `--| | ,-- Keteleeria_davidiana

| | | | | ,----- Abies_lasiocarpa

| | | `--| ,--| ,-- Pseudolarix_amabilis

`--| |	| | `--| ,-- Tsuga_canadensis

| | | | `--| ,-- Mataia_podocarpoides

| | `--| `----- Nothotsuga_longibracteata

| | | ,-- Pseudotsuga_menziesii

| | | ,----- Larix_decidua

| | `--| ,-- Cathaya_argyrophylla

| | `--| ,-- Picea_abies

`--|	`--| ,-- Eathiestrobus_mackenziei

| `----- Pinus_contorta

| ,-- Sciadopitys_verticillata

| ,--| ,-- Taxus_wallichiana

| | `--| ,-- Cunninghamia_lanceolata

| | `--| ,-- Taiwania_cryptomerioides

| | `--| ,-- Athrotaxis_selaginoides

| | `--| ,-- Sequoia_sempervirens

| | `--| ,-- Cryptomeria_japonica

| | `--| ,-- Cupressus_funebris

| | `----- Callitris_muelleri

`--|	,-- Pararaucaria_patagonica

| | ,-- **New_total_plant_from_Arkansas**

| ,--| ,----- Alvinia_bohemica_plant

| | `--| ,-- Himeriella_muensteri

| | `--| ,-- Kachaikestrobus_acuminatus

| | `----- Tomaxellia_biforme

| | ,-- Araucaria_mirabilis

`--|	,----- Araucaria_heterophylla

| ,--| ,-- Wollemia_nobilis

| | `----- Agathis_australis

| | ,-- Lagarostrobos_franklinii

`--|	,----- Prumnopitys_taxifolia

| ,--| ,-- Lepidothamnus_laxifolius

| | `----- Phyllocladus_aspleniifolius

`--| ,-- Saxegothaea_conspicua

`--| ,-- Microcachrys_tetragona

`--| ,-- Pherosphaera_hookeriana

`--| ,-- Acmopyle_pancheri

`--| ,-- Dacrydium_cupressinum

`--| ,-- Retrophyllum_rospigliosii

`----- Podocarpus_archboldii

Tree 40:

,-- Ginkgo_biloba

| ,-- Cordaixylon_dumusum

|--| | ,-- Emporia_lockardii

| | ,----- Hanskerpia_hamiltonensis

`--| | ,-- Voltzia_hexagona

| | | ,-- Nothodacrium_warrenii

`--| | ,----- Rissikia_media

| | | ,-- Pararaucaria_patagonica

`--| |	,--| ,-- **New_total_plant_from_Arkansas**

| | | `----- Alvinia_bohemica_plant

| | ,--| ,-- Himeriella_muensteri

`--| | `--| ,-- Kachaikestrobus_acuminatus

| | `----- Tomaxellia_biforme

| | ,-- Telemachus_aequatus

| | | ,-- Eathiestrobus_mackenziei

`--| |	| ,-- Cedrus_deodara

| | ,--| | ,-- Pseudotsuga_menziesii

| | | | | ,----- Larix_decidua

| | | `--| ,--| ,-- Cathaya_argyrophylla

| | | | | `--| ,-- Pinus_contorta

`--| |	| |	`----- Picea_abies

| | `--| ,-- Mataia_podocarpoides

| | | ,--| ,-- Keteleeria_davidiana

| | | | `----- Abies_lasiocarpa

| | `--| ,-- Pseudolarix_amabilis

| | | | ,-- Tsuga_canadensis

| | `--| ,----- Nothotsuga_longibracteata

`--|	`--| ,-- Schizolepidopsis_ediae

| `--| ,-- Schizolepidopsis_daohugouensis

| `----- Schizolepidopsis_canicularis

| ,-- Sciadopitys_verticillata

| ,--| ,-- Taxus_wallichiana

| | `--| ,-- Cunninghamia_lanceolata

| | `--| ,-- Taiwania_cryptomerioides

| | `--| ,-- Athrotaxis_selaginoides

| | `--| ,-- Sequoia_sempervirens

| | `--| ,-- Cryptomeria_japonica

| | `--| ,-- Cupressus_funebris

`--|	`----- Callitris_muelleri

| ,-- Araucaria_mirabilis

| ,----- Araucaria_heterophylla

| ,--| ,-- Wollemia_nobilis

| | `----- Agathis_australis

| | ,-- Lagarostrobos_franklinii

`--|	,----- Prumnopitys_taxifolia

| ,--| ,-- Lepidothamnus_laxifolius

| | `----- Phyllocladus_aspleniifolius

`--| ,-- Saxegothaea_conspicua

| | ,-- Pherosphaera_hookeriana

`--| ,----- Microcachrys_tetragona

`--| ,-- Acmopyle_pancheri

`--| ,-- Dacrydium_cupressinum

`--| ,-- Retrophyllum_rospigliosii

`----- Podocarpus_archboldii

Tree 41:

,-- Ginkgo_biloba

| ,-- Cordaixylon_dumusum

|--| | ,-- Emporia_lockardii

| | ,----- Hanskerpia_hamiltonensis

`--| | ,-- Voltzia_hexagona

| | | ,-- Nothodacrium_warrenii

`--| | ,----- Rissikia_media

| | | ,-- Alvinia_bohemica_plant

`--| |	,--| ,-- **New_total_plant_from_Arkansas**

| | | `----- Pararaucaria_patagonica

| | ,--| ,-- Himeriella_muensteri

`--| | `--| ,-- Kachaikestrobus_acuminatus

| | `----- Tomaxellia_biforme

| | ,-- Telemachus_aequatus

| | | ,-- Eathiestrobus_mackenziei

`--| |	| ,-- Cedrus_deodara

| | ,--| | ,-- Pseudotsuga_menziesii

| | | | | ,----- Larix_decidua

| | | `--| ,--| ,-- Cathaya_argyrophylla

| | | | | `--| ,-- Pinus_contorta

`--| |	| |	`----- Picea_abies

| | `--| ,-- Keteleeria_davidiana

| | | ,----- Abies_lasiocarpa

| | | | ,-- Pseudolarix_amabilis

| | `--| | ,-- Mataia_podocarpoides

| | `--| ,--| ,-- Tsuga_canadensis

| | | | `----- Nothotsuga_longibracteata

`--|	`--| ,-- Schizolepidopsis_ediae

| `--| ,-- Schizolepidopsis_daohugouensis

| `----- Schizolepidopsis_canicularis

| ,-- Sciadopitys_verticillata

| ,--| ,-- Taxus_wallichiana

| | `--| ,-- Cunninghamia_lanceolata

| | `--| ,-- Taiwania_cryptomerioides

| | `--| ,-- Athrotaxis_selaginoides

| | `--| ,-- Sequoia_sempervirens

| | `--| ,-- Cryptomeria_japonica

| | `--| ,-- Cupressus_funebris

`--|	`----- Callitris_muelleri

| ,-- Araucaria_mirabilis

| ,----- Araucaria_heterophylla

| ,--| ,-- Wollemia_nobilis

| | `----- Agathis_australis

| | ,-- Lagarostrobos_franklinii

`--|	,----- Prumnopitys_taxifolia

| ,--| ,-- Lepidothamnus_laxifolius

| | `----- Phyllocladus_aspleniifolius

`--| ,-- Saxegothaea_conspicua

| | ,-- Pherosphaera_hookeriana

`--| ,----- Microcachrys_tetragona

`--| ,-- Acmopyle_pancheri

`--| ,-- Dacrydium_cupressinum

`--| ,-- Retrophyllum_rospigliosii

`----- Podocarpus_archboldii

Tree 42:

,-- Ginkgo_biloba

| ,-- Cordaixylon_dumusum

|--| | ,-- Emporia_lockardii

| | ,----- Hanskerpia_hamiltonensis

`--| | ,-- Voltzia_hexagona

| | | ,-- Nothodacrium_warrenii

`--| | ,----- Rissikia_media

| | | ,-- Alvinia_bohemica_plant

`--| |	,--| ,-- **New_total_plant_from_Arkansas**

| | | `----- Pararaucaria_patagonica

| | ,--| ,-- Himeriella_muensteri

`--| | `--| ,-- Kachaikestrobus_acuminatus

| | `----- Tomaxellia_biforme

| | ,-- Telemachus_aequatus

| | | ,-- Eathiestrobus_mackenziei

| | | | ,-- Cedrus_deodara

`--| |	| |	,-- Mataia_podocarpoides

| | ,--| | ,--| ,-- Keteleeria_davidiana

| | | | | | `----- Abies_lasiocarpa

| | | `--| ,--| ,-- Pseudolarix_amabilis

| | | | | `--| ,-- Tsuga_canadensis

`--| |	| |	`----- Nothotsuga_longibracteata

| | `--| ,-- Pseudotsuga_menziesii

| | | ,----- Larix_decidua

| | `--| ,-- Cathaya_argyrophylla

| | `--| ,-- Pinus_contorta

| | `--| ,-- Picea_abies

| | `--| ,-- Schizolepidopsis_ediae

`--|	`--| ,-- Schizolepidopsis_daohugouensis

| `----- Schizolepidopsis_canicularis

| ,-- Sciadopitys_verticillata

| ,--| ,-- Taxus_wallichiana

| | `--| ,-- Cunninghamia_lanceolata

| | `--| ,-- Taiwania_cryptomerioides

| | `--| ,-- Athrotaxis_selaginoides

| | `--| ,-- Sequoia_sempervirens

| | `--| ,-- Cryptomeria_japonica

| | `--| ,-- Cupressus_funebris

`--|	`----- Callitris_muelleri

| ,-- Araucaria_mirabilis

| ,----- Araucaria_heterophylla

| ,--| ,-- Wollemia_nobilis

| | `----- Agathis_australis

| | ,-- Lagarostrobos_franklinii

`--|	,----- Prumnopitys_taxifolia

| ,--| ,-- Lepidothamnus_laxifolius

| | `----- Phyllocladus_aspleniifolius

`--| ,-- Saxegothaea_conspicua

| | ,-- Pherosphaera_hookeriana

`--| ,----- Microcachrys_tetragona

`--| ,-- Acmopyle_pancheri

`--| ,-- Dacrydium_cupressinum

`--| ,-- Retrophyllum_rospigliosii

`----- Podocarpus_archboldii

Tree 43:

,-- Ginkgo_biloba

| ,-- Cedrus_deodara

| | ,-- Pseudotsuga_menziesii

| | ,----- Larix_decidua

| | ,--| ,-- Cathaya_argyrophylla

| ,--| | `--| ,-- Picea_abies

| | | | `--| ,-- Eathiestrobus_mackenziei

|--| | | | `----- Pinus_contorta

| | `--| ,-- Keteleeria_davidiana

| | | ,----- Abies_lasiocarpa

| | | | ,-- Pseudolarix_amabilis

| | `--| | ,-- Tsuga_canadensis

| | `--| ,----- Nothotsuga_longibracteata

| | `--| ,-- Schizolepidopsis_ediae

`--|	`--| ,-- Schizolepidopsis_daohugouensis

| `----- Schizolepidopsis_canicularis

| ,-- Mataia_podocarpoides

| | ,-- Araucaria_mirabilis

| | ,----- Araucaria_heterophylla

| | ,--| ,-- Wollemia_nobilis

| | | `----- Agathis_australis

| | | ,-- Lagarostrobos_franklinii

| | ,--| ,----- Prumnopitys_taxifolia

`--| | | ,--| ,-- Lepidothamnus_laxifolius

| | | | `----- Phyllocladus_aspleniifolius

| | `--| ,-- Saxegothaea_conspicua

| | `--| ,-- Microcachrys_tetragona

| | `--| ,-- Pherosphaera_hookeriana

| | `--| ,-- Acmopyle_pancheri

| | `--| ,-- Dacrydium_cupressinum

`--|	`--| ,-- Retrophyllum_rospigliosii

| `----- Podocarpus_archboldii

| ,-- Pararaucaria_patagonica

| | ,-- **New_total_plant_from_Arkansas**

| ,--| ,----- Alvinia_bohemica_plant

| | `--| ,-- Himeriella_muensteri

| | `--| ,-- Kachaikestrobus_acuminatus

| | `----- Tomaxellia_biforme

`--| ,-- Sciadopitys_verticillata

| | ,-- Cunninghamia_lanceolata

| | ,--| ,-- Taiwania_cryptomerioides

`--| | `--| ,-- Athrotaxis_selaginoides

| | `--| ,-- Sequoia_sempervirens

| | `--| ,-- Cryptomeria_japonica

`--|	`--| ,-- Cupressus_funebris

| `----- Callitris_muelleri

| ,-- Telemachus_aequatus

| | ,-- Nothodacrium_warrenii

`--| ,----- Rissikia_media

| | ,-- Voltzia_hexagona

`--| |	,-- Emporia_lockardii

`--| ,----- Hanskerpia_hamiltonensis

`--| ,-- Cordaixylon_dumusum

`----- Taxus_wallichiana

Tree 44:

,-- Ginkgo_biloba

| ,-- Cordaixylon_dumusum

|--| | ,-- Emporia_lockardii

| | ,----- Hanskerpia_hamiltonensis

`--| | ,-- Voltzia_hexagona

| | | ,-- Nothodacrium_warrenii

`--| | ,----- Rissikia_media

| | | ,-- Telemachus_aequatus

`--| | |	,-- Schizolepidopsis_ediae

| | | ,--| ,-- Schizolepidopsis_daohugouensis

| | | | `----- Schizolepidopsis_canicularis

`--| |	| ,-- Cedrus_deodara

| | ,--| | ,-- Keteleeria_davidiana

| | | | | ,----- Abies_lasiocarpa

| | | `--| ,--| ,-- Pseudolarix_amabilis

`--| |	| | `--| ,-- Mataia_podocarpoides

| | | | `--| ,-- Tsuga_canadensis

| | `--| `----- Nothotsuga_longibracteata

| | | ,-- Pseudotsuga_menziesii

| | | ,----- Larix_decidua

| | `--| ,-- Cathaya_argyrophylla

| | `--| ,-- Pinus_contorta

| | `--| ,-- Eathiestrobus_mackenziei

`--|	`----- Picea_abies

| ,-- Sciadopitys_verticillata

| ,--| ,-- Taxus_wallichiana

| | `--| ,-- Cunninghamia_lanceolata

| | `--| ,-- Taiwania_cryptomerioides

| | `--| ,-- Athrotaxis_selaginoides

| | `--| ,-- Sequoia_sempervirens

| | `--| ,-- Cryptomeria_japonica

| | `--| ,-- Cupressus_funebris

| | `----- Callitris_muelleri

`--|	,-- Pararaucaria_patagonica

| | ,-- **New_total_plant_from_Arkansas**

| ,--| ,----- Alvinia_bohemica_plant

| | `--| ,-- Himeriella_muensteri

| | `--| ,-- Kachaikestrobus_acuminatus

| | `----- Tomaxellia_biforme

| | ,-- Araucaria_mirabilis

`--|	,----- Araucaria_heterophylla

| ,--| ,-- Wollemia_nobilis

| | `----- Agathis_australis

| | ,-- Lagarostrobos_franklinii

`--|	,----- Prumnopitys_taxifolia

| ,--| ,-- Lepidothamnus_laxifolius

| | `----- Phyllocladus_aspleniifolius

`--| ,-- Saxegothaea_conspicua

`--| ,-- Microcachrys_tetragona

`--| ,-- Pherosphaera_hookeriana

`--| ,-- Acmopyle_pancheri

`--| ,-- Dacrydium_cupressinum

`--| ,-- Retrophyllum_rospigliosii

`----- Podocarpus_archboldii

Tree 45:

,-- Ginkgo_biloba

| ,-- Cordaixylon_dumusum

|--| | ,-- Emporia_lockardii

| | ,----- Hanskerpia_hamiltonensis

`--| | ,-- Voltzia_hexagona

| | | ,-- Nothodacrium_warrenii

`--| | ,----- Rissikia_media

| | | ,-- Telemachus_aequatus

`--| | |	,-- Schizolepidopsis_ediae

| | | ,--| ,-- Schizolepidopsis_daohugouensis

| | | | `----- Schizolepidopsis_canicularis

`--| |	| ,-- Cedrus_deodara

| | ,--| | ,-- Keteleeria_davidiana

| | | | | ,----- Abies_lasiocarpa

| | | `--| ,--| ,-- Pseudolarix_amabilis

`--| |	| | `--| ,-- Tsuga_canadensis

| | | | `--| ,-- Mataia_podocarpoides

| | `--| `----- Nothotsuga_longibracteata

| | | ,-- Pseudotsuga_menziesii

| | | ,----- Larix_decidua

| | `--| ,-- Cathaya_argyrophylla

| | `--| ,-- Pinus_contorta

| | `--| ,-- Eathiestrobus_mackenziei

`--|	`----- Picea_abies

| ,-- Sciadopitys_verticillata

| ,--| ,-- Taxus_wallichiana

| | `--| ,-- Cunninghamia_lanceolata

| | `--| ,-- Taiwania_cryptomerioides

| | `--| ,-- Athrotaxis_selaginoides

| | `--| ,-- Sequoia_sempervirens

| | `--| ,-- Cryptomeria_japonica

| | `--| ,-- Cupressus_funebris

| | `----- Callitris_muelleri

`--|	,-- Pararaucaria_patagonica

| | ,-- **New_total_plant_from_Arkansas**

| ,--| ,----- Alvinia_bohemica_plant

| | `--| ,-- Himeriella_muensteri

| | `--| ,-- Kachaikestrobus_acuminatus

| | `----- Tomaxellia_biforme

| | ,-- Araucaria_mirabilis

`--|	,----- Araucaria_heterophylla

| ,--| ,-- Wollemia_nobilis

| | `----- Agathis_australis

| | ,-- Lagarostrobos_franklinii

`--|	,----- Prumnopitys_taxifolia

| ,--| ,-- Lepidothamnus_laxifolius

| | `----- Phyllocladus_aspleniifolius

`--| ,-- Saxegothaea_conspicua

`--| ,-- Microcachrys_tetragona

`--| ,-- Pherosphaera_hookeriana

`--| ,-- Acmopyle_pancheri

`--| ,-- Dacrydium_cupressinum

`--| ,-- Retrophyllum_rospigliosii

`----- Podocarpus_archboldii

Tree 46:

,-- Ginkgo_biloba

| ,-- Cordaixylon_dumusum

|--| | ,-- Emporia_lockardii

| | ,----- Hanskerpia_hamiltonensis

`--| | ,-- Voltzia_hexagona

| | | ,-- Nothodacrium_warrenii

`--| | ,----- Rissikia_media

| | | ,-- Telemachus_aequatus

`--| | |	,-- Schizolepidopsis_ediae

| | | ,--| ,-- Schizolepidopsis_daohugouensis

| | | | `----- Schizolepidopsis_canicularis

`--| |	| ,-- Cedrus_deodara

| | ,--| | ,-- Keteleeria_davidiana

| | | | | ,----- Abies_lasiocarpa

| | | `--| ,--| ,-- Mataia_podocarpoides

`--| |	| | `--| ,-- Pseudolarix_amabilis

| | | | `--| ,-- Tsuga_canadensis

| | `--| `----- Nothotsuga_longibracteata

| | | ,-- Pseudotsuga_menziesii

| | | ,----- Larix_decidua

| | `--| ,-- Cathaya_argyrophylla

| | `--| ,-- Pinus_contorta

| | `--| ,-- Eathiestrobus_mackenziei

`--|	`----- Picea_abies

| ,-- Sciadopitys_verticillata

| ,--| ,-- Taxus_wallichiana

| | `--| ,-- Cunninghamia_lanceolata

| | `--| ,-- Taiwania_cryptomerioides

| | `--| ,-- Athrotaxis_selaginoides

| | `--| ,-- Sequoia_sempervirens

| | `--| ,-- Cryptomeria_japonica

| | `--| ,-- Cupressus_funebris

| | `----- Callitris_muelleri

`--|	,-- Pararaucaria_patagonica

| | ,-- **New_total_plant_from_Arkansas**

| ,--| ,----- Alvinia_bohemica_plant

| | `--| ,-- Himeriella_muensteri

| | `--| ,-- Kachaikestrobus_acuminatus

| | `----- Tomaxellia_biforme

| | ,-- Araucaria_mirabilis

`--|	,----- Araucaria_heterophylla

| ,--| ,-- Wollemia_nobilis

| | `----- Agathis_australis

| | ,-- Lagarostrobos_franklinii

`--|	,----- Prumnopitys_taxifolia

| ,--| ,-- Lepidothamnus_laxifolius

| | `----- Phyllocladus_aspleniifolius

`--| ,-- Saxegothaea_conspicua

`--| ,-- Microcachrys_tetragona

`--| ,-- Pherosphaera_hookeriana

`--| ,-- Acmopyle_pancheri

`--| ,-- Dacrydium_cupressinum

`--| ,-- Retrophyllum_rospigliosii

`----- Podocarpus_archboldii

Tree 47:

,-- Ginkgo_biloba

| ,-- Cordaixylon_dumusum

|--| | ,-- Emporia_lockardii

| | ,----- Hanskerpia_hamiltonensis

`--| | ,-- Voltzia_hexagona

| | | ,-- Nothodacrium_warrenii

`--| | ,----- Rissikia_media

| | | ,-- Pararaucaria_patagonica

`--| |	|	,-- **New_total_plant_from_Arkansas**

| | ,--| ,----- Alvinia_bohemica_plant

| | | `--| ,-- Himeriella_muensteri

`--| |	`--| ,-- Kachaikestrobus_acuminatus

| | `----- Tomaxellia_biforme

| | ,-- Telemachus_aequatus

| | | ,-- Eathiestrobus_mackenziei

`--| |	| ,-- Cedrus_deodara

| | ,--| | ,-- Keteleeria_davidiana

| | | | | ,----- Abies_lasiocarpa

| | | `--| ,--| ,-- Pseudolarix_amabilis

| | | | | `--| ,-- Mataia_podocarpoides

`--| |	| |	`--| ,-- Tsuga_canadensis

| | `--| `----- Nothotsuga_longibracteata

| | | ,-- Pseudotsuga_menziesii

| | | ,----- Larix_decidua

| | `--| ,-- Cathaya_argyrophylla

| | `--| ,-- Pinus_contorta

| | `--| ,-- Picea_abies

`--|	`--| ,-- Schizolepidopsis_ediae

| `--| ,-- Schizolepidopsis_daohugouensis

| `----- Schizolepidopsis_canicularis

| ,-- Sciadopitys_verticillata

| ,--| ,-- Taxus_wallichiana

| | `--| ,-- Cunninghamia_lanceolata

| | `--| ,-- Taiwania_cryptomerioides

| | `--| ,-- Athrotaxis_selaginoides

| | `--| ,-- Sequoia_sempervirens

| | `--| ,-- Cryptomeria_japonica

| | `--| ,-- Cupressus_funebris

`--|	`----- Callitris_muelleri

| ,-- Araucaria_mirabilis

| ,----- Araucaria_heterophylla

| ,--| ,-- Wollemia_nobilis

| | `----- Agathis_australis

| | ,-- Lagarostrobos_franklinii

`--|	,----- Prumnopitys_taxifolia

| ,--| ,-- Lepidothamnus_laxifolius

| | `----- Phyllocladus_aspleniifolius

`--| ,-- Saxegothaea_conspicua

`--| ,-- Microcachrys_tetragona

`--| ,-- Pherosphaera_hookeriana

`--| ,-- Acmopyle_pancheri

`--| ,-- Dacrydium_cupressinum

`--| ,-- Retrophyllum_rospigliosii

`----- Podocarpus_archboldii

Tree 48:

,-- Ginkgo_biloba

| ,-- Cordaixylon_dumusum

|--| | ,-- Emporia_lockardii

| | ,----- Hanskerpia_hamiltonensis

`--| | ,-- Voltzia_hexagona

| | | ,-- Nothodacrium_warrenii

`--| | ,----- Rissikia_media

| | | ,-- Pararaucaria_patagonica

`--| |	,--| ,-- **New_total_plant_from_Arkansas**

| | | `----- Alvinia_bohemica_plant

| | ,--| ,-- Himeriella_muensteri

`--| | `--| ,-- Kachaikestrobus_acuminatus

| | `----- Tomaxellia_biforme

| | ,-- Telemachus_aequatus

| | | ,-- Eathiestrobus_mackenziei

`--| |	| ,-- Cedrus_deodara

| | ,--| | ,-- Pseudotsuga_menziesii

| | | | | ,----- Larix_decidua

| | | `--| ,--| ,-- Cathaya_argyrophylla

| | | | | `--| ,-- Pinus_contorta

`--| |	| |	`----- Picea_abies

| | `--| ,-- Keteleeria_davidiana

| | | ,----- Abies_lasiocarpa

| | | | ,-- Pseudolarix_amabilis

| | `--| | ,-- Mataia_podocarpoides

| | `--| ,--| ,-- Tsuga_canadensis

| | | | `----- Nothotsuga_longibracteata

`--|	`--| ,-- Schizolepidopsis_ediae

| `--| ,-- Schizolepidopsis_daohugouensis

| `----- Schizolepidopsis_canicularis

| ,-- Sciadopitys_verticillata

| ,--| ,-- Taxus_wallichiana

| | `--| ,-- Cunninghamia_lanceolata

| | `--| ,-- Taiwania_cryptomerioides

| | `--| ,-- Athrotaxis_selaginoides

| | `--| ,-- Sequoia_sempervirens

| | `--| ,-- Cryptomeria_japonica

| | `--| ,-- Cupressus_funebris

`--|	`----- Callitris_muelleri

| ,-- Araucaria_mirabilis

| ,----- Araucaria_heterophylla

| ,--| ,-- Wollemia_nobilis

| | `----- Agathis_australis

| | ,-- Lagarostrobos_franklinii

`--|	,----- Prumnopitys_taxifolia

| ,--| ,-- Lepidothamnus_laxifolius

| | `----- Phyllocladus_aspleniifolius

`--| ,-- Saxegothaea_conspicua

`--| ,-- Microcachrys_tetragona

`--| ,-- Pherosphaera_hookeriana

`--| ,-- Acmopyle_pancheri

`--| ,-- Dacrydium_cupressinum

`--| ,-- Retrophyllum_rospigliosii

`----- Podocarpus_archboldii

Tree 49:

,-- Ginkgo_biloba

| ,-- Cordaixylon_dumusum

|--| | ,-- Emporia_lockardii

| | ,----- Hanskerpia_hamiltonensis

`--| | ,-- Voltzia_hexagona

| | | ,-- Nothodacrium_warrenii

`--| | ,----- Rissikia_media

| | | ,-- Pararaucaria_patagonica

`--| |	,--| ,-- **New_total_plant_from_Arkansas**

| | | `----- Alvinia_bohemica_plant

| | ,--| ,-- Himeriella_muensteri

`--| | `--| ,-- Kachaikestrobus_acuminatus

| | `----- Tomaxellia_biforme

| | ,-- Telemachus_aequatus

| | | ,-- Eathiestrobus_mackenziei

| | | | ,-- Cedrus_deodara

`--| |	| |	,-- Mataia_podocarpoides

| | ,--| | ,--| ,-- Keteleeria_davidiana

| | | | | | `----- Abies_lasiocarpa

| | | `--| ,--| ,-- Pseudolarix_amabilis

| | | | | `--| ,-- Tsuga_canadensis

`--| |	| |	`----- Nothotsuga_longibracteata

| | `--| ,-- Pseudotsuga_menziesii

| | | ,----- Larix_decidua

| | `--| ,-- Cathaya_argyrophylla

| | `--| ,-- Pinus_contorta

| | `--| ,-- Picea_abies

| | `--| ,-- Schizolepidopsis_ediae

`--|	`--| ,-- Schizolepidopsis_daohugouensis

| `----- Schizolepidopsis_canicularis

| ,-- Sciadopitys_verticillata

| ,--| ,-- Taxus_wallichiana

| | `--| ,-- Cunninghamia_lanceolata

| | `--| ,-- Taiwania_cryptomerioides

| | `--| ,-- Athrotaxis_selaginoides

| | `--| ,-- Sequoia_sempervirens

| | `--| ,-- Cryptomeria_japonica

| | `--| ,-- Cupressus_funebris

`--|	`----- Callitris_muelleri

| ,-- Araucaria_mirabilis

| ,----- Araucaria_heterophylla

| ,--| ,-- Wollemia_nobilis

| | `----- Agathis_australis

| | ,-- Lagarostrobos_franklinii

`--|	,----- Prumnopitys_taxifolia

| ,--| ,-- Lepidothamnus_laxifolius

| | `----- Phyllocladus_aspleniifolius

`--| ,-- Saxegothaea_conspicua

`--| ,-- Microcachrys_tetragona

`--| ,-- Pherosphaera_hookeriana

`--| ,-- Acmopyle_pancheri

`--| ,-- Dacrydium_cupressinum

`--| ,-- Retrophyllum_rospigliosii

`----- Podocarpus_archboldii

Tree 50:

,-- Ginkgo_biloba

| ,-- Cordaixylon_dumusum

|--| | ,-- Emporia_lockardii

| | ,----- Hanskerpia_hamiltonensis

`--| | ,-- Voltzia_hexagona

| | | ,-- Nothodacrium_warrenii

`--| | ,----- Rissikia_media

| | | ,-- Pararaucaria_patagonica

`--| |	,--| ,-- **New_total_plant_from_Arkansas**

| | | `----- Alvinia_bohemica_plant

| | ,--| ,-- Himeriella_muensteri

`--| | `--| ,-- Kachaikestrobus_acuminatus

| | `----- Tomaxellia_biforme

| | ,-- Telemachus_aequatus

| | | ,-- Eathiestrobus_mackenziei

`--| |	| ,-- Cedrus_deodara

| | ,--| | ,-- Keteleeria_davidiana

| | | | | ,----- Abies_lasiocarpa

| | | `--| ,--| ,-- Pseudolarix_amabilis

| | | | | `--| ,-- Mataia_podocarpoides

`--| |	| |	`--| ,-- Tsuga_canadensis

| | `--| `----- Nothotsuga_longibracteata

| | | ,-- Pseudotsuga_menziesii

| | | ,----- Larix_decidua

| | `--| ,-- Cathaya_argyrophylla

| | `--| ,-- Pinus_contorta

| | `--| ,-- Picea_abies

`--|	`--| ,-- Schizolepidopsis_ediae

| `--| ,-- Schizolepidopsis_daohugouensis

| `----- Schizolepidopsis_canicularis

| ,-- Sciadopitys_verticillata

| ,--| ,-- Taxus_wallichiana

| | `--| ,-- Cunninghamia_lanceolata

| | `--| ,-- Taiwania_cryptomerioides

| | `--| ,-- Athrotaxis_selaginoides

| | `--| ,-- Sequoia_sempervirens

| | `--| ,-- Cryptomeria_japonica

| | `--| ,-- Cupressus_funebris

`--|	`----- Callitris_muelleri

| ,-- Araucaria_mirabilis

| ,----- Araucaria_heterophylla

| ,--| ,-- Wollemia_nobilis

| | `----- Agathis_australis

| | ,-- Lagarostrobos_franklinii

`--|	,----- Prumnopitys_taxifolia

| ,--| ,-- Lepidothamnus_laxifolius

| | `----- Phyllocladus_aspleniifolius

`--| ,-- Saxegothaea_conspicua

| | ,-- Pherosphaera_hookeriana

`--| ,----- Microcachrys_tetragona

`--| ,-- Acmopyle_pancheri

`--| ,-- Dacrydium_cupressinum

`--| ,-- Retrophyllum_rospigliosii

`----- Podocarpus_archboldii

Tree 51:

,-- Ginkgo_biloba

| ,-- Cordaixylon_dumusum

|--| | ,-- Emporia_lockardii

| | ,----- Hanskerpia_hamiltonensis

`--| | ,-- Voltzia_hexagona

| | | ,-- Nothodacrium_warrenii

`--| | ,----- Rissikia_media

| | | ,-- Pararaucaria_patagonica

`--| |	|	,-- **New_total_plant_from_Arkansas**

| | ,--| ,----- Alvinia_bohemica_plant

| | | `--| ,-- Himeriella_muensteri

`--| |	`--| ,-- Kachaikestrobus_acuminatus

| | `----- Tomaxellia_biforme

| | ,-- Telemachus_aequatus

| | | ,-- Eathiestrobus_mackenziei

`--| |	| ,-- Cedrus_deodara

| | | | ,-- Keteleeria_davidiana

| | ,--| | ,--| ,-- Mataia_podocarpoides

| | | | | | `----- Abies_lasiocarpa

| | | `--| ,--| ,-- Pseudolarix_amabilis

| | | | | `--| ,-- Tsuga_canadensis

`--| |	| |	`----- Nothotsuga_longibracteata

| | `--| ,-- Pseudotsuga_menziesii

| | | ,----- Larix_decidua

| | `--| ,-- Cathaya_argyrophylla

| | `--| ,-- Pinus_contorta

| | `--| ,-- Picea_abies

| | `--| ,-- Schizolepidopsis_ediae

`--|	`--| ,-- Schizolepidopsis_daohugouensis

| `----- Schizolepidopsis_canicularis

| ,-- Sciadopitys_verticillata

| ,--| ,-- Taxus_wallichiana

| | `--| ,-- Cunninghamia_lanceolata

| | `--| ,-- Taiwania_cryptomerioides

| | `--| ,-- Athrotaxis_selaginoides

| | `--| ,-- Sequoia_sempervirens

| | `--| ,-- Cryptomeria_japonica

| | `--| ,-- Cupressus_funebris

`--|	`----- Callitris_muelleri

| ,-- Araucaria_mirabilis

| ,----- Araucaria_heterophylla

| ,--| ,-- Wollemia_nobilis

| | `----- Agathis_australis

| | ,-- Lagarostrobos_franklinii

`--|	,----- Prumnopitys_taxifolia

| ,--| ,-- Lepidothamnus_laxifolius

| | `----- Phyllocladus_aspleniifolius

`--| ,-- Saxegothaea_conspicua

| | ,-- Pherosphaera_hookeriana

`--| ,----- Microcachrys_tetragona

`--| ,-- Acmopyle_pancheri

`--| ,-- Dacrydium_cupressinum

`--| ,-- Retrophyllum_rospigliosii

`----- Podocarpus_archboldii

Tree 52:

,-- Ginkgo_biloba

| ,-- Cordaixylon_dumusum

|--| | ,-- Emporia_lockardii

| | ,----- Hanskerpia_hamiltonensis

`--| | ,-- Voltzia_hexagona

| | | ,-- Nothodacrium_warrenii

`--| | ,----- Rissikia_media

| | | ,-- Pararaucaria_patagonica

`--| |	|	,-- **New_total_plant_from_Arkansas**

| | ,--| ,----- Alvinia_bohemica_plant

| | | `--| ,-- Himeriella_muensteri

`--| |	`--| ,-- Kachaikestrobus_acuminatus

| | `----- Tomaxellia_biforme

| | ,-- Telemachus_aequatus

| | | ,-- Eathiestrobus_mackenziei

`--| |	| ,-- Cedrus_deodara

| | ,--| | ,-- Keteleeria_davidiana

| | | | | ,----- Abies_lasiocarpa

| | | `--| ,--| ,-- Pseudolarix_amabilis

| | | | | `--| ,-- Mataia_podocarpoides

`--| |	| |	`--| ,-- Tsuga_canadensis

| | `--| `----- Nothotsuga_longibracteata

| | | ,-- Pseudotsuga_menziesii

| | | ,----- Larix_decidua

| | `--| ,-- Cathaya_argyrophylla

| | `--| ,-- Pinus_contorta

| | `--| ,-- Picea_abies

`--|	`--| ,-- Schizolepidopsis_ediae

| `--| ,-- Schizolepidopsis_daohugouensis

| `----- Schizolepidopsis_canicularis

| ,-- Sciadopitys_verticillata

| ,--| ,-- Taxus_wallichiana

| | `--| ,-- Cunninghamia_lanceolata

| | `--| ,-- Taiwania_cryptomerioides

| | `--| ,-- Athrotaxis_selaginoides

| | `--| ,-- Sequoia_sempervirens

| | `--| ,-- Cryptomeria_japonica

| | `--| ,-- Cupressus_funebris

`--|	`----- Callitris_muelleri

| ,-- Araucaria_mirabilis

| ,----- Araucaria_heterophylla

| ,--| ,-- Wollemia_nobilis

| | `----- Agathis_australis

| | ,-- Lagarostrobos_franklinii

`--|	,----- Prumnopitys_taxifolia

| ,--| ,-- Lepidothamnus_laxifolius

| | `----- Phyllocladus_aspleniifolius

`--| ,-- Saxegothaea_conspicua

| | ,-- Pherosphaera_hookeriana

`--| ,----- Microcachrys_tetragona

`--| ,-- Acmopyle_pancheri

`--| ,-- Dacrydium_cupressinum

`--| ,-- Retrophyllum_rospigliosii

`----- Podocarpus_archboldii

Tree 53:

,-- Ginkgo_biloba

| ,-- Cordaixylon_dumusum

|--| | ,-- Emporia_lockardii

| | ,----- Hanskerpia_hamiltonensis

`--| | ,-- Voltzia_hexagona

| | | ,-- Nothodacrium_warrenii

`--| | ,----- Rissikia_media

| | | ,-- Pararaucaria_patagonica

`--| |	|	,-- **New_total_plant_from_Arkansas**

| | ,--| ,----- Alvinia_bohemica_plant

| | | `--| ,-- Himeriella_muensteri

`--| |	`--| ,-- Kachaikestrobus_acuminatus

| | `----- Tomaxellia_biforme

| | ,-- Telemachus_aequatus

| | | ,-- Eathiestrobus_mackenziei

`--| |	| ,-- Cedrus_deodara

| | | | ,-- Keteleeria_davidiana

| | ,--| | ,----- Abies_lasiocarpa

| | | | | | ,-- Mataia_podocarpoides

| | | `--| ,--| ,----- Pseudolarix_amabilis

| | | | | `--| ,-- Tsuga_canadensis

`--| |	| |	`----- Nothotsuga_longibracteata

| | `--| ,-- Pseudotsuga_menziesii

| | | ,----- Larix_decidua

| | `--| ,-- Cathaya_argyrophylla

| | `--| ,-- Pinus_contorta

| | `--| ,-- Picea_abies

| | `--| ,-- Schizolepidopsis_ediae

`--|	`--| ,-- Schizolepidopsis_daohugouensis

| `----- Schizolepidopsis_canicularis

| ,-- Sciadopitys_verticillata

| ,--| ,-- Taxus_wallichiana

| | `--| ,-- Cunninghamia_lanceolata

| | `--| ,-- Taiwania_cryptomerioides

| | `--| ,-- Athrotaxis_selaginoides

| | `--| ,-- Sequoia_sempervirens

| | `--| ,-- Cryptomeria_japonica

| | `--| ,-- Cupressus_funebris

`--|	`----- Callitris_muelleri

| ,-- Araucaria_mirabilis

| ,----- Araucaria_heterophylla

| ,--| ,-- Wollemia_nobilis

| | `----- Agathis_australis

| | ,-- Lagarostrobos_franklinii

`--|	,----- Prumnopitys_taxifolia

| ,--| ,-- Lepidothamnus_laxifolius

| | `----- Phyllocladus_aspleniifolius

`--| ,-- Saxegothaea_conspicua

`--| ,-- Microcachrys_tetragona

`--| ,-- Pherosphaera_hookeriana

`--| ,-- Acmopyle_pancheri

`--| ,-- Dacrydium_cupressinum

`--| ,-- Retrophyllum_rospigliosii

`----- Podocarpus_archboldii

Tree 54:

,-- Ginkgo_biloba

| ,-- Cordaixylon_dumusum

|--| | ,-- Emporia_lockardii

| | ,----- Hanskerpia_hamiltonensis

`--| | ,-- Voltzia_hexagona

| | | ,-- Nothodacrium_warrenii

`--| | ,----- Rissikia_media

| | | ,-- Alvinia_bohemica_plant

`--| |	,--| ,-- **New_total_plant_from_Arkansas**

| | | `----- Pararaucaria_patagonica

| | ,--| ,-- Himeriella_muensteri

`--| | `--| ,-- Kachaikestrobus_acuminatus

| | `----- Tomaxellia_biforme

| | ,-- Telemachus_aequatus

| | | ,-- Eathiestrobus_mackenziei

| | | | ,-- Cedrus_deodara

`--| |	| |	,-- Keteleeria_davidiana

| | ,--| | ,----- Abies_lasiocarpa

| | | | | | ,-- Mataia_podocarpoides

| | | `--| ,--| ,----- Pseudolarix_amabilis

| | | | | `--| ,-- Tsuga_canadensis

`--| |	| |	`----- Nothotsuga_longibracteata

| | `--| ,-- Pseudotsuga_menziesii

| | | ,----- Larix_decidua

| | `--| ,-- Cathaya_argyrophylla

| | `--| ,-- Pinus_contorta

| | `--| ,-- Picea_abies

| | `--| ,-- Schizolepidopsis_ediae

`--|	`--| ,-- Schizolepidopsis_daohugouensis

| `----- Schizolepidopsis_canicularis

| ,-- Sciadopitys_verticillata

| ,--| ,-- Taxus_wallichiana

| | `--| ,-- Cunninghamia_lanceolata

| | `--| ,-- Taiwania_cryptomerioides

| | `--| ,-- Athrotaxis_selaginoides

| | `--| ,-- Sequoia_sempervirens

| | `--| ,-- Cryptomeria_japonica

| | `--| ,-- Cupressus_funebris

`--|	`----- Callitris_muelleri

| ,-- Araucaria_mirabilis

| ,----- Araucaria_heterophylla

| ,--| ,-- Wollemia_nobilis

| | `----- Agathis_australis

| | ,-- Lagarostrobos_franklinii

`--|	,----- Prumnopitys_taxifolia

| ,--| ,-- Lepidothamnus_laxifolius

| | `----- Phyllocladus_aspleniifolius

`--| ,-- Saxegothaea_conspicua

| | ,-- Pherosphaera_hookeriana

`--| ,----- Microcachrys_tetragona

`--| ,-- Acmopyle_pancheri

`--| ,-- Dacrydium_cupressinum

`--| ,-- Retrophyllum_rospigliosii

`----- Podocarpus_archboldii

Tree 55:

,-- Ginkgo_biloba

| ,-- Cordaixylon_dumusum

|--| | ,-- Emporia_lockardii

| | ,----- Hanskerpia_hamiltonensis

`--| | ,-- Voltzia_hexagona

| | | ,-- Nothodacrium_warrenii

`--| | ,----- Rissikia_media

| | | ,-- Pararaucaria_patagonica

`--| |	|	,-- **New_total_plant_from_Arkansas**

| | ,--| ,----- Alvinia_bohemica_plant

| | | `--| ,-- Himeriella_muensteri

`--| |	`--| ,-- Kachaikestrobus_acuminatus

| | `----- Tomaxellia_biforme

| | ,-- Telemachus_aequatus

| | | ,-- Eathiestrobus_mackenziei

`--| |	| ,-- Cedrus_deodara

| | ,--| | ,-- Mataia_podocarpoides

| | | | | | ,-- Keteleeria_davidiana

| | | `--| ,--| ,----- Abies_lasiocarpa

| | | | | `--| ,-- Pseudolarix_amabilis

`--| |	| |	`--| ,-- Tsuga_canadensis

| | `--| `----- Nothotsuga_longibracteata

| | | ,-- Pseudotsuga_menziesii

| | | ,----- Larix_decidua

| | `--| ,-- Cathaya_argyrophylla

| | `--| ,-- Pinus_contorta

| | `--| ,-- Picea_abies

`--|	`--| ,-- Schizolepidopsis_ediae

| `--| ,-- Schizolepidopsis_daohugouensis

| `----- Schizolepidopsis_canicularis

| ,-- Sciadopitys_verticillata

| ,--| ,-- Taxus_wallichiana

| | `--| ,-- Cunninghamia_lanceolata

| | `--| ,-- Taiwania_cryptomerioides

| | `--| ,-- Athrotaxis_selaginoides

| | `--| ,-- Sequoia_sempervirens

| | `--| ,-- Cryptomeria_japonica

| | `--| ,-- Cupressus_funebris

`--|	`----- Callitris_muelleri

| ,-- Araucaria_mirabilis

| ,----- Araucaria_heterophylla

| ,--| ,-- Wollemia_nobilis

| | `----- Agathis_australis

| | ,-- Lagarostrobos_franklinii

`--|	,----- Prumnopitys_taxifolia

| ,--| ,-- Lepidothamnus_laxifolius

| | `----- Phyllocladus_aspleniifolius

`--| ,-- Saxegothaea_conspicua

| | ,-- Pherosphaera_hookeriana

`--| ,----- Microcachrys_tetragona

`--| ,-- Acmopyle_pancheri

`--| ,-- Dacrydium_cupressinum

`--| ,-- Retrophyllum_rospigliosii

`----- Podocarpus_archboldii

Tree 56:

,-- Ginkgo_biloba

| ,-- Cordaixylon_dumusum

|--| | ,-- Emporia_lockardii

| | ,----- Hanskerpia_hamiltonensis

`--| | ,-- Voltzia_hexagona

| | | ,-- Nothodacrium_warrenii

`--| | ,----- Rissikia_media

| | | ,-- Pararaucaria_patagonica

`--| |	|	,-- **New_total_plant_from_Arkansas**

| | ,--| ,----- Alvinia_bohemica_plant

| | | `--| ,-- Himeriella_muensteri

`--| |	`--| ,-- Kachaikestrobus_acuminatus

| | `----- Tomaxellia_biforme

| | ,-- Telemachus_aequatus

| | | ,-- Eathiestrobus_mackenziei

`--| |	| ,-- Cedrus_deodara

| | ,--| | ,-- Keteleeria_davidiana

| | | | | ,----- Abies_lasiocarpa

| | | `--| ,--| ,-- Mataia_podocarpoides

| | | | | `--| ,-- Pseudolarix_amabilis

`--| |	| |	`--| ,-- Tsuga_canadensis

| | `--| `----- Nothotsuga_longibracteata

| | | ,-- Pseudotsuga_menziesii

| | | ,----- Larix_decidua

| | `--| ,-- Cathaya_argyrophylla

| | `--| ,-- Pinus_contorta

| | `--| ,-- Picea_abies

`--|	`--| ,-- Schizolepidopsis_ediae

| `--| ,-- Schizolepidopsis_daohugouensis

| `----- Schizolepidopsis_canicularis

| ,-- Sciadopitys_verticillata

| ,--| ,-- Taxus_wallichiana

| | `--| ,-- Cunninghamia_lanceolata

| | `--| ,-- Taiwania_cryptomerioides

| | `--| ,-- Athrotaxis_selaginoides

| | `--| ,-- Sequoia_sempervirens

| | `--| ,-- Cryptomeria_japonica

| | `--| ,-- Cupressus_funebris

`--|	`----- Callitris_muelleri

| ,-- Araucaria_mirabilis

| ,----- Araucaria_heterophylla

| ,--| ,-- Wollemia_nobilis

| | `----- Agathis_australis

| | ,-- Lagarostrobos_franklinii

`--|	,----- Prumnopitys_taxifolia

| ,--| ,-- Lepidothamnus_laxifolius

| | `----- Phyllocladus_aspleniifolius

`--| ,-- Saxegothaea_conspicua

| | ,-- Pherosphaera_hookeriana

`--| ,----- Microcachrys_tetragona

`--| ,-- Acmopyle_pancheri

`--| ,-- Dacrydium_cupressinum

`--| ,-- Retrophyllum_rospigliosii

`----- Podocarpus_archboldii

Tree 57:

,-- Ginkgo_biloba

| ,-- Cordaixylon_dumusum

|--| | ,-- Emporia_lockardii

| | ,----- Hanskerpia_hamiltonensis

`--| | ,-- Voltzia_hexagona

| | | ,-- Nothodacrium_warrenii

`--| | ,----- Rissikia_media

| | | ,-- Pararaucaria_patagonica

`--| |	|	,-- **New_total_plant_from_Arkansas**

| | ,--| ,----- Alvinia_bohemica_plant

| | | `--| ,-- Himeriella_muensteri

`--| |	`--| ,-- Kachaikestrobus_acuminatus

| | `----- Tomaxellia_biforme

| | ,-- Telemachus_aequatus

| | | ,-- Eathiestrobus_mackenziei

`--| |	| ,-- Cedrus_deodara

| | | | ,-- Mataia_podocarpoides

| | ,--| | ,--| ,-- Keteleeria_davidiana

| | | | | | `----- Abies_lasiocarpa

| | | `--| ,--| ,-- Pseudolarix_amabilis

| | | | | `--| ,-- Tsuga_canadensis

`--| |	| |	`----- Nothotsuga_longibracteata

| | `--| ,-- Pseudotsuga_menziesii

| | | ,----- Larix_decidua

| | `--| ,-- Cathaya_argyrophylla

| | `--| ,-- Pinus_contorta

| | `--| ,-- Picea_abies

| | `--| ,-- Schizolepidopsis_ediae

`--|	`--| ,-- Schizolepidopsis_daohugouensis

| `----- Schizolepidopsis_canicularis

| ,-- Sciadopitys_verticillata

| ,--| ,-- Taxus_wallichiana

| | `--| ,-- Cunninghamia_lanceolata

| | `--| ,-- Taiwania_cryptomerioides

| | `--| ,-- Athrotaxis_selaginoides

| | `--| ,-- Sequoia_sempervirens

| | `--| ,-- Cryptomeria_japonica

| | `--| ,-- Cupressus_funebris

`--|	`----- Callitris_muelleri

| ,-- Araucaria_mirabilis

| ,----- Araucaria_heterophylla

| ,--| ,-- Wollemia_nobilis

| | `----- Agathis_australis

| | ,-- Lagarostrobos_franklinii

`--|	,----- Prumnopitys_taxifolia

| ,--| ,-- Lepidothamnus_laxifolius

| | `----- Phyllocladus_aspleniifolius

`--| ,-- Saxegothaea_conspicua

| | ,-- Pherosphaera_hookeriana

`--| ,----- Microcachrys_tetragona

`--| ,-- Acmopyle_pancheri

`--| ,-- Dacrydium_cupressinum

`--| ,-- Retrophyllum_rospigliosii

`----- Podocarpus_archboldii

Tree 58:

,-- Ginkgo_biloba

| ,-- Cordaixylon_dumusum

|--| | ,-- Emporia_lockardii

| | ,----- Hanskerpia_hamiltonensis

`--| | ,-- Voltzia_hexagona

| | | ,-- Nothodacrium_warrenii

`--| | ,----- Rissikia_media

| | | ,-- Pararaucaria_patagonica

`--| |	|	,-- **New_total_plant_from_Arkansas**

| | ,--| ,----- Alvinia_bohemica_plant

| | | `--| ,-- Himeriella_muensteri

`--| |	`--| ,-- Kachaikestrobus_acuminatus

| | `----- Tomaxellia_biforme

| | ,-- Telemachus_aequatus

| | | ,-- Eathiestrobus_mackenziei

`--| |	| ,-- Cedrus_deodara

| | ,--| | ,-- Keteleeria_davidiana

| | | | | ,----- Abies_lasiocarpa

| | | `--| ,--| ,-- Pseudolarix_amabilis

| | | | | `--| ,-- Tsuga_canadensis

`--| |	| |	`--| ,-- Mataia_podocarpoides

| | `--| `----- Nothotsuga_longibracteata

| | | ,-- Pseudotsuga_menziesii

| | | ,----- Larix_decidua

| | `--| ,-- Cathaya_argyrophylla

| | `--| ,-- Pinus_contorta

| | `--| ,-- Picea_abies

`--|	`--| ,-- Schizolepidopsis_ediae

| `--| ,-- Schizolepidopsis_daohugouensis

| `----- Schizolepidopsis_canicularis

| ,-- Sciadopitys_verticillata

| ,--| ,-- Taxus_wallichiana

| | `--| ,-- Cunninghamia_lanceolata

| | `--| ,-- Taiwania_cryptomerioides

| | `--| ,-- Athrotaxis_selaginoides

| | `--| ,-- Sequoia_sempervirens

| | `--| ,-- Cryptomeria_japonica

| | `--| ,-- Cupressus_funebris

`--|	`----- Callitris_muelleri

| ,-- Araucaria_mirabilis

| ,----- Araucaria_heterophylla

| ,--| ,-- Wollemia_nobilis

| | `----- Agathis_australis

| | ,-- Lagarostrobos_franklinii

`--|	,----- Prumnopitys_taxifolia

| ,--| ,-- Lepidothamnus_laxifolius

| | `----- Phyllocladus_aspleniifolius

`--| ,-- Saxegothaea_conspicua

| | ,-- Pherosphaera_hookeriana

`--| ,----- Microcachrys_tetragona

`--| ,-- Acmopyle_pancheri

`--| ,-- Dacrydium_cupressinum

`--| ,-- Retrophyllum_rospigliosii

`----- Podocarpus_archboldii

Tree 59:

,-- Ginkgo_biloba

| ,-- Cordaixylon_dumusum

|--| | ,-- Emporia_lockardii

| | ,----- Hanskerpia_hamiltonensis

`--| | ,-- Voltzia_hexagona

| | | ,-- Nothodacrium_warrenii

`--| | ,----- Rissikia_media

| | | ,-- Pararaucaria_patagonica

`--| |	,--| ,-- **New_total_plant_from_Arkansas**

| | | `----- Alvinia_bohemica_plant

| | ,--| ,-- Himeriella_muensteri

`--| | `--| ,-- Kachaikestrobus_acuminatus

| | `----- Tomaxellia_biforme

| | ,-- Telemachus_aequatus

| | | ,-- Eathiestrobus_mackenziei

| | | | ,-- Cedrus_deodara

`--| |	| |	,-- Keteleeria_davidiana

| | ,--| | ,----- Abies_lasiocarpa

| | | | | | ,-- Mataia_podocarpoides

| | | `--| ,--| ,----- Pseudolarix_amabilis

| | | | | `--| ,-- Tsuga_canadensis

`--| |	| |	`----- Nothotsuga_longibracteata

| | `--| ,-- Pseudotsuga_menziesii

| | | ,----- Larix_decidua

| | `--| ,-- Cathaya_argyrophylla

| | `--| ,-- Pinus_contorta

| | `--| ,-- Picea_abies

| | `--| ,-- Schizolepidopsis_ediae

`--|	`--| ,-- Schizolepidopsis_daohugouensis

| `----- Schizolepidopsis_canicularis

| ,-- Sciadopitys_verticillata

| ,--| ,-- Taxus_wallichiana

| | `--| ,-- Cunninghamia_lanceolata

| | `--| ,-- Taiwania_cryptomerioides

| | `--| ,-- Athrotaxis_selaginoides

| | `--| ,-- Sequoia_sempervirens

| | `--| ,-- Cryptomeria_japonica

| | `--| ,-- Cupressus_funebris

`--|	`----- Callitris_muelleri

| ,-- Araucaria_mirabilis

| ,----- Araucaria_heterophylla

| ,--| ,-- Wollemia_nobilis

| | `----- Agathis_australis

| | ,-- Lagarostrobos_franklinii

`--|	,----- Prumnopitys_taxifolia

| ,--| ,-- Lepidothamnus_laxifolius

| | `----- Phyllocladus_aspleniifolius

`--| ,-- Saxegothaea_conspicua

| | ,-- Pherosphaera_hookeriana

`--| ,----- Microcachrys_tetragona

`--| ,-- Acmopyle_pancheri

`--| ,-- Dacrydium_cupressinum

`--| ,-- Retrophyllum_rospigliosii

`----- Podocarpus_archboldii

Tree 60:

,-- Ginkgo_biloba

| ,-- Cordaixylon_dumusum

|--| | ,-- Emporia_lockardii

| | ,----- Hanskerpia_hamiltonensis

`--| | ,-- Voltzia_hexagona

| | | ,-- Nothodacrium_warrenii

`--| | ,----- Rissikia_media

| | | ,-- Alvinia_bohemica_plant

`--| |	,--| ,-- **New_total_plant_from_Arkansas**

| | | `----- Pararaucaria_patagonica

| | ,--| ,-- Himeriella_muensteri

`--| | `--| ,-- Kachaikestrobus_acuminatus

| | `----- Tomaxellia_biforme

| | ,-- Telemachus_aequatus

| | | ,-- Eathiestrobus_mackenziei

`--| |	| ,-- Cedrus_deodara

| | ,--| | ,-- Mataia_podocarpoides

| | | `--| | ,-- Keteleeria_davidiana

| | | | | ,----- Abies_lasiocarpa

| | | `--| ,--| ,-- Pseudolarix_amabilis

`--| |	| | `--| ,-- Tsuga_canadensis

| | | | `----- Nothotsuga_longibracteata

| | `--| ,-- Pseudotsuga_menziesii

| | | ,----- Larix_decidua

| | `--| ,-- Cathaya_argyrophylla

| | `--| ,-- Pinus_contorta

| | `--| ,-- Picea_abies

`--|	`--| ,-- Schizolepidopsis_ediae

| `--| ,-- Schizolepidopsis_daohugouensis

| `----- Schizolepidopsis_canicularis

| ,-- Sciadopitys_verticillata

| ,--| ,-- Taxus_wallichiana

| | `--| ,-- Cunninghamia_lanceolata

| | `--| ,-- Taiwania_cryptomerioides

| | `--| ,-- Athrotaxis_selaginoides

| | `--| ,-- Sequoia_sempervirens

| | `--| ,-- Cryptomeria_japonica

| | `--| ,-- Cupressus_funebris

`--|	`----- Callitris_muelleri

| ,-- Araucaria_mirabilis

| ,----- Araucaria_heterophylla

| ,--| ,-- Wollemia_nobilis

| | `----- Agathis_australis

| | ,-- Lagarostrobos_franklinii

`--|	,----- Prumnopitys_taxifolia

| ,--| ,-- Lepidothamnus_laxifolius

| | `----- Phyllocladus_aspleniifolius

`--| ,-- Saxegothaea_conspicua

| | ,-- Pherosphaera_hookeriana

`--| ,----- Microcachrys_tetragona

`--| ,-- Acmopyle_pancheri

`--| ,-- Dacrydium_cupressinum

`--| ,-- Retrophyllum_rospigliosii

`----- Podocarpus_archboldii

Tree 61:

,-- Ginkgo_biloba

| ,-- Cordaixylon_dumusum

|--| | ,-- Emporia_lockardii

| | ,----- Hanskerpia_hamiltonensis

`--| | ,-- Voltzia_hexagona

| | | ,-- Nothodacrium_warrenii

`--| | ,----- Rissikia_media

| | | ,-- Pararaucaria_patagonica

`--| |	,--| ,-- **New_total_plant_from_Arkansas**

| | | `----- Alvinia_bohemica_plant

| | ,--| ,-- Himeriella_muensteri

`--| | `--| ,-- Kachaikestrobus_acuminatus

| | `----- Tomaxellia_biforme

| | ,-- Telemachus_aequatus

| | | ,-- Eathiestrobus_mackenziei

`--| |	| ,-- Cedrus_deodara

| | ,--| | ,-- Mataia_podocarpoides

| | | `--| | ,-- Keteleeria_davidiana

| | | | | ,----- Abies_lasiocarpa

| | | `--| ,--| ,-- Pseudolarix_amabilis

`--| |	| | `--| ,-- Tsuga_canadensis

| | | | `----- Nothotsuga_longibracteata

| | `--| ,-- Pseudotsuga_menziesii

| | | ,----- Larix_decidua

| | `--| ,-- Cathaya_argyrophylla

| | `--| ,-- Pinus_contorta

| | `--| ,-- Picea_abies

`--|	`--| ,-- Schizolepidopsis_ediae

| `--| ,-- Schizolepidopsis_daohugouensis

| `----- Schizolepidopsis_canicularis

| ,-- Sciadopitys_verticillata

| ,--| ,-- Taxus_wallichiana

| | `--| ,-- Cunninghamia_lanceolata

| | `--| ,-- Taiwania_cryptomerioides

| | `--| ,-- Athrotaxis_selaginoides

| | `--| ,-- Sequoia_sempervirens

| | `--| ,-- Cryptomeria_japonica

| | `--| ,-- Cupressus_funebris

`--|	`----- Callitris_muelleri

| ,-- Araucaria_mirabilis

| ,----- Araucaria_heterophylla

| ,--| ,-- Wollemia_nobilis

| | `----- Agathis_australis

| | ,-- Lagarostrobos_franklinii

`--|	,----- Prumnopitys_taxifolia

| ,--| ,-- Lepidothamnus_laxifolius

| | `----- Phyllocladus_aspleniifolius

`--| ,-- Saxegothaea_conspicua

| | ,-- Pherosphaera_hookeriana

`--| ,----- Microcachrys_tetragona

`--| ,-- Acmopyle_pancheri

`--| ,-- Dacrydium_cupressinum

`--| ,-- Retrophyllum_rospigliosii

`----- Podocarpus_archboldii

Tree 62:

,-- Ginkgo_biloba

| ,-- Cordaixylon_dumusum

|--| | ,-- Emporia_lockardii

| | ,----- Hanskerpia_hamiltonensis

`--| | ,-- Voltzia_hexagona

| | | ,-- Nothodacrium_warrenii

`--| | ,----- Rissikia_media

| | | ,-- Pararaucaria_patagonica

`--| |	,--| ,-- **New_total_plant_from_Arkansas**

| | | `----- Alvinia_bohemica_plant

| | ,--| ,-- Himeriella_muensteri

`--| | `--| ,-- Kachaikestrobus_acuminatus

| | `----- Tomaxellia_biforme

| | ,-- Telemachus_aequatus

| | | ,-- Eathiestrobus_mackenziei

`--| |	| ,-- Cedrus_deodara

| | ,--| | ,-- Mataia_podocarpoides

| | | `--| | ,-- Keteleeria_davidiana

| | | | | ,----- Abies_lasiocarpa

| | | `--| ,--| ,-- Pseudolarix_amabilis

`--| |	| | `--| ,-- Tsuga_canadensis

| | | | `----- Nothotsuga_longibracteata

| | `--| ,-- Pseudotsuga_menziesii

| | | ,----- Larix_decidua

| | `--| ,-- Cathaya_argyrophylla

| | `--| ,-- Pinus_contorta

| | `--| ,-- Picea_abies

`--|	`--| ,-- Schizolepidopsis_ediae

| `--| ,-- Schizolepidopsis_daohugouensis

| `----- Schizolepidopsis_canicularis

| ,-- Sciadopitys_verticillata

| ,--| ,-- Taxus_wallichiana

| | `--| ,-- Cunninghamia_lanceolata

| | `--| ,-- Taiwania_cryptomerioides

| | `--| ,-- Athrotaxis_selaginoides

| | `--| ,-- Sequoia_sempervirens

| | `--| ,-- Cryptomeria_japonica

| | `--| ,-- Cupressus_funebris

`--|	`----- Callitris_muelleri

| ,-- Araucaria_mirabilis

| ,----- Araucaria_heterophylla

| ,--| ,-- Wollemia_nobilis

| | `----- Agathis_australis

| | ,-- Lagarostrobos_franklinii

`--|	,----- Prumnopitys_taxifolia

| ,--| ,-- Lepidothamnus_laxifolius

| | `----- Phyllocladus_aspleniifolius

`--| ,-- Saxegothaea_conspicua

`--| ,-- Microcachrys_tetragona

`--| ,-- Pherosphaera_hookeriana

`--| ,-- Acmopyle_pancheri

`--| ,-- Dacrydium_cupressinum

`--| ,-- Retrophyllum_rospigliosii

`----- Podocarpus_archboldii

Tree 63:

,-- Ginkgo_biloba

| ,-- Cordaixylon_dumusum

|--| | ,-- Emporia_lockardii

| | ,----- Hanskerpia_hamiltonensis

`--| | ,-- Voltzia_hexagona

| | | ,-- Nothodacrium_warrenii

`--| | ,----- Rissikia_media

| | | ,-- **New_total_plant_from_Arkansas**

`--| |	,--| ,-- Alvinia_bohemica_plant

| | | `----- Pararaucaria_patagonica

| | ,--| ,-- Himeriella_muensteri

`--| | `--| ,-- Kachaikestrobus_acuminatus

| | `----- Tomaxellia_biforme

| | ,-- Telemachus_aequatus

| | | ,-- Eathiestrobus_mackenziei

`--| |	| ,-- Cedrus_deodara

| | ,--| | ,-- Keteleeria_davidiana

| | | | | ,----- Abies_lasiocarpa

| | | `--| ,--| ,-- Mataia_podocarpoides

| | | | | `--| ,-- Pseudolarix_amabilis

`--| |	| |	`--| ,-- Tsuga_canadensis

| | `--| `----- Nothotsuga_longibracteata

| | | ,-- Pseudotsuga_menziesii

| | | ,----- Larix_decidua

| | `--| ,-- Cathaya_argyrophylla

| | `--| ,-- Pinus_contorta

| | `--| ,-- Picea_abies

`--|	`--| ,-- Schizolepidopsis_ediae

| `--| ,-- Schizolepidopsis_daohugouensis

| `----- Schizolepidopsis_canicularis

| ,-- Sciadopitys_verticillata

| ,--| ,-- Taxus_wallichiana

| | `--| ,-- Cunninghamia_lanceolata

| | `--| ,-- Taiwania_cryptomerioides

| | `--| ,-- Athrotaxis_selaginoides

| | `--| ,-- Sequoia_sempervirens

| | `--| ,-- Cryptomeria_japonica

| | `--| ,-- Cupressus_funebris

`--|	`----- Callitris_muelleri

| ,-- Araucaria_mirabilis

| ,----- Araucaria_heterophylla

| ,--| ,-- Wollemia_nobilis

| | `----- Agathis_australis

| | ,-- Lagarostrobos_franklinii

`--|	,----- Prumnopitys_taxifolia

| ,--| ,-- Lepidothamnus_laxifolius

| | `----- Phyllocladus_aspleniifolius

`--| ,-- Saxegothaea_conspicua

`--| ,-- Microcachrys_tetragona

`--| ,-- Pherosphaera_hookeriana

`--| ,-- Acmopyle_pancheri

`--| ,-- Dacrydium_cupressinum

`--| ,-- Retrophyllum_rospigliosii

`----- Podocarpus_archboldii

Tree 64:

,-- Ginkgo_biloba

| ,-- Cordaixylon_dumusum

|--| | ,-- Emporia_lockardii

| | ,----- Hanskerpia_hamiltonensis

`--| | ,-- Voltzia_hexagona

| | | ,-- Nothodacrium_warrenii

`--| | ,----- Rissikia_media

| | | ,-- **New_total_plant_from_Arkansas**

`--| |	,--| ,-- Alvinia_bohemica_plant

| | | `----- Pararaucaria_patagonica

| | ,--| ,-- Himeriella_muensteri

`--| | `--| ,-- Kachaikestrobus_acuminatus

| | `----- Tomaxellia_biforme

| | ,-- Telemachus_aequatus

| | | ,-- Eathiestrobus_mackenziei

| | | | ,-- Cedrus_deodara

`--| |	| |	,-- Keteleeria_davidiana

| | ,--| | ,--| ,-- Mataia_podocarpoides

| | | | | | `----- Abies_lasiocarpa

| | | `--| ,--| ,-- Pseudolarix_amabilis

| | | | | `--| ,-- Tsuga_canadensis

`--| |	| |	`----- Nothotsuga_longibracteata

| | `--| ,-- Pseudotsuga_menziesii

| | | ,----- Larix_decidua

| | `--| ,-- Cathaya_argyrophylla

| | `--| ,-- Pinus_contorta

| | `--| ,-- Picea_abies

| | `--| ,-- Schizolepidopsis_ediae

`--|	`--| ,-- Schizolepidopsis_daohugouensis

| `----- Schizolepidopsis_canicularis

| ,-- Sciadopitys_verticillata

| ,--| ,-- Taxus_wallichiana

| | `--| ,-- Cunninghamia_lanceolata

| | `--| ,-- Taiwania_cryptomerioides

| | `--| ,-- Athrotaxis_selaginoides

| | `--| ,-- Sequoia_sempervirens

| | `--| ,-- Cryptomeria_japonica

| | `--| ,-- Cupressus_funebris

`--|	`----- Callitris_muelleri

| ,-- Araucaria_mirabilis

| ,----- Araucaria_heterophylla

| ,--| ,-- Wollemia_nobilis

| | `----- Agathis_australis

| | ,-- Lagarostrobos_franklinii

`--|	,----- Prumnopitys_taxifolia

| ,--| ,-- Lepidothamnus_laxifolius

| | `----- Phyllocladus_aspleniifolius

`--| ,-- Saxegothaea_conspicua

`--| ,-- Microcachrys_tetragona

`--| ,-- Pherosphaera_hookeriana

`--| ,-- Acmopyle_pancheri

`--| ,-- Dacrydium_cupressinum

`--| ,-- Retrophyllum_rospigliosii

`----- Podocarpus_archboldii

Tree 65:

,-- Ginkgo_biloba

| ,-- Cordaixylon_dumusum

|--| | ,-- Emporia_lockardii

| | ,----- Hanskerpia_hamiltonensis

`--| | ,-- Voltzia_hexagona

| | | ,-- Nothodacrium_warrenii

`--| | ,----- Rissikia_media

| | | ,-- **New_total_plant_from_Arkansas**

`--| |	,--| ,-- Alvinia_bohemica_plant

| | | `----- Pararaucaria_patagonica

| | ,--| ,-- Himeriella_muensteri

`--| | `--| ,-- Kachaikestrobus_acuminatus

| | `----- Tomaxellia_biforme

| | ,-- Telemachus_aequatus

| | | ,-- Eathiestrobus_mackenziei

| | | | ,-- Cedrus_deodara

`--| |	| |	,-- Keteleeria_davidiana

| | ,--| | ,----- Abies_lasiocarpa

| | | | | | ,-- Mataia_podocarpoides

| | | `--| ,--| ,----- Pseudolarix_amabilis

| | | | | `--| ,-- Tsuga_canadensis

`--| |	| |	`----- Nothotsuga_longibracteata

| | `--| ,-- Pseudotsuga_menziesii

| | | ,----- Larix_decidua

| | `--| ,-- Cathaya_argyrophylla

| | `--| ,-- Pinus_contorta

| | `--| ,-- Picea_abies

| | `--| ,-- Schizolepidopsis_ediae

`--|	`--| ,-- Schizolepidopsis_daohugouensis

| `----- Schizolepidopsis_canicularis

| ,-- Sciadopitys_verticillata

| ,--| ,-- Taxus_wallichiana

| | `--| ,-- Cunninghamia_lanceolata

| | `--| ,-- Taiwania_cryptomerioides

| | `--| ,-- Athrotaxis_selaginoides

| | `--| ,-- Sequoia_sempervirens

| | `--| ,-- Cryptomeria_japonica

| | `--| ,-- Cupressus_funebris

`--|	`----- Callitris_muelleri

| ,-- Araucaria_mirabilis

| ,----- Araucaria_heterophylla

| ,--| ,-- Wollemia_nobilis

| | `----- Agathis_australis

| | ,-- Lagarostrobos_franklinii

`--|	,----- Prumnopitys_taxifolia

| ,--| ,-- Lepidothamnus_laxifolius

| | `----- Phyllocladus_aspleniifolius

`--| ,-- Saxegothaea_conspicua

`--| ,-- Microcachrys_tetragona

`--| ,-- Pherosphaera_hookeriana

`--| ,-- Acmopyle_pancheri

`--| ,-- Dacrydium_cupressinum

`--| ,-- Retrophyllum_rospigliosii

`----- Podocarpus_archboldii

Tree 66:

,-- Ginkgo_biloba

| ,-- Cordaixylon_dumusum

|--| | ,-- Emporia_lockardii

| | ,----- Hanskerpia_hamiltonensis

`--| | ,-- Voltzia_hexagona

| | | ,-- Nothodacrium_warrenii

`--| | ,----- Rissikia_media

| | | ,-- **New_total_plant_from_Arkansas**

`--| |	,--| ,-- Alvinia_bohemica_plant

| | | `----- Pararaucaria_patagonica

| | ,--| ,-- Himeriella_muensteri

`--| | `--| ,-- Kachaikestrobus_acuminatus

| | `----- Tomaxellia_biforme

| | ,-- Telemachus_aequatus

| | | ,-- Eathiestrobus_mackenziei

`--| |	| ,-- Cedrus_deodara

| | ,--| | ,-- Keteleeria_davidiana

| | | | | ,----- Abies_lasiocarpa

| | | `--| ,--| ,-- Pseudolarix_amabilis

| | | | | `--| ,-- Mataia_podocarpoides

`--| |	| |	`--| ,-- Tsuga_canadensis

| | `--| `----- Nothotsuga_longibracteata

| | | ,-- Pseudotsuga_menziesii

| | | ,----- Larix_decidua

| | `--| ,-- Cathaya_argyrophylla

| | `--| ,-- Pinus_contorta

| | `--| ,-- Picea_abies

`--|	`--| ,-- Schizolepidopsis_ediae

| `--| ,-- Schizolepidopsis_daohugouensis

| `----- Schizolepidopsis_canicularis

| ,-- Sciadopitys_verticillata

| ,--| ,-- Taxus_wallichiana

| | `--| ,-- Cunninghamia_lanceolata

| | `--| ,-- Taiwania_cryptomerioides

| | `--| ,-- Athrotaxis_selaginoides

| | `--| ,-- Sequoia_sempervirens

| | `--| ,-- Cryptomeria_japonica

| | `--| ,-- Cupressus_funebris

`--|	`----- Callitris_muelleri

| ,-- Araucaria_mirabilis

| ,----- Araucaria_heterophylla

| ,--| ,-- Wollemia_nobilis

| | `----- Agathis_australis

| | ,-- Lagarostrobos_franklinii

`--|	,----- Prumnopitys_taxifolia

| ,--| ,-- Lepidothamnus_laxifolius

| | `----- Phyllocladus_aspleniifolius

`--| ,-- Saxegothaea_conspicua

`--| ,-- Microcachrys_tetragona

`--| ,-- Pherosphaera_hookeriana

`--| ,-- Acmopyle_pancheri

`--| ,-- Dacrydium_cupressinum

`--| ,-- Retrophyllum_rospigliosii

`----- Podocarpus_archboldii

Tree 67:

,-- Ginkgo_biloba

| ,-- Cordaixylon_dumusum

|--| | ,-- Emporia_lockardii

| | ,----- Hanskerpia_hamiltonensis

`--| | ,-- Voltzia_hexagona

| | | ,-- Nothodacrium_warrenii

`--| | ,----- Rissikia_media

| | | ,-- **New_total_plant_from_Arkansas**

`--| |	,--| ,-- Alvinia_bohemica_plant

| | | `----- Pararaucaria_patagonica

| | ,--| ,-- Himeriella_muensteri

`--| | `--| ,-- Kachaikestrobus_acuminatus

| | `----- Tomaxellia_biforme

| | ,-- Telemachus_aequatus

| | | ,-- Eathiestrobus_mackenziei

`--| |	| ,-- Cedrus_deodara

| | ,--| | ,-- Keteleeria_davidiana

| | | | | ,----- Abies_lasiocarpa

| | | `--| ,--| ,-- Pseudolarix_amabilis

| | | | | `--| ,-- Tsuga_canadensis

`--| |	| |	`--| ,-- Mataia_podocarpoides

| | `--| `----- Nothotsuga_longibracteata

| | | ,-- Pseudotsuga_menziesii

| | | ,----- Larix_decidua

| | `--| ,-- Cathaya_argyrophylla

| | `--| ,-- Pinus_contorta

| | `--| ,-- Picea_abies

`--|	`--| ,-- Schizolepidopsis_ediae

| `--| ,-- Schizolepidopsis_daohugouensis

| `----- Schizolepidopsis_canicularis

| ,-- Sciadopitys_verticillata

| ,--| ,-- Taxus_wallichiana

| | `--| ,-- Cunninghamia_lanceolata

| | `--| ,-- Taiwania_cryptomerioides

| | `--| ,-- Athrotaxis_selaginoides

| | `--| ,-- Sequoia_sempervirens

| | `--| ,-- Cryptomeria_japonica

| | `--| ,-- Cupressus_funebris

`--|	`----- Callitris_muelleri

| ,-- Araucaria_mirabilis

| ,----- Araucaria_heterophylla

| ,--| ,-- Wollemia_nobilis

| | `----- Agathis_australis

| | ,-- Lagarostrobos_franklinii

`--|	,----- Prumnopitys_taxifolia

| ,--| ,-- Lepidothamnus_laxifolius

| | `----- Phyllocladus_aspleniifolius

`--| ,-- Saxegothaea_conspicua

`--| ,-- Microcachrys_tetragona

`--| ,-- Pherosphaera_hookeriana

`--| ,-- Acmopyle_pancheri

`--| ,-- Dacrydium_cupressinum

`--| ,-- Retrophyllum_rospigliosii

`----- Podocarpus_archboldii

Tree 68:

,-- Ginkgo_biloba

| ,-- Cordaixylon_dumusum

|--| | ,-- Emporia_lockardii

| | ,----- Hanskerpia_hamiltonensis

`--| | ,-- Voltzia_hexagona

| | | ,-- Nothodacrium_warrenii

`--| | ,----- Rissikia_media

| | | ,-- **New_total_plant_from_Arkansas**

`--| |	,--| ,-- Alvinia_bohemica_plant

| | | `----- Pararaucaria_patagonica

| | ,--| ,-- Himeriella_muensteri

`--| | `--| ,-- Kachaikestrobus_acuminatus

| | `----- Tomaxellia_biforme

| | ,-- Telemachus_aequatus

| | | ,-- Eathiestrobus_mackenziei

`--| |	| ,-- Cedrus_deodara

| | ,--| | ,-- Mataia_podocarpoides

| | | | | | ,-- Keteleeria_davidiana

| | | `--| ,--| ,----- Abies_lasiocarpa

| | | | | `--| ,-- Pseudolarix_amabilis

`--| |	| |	`--| ,-- Tsuga_canadensis

| | `--| `----- Nothotsuga_longibracteata

| | | ,-- Pseudotsuga_menziesii

| | | ,----- Larix_decidua

| | `--| ,-- Cathaya_argyrophylla

| | `--| ,-- Pinus_contorta

| | `--| ,-- Picea_abies

`--|	`--| ,-- Schizolepidopsis_ediae

| `--| ,-- Schizolepidopsis_daohugouensis

| `----- Schizolepidopsis_canicularis

| ,-- Sciadopitys_verticillata

| ,--| ,-- Taxus_wallichiana

| | `--| ,-- Cunninghamia_lanceolata

| | `--| ,-- Taiwania_cryptomerioides

| | `--| ,-- Athrotaxis_selaginoides

| | `--| ,-- Sequoia_sempervirens

| | `--| ,-- Cryptomeria_japonica

| | `--| ,-- Cupressus_funebris

`--|	`----- Callitris_muelleri

| ,-- Araucaria_mirabilis

| ,----- Araucaria_heterophylla

| ,--| ,-- Wollemia_nobilis

| | `----- Agathis_australis

| | ,-- Lagarostrobos_franklinii

`--|	,----- Prumnopitys_taxifolia

| ,--| ,-- Lepidothamnus_laxifolius

| | `----- Phyllocladus_aspleniifolius

`--| ,-- Saxegothaea_conspicua

`--| ,-- Microcachrys_tetragona

`--| ,-- Pherosphaera_hookeriana

`--| ,-- Acmopyle_pancheri

`--| ,-- Dacrydium_cupressinum

`--| ,-- Retrophyllum_rospigliosii

`----- Podocarpus_archboldii

Tree 69:

,-- Ginkgo_biloba

| ,-- Cordaixylon_dumusum

|--| | ,-- Emporia_lockardii

| | ,----- Hanskerpia_hamiltonensis

`--| | ,-- Voltzia_hexagona

| | | ,-- Nothodacrium_warrenii

`--| | ,----- Rissikia_media

| | | ,-- Pararaucaria_patagonica

`--| |	|	,-- **New_total_plant_from_Arkansas**

| | ,--| ,----- Alvinia_bohemica_plant

| | | `--| ,-- Himeriella_muensteri

`--| |	`--| ,-- Kachaikestrobus_acuminatus

| | `----- Tomaxellia_biforme

| | ,-- Telemachus_aequatus

| | | ,-- Eathiestrobus_mackenziei

`--| |	| ,-- Cedrus_deodara

| | ,--| | ,-- Keteleeria_davidiana

| | | | | ,----- Abies_lasiocarpa

| | | `--| ,--| ,-- Mataia_podocarpoides

| | | | | `--| ,-- Pseudolarix_amabilis

`--| |	| |	`--| ,-- Tsuga_canadensis

| | `--| `----- Nothotsuga_longibracteata

| | | ,-- Pseudotsuga_menziesii

| | | ,----- Larix_decidua

| | `--| ,-- Cathaya_argyrophylla

| | `--| ,-- Pinus_contorta

| | `--| ,-- Picea_abies

`--|	`--| ,-- Schizolepidopsis_ediae

| `--| ,-- Schizolepidopsis_daohugouensis

| `----- Schizolepidopsis_canicularis

| ,-- Sciadopitys_verticillata

| ,--| ,-- Taxus_wallichiana

| | `--| ,-- Cunninghamia_lanceolata

| | `--| ,-- Taiwania_cryptomerioides

| | `--| ,-- Athrotaxis_selaginoides

| | `--| ,-- Sequoia_sempervirens

| | `--| ,-- Cryptomeria_japonica

| | `--| ,-- Cupressus_funebris

`--|	`----- Callitris_muelleri

| ,-- Araucaria_mirabilis

| ,----- Araucaria_heterophylla

| ,--| ,-- Wollemia_nobilis

| | `----- Agathis_australis

| | ,-- Lagarostrobos_franklinii

`--|	,----- Prumnopitys_taxifolia

| ,--| ,-- Lepidothamnus_laxifolius

| | `----- Phyllocladus_aspleniifolius

`--| ,-- Saxegothaea_conspicua

`--| ,-- Microcachrys_tetragona

`--| ,-- Pherosphaera_hookeriana

`--| ,-- Acmopyle_pancheri

`--| ,-- Dacrydium_cupressinum

`--| ,-- Retrophyllum_rospigliosii

`----- Podocarpus_archboldii

Tree 70:

,-- Ginkgo_biloba

| ,-- Cordaixylon_dumusum

|--| | ,-- Emporia_lockardii

| | ,----- Hanskerpia_hamiltonensis

`--| | ,-- Voltzia_hexagona

| | | ,-- Nothodacrium_warrenii

`--| | ,----- Rissikia_media

| | | ,-- Pararaucaria_patagonica

`--| |	|	,-- **New_total_plant_from_Arkansas**

| | ,--| ,----- Alvinia_bohemica_plant

| | | `--| ,-- Himeriella_muensteri

`--| |	`--| ,-- Kachaikestrobus_acuminatus

| | `----- Tomaxellia_biforme

| | ,-- Telemachus_aequatus

| | | ,-- Eathiestrobus_mackenziei

`--| |	| ,-- Cedrus_deodara

| | | | ,-- Keteleeria_davidiana

| | ,--| | ,--| ,-- Mataia_podocarpoides

| | | | | | `----- Abies_lasiocarpa

| | | `--| ,--| ,-- Pseudolarix_amabilis

| | | | | `--| ,-- Tsuga_canadensis

`--| |	| |	`----- Nothotsuga_longibracteata

| | `--| ,-- Pseudotsuga_menziesii

| | | ,----- Larix_decidua

| | `--| ,-- Cathaya_argyrophylla

| | `--| ,-- Pinus_contorta

| | `--| ,-- Picea_abies

| | `--| ,-- Schizolepidopsis_ediae

`--|	`--| ,-- Schizolepidopsis_daohugouensis

| `----- Schizolepidopsis_canicularis

| ,-- Sciadopitys_verticillata

| ,--| ,-- Taxus_wallichiana

| | `--| ,-- Cunninghamia_lanceolata

| | `--| ,-- Taiwania_cryptomerioides

| | `--| ,-- Athrotaxis_selaginoides

| | `--| ,-- Sequoia_sempervirens

| | `--| ,-- Cryptomeria_japonica

| | `--| ,-- Cupressus_funebris

`--|	`----- Callitris_muelleri

| ,-- Araucaria_mirabilis

| ,----- Araucaria_heterophylla

| ,--| ,-- Wollemia_nobilis

| | `----- Agathis_australis

| | ,-- Lagarostrobos_franklinii

`--|	,----- Prumnopitys_taxifolia

| ,--| ,-- Lepidothamnus_laxifolius

| | `----- Phyllocladus_aspleniifolius

`--| ,-- Saxegothaea_conspicua

`--| ,-- Microcachrys_tetragona

`--| ,-- Pherosphaera_hookeriana

`--| ,-- Acmopyle_pancheri

`--| ,-- Dacrydium_cupressinum

`--| ,-- Retrophyllum_rospigliosii

`----- Podocarpus_archboldii

Tree 71:

,-- Ginkgo_biloba

| ,-- Cordaixylon_dumusum

|--| | ,-- Emporia_lockardii

| | ,----- Hanskerpia_hamiltonensis

`--| | ,-- Voltzia_hexagona

| | | ,-- Nothodacrium_warrenii

`--| | ,----- Rissikia_media

| | | ,-- Pararaucaria_patagonica

`--| |	|	,-- **New_total_plant_from_Arkansas**

| | ,--| ,----- Alvinia_bohemica_plant

| | | `--| ,-- Himeriella_muensteri

`--| |	`--| ,-- Kachaikestrobus_acuminatus

| | `----- Tomaxellia_biforme

| | ,-- Telemachus_aequatus

| | | ,-- Eathiestrobus_mackenziei

`--| |	| ,-- Cedrus_deodara

| | ,--| | ,-- Keteleeria_davidiana

| | | | | ,----- Abies_lasiocarpa

| | | `--| ,--| ,-- Pseudolarix_amabilis

| | | | | `--| ,-- Tsuga_canadensis

`--| |	| |	`--| ,-- Mataia_podocarpoides

| | `--| `----- Nothotsuga_longibracteata

| | | ,-- Pseudotsuga_menziesii

| | | ,----- Larix_decidua

| | `--| ,-- Cathaya_argyrophylla

| | `--| ,-- Pinus_contorta

| | `--| ,-- Picea_abies

`--|	`--| ,-- Schizolepidopsis_ediae

| `--| ,-- Schizolepidopsis_daohugouensis

| `----- Schizolepidopsis_canicularis

| ,-- Sciadopitys_verticillata

| ,--| ,-- Taxus_wallichiana

| | `--| ,-- Cunninghamia_lanceolata

| | `--| ,-- Taiwania_cryptomerioides

| | `--| ,-- Athrotaxis_selaginoides

| | `--| ,-- Sequoia_sempervirens

| | `--| ,-- Cryptomeria_japonica

| | `--| ,-- Cupressus_funebris

`--|	`----- Callitris_muelleri

| ,-- Araucaria_mirabilis

| ,----- Araucaria_heterophylla

| ,--| ,-- Wollemia_nobilis

| | `----- Agathis_australis

| | ,-- Lagarostrobos_franklinii

`--|	,----- Prumnopitys_taxifolia

| ,--| ,-- Lepidothamnus_laxifolius

| | `----- Phyllocladus_aspleniifolius

`--| ,-- Saxegothaea_conspicua

`--| ,-- Microcachrys_tetragona

`--| ,-- Pherosphaera_hookeriana

`--| ,-- Acmopyle_pancheri

`--| ,-- Dacrydium_cupressinum

`--| ,-- Retrophyllum_rospigliosii

`----- Podocarpus_archboldii

Tree 72:

,-- Ginkgo_biloba

| ,-- Cordaixylon_dumusum

|--| | ,-- Emporia_lockardii

| | ,----- Hanskerpia_hamiltonensis

`--| | ,-- Voltzia_hexagona

| | | ,-- Nothodacrium_warrenii

`--| | ,----- Rissikia_media

| | | ,-- Pararaucaria_patagonica

`--| |	,--| ,-- **New_total_plant_from_Arkansas**

| | | `----- Alvinia_bohemica_plant

| | ,--| ,-- Himeriella_muensteri

`--| | `--| ,-- Kachaikestrobus_acuminatus

| | `----- Tomaxellia_biforme

| | ,-- Telemachus_aequatus

| | | ,-- Eathiestrobus_mackenziei

`--| |	| ,-- Cedrus_deodara

| | ,--| | ,-- Mataia_podocarpoides

| | | | | | ,-- Keteleeria_davidiana

| | | `--| ,--| ,----- Abies_lasiocarpa

| | | | | `--| ,-- Pseudolarix_amabilis

`--| |	| |	`--| ,-- Tsuga_canadensis

| | `--| `----- Nothotsuga_longibracteata

| | | ,-- Pseudotsuga_menziesii

| | | ,----- Larix_decidua

| | `--| ,-- Cathaya_argyrophylla

| | `--| ,-- Pinus_contorta

| | `--| ,-- Picea_abies

`--|	`--| ,-- Schizolepidopsis_ediae

| `--| ,-- Schizolepidopsis_daohugouensis

| `----- Schizolepidopsis_canicularis

| ,-- Sciadopitys_verticillata

| ,--| ,-- Taxus_wallichiana

| | `--| ,-- Cunninghamia_lanceolata

| | `--| ,-- Taiwania_cryptomerioides

| | `--| ,-- Athrotaxis_selaginoides

| | `--| ,-- Sequoia_sempervirens

| | `--| ,-- Cryptomeria_japonica

| | `--| ,-- Cupressus_funebris

`--|	`----- Callitris_muelleri

| ,-- Araucaria_mirabilis

| ,----- Araucaria_heterophylla

| ,--| ,-- Wollemia_nobilis

| | `----- Agathis_australis

| | ,-- Lagarostrobos_franklinii

`--|	,----- Prumnopitys_taxifolia

| ,--| ,-- Lepidothamnus_laxifolius

| | `----- Phyllocladus_aspleniifolius

`--| ,-- Saxegothaea_conspicua

| | ,-- Pherosphaera_hookeriana

`--| ,----- Microcachrys_tetragona

`--| ,-- Acmopyle_pancheri

`--| ,-- Dacrydium_cupressinum

`--| ,-- Retrophyllum_rospigliosii

`----- Podocarpus_archboldii

Tree 73:

,-- Ginkgo_biloba

| ,-- Cordaixylon_dumusum

|--| | ,-- Emporia_lockardii

| | ,----- Hanskerpia_hamiltonensis

`--| | ,-- Voltzia_hexagona

| | | ,-- Nothodacrium_warrenii

`--| | ,----- Rissikia_media

| | | ,-- **New_total_plant_from_Arkansas**

`--| |	,--| ,-- Alvinia_bohemica_plant

| | | `----- Pararaucaria_patagonica

| | ,--| ,-- Himeriella_muensteri

`--| | `--| ,-- Kachaikestrobus_acuminatus

| | `----- Tomaxellia_biforme

| | ,-- Telemachus_aequatus

| | | ,-- Eathiestrobus_mackenziei

| | | | ,-- Cedrus_deodara

`--| |	| |	,-- Keteleeria_davidiana

| | ,--| | ,--| ,-- Mataia_podocarpoides

| | | | | | `----- Abies_lasiocarpa

| | | `--| ,--| ,-- Pseudolarix_amabilis

| | | | | `--| ,-- Tsuga_canadensis

`--| |	| |	`----- Nothotsuga_longibracteata

| | `--| ,-- Pseudotsuga_menziesii

| | | ,----- Larix_decidua

| | `--| ,-- Cathaya_argyrophylla

| | `--| ,-- Pinus_contorta

| | `--| ,-- Picea_abies

| | `--| ,-- Schizolepidopsis_ediae

`--|	`--| ,-- Schizolepidopsis_daohugouensis

| `----- Schizolepidopsis_canicularis

| ,-- Sciadopitys_verticillata

| ,--| ,-- Taxus_wallichiana

| | `--| ,-- Cunninghamia_lanceolata

| | `--| ,-- Taiwania_cryptomerioides

| | `--| ,-- Athrotaxis_selaginoides

| | `--| ,-- Sequoia_sempervirens

| | `--| ,-- Cryptomeria_japonica

| | `--| ,-- Cupressus_funebris

`--|	`----- Callitris_muelleri

| ,-- Araucaria_mirabilis

| ,----- Araucaria_heterophylla

| ,--| ,-- Wollemia_nobilis

| | `----- Agathis_australis

| | ,-- Lagarostrobos_franklinii

`--|	,----- Prumnopitys_taxifolia

| ,--| ,-- Lepidothamnus_laxifolius

| | `----- Phyllocladus_aspleniifolius

`--| ,-- Saxegothaea_conspicua

| | ,-- Pherosphaera_hookeriana

`--| ,----- Microcachrys_tetragona

`--| ,-- Acmopyle_pancheri

`--| ,-- Dacrydium_cupressinum

`--| ,-- Retrophyllum_rospigliosii

`----- Podocarpus_archboldii

Tree 74:

,-- Ginkgo_biloba

| ,-- Cordaixylon_dumusum

|--| | ,-- Emporia_lockardii

| | ,----- Hanskerpia_hamiltonensis

`--| | ,-- Voltzia_hexagona

| | | ,-- Nothodacrium_warrenii

`--| | ,----- Rissikia_media

| | | ,-- **New_total_plant_from_Arkansas**

`--| |	,--| ,-- Alvinia_bohemica_plant

| | | `----- Pararaucaria_patagonica

| | ,--| ,-- Himeriella_muensteri

`--| | `--| ,-- Kachaikestrobus_acuminatus

| | `----- Tomaxellia_biforme

| | ,-- Telemachus_aequatus

| | | ,-- Eathiestrobus_mackenziei

`--| |	| ,-- Cedrus_deodara

| | ,--| | ,-- Keteleeria_davidiana

| | | | | ,----- Abies_lasiocarpa

| | | `--| ,--| ,-- Pseudolarix_amabilis

| | | | | `--| ,-- Mataia_podocarpoides

`--| |	| |	`--| ,-- Tsuga_canadensis

| | `--| `----- Nothotsuga_longibracteata

| | | ,-- Pseudotsuga_menziesii

| | | ,----- Larix_decidua

| | `--| ,-- Cathaya_argyrophylla

| | `--| ,-- Pinus_contorta

| | `--| ,-- Picea_abies

`--|	`--| ,-- Schizolepidopsis_ediae

| `--| ,-- Schizolepidopsis_daohugouensis

| `----- Schizolepidopsis_canicularis

| ,-- Sciadopitys_verticillata

| ,--| ,-- Taxus_wallichiana

| | `--| ,-- Cunninghamia_lanceolata

| | `--| ,-- Taiwania_cryptomerioides

| | `--| ,-- Athrotaxis_selaginoides

| | `--| ,-- Sequoia_sempervirens

| | `--| ,-- Cryptomeria_japonica

| | `--| ,-- Cupressus_funebris

`--|	`----- Callitris_muelleri

| ,-- Araucaria_mirabilis

| ,----- Araucaria_heterophylla

| ,--| ,-- Wollemia_nobilis

| | `----- Agathis_australis

| | ,-- Lagarostrobos_franklinii

`--|	,----- Prumnopitys_taxifolia

| ,--| ,-- Lepidothamnus_laxifolius

| | `----- Phyllocladus_aspleniifolius

`--| ,-- Saxegothaea_conspicua

| | ,-- Pherosphaera_hookeriana

`--| ,----- Microcachrys_tetragona

`--| ,-- Acmopyle_pancheri

`--| ,-- Dacrydium_cupressinum

`--| ,-- Retrophyllum_rospigliosii

`----- Podocarpus_archboldii

Tree 75:

,-- Ginkgo_biloba

| ,-- Cordaixylon_dumusum

|--| | ,-- Emporia_lockardii

| | ,----- Hanskerpia_hamiltonensis

`--| | ,-- Voltzia_hexagona

| | | ,-- Nothodacrium_warrenii

`--| | ,----- Rissikia_media

| | | ,-- **New_total_plant_from_Arkansas**

`--| |	,--| ,-- Alvinia_bohemica_plant

| | | `----- Pararaucaria_patagonica

| | ,--| ,-- Himeriella_muensteri

`--| | `--| ,-- Kachaikestrobus_acuminatus

| | `----- Tomaxellia_biforme

| | ,-- Telemachus_aequatus

| | | ,-- Eathiestrobus_mackenziei

`--| |	| ,-- Cedrus_deodara

| | ,--| | ,-- Keteleeria_davidiana

| | | | | ,----- Abies_lasiocarpa

| | | `--| ,--| ,-- Pseudolarix_amabilis

| | | | | `--| ,-- Tsuga_canadensis

`--| |	| |	`--| ,-- Mataia_podocarpoides

| | `--| `----- Nothotsuga_longibracteata

| | | ,-- Pseudotsuga_menziesii

| | | ,----- Larix_decidua

| | `--| ,-- Cathaya_argyrophylla

| | `--| ,-- Pinus_contorta

| | `--| ,-- Picea_abies

`--|	`--| ,-- Schizolepidopsis_ediae

| `--| ,-- Schizolepidopsis_daohugouensis

| `----- Schizolepidopsis_canicularis

| ,-- Sciadopitys_verticillata

| ,--| ,-- Taxus_wallichiana

| | `--| ,-- Cunninghamia_lanceolata

| | `--| ,-- Taiwania_cryptomerioides

| | `--| ,-- Athrotaxis_selaginoides

| | `--| ,-- Sequoia_sempervirens

| | `--| ,-- Cryptomeria_japonica

| | `--| ,-- Cupressus_funebris

`--|	`----- Callitris_muelleri

| ,-- Araucaria_mirabilis

| ,----- Araucaria_heterophylla

| ,--| ,-- Wollemia_nobilis

| | `----- Agathis_australis

| | ,-- Lagarostrobos_franklinii

`--|	,----- Prumnopitys_taxifolia

| ,--| ,-- Lepidothamnus_laxifolius

| | `----- Phyllocladus_aspleniifolius

`--| ,-- Saxegothaea_conspicua

| | ,-- Pherosphaera_hookeriana

`--| ,----- Microcachrys_tetragona

`--| ,-- Acmopyle_pancheri

`--| ,-- Dacrydium_cupressinum

`--| ,-- Retrophyllum_rospigliosii

`----- Podocarpus_archboldii

Tree 76:

,-- Ginkgo_biloba

| ,-- Cordaixylon_dumusum

|--| | ,-- Emporia_lockardii

| | ,----- Hanskerpia_hamiltonensis

`--| | ,-- Voltzia_hexagona

| | | ,-- Nothodacrium_warrenii

`--| | ,----- Rissikia_media

| | | ,-- Pararaucaria_patagonica

`--| |	,--| ,-- **New_total_plant_from_Arkansas**

| | | `----- Alvinia_bohemica_plant

| | ,--| ,-- Himeriella_muensteri

`--| | `--| ,-- Kachaikestrobus_acuminatus

| | `----- Tomaxellia_biforme

| | ,-- Telemachus_aequatus

| | | ,-- Eathiestrobus_mackenziei

| | | | ,-- Cedrus_deodara

`--| |	| |	,-- Keteleeria_davidiana

| | ,--| | ,--| ,-- Mataia_podocarpoides

| | | | | | `----- Abies_lasiocarpa

| | | `--| ,--| ,-- Pseudolarix_amabilis

| | | | | `--| ,-- Tsuga_canadensis

`--| |	| |	`----- Nothotsuga_longibracteata

| | `--| ,-- Pseudotsuga_menziesii

| | | ,----- Larix_decidua

| | `--| ,-- Cathaya_argyrophylla

| | `--| ,-- Pinus_contorta

| | `--| ,-- Picea_abies

| | `--| ,-- Schizolepidopsis_ediae

`--|	`--| ,-- Schizolepidopsis_daohugouensis

| `----- Schizolepidopsis_canicularis

| ,-- Sciadopitys_verticillata

| ,--| ,-- Taxus_wallichiana

| | `--| ,-- Cunninghamia_lanceolata

| | `--| ,-- Taiwania_cryptomerioides

| | `--| ,-- Athrotaxis_selaginoides

| | `--| ,-- Sequoia_sempervirens

| | `--| ,-- Cryptomeria_japonica

| | `--| ,-- Cupressus_funebris

`--|	`----- Callitris_muelleri

| ,-- Araucaria_mirabilis

| ,----- Araucaria_heterophylla

| ,--| ,-- Wollemia_nobilis

| | `----- Agathis_australis

| | ,-- Lagarostrobos_franklinii

`--|	,----- Prumnopitys_taxifolia

| ,--| ,-- Lepidothamnus_laxifolius

| | `----- Phyllocladus_aspleniifolius

`--| ,-- Saxegothaea_conspicua

`--| ,-- Microcachrys_tetragona

`--| ,-- Pherosphaera_hookeriana

`--| ,-- Acmopyle_pancheri

`--| ,-- Dacrydium_cupressinum

`--| ,-- Retrophyllum_rospigliosii

`----- Podocarpus_archboldii

Tree 77:

,-- Ginkgo_biloba

| ,-- Cordaixylon_dumusum

|--| | ,-- Emporia_lockardii

| | ,----- Hanskerpia_hamiltonensis

`--| | ,-- Voltzia_hexagona

| | | ,-- Nothodacrium_warrenii

`--| | ,----- Rissikia_media

| | | ,-- Pararaucaria_patagonica

`--| |	,--| ,-- **New_total_plant_from_Arkansas**

| | | `----- Alvinia_bohemica_plant

| | ,--| ,-- Himeriella_muensteri

`--| | `--| ,-- Kachaikestrobus_acuminatus

| | `----- Tomaxellia_biforme

| | ,-- Telemachus_aequatus

| | | ,-- Eathiestrobus_mackenziei

| | | | ,-- Cedrus_deodara

`--| |	| |	,-- Keteleeria_davidiana

| | ,--| | ,----- Abies_lasiocarpa

| | | | | | ,-- Mataia_podocarpoides

| | | `--| ,--| ,----- Pseudolarix_amabilis

| | | | | `--| ,-- Tsuga_canadensis

`--| |	| |	`----- Nothotsuga_longibracteata

| | `--| ,-- Pseudotsuga_menziesii

| | | ,----- Larix_decidua

| | `--| ,-- Cathaya_argyrophylla

| | `--| ,-- Pinus_contorta

| | `--| ,-- Picea_abies

| | `--| ,-- Schizolepidopsis_ediae

`--|	`--| ,-- Schizolepidopsis_daohugouensis

| `----- Schizolepidopsis_canicularis

| ,-- Sciadopitys_verticillata

| ,--| ,-- Taxus_wallichiana

| | `--| ,-- Cunninghamia_lanceolata

| | `--| ,-- Taiwania_cryptomerioides

| | `--| ,-- Athrotaxis_selaginoides

| | `--| ,-- Sequoia_sempervirens

| | `--| ,-- Cryptomeria_japonica

| | `--| ,-- Cupressus_funebris

`--|	`----- Callitris_muelleri

| ,-- Araucaria_mirabilis

| ,----- Araucaria_heterophylla

| ,--| ,-- Wollemia_nobilis

| | `----- Agathis_australis

| | ,-- Lagarostrobos_franklinii

`--|	,----- Prumnopitys_taxifolia

| ,--| ,-- Lepidothamnus_laxifolius

| | `----- Phyllocladus_aspleniifolius

`--| ,-- Saxegothaea_conspicua

`--| ,-- Microcachrys_tetragona

`--| ,-- Pherosphaera_hookeriana

`--| ,-- Acmopyle_pancheri

`--| ,-- Dacrydium_cupressinum

`--| ,-- Retrophyllum_rospigliosii

`----- Podocarpus_archboldii

Tree 78:

,-- Ginkgo_biloba

| ,-- Cordaixylon_dumusum

|--| | ,-- Emporia_lockardii

| | ,----- Hanskerpia_hamiltonensis

`--| | ,-- Voltzia_hexagona

| | | ,-- Nothodacrium_warrenii

`--| | ,----- Rissikia_media

| | | ,-- Pararaucaria_patagonica

`--| |	,--| ,-- **New_total_plant_from_Arkansas**

| | | `----- Alvinia_bohemica_plant

| | ,--| ,-- Himeriella_muensteri

`--| | `--| ,-- Kachaikestrobus_acuminatus

| | `----- Tomaxellia_biforme

| | ,-- Telemachus_aequatus

| | | ,-- Eathiestrobus_mackenziei

`--| |	| ,-- Cedrus_deodara

| | ,--| | ,-- Keteleeria_davidiana

| | | | | ,----- Abies_lasiocarpa

| | | `--| ,--| ,-- Pseudolarix_amabilis

| | | | | `--| ,-- Mataia_podocarpoides

`--| |	| |	`--| ,-- Tsuga_canadensis

| | `--| `----- Nothotsuga_longibracteata

| | | ,-- Pseudotsuga_menziesii

| | | ,----- Larix_decidua

| | `--| ,-- Cathaya_argyrophylla

| | `--| ,-- Pinus_contorta

| | `--| ,-- Picea_abies

`--|	`--| ,-- Schizolepidopsis_ediae

| `--| ,-- Schizolepidopsis_daohugouensis

| `----- Schizolepidopsis_canicularis

| ,-- Sciadopitys_verticillata

| ,--| ,-- Taxus_wallichiana

| | `--| ,-- Cunninghamia_lanceolata

| | `--| ,-- Taiwania_cryptomerioides

| | `--| ,-- Athrotaxis_selaginoides

| | `--| ,-- Sequoia_sempervirens

| | `--| ,-- Cryptomeria_japonica

| | `--| ,-- Cupressus_funebris

`--|	`----- Callitris_muelleri

| ,-- Araucaria_mirabilis

| ,----- Araucaria_heterophylla

| ,--| ,-- Wollemia_nobilis

| | `----- Agathis_australis

| | ,-- Lagarostrobos_franklinii

`--|	,----- Prumnopitys_taxifolia

| ,--| ,-- Lepidothamnus_laxifolius

| | `----- Phyllocladus_aspleniifolius

`--| ,-- Saxegothaea_conspicua

`--| ,-- Microcachrys_tetragona

`--| ,-- Pherosphaera_hookeriana

`--| ,-- Acmopyle_pancheri

`--| ,-- Dacrydium_cupressinum

`--| ,-- Retrophyllum_rospigliosii

`----- Podocarpus_archboldii

Tree 79:

,-- Ginkgo_biloba

| ,-- Cordaixylon_dumusum

|--| | ,-- Emporia_lockardii

| | ,----- Hanskerpia_hamiltonensis

`--| | ,-- Voltzia_hexagona

| | | ,-- Nothodacrium_warrenii

`--| | ,----- Rissikia_media

| | | ,-- Pararaucaria_patagonica

`--| |	,--| ,-- **New_total_plant_from_Arkansas**

| | | `----- Alvinia_bohemica_plant

| | ,--| ,-- Himeriella_muensteri

`--| | `--| ,-- Kachaikestrobus_acuminatus

| | `----- Tomaxellia_biforme

| | ,-- Telemachus_aequatus

| | | ,-- Eathiestrobus_mackenziei

`--| |	| ,-- Cedrus_deodara

| | ,--| | ,-- Keteleeria_davidiana

| | | | | ,----- Abies_lasiocarpa

| | | `--| ,--| ,-- Pseudolarix_amabilis

| | | | | `--| ,-- Tsuga_canadensis

`--| |	| |	`--| ,-- Mataia_podocarpoides

| | `--| `----- Nothotsuga_longibracteata

| | | ,-- Pseudotsuga_menziesii

| | | ,----- Larix_decidua

| | `--| ,-- Cathaya_argyrophylla

| | `--| ,-- Pinus_contorta

| | `--| ,-- Picea_abies

`--|	`--| ,-- Schizolepidopsis_ediae

| `--| ,-- Schizolepidopsis_daohugouensis

| `----- Schizolepidopsis_canicularis

| ,-- Sciadopitys_verticillata

| ,--| ,-- Taxus_wallichiana

| | `--| ,-- Cunninghamia_lanceolata

| | `--| ,-- Taiwania_cryptomerioides

| | `--| ,-- Athrotaxis_selaginoides

| | `--| ,-- Sequoia_sempervirens

| | `--| ,-- Cryptomeria_japonica

| | `--| ,-- Cupressus_funebris

`--|	`----- Callitris_muelleri

| ,-- Araucaria_mirabilis

| ,----- Araucaria_heterophylla

| ,--| ,-- Wollemia_nobilis

| | `----- Agathis_australis

| | ,-- Lagarostrobos_franklinii

`--|	,----- Prumnopitys_taxifolia

| ,--| ,-- Lepidothamnus_laxifolius

| | `----- Phyllocladus_aspleniifolius

`--| ,-- Saxegothaea_conspicua

`--| ,-- Microcachrys_tetragona

`--| ,-- Pherosphaera_hookeriana

`--| ,-- Acmopyle_pancheri

`--| ,-- Dacrydium_cupressinum

`--| ,-- Retrophyllum_rospigliosii

`----- Podocarpus_archboldii

Tree 80:

,-- Ginkgo_biloba

| ,-- Cordaixylon_dumusum

|--| | ,-- Emporia_lockardii

`--| | ,-- Hanskerpia_hamiltonensis

`--| | ,-- Voltzia_hexagona

`--| |	,-- Nothodacrium_warrenii

| | ,----- Rissikia_media

`--| | ,-- Telemachus_aequatus

| | | ,-- Schizolepidopsis_ediae

| | | | ,-- Schizolepidopsis_daohugouensis

`--| | ,--| ,----- Schizolepidopsis_canicularis

| | | | | ,-- Cedrus_deodara

| | | `--| | ,-- Mataia_podocarpoides

| | | | | | ,-- Keteleeria_davidiana

`--| |	`--| ,--| ,----- Abies_lasiocarpa

| | | | `--| ,-- Pseudolarix_amabilis

| | | | `--| ,-- Tsuga_canadensis

| | `--| `----- Nothotsuga_longibracteata

| | | ,-- Pseudotsuga_menziesii

| | | ,----- Larix_decidua

`--|	`--| ,-- Cathaya_argyrophylla

| `--| ,-- Picea_abies

| `--| ,-- Eathiestrobus_mackenziei

| `----- Pinus_contorta

| ,-- **New_total_plant_from_Arkansas**

| ,----- Alvinia_bohemica_plant

| ,--| ,-- Himeriella_muensteri

| | `--| ,-- Kachaikestrobus_acuminatus

| | `----- Tomaxellia_biforme

| | ,-- Pararaucaria_patagonica

`--| |	,-- Sciadopitys_verticillata

| | ,--| ,-- Taxus_wallichiana

| | | `--| ,-- Cunninghamia_lanceolata

| | | `--| ,-- Taiwania_cryptomerioides

`--| |	`--| ,-- Athrotaxis_selaginoides

| | `--| ,-- Sequoia_sempervirens

| | `--| ,-- Cryptomeria_japonica

| | `--| ,-- Cupressus_funebris

`--|	`----- Callitris_muelleri

| ,-- Araucaria_mirabilis

| ,----- Araucaria_heterophylla

| ,--| ,-- Wollemia_nobilis

| | `----- Agathis_australis

| | ,-- Lagarostrobos_franklinii

`--|	,----- Prumnopitys_taxifolia

| ,--| ,-- Lepidothamnus_laxifolius

| | `----- Phyllocladus_aspleniifolius

`--| ,-- Saxegothaea_conspicua

`--| ,-- Microcachrys_tetragona

`--| ,-- Pherosphaera_hookeriana

`--| ,-- Acmopyle_pancheri

`--| ,-- Dacrydium_cupressinum

`--| ,-- Retrophyllum_rospigliosii

`----- Podocarpus_archboldii

Tree 81:

,-- Ginkgo_biloba

| ,-- Cordaixylon_dumusum

|--| | ,-- Emporia_lockardii

`--| | ,-- Hanskerpia_hamiltonensis

`--| | ,-- Voltzia_hexagona

`--| |	,-- Nothodacrium_warrenii

| | ,----- Rissikia_media

`--| | ,-- Telemachus_aequatus

| | | ,-- Schizolepidopsis_ediae

| | | | ,-- Schizolepidopsis_daohugouensis

`--| |	| ,----- Schizolepidopsis_canicularis

| | ,--| | ,-- Cedrus_deodara

| | | | | | ,-- Keteleeria_davidiana

| | | `--| | ,--| ,-- Mataia_podocarpoides

`--| |	| |	| `----- Abies_lasiocarpa

| | `--| ,--| ,-- Pseudolarix_amabilis

| | | | `--| ,-- Tsuga_canadensis

| | | | `----- Nothotsuga_longibracteata

| | `--| ,-- Pseudotsuga_menziesii

| | | ,----- Larix_decidua

| | `--| ,-- Cathaya_argyrophylla

`--|	`--| ,-- Pinus_contorta

| `--| ,-- Eathiestrobus_mackenziei

| `----- Picea_abies

| ,-- **New_total_plant_from_Arkansas**

| ,----- Alvinia_bohemica_plant

| ,--| ,-- Himeriella_muensteri

| | `--| ,-- Kachaikestrobus_acuminatus

| | `----- Tomaxellia_biforme

| | ,-- Pararaucaria_patagonica

`--| |	,-- Sciadopitys_verticillata

| | ,--| ,-- Taxus_wallichiana

| | | `--| ,-- Cunninghamia_lanceolata

| | | `--| ,-- Taiwania_cryptomerioides

`--| |	`--| ,-- Athrotaxis_selaginoides

| | `--| ,-- Sequoia_sempervirens

| | `--| ,-- Cryptomeria_japonica

| | `--| ,-- Cupressus_funebris

`--|	`----- Callitris_muelleri

| ,-- Araucaria_mirabilis

| ,----- Araucaria_heterophylla

| ,--| ,-- Wollemia_nobilis

| | `----- Agathis_australis

| | ,-- Lagarostrobos_franklinii

`--|	,----- Prumnopitys_taxifolia

| ,--| ,-- Lepidothamnus_laxifolius

| | `----- Phyllocladus_aspleniifolius

`--| ,-- Saxegothaea_conspicua

`--| ,-- Microcachrys_tetragona

`--| ,-- Pherosphaera_hookeriana

`--| ,-- Acmopyle_pancheri

`--| ,-- Dacrydium_cupressinum

`--| ,-- Retrophyllum_rospigliosii

`----- Podocarpus_archboldii

Tree 82:

,-- Ginkgo_biloba

| ,-- Cordaixylon_dumusum

|--| | ,-- Emporia_lockardii

`--| | ,-- Hanskerpia_hamiltonensis

`--| | ,-- Voltzia_hexagona

`--| |	,-- Nothodacrium_warrenii

| | ,----- Rissikia_media

`--| | ,-- Telemachus_aequatus

| | | ,-- Schizolepidopsis_ediae

| | | | ,-- Schizolepidopsis_daohugouensis

`--| |	| ,----- Schizolepidopsis_canicularis

| | ,--| | ,-- Cedrus_deodara

| | | | | | ,-- Keteleeria_davidiana

| | | `--| | ,----- Abies_lasiocarpa

`--| |	| |	|	,-- Mataia_podocarpoides

| | `--| ,--| ,----- Pseudolarix_amabilis

| | | | `--| ,-- Tsuga_canadensis

| | | | `----- Nothotsuga_longibracteata

| | `--| ,-- Pseudotsuga_menziesii

| | | ,----- Larix_decidua

| | `--| ,-- Cathaya_argyrophylla

`--|	`--| ,-- Pinus_contorta

| `--| ,-- Eathiestrobus_mackenziei

| `----- Picea_abies

| ,-- **New_total_plant_from_Arkansas**

| ,----- Alvinia_bohemica_plant

| ,--| ,-- Himeriella_muensteri

| | `--| ,-- Kachaikestrobus_acuminatus

| | `----- Tomaxellia_biforme

| | ,-- Pararaucaria_patagonica

`--| |	,-- Sciadopitys_verticillata

| | ,--| ,-- Taxus_wallichiana

| | | `--| ,-- Cunninghamia_lanceolata

| | | `--| ,-- Taiwania_cryptomerioides

`--| |	`--| ,-- Athrotaxis_selaginoides

| | `--| ,-- Sequoia_sempervirens

| | `--| ,-- Cryptomeria_japonica

| | `--| ,-- Cupressus_funebris

`--|	`----- Callitris_muelleri

| ,-- Araucaria_mirabilis

| ,----- Araucaria_heterophylla

| ,--| ,-- Wollemia_nobilis

| | `----- Agathis_australis

| | ,-- Lagarostrobos_franklinii

`--|	,----- Prumnopitys_taxifolia

| ,--| ,-- Lepidothamnus_laxifolius

| | `----- Phyllocladus_aspleniifolius

`--| ,-- Saxegothaea_conspicua

`--| ,-- Microcachrys_tetragona

`--| ,-- Pherosphaera_hookeriana

`--| ,-- Acmopyle_pancheri

`--| ,-- Dacrydium_cupressinum

`--| ,-- Retrophyllum_rospigliosii

`----- Podocarpus_archboldii

Tree 83:

,-- Ginkgo_biloba

| ,-- Cordaixylon_dumusum

|--| | ,-- Emporia_lockardii

`--| | ,-- Hanskerpia_hamiltonensis

`--| | ,-- Voltzia_hexagona

`--| |	,-- Nothodacrium_warrenii

| | ,----- Rissikia_media

`--| | ,-- Telemachus_aequatus

| | | ,-- Schizolepidopsis_ediae

| | | | ,-- Schizolepidopsis_daohugouensis

`--| | ,--| ,----- Schizolepidopsis_canicularis

| | | | | ,-- Cedrus_deodara

| | | `--| | ,-- Keteleeria_davidiana

| | | | | ,----- Abies_lasiocarpa

`--| |	`--| ,--| ,-- Pseudolarix_amabilis

| | | | `--| ,-- Mataia_podocarpoides

| | | | `--| ,-- Tsuga_canadensis

| | `--| `----- Nothotsuga_longibracteata

| | | ,-- Pseudotsuga_menziesii

| | | ,----- Larix_decidua

`--|	`--| ,-- Cathaya_argyrophylla

| `--| ,-- Pinus_contorta

| `--| ,-- Eathiestrobus_mackenziei

| `----- Picea_abies

| ,-- **New_total_plant_from_Arkansas**

| ,----- Alvinia_bohemica_plant

| ,--| ,-- Himeriella_muensteri

| | `--| ,-- Kachaikestrobus_acuminatus

| | `----- Tomaxellia_biforme

| | ,-- Pararaucaria_patagonica

`--| |	,-- Sciadopitys_verticillata

| | ,--| ,-- Taxus_wallichiana

| | | `--| ,-- Cunninghamia_lanceolata

| | | `--| ,-- Taiwania_cryptomerioides

`--| |	`--| ,-- Athrotaxis_selaginoides

| | `--| ,-- Sequoia_sempervirens

| | `--| ,-- Cryptomeria_japonica

| | `--| ,-- Cupressus_funebris

`--|	`----- Callitris_muelleri

| ,-- Araucaria_mirabilis

| ,----- Araucaria_heterophylla

| ,--| ,-- Wollemia_nobilis

| | `----- Agathis_australis

| | ,-- Lagarostrobos_franklinii

`--|	,----- Prumnopitys_taxifolia

| ,--| ,-- Lepidothamnus_laxifolius

| | `----- Phyllocladus_aspleniifolius

`--| ,-- Saxegothaea_conspicua

`--| ,-- Microcachrys_tetragona

`--| ,-- Pherosphaera_hookeriana

`--| ,-- Acmopyle_pancheri

`--| ,-- Dacrydium_cupressinum

`--| ,-- Retrophyllum_rospigliosii

`----- Podocarpus_archboldii

Tree 84:

,-- Ginkgo_biloba

| ,-- Cordaixylon_dumusum

|--| | ,-- Emporia_lockardii

`--| | ,-- Hanskerpia_hamiltonensis

`--| | ,-- Voltzia_hexagona

`--| |	,-- Nothodacrium_warrenii

| | ,----- Rissikia_media

`--| | ,-- Telemachus_aequatus

| | | ,-- Schizolepidopsis_ediae

| | | | ,-- Schizolepidopsis_daohugouensis

`--| | ,--| ,----- Schizolepidopsis_canicularis

| | | | | ,-- Cedrus_deodara

| | | `--| | ,-- Keteleeria_davidiana

| | | | | ,----- Abies_lasiocarpa

`--| |	`--| ,--| ,-- Pseudolarix_amabilis

| | | | `--| ,-- Tsuga_canadensis

| | | | `--| ,-- Mataia_podocarpoides

| | `--| `----- Nothotsuga_longibracteata

| | | ,-- Pseudotsuga_menziesii

| | | ,----- Larix_decidua

`--|	`--| ,-- Cathaya_argyrophylla

| `--| ,-- Pinus_contorta

| `--| ,-- Eathiestrobus_mackenziei

| `----- Picea_abies

| ,-- **New_total_plant_from_Arkansas**

| ,----- Alvinia_bohemica_plant

| ,--| ,-- Himeriella_muensteri

| | `--| ,-- Kachaikestrobus_acuminatus

| | `----- Tomaxellia_biforme

| | ,-- Pararaucaria_patagonica

`--| |	,-- Sciadopitys_verticillata

| | ,--| ,-- Taxus_wallichiana

| | | `--| ,-- Cunninghamia_lanceolata

| | | `--| ,-- Taiwania_cryptomerioides

`--| |	`--| ,-- Athrotaxis_selaginoides

| | `--| ,-- Sequoia_sempervirens

| | `--| ,-- Cryptomeria_japonica

| | `--| ,-- Cupressus_funebris

`--|	`----- Callitris_muelleri

| ,-- Araucaria_mirabilis

| ,----- Araucaria_heterophylla

| ,--| ,-- Wollemia_nobilis

| | `----- Agathis_australis

| | ,-- Lagarostrobos_franklinii

`--|	,----- Prumnopitys_taxifolia

| ,--| ,-- Lepidothamnus_laxifolius

| | `----- Phyllocladus_aspleniifolius

`--| ,-- Saxegothaea_conspicua

`--| ,-- Microcachrys_tetragona

`--| ,-- Pherosphaera_hookeriana

`--| ,-- Acmopyle_pancheri

`--| ,-- Dacrydium_cupressinum

`--| ,-- Retrophyllum_rospigliosii

`----- Podocarpus_archboldii

Tree 85:

,-- Ginkgo_biloba

| ,-- Cordaixylon_dumusum

|--| | ,-- Emporia_lockardii

`--| | ,-- Hanskerpia_hamiltonensis

`--| | ,-- Voltzia_hexagona

`--| |	,-- Nothodacrium_warrenii

| | ,----- Rissikia_media

`--| | ,-- Telemachus_aequatus

| | | ,-- Schizolepidopsis_ediae

| | | | ,-- Schizolepidopsis_daohugouensis

`--| | ,--| ,----- Schizolepidopsis_canicularis

| | | | | ,-- Cedrus_deodara

| | | `--| | ,-- Keteleeria_davidiana

| | | | | ,----- Abies_lasiocarpa

`--| |	`--| ,--| ,-- Mataia_podocarpoides

| | | | `--| ,-- Pseudolarix_amabilis

| | | | `--| ,-- Tsuga_canadensis

| | `--| `----- Nothotsuga_longibracteata

| | | ,-- Pseudotsuga_menziesii

| | | ,----- Larix_decidua

`--|	`--| ,-- Cathaya_argyrophylla

| `--| ,-- Pinus_contorta

| `--| ,-- Eathiestrobus_mackenziei

| `----- Picea_abies

| ,-- **New_total_plant_from_Arkansas**

| ,----- Alvinia_bohemica_plant

| ,--| ,-- Himeriella_muensteri

| | `--| ,-- Kachaikestrobus_acuminatus

| | `----- Tomaxellia_biforme

| | ,-- Pararaucaria_patagonica

`--| |	,-- Sciadopitys_verticillata

| | ,--| ,-- Taxus_wallichiana

| | | `--| ,-- Cunninghamia_lanceolata

| | | `--| ,-- Taiwania_cryptomerioides

`--| |	`--| ,-- Athrotaxis_selaginoides

| | `--| ,-- Sequoia_sempervirens

| | `--| ,-- Cryptomeria_japonica

| | `--| ,-- Cupressus_funebris

`--|	`----- Callitris_muelleri

| ,-- Araucaria_mirabilis

| ,----- Araucaria_heterophylla

| ,--| ,-- Wollemia_nobilis

| | `----- Agathis_australis

| | ,-- Lagarostrobos_franklinii

`--|	,----- Prumnopitys_taxifolia

| ,--| ,-- Lepidothamnus_laxifolius

| | `----- Phyllocladus_aspleniifolius

`--| ,-- Saxegothaea_conspicua

`--| ,-- Microcachrys_tetragona

`--| ,-- Pherosphaera_hookeriana

`--| ,-- Acmopyle_pancheri

`--| ,-- Dacrydium_cupressinum

`--| ,-- Retrophyllum_rospigliosii

`----- Podocarpus_archboldii

Tree 86:

,-- Ginkgo_biloba

| ,-- Cordaixylon_dumusum

|--| | ,-- Emporia_lockardii

`--| | ,-- Hanskerpia_hamiltonensis

`--| | ,-- Voltzia_hexagona

`--| |	,-- Nothodacrium_warrenii

| | ,----- Rissikia_media

`--| | ,-- Telemachus_aequatus

| | | ,-- Schizolepidopsis_ediae

| | | | ,-- Schizolepidopsis_daohugouensis

`--| |	| ,----- Schizolepidopsis_canicularis

| | ,--| | ,-- Cedrus_deodara

| | | | | | ,-- Mataia_podocarpoides

| | | `--| | ,--| ,-- Keteleeria_davidiana

`--| |	| |	| `----- Abies_lasiocarpa

| | `--| ,--| ,-- Pseudolarix_amabilis

| | | | `--| ,-- Tsuga_canadensis

| | | | `----- Nothotsuga_longibracteata

| | `--| ,-- Pseudotsuga_menziesii

| | | ,----- Larix_decidua

| | `--| ,-- Cathaya_argyrophylla

`--|	`--| ,-- Pinus_contorta

| `--| ,-- Eathiestrobus_mackenziei

| `----- Picea_abies

| ,-- **New_total_plant_from_Arkansas**

| ,----- Alvinia_bohemica_plant

| ,--| ,-- Himeriella_muensteri

| | `--| ,-- Kachaikestrobus_acuminatus

| | `----- Tomaxellia_biforme

| | ,-- Pararaucaria_patagonica

`--| |	,-- Sciadopitys_verticillata

| | ,--| ,-- Taxus_wallichiana

| | | `--| ,-- Cunninghamia_lanceolata

| | | `--| ,-- Taiwania_cryptomerioides

`--| |	`--| ,-- Athrotaxis_selaginoides

| | `--| ,-- Sequoia_sempervirens

| | `--| ,-- Cryptomeria_japonica

| | `--| ,-- Cupressus_funebris

`--|	`----- Callitris_muelleri

| ,-- Araucaria_mirabilis

| ,----- Araucaria_heterophylla

| ,--| ,-- Wollemia_nobilis

| | `----- Agathis_australis

| | ,-- Lagarostrobos_franklinii

`--|	,----- Prumnopitys_taxifolia

| ,--| ,-- Lepidothamnus_laxifolius

| | `----- Phyllocladus_aspleniifolius

`--| ,-- Saxegothaea_conspicua

`--| ,-- Microcachrys_tetragona

`--| ,-- Pherosphaera_hookeriana

`--| ,-- Acmopyle_pancheri

`--| ,-- Dacrydium_cupressinum

`--| ,-- Retrophyllum_rospigliosii

`----- Podocarpus_archboldii

Tree 87:

,-- Ginkgo_biloba

| ,-- Cordaixylon_dumusum

|--| | ,-- Emporia_lockardii

`--| ,----- Hanskerpia_hamiltonensis

| | ,-- Voltzia_hexagona

`--| |	,-- Nothodacrium_warrenii

| | ,----- Rissikia_media

`--| | ,-- Telemachus_aequatus

| | | ,-- Schizolepidopsis_ediae

| | | | ,-- Schizolepidopsis_daohugouensis

`--| | ,--| ,----- Schizolepidopsis_canicularis

| | | | | ,-- Cedrus_deodara

| | | `--| | ,-- Mataia_podocarpoides

| | | | | | ,-- Keteleeria_davidiana

`--| |	`--| ,--| ,----- Abies_lasiocarpa

| | | | `--| ,-- Pseudolarix_amabilis

| | | | `--| ,-- Tsuga_canadensis

| | `--| `----- Nothotsuga_longibracteata

| | | ,-- Pseudotsuga_menziesii

| | | ,----- Larix_decidua

`--|	`--| ,-- Cathaya_argyrophylla

| `--| ,-- Picea_abies

| `--| ,-- Eathiestrobus_mackenziei

| `----- Pinus_contorta

| ,-- **New_total_plant_from_Arkansas**

| ,----- Alvinia_bohemica_plant

| ,--| ,-- Himeriella_muensteri

| | `--| ,-- Kachaikestrobus_acuminatus

| | `----- Tomaxellia_biforme

| | ,-- Pararaucaria_patagonica

`--| |	,-- Sciadopitys_verticillata

| | ,--| ,-- Taxus_wallichiana

| | | `--| ,-- Cunninghamia_lanceolata

| | | `--| ,-- Taiwania_cryptomerioides

`--| |	`--| ,-- Athrotaxis_selaginoides

| | `--| ,-- Sequoia_sempervirens

| | `--| ,-- Cryptomeria_japonica

| | `--| ,-- Cupressus_funebris

`--|	`----- Callitris_muelleri

| ,-- Araucaria_mirabilis

| ,----- Araucaria_heterophylla

| ,--| ,-- Wollemia_nobilis

| | `----- Agathis_australis

| | ,-- Lagarostrobos_franklinii

`--|	,----- Prumnopitys_taxifolia

| ,--| ,-- Lepidothamnus_laxifolius

| | `----- Phyllocladus_aspleniifolius

`--| ,-- Saxegothaea_conspicua

`--| ,-- Microcachrys_tetragona

`--| ,-- Pherosphaera_hookeriana

`--| ,-- Acmopyle_pancheri

`--| ,-- Dacrydium_cupressinum

`--| ,-- Retrophyllum_rospigliosii

`----- Podocarpus_archboldii

Tree 88:

,-- Ginkgo_biloba

| ,-- Cordaixylon_dumusum

|--| | ,-- Emporia_lockardii

`--| ,----- Hanskerpia_hamiltonensis

| | ,-- Voltzia_hexagona

`--| |	,-- Nothodacrium_warrenii

| | ,----- Rissikia_media

`--| | ,-- Telemachus_aequatus

| | | ,-- Schizolepidopsis_ediae

| | | | ,-- Schizolepidopsis_daohugouensis

`--| |	| ,----- Schizolepidopsis_canicularis

| | ,--| | ,-- Cedrus_deodara

| | | | | | ,-- Keteleeria_davidiana

| | | `--| | ,--| ,-- Mataia_podocarpoides

`--| |	| |	| `----- Abies_lasiocarpa

| | `--| ,--| ,-- Pseudolarix_amabilis

| | | | `--| ,-- Tsuga_canadensis

| | | | `----- Nothotsuga_longibracteata

| | `--| ,-- Pseudotsuga_menziesii

| | | ,----- Larix_decidua

| | `--| ,-- Cathaya_argyrophylla

`--|	`--| ,-- Pinus_contorta

| `--| ,-- Eathiestrobus_mackenziei

| `----- Picea_abies

| ,-- **New_total_plant_from_Arkansas**

| ,----- Alvinia_bohemica_plant

| ,--| ,-- Himeriella_muensteri

| | `--| ,-- Kachaikestrobus_acuminatus

| | `----- Tomaxellia_biforme

| | ,-- Pararaucaria_patagonica

`--| |	,-- Sciadopitys_verticillata

| | ,--| ,-- Taxus_wallichiana

| | | `--| ,-- Cunninghamia_lanceolata

| | | `--| ,-- Taiwania_cryptomerioides

`--| |	`--| ,-- Athrotaxis_selaginoides

| | `--| ,-- Sequoia_sempervirens

| | `--| ,-- Cryptomeria_japonica

| | `--| ,-- Cupressus_funebris

`--|	`----- Callitris_muelleri

| ,-- Araucaria_mirabilis

| ,----- Araucaria_heterophylla

| ,--| ,-- Wollemia_nobilis

| | `----- Agathis_australis

| | ,-- Lagarostrobos_franklinii

`--|	,----- Prumnopitys_taxifolia

| ,--| ,-- Lepidothamnus_laxifolius

| | `----- Phyllocladus_aspleniifolius

`--| ,-- Saxegothaea_conspicua

`--| ,-- Microcachrys_tetragona

`--| ,-- Pherosphaera_hookeriana

`--| ,-- Acmopyle_pancheri

`--| ,-- Dacrydium_cupressinum

`--| ,-- Retrophyllum_rospigliosii

`----- Podocarpus_archboldii

Tree 89:

,-- Ginkgo_biloba

| ,-- Cordaixylon_dumusum

|--| | ,-- Emporia_lockardii

`--| ,----- Hanskerpia_hamiltonensis

| | ,-- Voltzia_hexagona

`--| |	,-- Nothodacrium_warrenii

| | ,----- Rissikia_media

`--| | ,-- Telemachus_aequatus

| | | ,-- Schizolepidopsis_ediae

| | | | ,-- Schizolepidopsis_daohugouensis

`--| |	| ,----- Schizolepidopsis_canicularis

| | ,--| | ,-- Cedrus_deodara

| | | | | | ,-- Keteleeria_davidiana

| | | `--| | ,----- Abies_lasiocarpa

`--| |	| |	|	,-- Mataia_podocarpoides

| | `--| ,--| ,----- Pseudolarix_amabilis

| | | | `--| ,-- Tsuga_canadensis

| | | | `----- Nothotsuga_longibracteata

| | `--| ,-- Pseudotsuga_menziesii

| | | ,----- Larix_decidua

| | `--| ,-- Cathaya_argyrophylla

`--|	`--| ,-- Pinus_contorta

| `--| ,-- Eathiestrobus_mackenziei

| `----- Picea_abies

| ,-- **New_total_plant_from_Arkansas**

| ,----- Alvinia_bohemica_plant

| ,--| ,-- Himeriella_muensteri

| | `--| ,-- Kachaikestrobus_acuminatus

| | `----- Tomaxellia_biforme

| | ,-- Pararaucaria_patagonica

`--| |	,-- Sciadopitys_verticillata

| | ,--| ,-- Taxus_wallichiana

| | | `--| ,-- Cunninghamia_lanceolata

| | | `--| ,-- Taiwania_cryptomerioides

`--| |	`--| ,-- Athrotaxis_selaginoides

| | `--| ,-- Sequoia_sempervirens

| | `--| ,-- Cryptomeria_japonica

| | `--| ,-- Cupressus_funebris

`--|	`----- Callitris_muelleri

| ,-- Araucaria_mirabilis

| ,----- Araucaria_heterophylla

| ,--| ,-- Wollemia_nobilis

| | `----- Agathis_australis

| | ,-- Lagarostrobos_franklinii

`--|	,----- Prumnopitys_taxifolia

| ,--| ,-- Lepidothamnus_laxifolius

| | `----- Phyllocladus_aspleniifolius

`--| ,-- Saxegothaea_conspicua

`--| ,-- Microcachrys_tetragona

`--| ,-- Pherosphaera_hookeriana

`--| ,-- Acmopyle_pancheri

`--| ,-- Dacrydium_cupressinum

`--| ,-- Retrophyllum_rospigliosii

`----- Podocarpus_archboldii

Tree 90:

,-- Ginkgo_biloba

| ,-- Cordaixylon_dumusum

|--| | ,-- Emporia_lockardii

`--| ,----- Hanskerpia_hamiltonensis

| | ,-- Voltzia_hexagona

`--| |	,-- Nothodacrium_warrenii

| | ,----- Rissikia_media

`--| | ,-- Telemachus_aequatus

| | | ,-- Schizolepidopsis_ediae

| | | | ,-- Schizolepidopsis_daohugouensis

`--| | ,--| ,----- Schizolepidopsis_canicularis

| | | | | ,-- Cedrus_deodara

| | | `--| | ,-- Keteleeria_davidiana

| | | | | ,----- Abies_lasiocarpa

`--| |	`--| ,--| ,-- Pseudolarix_amabilis

| | | | `--| ,-- Mataia_podocarpoides

| | | | `--| ,-- Tsuga_canadensis

| | `--| `----- Nothotsuga_longibracteata

| | | ,-- Pseudotsuga_menziesii

| | | ,----- Larix_decidua

`--|	`--| ,-- Cathaya_argyrophylla

| `--| ,-- Pinus_contorta

| `--| ,-- Eathiestrobus_mackenziei

| `----- Picea_abies

| ,-- **New_total_plant_from_Arkansas**

| ,----- Alvinia_bohemica_plant

| ,--| ,-- Himeriella_muensteri

| | `--| ,-- Kachaikestrobus_acuminatus

| | `----- Tomaxellia_biforme

| | ,-- Pararaucaria_patagonica

`--| |	,-- Sciadopitys_verticillata

| | ,--| ,-- Taxus_wallichiana

| | | `--| ,-- Cunninghamia_lanceolata

| | | `--| ,-- Taiwania_cryptomerioides

`--| |	`--| ,-- Athrotaxis_selaginoides

| | `--| ,-- Sequoia_sempervirens

| | `--| ,-- Cryptomeria_japonica

| | `--| ,-- Cupressus_funebris

`--|	`----- Callitris_muelleri

| ,-- Araucaria_mirabilis

| ,----- Araucaria_heterophylla

| ,--| ,-- Wollemia_nobilis

| | `----- Agathis_australis

| | ,-- Lagarostrobos_franklinii

`--|	,----- Prumnopitys_taxifolia

| ,--| ,-- Lepidothamnus_laxifolius

| | `----- Phyllocladus_aspleniifolius

`--| ,-- Saxegothaea_conspicua

`--| ,-- Microcachrys_tetragona

`--| ,-- Pherosphaera_hookeriana

`--| ,-- Acmopyle_pancheri

`--| ,-- Dacrydium_cupressinum

`--| ,-- Retrophyllum_rospigliosii

`----- Podocarpus_archboldii

Tree 91:

,-- Ginkgo_biloba

| ,-- Cordaixylon_dumusum

|--| | ,-- Emporia_lockardii

`--| ,----- Hanskerpia_hamiltonensis

| | ,-- Voltzia_hexagona

`--| |	,-- Nothodacrium_warrenii

| | ,----- Rissikia_media

`--| | ,-- Telemachus_aequatus

| | | ,-- Schizolepidopsis_ediae

| | | | ,-- Schizolepidopsis_daohugouensis

`--| | ,--| ,----- Schizolepidopsis_canicularis

| | | | | ,-- Cedrus_deodara

| | | `--| | ,-- Keteleeria_davidiana

| | | | | ,----- Abies_lasiocarpa

`--| |	`--| ,--| ,-- Pseudolarix_amabilis

| | | | `--| ,-- Tsuga_canadensis

| | | | `--| ,-- Mataia_podocarpoides

| | `--| `----- Nothotsuga_longibracteata

| | | ,-- Pseudotsuga_menziesii

| | | ,----- Larix_decidua

`--|	`--| ,-- Cathaya_argyrophylla

| `--| ,-- Pinus_contorta

| `--| ,-- Eathiestrobus_mackenziei

| `----- Picea_abies

| ,-- **New_total_plant_from_Arkansas**

| ,----- Alvinia_bohemica_plant

| ,--| ,-- Himeriella_muensteri

| | `--| ,-- Kachaikestrobus_acuminatus

| | `----- Tomaxellia_biforme

| | ,-- Pararaucaria_patagonica

`--| |	,-- Sciadopitys_verticillata

| | ,--| ,-- Taxus_wallichiana

| | | `--| ,-- Cunninghamia_lanceolata

| | | `--| ,-- Taiwania_cryptomerioides

`--| |	`--| ,-- Athrotaxis_selaginoides

| | `--| ,-- Sequoia_sempervirens

| | `--| ,-- Cryptomeria_japonica

| | `--| ,-- Cupressus_funebris

`--|	`----- Callitris_muelleri

| ,-- Araucaria_mirabilis

| ,----- Araucaria_heterophylla

| ,--| ,-- Wollemia_nobilis

| | `----- Agathis_australis

| | ,-- Lagarostrobos_franklinii

`--|	,----- Prumnopitys_taxifolia

| ,--| ,-- Lepidothamnus_laxifolius

| | `----- Phyllocladus_aspleniifolius

`--| ,-- Saxegothaea_conspicua

`--| ,-- Microcachrys_tetragona

`--| ,-- Pherosphaera_hookeriana

`--| ,-- Acmopyle_pancheri

`--| ,-- Dacrydium_cupressinum

`--| ,-- Retrophyllum_rospigliosii

`----- Podocarpus_archboldii

Tree 92:

,-- Ginkgo_biloba

| ,-- Cordaixylon_dumusum

|--| | ,-- Emporia_lockardii

`--| ,----- Hanskerpia_hamiltonensis

| | ,-- Voltzia_hexagona

`--| |	,-- Nothodacrium_warrenii

| | ,----- Rissikia_media

`--| | ,-- Telemachus_aequatus

| | | ,-- Schizolepidopsis_ediae

| | | | ,-- Schizolepidopsis_daohugouensis

`--| | ,--| ,----- Schizolepidopsis_canicularis

| | | | | ,-- Cedrus_deodara

| | | `--| | ,-- Keteleeria_davidiana

| | | | | ,----- Abies_lasiocarpa

`--| |	`--| ,--| ,-- Mataia_podocarpoides

| | | | `--| ,-- Pseudolarix_amabilis

| | | | `--| ,-- Tsuga_canadensis

| | `--| `----- Nothotsuga_longibracteata

| | | ,-- Pseudotsuga_menziesii

| | | ,----- Larix_decidua

`--|	`--| ,-- Cathaya_argyrophylla

| `--| ,-- Pinus_contorta

| `--| ,-- Eathiestrobus_mackenziei

| `----- Picea_abies

| ,-- **New_total_plant_from_Arkansas**

| ,----- Alvinia_bohemica_plant

| ,--| ,-- Himeriella_muensteri

| | `--| ,-- Kachaikestrobus_acuminatus

| | `----- Tomaxellia_biforme

| | ,-- Pararaucaria_patagonica

`--| |	,-- Sciadopitys_verticillata

| | ,--| ,-- Taxus_wallichiana

| | | `--| ,-- Cunninghamia_lanceolata

| | | `--| ,-- Taiwania_cryptomerioides

`--| |	`--| ,-- Athrotaxis_selaginoides

| | `--| ,-- Sequoia_sempervirens

| | `--| ,-- Cryptomeria_japonica

| | `--| ,-- Cupressus_funebris

`--|	`----- Callitris_muelleri

| ,-- Araucaria_mirabilis

| ,----- Araucaria_heterophylla

| ,--| ,-- Wollemia_nobilis

| | `----- Agathis_australis

| | ,-- Lagarostrobos_franklinii

`--|	,----- Prumnopitys_taxifolia

| ,--| ,-- Lepidothamnus_laxifolius

| | `----- Phyllocladus_aspleniifolius

`--| ,-- Saxegothaea_conspicua

`--| ,-- Microcachrys_tetragona

`--| ,-- Pherosphaera_hookeriana

`--| ,-- Acmopyle_pancheri

`--| ,-- Dacrydium_cupressinum

`--| ,-- Retrophyllum_rospigliosii

`----- Podocarpus_archboldii

Tree 93:

,-- Ginkgo_biloba

| ,-- Cordaixylon_dumusum

|--| | ,-- Emporia_lockardii

`--| ,----- Hanskerpia_hamiltonensis

| | ,-- Voltzia_hexagona

`--| |	,-- Nothodacrium_warrenii

| | ,----- Rissikia_media

`--| | ,-- Telemachus_aequatus

| | | ,-- Schizolepidopsis_ediae

| | | | ,-- Schizolepidopsis_daohugouensis

`--| |	| ,----- Schizolepidopsis_canicularis

| | ,--| | ,-- Cedrus_deodara

| | | | | | ,-- Mataia_podocarpoides

| | | `--| | ,--| ,-- Keteleeria_davidiana

`--| |	| |	| `----- Abies_lasiocarpa

| | `--| ,--| ,-- Pseudolarix_amabilis

| | | | `--| ,-- Tsuga_canadensis

| | | | `----- Nothotsuga_longibracteata

| | `--| ,-- Pseudotsuga_menziesii

| | | ,----- Larix_decidua

| | `--| ,-- Cathaya_argyrophylla

`--|	`--| ,-- Pinus_contorta

| `--| ,-- Eathiestrobus_mackenziei

| `----- Picea_abies

| ,-- **New_total_plant_from_Arkansas**

| ,----- Alvinia_bohemica_plant

| ,--| ,-- Himeriella_muensteri

| | `--| ,-- Kachaikestrobus_acuminatus

| | `----- Tomaxellia_biforme

| | ,-- Pararaucaria_patagonica

`--| |	,-- Sciadopitys_verticillata

| | ,--| ,-- Taxus_wallichiana

| | | `--| ,-- Cunninghamia_lanceolata

| | | `--| ,-- Taiwania_cryptomerioides

`--| |	`--| ,-- Athrotaxis_selaginoides

| | `--| ,-- Sequoia_sempervirens

| | `--| ,-- Cryptomeria_japonica

| | `--| ,-- Cupressus_funebris

`--|	`----- Callitris_muelleri

| ,-- Araucaria_mirabilis

| ,----- Araucaria_heterophylla

| ,--| ,-- Wollemia_nobilis

| | `----- Agathis_australis

| | ,-- Lagarostrobos_franklinii

`--|	,----- Prumnopitys_taxifolia

| ,--| ,-- Lepidothamnus_laxifolius

| | `----- Phyllocladus_aspleniifolius

`--| ,-- Saxegothaea_conspicua

`--| ,-- Microcachrys_tetragona

`--| ,-- Pherosphaera_hookeriana

`--| ,-- Acmopyle_pancheri

`--| ,-- Dacrydium_cupressinum

`--| ,-- Retrophyllum_rospigliosii

`----- Podocarpus_archboldii

Tree 94:

,-- Ginkgo_biloba

| ,-- Cordaixylon_dumusum

|--| | ,-- Emporia_lockardii

| | ,----- Hanskerpia_hamiltonensis

`--| | ,-- Voltzia_hexagona

| | | ,-- Nothodacrium_warrenii

`--| | ,----- Rissikia_media

| | | ,-- Pararaucaria_patagonica

`--| |	,--| ,-- **New_total_plant_from_Arkansas**

| | | `----- Alvinia_bohemica_plant

| | ,--| ,-- Himeriella_muensteri

`--| | `--| ,-- Kachaikestrobus_acuminatus

| | `----- Tomaxellia_biforme

| | ,-- Telemachus_aequatus

| | | ,-- Eathiestrobus_mackenziei

| | | | ,-- Cedrus_deodara

`--| |	| |	,-- Mataia_podocarpoides

| | ,--| | ,--| ,-- Keteleeria_davidiana

| | | | | | `----- Abies_lasiocarpa

| | | `--| ,--| ,-- Pseudolarix_amabilis

| | | | | `--| ,-- Tsuga_canadensis

`--| |	| |	`----- Nothotsuga_longibracteata

| | `--| ,-- Pseudotsuga_menziesii

| | | ,----- Larix_decidua

| | `--| ,-- Cathaya_argyrophylla

| | `--| ,-- Pinus_contorta

| | `--| ,-- Picea_abies

| | `--| ,-- Schizolepidopsis_ediae

`--|	`--| ,-- Schizolepidopsis_daohugouensis

| `----- Schizolepidopsis_canicularis

| ,-- Sciadopitys_verticillata

| ,--| ,-- Taxus_wallichiana

| | `--| ,-- Cunninghamia_lanceolata

| | `--| ,-- Taiwania_cryptomerioides

| | `--| ,-- Athrotaxis_selaginoides

| | `--| ,-- Sequoia_sempervirens

| | `--| ,-- Cryptomeria_japonica

| | `--| ,-- Cupressus_funebris

`--|	`----- Callitris_muelleri

| ,-- Araucaria_mirabilis

| ,----- Araucaria_heterophylla

| ,--| ,-- Wollemia_nobilis

| | `----- Agathis_australis

| | ,-- Lagarostrobos_franklinii

`--|	,----- Prumnopitys_taxifolia

| ,--| ,-- Lepidothamnus_laxifolius

| | `----- Phyllocladus_aspleniifolius

`--| ,-- Saxegothaea_conspicua

| | ,-- Pherosphaera_hookeriana

`--| ,----- Microcachrys_tetragona

`--| ,-- Acmopyle_pancheri

`--| ,-- Dacrydium_cupressinum

`--| ,-- Retrophyllum_rospigliosii

`----- Podocarpus_archboldii

Tree 95:

,-- Ginkgo_biloba

| ,-- Cordaixylon_dumusum

|--| | ,-- Emporia_lockardii

| | ,----- Hanskerpia_hamiltonensis

`--| | ,-- Voltzia_hexagona

| | | ,-- Nothodacrium_warrenii

`--| | ,----- Rissikia_media

| | | ,-- Alvinia_bohemica_plant

`--| |	,--| ,-- **New_total_plant_from_Arkansas**

| | | `----- Pararaucaria_patagonica

| | ,--| ,-- Himeriella_muensteri

`--| | `--| ,-- Kachaikestrobus_acuminatus

| | `----- Tomaxellia_biforme

| | ,-- Telemachus_aequatus

| | | ,-- Eathiestrobus_mackenziei

`--| |	| ,-- Cedrus_deodara

| | ,--| | ,-- Keteleeria_davidiana

| | | | | ,----- Abies_lasiocarpa

| | | `--| ,--| ,-- Mataia_podocarpoides

| | | | | `--| ,-- Pseudolarix_amabilis

`--| |	| |	`--| ,-- Tsuga_canadensis

| | `--| `----- Nothotsuga_longibracteata

| | | ,-- Pseudotsuga_menziesii

| | | ,----- Larix_decidua

| | `--| ,-- Cathaya_argyrophylla

| | `--| ,-- Pinus_contorta

| | `--| ,-- Picea_abies

`--|	`--| ,-- Schizolepidopsis_ediae

| `--| ,-- Schizolepidopsis_daohugouensis

| `----- Schizolepidopsis_canicularis

| ,-- Sciadopitys_verticillata

| ,--| ,-- Taxus_wallichiana

| | `--| ,-- Cunninghamia_lanceolata

| | `--| ,-- Taiwania_cryptomerioides

| | `--| ,-- Athrotaxis_selaginoides

| | `--| ,-- Sequoia_sempervirens

| | `--| ,-- Cryptomeria_japonica

| | `--| ,-- Cupressus_funebris

`--|	`----- Callitris_muelleri

| ,-- Araucaria_mirabilis

| ,----- Araucaria_heterophylla

| ,--| ,-- Wollemia_nobilis

| | `----- Agathis_australis

| | ,-- Lagarostrobos_franklinii

`--|	,----- Prumnopitys_taxifolia

| ,--| ,-- Lepidothamnus_laxifolius

| | `----- Phyllocladus_aspleniifolius

`--| ,-- Saxegothaea_conspicua

| | ,-- Pherosphaera_hookeriana

`--| ,----- Microcachrys_tetragona

`--| ,-- Acmopyle_pancheri

`--| ,-- Dacrydium_cupressinum

`--| ,-- Retrophyllum_rospigliosii

`----- Podocarpus_archboldii

Tree 96:

,-- Ginkgo_biloba

| ,-- Cordaixylon_dumusum

|--| | ,-- Emporia_lockardii

| | ,----- Hanskerpia_hamiltonensis

`--| | ,-- Voltzia_hexagona

| | | ,-- Nothodacrium_warrenii

`--| | ,----- Rissikia_media

| | | ,-- Pararaucaria_patagonica

`--| |	,--| ,-- **New_total_plant_from_Arkansas**

| | | `----- Alvinia_bohemica_plant

| | ,--| ,-- Himeriella_muensteri

`--| | `--| ,-- Kachaikestrobus_acuminatus

| | `----- Tomaxellia_biforme

| | ,-- Telemachus_aequatus

| | | ,-- Eathiestrobus_mackenziei

`--| |	| ,-- Cedrus_deodara

| | ,--| | ,-- Keteleeria_davidiana

| | | | | ,----- Abies_lasiocarpa

| | | `--| ,--| ,-- Mataia_podocarpoides

| | | | | `--| ,-- Pseudolarix_amabilis

`--| |	| |	`--| ,-- Tsuga_canadensis

| | `--| `----- Nothotsuga_longibracteata

| | | ,-- Pseudotsuga_menziesii

| | | ,----- Larix_decidua

| | `--| ,-- Cathaya_argyrophylla

| | `--| ,-- Pinus_contorta

| | `--| ,-- Picea_abies

`--|	`--| ,-- Schizolepidopsis_ediae

| `--| ,-- Schizolepidopsis_daohugouensis

| `----- Schizolepidopsis_canicularis

| ,-- Sciadopitys_verticillata

| ,--| ,-- Taxus_wallichiana

| | `--| ,-- Cunninghamia_lanceolata

| | `--| ,-- Taiwania_cryptomerioides

| | `--| ,-- Athrotaxis_selaginoides

| | `--| ,-- Sequoia_sempervirens

| | `--| ,-- Cryptomeria_japonica

| | `--| ,-- Cupressus_funebris

`--|	`----- Callitris_muelleri

| ,-- Araucaria_mirabilis

| ,----- Araucaria_heterophylla

| ,--| ,-- Wollemia_nobilis

| | `----- Agathis_australis

| | ,-- Lagarostrobos_franklinii

`--|	,----- Prumnopitys_taxifolia

| ,--| ,-- Lepidothamnus_laxifolius

| | `----- Phyllocladus_aspleniifolius

`--| ,-- Saxegothaea_conspicua

`--| ,-- Microcachrys_tetragona

`--| ,-- Pherosphaera_hookeriana

`--| ,-- Acmopyle_pancheri

`--| ,-- Dacrydium_cupressinum

`--| ,-- Retrophyllum_rospigliosii

`----- Podocarpus_archboldii

Tree 97:

,-- Ginkgo_biloba

| ,-- Cordaixylon_dumusum

|--| | ,-- Emporia_lockardii

| | ,----- Hanskerpia_hamiltonensis

`--| | ,-- Voltzia_hexagona

| | | ,-- Nothodacrium_warrenii

`--| | ,----- Rissikia_media

| | | ,-- Pararaucaria_patagonica

`--| |	,--| ,-- **New_total_plant_from_Arkansas**

| | | `----- Alvinia_bohemica_plant

| | ,--| ,-- Himeriella_muensteri

`--| | `--| ,-- Kachaikestrobus_acuminatus

| | `----- Tomaxellia_biforme

| | ,-- Telemachus_aequatus

| | | ,-- Eathiestrobus_mackenziei

`--| |	| ,-- Cedrus_deodara

| | ,--| | ,-- Keteleeria_davidiana

| | | | | ,----- Abies_lasiocarpa

| | | `--| ,--| ,-- Mataia_podocarpoides

| | | | | `--| ,-- Pseudolarix_amabilis

`--| |	| |	`--| ,-- Tsuga_canadensis

| | `--| `----- Nothotsuga_longibracteata

| | | ,-- Pseudotsuga_menziesii

| | | ,----- Larix_decidua

| | `--| ,-- Cathaya_argyrophylla

| | `--| ,-- Pinus_contorta

| | `--| ,-- Picea_abies

`--|	`--| ,-- Schizolepidopsis_ediae

| `--| ,-- Schizolepidopsis_daohugouensis

| `----- Schizolepidopsis_canicularis

| ,-- Sciadopitys_verticillata

| ,--| ,-- Taxus_wallichiana

| | `--| ,-- Cunninghamia_lanceolata

| | `--| ,-- Taiwania_cryptomerioides

| | `--| ,-- Athrotaxis_selaginoides

| | `--| ,-- Sequoia_sempervirens

| | `--| ,-- Cryptomeria_japonica

| | `--| ,-- Cupressus_funebris

`--|	`----- Callitris_muelleri

| ,-- Araucaria_mirabilis

| ,----- Araucaria_heterophylla

| ,--| ,-- Wollemia_nobilis

| | `----- Agathis_australis

| | ,-- Lagarostrobos_franklinii

`--|	,----- Prumnopitys_taxifolia

| ,--| ,-- Lepidothamnus_laxifolius

| | `----- Phyllocladus_aspleniifolius

`--| ,-- Saxegothaea_conspicua

| | ,-- Pherosphaera_hookeriana

`--| ,----- Microcachrys_tetragona

`--| ,-- Acmopyle_pancheri

`--| ,-- Dacrydium_cupressinum

`--| ,-- Retrophyllum_rospigliosii

`----- Podocarpus_archboldii

Tree 98:

,-- Ginkgo_biloba

| ,-- Cordaixylon_dumusum

|--| | ,-- Emporia_lockardii

| | ,----- Hanskerpia_hamiltonensis

`--| | ,-- Voltzia_hexagona

| | | ,-- Nothodacrium_warrenii

`--| | ,----- Rissikia_media

| | | ,-- Pararaucaria_patagonica

`--| |	,--| ,-- **New_total_plant_from_Arkansas**

| | | `----- Alvinia_bohemica_plant

| | ,--| ,-- Himeriella_muensteri

`--| | `--| ,-- Kachaikestrobus_acuminatus

| | `----- Tomaxellia_biforme

| | ,-- Telemachus_aequatus

| | | ,-- Eathiestrobus_mackenziei

| | | | ,-- Cedrus_deodara

`--| |	| |	,-- Keteleeria_davidiana

| | ,--| | ,--| ,-- Mataia_podocarpoides

| | | | | | `----- Abies_lasiocarpa

| | | `--| ,--| ,-- Pseudolarix_amabilis

| | | | | `--| ,-- Tsuga_canadensis

`--| |	| |	`----- Nothotsuga_longibracteata

| | `--| ,-- Pseudotsuga_menziesii

| | | ,----- Larix_decidua

| | `--| ,-- Cathaya_argyrophylla

| | `--| ,-- Pinus_contorta

| | `--| ,-- Picea_abies

| | `--| ,-- Schizolepidopsis_ediae

`--|	`--| ,-- Schizolepidopsis_daohugouensis

| `----- Schizolepidopsis_canicularis

| ,-- Sciadopitys_verticillata

| ,--| ,-- Taxus_wallichiana

| | `--| ,-- Cunninghamia_lanceolata

| | `--| ,-- Taiwania_cryptomerioides

| | `--| ,-- Athrotaxis_selaginoides

| | `--| ,-- Sequoia_sempervirens

| | `--| ,-- Cryptomeria_japonica

| | `--| ,-- Cupressus_funebris

`--|	`----- Callitris_muelleri

| ,-- Araucaria_mirabilis

| ,----- Araucaria_heterophylla

| ,--| ,-- Wollemia_nobilis

| | `----- Agathis_australis

| | ,-- Lagarostrobos_franklinii

`--|	,----- Prumnopitys_taxifolia

| ,--| ,-- Lepidothamnus_laxifolius

| | `----- Phyllocladus_aspleniifolius

`--| ,-- Saxegothaea_conspicua

| | ,-- Pherosphaera_hookeriana

`--| ,----- Microcachrys_tetragona

`--| ,-- Acmopyle_pancheri

`--| ,-- Dacrydium_cupressinum

`--| ,-- Retrophyllum_rospigliosii

`----- Podocarpus_archboldii

Tree 99:

,-- Ginkgo_biloba

| ,-- Cordaixylon_dumusum

|--| | ,-- Emporia_lockardii

| | ,----- Hanskerpia_hamiltonensis

`--| | ,-- Voltzia_hexagona

| | | ,-- Nothodacrium_warrenii

`--| | ,----- Rissikia_media

| | | ,-- Pararaucaria_patagonica

`--| |	,--| ,-- **New_total_plant_from_Arkansas**

| | | `----- Alvinia_bohemica_plant

| | ,--| ,-- Himeriella_muensteri

`--| | `--| ,-- Kachaikestrobus_acuminatus

| | `----- Tomaxellia_biforme

| | ,-- Telemachus_aequatus

| | | ,-- Eathiestrobus_mackenziei

`--| |	| ,-- Cedrus_deodara

| | ,--| | ,-- Keteleeria_davidiana

| | | | | ,----- Abies_lasiocarpa

| | | `--| ,--| ,-- Pseudolarix_amabilis

| | | | | `--| ,-- Tsuga_canadensis

`--| |	| |	`--| ,-- Mataia_podocarpoides

| | `--| `----- Nothotsuga_longibracteata

| | | ,-- Pseudotsuga_menziesii

| | | ,----- Larix_decidua

| | `--| ,-- Cathaya_argyrophylla

| | `--| ,-- Pinus_contorta

| | `--| ,-- Picea_abies

`--|	`--| ,-- Schizolepidopsis_ediae

| `--| ,-- Schizolepidopsis_daohugouensis

| `----- Schizolepidopsis_canicularis

| ,-- Sciadopitys_verticillata

| ,--| ,-- Taxus_wallichiana

| | `--| ,-- Cunninghamia_lanceolata

| | `--| ,-- Taiwania_cryptomerioides

| | `--| ,-- Athrotaxis_selaginoides

| | `--| ,-- Sequoia_sempervirens

| | `--| ,-- Cryptomeria_japonica

| | `--| ,-- Cupressus_funebris

`--|	`----- Callitris_muelleri

| ,-- Araucaria_mirabilis

| ,----- Araucaria_heterophylla

| ,--| ,-- Wollemia_nobilis

| | `----- Agathis_australis

| | ,-- Lagarostrobos_franklinii

`--|	,----- Prumnopitys_taxifolia

| ,--| ,-- Lepidothamnus_laxifolius

| | `----- Phyllocladus_aspleniifolius

`--| ,-- Saxegothaea_conspicua

| | ,-- Pherosphaera_hookeriana

`--| ,----- Microcachrys_tetragona

`--| ,-- Acmopyle_pancheri

`--| ,-- Dacrydium_cupressinum

`--| ,-- Retrophyllum_rospigliosii

`----- Podocarpus_archboldii

Tree 100:

,-- Ginkgo_biloba

| ,-- Cordaixylon_dumusum

|--| | ,-- Emporia_lockardii

`--| | ,-- Hanskerpia_hamiltonensis

`--| | ,-- Voltzia_hexagona

`--| |	,-- Nothodacrium_warrenii

| | ,----- Rissikia_media

`--| | ,-- Telemachus_aequatus

| | | ,-- Schizolepidopsis_ediae

| | | | ,-- Schizolepidopsis_daohugouensis

`--| |	| ,----- Schizolepidopsis_canicularis

| | ,--| | ,-- Cedrus_deodara

| | | | | | ,-- Keteleeria_davidiana

| | | `--| | ,--| ,-- Mataia_podocarpoides

`--| |	| |	| `----- Abies_lasiocarpa

| | `--| ,--| ,-- Pseudolarix_amabilis

| | | | `--| ,-- Tsuga_canadensis

| | | | `----- Nothotsuga_longibracteata

| | `--| ,-- Pseudotsuga_menziesii

| | | ,----- Larix_decidua

| | `--| ,-- Cathaya_argyrophylla

`--|	`--| ,-- Picea_abies

| `--| ,-- Eathiestrobus_mackenziei

| `----- Pinus_contorta

| ,-- **New_total_plant_from_Arkansas**

| ,----- Alvinia_bohemica_plant

| ,--| ,-- Himeriella_muensteri

| | `--| ,-- Kachaikestrobus_acuminatus

| | `----- Tomaxellia_biforme

| | ,-- Pararaucaria_patagonica

`--| |	,-- Sciadopitys_verticillata

| | ,--| ,-- Taxus_wallichiana

| | | `--| ,-- Cunninghamia_lanceolata

| | | `--| ,-- Taiwania_cryptomerioides

`--| |	`--| ,-- Athrotaxis_selaginoides

| | `--| ,-- Sequoia_sempervirens

| | `--| ,-- Cryptomeria_japonica

| | `--| ,-- Cupressus_funebris

`--|	`----- Callitris_muelleri

| ,-- Araucaria_mirabilis

| ,----- Araucaria_heterophylla

| ,--| ,-- Wollemia_nobilis

| | `----- Agathis_australis

| | ,-- Lagarostrobos_franklinii

`--|	,----- Prumnopitys_taxifolia

| ,--| ,-- Lepidothamnus_laxifolius

| | `----- Phyllocladus_aspleniifolius

`--| ,-- Saxegothaea_conspicua

`--| ,-- Microcachrys_tetragona

`--| ,-- Pherosphaera_hookeriana

`--| ,-- Acmopyle_pancheri

`--| ,-- Dacrydium_cupressinum

`--| ,-- Retrophyllum_rospigliosii

`----- Podocarpus_archboldii

Tree 101:

,-- Ginkgo_biloba

| ,-- Cordaixylon_dumusum

|--| | ,-- Emporia_lockardii

`--| | ,-- Hanskerpia_hamiltonensis

`--| | ,-- Voltzia_hexagona

`--| |	,-- Nothodacrium_warrenii

| | ,----- Rissikia_media

`--| | ,-- Telemachus_aequatus

| | | ,-- Schizolepidopsis_ediae

| | | | ,-- Schizolepidopsis_daohugouensis

`--| |	| ,----- Schizolepidopsis_canicularis

| | ,--| | ,-- Cedrus_deodara

| | | | | | ,-- Keteleeria_davidiana

| | | `--| | ,----- Abies_lasiocarpa

`--| |	| |	|	,-- Mataia_podocarpoides

| | `--| ,--| ,----- Pseudolarix_amabilis

| | | | `--| ,-- Tsuga_canadensis

| | | | `----- Nothotsuga_longibracteata

| | `--| ,-- Pseudotsuga_menziesii

| | | ,----- Larix_decidua

| | `--| ,-- Cathaya_argyrophylla

`--|	`--| ,-- Picea_abies

| `--| ,-- Eathiestrobus_mackenziei

| `----- Pinus_contorta

| ,-- **New_total_plant_from_Arkansas**

| ,----- Alvinia_bohemica_plant

| ,--| ,-- Himeriella_muensteri

| | `--| ,-- Kachaikestrobus_acuminatus

| | `----- Tomaxellia_biforme

| | ,-- Pararaucaria_patagonica

`--| |	,-- Sciadopitys_verticillata

| | ,--| ,-- Taxus_wallichiana

| | | `--| ,-- Cunninghamia_lanceolata

| | | `--| ,-- Taiwania_cryptomerioides

`--| |	`--| ,-- Athrotaxis_selaginoides

| | `--| ,-- Sequoia_sempervirens

| | `--| ,-- Cryptomeria_japonica

| | `--| ,-- Cupressus_funebris

`--|	`----- Callitris_muelleri

| ,-- Araucaria_mirabilis

| ,----- Araucaria_heterophylla

| ,--| ,-- Wollemia_nobilis

| | `----- Agathis_australis

| | ,-- Lagarostrobos_franklinii

`--|	,----- Prumnopitys_taxifolia

| ,--| ,-- Lepidothamnus_laxifolius

| | `----- Phyllocladus_aspleniifolius

`--| ,-- Saxegothaea_conspicua

`--| ,-- Microcachrys_tetragona

`--| ,-- Pherosphaera_hookeriana

`--| ,-- Acmopyle_pancheri

`--| ,-- Dacrydium_cupressinum

`--| ,-- Retrophyllum_rospigliosii

`----- Podocarpus_archboldii

Tree 102:

,-- Ginkgo_biloba

| ,-- Cordaixylon_dumusum

|--| | ,-- Emporia_lockardii

`--| | ,-- Hanskerpia_hamiltonensis

`--| | ,-- Voltzia_hexagona

`--| |	,-- Nothodacrium_warrenii

| | ,----- Rissikia_media

`--| | ,-- Telemachus_aequatus

| | | ,-- Schizolepidopsis_ediae

| | | | ,-- Schizolepidopsis_daohugouensis

`--| | ,--| ,----- Schizolepidopsis_canicularis

| | | | | ,-- Cedrus_deodara

| | | `--| | ,-- Keteleeria_davidiana

| | | | | ,----- Abies_lasiocarpa

`--| |	`--| ,--| ,-- Pseudolarix_amabilis

| | | | `--| ,-- Mataia_podocarpoides

| | | | `--| ,-- Tsuga_canadensis

| | `--| `----- Nothotsuga_longibracteata

| | | ,-- Pseudotsuga_menziesii

| | | ,----- Larix_decidua

`--|	`--| ,-- Cathaya_argyrophylla

| `--| ,-- Picea_abies

| `--| ,-- Eathiestrobus_mackenziei

| `----- Pinus_contorta

| ,-- **New_total_plant_from_Arkansas**

| ,----- Alvinia_bohemica_plant

| ,--| ,-- Himeriella_muensteri

| | `--| ,-- Kachaikestrobus_acuminatus

| | `----- Tomaxellia_biforme

| | ,-- Pararaucaria_patagonica

`--| |	,-- Sciadopitys_verticillata

| | ,--| ,-- Taxus_wallichiana

| | | `--| ,-- Cunninghamia_lanceolata

| | | `--| ,-- Taiwania_cryptomerioides

`--| |	`--| ,-- Athrotaxis_selaginoides

| | `--| ,-- Sequoia_sempervirens

| | `--| ,-- Cryptomeria_japonica

| | `--| ,-- Cupressus_funebris

`--|	`----- Callitris_muelleri

| ,-- Araucaria_mirabilis

| ,----- Araucaria_heterophylla

| ,--| ,-- Wollemia_nobilis

| | `----- Agathis_australis

| | ,-- Lagarostrobos_franklinii

`--|	,----- Prumnopitys_taxifolia

| ,--| ,-- Lepidothamnus_laxifolius

| | `----- Phyllocladus_aspleniifolius

`--| ,-- Saxegothaea_conspicua

`--| ,-- Microcachrys_tetragona

`--| ,-- Pherosphaera_hookeriana

`--| ,-- Acmopyle_pancheri

`--| ,-- Dacrydium_cupressinum

`--| ,-- Retrophyllum_rospigliosii

`----- Podocarpus_archboldii

Tree 103:

,-- Ginkgo_biloba

| ,-- Cordaixylon_dumusum

|--| | ,-- Emporia_lockardii

`--| | ,-- Hanskerpia_hamiltonensis

`--| | ,-- Voltzia_hexagona

`--| |	,-- Nothodacrium_warrenii

| | ,----- Rissikia_media

`--| | ,-- Telemachus_aequatus

| | | ,-- Schizolepidopsis_ediae

| | | | ,-- Schizolepidopsis_daohugouensis

`--| | ,--| ,----- Schizolepidopsis_canicularis

| | | | | ,-- Cedrus_deodara

| | | `--| | ,-- Keteleeria_davidiana

| | | | | ,----- Abies_lasiocarpa

`--| |	`--| ,--| ,-- Pseudolarix_amabilis

| | | | `--| ,-- Tsuga_canadensis

| | | | `--| ,-- Mataia_podocarpoides

| | `--| `----- Nothotsuga_longibracteata

| | | ,-- Pseudotsuga_menziesii

| | | ,----- Larix_decidua

`--|	`--| ,-- Cathaya_argyrophylla

| `--| ,-- Picea_abies

| `--| ,-- Eathiestrobus_mackenziei

| `----- Pinus_contorta

| ,-- **New_total_plant_from_Arkansas**

| ,----- Alvinia_bohemica_plant

| ,--| ,-- Himeriella_muensteri

| | `--| ,-- Kachaikestrobus_acuminatus

| | `----- Tomaxellia_biforme

| | ,-- Pararaucaria_patagonica

`--| |	,-- Sciadopitys_verticillata

| | ,--| ,-- Taxus_wallichiana

| | | `--| ,-- Cunninghamia_lanceolata

| | | `--| ,-- Taiwania_cryptomerioides

`--| |	`--| ,-- Athrotaxis_selaginoides

| | `--| ,-- Sequoia_sempervirens

| | `--| ,-- Cryptomeria_japonica

| | `--| ,-- Cupressus_funebris

`--|	`----- Callitris_muelleri

| ,-- Araucaria_mirabilis

| ,----- Araucaria_heterophylla

| ,--| ,-- Wollemia_nobilis

| | `----- Agathis_australis

| | ,-- Lagarostrobos_franklinii

`--|	,----- Prumnopitys_taxifolia

| ,--| ,-- Lepidothamnus_laxifolius

| | `----- Phyllocladus_aspleniifolius

`--| ,-- Saxegothaea_conspicua

`--| ,-- Microcachrys_tetragona

`--| ,-- Pherosphaera_hookeriana

`--| ,-- Acmopyle_pancheri

`--| ,-- Dacrydium_cupressinum

`--| ,-- Retrophyllum_rospigliosii

`----- Podocarpus_archboldii

Tree 104:

,-- Ginkgo_biloba

| ,-- Cordaixylon_dumusum

|--| | ,-- Emporia_lockardii

`--| | ,-- Hanskerpia_hamiltonensis

`--| | ,-- Voltzia_hexagona

`--| |	,-- Nothodacrium_warrenii

| | ,----- Rissikia_media

`--| | ,-- Telemachus_aequatus

| | | ,-- Schizolepidopsis_ediae

| | | | ,-- Schizolepidopsis_daohugouensis

`--| | ,--| ,----- Schizolepidopsis_canicularis

| | | | | ,-- Cedrus_deodara

| | | `--| | ,-- Keteleeria_davidiana

| | | | | ,----- Abies_lasiocarpa

`--| |	`--| ,--| ,-- Mataia_podocarpoides

| | | | `--| ,-- Pseudolarix_amabilis

| | | | `--| ,-- Tsuga_canadensis

| | `--| `----- Nothotsuga_longibracteata

| | | ,-- Pseudotsuga_menziesii

| | | ,----- Larix_decidua

`--|	`--| ,-- Cathaya_argyrophylla

| `--| ,-- Picea_abies

| `--| ,-- Eathiestrobus_mackenziei

| `----- Pinus_contorta

| ,-- **New_total_plant_from_Arkansas**

| ,----- Alvinia_bohemica_plant

| ,--| ,-- Himeriella_muensteri

| | `--| ,-- Kachaikestrobus_acuminatus

| | `----- Tomaxellia_biforme

| | ,-- Pararaucaria_patagonica

`--| |	,-- Sciadopitys_verticillata

| | ,--| ,-- Taxus_wallichiana

| | | `--| ,-- Cunninghamia_lanceolata

| | | `--| ,-- Taiwania_cryptomerioides

`--| |	`--| ,-- Athrotaxis_selaginoides

| | `--| ,-- Sequoia_sempervirens

| | `--| ,-- Cryptomeria_japonica

| | `--| ,-- Cupressus_funebris

`--|	`----- Callitris_muelleri

| ,-- Araucaria_mirabilis

| ,----- Araucaria_heterophylla

| ,--| ,-- Wollemia_nobilis

| | `----- Agathis_australis

| | ,-- Lagarostrobos_franklinii

`--|	,----- Prumnopitys_taxifolia

| ,--| ,-- Lepidothamnus_laxifolius

| | `----- Phyllocladus_aspleniifolius

`--| ,-- Saxegothaea_conspicua

`--| ,-- Microcachrys_tetragona

`--| ,-- Pherosphaera_hookeriana

`--| ,-- Acmopyle_pancheri

`--| ,-- Dacrydium_cupressinum

`--| ,-- Retrophyllum_rospigliosii

`----- Podocarpus_archboldii

Tree 105:

,-- Ginkgo_biloba

| ,-- Cordaixylon_dumusum

|--| | ,-- Emporia_lockardii

`--| | ,-- Hanskerpia_hamiltonensis

`--| | ,-- Voltzia_hexagona

`--| |	,-- Nothodacrium_warrenii

| | ,----- Rissikia_media

`--| | ,-- Telemachus_aequatus

| | | ,-- Schizolepidopsis_ediae

| | | | ,-- Schizolepidopsis_daohugouensis

`--| |	| ,----- Schizolepidopsis_canicularis

| | ,--| | ,-- Cedrus_deodara

| | | | | | ,-- Mataia_podocarpoides

| | | `--| | ,--| ,-- Keteleeria_davidiana

`--| |	| |	| `----- Abies_lasiocarpa

| | `--| ,--| ,-- Pseudolarix_amabilis

| | | | `--| ,-- Tsuga_canadensis

| | | | `----- Nothotsuga_longibracteata

| | `--| ,-- Pseudotsuga_menziesii

| | | ,----- Larix_decidua

| | `--| ,-- Cathaya_argyrophylla

`--|	`--| ,-- Picea_abies

| `--| ,-- Eathiestrobus_mackenziei

| `----- Pinus_contorta

| ,-- **New_total_plant_from_Arkansas**

| ,----- Alvinia_bohemica_plant

| ,--| ,-- Himeriella_muensteri

| | `--| ,-- Kachaikestrobus_acuminatus

| | `----- Tomaxellia_biforme

| | ,-- Pararaucaria_patagonica

`--| |	,-- Sciadopitys_verticillata

| | ,--| ,-- Taxus_wallichiana

| | | `--| ,-- Cunninghamia_lanceolata

| | | `--| ,-- Taiwania_cryptomerioides

`--| |	`--| ,-- Athrotaxis_selaginoides

| | `--| ,-- Sequoia_sempervirens

| | `--| ,-- Cryptomeria_japonica

| | `--| ,-- Cupressus_funebris

`--|	`----- Callitris_muelleri

| ,-- Araucaria_mirabilis

| ,----- Araucaria_heterophylla

| ,--| ,-- Wollemia_nobilis

| | `----- Agathis_australis

| | ,-- Lagarostrobos_franklinii

`--|	,----- Prumnopitys_taxifolia

| ,--| ,-- Lepidothamnus_laxifolius

| | `----- Phyllocladus_aspleniifolius

`--| ,-- Saxegothaea_conspicua

`--| ,-- Microcachrys_tetragona

`--| ,-- Pherosphaera_hookeriana

`--| ,-- Acmopyle_pancheri

`--| ,-- Dacrydium_cupressinum

`--| ,-- Retrophyllum_rospigliosii

`----- Podocarpus_archboldii

Tree 106:

,-- Ginkgo_biloba

| ,-- Cordaixylon_dumusum

|--| | ,-- Emporia_lockardii

`--| ,----- Hanskerpia_hamiltonensis

| | ,-- Voltzia_hexagona

`--| |	,-- Nothodacrium_warrenii

| | ,----- Rissikia_media

`--| | ,-- Telemachus_aequatus

| | | ,-- Schizolepidopsis_ediae

| | | | ,-- Schizolepidopsis_daohugouensis

`--| |	| ,----- Schizolepidopsis_canicularis

| | ,--| | ,-- Cedrus_deodara

| | | | | | ,-- Keteleeria_davidiana

| | | `--| | ,--| ,-- Mataia_podocarpoides

`--| |	| |	| `----- Abies_lasiocarpa

| | `--| ,--| ,-- Pseudolarix_amabilis

| | | | `--| ,-- Tsuga_canadensis

| | | | `----- Nothotsuga_longibracteata

| | `--| ,-- Pseudotsuga_menziesii

| | | ,----- Larix_decidua

| | `--| ,-- Cathaya_argyrophylla

`--|	`--| ,-- Picea_abies

| `--| ,-- Eathiestrobus_mackenziei

| `----- Pinus_contorta

| ,-- **New_total_plant_from_Arkansas**

| ,----- Alvinia_bohemica_plant

| ,--| ,-- Himeriella_muensteri

| | `--| ,-- Kachaikestrobus_acuminatus

| | `----- Tomaxellia_biforme

| | ,-- Pararaucaria_patagonica

`--| |	,-- Sciadopitys_verticillata

| | ,--| ,-- Taxus_wallichiana

| | | `--| ,-- Cunninghamia_lanceolata

| | | `--| ,-- Taiwania_cryptomerioides

`--| |	`--| ,-- Athrotaxis_selaginoides

| | `--| ,-- Sequoia_sempervirens

| | `--| ,-- Cryptomeria_japonica

| | `--| ,-- Cupressus_funebris

`--|	`----- Callitris_muelleri

| ,-- Araucaria_mirabilis

| ,----- Araucaria_heterophylla

| ,--| ,-- Wollemia_nobilis

| | `----- Agathis_australis

| | ,-- Lagarostrobos_franklinii

`--|	,----- Prumnopitys_taxifolia

| ,--| ,-- Lepidothamnus_laxifolius

| | `----- Phyllocladus_aspleniifolius

`--| ,-- Saxegothaea_conspicua

`--| ,-- Microcachrys_tetragona

`--| ,-- Pherosphaera_hookeriana

`--| ,-- Acmopyle_pancheri

`--| ,-- Dacrydium_cupressinum

`--| ,-- Retrophyllum_rospigliosii

`----- Podocarpus_archboldii

Tree 107:

,-- Ginkgo_biloba

| ,-- Cordaixylon_dumusum

|--| | ,-- Emporia_lockardii

`--| ,----- Hanskerpia_hamiltonensis

| | ,-- Voltzia_hexagona

`--| |	,-- Nothodacrium_warrenii

| | ,----- Rissikia_media

`--| | ,-- Telemachus_aequatus

| | | ,-- Schizolepidopsis_ediae

| | | | ,-- Schizolepidopsis_daohugouensis

`--| |	| ,----- Schizolepidopsis_canicularis

| | ,--| | ,-- Cedrus_deodara

| | | | | | ,-- Keteleeria_davidiana

| | | `--| | ,----- Abies_lasiocarpa

`--| |	| |	|	,-- Mataia_podocarpoides

| | `--| ,--| ,----- Pseudolarix_amabilis

| | | | `--| ,-- Tsuga_canadensis

| | | | `----- Nothotsuga_longibracteata

| | `--| ,-- Pseudotsuga_menziesii

| | | ,----- Larix_decidua

| | `--| ,-- Cathaya_argyrophylla

`--|	`--| ,-- Picea_abies

| `--| ,-- Eathiestrobus_mackenziei

| `----- Pinus_contorta

| ,-- **New_total_plant_from_Arkansas**

| ,----- Alvinia_bohemica_plant

| ,--| ,-- Himeriella_muensteri

| | `--| ,-- Kachaikestrobus_acuminatus

| | `----- Tomaxellia_biforme

| | ,-- Pararaucaria_patagonica

`--| |	,-- Sciadopitys_verticillata

| | ,--| ,-- Taxus_wallichiana

| | | `--| ,-- Cunninghamia_lanceolata

| | | `--| ,-- Taiwania_cryptomerioides

`--| |	`--| ,-- Athrotaxis_selaginoides

| | `--| ,-- Sequoia_sempervirens

| | `--| ,-- Cryptomeria_japonica

| | `--| ,-- Cupressus_funebris

`--|	`----- Callitris_muelleri

| ,-- Araucaria_mirabilis

| ,----- Araucaria_heterophylla

| ,--| ,-- Wollemia_nobilis

| | `----- Agathis_australis

| | ,-- Lagarostrobos_franklinii

`--|	,----- Prumnopitys_taxifolia

| ,--| ,-- Lepidothamnus_laxifolius

| | `----- Phyllocladus_aspleniifolius

`--| ,-- Saxegothaea_conspicua

`--| ,-- Microcachrys_tetragona

`--| ,-- Pherosphaera_hookeriana

`--| ,-- Acmopyle_pancheri

`--| ,-- Dacrydium_cupressinum

`--| ,-- Retrophyllum_rospigliosii

`----- Podocarpus_archboldii

Tree 108:

,-- Ginkgo_biloba

| ,-- Cordaixylon_dumusum

|--| | ,-- Emporia_lockardii

`--| ,----- Hanskerpia_hamiltonensis

| | ,-- Voltzia_hexagona

`--| |	,-- Nothodacrium_warrenii

| | ,----- Rissikia_media

`--| | ,-- Telemachus_aequatus

| | | ,-- Schizolepidopsis_ediae

| | | | ,-- Schizolepidopsis_daohugouensis

`--| | ,--| ,----- Schizolepidopsis_canicularis

| | | | | ,-- Cedrus_deodara

| | | `--| | ,-- Keteleeria_davidiana

| | | | | ,----- Abies_lasiocarpa

`--| |	`--| ,--| ,-- Pseudolarix_amabilis

| | | | `--| ,-- Mataia_podocarpoides

| | | | `--| ,-- Tsuga_canadensis

| | `--| `----- Nothotsuga_longibracteata

| | | ,-- Pseudotsuga_menziesii

| | | ,----- Larix_decidua

`--|	`--| ,-- Cathaya_argyrophylla

| `--| ,-- Picea_abies

| `--| ,-- Eathiestrobus_mackenziei

| `----- Pinus_contorta

| ,-- **New_total_plant_from_Arkansas**

| ,----- Alvinia_bohemica_plant

| ,--| ,-- Himeriella_muensteri

| | `--| ,-- Kachaikestrobus_acuminatus

| | `----- Tomaxellia_biforme

| | ,-- Pararaucaria_patagonica

`--| |	,-- Sciadopitys_verticillata

| | ,--| ,-- Taxus_wallichiana

| | | `--| ,-- Cunninghamia_lanceolata

| | | `--| ,-- Taiwania_cryptomerioides

`--| |	`--| ,-- Athrotaxis_selaginoides

| | `--| ,-- Sequoia_sempervirens

| | `--| ,-- Cryptomeria_japonica

| | `--| ,-- Cupressus_funebris

`--|	`----- Callitris_muelleri

| ,-- Araucaria_mirabilis

| ,----- Araucaria_heterophylla

| ,--| ,-- Wollemia_nobilis

| | `----- Agathis_australis

| | ,-- Lagarostrobos_franklinii

`--|	,----- Prumnopitys_taxifolia

| ,--| ,-- Lepidothamnus_laxifolius

| | `----- Phyllocladus_aspleniifolius

`--| ,-- Saxegothaea_conspicua

`--| ,-- Microcachrys_tetragona

`--| ,-- Pherosphaera_hookeriana

`--| ,-- Acmopyle_pancheri

`--| ,-- Dacrydium_cupressinum

`--| ,-- Retrophyllum_rospigliosii

`----- Podocarpus_archboldii

Tree 109:

,-- Ginkgo_biloba

| ,-- Cordaixylon_dumusum

|--| | ,-- Emporia_lockardii

`--| ,----- Hanskerpia_hamiltonensis

| | ,-- Voltzia_hexagona

`--| |	,-- Nothodacrium_warrenii

| | ,----- Rissikia_media

`--| | ,-- Telemachus_aequatus

| | | ,-- Schizolepidopsis_ediae

| | | | ,-- Schizolepidopsis_daohugouensis

`--| | ,--| ,----- Schizolepidopsis_canicularis

| | | | | ,-- Cedrus_deodara

| | | `--| | ,-- Keteleeria_davidiana

| | | | | ,----- Abies_lasiocarpa

`--| |	`--| ,--| ,-- Pseudolarix_amabilis

| | | | `--| ,-- Tsuga_canadensis

| | | | `--| ,-- Mataia_podocarpoides

| | `--| `----- Nothotsuga_longibracteata

| | | ,-- Pseudotsuga_menziesii

| | | ,----- Larix_decidua

`--|	`--| ,-- Cathaya_argyrophylla

| `--| ,-- Picea_abies

| `--| ,-- Eathiestrobus_mackenziei

| `----- Pinus_contorta

| ,-- **New_total_plant_from_Arkansas**

| ,----- Alvinia_bohemica_plant

| ,--| ,-- Himeriella_muensteri

| | `--| ,-- Kachaikestrobus_acuminatus

| | `----- Tomaxellia_biforme

| | ,-- Pararaucaria_patagonica

`--| |	,-- Sciadopitys_verticillata

| | ,--| ,-- Taxus_wallichiana

| | | `--| ,-- Cunninghamia_lanceolata

| | | `--| ,-- Taiwania_cryptomerioides

`--| |	`--| ,-- Athrotaxis_selaginoides

| | `--| ,-- Sequoia_sempervirens

| | `--| ,-- Cryptomeria_japonica

| | `--| ,-- Cupressus_funebris

`--|	`----- Callitris_muelleri

| ,-- Araucaria_mirabilis

| ,----- Araucaria_heterophylla

| ,--| ,-- Wollemia_nobilis

| | `----- Agathis_australis

| | ,-- Lagarostrobos_franklinii

`--|	,----- Prumnopitys_taxifolia

| ,--| ,-- Lepidothamnus_laxifolius

| | `----- Phyllocladus_aspleniifolius

`--| ,-- Saxegothaea_conspicua

`--| ,-- Microcachrys_tetragona

`--| ,-- Pherosphaera_hookeriana

`--| ,-- Acmopyle_pancheri

`--| ,-- Dacrydium_cupressinum

`--| ,-- Retrophyllum_rospigliosii

`----- Podocarpus_archboldii

Tree 110:

,-- Ginkgo_biloba

| ,-- Cordaixylon_dumusum

|--| | ,-- Emporia_lockardii

`--| ,----- Hanskerpia_hamiltonensis

| | ,-- Voltzia_hexagona

`--| |	,-- Nothodacrium_warrenii

| | ,----- Rissikia_media

`--| | ,-- Telemachus_aequatus

| | | ,-- Schizolepidopsis_ediae

| | | | ,-- Schizolepidopsis_daohugouensis

`--| | ,--| ,----- Schizolepidopsis_canicularis

| | | | | ,-- Cedrus_deodara

| | | `--| | ,-- Keteleeria_davidiana

| | | | | ,----- Abies_lasiocarpa

`--| |	`--| ,--| ,-- Mataia_podocarpoides

| | | | `--| ,-- Pseudolarix_amabilis

| | | | `--| ,-- Tsuga_canadensis

| | `--| `----- Nothotsuga_longibracteata

| | | ,-- Pseudotsuga_menziesii

| | | ,----- Larix_decidua

`--|	`--| ,-- Cathaya_argyrophylla

| `--| ,-- Picea_abies

| `--| ,-- Eathiestrobus_mackenziei

| `----- Pinus_contorta

| ,-- **New_total_plant_from_Arkansas**

| ,----- Alvinia_bohemica_plant

| ,--| ,-- Himeriella_muensteri

| | `--| ,-- Kachaikestrobus_acuminatus

| | `----- Tomaxellia_biforme

| | ,-- Pararaucaria_patagonica

`--| |	,-- Sciadopitys_verticillata

| | ,--| ,-- Taxus_wallichiana

| | | `--| ,-- Cunninghamia_lanceolata

| | | `--| ,-- Taiwania_cryptomerioides

`--| |	`--| ,-- Athrotaxis_selaginoides

| | `--| ,-- Sequoia_sempervirens

| | `--| ,-- Cryptomeria_japonica

| | `--| ,-- Cupressus_funebris

`--|	`----- Callitris_muelleri

| ,-- Araucaria_mirabilis

| ,----- Araucaria_heterophylla

| ,--| ,-- Wollemia_nobilis

| | `----- Agathis_australis

| | ,-- Lagarostrobos_franklinii

`--|	,----- Prumnopitys_taxifolia

| ,--| ,-- Lepidothamnus_laxifolius

| | `----- Phyllocladus_aspleniifolius

`--| ,-- Saxegothaea_conspicua

`--| ,-- Microcachrys_tetragona

`--| ,-- Pherosphaera_hookeriana

`--| ,-- Acmopyle_pancheri

`--| ,-- Dacrydium_cupressinum

`--| ,-- Retrophyllum_rospigliosii

`----- Podocarpus_archboldii

Tree 111:

,-- Ginkgo_biloba

| ,-- Cordaixylon_dumusum

|--| | ,-- Emporia_lockardii

`--| ,----- Hanskerpia_hamiltonensis

| | ,-- Voltzia_hexagona

`--| |	,-- Nothodacrium_warrenii

| | ,----- Rissikia_media

`--| | ,-- Telemachus_aequatus

| | | ,-- Schizolepidopsis_ediae

| | | | ,-- Schizolepidopsis_daohugouensis

`--| |	| ,----- Schizolepidopsis_canicularis

| | ,--| | ,-- Cedrus_deodara

| | | | | | ,-- Mataia_podocarpoides

| | | `--| | ,--| ,-- Keteleeria_davidiana

`--| |	| |	| `----- Abies_lasiocarpa

| | `--| ,--| ,-- Pseudolarix_amabilis

| | | | `--| ,-- Tsuga_canadensis

| | | | `----- Nothotsuga_longibracteata

| | `--| ,-- Pseudotsuga_menziesii

| | | ,----- Larix_decidua

| | `--| ,-- Cathaya_argyrophylla

`--|	`--| ,-- Picea_abies

| `--| ,-- Eathiestrobus_mackenziei

| `----- Pinus_contorta

| ,-- **New_total_plant_from_Arkansas**

| ,----- Alvinia_bohemica_plant

| ,--| ,-- Himeriella_muensteri

| | `--| ,-- Kachaikestrobus_acuminatus

| | `----- Tomaxellia_biforme

| | ,-- Pararaucaria_patagonica

`--| |	,-- Sciadopitys_verticillata

| | ,--| ,-- Taxus_wallichiana

| | | `--| ,-- Cunninghamia_lanceolata

| | | `--| ,-- Taiwania_cryptomerioides

`--| |	`--| ,-- Athrotaxis_selaginoides

| | `--| ,-- Sequoia_sempervirens

| | `--| ,-- Cryptomeria_japonica

| | `--| ,-- Cupressus_funebris

`--|	`----- Callitris_muelleri

| ,-- Araucaria_mirabilis

| ,----- Araucaria_heterophylla

| ,--| ,-- Wollemia_nobilis

| | `----- Agathis_australis

| | ,-- Lagarostrobos_franklinii

`--|	,----- Prumnopitys_taxifolia

| ,--| ,-- Lepidothamnus_laxifolius

| | `----- Phyllocladus_aspleniifolius

`--| ,-- Saxegothaea_conspicua

`--| ,-- Microcachrys_tetragona

`--| ,-- Pherosphaera_hookeriana

`--| ,-- Acmopyle_pancheri

`--| ,-- Dacrydium_cupressinum

`--| ,-- Retrophyllum_rospigliosii

`----- Podocarpus_archboldii

**Character 64 active and treated as additive, constrain of Pinaceae main clades off (all most parsimonious trees)**

Tree 0:

,-- Ginkgo_biloba

| ,-- Cordaixylon_dumusum

|--| | ,-- Emporia_lockardii

| | ,----- Hanskerpia_hamiltonensis

`--| | ,-- Voltzia_hexagona

| | | ,-- Nothodacrium_warrenii

`--| | ,----- Rissikia_media

| | | ,-- Telemachus_aequatus

`--| | |	,-- Schizolepidopsis_ediae

| | | ,--| ,-- Schizolepidopsis_daohugouensis

| | | | `----- Schizolepidopsis_canicularis

`--| |	| ,-- Cedrus_deodara

| | ,--| | ,-- Mataia_podocarpoides

| | | | | | ,-- Keteleeria_davidiana

| | | `--| ,--| ,----- Abies_lasiocarpa

`--| |	| | `--| ,-- Pseudolarix_amabilis

| | | | `--| ,-- Tsuga_canadensis

| | `--| `----- Nothotsuga_longibracteata

| | | ,-- Pseudotsuga_menziesii

| | | ,----- Larix_decidua

| | `--| ,-- Cathaya_argyrophylla

| | `--| ,-- Picea_abies

| | `--| ,-- Eathiestrobus_mackenziei

`--|	`----- Pinus_contorta

| ,-- Sciadopitys_verticillata

| ,--| ,-- Taxus_wallichiana

| | `--| ,-- Cunninghamia_lanceolata

| | `--| ,-- Taiwania_cryptomerioides

| | `--| ,-- Athrotaxis_selaginoides

| | `--| ,-- Sequoia_sempervirens

| | `--| ,-- Cryptomeria_japonica

| | `--| ,-- Cupressus_funebris

| | `----- Callitris_muelleri

`--|	,-- Pararaucaria_patagonica

| | ,-- **New_total_plant_from_Arkansas**

| ,--| ,----- Alvinia_bohemica_plant

| | `--| ,-- Himeriella_muensteri

| | `--| ,-- Kachaikestrobus_acuminatus

| | `----- Tomaxellia_biforme

| | ,-- Araucaria_mirabilis

`--|	,----- Araucaria_heterophylla

| ,--| ,-- Wollemia_nobilis

| | `----- Agathis_australis

| | ,-- Lagarostrobos_franklinii

`--|	,----- Prumnopitys_taxifolia

| ,--| ,-- Lepidothamnus_laxifolius

| | `----- Phyllocladus_aspleniifolius

`--| ,-- Saxegothaea_conspicua

`--| ,-- Microcachrys_tetragona

`--| ,-- Pherosphaera_hookeriana

`--| ,-- Acmopyle_pancheri

`--| ,-- Dacrydium_cupressinum

`--| ,-- Retrophyllum_rospigliosii

`----- Podocarpus_archboldii

Tree 1:

,-- Ginkgo_biloba

| ,-- Cordaixylon_dumusum

|--| | ,-- Emporia_lockardii

| | ,----- Hanskerpia_hamiltonensis

`--| | ,-- Voltzia_hexagona

| | | ,-- Nothodacrium_warrenii

`--| | ,----- Rissikia_media

| | | ,-- Alvinia_bohemica_plant

`--| |	,--| ,-- **New_total_plant_from_Arkansas**

| | | `----- Pararaucaria_patagonica

| | ,--| ,-- Himeriella_muensteri

`--| | `--| ,-- Kachaikestrobus_acuminatus

| | `----- Tomaxellia_biforme

| | ,-- Telemachus_aequatus

| | | ,-- Eathiestrobus_mackenziei

`--| |	| ,-- Cedrus_deodara

| | ,--| | ,-- Mataia_podocarpoides

| | | `--| | ,-- Keteleeria_davidiana

| | | | | ,----- Abies_lasiocarpa

| | | `--| ,--| ,-- Pseudolarix_amabilis

`--| |	| | `--| ,-- Tsuga_canadensis

| | | | `----- Nothotsuga_longibracteata

| | `--| ,-- Pseudotsuga_menziesii

| | | ,----- Larix_decidua

| | `--| ,-- Cathaya_argyrophylla

| | `--| ,-- Pinus_contorta

| | `--| ,-- Picea_abies

`--|	`--| ,-- Schizolepidopsis_ediae

| `--| ,-- Schizolepidopsis_daohugouensis

| `----- Schizolepidopsis_canicularis

| ,-- Sciadopitys_verticillata

| ,--| ,-- Taxus_wallichiana

| | `--| ,-- Cunninghamia_lanceolata

| | `--| ,-- Taiwania_cryptomerioides

| | `--| ,-- Athrotaxis_selaginoides

| | `--| ,-- Sequoia_sempervirens

| | `--| ,-- Cryptomeria_japonica

| | `--| ,-- Cupressus_funebris

`--|	`----- Callitris_muelleri

| ,-- Araucaria_mirabilis

| ,----- Araucaria_heterophylla

| ,--| ,-- Wollemia_nobilis

| | `----- Agathis_australis

| | ,-- Lagarostrobos_franklinii

`--|	,----- Prumnopitys_taxifolia

| ,--| ,-- Lepidothamnus_laxifolius

| | `----- Phyllocladus_aspleniifolius

`--| ,-- Saxegothaea_conspicua

`--| ,-- Microcachrys_tetragona

`--| ,-- Pherosphaera_hookeriana

`--| ,-- Acmopyle_pancheri

`--| ,-- Dacrydium_cupressinum

`--| ,-- Retrophyllum_rospigliosii

`----- Podocarpus_archboldii

Tree 2:

,-- Ginkgo_biloba

| ,-- Cordaixylon_dumusum

|--| | ,-- Emporia_lockardii

| | ,----- Hanskerpia_hamiltonensis

`--| | ,-- Voltzia_hexagona

| | | ,-- Nothodacrium_warrenii

`--| | ,----- Rissikia_media

| | | ,-- **New_total_plant_from_Arkansas**

`--| |	,--| ,-- Alvinia_bohemica_plant

| | | `----- Pararaucaria_patagonica

| | ,--| ,-- Himeriella_muensteri

`--| | `--| ,-- Kachaikestrobus_acuminatus

| | `----- Tomaxellia_biforme

| | ,-- Telemachus_aequatus

| | | ,-- Eathiestrobus_mackenziei

`--| |	| ,-- Cedrus_deodara

| | ,--| | ,-- Pseudotsuga_menziesii

| | | | | ,----- Larix_decidua

| | | `--| ,--| ,-- Cathaya_argyrophylla

| | | | | `--| ,-- Pinus_contorta

`--| |	| |	`----- Picea_abies

| | `--| ,-- Mataia_podocarpoides

| | | ,--| ,-- Keteleeria_davidiana

| | | | `----- Abies_lasiocarpa

| | `--| ,-- Pseudolarix_amabilis

| | | | ,-- Tsuga_canadensis

| | `--| ,----- Nothotsuga_longibracteata

`--|	`--| ,-- Schizolepidopsis_ediae

| `--| ,-- Schizolepidopsis_daohugouensis

| `----- Schizolepidopsis_canicularis

| ,-- Sciadopitys_verticillata

| ,--| ,-- Taxus_wallichiana

| | `--| ,-- Cunninghamia_lanceolata

| | `--| ,-- Taiwania_cryptomerioides

| | `--| ,-- Athrotaxis_selaginoides

| | `--| ,-- Sequoia_sempervirens

| | `--| ,-- Cryptomeria_japonica

| | `--| ,-- Cupressus_funebris

`--|	`----- Callitris_muelleri

| ,-- Araucaria_mirabilis

| ,----- Araucaria_heterophylla

| ,--| ,-- Wollemia_nobilis

| | `----- Agathis_australis

| | ,-- Lagarostrobos_franklinii

`--|	,----- Prumnopitys_taxifolia

| ,--| ,-- Lepidothamnus_laxifolius

| | `----- Phyllocladus_aspleniifolius

`--| ,-- Saxegothaea_conspicua

`--| ,-- Microcachrys_tetragona

`--| ,-- Pherosphaera_hookeriana

`--| ,-- Acmopyle_pancheri

`--| ,-- Dacrydium_cupressinum

`--| ,-- Retrophyllum_rospigliosii

`----- Podocarpus_archboldii

Tree 3:

,-- Ginkgo_biloba

| ,-- Cordaixylon_dumusum

|--| | ,-- Emporia_lockardii

| | ,----- Hanskerpia_hamiltonensis

`--| | ,-- Voltzia_hexagona

| | | ,-- Nothodacrium_warrenii

`--| | ,----- Rissikia_media

| | | ,-- Pararaucaria_patagonica

`--| |	|	,-- **New_total_plant_from_Arkansas**

| | ,--| ,----- Alvinia_bohemica_plant

| | | `--| ,-- Himeriella_muensteri

`--| |	`--| ,-- Kachaikestrobus_acuminatus

| | `----- Tomaxellia_biforme

| | ,-- Telemachus_aequatus

| | | ,-- Eathiestrobus_mackenziei

`--| |	| ,-- Cedrus_deodara

| | ,--| | ,-- Mataia_podocarpoides

| | | `--| | ,-- Keteleeria_davidiana

| | | | | ,----- Abies_lasiocarpa

| | | `--| ,--| ,-- Pseudolarix_amabilis

`--| |	| | `--| ,-- Tsuga_canadensis

| | | | `----- Nothotsuga_longibracteata

| | `--| ,-- Pseudotsuga_menziesii

| | | ,----- Larix_decidua

| | `--| ,-- Cathaya_argyrophylla

| | `--| ,-- Pinus_contorta

| | `--| ,-- Picea_abies

`--|	`--| ,-- Schizolepidopsis_ediae

| `--| ,-- Schizolepidopsis_daohugouensis

| `----- Schizolepidopsis_canicularis

| ,-- Sciadopitys_verticillata

| ,--| ,-- Taxus_wallichiana

| | `--| ,-- Cunninghamia_lanceolata

| | `--| ,-- Taiwania_cryptomerioides

| | `--| ,-- Athrotaxis_selaginoides

| | `--| ,-- Sequoia_sempervirens

| | `--| ,-- Cryptomeria_japonica

| | `--| ,-- Cupressus_funebris

`--|	`----- Callitris_muelleri

| ,-- Araucaria_mirabilis

| ,----- Araucaria_heterophylla

| ,--| ,-- Wollemia_nobilis

| | `----- Agathis_australis

| | ,-- Lagarostrobos_franklinii

`--|	,----- Prumnopitys_taxifolia

| ,--| ,-- Lepidothamnus_laxifolius

| | `----- Phyllocladus_aspleniifolius

`--| ,-- Saxegothaea_conspicua

`--| ,-- Microcachrys_tetragona

`--| ,-- Pherosphaera_hookeriana

`--| ,-- Acmopyle_pancheri

`--| ,-- Dacrydium_cupressinum

`--| ,-- Retrophyllum_rospigliosii

`----- Podocarpus_archboldii

Tree 4:

,-- Ginkgo_biloba

| ,-- Cordaixylon_dumusum

|--| | ,-- Emporia_lockardii

| | ,----- Hanskerpia_hamiltonensis

`--| | ,-- Voltzia_hexagona

| | | ,-- Nothodacrium_warrenii

`--| | ,----- Rissikia_media

| | | ,-- Pararaucaria_patagonica

`--| |	,--| ,-- **New_total_plant_from_Arkansas**

| | | `----- Alvinia_bohemica_plant

| | ,--| ,-- Himeriella_muensteri

`--| | `--| ,-- Kachaikestrobus_acuminatus

| | `----- Tomaxellia_biforme

| | ,-- Telemachus_aequatus

| | | ,-- Eathiestrobus_mackenziei

`--| |	| ,-- Cedrus_deodara

| | ,--| | ,-- Pseudotsuga_menziesii

| | | | | ,----- Larix_decidua

| | | `--| ,--| ,-- Cathaya_argyrophylla

| | | | | `--| ,-- Pinus_contorta

`--| |	| |	`----- Picea_abies

| | `--| ,-- Mataia_podocarpoides

| | | ,--| ,-- Keteleeria_davidiana

| | | | `----- Abies_lasiocarpa

| | `--| ,-- Pseudolarix_amabilis

| | | | ,-- Tsuga_canadensis

| | `--| ,----- Nothotsuga_longibracteata

`--|	`--| ,-- Schizolepidopsis_ediae

| `--| ,-- Schizolepidopsis_daohugouensis

| `----- Schizolepidopsis_canicularis

| ,-- Sciadopitys_verticillata

| ,--| ,-- Taxus_wallichiana

| | `--| ,-- Cunninghamia_lanceolata

| | `--| ,-- Taiwania_cryptomerioides

| | `--| ,-- Athrotaxis_selaginoides

| | `--| ,-- Sequoia_sempervirens

| | `--| ,-- Cryptomeria_japonica

| | `--| ,-- Cupressus_funebris

`--|	`----- Callitris_muelleri

| ,-- Araucaria_mirabilis

| ,----- Araucaria_heterophylla

| ,--| ,-- Wollemia_nobilis

| | `----- Agathis_australis

| | ,-- Lagarostrobos_franklinii

`--|	,----- Prumnopitys_taxifolia

| ,--| ,-- Lepidothamnus_laxifolius

| | `----- Phyllocladus_aspleniifolius

`--| ,-- Saxegothaea_conspicua

`--| ,-- Microcachrys_tetragona

`--| ,-- Pherosphaera_hookeriana

`--| ,-- Acmopyle_pancheri

`--| ,-- Dacrydium_cupressinum

`--| ,-- Retrophyllum_rospigliosii

`----- Podocarpus_archboldii

Tree 5:

,-- Ginkgo_biloba

| ,-- Cordaixylon_dumusum

|--| | ,-- Emporia_lockardii

| | ,----- Hanskerpia_hamiltonensis

`--| | ,-- Voltzia_hexagona

| | | ,-- Nothodacrium_warrenii

`--| | ,----- Rissikia_media

| | | ,-- Telemachus_aequatus

`--| | |	,-- Schizolepidopsis_ediae

| | | ,--| ,-- Schizolepidopsis_daohugouensis

| | | | `----- Schizolepidopsis_canicularis

`--| |	| ,-- Cedrus_deodara

| | ,--| | ,-- Keteleeria_davidiana

| | | | | ,----- Abies_lasiocarpa

| | | `--| ,--| ,-- Pseudolarix_amabilis

`--| |	| | `--| ,-- Mataia_podocarpoides

| | | | `--| ,-- Tsuga_canadensis

| | `--| `----- Nothotsuga_longibracteata

| | | ,-- Pseudotsuga_menziesii

| | | ,----- Larix_decidua

| | `--| ,-- Cathaya_argyrophylla

| | `--| ,-- Pinus_contorta

| | `--| ,-- Eathiestrobus_mackenziei

`--|	`----- Picea_abies

| ,-- Sciadopitys_verticillata

| ,--| ,-- Taxus_wallichiana

| | `--| ,-- Cunninghamia_lanceolata

| | `--| ,-- Taiwania_cryptomerioides

| | `--| ,-- Athrotaxis_selaginoides

| | `--| ,-- Sequoia_sempervirens

| | `--| ,-- Cryptomeria_japonica

| | `--| ,-- Cupressus_funebris

| | `----- Callitris_muelleri

`--|	,-- Pararaucaria_patagonica

| | ,-- **New_total_plant_from_Arkansas**

| ,--| ,----- Alvinia_bohemica_plant

| | `--| ,-- Himeriella_muensteri

| | `--| ,-- Kachaikestrobus_acuminatus

| | `----- Tomaxellia_biforme

| | ,-- Araucaria_mirabilis

`--|	,----- Araucaria_heterophylla

| ,--| ,-- Wollemia_nobilis

| | `----- Agathis_australis

| | ,-- Lagarostrobos_franklinii

`--|	,----- Prumnopitys_taxifolia

| ,--| ,-- Lepidothamnus_laxifolius

| | `----- Phyllocladus_aspleniifolius

`--| ,-- Saxegothaea_conspicua

`--| ,-- Microcachrys_tetragona

`--| ,-- Pherosphaera_hookeriana

`--| ,-- Acmopyle_pancheri

`--| ,-- Dacrydium_cupressinum

`--| ,-- Retrophyllum_rospigliosii

`----- Podocarpus_archboldii

Tree 6:

,-- Ginkgo_biloba

| ,-- Mataia_podocarpoides

| | ,-- Eathiestrobus_mackenziei

| | | ,-- Cedrus_deodara

| | ,--| | ,-- Pseudotsuga_menziesii

|--| | | | | ,----- Larix_decidua

| | | `--| ,--| ,-- Cathaya_argyrophylla

| | | | | `--| ,-- Pinus_contorta

| | | | | `----- Picea_abies

`--| |	`--|	,-- Keteleeria_davidiana

| | | ,----- Abies_lasiocarpa

| | | | ,-- Pseudolarix_amabilis

| | `--| | ,-- Tsuga_canadensis

| | `--| ,----- Nothotsuga_longibracteata

| | `--| ,-- Schizolepidopsis_ediae

| | `--| ,-- Schizolepidopsis_daohugouensis

| | `----- Schizolepidopsis_canicularis

`--|	,-- Araucaria_mirabilis

| ,----- Araucaria_heterophylla

| ,--| ,-- Wollemia_nobilis

| | `----- Agathis_australis

| | ,-- Lagarostrobos_franklinii

| ,--| ,----- Prumnopitys_taxifolia

| | | ,--| ,-- Lepidothamnus_laxifolius

| | | | `----- Phyllocladus_aspleniifolius

| | `--| ,-- Saxegothaea_conspicua

| | `--| ,-- Microcachrys_tetragona

| | `--| ,-- Pherosphaera_hookeriana

| | `--| ,-- Acmopyle_pancheri

| | `--| ,-- Dacrydium_cupressinum

`--|	`--| ,-- Retrophyllum_rospigliosii

| `----- Podocarpus_archboldii

| ,-- Pararaucaria_patagonica

| | ,-- **New_total_plant_from_Arkansas**

| ,--| ,----- Alvinia_bohemica_plant

| | `--| ,-- Himeriella_muensteri

| | `--| ,-- Kachaikestrobus_acuminatus

| | `----- Tomaxellia_biforme

`--| ,-- Sciadopitys_verticillata

| | ,-- Cunninghamia_lanceolata

| | ,--| ,-- Taiwania_cryptomerioides

`--| | `--| ,-- Athrotaxis_selaginoides

| | `--| ,-- Sequoia_sempervirens

| | `--| ,-- Cryptomeria_japonica

`--|	`--| ,-- Cupressus_funebris

| `----- Callitris_muelleri

| ,-- Telemachus_aequatus

| | ,-- Nothodacrium_warrenii

`--| ,----- Rissikia_media

| | ,-- Voltzia_hexagona

`--| |	,-- Emporia_lockardii

`--| ,----- Hanskerpia_hamiltonensis

`--| ,-- Cordaixylon_dumusum

`----- Taxus_wallichiana

Tree 7:

,-- Ginkgo_biloba

| ,-- Cordaixylon_dumusum

|--| | ,-- Emporia_lockardii

| | ,----- Hanskerpia_hamiltonensis

`--| | ,-- Voltzia_hexagona

| | | ,-- Nothodacrium_warrenii

`--| | ,----- Rissikia_media

| | | ,-- Telemachus_aequatus

`--| | |	,-- Schizolepidopsis_ediae

| | | ,--| ,-- Schizolepidopsis_daohugouensis

| | | | `----- Schizolepidopsis_canicularis

`--| |	| ,-- Cedrus_deodara

| | ,--| | ,-- Mataia_podocarpoides

| | | | | | ,-- Keteleeria_davidiana

| | | `--| ,--| ,----- Abies_lasiocarpa

`--| |	| | `--| ,-- Pseudolarix_amabilis

| | | | `--| ,-- Tsuga_canadensis

| | `--| `----- Nothotsuga_longibracteata

| | | ,-- Pseudotsuga_menziesii

| | | ,----- Larix_decidua

| | `--| ,-- Cathaya_argyrophylla

| | `--| ,-- Pinus_contorta

| | `--| ,-- Eathiestrobus_mackenziei

`--|	`----- Picea_abies

| ,-- Sciadopitys_verticillata

| ,--| ,-- Taxus_wallichiana

| | `--| ,-- Cunninghamia_lanceolata

| | `--| ,-- Taiwania_cryptomerioides

| | `--| ,-- Athrotaxis_selaginoides

| | `--| ,-- Sequoia_sempervirens

| | `--| ,-- Cryptomeria_japonica

| | `--| ,-- Cupressus_funebris

| | `----- Callitris_muelleri

`--|	,-- Pararaucaria_patagonica

| | ,-- **New_total_plant_from_Arkansas**

| ,--| ,----- Alvinia_bohemica_plant

| | `--| ,-- Himeriella_muensteri

| | `--| ,-- Kachaikestrobus_acuminatus

| | `----- Tomaxellia_biforme

| | ,-- Araucaria_mirabilis

`--|	,----- Araucaria_heterophylla

| ,--| ,-- Wollemia_nobilis

| | `----- Agathis_australis

| | ,-- Lagarostrobos_franklinii

`--|	,----- Prumnopitys_taxifolia

| ,--| ,-- Lepidothamnus_laxifolius

| | `----- Phyllocladus_aspleniifolius

`--| ,-- Saxegothaea_conspicua

`--| ,-- Microcachrys_tetragona

`--| ,-- Pherosphaera_hookeriana

`--| ,-- Acmopyle_pancheri

`--| ,-- Dacrydium_cupressinum

`--| ,-- Retrophyllum_rospigliosii

`----- Podocarpus_archboldii

Tree 8:

,-- Ginkgo_biloba

| ,-- Cordaixylon_dumusum

|--| | ,-- Emporia_lockardii

| | ,----- Hanskerpia_hamiltonensis

`--| | ,-- Voltzia_hexagona

| | | ,-- Nothodacrium_warrenii

`--| | ,----- Rissikia_media

| | | ,-- Pararaucaria_patagonica

`--| |	|	,-- **New_total_plant_from_Arkansas**

| | ,--| ,----- Alvinia_bohemica_plant

| | | `--| ,-- Himeriella_muensteri

`--| |	`--| ,-- Kachaikestrobus_acuminatus

| | `----- Tomaxellia_biforme

| | ,-- Telemachus_aequatus

| | | ,-- Eathiestrobus_mackenziei

`--| |	| ,-- Cedrus_deodara

| | ,--| | ,-- Pseudotsuga_menziesii

| | | | | ,----- Larix_decidua

| | | `--| ,--| ,-- Cathaya_argyrophylla

| | | | | `--| ,-- Pinus_contorta

`--| |	| |	`----- Picea_abies

| | `--| ,-- Mataia_podocarpoides

| | | ,--| ,-- Keteleeria_davidiana

| | | | `----- Abies_lasiocarpa

| | `--| ,-- Pseudolarix_amabilis

| | | | ,-- Tsuga_canadensis

| | `--| ,----- Nothotsuga_longibracteata

`--|	`--| ,-- Schizolepidopsis_ediae

| `--| ,-- Schizolepidopsis_daohugouensis

| `----- Schizolepidopsis_canicularis

| ,-- Sciadopitys_verticillata

| ,--| ,-- Taxus_wallichiana

| | `--| ,-- Cunninghamia_lanceolata

| | `--| ,-- Taiwania_cryptomerioides

| | `--| ,-- Athrotaxis_selaginoides

| | `--| ,-- Sequoia_sempervirens

| | `--| ,-- Cryptomeria_japonica

| | `--| ,-- Cupressus_funebris

`--|	`----- Callitris_muelleri

| ,-- Araucaria_mirabilis

| ,----- Araucaria_heterophylla

| ,--| ,-- Wollemia_nobilis

| | `----- Agathis_australis

| | ,-- Lagarostrobos_franklinii

`--|	,----- Prumnopitys_taxifolia

| ,--| ,-- Lepidothamnus_laxifolius

| | `----- Phyllocladus_aspleniifolius

`--| ,-- Saxegothaea_conspicua

`--| ,-- Microcachrys_tetragona

`--| ,-- Pherosphaera_hookeriana

`--| ,-- Acmopyle_pancheri

`--| ,-- Dacrydium_cupressinum

`--| ,-- Retrophyllum_rospigliosii

`----- Podocarpus_archboldii

Tree 9:

,-- Ginkgo_biloba

| ,-- Cordaixylon_dumusum

|--| | ,-- Emporia_lockardii

| | ,----- Hanskerpia_hamiltonensis

`--| | ,-- Voltzia_hexagona

| | | ,-- Nothodacrium_warrenii

`--| | ,----- Rissikia_media

| | | ,-- Telemachus_aequatus

`--| | |	,-- Schizolepidopsis_ediae

| | | ,--| ,-- Schizolepidopsis_daohugouensis

| | | | `----- Schizolepidopsis_canicularis

`--| |	| ,-- Cedrus_deodara

| | ,--| | ,-- Keteleeria_davidiana

| | | | | ,----- Abies_lasiocarpa

| | | `--| ,--| ,-- Pseudolarix_amabilis

`--| |	| | `--| ,-- Mataia_podocarpoides

| | | | `--| ,-- Tsuga_canadensis

| | `--| `----- Nothotsuga_longibracteata

| | | ,-- Pseudotsuga_menziesii

| | | ,----- Larix_decidua

| | `--| ,-- Cathaya_argyrophylla

| | `--| ,-- Picea_abies

| | `--| ,-- Eathiestrobus_mackenziei

`--|	`----- Pinus_contorta

| ,-- Sciadopitys_verticillata

| ,--| ,-- Taxus_wallichiana

| | `--| ,-- Cunninghamia_lanceolata

| | `--| ,-- Taiwania_cryptomerioides

| | `--| ,-- Athrotaxis_selaginoides

| | `--| ,-- Sequoia_sempervirens

| | `--| ,-- Cryptomeria_japonica

| | `--| ,-- Cupressus_funebris

| | `----- Callitris_muelleri

`--|	,-- Pararaucaria_patagonica

| | ,-- **New_total_plant_from_Arkansas**

| ,--| ,----- Alvinia_bohemica_plant

| | `--| ,-- Himeriella_muensteri

| | `--| ,-- Kachaikestrobus_acuminatus

| | `----- Tomaxellia_biforme

| | ,-- Araucaria_mirabilis

`--|	,----- Araucaria_heterophylla

| ,--| ,-- Wollemia_nobilis

| | `----- Agathis_australis

| | ,-- Lagarostrobos_franklinii

`--|	,----- Prumnopitys_taxifolia

| ,--| ,-- Lepidothamnus_laxifolius

| | `----- Phyllocladus_aspleniifolius

`--| ,-- Saxegothaea_conspicua

`--| ,-- Microcachrys_tetragona

`--| ,-- Pherosphaera_hookeriana

`--| ,-- Acmopyle_pancheri

`--| ,-- Dacrydium_cupressinum

`--| ,-- Retrophyllum_rospigliosii

`----- Podocarpus_archboldii

Tree 10:

,-- Ginkgo_biloba

| ,-- Eathiestrobus_mackenziei

| | ,-- Cedrus_deodara

| ,--| | ,-- Keteleeria_davidiana

| | | | ,----- Abies_lasiocarpa

| | `--| ,--| ,-- Pseudolarix_amabilis

| | | | `--| ,-- Tsuga_canadensis

|--| | | | `----- Nothotsuga_longibracteata

| | `--| ,-- Pseudotsuga_menziesii

| | | ,----- Larix_decidua

| | `--| ,-- Cathaya_argyrophylla

| | `--| ,-- Pinus_contorta

| | `--| ,-- Picea_abies

`--|	`--| ,-- Schizolepidopsis_ediae

| `--| ,-- Schizolepidopsis_daohugouensis

| `----- Schizolepidopsis_canicularis

| ,-- Mataia_podocarpoides

| | ,-- Araucaria_mirabilis

| | ,----- Araucaria_heterophylla

| | ,--| ,-- Wollemia_nobilis

| | | `----- Agathis_australis

| | | ,-- Lagarostrobos_franklinii

| | ,--| ,----- Prumnopitys_taxifolia

`--| | | ,--| ,-- Lepidothamnus_laxifolius

| | | | `----- Phyllocladus_aspleniifolius

| | `--| ,-- Saxegothaea_conspicua

| | `--| ,-- Microcachrys_tetragona

| | `--| ,-- Pherosphaera_hookeriana

| | `--| ,-- Acmopyle_pancheri

| | `--| ,-- Dacrydium_cupressinum

`--|	`--| ,-- Retrophyllum_rospigliosii

| `----- Podocarpus_archboldii

| ,-- Pararaucaria_patagonica

| | ,-- **New_total_plant_from_Arkansas**

| ,--| ,----- Alvinia_bohemica_plant

| | `--| ,-- Himeriella_muensteri

| | `--| ,-- Kachaikestrobus_acuminatus

| | `----- Tomaxellia_biforme

`--| ,-- Sciadopitys_verticillata

| | ,-- Cunninghamia_lanceolata

| | ,--| ,-- Taiwania_cryptomerioides

`--| | `--| ,-- Athrotaxis_selaginoides

| | `--| ,-- Sequoia_sempervirens

| | `--| ,-- Cryptomeria_japonica

`--|	`--| ,-- Cupressus_funebris

| `----- Callitris_muelleri

| ,-- Telemachus_aequatus

| | ,-- Nothodacrium_warrenii

`--| ,----- Rissikia_media

| | ,-- Voltzia_hexagona

`--| |	,-- Emporia_lockardii

`--| ,----- Hanskerpia_hamiltonensis

`--| ,-- Cordaixylon_dumusum

`----- Taxus_wallichiana

Tree 11:

,-- Ginkgo_biloba

| ,-- Mataia_podocarpoides

| | ,-- Cedrus_deodara

| | | ,-- Keteleeria_davidiana

| | | ,----- Abies_lasiocarpa

|--| | ,--| ,--| ,-- Pseudolarix_amabilis

| | | | | `--| ,-- Tsuga_canadensis

| | | | | `----- Nothotsuga_longibracteata

| | | `--| ,-- Pseudotsuga_menziesii

| | | | ,----- Larix_decidua

`--| |	| | ,-- Cathaya_argyrophylla

| | `--| | ,-- Eathiestrobus_mackenziei

| | `--| ,----- Pinus_contorta

| | `--| ,-- Picea_abies

| | `--| ,-- Schizolepidopsis_ediae

| | `--| ,-- Schizolepidopsis_daohugouensis

| | `----- Schizolepidopsis_canicularis

| | ,-- Araucaria_mirabilis

`--|	,----- Araucaria_heterophylla

| ,--| ,-- Wollemia_nobilis

| | `----- Agathis_australis

| | ,-- Lagarostrobos_franklinii

| ,--| ,----- Prumnopitys_taxifolia

| | | ,--| ,-- Lepidothamnus_laxifolius

| | | | `----- Phyllocladus_aspleniifolius

| | `--| ,-- Saxegothaea_conspicua

| | `--| ,-- Microcachrys_tetragona

| | `--| ,-- Pherosphaera_hookeriana

| | `--| ,-- Acmopyle_pancheri

| | `--| ,-- Dacrydium_cupressinum

`--|	`--| ,-- Retrophyllum_rospigliosii

| `----- Podocarpus_archboldii

| ,-- Pararaucaria_patagonica

| | ,-- **New_total_plant_from_Arkansas**

| ,--| ,----- Alvinia_bohemica_plant

| | `--| ,-- Himeriella_muensteri

| | `--| ,-- Kachaikestrobus_acuminatus

| | `----- Tomaxellia_biforme

`--| ,-- Sciadopitys_verticillata

| | ,-- Cunninghamia_lanceolata

| | ,--| ,-- Taiwania_cryptomerioides

`--| | `--| ,-- Athrotaxis_selaginoides

| | `--| ,-- Sequoia_sempervirens

| | `--| ,-- Cryptomeria_japonica

`--|	`--| ,-- Cupressus_funebris

| `----- Callitris_muelleri

| ,-- Telemachus_aequatus

| | ,-- Nothodacrium_warrenii

`--| ,----- Rissikia_media

| | ,-- Voltzia_hexagona

`--| |	,-- Emporia_lockardii

`--| ,----- Hanskerpia_hamiltonensis

`--| ,-- Cordaixylon_dumusum

`----- Taxus_wallichiana

Tree 12:

,-- Ginkgo_biloba

| ,-- Mataia_podocarpoides

| | ,-- Cedrus_deodara

| | | ,-- Pseudotsuga_menziesii

| | | ,----- Larix_decidua

|--| | | ,--| ,-- Cathaya_argyrophylla

| | ,--| | `--| ,-- Pinus_contorta

| | | | | `--| ,-- Eathiestrobus_mackenziei

| | | | | `----- Picea_abies

| | | `--| ,-- Keteleeria_davidiana

`--| |	| ,----- Abies_lasiocarpa

| | | | ,-- Pseudolarix_amabilis

| | `--| | ,-- Tsuga_canadensis

| | `--| ,----- Nothotsuga_longibracteata

| | `--| ,-- Schizolepidopsis_ediae

| | `--| ,-- Schizolepidopsis_daohugouensis

| | `----- Schizolepidopsis_canicularis

| | ,-- Araucaria_mirabilis

`--|	,----- Araucaria_heterophylla

| ,--| ,-- Wollemia_nobilis

| | `----- Agathis_australis

| | ,-- Lagarostrobos_franklinii

| ,--| ,----- Prumnopitys_taxifolia

| | | ,--| ,-- Lepidothamnus_laxifolius

| | | | `----- Phyllocladus_aspleniifolius

| | `--| ,-- Saxegothaea_conspicua

| | `--| ,-- Microcachrys_tetragona

| | `--| ,-- Pherosphaera_hookeriana

| | `--| ,-- Acmopyle_pancheri

| | `--| ,-- Dacrydium_cupressinum

`--|	`--| ,-- Retrophyllum_rospigliosii

| `----- Podocarpus_archboldii

| ,-- Pararaucaria_patagonica

| | ,-- **New_total_plant_from_Arkansas**

| ,--| ,----- Alvinia_bohemica_plant

| | `--| ,-- Himeriella_muensteri

| | `--| ,-- Kachaikestrobus_acuminatus

| | `----- Tomaxellia_biforme

`--| ,-- Sciadopitys_verticillata

| | ,-- Cunninghamia_lanceolata

| | ,--| ,-- Taiwania_cryptomerioides

`--| | `--| ,-- Athrotaxis_selaginoides

| | `--| ,-- Sequoia_sempervirens

| | `--| ,-- Cryptomeria_japonica

`--|	`--| ,-- Cupressus_funebris

| `----- Callitris_muelleri

| ,-- Telemachus_aequatus

| | ,-- Nothodacrium_warrenii

`--| ,----- Rissikia_media

| | ,-- Voltzia_hexagona

`--| |	,-- Emporia_lockardii

`--| ,----- Hanskerpia_hamiltonensis

`--| ,-- Cordaixylon_dumusum

`----- Taxus_wallichiana

Tree 13:

,-- Ginkgo_biloba

| ,-- Cedrus_deodara

| | ,-- Keteleeria_davidiana

| | ,----- Abies_lasiocarpa

| ,--| ,--| ,-- Pseudolarix_amabilis

| | | | `--| ,-- Tsuga_canadensis

| | | | `----- Nothotsuga_longibracteata

|--| | `--| ,-- Pseudotsuga_menziesii

| | | ,----- Larix_decidua

| | | | ,-- Cathaya_argyrophylla

| | `--| | ,-- Eathiestrobus_mackenziei

| | `--| ,----- Pinus_contorta

| | `--| ,-- Picea_abies

| | `--| ,-- Schizolepidopsis_ediae

`--|	`--| ,-- Schizolepidopsis_daohugouensis

| `----- Schizolepidopsis_canicularis

| ,-- Mataia_podocarpoides

| | ,-- Araucaria_mirabilis

| | ,----- Araucaria_heterophylla

| | ,--| ,-- Wollemia_nobilis

| | | `----- Agathis_australis

| | | ,-- Lagarostrobos_franklinii

| | ,--| ,----- Prumnopitys_taxifolia

`--| | | ,--| ,-- Lepidothamnus_laxifolius

| | | | `----- Phyllocladus_aspleniifolius

| | `--| ,-- Saxegothaea_conspicua

| | `--| ,-- Microcachrys_tetragona

| | `--| ,-- Pherosphaera_hookeriana

| | `--| ,-- Acmopyle_pancheri

| | `--| ,-- Dacrydium_cupressinum

`--|	`--| ,-- Retrophyllum_rospigliosii

| `----- Podocarpus_archboldii

| ,-- Pararaucaria_patagonica

| | ,-- **New_total_plant_from_Arkansas**

| ,--| ,----- Alvinia_bohemica_plant

| | `--| ,-- Himeriella_muensteri

| | `--| ,-- Kachaikestrobus_acuminatus

| | `----- Tomaxellia_biforme

`--| ,-- Sciadopitys_verticillata

| | ,-- Cunninghamia_lanceolata

| | ,--| ,-- Taiwania_cryptomerioides

`--| | `--| ,-- Athrotaxis_selaginoides

| | `--| ,-- Sequoia_sempervirens

| | `--| ,-- Cryptomeria_japonica

`--|	`--| ,-- Cupressus_funebris

| `----- Callitris_muelleri

| ,-- Telemachus_aequatus

| | ,-- Nothodacrium_warrenii

`--| ,----- Rissikia_media

| | ,-- Voltzia_hexagona

`--| |	,-- Emporia_lockardii

`--| ,----- Hanskerpia_hamiltonensis

`--| ,-- Cordaixylon_dumusum

`----- Taxus_wallichiana

Tree 14:

,-- Ginkgo_biloba

| ,-- Eathiestrobus_mackenziei

| | ,-- Cedrus_deodara

| ,--| | ,-- Pseudotsuga_menziesii

| | | | ,----- Larix_decidua

| | `--| ,--| ,-- Cathaya_argyrophylla

| | | | `--| ,-- Pinus_contorta

|--| | | | `----- Picea_abies

| | `--| ,-- Keteleeria_davidiana

| | | ,----- Abies_lasiocarpa

| | | | ,-- Pseudolarix_amabilis

| | `--| | ,-- Tsuga_canadensis

| | `--| ,----- Nothotsuga_longibracteata

`--|	`--| ,-- Schizolepidopsis_ediae

| `--| ,-- Schizolepidopsis_daohugouensis

| `----- Schizolepidopsis_canicularis

| ,-- Mataia_podocarpoides

| | ,-- Araucaria_mirabilis

| | ,----- Araucaria_heterophylla

| | ,--| ,-- Wollemia_nobilis

| | | `----- Agathis_australis

| | | ,-- Lagarostrobos_franklinii

| | ,--| ,----- Prumnopitys_taxifolia

`--| | | ,--| ,-- Lepidothamnus_laxifolius

| | | | `----- Phyllocladus_aspleniifolius

| | `--| ,-- Saxegothaea_conspicua

| | `--| ,-- Microcachrys_tetragona

| | `--| ,-- Pherosphaera_hookeriana

| | `--| ,-- Acmopyle_pancheri

| | `--| ,-- Dacrydium_cupressinum

`--|	`--| ,-- Retrophyllum_rospigliosii

| `----- Podocarpus_archboldii

| ,-- Pararaucaria_patagonica

| | ,-- **New_total_plant_from_Arkansas**

| ,--| ,----- Alvinia_bohemica_plant

| | `--| ,-- Himeriella_muensteri

| | `--| ,-- Kachaikestrobus_acuminatus

| | `----- Tomaxellia_biforme

`--| ,-- Sciadopitys_verticillata

| | ,-- Cunninghamia_lanceolata

| | ,--| ,-- Taiwania_cryptomerioides

`--| | `--| ,-- Athrotaxis_selaginoides

| | `--| ,-- Sequoia_sempervirens

| | `--| ,-- Cryptomeria_japonica

`--|	`--| ,-- Cupressus_funebris

| `----- Callitris_muelleri

| ,-- Telemachus_aequatus

| | ,-- Nothodacrium_warrenii

`--| ,----- Rissikia_media

| | ,-- Voltzia_hexagona

`--| |	,-- Emporia_lockardii

`--| ,----- Hanskerpia_hamiltonensis

`--| ,-- Cordaixylon_dumusum

`----- Taxus_wallichiana

Tree 15:

,-- Ginkgo_biloba

| ,-- Mataia_podocarpoides

| | ,-- Cedrus_deodara

| | | ,-- Pseudotsuga_menziesii

| | | ,----- Larix_decidua

|--| | | ,--| ,-- Cathaya_argyrophylla

| | ,--| | `--| ,-- Picea_abies

| | | | | `--| ,-- Eathiestrobus_mackenziei

| | | | | `----- Pinus_contorta

| | | `--| ,-- Keteleeria_davidiana

`--| |	| ,----- Abies_lasiocarpa

| | | | ,-- Pseudolarix_amabilis

| | `--| | ,-- Tsuga_canadensis

| | `--| ,----- Nothotsuga_longibracteata

| | `--| ,-- Schizolepidopsis_ediae

| | `--| ,-- Schizolepidopsis_daohugouensis

| | `----- Schizolepidopsis_canicularis

| | ,-- Araucaria_mirabilis

`--|	,----- Araucaria_heterophylla

| ,--| ,-- Wollemia_nobilis

| | `----- Agathis_australis

| | ,-- Lagarostrobos_franklinii

| ,--| ,----- Prumnopitys_taxifolia

| | | ,--| ,-- Lepidothamnus_laxifolius

| | | | `----- Phyllocladus_aspleniifolius

| | `--| ,-- Saxegothaea_conspicua

| | `--| ,-- Microcachrys_tetragona

| | `--| ,-- Pherosphaera_hookeriana

| | `--| ,-- Acmopyle_pancheri

| | `--| ,-- Dacrydium_cupressinum

`--|	`--| ,-- Retrophyllum_rospigliosii

| `----- Podocarpus_archboldii

| ,-- Pararaucaria_patagonica

| | ,-- **New_total_plant_from_Arkansas**

| ,--| ,----- Alvinia_bohemica_plant

| | `--| ,-- Himeriella_muensteri

| | `--| ,-- Kachaikestrobus_acuminatus

| | `----- Tomaxellia_biforme

`--| ,-- Sciadopitys_verticillata

| | ,-- Cunninghamia_lanceolata

| | ,--| ,-- Taiwania_cryptomerioides

`--| | `--| ,-- Athrotaxis_selaginoides

| | `--| ,-- Sequoia_sempervirens

| | `--| ,-- Cryptomeria_japonica

`--|	`--| ,-- Cupressus_funebris

| `----- Callitris_muelleri

| ,-- Telemachus_aequatus

| | ,-- Nothodacrium_warrenii

`--| ,----- Rissikia_media

| | ,-- Voltzia_hexagona

`--| |	,-- Emporia_lockardii

`--| ,----- Hanskerpia_hamiltonensis

`--| ,-- Cordaixylon_dumusum

`----- Taxus_wallichiana

Tree 16:

,-- Ginkgo_biloba

| ,-- Cedrus_deodara

| | ,-- Pseudotsuga_menziesii

| | ,----- Larix_decidua

| | ,--| ,-- Cathaya_argyrophylla

| ,--| | `--| ,-- Picea_abies

| | | | `--| ,-- Eathiestrobus_mackenziei

|--| | | | `----- Pinus_contorta

| | `--| ,-- Keteleeria_davidiana

| | | ,----- Abies_lasiocarpa

| | | | ,-- Pseudolarix_amabilis

| | `--| | ,-- Tsuga_canadensis

| | `--| ,----- Nothotsuga_longibracteata

| | `--| ,-- Schizolepidopsis_ediae

`--|	`--| ,-- Schizolepidopsis_daohugouensis

| `----- Schizolepidopsis_canicularis

| ,-- Mataia_podocarpoides

| | ,-- Araucaria_mirabilis

| | ,----- Araucaria_heterophylla

| | ,--| ,-- Wollemia_nobilis

| | | `----- Agathis_australis

| | | ,-- Lagarostrobos_franklinii

| | ,--| ,----- Prumnopitys_taxifolia

`--| | | ,--| ,-- Lepidothamnus_laxifolius

| | | | `----- Phyllocladus_aspleniifolius

| | `--| ,-- Saxegothaea_conspicua

| | `--| ,-- Microcachrys_tetragona

| | `--| ,-- Pherosphaera_hookeriana

| | `--| ,-- Acmopyle_pancheri

| | `--| ,-- Dacrydium_cupressinum

`--|	`--| ,-- Retrophyllum_rospigliosii

| `----- Podocarpus_archboldii

| ,-- Pararaucaria_patagonica

| | ,-- **New_total_plant_from_Arkansas**

| ,--| ,----- Alvinia_bohemica_plant

| | `--| ,-- Himeriella_muensteri

| | `--| ,-- Kachaikestrobus_acuminatus

| | `----- Tomaxellia_biforme

`--| ,-- Sciadopitys_verticillata

| | ,-- Cunninghamia_lanceolata

| | ,--| ,-- Taiwania_cryptomerioides

`--| | `--| ,-- Athrotaxis_selaginoides

| | `--| ,-- Sequoia_sempervirens

| | `--| ,-- Cryptomeria_japonica

`--|	`--| ,-- Cupressus_funebris

| `----- Callitris_muelleri

| ,-- Telemachus_aequatus

| | ,-- Nothodacrium_warrenii

`--| ,----- Rissikia_media

| | ,-- Voltzia_hexagona

`--| |	,-- Emporia_lockardii

`--| ,----- Hanskerpia_hamiltonensis

`--| ,-- Cordaixylon_dumusum

`----- Taxus_wallichiana

Tree 17:

,-- Ginkgo_biloba

| ,-- Cordaixylon_dumusum

|--| | ,-- Emporia_lockardii

`--| ,----- Hanskerpia_hamiltonensis

| | ,-- Voltzia_hexagona

`--| |	,-- Nothodacrium_warrenii

| | ,----- Rissikia_media

`--| | ,-- Telemachus_aequatus

| | | ,-- Schizolepidopsis_ediae

| | | | ,-- Schizolepidopsis_daohugouensis

`--| | ,--| ,----- Schizolepidopsis_canicularis

| | | | | ,-- Cedrus_deodara

| | | `--| | ,-- Mataia_podocarpoides

| | | | | | ,-- Keteleeria_davidiana

`--| |	`--| ,--| ,----- Abies_lasiocarpa

| | | | `--| ,-- Pseudolarix_amabilis

| | | | `--| ,-- Tsuga_canadensis

| | `--| `----- Nothotsuga_longibracteata

| | | ,-- Pseudotsuga_menziesii

| | | ,----- Larix_decidua

`--|	`--| ,-- Cathaya_argyrophylla

| `--| ,-- Picea_abies

| `--| ,-- Eathiestrobus_mackenziei

| `----- Pinus_contorta

| ,-- **New_total_plant_from_Arkansas**

| ,----- Alvinia_bohemica_plant

| ,--| ,-- Himeriella_muensteri

| | `--| ,-- Kachaikestrobus_acuminatus

| | `----- Tomaxellia_biforme

| | ,-- Pararaucaria_patagonica

`--| |	,-- Sciadopitys_verticillata

| | ,--| ,-- Taxus_wallichiana

| | | `--| ,-- Cunninghamia_lanceolata

| | | `--| ,-- Taiwania_cryptomerioides

`--| |	`--| ,-- Athrotaxis_selaginoides

| | `--| ,-- Sequoia_sempervirens

| | `--| ,-- Cryptomeria_japonica

| | `--| ,-- Cupressus_funebris

`--|	`----- Callitris_muelleri

| ,-- Araucaria_mirabilis

| ,----- Araucaria_heterophylla

| ,--| ,-- Wollemia_nobilis

| | `----- Agathis_australis

| | ,-- Lagarostrobos_franklinii

`--|	,----- Prumnopitys_taxifolia

| ,--| ,-- Lepidothamnus_laxifolius

| | `----- Phyllocladus_aspleniifolius

`--| ,-- Saxegothaea_conspicua

`--| ,-- Microcachrys_tetragona

`--| ,-- Pherosphaera_hookeriana

`--| ,-- Acmopyle_pancheri

`--| ,-- Dacrydium_cupressinum

`--| ,-- Retrophyllum_rospigliosii

`----- Podocarpus_archboldii

Tree 18:

,-- Ginkgo_biloba

| ,-- Mataia_podocarpoides

| | ,-- Eathiestrobus_mackenziei

| | | ,-- Cedrus_deodara

| | ,--| | ,-- Keteleeria_davidiana

|--| | | | | ,----- Abies_lasiocarpa

| | | `--| ,--| ,-- Pseudolarix_amabilis

| | | | | `--| ,-- Tsuga_canadensis

| | | | | `----- Nothotsuga_longibracteata

`--| |	`--|	,-- Pseudotsuga_menziesii

| | | ,----- Larix_decidua

| | `--| ,-- Cathaya_argyrophylla

| | `--| ,-- Pinus_contorta

| | `--| ,-- Picea_abies

| | `--| ,-- Schizolepidopsis_ediae

| | `--| ,-- Schizolepidopsis_daohugouensis

| | `----- Schizolepidopsis_canicularis

`--|	,-- Araucaria_mirabilis

| ,----- Araucaria_heterophylla

| ,--| ,-- Wollemia_nobilis

| | `----- Agathis_australis

| | ,-- Lagarostrobos_franklinii

| ,--| ,----- Prumnopitys_taxifolia

| | | ,--| ,-- Lepidothamnus_laxifolius

| | | | `----- Phyllocladus_aspleniifolius

| | `--| ,-- Saxegothaea_conspicua

| | `--| ,-- Microcachrys_tetragona

| | `--| ,-- Pherosphaera_hookeriana

| | `--| ,-- Acmopyle_pancheri

| | `--| ,-- Dacrydium_cupressinum

`--|	`--| ,-- Retrophyllum_rospigliosii

| `----- Podocarpus_archboldii

| ,-- Pararaucaria_patagonica

| | ,-- **New_total_plant_from_Arkansas**

| ,--| ,----- Alvinia_bohemica_plant

| | `--| ,-- Himeriella_muensteri

| | `--| ,-- Kachaikestrobus_acuminatus

| | `----- Tomaxellia_biforme

`--| ,-- Sciadopitys_verticillata

| | ,-- Cunninghamia_lanceolata

| | ,--| ,-- Taiwania_cryptomerioides

`--| | `--| ,-- Athrotaxis_selaginoides

| | `--| ,-- Sequoia_sempervirens

| | `--| ,-- Cryptomeria_japonica

`--|	`--| ,-- Cupressus_funebris

| `----- Callitris_muelleri

| ,-- Telemachus_aequatus

| | ,-- Nothodacrium_warrenii

`--| ,----- Rissikia_media

| | ,-- Voltzia_hexagona

`--| |	,-- Emporia_lockardii

`--| ,----- Hanskerpia_hamiltonensis

`--| ,-- Cordaixylon_dumusum

`----- Taxus_wallichiana

Tree 19:

,-- Ginkgo_biloba

| ,-- Cordaixylon_dumusum

|--| | ,-- Emporia_lockardii

| | ,----- Hanskerpia_hamiltonensis

`--| | ,-- Voltzia_hexagona

| | | ,-- Nothodacrium_warrenii

`--| | ,----- Rissikia_media

| | | ,-- Pararaucaria_patagonica

`--| |	|	,-- **New_total_plant_from_Arkansas**

| | ,--| ,----- Alvinia_bohemica_plant

| | | `--| ,-- Himeriella_muensteri

`--| |	`--| ,-- Kachaikestrobus_acuminatus

| | `----- Tomaxellia_biforme

| | ,-- Telemachus_aequatus

| | | ,-- Eathiestrobus_mackenziei

`--| |	| ,-- Cedrus_deodara

| | ,--| | ,-- Pseudotsuga_menziesii

| | | | | ,----- Larix_decidua

| | | `--| ,--| ,-- Cathaya_argyrophylla

| | | | | `--| ,-- Pinus_contorta

`--| |	| |	`----- Picea_abies

| | `--| ,-- Keteleeria_davidiana

| | | ,----- Abies_lasiocarpa

| | | | ,-- Pseudolarix_amabilis

| | `--| | ,-- Mataia_podocarpoides

| | `--| ,--| ,-- Tsuga_canadensis

| | | | `----- Nothotsuga_longibracteata

`--|	`--| ,-- Schizolepidopsis_ediae

| `--| ,-- Schizolepidopsis_daohugouensis

| `----- Schizolepidopsis_canicularis

| ,-- Sciadopitys_verticillata

| ,--| ,-- Taxus_wallichiana

| | `--| ,-- Cunninghamia_lanceolata

| | `--| ,-- Taiwania_cryptomerioides

| | `--| ,-- Athrotaxis_selaginoides

| | `--| ,-- Sequoia_sempervirens

| | `--| ,-- Cryptomeria_japonica

| | `--| ,-- Cupressus_funebris

`--|	`----- Callitris_muelleri

| ,-- Araucaria_mirabilis

| ,----- Araucaria_heterophylla

| ,--| ,-- Wollemia_nobilis

| | `----- Agathis_australis

| | ,-- Lagarostrobos_franklinii

`--|	,----- Prumnopitys_taxifolia

| ,--| ,-- Lepidothamnus_laxifolius

| | `----- Phyllocladus_aspleniifolius

`--| ,-- Saxegothaea_conspicua

`--| ,-- Microcachrys_tetragona

`--| ,-- Pherosphaera_hookeriana

`--| ,-- Acmopyle_pancheri

`--| ,-- Dacrydium_cupressinum

`--| ,-- Retrophyllum_rospigliosii

`----- Podocarpus_archboldii

Tree 20:

,-- Ginkgo_biloba

| ,-- Cordaixylon_dumusum

|--| | ,-- Emporia_lockardii

| | ,----- Hanskerpia_hamiltonensis

`--| | ,-- Voltzia_hexagona

| | | ,-- Nothodacrium_warrenii

`--| | ,----- Rissikia_media

| | | ,-- Telemachus_aequatus

`--| | |	,-- Schizolepidopsis_ediae

| | | | ,-- Schizolepidopsis_daohugouensis

| | | | ,----- Schizolepidopsis_canicularis

`--| | ,--| | ,-- Cedrus_deodara

| | | | | | ,-- Keteleeria_davidiana

| | | `--| | ,----- Abies_lasiocarpa

| | | | | | ,-- Mataia_podocarpoides

`--| |	`--| ,--| ,----- Pseudolarix_amabilis

| | | | `--| ,-- Tsuga_canadensis

| | | | `----- Nothotsuga_longibracteata

| | `--| ,-- Pseudotsuga_menziesii

| | | ,----- Larix_decidua

| | `--| ,-- Cathaya_argyrophylla

| | `--| ,-- Pinus_contorta

`--|	`--| ,-- Eathiestrobus_mackenziei

| `----- Picea_abies

| ,-- Sciadopitys_verticillata

| ,--| ,-- Taxus_wallichiana

| | `--| ,-- Cunninghamia_lanceolata

| | `--| ,-- Taiwania_cryptomerioides

| | `--| ,-- Athrotaxis_selaginoides

| | `--| ,-- Sequoia_sempervirens

| | `--| ,-- Cryptomeria_japonica

| | `--| ,-- Cupressus_funebris

| | `----- Callitris_muelleri

`--|	,-- Pararaucaria_patagonica

| | ,-- **New_total_plant_from_Arkansas**

| ,--| ,----- Alvinia_bohemica_plant

| | `--| ,-- Himeriella_muensteri

| | `--| ,-- Kachaikestrobus_acuminatus

| | `----- Tomaxellia_biforme

| | ,-- Araucaria_mirabilis

`--|	,----- Araucaria_heterophylla

| ,--| ,-- Wollemia_nobilis

| | `----- Agathis_australis

| | ,-- Lagarostrobos_franklinii

`--|	,----- Prumnopitys_taxifolia

| ,--| ,-- Lepidothamnus_laxifolius

| | `----- Phyllocladus_aspleniifolius

`--| ,-- Saxegothaea_conspicua

`--| ,-- Microcachrys_tetragona

`--| ,-- Pherosphaera_hookeriana

`--| ,-- Acmopyle_pancheri

`--| ,-- Dacrydium_cupressinum

`--| ,-- Retrophyllum_rospigliosii

`----- Podocarpus_archboldii

Tree 21:

,-- Ginkgo_biloba

| ,-- Cordaixylon_dumusum

|--| | ,-- Emporia_lockardii

| | ,----- Hanskerpia_hamiltonensis

`--| | ,-- Voltzia_hexagona

| | | ,-- Nothodacrium_warrenii

`--| | ,----- Rissikia_media

| | | ,-- Telemachus_aequatus

`--| | |	,-- Schizolepidopsis_ediae

| | | | ,-- Schizolepidopsis_daohugouensis

| | | | ,----- Schizolepidopsis_canicularis

`--| | ,--| | ,-- Cedrus_deodara

| | | | | | ,-- Keteleeria_davidiana

| | | `--| | ,----- Abies_lasiocarpa

| | | | | | ,-- Mataia_podocarpoides

`--| |	`--| ,--| ,----- Pseudolarix_amabilis

| | | | `--| ,-- Tsuga_canadensis

| | | | `----- Nothotsuga_longibracteata

| | `--| ,-- Pseudotsuga_menziesii

| | | ,----- Larix_decidua

| | `--| ,-- Cathaya_argyrophylla

| | `--| ,-- Picea_abies

`--|	`--| ,-- Eathiestrobus_mackenziei

| `----- Pinus_contorta

| ,-- Sciadopitys_verticillata

| ,--| ,-- Taxus_wallichiana

| | `--| ,-- Cunninghamia_lanceolata

| | `--| ,-- Taiwania_cryptomerioides

| | `--| ,-- Athrotaxis_selaginoides

| | `--| ,-- Sequoia_sempervirens

| | `--| ,-- Cryptomeria_japonica

| | `--| ,-- Cupressus_funebris

| | `----- Callitris_muelleri

`--|	,-- Pararaucaria_patagonica

| | ,-- **New_total_plant_from_Arkansas**

| ,--| ,----- Alvinia_bohemica_plant

| | `--| ,-- Himeriella_muensteri

| | `--| ,-- Kachaikestrobus_acuminatus

| | `----- Tomaxellia_biforme

| | ,-- Araucaria_mirabilis

`--|	,----- Araucaria_heterophylla

| ,--| ,-- Wollemia_nobilis

| | `----- Agathis_australis

| | ,-- Lagarostrobos_franklinii

`--|	,----- Prumnopitys_taxifolia

| ,--| ,-- Lepidothamnus_laxifolius

| | `----- Phyllocladus_aspleniifolius

`--| ,-- Saxegothaea_conspicua

`--| ,-- Microcachrys_tetragona

`--| ,-- Pherosphaera_hookeriana

`--| ,-- Acmopyle_pancheri

`--| ,-- Dacrydium_cupressinum

`--| ,-- Retrophyllum_rospigliosii

`----- Podocarpus_archboldii

Tree 22:

,-- Ginkgo_biloba

| ,-- Cordaixylon_dumusum

|--| | ,-- Emporia_lockardii

| | ,----- Hanskerpia_hamiltonensis

`--| | ,-- Voltzia_hexagona

| | | ,-- Nothodacrium_warrenii

`--| | ,----- Rissikia_media

| | | ,-- Telemachus_aequatus

`--| | |	,-- Schizolepidopsis_ediae

| | | | ,-- Schizolepidopsis_daohugouensis

| | | ,--| ,----- Schizolepidopsis_canicularis

`--| | | | | ,-- Cedrus_deodara

| | | `--| | ,-- Keteleeria_davidiana

| | | | | ,----- Abies_lasiocarpa

| | | `--| ,--| ,-- Pseudolarix_amabilis

`--| |	| | `--| ,-- Tsuga_canadensis

| | | | `--| ,-- Mataia_podocarpoides

| | `--| `----- Nothotsuga_longibracteata

| | | ,-- Pseudotsuga_menziesii

| | | ,----- Larix_decidua

| | `--| ,-- Cathaya_argyrophylla

| | `--| ,-- Picea_abies

`--|	`--| ,-- Eathiestrobus_mackenziei

| `----- Pinus_contorta

| ,-- Sciadopitys_verticillata

| ,--| ,-- Taxus_wallichiana

| | `--| ,-- Cunninghamia_lanceolata

| | `--| ,-- Taiwania_cryptomerioides

| | `--| ,-- Athrotaxis_selaginoides

| | `--| ,-- Sequoia_sempervirens

| | `--| ,-- Cryptomeria_japonica

| | `--| ,-- Cupressus_funebris

| | `----- Callitris_muelleri

`--|	,-- Pararaucaria_patagonica

| | ,-- **New_total_plant_from_Arkansas**

| ,--| ,----- Alvinia_bohemica_plant

| | `--| ,-- Himeriella_muensteri

| | `--| ,-- Kachaikestrobus_acuminatus

| | `----- Tomaxellia_biforme

| | ,-- Araucaria_mirabilis

`--|	,----- Araucaria_heterophylla

| ,--| ,-- Wollemia_nobilis

| | `----- Agathis_australis

| | ,-- Lagarostrobos_franklinii

`--|	,----- Prumnopitys_taxifolia

| ,--| ,-- Lepidothamnus_laxifolius

| | `----- Phyllocladus_aspleniifolius

`--| ,-- Saxegothaea_conspicua

`--| ,-- Microcachrys_tetragona

`--| ,-- Pherosphaera_hookeriana

`--| ,-- Acmopyle_pancheri

`--| ,-- Dacrydium_cupressinum

`--| ,-- Retrophyllum_rospigliosii

`----- Podocarpus_archboldii

Tree 23:

,-- Ginkgo_biloba

| ,-- Cordaixylon_dumusum

|--| | ,-- Emporia_lockardii

`--| ,----- Hanskerpia_hamiltonensis

| | ,-- Voltzia_hexagona

`--| |	,-- Nothodacrium_warrenii

| | ,----- Rissikia_media

`--| | ,-- Telemachus_aequatus

| | | ,-- Schizolepidopsis_ediae

| | | ,--| ,-- Schizolepidopsis_daohugouensis

`--| |	| `----- Schizolepidopsis_canicularis

| | | ,-- Cedrus_deodara

| | ,--| | ,-- Mataia_podocarpoides

| | | | | | ,-- Keteleeria_davidiana

`--| | `--| ,--| ,----- Abies_lasiocarpa

| | | | `--| ,-- Pseudolarix_amabilis

| | | | `--| ,-- Tsuga_canadensis

| | `--| `----- Nothotsuga_longibracteata

| | | ,-- Pseudotsuga_menziesii

| | | ,----- Larix_decidua

| | `--| ,-- Cathaya_argyrophylla

`--|	`--| ,-- Pinus_contorta

| `--| ,-- Eathiestrobus_mackenziei

| `----- Picea_abies

| ,-- **New_total_plant_from_Arkansas**

| ,----- Alvinia_bohemica_plant

| ,--| ,-- Himeriella_muensteri

| | `--| ,-- Kachaikestrobus_acuminatus

| | `----- Tomaxellia_biforme

| | ,-- Pararaucaria_patagonica

`--| |	,-- Sciadopitys_verticillata

| | ,--| ,-- Taxus_wallichiana

| | | `--| ,-- Cunninghamia_lanceolata

| | | `--| ,-- Taiwania_cryptomerioides

`--| |	`--| ,-- Athrotaxis_selaginoides

| | `--| ,-- Sequoia_sempervirens

| | `--| ,-- Cryptomeria_japonica

| | `--| ,-- Cupressus_funebris

`--|	`----- Callitris_muelleri

| ,-- Araucaria_mirabilis

| ,----- Araucaria_heterophylla

| ,--| ,-- Wollemia_nobilis

| | `----- Agathis_australis

| | ,-- Lagarostrobos_franklinii

`--|	,----- Prumnopitys_taxifolia

| ,--| ,-- Lepidothamnus_laxifolius

| | `----- Phyllocladus_aspleniifolius

`--| ,-- Saxegothaea_conspicua

`--| ,-- Microcachrys_tetragona

`--| ,-- Pherosphaera_hookeriana

`--| ,-- Acmopyle_pancheri

`--| ,-- Dacrydium_cupressinum

`--| ,-- Retrophyllum_rospigliosii

`----- Podocarpus_archboldii

Tree 24:

,-- Ginkgo_biloba

| ,-- Cordaixylon_dumusum

|--| | ,-- Emporia_lockardii

| | ,----- Hanskerpia_hamiltonensis

`--| | ,-- Voltzia_hexagona

| | | ,-- Nothodacrium_warrenii

`--| | ,----- Rissikia_media

| | | ,-- Pararaucaria_patagonica

`--| |	,--| ,-- **New_total_plant_from_Arkansas**

| | | `----- Alvinia_bohemica_plant

| | ,--| ,-- Himeriella_muensteri

`--| | `--| ,-- Kachaikestrobus_acuminatus

| | `----- Tomaxellia_biforme

| | ,-- Telemachus_aequatus

| | | ,-- Eathiestrobus_mackenziei

`--| |	| ,-- Cedrus_deodara

| | ,--| | ,-- Mataia_podocarpoides

| | | `--| | ,-- Keteleeria_davidiana

| | | | | ,----- Abies_lasiocarpa

| | | `--| ,--| ,-- Pseudolarix_amabilis

`--| |	| | `--| ,-- Tsuga_canadensis

| | | | `----- Nothotsuga_longibracteata

| | `--| ,-- Pseudotsuga_menziesii

| | | ,----- Larix_decidua

| | `--| ,-- Cathaya_argyrophylla

| | `--| ,-- Pinus_contorta

| | `--| ,-- Picea_abies

`--|	`--| ,-- Schizolepidopsis_ediae

| `--| ,-- Schizolepidopsis_daohugouensis

| `----- Schizolepidopsis_canicularis

| ,-- Sciadopitys_verticillata

| ,--| ,-- Taxus_wallichiana

| | `--| ,-- Cunninghamia_lanceolata

| | `--| ,-- Taiwania_cryptomerioides

| | `--| ,-- Athrotaxis_selaginoides

| | `--| ,-- Sequoia_sempervirens

| | `--| ,-- Cryptomeria_japonica

| | `--| ,-- Cupressus_funebris

`--|	`----- Callitris_muelleri

| ,-- Araucaria_mirabilis

| ,----- Araucaria_heterophylla

| ,--| ,-- Wollemia_nobilis

| | `----- Agathis_australis

| | ,-- Lagarostrobos_franklinii

`--|	,----- Prumnopitys_taxifolia

| ,--| ,-- Lepidothamnus_laxifolius

| | `----- Phyllocladus_aspleniifolius

`--| ,-- Saxegothaea_conspicua

`--| ,-- Microcachrys_tetragona

`--| ,-- Pherosphaera_hookeriana

`--| ,-- Acmopyle_pancheri

`--| ,-- Dacrydium_cupressinum

`--| ,-- Retrophyllum_rospigliosii

`----- Podocarpus_archboldii

Tree 25:

,-- Ginkgo_biloba

| ,-- Cordaixylon_dumusum

|--| | ,-- Emporia_lockardii

| | ,----- Hanskerpia_hamiltonensis

`--| | ,-- Voltzia_hexagona

| | | ,-- Nothodacrium_warrenii

`--| | ,----- Rissikia_media

| | | ,-- **New_total_plant_from_Arkansas**

`--| |	,--| ,-- Alvinia_bohemica_plant

| | | `----- Pararaucaria_patagonica

| | ,--| ,-- Himeriella_muensteri

`--| | `--| ,-- Kachaikestrobus_acuminatus

| | `----- Tomaxellia_biforme

| | ,-- Telemachus_aequatus

| | | ,-- Eathiestrobus_mackenziei

`--| |	| ,-- Cedrus_deodara

| | ,--| | ,-- Pseudotsuga_menziesii

| | | | | ,----- Larix_decidua

| | | `--| ,--| ,-- Cathaya_argyrophylla

| | | | | `--| ,-- Pinus_contorta

`--| |	| |	`----- Picea_abies

| | `--| ,-- Keteleeria_davidiana

| | | ,----- Abies_lasiocarpa

| | | | ,-- Pseudolarix_amabilis

| | `--| | ,-- Mataia_podocarpoides

| | `--| ,--| ,-- Tsuga_canadensis

| | | | `----- Nothotsuga_longibracteata

`--|	`--| ,-- Schizolepidopsis_ediae

| `--| ,-- Schizolepidopsis_daohugouensis

| `----- Schizolepidopsis_canicularis

| ,-- Sciadopitys_verticillata

| ,--| ,-- Taxus_wallichiana

| | `--| ,-- Cunninghamia_lanceolata

| | `--| ,-- Taiwania_cryptomerioides

| | `--| ,-- Athrotaxis_selaginoides

| | `--| ,-- Sequoia_sempervirens

| | `--| ,-- Cryptomeria_japonica

| | `--| ,-- Cupressus_funebris

`--|	`----- Callitris_muelleri

| ,-- Araucaria_mirabilis

| ,----- Araucaria_heterophylla

| ,--| ,-- Wollemia_nobilis

| | `----- Agathis_australis

| | ,-- Lagarostrobos_franklinii

`--|	,----- Prumnopitys_taxifolia

| ,--| ,-- Lepidothamnus_laxifolius

| | `----- Phyllocladus_aspleniifolius

`--| ,-- Saxegothaea_conspicua

`--| ,-- Microcachrys_tetragona

`--| ,-- Pherosphaera_hookeriana

`--| ,-- Acmopyle_pancheri

`--| ,-- Dacrydium_cupressinum

`--| ,-- Retrophyllum_rospigliosii

`----- Podocarpus_archboldii

Tree 26:

,-- Ginkgo_biloba

| ,-- Cedrus_deodara

| | ,-- Pseudotsuga_menziesii

| | ,----- Larix_decidua

| | ,--| ,-- Cathaya_argyrophylla

| ,--| | `--| ,-- Pinus_contorta

| | | | `--| ,-- Eathiestrobus_mackenziei

|--| | | | `----- Picea_abies

| | `--| ,-- Keteleeria_davidiana

| | | ,----- Abies_lasiocarpa

| | | | ,-- Pseudolarix_amabilis

| | `--| | ,-- Tsuga_canadensis

| | `--| ,----- Nothotsuga_longibracteata

| | `--| ,-- Schizolepidopsis_ediae

`--|	`--| ,-- Schizolepidopsis_daohugouensis

| `----- Schizolepidopsis_canicularis

| ,-- Mataia_podocarpoides

| | ,-- Araucaria_mirabilis

| | ,----- Araucaria_heterophylla

| | ,--| ,-- Wollemia_nobilis

| | | `----- Agathis_australis

| | | ,-- Lagarostrobos_franklinii

| | ,--| ,----- Prumnopitys_taxifolia

`--| | | ,--| ,-- Lepidothamnus_laxifolius

| | | | `----- Phyllocladus_aspleniifolius

| | `--| ,-- Saxegothaea_conspicua

| | `--| ,-- Microcachrys_tetragona

| | `--| ,-- Pherosphaera_hookeriana

| | `--| ,-- Acmopyle_pancheri

| | `--| ,-- Dacrydium_cupressinum

`--|	`--| ,-- Retrophyllum_rospigliosii

| `----- Podocarpus_archboldii

| ,-- Pararaucaria_patagonica

| | ,-- **New_total_plant_from_Arkansas**

| ,--| ,----- Alvinia_bohemica_plant

| | `--| ,-- Himeriella_muensteri

| | `--| ,-- Kachaikestrobus_acuminatus

| | `----- Tomaxellia_biforme

`--| ,-- Sciadopitys_verticillata

| | ,-- Cunninghamia_lanceolata

| | ,--| ,-- Taiwania_cryptomerioides

`--| | `--| ,-- Athrotaxis_selaginoides

| | `--| ,-- Sequoia_sempervirens

| | `--| ,-- Cryptomeria_japonica

`--|	`--| ,-- Cupressus_funebris

| `----- Callitris_muelleri

| ,-- Telemachus_aequatus

| | ,-- Nothodacrium_warrenii

`--| ,----- Rissikia_media

| | ,-- Voltzia_hexagona

`--| |	,-- Emporia_lockardii

`--| ,----- Hanskerpia_hamiltonensis

`--| ,-- Cordaixylon_dumusum

`----- Taxus_wallichiana

Tree 27:

,-- Ginkgo_biloba

| ,-- Cordaixylon_dumusum

|--| | ,-- Emporia_lockardii

| | ,----- Hanskerpia_hamiltonensis

`--| | ,-- Voltzia_hexagona

| | | ,-- Nothodacrium_warrenii

`--| | ,----- Rissikia_media

| | | ,-- Telemachus_aequatus

`--| | |	,-- Schizolepidopsis_ediae

| | | ,--| ,-- Schizolepidopsis_daohugouensis

| | | | `----- Schizolepidopsis_canicularis

`--| |	| ,-- Cedrus_deodara

| | ,--| | ,-- Keteleeria_davidiana

| | | | | ,--| ,-- Mataia_podocarpoides

| | | | | | `----- Abies_lasiocarpa

`--| | `--| ,--| ,-- Pseudolarix_amabilis

| | | | `--| ,-- Tsuga_canadensis

| | | | `----- Nothotsuga_longibracteata

| | `--| ,-- Pseudotsuga_menziesii

| | | ,----- Larix_decidua

| | `--| ,-- Cathaya_argyrophylla

| | `--| ,-- Picea_abies

| | `--| ,-- Eathiestrobus_mackenziei

`--|	`----- Pinus_contorta

| ,-- Sciadopitys_verticillata

| ,--| ,-- Taxus_wallichiana

| | `--| ,-- Cunninghamia_lanceolata

| | `--| ,-- Taiwania_cryptomerioides

| | `--| ,-- Athrotaxis_selaginoides

| | `--| ,-- Sequoia_sempervirens

| | `--| ,-- Cryptomeria_japonica

| | `--| ,-- Cupressus_funebris

| | `----- Callitris_muelleri

`--|	,-- Pararaucaria_patagonica

| | ,-- **New_total_plant_from_Arkansas**

| ,--| ,----- Alvinia_bohemica_plant

| | `--| ,-- Himeriella_muensteri

| | `--| ,-- Kachaikestrobus_acuminatus

| | `----- Tomaxellia_biforme

| | ,-- Araucaria_mirabilis

`--|	,----- Araucaria_heterophylla

| ,--| ,-- Wollemia_nobilis

| | `----- Agathis_australis

| | ,-- Lagarostrobos_franklinii

`--|	,----- Prumnopitys_taxifolia

| ,--| ,-- Lepidothamnus_laxifolius

| | `----- Phyllocladus_aspleniifolius

`--| ,-- Saxegothaea_conspicua

`--| ,-- Microcachrys_tetragona

`--| ,-- Pherosphaera_hookeriana

`--| ,-- Acmopyle_pancheri

`--| ,-- Dacrydium_cupressinum

`--| ,-- Retrophyllum_rospigliosii

`----- Podocarpus_archboldii

Tree 28:

,-- Ginkgo_biloba

| ,-- Cordaixylon_dumusum

|--| | ,-- Emporia_lockardii

| | ,----- Hanskerpia_hamiltonensis

`--| | ,-- Voltzia_hexagona

| | | ,-- Nothodacrium_warrenii

`--| | ,----- Rissikia_media

| | | ,-- Telemachus_aequatus

`--| | |	,-- Schizolepidopsis_ediae

| | | ,--| ,-- Schizolepidopsis_daohugouensis

| | | | `----- Schizolepidopsis_canicularis

`--| |	| ,-- Cedrus_deodara

| | ,--| | ,-- Keteleeria_davidiana

| | | | | ,----- Abies_lasiocarpa

| | | `--| ,--| ,-- Mataia_podocarpoides

`--| |	| | `--| ,-- Pseudolarix_amabilis

| | | | `--| ,-- Tsuga_canadensis

| | `--| `----- Nothotsuga_longibracteata

| | | ,-- Pseudotsuga_menziesii

| | | ,----- Larix_decidua

| | `--| ,-- Cathaya_argyrophylla

| | `--| ,-- Picea_abies

| | `--| ,-- Eathiestrobus_mackenziei

`--|	`----- Pinus_contorta

| ,-- Sciadopitys_verticillata

| ,--| ,-- Taxus_wallichiana

| | `--| ,-- Cunninghamia_lanceolata

| | `--| ,-- Taiwania_cryptomerioides

| | `--| ,-- Athrotaxis_selaginoides

| | `--| ,-- Sequoia_sempervirens

| | `--| ,-- Cryptomeria_japonica

| | `--| ,-- Cupressus_funebris

| | `----- Callitris_muelleri

`--|	,-- Pararaucaria_patagonica

| | ,-- **New_total_plant_from_Arkansas**

| ,--| ,----- Alvinia_bohemica_plant

| | `--| ,-- Himeriella_muensteri

| | `--| ,-- Kachaikestrobus_acuminatus

| | `----- Tomaxellia_biforme

| | ,-- Araucaria_mirabilis

`--|	,----- Araucaria_heterophylla

| ,--| ,-- Wollemia_nobilis

| | `----- Agathis_australis

| | ,-- Lagarostrobos_franklinii

`--|	,----- Prumnopitys_taxifolia

| ,--| ,-- Lepidothamnus_laxifolius

| | `----- Phyllocladus_aspleniifolius

`--| ,-- Saxegothaea_conspicua

`--| ,-- Microcachrys_tetragona

`--| ,-- Pherosphaera_hookeriana

`--| ,-- Acmopyle_pancheri

`--| ,-- Dacrydium_cupressinum

`--| ,-- Retrophyllum_rospigliosii

`----- Podocarpus_archboldii

Tree 29:

,-- Ginkgo_biloba

| ,-- Cordaixylon_dumusum

|--| | ,-- Emporia_lockardii

| | ,----- Hanskerpia_hamiltonensis

`--| | ,-- Voltzia_hexagona

| | | ,-- Nothodacrium_warrenii

`--| | ,----- Rissikia_media

| | | ,-- Telemachus_aequatus

`--| | |	,-- Schizolepidopsis_ediae

| | | ,--| ,-- Schizolepidopsis_daohugouensis

| | | | `----- Schizolepidopsis_canicularis

`--| |	| ,-- Cedrus_deodara

| | ,--| | ,-- Mataia_podocarpoides

| | | | | ,--| ,-- Keteleeria_davidiana

| | | | | | `----- Abies_lasiocarpa

`--| | `--| ,--| ,-- Pseudolarix_amabilis

| | | | `--| ,-- Tsuga_canadensis

| | | | `----- Nothotsuga_longibracteata

| | `--| ,-- Pseudotsuga_menziesii

| | | ,----- Larix_decidua

| | `--| ,-- Cathaya_argyrophylla

| | `--| ,-- Picea_abies

| | `--| ,-- Eathiestrobus_mackenziei

`--|	`----- Pinus_contorta

| ,-- Sciadopitys_verticillata

| ,--| ,-- Taxus_wallichiana

| | `--| ,-- Cunninghamia_lanceolata

| | `--| ,-- Taiwania_cryptomerioides

| | `--| ,-- Athrotaxis_selaginoides

| | `--| ,-- Sequoia_sempervirens

| | `--| ,-- Cryptomeria_japonica

| | `--| ,-- Cupressus_funebris

| | `----- Callitris_muelleri

`--|	,-- Pararaucaria_patagonica

| | ,-- **New_total_plant_from_Arkansas**

| ,--| ,----- Alvinia_bohemica_plant

| | `--| ,-- Himeriella_muensteri

| | `--| ,-- Kachaikestrobus_acuminatus

| | `----- Tomaxellia_biforme

| | ,-- Araucaria_mirabilis

`--|	,----- Araucaria_heterophylla

| ,--| ,-- Wollemia_nobilis

| | `----- Agathis_australis

| | ,-- Lagarostrobos_franklinii

`--|	,----- Prumnopitys_taxifolia

| ,--| ,-- Lepidothamnus_laxifolius

| | `----- Phyllocladus_aspleniifolius

`--| ,-- Saxegothaea_conspicua

`--| ,-- Microcachrys_tetragona

`--| ,-- Pherosphaera_hookeriana

`--| ,-- Acmopyle_pancheri

`--| ,-- Dacrydium_cupressinum

`--| ,-- Retrophyllum_rospigliosii

`----- Podocarpus_archboldii

Tree 30:

,-- Ginkgo_biloba

| ,-- Cordaixylon_dumusum

|--| | ,-- Emporia_lockardii

| | ,----- Hanskerpia_hamiltonensis

`--| | ,-- Voltzia_hexagona

| | | ,-- Nothodacrium_warrenii

`--| | ,----- Rissikia_media

| | | ,-- Alvinia_bohemica_plant

`--| |	,--| ,-- **New_total_plant_from_Arkansas**

| | | `----- Pararaucaria_patagonica

| | ,--| ,-- Himeriella_muensteri

`--| | `--| ,-- Kachaikestrobus_acuminatus

| | `----- Tomaxellia_biforme

| | ,-- Telemachus_aequatus

| | | ,-- Eathiestrobus_mackenziei

| | | | ,-- Cedrus_deodara

`--| |	| |	,-- Mataia_podocarpoides

| | ,--| | ,--| ,-- Keteleeria_davidiana

| | | | | | `----- Abies_lasiocarpa

| | | `--| ,--| ,-- Pseudolarix_amabilis

| | | | | `--| ,-- Tsuga_canadensis

`--| |	| |	`----- Nothotsuga_longibracteata

| | `--| ,-- Pseudotsuga_menziesii

| | | ,----- Larix_decidua

| | `--| ,-- Cathaya_argyrophylla

| | `--| ,-- Pinus_contorta

| | `--| ,-- Picea_abies

| | `--| ,-- Schizolepidopsis_ediae

`--|	`--| ,-- Schizolepidopsis_daohugouensis

| `----- Schizolepidopsis_canicularis

| ,-- Sciadopitys_verticillata

| ,--| ,-- Taxus_wallichiana

| | `--| ,-- Cunninghamia_lanceolata

| | `--| ,-- Taiwania_cryptomerioides

| | `--| ,-- Athrotaxis_selaginoides

| | `--| ,-- Sequoia_sempervirens

| | `--| ,-- Cryptomeria_japonica

| | `--| ,-- Cupressus_funebris

`--|	`----- Callitris_muelleri

| ,-- Araucaria_mirabilis

| ,----- Araucaria_heterophylla

| ,--| ,-- Wollemia_nobilis

| | `----- Agathis_australis

| | ,-- Lagarostrobos_franklinii

`--|	,----- Prumnopitys_taxifolia

| ,--| ,-- Lepidothamnus_laxifolius

| | `----- Phyllocladus_aspleniifolius

`--| ,-- Saxegothaea_conspicua

`--| ,-- Microcachrys_tetragona

`--| ,-- Pherosphaera_hookeriana

`--| ,-- Acmopyle_pancheri

`--| ,-- Dacrydium_cupressinum

`--| ,-- Retrophyllum_rospigliosii

`----- Podocarpus_archboldii

Tree 31:

,-- Ginkgo_biloba

| ,-- Cordaixylon_dumusum

|--| | ,-- Emporia_lockardii

| | ,----- Hanskerpia_hamiltonensis

`--| | ,-- Voltzia_hexagona

| | | ,-- Nothodacrium_warrenii

`--| | ,----- Rissikia_media

| | | ,-- Alvinia_bohemica_plant

`--| |	,--| ,-- **New_total_plant_from_Arkansas**

| | | `----- Pararaucaria_patagonica

| | ,--| ,-- Himeriella_muensteri

`--| | `--| ,-- Kachaikestrobus_acuminatus

| | `----- Tomaxellia_biforme

| | ,-- Telemachus_aequatus

| | | ,-- Eathiestrobus_mackenziei

`--| |	| ,-- Cedrus_deodara

| | ,--| | ,-- Keteleeria_davidiana

| | | | | ,----- Abies_lasiocarpa

| | | `--| ,--| ,-- Mataia_podocarpoides

| | | | | `--| ,-- Pseudolarix_amabilis

`--| |	| |	`--| ,-- Tsuga_canadensis

| | `--| `----- Nothotsuga_longibracteata

| | | ,-- Pseudotsuga_menziesii

| | | ,----- Larix_decidua

| | `--| ,-- Cathaya_argyrophylla

| | `--| ,-- Pinus_contorta

| | `--| ,-- Picea_abies

`--|	`--| ,-- Schizolepidopsis_ediae

| `--| ,-- Schizolepidopsis_daohugouensis

| `----- Schizolepidopsis_canicularis

| ,-- Sciadopitys_verticillata

| ,--| ,-- Taxus_wallichiana

| | `--| ,-- Cunninghamia_lanceolata

| | `--| ,-- Taiwania_cryptomerioides

| | `--| ,-- Athrotaxis_selaginoides

| | `--| ,-- Sequoia_sempervirens

| | `--| ,-- Cryptomeria_japonica

| | `--| ,-- Cupressus_funebris

`--|	`----- Callitris_muelleri

| ,-- Araucaria_mirabilis

| ,----- Araucaria_heterophylla

| ,--| ,-- Wollemia_nobilis

| | `----- Agathis_australis

| | ,-- Lagarostrobos_franklinii

`--|	,----- Prumnopitys_taxifolia

| ,--| ,-- Lepidothamnus_laxifolius

| | `----- Phyllocladus_aspleniifolius

`--| ,-- Saxegothaea_conspicua

`--| ,-- Microcachrys_tetragona

`--| ,-- Pherosphaera_hookeriana

`--| ,-- Acmopyle_pancheri

`--| ,-- Dacrydium_cupressinum

`--| ,-- Retrophyllum_rospigliosii

`----- Podocarpus_archboldii

Tree 32:

,-- Ginkgo_biloba

| ,-- Cordaixylon_dumusum

|--| | ,-- Emporia_lockardii

| | ,----- Hanskerpia_hamiltonensis

`--| | ,-- Voltzia_hexagona

| | | ,-- Nothodacrium_warrenii

`--| | ,----- Rissikia_media

| | | ,-- Alvinia_bohemica_plant

`--| |	,--| ,-- **New_total_plant_from_Arkansas**

| | | `----- Pararaucaria_patagonica

| | ,--| ,-- Himeriella_muensteri

`--| | `--| ,-- Kachaikestrobus_acuminatus

| | `----- Tomaxellia_biforme

| | ,-- Telemachus_aequatus

| | | ,-- Eathiestrobus_mackenziei

| | | | ,-- Cedrus_deodara

`--| |	| |	,-- Keteleeria_davidiana

| | ,--| | ,--| ,-- Mataia_podocarpoides

| | | | | | `----- Abies_lasiocarpa

| | | `--| ,--| ,-- Pseudolarix_amabilis

| | | | | `--| ,-- Tsuga_canadensis

`--| |	| |	`----- Nothotsuga_longibracteata

| | `--| ,-- Pseudotsuga_menziesii

| | | ,----- Larix_decidua

| | `--| ,-- Cathaya_argyrophylla

| | `--| ,-- Pinus_contorta

| | `--| ,-- Picea_abies

| | `--| ,-- Schizolepidopsis_ediae

`--|	`--| ,-- Schizolepidopsis_daohugouensis

| `----- Schizolepidopsis_canicularis

| ,-- Sciadopitys_verticillata

| ,--| ,-- Taxus_wallichiana

| | `--| ,-- Cunninghamia_lanceolata

| | `--| ,-- Taiwania_cryptomerioides

| | `--| ,-- Athrotaxis_selaginoides

| | `--| ,-- Sequoia_sempervirens

| | `--| ,-- Cryptomeria_japonica

| | `--| ,-- Cupressus_funebris

`--|	`----- Callitris_muelleri

| ,-- Araucaria_mirabilis

| ,----- Araucaria_heterophylla

| ,--| ,-- Wollemia_nobilis

| | `----- Agathis_australis

| | ,-- Lagarostrobos_franklinii

`--|	,----- Prumnopitys_taxifolia

| ,--| ,-- Lepidothamnus_laxifolius

| | `----- Phyllocladus_aspleniifolius

`--| ,-- Saxegothaea_conspicua

`--| ,-- Microcachrys_tetragona

`--| ,-- Pherosphaera_hookeriana

`--| ,-- Acmopyle_pancheri

`--| ,-- Dacrydium_cupressinum

`--| ,-- Retrophyllum_rospigliosii

`----- Podocarpus_archboldii

Tree 33:

,-- Ginkgo_biloba

| ,-- Cordaixylon_dumusum

|--| | ,-- Emporia_lockardii

| | ,----- Hanskerpia_hamiltonensis

`--| | ,-- Voltzia_hexagona

| | | ,-- Nothodacrium_warrenii

`--| | ,----- Rissikia_media

| | | ,-- Alvinia_bohemica_plant

`--| |	,--| ,-- **New_total_plant_from_Arkansas**

| | | `----- Pararaucaria_patagonica

| | ,--| ,-- Himeriella_muensteri

`--| | `--| ,-- Kachaikestrobus_acuminatus

| | `----- Tomaxellia_biforme

| | ,-- Telemachus_aequatus

| | | ,-- Eathiestrobus_mackenziei

| | | | ,-- Cedrus_deodara

`--| |	| |	,-- Keteleeria_davidiana

| | ,--| | ,----- Abies_lasiocarpa

| | | | | | ,-- Mataia_podocarpoides

| | | `--| ,--| ,----- Pseudolarix_amabilis

| | | | | `--| ,-- Tsuga_canadensis

`--| |	| |	`----- Nothotsuga_longibracteata

| | `--| ,-- Pseudotsuga_menziesii

| | | ,----- Larix_decidua

| | `--| ,-- Cathaya_argyrophylla

| | `--| ,-- Pinus_contorta

| | `--| ,-- Picea_abies

| | `--| ,-- Schizolepidopsis_ediae

`--|	`--| ,-- Schizolepidopsis_daohugouensis

| `----- Schizolepidopsis_canicularis

| ,-- Sciadopitys_verticillata

| ,--| ,-- Taxus_wallichiana

| | `--| ,-- Cunninghamia_lanceolata

| | `--| ,-- Taiwania_cryptomerioides

| | `--| ,-- Athrotaxis_selaginoides

| | `--| ,-- Sequoia_sempervirens

| | `--| ,-- Cryptomeria_japonica

| | `--| ,-- Cupressus_funebris

`--|	`----- Callitris_muelleri

| ,-- Araucaria_mirabilis

| ,----- Araucaria_heterophylla

| ,--| ,-- Wollemia_nobilis

| | `----- Agathis_australis

| | ,-- Lagarostrobos_franklinii

`--|	,----- Prumnopitys_taxifolia

| ,--| ,-- Lepidothamnus_laxifolius

| | `----- Phyllocladus_aspleniifolius

`--| ,-- Saxegothaea_conspicua

`--| ,-- Microcachrys_tetragona

`--| ,-- Pherosphaera_hookeriana

`--| ,-- Acmopyle_pancheri

`--| ,-- Dacrydium_cupressinum

`--| ,-- Retrophyllum_rospigliosii

`----- Podocarpus_archboldii

Tree 34:

,-- Ginkgo_biloba

| ,-- Cordaixylon_dumusum

|--| | ,-- Emporia_lockardii

| | ,----- Hanskerpia_hamiltonensis

`--| | ,-- Voltzia_hexagona

| | | ,-- Nothodacrium_warrenii

`--| | ,----- Rissikia_media

| | | ,-- Alvinia_bohemica_plant

`--| |	,--| ,-- **New_total_plant_from_Arkansas**

| | | `----- Pararaucaria_patagonica

| | ,--| ,-- Himeriella_muensteri

`--| | `--| ,-- Kachaikestrobus_acuminatus

| | `----- Tomaxellia_biforme

| | ,-- Telemachus_aequatus

| | | ,-- Eathiestrobus_mackenziei

`--| |	| ,-- Cedrus_deodara

| | ,--| | ,-- Keteleeria_davidiana

| | | | | ,----- Abies_lasiocarpa

| | | `--| ,--| ,-- Pseudolarix_amabilis

| | | | | `--| ,-- Mataia_podocarpoides

`--| |	| |	`--| ,-- Tsuga_canadensis

| | `--| `----- Nothotsuga_longibracteata

| | | ,-- Pseudotsuga_menziesii

| | | ,----- Larix_decidua

| | `--| ,-- Cathaya_argyrophylla

| | `--| ,-- Pinus_contorta

| | `--| ,-- Picea_abies

`--|	`--| ,-- Schizolepidopsis_ediae

| `--| ,-- Schizolepidopsis_daohugouensis

| `----- Schizolepidopsis_canicularis

| ,-- Sciadopitys_verticillata

| ,--| ,-- Taxus_wallichiana

| | `--| ,-- Cunninghamia_lanceolata

| | `--| ,-- Taiwania_cryptomerioides

| | `--| ,-- Athrotaxis_selaginoides

| | `--| ,-- Sequoia_sempervirens

| | `--| ,-- Cryptomeria_japonica

| | `--| ,-- Cupressus_funebris

`--|	`----- Callitris_muelleri

| ,-- Araucaria_mirabilis

| ,----- Araucaria_heterophylla

| ,--| ,-- Wollemia_nobilis

| | `----- Agathis_australis

| | ,-- Lagarostrobos_franklinii

`--|	,----- Prumnopitys_taxifolia

| ,--| ,-- Lepidothamnus_laxifolius

| | `----- Phyllocladus_aspleniifolius

`--| ,-- Saxegothaea_conspicua

`--| ,-- Microcachrys_tetragona

`--| ,-- Pherosphaera_hookeriana

`--| ,-- Acmopyle_pancheri

`--| ,-- Dacrydium_cupressinum

`--| ,-- Retrophyllum_rospigliosii

`----- Podocarpus_archboldii

Tree 35:

,-- Ginkgo_biloba

| ,-- Cordaixylon_dumusum

|--| | ,-- Emporia_lockardii

| | ,----- Hanskerpia_hamiltonensis

`--| | ,-- Voltzia_hexagona

| | | ,-- Nothodacrium_warrenii

`--| | ,----- Rissikia_media

| | | ,-- Alvinia_bohemica_plant

`--| |	,--| ,-- **New_total_plant_from_Arkansas**

| | | `----- Pararaucaria_patagonica

| | ,--| ,-- Himeriella_muensteri

`--| | `--| ,-- Kachaikestrobus_acuminatus

| | `----- Tomaxellia_biforme

| | ,-- Telemachus_aequatus

| | | ,-- Eathiestrobus_mackenziei

`--| |	| ,-- Cedrus_deodara

| | ,--| | ,-- Keteleeria_davidiana

| | | | | ,----- Abies_lasiocarpa

| | | `--| ,--| ,-- Pseudolarix_amabilis

| | | | | `--| ,-- Tsuga_canadensis

`--| |	| |	`--| ,-- Mataia_podocarpoides

| | `--| `----- Nothotsuga_longibracteata

| | | ,-- Pseudotsuga_menziesii

| | | ,----- Larix_decidua

| | `--| ,-- Cathaya_argyrophylla

| | `--| ,-- Pinus_contorta

| | `--| ,-- Picea_abies

`--|	`--| ,-- Schizolepidopsis_ediae

| `--| ,-- Schizolepidopsis_daohugouensis

| `----- Schizolepidopsis_canicularis

| ,-- Sciadopitys_verticillata

| ,--| ,-- Taxus_wallichiana

| | `--| ,-- Cunninghamia_lanceolata

| | `--| ,-- Taiwania_cryptomerioides

| | `--| ,-- Athrotaxis_selaginoides

| | `--| ,-- Sequoia_sempervirens

| | `--| ,-- Cryptomeria_japonica

| | `--| ,-- Cupressus_funebris

`--|	`----- Callitris_muelleri

| ,-- Araucaria_mirabilis

| ,----- Araucaria_heterophylla

| ,--| ,-- Wollemia_nobilis

| | `----- Agathis_australis

| | ,-- Lagarostrobos_franklinii

`--|	,----- Prumnopitys_taxifolia

| ,--| ,-- Lepidothamnus_laxifolius

| | `----- Phyllocladus_aspleniifolius

`--| ,-- Saxegothaea_conspicua

`--| ,-- Microcachrys_tetragona

`--| ,-- Pherosphaera_hookeriana

`--| ,-- Acmopyle_pancheri

`--| ,-- Dacrydium_cupressinum

`--| ,-- Retrophyllum_rospigliosii

`----- Podocarpus_archboldii

Tree 36:

,-- Ginkgo_biloba

| ,-- Cordaixylon_dumusum

|--| | ,-- Emporia_lockardii

| | ,----- Hanskerpia_hamiltonensis

`--| | ,-- Voltzia_hexagona

| | | ,-- Nothodacrium_warrenii

`--| | ,----- Rissikia_media

| | | ,-- Alvinia_bohemica_plant

`--| |	,--| ,-- **New_total_plant_from_Arkansas**

| | | `----- Pararaucaria_patagonica

| | ,--| ,-- Himeriella_muensteri

`--| | `--| ,-- Kachaikestrobus_acuminatus

| | `----- Tomaxellia_biforme

| | ,-- Telemachus_aequatus

| | | ,-- Eathiestrobus_mackenziei

`--| |	| ,-- Cedrus_deodara

| | ,--| | ,-- Mataia_podocarpoides

| | | | | | ,-- Keteleeria_davidiana

| | | `--| ,--| ,----- Abies_lasiocarpa

| | | | | `--| ,-- Pseudolarix_amabilis

`--| |	| |	`--| ,-- Tsuga_canadensis

| | `--| `----- Nothotsuga_longibracteata

| | | ,-- Pseudotsuga_menziesii

| | | ,----- Larix_decidua

| | `--| ,-- Cathaya_argyrophylla

| | `--| ,-- Pinus_contorta

| | `--| ,-- Picea_abies

`--|	`--| ,-- Schizolepidopsis_ediae

| `--| ,-- Schizolepidopsis_daohugouensis

| `----- Schizolepidopsis_canicularis

| ,-- Sciadopitys_verticillata

| ,--| ,-- Taxus_wallichiana

| | `--| ,-- Cunninghamia_lanceolata

| | `--| ,-- Taiwania_cryptomerioides

| | `--| ,-- Athrotaxis_selaginoides

| | `--| ,-- Sequoia_sempervirens

| | `--| ,-- Cryptomeria_japonica

| | `--| ,-- Cupressus_funebris

`--|	`----- Callitris_muelleri

| ,-- Araucaria_mirabilis

| ,----- Araucaria_heterophylla

| ,--| ,-- Wollemia_nobilis

| | `----- Agathis_australis

| | ,-- Lagarostrobos_franklinii

`--|	,----- Prumnopitys_taxifolia

| ,--| ,-- Lepidothamnus_laxifolius

| | `----- Phyllocladus_aspleniifolius

`--| ,-- Saxegothaea_conspicua

`--| ,-- Microcachrys_tetragona

`--| ,-- Pherosphaera_hookeriana

`--| ,-- Acmopyle_pancheri

`--| ,-- Dacrydium_cupressinum

`--| ,-- Retrophyllum_rospigliosii

`----- Podocarpus_archboldii

Tree 37:

,-- Ginkgo_biloba

| ,-- Cordaixylon_dumusum

|--| | ,-- Emporia_lockardii

| | ,----- Hanskerpia_hamiltonensis

`--| | ,-- Voltzia_hexagona

| | | ,-- Nothodacrium_warrenii

`--| | ,----- Rissikia_media

| | | ,-- Pararaucaria_patagonica

`--| |	|	,-- **New_total_plant_from_Arkansas**

| | ,--| ,----- Alvinia_bohemica_plant

| | | `--| ,-- Himeriella_muensteri

`--| |	`--| ,-- Kachaikestrobus_acuminatus

| | `----- Tomaxellia_biforme

| | ,-- Telemachus_aequatus

| | | ,-- Eathiestrobus_mackenziei

`--| |	| ,-- Cedrus_deodara

| | | | ,-- Mataia_podocarpoides

| | ,--| | ,--| ,-- Keteleeria_davidiana

| | | | | | `----- Abies_lasiocarpa

| | | `--| ,--| ,-- Pseudolarix_amabilis

| | | | | `--| ,-- Tsuga_canadensis

`--| |	| |	`----- Nothotsuga_longibracteata

| | `--| ,-- Pseudotsuga_menziesii

| | | ,----- Larix_decidua

| | `--| ,-- Cathaya_argyrophylla

| | `--| ,-- Pinus_contorta

| | `--| ,-- Picea_abies

| | `--| ,-- Schizolepidopsis_ediae

`--|	`--| ,-- Schizolepidopsis_daohugouensis

| `----- Schizolepidopsis_canicularis

| ,-- Sciadopitys_verticillata

| ,--| ,-- Taxus_wallichiana

| | `--| ,-- Cunninghamia_lanceolata

| | `--| ,-- Taiwania_cryptomerioides

| | `--| ,-- Athrotaxis_selaginoides

| | `--| ,-- Sequoia_sempervirens

| | `--| ,-- Cryptomeria_japonica

| | `--| ,-- Cupressus_funebris

`--|	`----- Callitris_muelleri

| ,-- Araucaria_mirabilis

| ,----- Araucaria_heterophylla

| ,--| ,-- Wollemia_nobilis

| | `----- Agathis_australis

| | ,-- Lagarostrobos_franklinii

`--|	,----- Prumnopitys_taxifolia

| ,--| ,-- Lepidothamnus_laxifolius

| | `----- Phyllocladus_aspleniifolius

`--| ,-- Saxegothaea_conspicua

`--| ,-- Microcachrys_tetragona

`--| ,-- Pherosphaera_hookeriana

`--| ,-- Acmopyle_pancheri

`--| ,-- Dacrydium_cupressinum

`--| ,-- Retrophyllum_rospigliosii

`----- Podocarpus_archboldii

Tree 38:

,-- Ginkgo_biloba

| ,-- Cordaixylon_dumusum

|--| | ,-- Emporia_lockardii

| | ,----- Hanskerpia_hamiltonensis

`--| | ,-- Voltzia_hexagona

| | | ,-- Nothodacrium_warrenii

`--| | ,----- Rissikia_media

| | | ,-- Pararaucaria_patagonica

`--| |	|	,-- **New_total_plant_from_Arkansas**

| | ,--| ,----- Alvinia_bohemica_plant

| | | `--| ,-- Himeriella_muensteri

`--| |	`--| ,-- Kachaikestrobus_acuminatus

| | `----- Tomaxellia_biforme

| | ,-- Telemachus_aequatus

| | | ,-- Eathiestrobus_mackenziei

`--| |	| ,-- Cedrus_deodara

| | ,--| | ,-- Keteleeria_davidiana

| | | | | ,----- Abies_lasiocarpa

| | | `--| ,--| ,-- Mataia_podocarpoides

| | | | | `--| ,-- Pseudolarix_amabilis

`--| |	| |	`--| ,-- Tsuga_canadensis

| | `--| `----- Nothotsuga_longibracteata

| | | ,-- Pseudotsuga_menziesii

| | | ,----- Larix_decidua

| | `--| ,-- Cathaya_argyrophylla

| | `--| ,-- Pinus_contorta

| | `--| ,-- Picea_abies

`--|	`--| ,-- Schizolepidopsis_ediae

| `--| ,-- Schizolepidopsis_daohugouensis

| `----- Schizolepidopsis_canicularis

| ,-- Sciadopitys_verticillata

| ,--| ,-- Taxus_wallichiana

| | `--| ,-- Cunninghamia_lanceolata

| | `--| ,-- Taiwania_cryptomerioides

| | `--| ,-- Athrotaxis_selaginoides

| | `--| ,-- Sequoia_sempervirens

| | `--| ,-- Cryptomeria_japonica

| | `--| ,-- Cupressus_funebris

`--|	`----- Callitris_muelleri

| ,-- Araucaria_mirabilis

| ,----- Araucaria_heterophylla

| ,--| ,-- Wollemia_nobilis

| | `----- Agathis_australis

| | ,-- Lagarostrobos_franklinii

`--|	,----- Prumnopitys_taxifolia

| ,--| ,-- Lepidothamnus_laxifolius

| | `----- Phyllocladus_aspleniifolius

`--| ,-- Saxegothaea_conspicua

`--| ,-- Microcachrys_tetragona

`--| ,-- Pherosphaera_hookeriana

`--| ,-- Acmopyle_pancheri

`--| ,-- Dacrydium_cupressinum

`--| ,-- Retrophyllum_rospigliosii

`----- Podocarpus_archboldii

Tree 39:

,-- Ginkgo_biloba

| ,-- Cordaixylon_dumusum

|--| | ,-- Emporia_lockardii

| | ,----- Hanskerpia_hamiltonensis

`--| | ,-- Voltzia_hexagona

| | | ,-- Nothodacrium_warrenii

`--| | ,----- Rissikia_media

| | | ,-- Pararaucaria_patagonica

`--| |	|	,-- **New_total_plant_from_Arkansas**

| | ,--| ,----- Alvinia_bohemica_plant

| | | `--| ,-- Himeriella_muensteri

`--| |	`--| ,-- Kachaikestrobus_acuminatus

| | `----- Tomaxellia_biforme

| | ,-- Telemachus_aequatus

| | | ,-- Eathiestrobus_mackenziei

`--| |	| ,-- Cedrus_deodara

| | | | ,-- Keteleeria_davidiana

| | ,--| | ,--| ,-- Mataia_podocarpoides

| | | | | | `----- Abies_lasiocarpa

| | | `--| ,--| ,-- Pseudolarix_amabilis

| | | | | `--| ,-- Tsuga_canadensis

`--| |	| |	`----- Nothotsuga_longibracteata

| | `--| ,-- Pseudotsuga_menziesii

| | | ,----- Larix_decidua

| | `--| ,-- Cathaya_argyrophylla

| | `--| ,-- Pinus_contorta

| | `--| ,-- Picea_abies

| | `--| ,-- Schizolepidopsis_ediae

`--|	`--| ,-- Schizolepidopsis_daohugouensis

| `----- Schizolepidopsis_canicularis

| ,-- Sciadopitys_verticillata

| ,--| ,-- Taxus_wallichiana

| | `--| ,-- Cunninghamia_lanceolata

| | `--| ,-- Taiwania_cryptomerioides

| | `--| ,-- Athrotaxis_selaginoides

| | `--| ,-- Sequoia_sempervirens

| | `--| ,-- Cryptomeria_japonica

| | `--| ,-- Cupressus_funebris

`--|	`----- Callitris_muelleri

| ,-- Araucaria_mirabilis

| ,----- Araucaria_heterophylla

| ,--| ,-- Wollemia_nobilis

| | `----- Agathis_australis

| | ,-- Lagarostrobos_franklinii

`--|	,----- Prumnopitys_taxifolia

| ,--| ,-- Lepidothamnus_laxifolius

| | `----- Phyllocladus_aspleniifolius

`--| ,-- Saxegothaea_conspicua

`--| ,-- Microcachrys_tetragona

`--| ,-- Pherosphaera_hookeriana

`--| ,-- Acmopyle_pancheri

`--| ,-- Dacrydium_cupressinum

`--| ,-- Retrophyllum_rospigliosii

`----- Podocarpus_archboldii

Tree 40:

,-- Ginkgo_biloba

| ,-- Cordaixylon_dumusum

|--| | ,-- Emporia_lockardii

| | ,----- Hanskerpia_hamiltonensis

`--| | ,-- Voltzia_hexagona

| | | ,-- Nothodacrium_warrenii

`--| | ,----- Rissikia_media

| | | ,-- Pararaucaria_patagonica

`--| |	|	,-- **New_total_plant_from_Arkansas**

| | ,--| ,----- Alvinia_bohemica_plant

| | | `--| ,-- Himeriella_muensteri

`--| |	`--| ,-- Kachaikestrobus_acuminatus

| | `----- Tomaxellia_biforme

| | ,-- Telemachus_aequatus

| | | ,-- Eathiestrobus_mackenziei

`--| |	| ,-- Cedrus_deodara

| | | | ,-- Keteleeria_davidiana

| | ,--| | ,----- Abies_lasiocarpa

| | | | | | ,-- Mataia_podocarpoides

| | | `--| ,--| ,----- Pseudolarix_amabilis

| | | | | `--| ,-- Tsuga_canadensis

`--| |	| |	`----- Nothotsuga_longibracteata

| | `--| ,-- Pseudotsuga_menziesii

| | | ,----- Larix_decidua

| | `--| ,-- Cathaya_argyrophylla

| | `--| ,-- Pinus_contorta

| | `--| ,-- Picea_abies

| | `--| ,-- Schizolepidopsis_ediae

`--|	`--| ,-- Schizolepidopsis_daohugouensis

| `----- Schizolepidopsis_canicularis

| ,-- Sciadopitys_verticillata

| ,--| ,-- Taxus_wallichiana

| | `--| ,-- Cunninghamia_lanceolata

| | `--| ,-- Taiwania_cryptomerioides

| | `--| ,-- Athrotaxis_selaginoides

| | `--| ,-- Sequoia_sempervirens

| | `--| ,-- Cryptomeria_japonica

| | `--| ,-- Cupressus_funebris

`--|	`----- Callitris_muelleri

| ,-- Araucaria_mirabilis

| ,----- Araucaria_heterophylla

| ,--| ,-- Wollemia_nobilis

| | `----- Agathis_australis

| | ,-- Lagarostrobos_franklinii

`--|	,----- Prumnopitys_taxifolia

| ,--| ,-- Lepidothamnus_laxifolius

| | `----- Phyllocladus_aspleniifolius

`--| ,-- Saxegothaea_conspicua

`--| ,-- Microcachrys_tetragona

`--| ,-- Pherosphaera_hookeriana

`--| ,-- Acmopyle_pancheri

`--| ,-- Dacrydium_cupressinum

`--| ,-- Retrophyllum_rospigliosii

`----- Podocarpus_archboldii

Tree 41:

,-- Ginkgo_biloba

| ,-- Cordaixylon_dumusum

|--| | ,-- Emporia_lockardii

| | ,----- Hanskerpia_hamiltonensis

`--| | ,-- Voltzia_hexagona

| | | ,-- Nothodacrium_warrenii

`--| | ,----- Rissikia_media

| | | ,-- Pararaucaria_patagonica

`--| |	|	,-- **New_total_plant_from_Arkansas**

| | ,--| ,----- Alvinia_bohemica_plant

| | | `--| ,-- Himeriella_muensteri

`--| |	`--| ,-- Kachaikestrobus_acuminatus

| | `----- Tomaxellia_biforme

| | ,-- Telemachus_aequatus

| | | ,-- Eathiestrobus_mackenziei

`--| |	| ,-- Cedrus_deodara

| | ,--| | ,-- Keteleeria_davidiana

| | | | | ,----- Abies_lasiocarpa

| | | `--| ,--| ,-- Pseudolarix_amabilis

| | | | | `--| ,-- Mataia_podocarpoides

`--| |	| |	`--| ,-- Tsuga_canadensis

| | `--| `----- Nothotsuga_longibracteata

| | | ,-- Pseudotsuga_menziesii

| | | ,----- Larix_decidua

| | `--| ,-- Cathaya_argyrophylla

| | `--| ,-- Pinus_contorta

| | `--| ,-- Picea_abies

`--|	`--| ,-- Schizolepidopsis_ediae

| `--| ,-- Schizolepidopsis_daohugouensis

| `----- Schizolepidopsis_canicularis

| ,-- Sciadopitys_verticillata

| ,--| ,-- Taxus_wallichiana

| | `--| ,-- Cunninghamia_lanceolata

| | `--| ,-- Taiwania_cryptomerioides

| | `--| ,-- Athrotaxis_selaginoides

| | `--| ,-- Sequoia_sempervirens

| | `--| ,-- Cryptomeria_japonica

| | `--| ,-- Cupressus_funebris

`--|	`----- Callitris_muelleri

| ,-- Araucaria_mirabilis

| ,----- Araucaria_heterophylla

| ,--| ,-- Wollemia_nobilis

| | `----- Agathis_australis

| | ,-- Lagarostrobos_franklinii

`--|	,----- Prumnopitys_taxifolia

| ,--| ,-- Lepidothamnus_laxifolius

| | `----- Phyllocladus_aspleniifolius

`--| ,-- Saxegothaea_conspicua

`--| ,-- Microcachrys_tetragona

`--| ,-- Pherosphaera_hookeriana

`--| ,-- Acmopyle_pancheri

`--| ,-- Dacrydium_cupressinum

`--| ,-- Retrophyllum_rospigliosii

`----- Podocarpus_archboldii

Tree 42:

,-- Ginkgo_biloba

| ,-- Cordaixylon_dumusum

|--| | ,-- Emporia_lockardii

| | ,----- Hanskerpia_hamiltonensis

`--| | ,-- Voltzia_hexagona

| | | ,-- Nothodacrium_warrenii

`--| | ,----- Rissikia_media

| | | ,-- Pararaucaria_patagonica

`--| |	|	,-- **New_total_plant_from_Arkansas**

| | ,--| ,----- Alvinia_bohemica_plant

| | | `--| ,-- Himeriella_muensteri

`--| |	`--| ,-- Kachaikestrobus_acuminatus

| | `----- Tomaxellia_biforme

| | ,-- Telemachus_aequatus

| | | ,-- Eathiestrobus_mackenziei

`--| |	| ,-- Cedrus_deodara

| | ,--| | ,-- Keteleeria_davidiana

| | | | | ,----- Abies_lasiocarpa

| | | `--| ,--| ,-- Pseudolarix_amabilis

| | | | | `--| ,-- Tsuga_canadensis

`--| |	| |	`--| ,-- Mataia_podocarpoides

| | `--| `----- Nothotsuga_longibracteata

| | | ,-- Pseudotsuga_menziesii

| | | ,----- Larix_decidua

| | `--| ,-- Cathaya_argyrophylla

| | `--| ,-- Pinus_contorta

| | `--| ,-- Picea_abies

`--|	`--| ,-- Schizolepidopsis_ediae

| `--| ,-- Schizolepidopsis_daohugouensis

| `----- Schizolepidopsis_canicularis

| ,-- Sciadopitys_verticillata

| ,--| ,-- Taxus_wallichiana

| | `--| ,-- Cunninghamia_lanceolata

| | `--| ,-- Taiwania_cryptomerioides

| | `--| ,-- Athrotaxis_selaginoides

| | `--| ,-- Sequoia_sempervirens

| | `--| ,-- Cryptomeria_japonica

| | `--| ,-- Cupressus_funebris

`--|	`----- Callitris_muelleri

| ,-- Araucaria_mirabilis

| ,----- Araucaria_heterophylla

| ,--| ,-- Wollemia_nobilis

| | `----- Agathis_australis

| | ,-- Lagarostrobos_franklinii

`--|	,----- Prumnopitys_taxifolia

| ,--| ,-- Lepidothamnus_laxifolius

| | `----- Phyllocladus_aspleniifolius

`--| ,-- Saxegothaea_conspicua

`--| ,-- Microcachrys_tetragona

`--| ,-- Pherosphaera_hookeriana

`--| ,-- Acmopyle_pancheri

`--| ,-- Dacrydium_cupressinum

`--| ,-- Retrophyllum_rospigliosii

`----- Podocarpus_archboldii

Tree 43:

,-- Ginkgo_biloba

| ,-- Cordaixylon_dumusum

|--| | ,-- Emporia_lockardii

| | ,----- Hanskerpia_hamiltonensis

`--| | ,-- Voltzia_hexagona

| | | ,-- Nothodacrium_warrenii

`--| | ,----- Rissikia_media

| | | ,-- Pararaucaria_patagonica

`--| |	|	,-- **New_total_plant_from_Arkansas**

| | ,--| ,----- Alvinia_bohemica_plant

| | | `--| ,-- Himeriella_muensteri

`--| |	`--| ,-- Kachaikestrobus_acuminatus

| | `----- Tomaxellia_biforme

| | ,-- Telemachus_aequatus

| | | ,-- Eathiestrobus_mackenziei

`--| |	| ,-- Cedrus_deodara

| | ,--| | ,-- Mataia_podocarpoides

| | | | | | ,-- Keteleeria_davidiana

| | | `--| ,--| ,----- Abies_lasiocarpa

| | | | | `--| ,-- Pseudolarix_amabilis

`--| |	| |	`--| ,-- Tsuga_canadensis

| | `--| `----- Nothotsuga_longibracteata

| | | ,-- Pseudotsuga_menziesii

| | | ,----- Larix_decidua

| | `--| ,-- Cathaya_argyrophylla

| | `--| ,-- Pinus_contorta

| | `--| ,-- Picea_abies

`--|	`--| ,-- Schizolepidopsis_ediae

| `--| ,-- Schizolepidopsis_daohugouensis

| `----- Schizolepidopsis_canicularis

| ,-- Sciadopitys_verticillata

| ,--| ,-- Taxus_wallichiana

| | `--| ,-- Cunninghamia_lanceolata

| | `--| ,-- Taiwania_cryptomerioides

| | `--| ,-- Athrotaxis_selaginoides

| | `--| ,-- Sequoia_sempervirens

| | `--| ,-- Cryptomeria_japonica

| | `--| ,-- Cupressus_funebris

`--|	`----- Callitris_muelleri

| ,-- Araucaria_mirabilis

| ,----- Araucaria_heterophylla

| ,--| ,-- Wollemia_nobilis

| | `----- Agathis_australis

| | ,-- Lagarostrobos_franklinii

`--|	,----- Prumnopitys_taxifolia

| ,--| ,-- Lepidothamnus_laxifolius

| | `----- Phyllocladus_aspleniifolius

`--| ,-- Saxegothaea_conspicua

`--| ,-- Microcachrys_tetragona

`--| ,-- Pherosphaera_hookeriana

`--| ,-- Acmopyle_pancheri

`--| ,-- Dacrydium_cupressinum

`--| ,-- Retrophyllum_rospigliosii

`----- Podocarpus_archboldii

Tree 44:

,-- Ginkgo_biloba

| ,-- Cordaixylon_dumusum

|--| | ,-- Emporia_lockardii

| | ,----- Hanskerpia_hamiltonensis

`--| | ,-- Voltzia_hexagona

| | | ,-- Nothodacrium_warrenii

`--| | ,----- Rissikia_media

| | | ,-- Pararaucaria_patagonica

`--| |	,--| ,-- **New_total_plant_from_Arkansas**

| | | `----- Alvinia_bohemica_plant

| | ,--| ,-- Himeriella_muensteri

`--| | `--| ,-- Kachaikestrobus_acuminatus

| | `----- Tomaxellia_biforme

| | ,-- Telemachus_aequatus

| | | ,-- Eathiestrobus_mackenziei

`--| |	| ,-- Cedrus_deodara

| | ,--| | ,-- Pseudotsuga_menziesii

| | | | | ,----- Larix_decidua

| | | `--| ,--| ,-- Cathaya_argyrophylla

| | | | | `--| ,-- Pinus_contorta

`--| |	| |	`----- Picea_abies

| | `--| ,-- Keteleeria_davidiana

| | | ,----- Abies_lasiocarpa

| | | | ,-- Pseudolarix_amabilis

| | `--| | ,-- Mataia_podocarpoides

| | `--| ,--| ,-- Tsuga_canadensis

| | | | `----- Nothotsuga_longibracteata

`--|	`--| ,-- Schizolepidopsis_ediae

| `--| ,-- Schizolepidopsis_daohugouensis

| `----- Schizolepidopsis_canicularis

| ,-- Sciadopitys_verticillata

| ,--| ,-- Taxus_wallichiana

| | `--| ,-- Cunninghamia_lanceolata

| | `--| ,-- Taiwania_cryptomerioides

| | `--| ,-- Athrotaxis_selaginoides

| | `--| ,-- Sequoia_sempervirens

| | `--| ,-- Cryptomeria_japonica

| | `--| ,-- Cupressus_funebris

`--|	`----- Callitris_muelleri

| ,-- Araucaria_mirabilis

| ,----- Araucaria_heterophylla

| ,--| ,-- Wollemia_nobilis

| | `----- Agathis_australis

| | ,-- Lagarostrobos_franklinii

`--|	,----- Prumnopitys_taxifolia

| ,--| ,-- Lepidothamnus_laxifolius

| | `----- Phyllocladus_aspleniifolius

`--| ,-- Saxegothaea_conspicua

`--| ,-- Microcachrys_tetragona

`--| ,-- Pherosphaera_hookeriana

`--| ,-- Acmopyle_pancheri

`--| ,-- Dacrydium_cupressinum

`--| ,-- Retrophyllum_rospigliosii

`----- Podocarpus_archboldii

Tree 45:

,-- Ginkgo_biloba

| ,-- Cordaixylon_dumusum

|--| | ,-- Emporia_lockardii

| | ,----- Hanskerpia_hamiltonensis

`--| | ,-- Voltzia_hexagona

| | | ,-- Nothodacrium_warrenii

`--| | ,----- Rissikia_media

| | | ,-- Pararaucaria_patagonica

`--| |	,--| ,-- **New_total_plant_from_Arkansas**

| | | `----- Alvinia_bohemica_plant

| | ,--| ,-- Himeriella_muensteri

`--| | `--| ,-- Kachaikestrobus_acuminatus

| | `----- Tomaxellia_biforme

| | ,-- Telemachus_aequatus

| | | ,-- Eathiestrobus_mackenziei

| | | | ,-- Cedrus_deodara

`--| |	| |	,-- Mataia_podocarpoides

| | ,--| | ,--| ,-- Keteleeria_davidiana

| | | | | | `----- Abies_lasiocarpa

| | | `--| ,--| ,-- Pseudolarix_amabilis

| | | | | `--| ,-- Tsuga_canadensis

`--| |	| |	`----- Nothotsuga_longibracteata

| | `--| ,-- Pseudotsuga_menziesii

| | | ,----- Larix_decidua

| | `--| ,-- Cathaya_argyrophylla

| | `--| ,-- Pinus_contorta

| | `--| ,-- Picea_abies

| | `--| ,-- Schizolepidopsis_ediae

`--|	`--| ,-- Schizolepidopsis_daohugouensis

| `----- Schizolepidopsis_canicularis

| ,-- Sciadopitys_verticillata

| ,--| ,-- Taxus_wallichiana

| | `--| ,-- Cunninghamia_lanceolata

| | `--| ,-- Taiwania_cryptomerioides

| | `--| ,-- Athrotaxis_selaginoides

| | `--| ,-- Sequoia_sempervirens

| | `--| ,-- Cryptomeria_japonica

| | `--| ,-- Cupressus_funebris

`--|	`----- Callitris_muelleri

| ,-- Araucaria_mirabilis

| ,----- Araucaria_heterophylla

| ,--| ,-- Wollemia_nobilis

| | `----- Agathis_australis

| | ,-- Lagarostrobos_franklinii

`--|	,----- Prumnopitys_taxifolia

| ,--| ,-- Lepidothamnus_laxifolius

| | `----- Phyllocladus_aspleniifolius

`--| ,-- Saxegothaea_conspicua

`--| ,-- Microcachrys_tetragona

`--| ,-- Pherosphaera_hookeriana

`--| ,-- Acmopyle_pancheri

`--| ,-- Dacrydium_cupressinum

`--| ,-- Retrophyllum_rospigliosii

`----- Podocarpus_archboldii

Tree 46:

,-- Ginkgo_biloba

| ,-- Cordaixylon_dumusum

|--| | ,-- Emporia_lockardii

| | ,----- Hanskerpia_hamiltonensis

`--| | ,-- Voltzia_hexagona

| | | ,-- Nothodacrium_warrenii

`--| | ,----- Rissikia_media

| | | ,-- Telemachus_aequatus

`--| | |	,-- Schizolepidopsis_ediae

| | | ,--| ,-- Schizolepidopsis_daohugouensis

| | | | `----- Schizolepidopsis_canicularis

`--| |	| ,-- Cedrus_deodara

| | ,--| | ,-- Mataia_podocarpoides

| | | | | ,--| ,-- Keteleeria_davidiana

| | | | | | `----- Abies_lasiocarpa

`--| | `--| ,--| ,-- Pseudolarix_amabilis

| | | | `--| ,-- Tsuga_canadensis

| | | | `----- Nothotsuga_longibracteata

| | `--| ,-- Pseudotsuga_menziesii

| | | ,----- Larix_decidua

| | `--| ,-- Cathaya_argyrophylla

| | `--| ,-- Pinus_contorta

| | `--| ,-- Eathiestrobus_mackenziei

`--|	`----- Picea_abies

| ,-- Sciadopitys_verticillata

| ,--| ,-- Taxus_wallichiana

| | `--| ,-- Cunninghamia_lanceolata

| | `--| ,-- Taiwania_cryptomerioides

| | `--| ,-- Athrotaxis_selaginoides

| | `--| ,-- Sequoia_sempervirens

| | `--| ,-- Cryptomeria_japonica

| | `--| ,-- Cupressus_funebris

| | `----- Callitris_muelleri

`--|	,-- Pararaucaria_patagonica

| | ,-- **New_total_plant_from_Arkansas**

| ,--| ,----- Alvinia_bohemica_plant

| | `--| ,-- Himeriella_muensteri

| | `--| ,-- Kachaikestrobus_acuminatus

| | `----- Tomaxellia_biforme

| | ,-- Araucaria_mirabilis

`--|	,----- Araucaria_heterophylla

| ,--| ,-- Wollemia_nobilis

| | `----- Agathis_australis

| | ,-- Lagarostrobos_franklinii

`--|	,----- Prumnopitys_taxifolia

| ,--| ,-- Lepidothamnus_laxifolius

| | `----- Phyllocladus_aspleniifolius

`--| ,-- Saxegothaea_conspicua

`--| ,-- Microcachrys_tetragona

`--| ,-- Pherosphaera_hookeriana

`--| ,-- Acmopyle_pancheri

`--| ,-- Dacrydium_cupressinum

`--| ,-- Retrophyllum_rospigliosii

`----- Podocarpus_archboldii

Tree 47:

,-- Ginkgo_biloba

| ,-- Cordaixylon_dumusum

|--| | ,-- Emporia_lockardii

| | ,----- Hanskerpia_hamiltonensis

`--| | ,-- Voltzia_hexagona

| | | ,-- Nothodacrium_warrenii

`--| | ,----- Rissikia_media

| | | ,-- Telemachus_aequatus

`--| | |	,-- Schizolepidopsis_ediae

| | | ,--| ,-- Schizolepidopsis_daohugouensis

| | | | `----- Schizolepidopsis_canicularis

`--| |	| ,-- Cedrus_deodara

| | ,--| | ,-- Keteleeria_davidiana

| | | | | ,----- Abies_lasiocarpa

| | | `--| ,--| ,-- Mataia_podocarpoides

`--| |	| | `--| ,-- Pseudolarix_amabilis

| | | | `--| ,-- Tsuga_canadensis

| | `--| `----- Nothotsuga_longibracteata

| | | ,-- Pseudotsuga_menziesii

| | | ,----- Larix_decidua

| | `--| ,-- Cathaya_argyrophylla

| | `--| ,-- Pinus_contorta

| | `--| ,-- Eathiestrobus_mackenziei

`--|	`----- Picea_abies

| ,-- Sciadopitys_verticillata

| ,--| ,-- Taxus_wallichiana

| | `--| ,-- Cunninghamia_lanceolata

| | `--| ,-- Taiwania_cryptomerioides

| | `--| ,-- Athrotaxis_selaginoides

| | `--| ,-- Sequoia_sempervirens

| | `--| ,-- Cryptomeria_japonica

| | `--| ,-- Cupressus_funebris

| | `----- Callitris_muelleri

`--|	,-- Pararaucaria_patagonica

| | ,-- **New_total_plant_from_Arkansas**

| ,--| ,----- Alvinia_bohemica_plant

| | `--| ,-- Himeriella_muensteri

| | `--| ,-- Kachaikestrobus_acuminatus

| | `----- Tomaxellia_biforme

| | ,-- Araucaria_mirabilis

`--|	,----- Araucaria_heterophylla

| ,--| ,-- Wollemia_nobilis

| | `----- Agathis_australis

| | ,-- Lagarostrobos_franklinii

`--|	,----- Prumnopitys_taxifolia

| ,--| ,-- Lepidothamnus_laxifolius

| | `----- Phyllocladus_aspleniifolius

`--| ,-- Saxegothaea_conspicua

`--| ,-- Microcachrys_tetragona

`--| ,-- Pherosphaera_hookeriana

`--| ,-- Acmopyle_pancheri

`--| ,-- Dacrydium_cupressinum

`--| ,-- Retrophyllum_rospigliosii

`----- Podocarpus_archboldii

Tree 48:

,-- Ginkgo_biloba

| ,-- Cordaixylon_dumusum

|--| | ,-- Emporia_lockardii

| | ,----- Hanskerpia_hamiltonensis

`--| | ,-- Voltzia_hexagona

| | | ,-- Nothodacrium_warrenii

`--| | ,----- Rissikia_media

| | | ,-- Telemachus_aequatus

`--| | |	,-- Schizolepidopsis_ediae

| | | ,--| ,-- Schizolepidopsis_daohugouensis

| | | | `----- Schizolepidopsis_canicularis

`--| |	| ,-- Cedrus_deodara

| | ,--| | ,-- Keteleeria_davidiana

| | | | | ,--| ,-- Mataia_podocarpoides

| | | | | | `----- Abies_lasiocarpa

`--| | `--| ,--| ,-- Pseudolarix_amabilis

| | | | `--| ,-- Tsuga_canadensis

| | | | `----- Nothotsuga_longibracteata

| | `--| ,-- Pseudotsuga_menziesii

| | | ,----- Larix_decidua

| | `--| ,-- Cathaya_argyrophylla

| | `--| ,-- Pinus_contorta

| | `--| ,-- Eathiestrobus_mackenziei

`--|	`----- Picea_abies

| ,-- Sciadopitys_verticillata

| ,--| ,-- Taxus_wallichiana

| | `--| ,-- Cunninghamia_lanceolata

| | `--| ,-- Taiwania_cryptomerioides

| | `--| ,-- Athrotaxis_selaginoides

| | `--| ,-- Sequoia_sempervirens

| | `--| ,-- Cryptomeria_japonica

| | `--| ,-- Cupressus_funebris

| | `----- Callitris_muelleri

`--|	,-- Pararaucaria_patagonica

| | ,-- **New_total_plant_from_Arkansas**

| ,--| ,----- Alvinia_bohemica_plant

| | `--| ,-- Himeriella_muensteri

| | `--| ,-- Kachaikestrobus_acuminatus

| | `----- Tomaxellia_biforme

| | ,-- Araucaria_mirabilis

`--|	,----- Araucaria_heterophylla

| ,--| ,-- Wollemia_nobilis

| | `----- Agathis_australis

| | ,-- Lagarostrobos_franklinii

`--|	,----- Prumnopitys_taxifolia

| ,--| ,-- Lepidothamnus_laxifolius

| | `----- Phyllocladus_aspleniifolius

`--| ,-- Saxegothaea_conspicua

`--| ,-- Microcachrys_tetragona

`--| ,-- Pherosphaera_hookeriana

`--| ,-- Acmopyle_pancheri

`--| ,-- Dacrydium_cupressinum

`--| ,-- Retrophyllum_rospigliosii

`----- Podocarpus_archboldii

Tree 49:

,-- Ginkgo_biloba

| ,-- Cordaixylon_dumusum

|--| | ,-- Emporia_lockardii

| | ,----- Hanskerpia_hamiltonensis

`--| | ,-- Voltzia_hexagona

| | | ,-- Nothodacrium_warrenii

`--| | ,----- Rissikia_media

| | | ,-- Telemachus_aequatus

`--| | |	,-- Schizolepidopsis_ediae

| | | ,--| ,-- Schizolepidopsis_daohugouensis

| | | | `----- Schizolepidopsis_canicularis

`--| |	| ,-- Cedrus_deodara

| | ,--| | ,-- Keteleeria_davidiana

| | | | | ,----- Abies_lasiocarpa

| | | `--| ,--| ,-- Pseudolarix_amabilis

`--| |	| | `--| ,-- Tsuga_canadensis

| | | | `--| ,-- Mataia_podocarpoides

| | `--| `----- Nothotsuga_longibracteata

| | | ,-- Pseudotsuga_menziesii

| | | ,----- Larix_decidua

| | `--| ,-- Cathaya_argyrophylla

| | `--| ,-- Pinus_contorta

| | `--| ,-- Eathiestrobus_mackenziei

`--|	`----- Picea_abies

| ,-- Sciadopitys_verticillata

| ,--| ,-- Taxus_wallichiana

| | `--| ,-- Cunninghamia_lanceolata

| | `--| ,-- Taiwania_cryptomerioides

| | `--| ,-- Athrotaxis_selaginoides

| | `--| ,-- Sequoia_sempervirens

| | `--| ,-- Cryptomeria_japonica

| | `--| ,-- Cupressus_funebris

| | `----- Callitris_muelleri

`--|	,-- Pararaucaria_patagonica

| | ,-- **New_total_plant_from_Arkansas**

| ,--| ,----- Alvinia_bohemica_plant

| | `--| ,-- Himeriella_muensteri

| | `--| ,-- Kachaikestrobus_acuminatus

| | `----- Tomaxellia_biforme

| | ,-- Araucaria_mirabilis

`--|	,----- Araucaria_heterophylla

| ,--| ,-- Wollemia_nobilis

| | `----- Agathis_australis

| | ,-- Lagarostrobos_franklinii

`--|	,----- Prumnopitys_taxifolia

| ,--| ,-- Lepidothamnus_laxifolius

| | `----- Phyllocladus_aspleniifolius

`--| ,-- Saxegothaea_conspicua

`--| ,-- Microcachrys_tetragona

`--| ,-- Pherosphaera_hookeriana

`--| ,-- Acmopyle_pancheri

`--| ,-- Dacrydium_cupressinum

`--| ,-- Retrophyllum_rospigliosii

`----- Podocarpus_archboldii

Tree 50:

,-- Ginkgo_biloba

| ,-- Cordaixylon_dumusum

|--| | ,-- Emporia_lockardii

`--| | ,-- Hanskerpia_hamiltonensis

`--| | ,-- Voltzia_hexagona

`--| |	,-- Nothodacrium_warrenii

| | ,----- Rissikia_media

`--| | ,-- Telemachus_aequatus

| | | ,-- Schizolepidopsis_ediae

| | | | ,-- Schizolepidopsis_daohugouensis

`--| | ,--| ,----- Schizolepidopsis_canicularis

| | | | | ,-- Cedrus_deodara

| | | `--| | ,-- Mataia_podocarpoides

| | | | | | ,-- Keteleeria_davidiana

`--| |	`--| ,--| ,----- Abies_lasiocarpa

| | | | `--| ,-- Pseudolarix_amabilis

| | | | `--| ,-- Tsuga_canadensis

| | `--| `----- Nothotsuga_longibracteata

| | | ,-- Pseudotsuga_menziesii

| | | ,----- Larix_decidua

`--|	`--| ,-- Cathaya_argyrophylla

| `--| ,-- Picea_abies

| `--| ,-- Eathiestrobus_mackenziei

| `----- Pinus_contorta

| ,-- **New_total_plant_from_Arkansas**

| ,----- Alvinia_bohemica_plant

| ,--| ,-- Himeriella_muensteri

| | `--| ,-- Kachaikestrobus_acuminatus

| | `----- Tomaxellia_biforme

| | ,-- Pararaucaria_patagonica

`--| |	,-- Sciadopitys_verticillata

| | ,--| ,-- Taxus_wallichiana

| | | `--| ,-- Cunninghamia_lanceolata

| | | `--| ,-- Taiwania_cryptomerioides

`--| |	`--| ,-- Athrotaxis_selaginoides

| | `--| ,-- Sequoia_sempervirens

| | `--| ,-- Cryptomeria_japonica

| | `--| ,-- Cupressus_funebris

`--|	`----- Callitris_muelleri

| ,-- Araucaria_mirabilis

| ,----- Araucaria_heterophylla

| ,--| ,-- Wollemia_nobilis

| | `----- Agathis_australis

| | ,-- Lagarostrobos_franklinii

`--|	,----- Prumnopitys_taxifolia

| ,--| ,-- Lepidothamnus_laxifolius

| | `----- Phyllocladus_aspleniifolius

`--| ,-- Saxegothaea_conspicua

`--| ,-- Microcachrys_tetragona

`--| ,-- Pherosphaera_hookeriana

`--| ,-- Acmopyle_pancheri

`--| ,-- Dacrydium_cupressinum

`--| ,-- Retrophyllum_rospigliosii

`----- Podocarpus_archboldii

Tree 51:

,-- Ginkgo_biloba

| ,-- Cordaixylon_dumusum

|--| | ,-- Emporia_lockardii

`--| ,----- Hanskerpia_hamiltonensis

| | ,-- Voltzia_hexagona

`--| |	,-- Nothodacrium_warrenii

| | ,----- Rissikia_media

`--| | ,-- Telemachus_aequatus

| | | ,-- Schizolepidopsis_ediae

| | | | ,-- Schizolepidopsis_daohugouensis

`--| |	| ,----- Schizolepidopsis_canicularis

| | ,--| | ,-- Cedrus_deodara

| | | | | | ,-- Keteleeria_davidiana

| | | `--| | ,--| ,-- Mataia_podocarpoides

`--| |	| |	| `----- Abies_lasiocarpa

| | `--| ,--| ,-- Pseudolarix_amabilis

| | | | `--| ,-- Tsuga_canadensis

| | | | `----- Nothotsuga_longibracteata

| | `--| ,-- Pseudotsuga_menziesii

| | | ,----- Larix_decidua

| | `--| ,-- Cathaya_argyrophylla

`--|	`--| ,-- Picea_abies

| `--| ,-- Eathiestrobus_mackenziei

| `----- Pinus_contorta

| ,-- **New_total_plant_from_Arkansas**

| ,----- Alvinia_bohemica_plant

| ,--| ,-- Himeriella_muensteri

| | `--| ,-- Kachaikestrobus_acuminatus

| | `----- Tomaxellia_biforme

| | ,-- Pararaucaria_patagonica

`--| |	,-- Sciadopitys_verticillata

| | ,--| ,-- Taxus_wallichiana

| | | `--| ,-- Cunninghamia_lanceolata

| | | `--| ,-- Taiwania_cryptomerioides

`--| |	`--| ,-- Athrotaxis_selaginoides

| | `--| ,-- Sequoia_sempervirens

| | `--| ,-- Cryptomeria_japonica

| | `--| ,-- Cupressus_funebris

`--|	`----- Callitris_muelleri

| ,-- Araucaria_mirabilis

| ,----- Araucaria_heterophylla

| ,--| ,-- Wollemia_nobilis

| | `----- Agathis_australis

| | ,-- Lagarostrobos_franklinii

`--|	,----- Prumnopitys_taxifolia

| ,--| ,-- Lepidothamnus_laxifolius

| | `----- Phyllocladus_aspleniifolius

`--| ,-- Saxegothaea_conspicua

`--| ,-- Microcachrys_tetragona

`--| ,-- Pherosphaera_hookeriana

`--| ,-- Acmopyle_pancheri

`--| ,-- Dacrydium_cupressinum

`--| ,-- Retrophyllum_rospigliosii

`----- Podocarpus_archboldii

Tree 52:

,-- Ginkgo_biloba

| ,-- Cordaixylon_dumusum

|--| | ,-- Emporia_lockardii

`--| ,----- Hanskerpia_hamiltonensis

| | ,-- Voltzia_hexagona

`--| |	,-- Nothodacrium_warrenii

| | ,----- Rissikia_media

`--| | ,-- Telemachus_aequatus

| | | ,-- Schizolepidopsis_ediae

| | | | ,-- Schizolepidopsis_daohugouensis

`--| |	| ,----- Schizolepidopsis_canicularis

| | ,--| | ,-- Cedrus_deodara

| | | | | | ,-- Keteleeria_davidiana

| | | `--| | ,----- Abies_lasiocarpa

`--| |	| |	|	,-- Mataia_podocarpoides

| | `--| ,--| ,----- Pseudolarix_amabilis

| | | | `--| ,-- Tsuga_canadensis

| | | | `----- Nothotsuga_longibracteata

| | `--| ,-- Pseudotsuga_menziesii

| | | ,----- Larix_decidua

| | `--| ,-- Cathaya_argyrophylla

`--|	`--| ,-- Picea_abies

| `--| ,-- Eathiestrobus_mackenziei

| `----- Pinus_contorta

| ,-- **New_total_plant_from_Arkansas**

| ,----- Alvinia_bohemica_plant

| ,--| ,-- Himeriella_muensteri

| | `--| ,-- Kachaikestrobus_acuminatus

| | `----- Tomaxellia_biforme

| | ,-- Pararaucaria_patagonica

`--| |	,-- Sciadopitys_verticillata

| | ,--| ,-- Taxus_wallichiana

| | | `--| ,-- Cunninghamia_lanceolata

| | | `--| ,-- Taiwania_cryptomerioides

`--| |	`--| ,-- Athrotaxis_selaginoides

| | `--| ,-- Sequoia_sempervirens

| | `--| ,-- Cryptomeria_japonica

| | `--| ,-- Cupressus_funebris

`--|	`----- Callitris_muelleri

| ,-- Araucaria_mirabilis

| ,----- Araucaria_heterophylla

| ,--| ,-- Wollemia_nobilis

| | `----- Agathis_australis

| | ,-- Lagarostrobos_franklinii

`--|	,----- Prumnopitys_taxifolia

| ,--| ,-- Lepidothamnus_laxifolius

| | `----- Phyllocladus_aspleniifolius

`--| ,-- Saxegothaea_conspicua

`--| ,-- Microcachrys_tetragona

`--| ,-- Pherosphaera_hookeriana

`--| ,-- Acmopyle_pancheri

`--| ,-- Dacrydium_cupressinum

`--| ,-- Retrophyllum_rospigliosii

`----- Podocarpus_archboldii

Tree 53:

,-- Ginkgo_biloba

| ,-- Cordaixylon_dumusum

|--| | ,-- Emporia_lockardii

`--| ,----- Hanskerpia_hamiltonensis

| | ,-- Voltzia_hexagona

`--| |	,-- Nothodacrium_warrenii

| | ,----- Rissikia_media

`--| | ,-- Telemachus_aequatus

| | | ,-- Schizolepidopsis_ediae

| | | | ,-- Schizolepidopsis_daohugouensis

`--| | ,--| ,----- Schizolepidopsis_canicularis

| | | | | ,-- Cedrus_deodara

| | | `--| | ,-- Keteleeria_davidiana

| | | | | ,----- Abies_lasiocarpa

`--| |	`--| ,--| ,-- Pseudolarix_amabilis

| | | | `--| ,-- Mataia_podocarpoides

| | | | `--| ,-- Tsuga_canadensis

| | `--| `----- Nothotsuga_longibracteata

| | | ,-- Pseudotsuga_menziesii

| | | ,----- Larix_decidua

`--|	`--| ,-- Cathaya_argyrophylla

| `--| ,-- Picea_abies

| `--| ,-- Eathiestrobus_mackenziei

| `----- Pinus_contorta

| ,-- **New_total_plant_from_Arkansas**

| ,----- Alvinia_bohemica_plant

| ,--| ,-- Himeriella_muensteri

| | `--| ,-- Kachaikestrobus_acuminatus

| | `----- Tomaxellia_biforme

| | ,-- Pararaucaria_patagonica

`--| |	,-- Sciadopitys_verticillata

| | ,--| ,-- Taxus_wallichiana

| | | `--| ,-- Cunninghamia_lanceolata

| | | `--| ,-- Taiwania_cryptomerioides

`--| |	`--| ,-- Athrotaxis_selaginoides

| | `--| ,-- Sequoia_sempervirens

| | `--| ,-- Cryptomeria_japonica

| | `--| ,-- Cupressus_funebris

`--|	`----- Callitris_muelleri

| ,-- Araucaria_mirabilis

| ,----- Araucaria_heterophylla

| ,--| ,-- Wollemia_nobilis

| | `----- Agathis_australis

| | ,-- Lagarostrobos_franklinii

`--|	,----- Prumnopitys_taxifolia

| ,--| ,-- Lepidothamnus_laxifolius

| | `----- Phyllocladus_aspleniifolius

`--| ,-- Saxegothaea_conspicua

`--| ,-- Microcachrys_tetragona

`--| ,-- Pherosphaera_hookeriana

`--| ,-- Acmopyle_pancheri

`--| ,-- Dacrydium_cupressinum

`--| ,-- Retrophyllum_rospigliosii

`----- Podocarpus_archboldii

Tree 54:

,-- Ginkgo_biloba

| ,-- Cordaixylon_dumusum

|--| | ,-- Emporia_lockardii

`--| ,----- Hanskerpia_hamiltonensis

| | ,-- Voltzia_hexagona

`--| |	,-- Nothodacrium_warrenii

| | ,----- Rissikia_media

`--| | ,-- Telemachus_aequatus

| | | ,-- Schizolepidopsis_ediae

| | | | ,-- Schizolepidopsis_daohugouensis

`--| | ,--| ,----- Schizolepidopsis_canicularis

| | | | | ,-- Cedrus_deodara

| | | `--| | ,-- Keteleeria_davidiana

| | | | | ,----- Abies_lasiocarpa

`--| |	`--| ,--| ,-- Pseudolarix_amabilis

| | | | `--| ,-- Tsuga_canadensis

| | | | `--| ,-- Mataia_podocarpoides

| | `--| `----- Nothotsuga_longibracteata

| | | ,-- Pseudotsuga_menziesii

| | | ,----- Larix_decidua

`--|	`--| ,-- Cathaya_argyrophylla

| `--| ,-- Picea_abies

| `--| ,-- Eathiestrobus_mackenziei

| `----- Pinus_contorta

| ,-- **New_total_plant_from_Arkansas**

| ,----- Alvinia_bohemica_plant

| ,--| ,-- Himeriella_muensteri

| | `--| ,-- Kachaikestrobus_acuminatus

| | `----- Tomaxellia_biforme

| | ,-- Pararaucaria_patagonica

`--| |	,-- Sciadopitys_verticillata

| | ,--| ,-- Taxus_wallichiana

| | | `--| ,-- Cunninghamia_lanceolata

| | | `--| ,-- Taiwania_cryptomerioides

`--| |	`--| ,-- Athrotaxis_selaginoides

| | `--| ,-- Sequoia_sempervirens

| | `--| ,-- Cryptomeria_japonica

| | `--| ,-- Cupressus_funebris

`--|	`----- Callitris_muelleri

| ,-- Araucaria_mirabilis

| ,----- Araucaria_heterophylla

| ,--| ,-- Wollemia_nobilis

| | `----- Agathis_australis

| | ,-- Lagarostrobos_franklinii

`--|	,----- Prumnopitys_taxifolia

| ,--| ,-- Lepidothamnus_laxifolius

| | `----- Phyllocladus_aspleniifolius

`--| ,-- Saxegothaea_conspicua

`--| ,-- Microcachrys_tetragona

`--| ,-- Pherosphaera_hookeriana

`--| ,-- Acmopyle_pancheri

`--| ,-- Dacrydium_cupressinum

`--| ,-- Retrophyllum_rospigliosii

`----- Podocarpus_archboldii

Tree 55:

,-- Ginkgo_biloba

| ,-- Cordaixylon_dumusum

|--| | ,-- Emporia_lockardii

`--| ,----- Hanskerpia_hamiltonensis

| | ,-- Voltzia_hexagona

`--| |	,-- Nothodacrium_warrenii

| | ,----- Rissikia_media

`--| | ,-- Telemachus_aequatus

| | | ,-- Schizolepidopsis_ediae

| | | | ,-- Schizolepidopsis_daohugouensis

`--| | ,--| ,----- Schizolepidopsis_canicularis

| | | | | ,-- Cedrus_deodara

| | | `--| | ,-- Keteleeria_davidiana

| | | | | ,----- Abies_lasiocarpa

`--| |	`--| ,--| ,-- Mataia_podocarpoides

| | | | `--| ,-- Pseudolarix_amabilis

| | | | `--| ,-- Tsuga_canadensis

| | `--| `----- Nothotsuga_longibracteata

| | | ,-- Pseudotsuga_menziesii

| | | ,----- Larix_decidua

`--|	`--| ,-- Cathaya_argyrophylla

| `--| ,-- Picea_abies

| `--| ,-- Eathiestrobus_mackenziei

| `----- Pinus_contorta

| ,-- **New_total_plant_from_Arkansas**

| ,----- Alvinia_bohemica_plant

| ,--| ,-- Himeriella_muensteri

| | `--| ,-- Kachaikestrobus_acuminatus

| | `----- Tomaxellia_biforme

| | ,-- Pararaucaria_patagonica

`--| |	,-- Sciadopitys_verticillata

| | ,--| ,-- Taxus_wallichiana

| | | `--| ,-- Cunninghamia_lanceolata

| | | `--| ,-- Taiwania_cryptomerioides

`--| |	`--| ,-- Athrotaxis_selaginoides

| | `--| ,-- Sequoia_sempervirens

| | `--| ,-- Cryptomeria_japonica

| | `--| ,-- Cupressus_funebris

`--|	`----- Callitris_muelleri

| ,-- Araucaria_mirabilis

| ,----- Araucaria_heterophylla

| ,--| ,-- Wollemia_nobilis

| | `----- Agathis_australis

| | ,-- Lagarostrobos_franklinii

`--|	,----- Prumnopitys_taxifolia

| ,--| ,-- Lepidothamnus_laxifolius

| | `----- Phyllocladus_aspleniifolius

`--| ,-- Saxegothaea_conspicua

`--| ,-- Microcachrys_tetragona

`--| ,-- Pherosphaera_hookeriana

`--| ,-- Acmopyle_pancheri

`--| ,-- Dacrydium_cupressinum

`--| ,-- Retrophyllum_rospigliosii

`----- Podocarpus_archboldii

Tree 56:

,-- Ginkgo_biloba

| ,-- Cordaixylon_dumusum

|--| | ,-- Emporia_lockardii

`--| ,----- Hanskerpia_hamiltonensis

| | ,-- Voltzia_hexagona

`--| |	,-- Nothodacrium_warrenii

| | ,----- Rissikia_media

`--| | ,-- Telemachus_aequatus

| | | ,-- Schizolepidopsis_ediae

| | | | ,-- Schizolepidopsis_daohugouensis

`--| |	| ,----- Schizolepidopsis_canicularis

| | ,--| | ,-- Cedrus_deodara

| | | | | | ,-- Mataia_podocarpoides

| | | `--| | ,--| ,-- Keteleeria_davidiana

`--| |	| |	| `----- Abies_lasiocarpa

| | `--| ,--| ,-- Pseudolarix_amabilis

| | | | `--| ,-- Tsuga_canadensis

| | | | `----- Nothotsuga_longibracteata

| | `--| ,-- Pseudotsuga_menziesii

| | | ,----- Larix_decidua

| | `--| ,-- Cathaya_argyrophylla

`--|	`--| ,-- Picea_abies

| `--| ,-- Eathiestrobus_mackenziei

| `----- Pinus_contorta

| ,-- **New_total_plant_from_Arkansas**

| ,----- Alvinia_bohemica_plant

| ,--| ,-- Himeriella_muensteri

| | `--| ,-- Kachaikestrobus_acuminatus

| | `----- Tomaxellia_biforme

| | ,-- Pararaucaria_patagonica

`--| |	,-- Sciadopitys_verticillata

| | ,--| ,-- Taxus_wallichiana

| | | `--| ,-- Cunninghamia_lanceolata

| | | `--| ,-- Taiwania_cryptomerioides

`--| |	`--| ,-- Athrotaxis_selaginoides

| | `--| ,-- Sequoia_sempervirens

| | `--| ,-- Cryptomeria_japonica

| | `--| ,-- Cupressus_funebris

`--|	`----- Callitris_muelleri

| ,-- Araucaria_mirabilis

| ,----- Araucaria_heterophylla

| ,--| ,-- Wollemia_nobilis

| | `----- Agathis_australis

| | ,-- Lagarostrobos_franklinii

`--|	,----- Prumnopitys_taxifolia

| ,--| ,-- Lepidothamnus_laxifolius

| | `----- Phyllocladus_aspleniifolius

`--| ,-- Saxegothaea_conspicua

`--| ,-- Microcachrys_tetragona

`--| ,-- Pherosphaera_hookeriana

`--| ,-- Acmopyle_pancheri

`--| ,-- Dacrydium_cupressinum

`--| ,-- Retrophyllum_rospigliosii

`----- Podocarpus_archboldii

Tree 57:

,-- Ginkgo_biloba

| ,-- Cordaixylon_dumusum

|--| | ,-- Emporia_lockardii

`--| | ,-- Hanskerpia_hamiltonensis

`--| | ,-- Voltzia_hexagona

`--| |	,-- Nothodacrium_warrenii

| | ,----- Rissikia_media

`--| | ,-- Telemachus_aequatus

| | | ,-- Schizolepidopsis_ediae

| | | ,--| ,-- Schizolepidopsis_daohugouensis

`--| |	| `----- Schizolepidopsis_canicularis

| | | ,-- Cedrus_deodara

| | ,--| | ,-- Mataia_podocarpoides

| | | | | | ,-- Keteleeria_davidiana

`--| | `--| ,--| ,----- Abies_lasiocarpa

| | | | `--| ,-- Pseudolarix_amabilis

| | | | `--| ,-- Tsuga_canadensis

| | `--| `----- Nothotsuga_longibracteata

| | | ,-- Pseudotsuga_menziesii

| | | ,----- Larix_decidua

| | `--| ,-- Cathaya_argyrophylla

`--|	`--| ,-- Pinus_contorta

| `--| ,-- Eathiestrobus_mackenziei

| `----- Picea_abies

| ,-- **New_total_plant_from_Arkansas**

| ,----- Alvinia_bohemica_plant

| ,--| ,-- Himeriella_muensteri

| | `--| ,-- Kachaikestrobus_acuminatus

| | `----- Tomaxellia_biforme

| | ,-- Pararaucaria_patagonica

`--| |	,-- Sciadopitys_verticillata

| | ,--| ,-- Taxus_wallichiana

| | | `--| ,-- Cunninghamia_lanceolata

| | | `--| ,-- Taiwania_cryptomerioides

`--| |	`--| ,-- Athrotaxis_selaginoides

| | `--| ,-- Sequoia_sempervirens

| | `--| ,-- Cryptomeria_japonica

| | `--| ,-- Cupressus_funebris

`--|	`----- Callitris_muelleri

| ,-- Araucaria_mirabilis

| ,----- Araucaria_heterophylla

| ,--| ,-- Wollemia_nobilis

| | `----- Agathis_australis

| | ,-- Lagarostrobos_franklinii

`--|	,----- Prumnopitys_taxifolia

| ,--| ,-- Lepidothamnus_laxifolius

| | `----- Phyllocladus_aspleniifolius

`--| ,-- Saxegothaea_conspicua

`--| ,-- Microcachrys_tetragona

`--| ,-- Pherosphaera_hookeriana

`--| ,-- Acmopyle_pancheri

`--| ,-- Dacrydium_cupressinum

`--| ,-- Retrophyllum_rospigliosii

`----- Podocarpus_archboldii

Tree 58:

,-- Ginkgo_biloba

| ,-- Cordaixylon_dumusum

|--| | ,-- Emporia_lockardii

`--| ,----- Hanskerpia_hamiltonensis

| | ,-- Voltzia_hexagona

`--| |	,-- Nothodacrium_warrenii

| | ,----- Rissikia_media

`--| | ,-- Telemachus_aequatus

| | | ,-- Schizolepidopsis_ediae

| | | ,--| ,-- Schizolepidopsis_daohugouensis

`--| |	| `----- Schizolepidopsis_canicularis

| | | ,-- Cedrus_deodara

| | ,--| | ,-- Keteleeria_davidiana

| | | | | ,--| ,-- Mataia_podocarpoides

`--| | | |	| `----- Abies_lasiocarpa

| | `--| ,--| ,-- Pseudolarix_amabilis

| | | | `--| ,-- Tsuga_canadensis

| | | | `----- Nothotsuga_longibracteata

| | `--| ,-- Pseudotsuga_menziesii

| | | ,----- Larix_decidua

| | `--| ,-- Cathaya_argyrophylla

`--|	`--| ,-- Pinus_contorta

| `--| ,-- Eathiestrobus_mackenziei

| `----- Picea_abies

| ,-- **New_total_plant_from_Arkansas**

| ,----- Alvinia_bohemica_plant

| ,--| ,-- Himeriella_muensteri

| | `--| ,-- Kachaikestrobus_acuminatus

| | `----- Tomaxellia_biforme

| | ,-- Pararaucaria_patagonica

`--| |	,-- Sciadopitys_verticillata

| | ,--| ,-- Taxus_wallichiana

| | | `--| ,-- Cunninghamia_lanceolata

| | | `--| ,-- Taiwania_cryptomerioides

`--| |	`--| ,-- Athrotaxis_selaginoides

| | `--| ,-- Sequoia_sempervirens

| | `--| ,-- Cryptomeria_japonica

| | `--| ,-- Cupressus_funebris

`--|	`----- Callitris_muelleri

| ,-- Araucaria_mirabilis

| ,----- Araucaria_heterophylla

| ,--| ,-- Wollemia_nobilis

| | `----- Agathis_australis

| | ,-- Lagarostrobos_franklinii

`--|	,----- Prumnopitys_taxifolia

| ,--| ,-- Lepidothamnus_laxifolius

| | `----- Phyllocladus_aspleniifolius

`--| ,-- Saxegothaea_conspicua

`--| ,-- Microcachrys_tetragona

`--| ,-- Pherosphaera_hookeriana

`--| ,-- Acmopyle_pancheri

`--| ,-- Dacrydium_cupressinum

`--| ,-- Retrophyllum_rospigliosii

`----- Podocarpus_archboldii

Tree 59:

,-- Ginkgo_biloba

| ,-- Cordaixylon_dumusum

|--| | ,-- Emporia_lockardii

`--| ,----- Hanskerpia_hamiltonensis

| | ,-- Voltzia_hexagona

`--| |	,-- Nothodacrium_warrenii

| | ,----- Rissikia_media

`--| | ,-- Telemachus_aequatus

| | | ,-- Schizolepidopsis_ediae

| | | ,--| ,-- Schizolepidopsis_daohugouensis

`--| |	| `----- Schizolepidopsis_canicularis

| | | ,-- Cedrus_deodara

| | ,--| | ,-- Keteleeria_davidiana

| | | | | ,----- Abies_lasiocarpa

`--| | | |	|	,-- Mataia_podocarpoides

| | `--| ,--| ,----- Pseudolarix_amabilis

| | | | `--| ,-- Tsuga_canadensis

| | | | `----- Nothotsuga_longibracteata

| | `--| ,-- Pseudotsuga_menziesii

| | | ,----- Larix_decidua

| | `--| ,-- Cathaya_argyrophylla

`--|	`--| ,-- Pinus_contorta

| `--| ,-- Eathiestrobus_mackenziei

| `----- Picea_abies

| ,-- **New_total_plant_from_Arkansas**

| ,----- Alvinia_bohemica_plant

| ,--| ,-- Himeriella_muensteri

| | `--| ,-- Kachaikestrobus_acuminatus

| | `----- Tomaxellia_biforme

| | ,-- Pararaucaria_patagonica

`--| |	,-- Sciadopitys_verticillata

| | ,--| ,-- Taxus_wallichiana

| | | `--| ,-- Cunninghamia_lanceolata

| | | `--| ,-- Taiwania_cryptomerioides

`--| |	`--| ,-- Athrotaxis_selaginoides

| | `--| ,-- Sequoia_sempervirens

| | `--| ,-- Cryptomeria_japonica

| | `--| ,-- Cupressus_funebris

`--|	`----- Callitris_muelleri

| ,-- Araucaria_mirabilis

| ,----- Araucaria_heterophylla

| ,--| ,-- Wollemia_nobilis

| | `----- Agathis_australis

| | ,-- Lagarostrobos_franklinii

`--|	,----- Prumnopitys_taxifolia

| ,--| ,-- Lepidothamnus_laxifolius

| | `----- Phyllocladus_aspleniifolius

`--| ,-- Saxegothaea_conspicua

`--| ,-- Microcachrys_tetragona

`--| ,-- Pherosphaera_hookeriana

`--| ,-- Acmopyle_pancheri

`--| ,-- Dacrydium_cupressinum

`--| ,-- Retrophyllum_rospigliosii

`----- Podocarpus_archboldii

Tree 60:

,-- Ginkgo_biloba

| ,-- Cordaixylon_dumusum

|--| | ,-- Emporia_lockardii

`--| ,----- Hanskerpia_hamiltonensis

| | ,-- Voltzia_hexagona

`--| |	,-- Nothodacrium_warrenii

| | ,----- Rissikia_media

`--| | ,-- Telemachus_aequatus

| | | ,-- Schizolepidopsis_ediae

| | | ,--| ,-- Schizolepidopsis_daohugouensis

`--| |	| `----- Schizolepidopsis_canicularis

| | | ,-- Cedrus_deodara

| | ,--| | ,-- Keteleeria_davidiana

| | | | | ,----- Abies_lasiocarpa

`--| | `--| ,--| ,-- Pseudolarix_amabilis

| | | | `--| ,-- Mataia_podocarpoides

| | | | `--| ,-- Tsuga_canadensis

| | `--| `----- Nothotsuga_longibracteata

| | | ,-- Pseudotsuga_menziesii

| | | ,----- Larix_decidua

| | `--| ,-- Cathaya_argyrophylla

`--|	`--| ,-- Pinus_contorta

| `--| ,-- Eathiestrobus_mackenziei

| `----- Picea_abies

| ,-- **New_total_plant_from_Arkansas**

| ,----- Alvinia_bohemica_plant

| ,--| ,-- Himeriella_muensteri

| | `--| ,-- Kachaikestrobus_acuminatus

| | `----- Tomaxellia_biforme

| | ,-- Pararaucaria_patagonica

`--| |	,-- Sciadopitys_verticillata

| | ,--| ,-- Taxus_wallichiana

| | | `--| ,-- Cunninghamia_lanceolata

| | | `--| ,-- Taiwania_cryptomerioides

`--| |	`--| ,-- Athrotaxis_selaginoides

| | `--| ,-- Sequoia_sempervirens

| | `--| ,-- Cryptomeria_japonica

| | `--| ,-- Cupressus_funebris

`--|	`----- Callitris_muelleri

| ,-- Araucaria_mirabilis

| ,----- Araucaria_heterophylla

| ,--| ,-- Wollemia_nobilis

| | `----- Agathis_australis

| | ,-- Lagarostrobos_franklinii

`--|	,----- Prumnopitys_taxifolia

| ,--| ,-- Lepidothamnus_laxifolius

| | `----- Phyllocladus_aspleniifolius

`--| ,-- Saxegothaea_conspicua

`--| ,-- Microcachrys_tetragona

`--| ,-- Pherosphaera_hookeriana

`--| ,-- Acmopyle_pancheri

`--| ,-- Dacrydium_cupressinum

`--| ,-- Retrophyllum_rospigliosii

`----- Podocarpus_archboldii

Tree 61:

,-- Ginkgo_biloba

| ,-- Cordaixylon_dumusum

|--| | ,-- Emporia_lockardii

`--| ,----- Hanskerpia_hamiltonensis

| | ,-- Voltzia_hexagona

`--| |	,-- Nothodacrium_warrenii

| | ,----- Rissikia_media

`--| | ,-- Telemachus_aequatus

| | | ,-- Schizolepidopsis_ediae

| | | ,--| ,-- Schizolepidopsis_daohugouensis

`--| |	| `----- Schizolepidopsis_canicularis

| | | ,-- Cedrus_deodara

| | ,--| | ,-- Keteleeria_davidiana

| | | | | ,----- Abies_lasiocarpa

`--| | `--| ,--| ,-- Pseudolarix_amabilis

| | | | `--| ,-- Tsuga_canadensis

| | | | `--| ,-- Mataia_podocarpoides

| | `--| `----- Nothotsuga_longibracteata

| | | ,-- Pseudotsuga_menziesii

| | | ,----- Larix_decidua

| | `--| ,-- Cathaya_argyrophylla

`--|	`--| ,-- Pinus_contorta

| `--| ,-- Eathiestrobus_mackenziei

| `----- Picea_abies

| ,-- **New_total_plant_from_Arkansas**

| ,----- Alvinia_bohemica_plant

| ,--| ,-- Himeriella_muensteri

| | `--| ,-- Kachaikestrobus_acuminatus

| | `----- Tomaxellia_biforme

| | ,-- Pararaucaria_patagonica

`--| |	,-- Sciadopitys_verticillata

| | ,--| ,-- Taxus_wallichiana

| | | `--| ,-- Cunninghamia_lanceolata

| | | `--| ,-- Taiwania_cryptomerioides

`--| |	`--| ,-- Athrotaxis_selaginoides

| | `--| ,-- Sequoia_sempervirens

| | `--| ,-- Cryptomeria_japonica

| | `--| ,-- Cupressus_funebris

`--|	`----- Callitris_muelleri

| ,-- Araucaria_mirabilis

| ,----- Araucaria_heterophylla

| ,--| ,-- Wollemia_nobilis

| | `----- Agathis_australis

| | ,-- Lagarostrobos_franklinii

`--|	,----- Prumnopitys_taxifolia

| ,--| ,-- Lepidothamnus_laxifolius

| | `----- Phyllocladus_aspleniifolius

`--| ,-- Saxegothaea_conspicua

`--| ,-- Microcachrys_tetragona

`--| ,-- Pherosphaera_hookeriana

`--| ,-- Acmopyle_pancheri

`--| ,-- Dacrydium_cupressinum

`--| ,-- Retrophyllum_rospigliosii

`----- Podocarpus_archboldii

Tree 62:

,-- Ginkgo_biloba

| ,-- Cordaixylon_dumusum

|--| | ,-- Emporia_lockardii

`--| ,----- Hanskerpia_hamiltonensis

| | ,-- Voltzia_hexagona

`--| |	,-- Nothodacrium_warrenii

| | ,----- Rissikia_media

`--| | ,-- Telemachus_aequatus

| | | ,-- Schizolepidopsis_ediae

| | | ,--| ,-- Schizolepidopsis_daohugouensis

`--| |	| `----- Schizolepidopsis_canicularis

| | | ,-- Cedrus_deodara

| | ,--| | ,-- Keteleeria_davidiana

| | | | | ,----- Abies_lasiocarpa

`--| | `--| ,--| ,-- Mataia_podocarpoides

| | | | `--| ,-- Pseudolarix_amabilis

| | | | `--| ,-- Tsuga_canadensis

| | `--| `----- Nothotsuga_longibracteata

| | | ,-- Pseudotsuga_menziesii

| | | ,----- Larix_decidua

| | `--| ,-- Cathaya_argyrophylla

`--|	`--| ,-- Pinus_contorta

| `--| ,-- Eathiestrobus_mackenziei

| `----- Picea_abies

| ,-- **New_total_plant_from_Arkansas**

| ,----- Alvinia_bohemica_plant

| ,--| ,-- Himeriella_muensteri

| | `--| ,-- Kachaikestrobus_acuminatus

| | `----- Tomaxellia_biforme

| | ,-- Pararaucaria_patagonica

`--| |	,-- Sciadopitys_verticillata

| | ,--| ,-- Taxus_wallichiana

| | | `--| ,-- Cunninghamia_lanceolata

| | | `--| ,-- Taiwania_cryptomerioides

`--| |	`--| ,-- Athrotaxis_selaginoides

| | `--| ,-- Sequoia_sempervirens

| | `--| ,-- Cryptomeria_japonica

| | `--| ,-- Cupressus_funebris

`--|	`----- Callitris_muelleri

| ,-- Araucaria_mirabilis

| ,----- Araucaria_heterophylla

| ,--| ,-- Wollemia_nobilis

| | `----- Agathis_australis

| | ,-- Lagarostrobos_franklinii

`--|	,----- Prumnopitys_taxifolia

| ,--| ,-- Lepidothamnus_laxifolius

| | `----- Phyllocladus_aspleniifolius

`--| ,-- Saxegothaea_conspicua

`--| ,-- Microcachrys_tetragona

`--| ,-- Pherosphaera_hookeriana

`--| ,-- Acmopyle_pancheri

`--| ,-- Dacrydium_cupressinum

`--| ,-- Retrophyllum_rospigliosii

`----- Podocarpus_archboldii

Tree 63:

,-- Ginkgo_biloba

| ,-- Cordaixylon_dumusum

|--| | ,-- Emporia_lockardii

`--| ,----- Hanskerpia_hamiltonensis

| | ,-- Voltzia_hexagona

`--| |	,-- Nothodacrium_warrenii

| | ,----- Rissikia_media

`--| | ,-- Telemachus_aequatus

| | | ,-- Schizolepidopsis_ediae

| | | ,--| ,-- Schizolepidopsis_daohugouensis

`--| |	| `----- Schizolepidopsis_canicularis

| | | ,-- Cedrus_deodara

| | ,--| | ,-- Mataia_podocarpoides

| | | | | ,--| ,-- Keteleeria_davidiana

`--| | | |	| `----- Abies_lasiocarpa

| | `--| ,--| ,-- Pseudolarix_amabilis

| | | | `--| ,-- Tsuga_canadensis

| | | | `----- Nothotsuga_longibracteata

| | `--| ,-- Pseudotsuga_menziesii

| | | ,----- Larix_decidua

| | `--| ,-- Cathaya_argyrophylla

`--|	`--| ,-- Pinus_contorta

| `--| ,-- Eathiestrobus_mackenziei

| `----- Picea_abies

| ,-- **New_total_plant_from_Arkansas**

| ,----- Alvinia_bohemica_plant

| ,--| ,-- Himeriella_muensteri

| | `--| ,-- Kachaikestrobus_acuminatus

| | `----- Tomaxellia_biforme

| | ,-- Pararaucaria_patagonica

`--| |	,-- Sciadopitys_verticillata

| | ,--| ,-- Taxus_wallichiana

| | | `--| ,-- Cunninghamia_lanceolata

| | | `--| ,-- Taiwania_cryptomerioides

`--| |	`--| ,-- Athrotaxis_selaginoides

| | `--| ,-- Sequoia_sempervirens

| | `--| ,-- Cryptomeria_japonica

| | `--| ,-- Cupressus_funebris

`--|	`----- Callitris_muelleri

| ,-- Araucaria_mirabilis

| ,----- Araucaria_heterophylla

| ,--| ,-- Wollemia_nobilis

| | `----- Agathis_australis

| | ,-- Lagarostrobos_franklinii

`--|	,----- Prumnopitys_taxifolia

| ,--| ,-- Lepidothamnus_laxifolius

| | `----- Phyllocladus_aspleniifolius

`--| ,-- Saxegothaea_conspicua

`--| ,-- Microcachrys_tetragona

`--| ,-- Pherosphaera_hookeriana

`--| ,-- Acmopyle_pancheri

`--| ,-- Dacrydium_cupressinum

`--| ,-- Retrophyllum_rospigliosii

`----- Podocarpus_archboldii

Tree 64:

,-- Ginkgo_biloba

| ,-- Cordaixylon_dumusum

|--| | ,-- Emporia_lockardii

| | ,----- Hanskerpia_hamiltonensis

`--| | ,-- Voltzia_hexagona

| | | ,-- Nothodacrium_warrenii

`--| | ,----- Rissikia_media

| | | ,-- Pararaucaria_patagonica

`--| |	,--| ,-- **New_total_plant_from_Arkansas**

| | | `----- Alvinia_bohemica_plant

| | ,--| ,-- Himeriella_muensteri

`--| | `--| ,-- Kachaikestrobus_acuminatus

| | `----- Tomaxellia_biforme

| | ,-- Telemachus_aequatus

| | | ,-- Eathiestrobus_mackenziei

`--| |	| ,-- Cedrus_deodara

| | ,--| | ,-- Keteleeria_davidiana

| | | | | ,----- Abies_lasiocarpa

| | | `--| ,--| ,-- Mataia_podocarpoides

| | | | | `--| ,-- Pseudolarix_amabilis

`--| |	| |	`--| ,-- Tsuga_canadensis

| | `--| `----- Nothotsuga_longibracteata

| | | ,-- Pseudotsuga_menziesii

| | | ,----- Larix_decidua

| | `--| ,-- Cathaya_argyrophylla

| | `--| ,-- Pinus_contorta

| | `--| ,-- Picea_abies

`--|	`--| ,-- Schizolepidopsis_ediae

| `--| ,-- Schizolepidopsis_daohugouensis

| `----- Schizolepidopsis_canicularis

| ,-- Sciadopitys_verticillata

| ,--| ,-- Taxus_wallichiana

| | `--| ,-- Cunninghamia_lanceolata

| | `--| ,-- Taiwania_cryptomerioides

| | `--| ,-- Athrotaxis_selaginoides

| | `--| ,-- Sequoia_sempervirens

| | `--| ,-- Cryptomeria_japonica

| | `--| ,-- Cupressus_funebris

`--|	`----- Callitris_muelleri

| ,-- Araucaria_mirabilis

| ,----- Araucaria_heterophylla

| ,--| ,-- Wollemia_nobilis

| | `----- Agathis_australis

| | ,-- Lagarostrobos_franklinii

`--|	,----- Prumnopitys_taxifolia

| ,--| ,-- Lepidothamnus_laxifolius

| | `----- Phyllocladus_aspleniifolius

`--| ,-- Saxegothaea_conspicua

`--| ,-- Microcachrys_tetragona

`--| ,-- Pherosphaera_hookeriana

`--| ,-- Acmopyle_pancheri

`--| ,-- Dacrydium_cupressinum

`--| ,-- Retrophyllum_rospigliosii

`----- Podocarpus_archboldii

Tree 65:

,-- Ginkgo_biloba

| ,-- Cordaixylon_dumusum

|--| | ,-- Emporia_lockardii

| | ,----- Hanskerpia_hamiltonensis

`--| | ,-- Voltzia_hexagona

| | | ,-- Nothodacrium_warrenii

`--| | ,----- Rissikia_media

| | | ,-- Pararaucaria_patagonica

`--| |	,--| ,-- **New_total_plant_from_Arkansas**

| | | `----- Alvinia_bohemica_plant

| | ,--| ,-- Himeriella_muensteri

`--| | `--| ,-- Kachaikestrobus_acuminatus

| | `----- Tomaxellia_biforme

| | ,-- Telemachus_aequatus

| | | ,-- Eathiestrobus_mackenziei

| | | | ,-- Cedrus_deodara

`--| |	| |	,-- Keteleeria_davidiana

| | ,--| | ,--| ,-- Mataia_podocarpoides

| | | | | | `----- Abies_lasiocarpa

| | | `--| ,--| ,-- Pseudolarix_amabilis

| | | | | `--| ,-- Tsuga_canadensis

`--| |	| |	`----- Nothotsuga_longibracteata

| | `--| ,-- Pseudotsuga_menziesii

| | | ,----- Larix_decidua

| | `--| ,-- Cathaya_argyrophylla

| | `--| ,-- Pinus_contorta

| | `--| ,-- Picea_abies

| | `--| ,-- Schizolepidopsis_ediae

`--|	`--| ,-- Schizolepidopsis_daohugouensis

| `----- Schizolepidopsis_canicularis

| ,-- Sciadopitys_verticillata

| ,--| ,-- Taxus_wallichiana

| | `--| ,-- Cunninghamia_lanceolata

| | `--| ,-- Taiwania_cryptomerioides

| | `--| ,-- Athrotaxis_selaginoides

| | `--| ,-- Sequoia_sempervirens

| | `--| ,-- Cryptomeria_japonica

| | `--| ,-- Cupressus_funebris

`--|	`----- Callitris_muelleri

| ,-- Araucaria_mirabilis

| ,----- Araucaria_heterophylla

| ,--| ,-- Wollemia_nobilis

| | `----- Agathis_australis

| | ,-- Lagarostrobos_franklinii

`--|	,----- Prumnopitys_taxifolia

| ,--| ,-- Lepidothamnus_laxifolius

| | `----- Phyllocladus_aspleniifolius

`--| ,-- Saxegothaea_conspicua

`--| ,-- Microcachrys_tetragona

`--| ,-- Pherosphaera_hookeriana

`--| ,-- Acmopyle_pancheri

`--| ,-- Dacrydium_cupressinum

`--| ,-- Retrophyllum_rospigliosii

`----- Podocarpus_archboldii

Tree 66:

,-- Ginkgo_biloba

| ,-- Cordaixylon_dumusum

|--| | ,-- Emporia_lockardii

| | ,----- Hanskerpia_hamiltonensis

`--| | ,-- Voltzia_hexagona

| | | ,-- Nothodacrium_warrenii

`--| | ,----- Rissikia_media

| | | ,-- Pararaucaria_patagonica

`--| |	,--| ,-- **New_total_plant_from_Arkansas**

| | | `----- Alvinia_bohemica_plant

| | ,--| ,-- Himeriella_muensteri

`--| | `--| ,-- Kachaikestrobus_acuminatus

| | `----- Tomaxellia_biforme

| | ,-- Telemachus_aequatus

| | | ,-- Eathiestrobus_mackenziei

| | | | ,-- Cedrus_deodara

`--| |	| |	,-- Keteleeria_davidiana

| | ,--| | ,----- Abies_lasiocarpa

| | | | | | ,-- Mataia_podocarpoides

| | | `--| ,--| ,----- Pseudolarix_amabilis

| | | | | `--| ,-- Tsuga_canadensis

`--| |	| |	`----- Nothotsuga_longibracteata

| | `--| ,-- Pseudotsuga_menziesii

| | | ,----- Larix_decidua

| | `--| ,-- Cathaya_argyrophylla

| | `--| ,-- Pinus_contorta

| | `--| ,-- Picea_abies

| | `--| ,-- Schizolepidopsis_ediae

`--|	`--| ,-- Schizolepidopsis_daohugouensis

| `----- Schizolepidopsis_canicularis

| ,-- Sciadopitys_verticillata

| ,--| ,-- Taxus_wallichiana

| | `--| ,-- Cunninghamia_lanceolata

| | `--| ,-- Taiwania_cryptomerioides

| | `--| ,-- Athrotaxis_selaginoides

| | `--| ,-- Sequoia_sempervirens

| | `--| ,-- Cryptomeria_japonica

| | `--| ,-- Cupressus_funebris

`--|	`----- Callitris_muelleri

| ,-- Araucaria_mirabilis

| ,----- Araucaria_heterophylla

| ,--| ,-- Wollemia_nobilis

| | `----- Agathis_australis

| | ,-- Lagarostrobos_franklinii

`--|	,----- Prumnopitys_taxifolia

| ,--| ,-- Lepidothamnus_laxifolius

| | `----- Phyllocladus_aspleniifolius

`--| ,-- Saxegothaea_conspicua

`--| ,-- Microcachrys_tetragona

`--| ,-- Pherosphaera_hookeriana

`--| ,-- Acmopyle_pancheri

`--| ,-- Dacrydium_cupressinum

`--| ,-- Retrophyllum_rospigliosii

`----- Podocarpus_archboldii

Tree 67:

,-- Ginkgo_biloba

| ,-- Cordaixylon_dumusum

|--| | ,-- Emporia_lockardii

| | ,----- Hanskerpia_hamiltonensis

`--| | ,-- Voltzia_hexagona

| | | ,-- Nothodacrium_warrenii

`--| | ,----- Rissikia_media

| | | ,-- Pararaucaria_patagonica

`--| |	,--| ,-- **New_total_plant_from_Arkansas**

| | | `----- Alvinia_bohemica_plant

| | ,--| ,-- Himeriella_muensteri

`--| | `--| ,-- Kachaikestrobus_acuminatus

| | `----- Tomaxellia_biforme

| | ,-- Telemachus_aequatus

| | | ,-- Eathiestrobus_mackenziei

`--| |	| ,-- Cedrus_deodara

| | ,--| | ,-- Keteleeria_davidiana

| | | | | ,----- Abies_lasiocarpa

| | | `--| ,--| ,-- Pseudolarix_amabilis

| | | | | `--| ,-- Mataia_podocarpoides

`--| |	| |	`--| ,-- Tsuga_canadensis

| | `--| `----- Nothotsuga_longibracteata

| | | ,-- Pseudotsuga_menziesii

| | | ,----- Larix_decidua

| | `--| ,-- Cathaya_argyrophylla

| | `--| ,-- Pinus_contorta

| | `--| ,-- Picea_abies

`--|	`--| ,-- Schizolepidopsis_ediae

| `--| ,-- Schizolepidopsis_daohugouensis

| `----- Schizolepidopsis_canicularis

| ,-- Sciadopitys_verticillata

| ,--| ,-- Taxus_wallichiana

| | `--| ,-- Cunninghamia_lanceolata

| | `--| ,-- Taiwania_cryptomerioides

| | `--| ,-- Athrotaxis_selaginoides

| | `--| ,-- Sequoia_sempervirens

| | `--| ,-- Cryptomeria_japonica

| | `--| ,-- Cupressus_funebris

`--|	`----- Callitris_muelleri

| ,-- Araucaria_mirabilis

| ,----- Araucaria_heterophylla

| ,--| ,-- Wollemia_nobilis

| | `----- Agathis_australis

| | ,-- Lagarostrobos_franklinii

`--|	,----- Prumnopitys_taxifolia

| ,--| ,-- Lepidothamnus_laxifolius

| | `----- Phyllocladus_aspleniifolius

`--| ,-- Saxegothaea_conspicua

`--| ,-- Microcachrys_tetragona

`--| ,-- Pherosphaera_hookeriana

`--| ,-- Acmopyle_pancheri

`--| ,-- Dacrydium_cupressinum

`--| ,-- Retrophyllum_rospigliosii

`----- Podocarpus_archboldii

Tree 68:

,-- Ginkgo_biloba

| ,-- Cordaixylon_dumusum

|--| | ,-- Emporia_lockardii

| | ,----- Hanskerpia_hamiltonensis

`--| | ,-- Voltzia_hexagona

| | | ,-- Nothodacrium_warrenii

`--| | ,----- Rissikia_media

| | | ,-- Pararaucaria_patagonica

`--| |	,--| ,-- **New_total_plant_from_Arkansas**

| | | `----- Alvinia_bohemica_plant

| | ,--| ,-- Himeriella_muensteri

`--| | `--| ,-- Kachaikestrobus_acuminatus

| | `----- Tomaxellia_biforme

| | ,-- Telemachus_aequatus

| | | ,-- Eathiestrobus_mackenziei

`--| |	| ,-- Cedrus_deodara

| | ,--| | ,-- Keteleeria_davidiana

| | | | | ,----- Abies_lasiocarpa

| | | `--| ,--| ,-- Pseudolarix_amabilis

| | | | | `--| ,-- Tsuga_canadensis

`--| |	| |	`--| ,-- Mataia_podocarpoides

| | `--| `----- Nothotsuga_longibracteata

| | | ,-- Pseudotsuga_menziesii

| | | ,----- Larix_decidua

| | `--| ,-- Cathaya_argyrophylla

| | `--| ,-- Pinus_contorta

| | `--| ,-- Picea_abies

`--|	`--| ,-- Schizolepidopsis_ediae

| `--| ,-- Schizolepidopsis_daohugouensis

| `----- Schizolepidopsis_canicularis

| ,-- Sciadopitys_verticillata

| ,--| ,-- Taxus_wallichiana

| | `--| ,-- Cunninghamia_lanceolata

| | `--| ,-- Taiwania_cryptomerioides

| | `--| ,-- Athrotaxis_selaginoides

| | `--| ,-- Sequoia_sempervirens

| | `--| ,-- Cryptomeria_japonica

| | `--| ,-- Cupressus_funebris

`--|	`----- Callitris_muelleri

| ,-- Araucaria_mirabilis

| ,----- Araucaria_heterophylla

| ,--| ,-- Wollemia_nobilis

| | `----- Agathis_australis

| | ,-- Lagarostrobos_franklinii

`--|	,----- Prumnopitys_taxifolia

| ,--| ,-- Lepidothamnus_laxifolius

| | `----- Phyllocladus_aspleniifolius

`--| ,-- Saxegothaea_conspicua

`--| ,-- Microcachrys_tetragona

`--| ,-- Pherosphaera_hookeriana

`--| ,-- Acmopyle_pancheri

`--| ,-- Dacrydium_cupressinum

`--| ,-- Retrophyllum_rospigliosii

`----- Podocarpus_archboldii

Tree 69:

,-- Ginkgo_biloba

| ,-- Cordaixylon_dumusum

|--| | ,-- Emporia_lockardii

| | ,----- Hanskerpia_hamiltonensis

`--| | ,-- Voltzia_hexagona

| | | ,-- Nothodacrium_warrenii

`--| | ,----- Rissikia_media

| | | ,-- Pararaucaria_patagonica

`--| |	,--| ,-- **New_total_plant_from_Arkansas**

| | | `----- Alvinia_bohemica_plant

| | ,--| ,-- Himeriella_muensteri

`--| | `--| ,-- Kachaikestrobus_acuminatus

| | `----- Tomaxellia_biforme

| | ,-- Telemachus_aequatus

| | | ,-- Eathiestrobus_mackenziei

`--| |	| ,-- Cedrus_deodara

| | ,--| | ,-- Mataia_podocarpoides

| | | | | | ,-- Keteleeria_davidiana

| | | `--| ,--| ,----- Abies_lasiocarpa

| | | | | `--| ,-- Pseudolarix_amabilis

`--| |	| |	`--| ,-- Tsuga_canadensis

| | `--| `----- Nothotsuga_longibracteata

| | | ,-- Pseudotsuga_menziesii

| | | ,----- Larix_decidua

| | `--| ,-- Cathaya_argyrophylla

| | `--| ,-- Pinus_contorta

| | `--| ,-- Picea_abies

`--|	`--| ,-- Schizolepidopsis_ediae

| `--| ,-- Schizolepidopsis_daohugouensis

| `----- Schizolepidopsis_canicularis

| ,-- Sciadopitys_verticillata

| ,--| ,-- Taxus_wallichiana

| | `--| ,-- Cunninghamia_lanceolata

| | `--| ,-- Taiwania_cryptomerioides

| | `--| ,-- Athrotaxis_selaginoides

| | `--| ,-- Sequoia_sempervirens

| | `--| ,-- Cryptomeria_japonica

| | `--| ,-- Cupressus_funebris

`--|	`----- Callitris_muelleri

| ,-- Araucaria_mirabilis

| ,----- Araucaria_heterophylla

| ,--| ,-- Wollemia_nobilis

| | `----- Agathis_australis

| | ,-- Lagarostrobos_franklinii

`--|	,----- Prumnopitys_taxifolia

| ,--| ,-- Lepidothamnus_laxifolius

| | `----- Phyllocladus_aspleniifolius

`--| ,-- Saxegothaea_conspicua

`--| ,-- Microcachrys_tetragona

`--| ,-- Pherosphaera_hookeriana

`--| ,-- Acmopyle_pancheri

`--| ,-- Dacrydium_cupressinum

`--| ,-- Retrophyllum_rospigliosii

`----- Podocarpus_archboldii

Tree 70:

,-- Ginkgo_biloba

| ,-- Cordaixylon_dumusum

|--| | ,-- Emporia_lockardii

`--| | ,-- Hanskerpia_hamiltonensis

`--| | ,-- Voltzia_hexagona

`--| |	,-- Nothodacrium_warrenii

| | ,----- Rissikia_media

`--| | ,-- Telemachus_aequatus

| | | ,-- Schizolepidopsis_ediae

| | | | ,-- Schizolepidopsis_daohugouensis

`--| |	| ,----- Schizolepidopsis_canicularis

| | ,--| | ,-- Cedrus_deodara

| | | | | | ,-- Keteleeria_davidiana

| | | `--| | ,--| ,-- Mataia_podocarpoides

`--| |	| |	| `----- Abies_lasiocarpa

| | `--| ,--| ,-- Pseudolarix_amabilis

| | | | `--| ,-- Tsuga_canadensis

| | | | `----- Nothotsuga_longibracteata

| | `--| ,-- Pseudotsuga_menziesii

| | | ,----- Larix_decidua

| | `--| ,-- Cathaya_argyrophylla

`--|	`--| ,-- Picea_abies

| `--| ,-- Eathiestrobus_mackenziei

| `----- Pinus_contorta

| ,-- **New_total_plant_from_Arkansas**

| ,----- Alvinia_bohemica_plant

| ,--| ,-- Himeriella_muensteri

| | `--| ,-- Kachaikestrobus_acuminatus

| | `----- Tomaxellia_biforme

| | ,-- Pararaucaria_patagonica

`--| |	,-- Sciadopitys_verticillata

| | ,--| ,-- Taxus_wallichiana

| | | `--| ,-- Cunninghamia_lanceolata

| | | `--| ,-- Taiwania_cryptomerioides

`--| |	`--| ,-- Athrotaxis_selaginoides

| | `--| ,-- Sequoia_sempervirens

| | `--| ,-- Cryptomeria_japonica

| | `--| ,-- Cupressus_funebris

`--|	`----- Callitris_muelleri

| ,-- Araucaria_mirabilis

| ,----- Araucaria_heterophylla

| ,--| ,-- Wollemia_nobilis

| | `----- Agathis_australis

| | ,-- Lagarostrobos_franklinii

`--|	,----- Prumnopitys_taxifolia

| ,--| ,-- Lepidothamnus_laxifolius

| | `----- Phyllocladus_aspleniifolius

`--| ,-- Saxegothaea_conspicua

`--| ,-- Microcachrys_tetragona

`--| ,-- Pherosphaera_hookeriana

`--| ,-- Acmopyle_pancheri

`--| ,-- Dacrydium_cupressinum

`--| ,-- Retrophyllum_rospigliosii

`----- Podocarpus_archboldii

Tree 71:

,-- Ginkgo_biloba

| ,-- Cordaixylon_dumusum

|--| | ,-- Emporia_lockardii

`--| | ,-- Hanskerpia_hamiltonensis

`--| | ,-- Voltzia_hexagona

`--| |	,-- Nothodacrium_warrenii

| | ,----- Rissikia_media

`--| | ,-- Telemachus_aequatus

| | | ,-- Schizolepidopsis_ediae

| | | | ,-- Schizolepidopsis_daohugouensis

`--| |	| ,----- Schizolepidopsis_canicularis

| | ,--| | ,-- Cedrus_deodara

| | | | | | ,-- Keteleeria_davidiana

| | | `--| | ,----- Abies_lasiocarpa

`--| |	| |	|	,-- Mataia_podocarpoides

| | `--| ,--| ,----- Pseudolarix_amabilis

| | | | `--| ,-- Tsuga_canadensis

| | | | `----- Nothotsuga_longibracteata

| | `--| ,-- Pseudotsuga_menziesii

| | | ,----- Larix_decidua

| | `--| ,-- Cathaya_argyrophylla

`--|	`--| ,-- Picea_abies

| `--| ,-- Eathiestrobus_mackenziei

| `----- Pinus_contorta

| ,-- **New_total_plant_from_Arkansas**

| ,----- Alvinia_bohemica_plant

| ,--| ,-- Himeriella_muensteri

| | `--| ,-- Kachaikestrobus_acuminatus

| | `----- Tomaxellia_biforme

| | ,-- Pararaucaria_patagonica

`--| |	,-- Sciadopitys_verticillata

| | ,--| ,-- Taxus_wallichiana

| | | `--| ,-- Cunninghamia_lanceolata

| | | `--| ,-- Taiwania_cryptomerioides

`--| |	`--| ,-- Athrotaxis_selaginoides

| | `--| ,-- Sequoia_sempervirens

| | `--| ,-- Cryptomeria_japonica

| | `--| ,-- Cupressus_funebris

`--|	`----- Callitris_muelleri

| ,-- Araucaria_mirabilis

| ,----- Araucaria_heterophylla

| ,--| ,-- Wollemia_nobilis

| | `----- Agathis_australis

| | ,-- Lagarostrobos_franklinii

`--|	,----- Prumnopitys_taxifolia

| ,--| ,-- Lepidothamnus_laxifolius

| | `----- Phyllocladus_aspleniifolius

`--| ,-- Saxegothaea_conspicua

`--| ,-- Microcachrys_tetragona

`--| ,-- Pherosphaera_hookeriana

`--| ,-- Acmopyle_pancheri

`--| ,-- Dacrydium_cupressinum

`--| ,-- Retrophyllum_rospigliosii

`----- Podocarpus_archboldii

Tree 72:

,-- Ginkgo_biloba

| ,-- Cordaixylon_dumusum

|--| | ,-- Emporia_lockardii

`--| | ,-- Hanskerpia_hamiltonensis

`--| | ,-- Voltzia_hexagona

`--| |	,-- Nothodacrium_warrenii

| | ,----- Rissikia_media

`--| | ,-- Telemachus_aequatus

| | | ,-- Schizolepidopsis_ediae

| | | | ,-- Schizolepidopsis_daohugouensis

`--| | ,--| ,----- Schizolepidopsis_canicularis

| | | | | ,-- Cedrus_deodara

| | | `--| | ,-- Keteleeria_davidiana

| | | | | ,----- Abies_lasiocarpa

`--| |	`--| ,--| ,-- Pseudolarix_amabilis

| | | | `--| ,-- Mataia_podocarpoides

| | | | `--| ,-- Tsuga_canadensis

| | `--| `----- Nothotsuga_longibracteata

| | | ,-- Pseudotsuga_menziesii

| | | ,----- Larix_decidua

`--|	`--| ,-- Cathaya_argyrophylla

| `--| ,-- Picea_abies

| `--| ,-- Eathiestrobus_mackenziei

| `----- Pinus_contorta

| ,-- **New_total_plant_from_Arkansas**

| ,----- Alvinia_bohemica_plant

| ,--| ,-- Himeriella_muensteri

| | `--| ,-- Kachaikestrobus_acuminatus

| | `----- Tomaxellia_biforme

| | ,-- Pararaucaria_patagonica

`--| |	,-- Sciadopitys_verticillata

| | ,--| ,-- Taxus_wallichiana

| | | `--| ,-- Cunninghamia_lanceolata

| | | `--| ,-- Taiwania_cryptomerioides

`--| |	`--| ,-- Athrotaxis_selaginoides

| | `--| ,-- Sequoia_sempervirens

| | `--| ,-- Cryptomeria_japonica

| | `--| ,-- Cupressus_funebris

`--|	`----- Callitris_muelleri

| ,-- Araucaria_mirabilis

| ,----- Araucaria_heterophylla

| ,--| ,-- Wollemia_nobilis

| | `----- Agathis_australis

| | ,-- Lagarostrobos_franklinii

`--|	,----- Prumnopitys_taxifolia

| ,--| ,-- Lepidothamnus_laxifolius

| | `----- Phyllocladus_aspleniifolius

`--| ,-- Saxegothaea_conspicua

`--| ,-- Microcachrys_tetragona

`--| ,-- Pherosphaera_hookeriana

`--| ,-- Acmopyle_pancheri

`--| ,-- Dacrydium_cupressinum

`--| ,-- Retrophyllum_rospigliosii

`----- Podocarpus_archboldii

Tree 73:

,-- Ginkgo_biloba

| ,-- Cordaixylon_dumusum

|--| | ,-- Emporia_lockardii

`--| | ,-- Hanskerpia_hamiltonensis

`--| | ,-- Voltzia_hexagona

`--| |	,-- Nothodacrium_warrenii

| | ,----- Rissikia_media

`--| | ,-- Telemachus_aequatus

| | | ,-- Schizolepidopsis_ediae

| | | | ,-- Schizolepidopsis_daohugouensis

`--| | ,--| ,----- Schizolepidopsis_canicularis

| | | | | ,-- Cedrus_deodara

| | | `--| | ,-- Keteleeria_davidiana

| | | | | ,----- Abies_lasiocarpa

`--| |	`--| ,--| ,-- Pseudolarix_amabilis

| | | | `--| ,-- Tsuga_canadensis

| | | | `--| ,-- Mataia_podocarpoides

| | `--| `----- Nothotsuga_longibracteata

| | | ,-- Pseudotsuga_menziesii

| | | ,----- Larix_decidua

`--|	`--| ,-- Cathaya_argyrophylla

| `--| ,-- Picea_abies

| `--| ,-- Eathiestrobus_mackenziei

| `----- Pinus_contorta

| ,-- **New_total_plant_from_Arkansas**

| ,----- Alvinia_bohemica_plant

| ,--| ,-- Himeriella_muensteri

| | `--| ,-- Kachaikestrobus_acuminatus

| | `----- Tomaxellia_biforme

| | ,-- Pararaucaria_patagonica

`--| |	,-- Sciadopitys_verticillata

| | ,--| ,-- Taxus_wallichiana

| | | `--| ,-- Cunninghamia_lanceolata

| | | `--| ,-- Taiwania_cryptomerioides

`--| |	`--| ,-- Athrotaxis_selaginoides

| | `--| ,-- Sequoia_sempervirens

| | `--| ,-- Cryptomeria_japonica

| | `--| ,-- Cupressus_funebris

`--|	`----- Callitris_muelleri

| ,-- Araucaria_mirabilis

| ,----- Araucaria_heterophylla

| ,--| ,-- Wollemia_nobilis

| | `----- Agathis_australis

| | ,-- Lagarostrobos_franklinii

`--|	,----- Prumnopitys_taxifolia

| ,--| ,-- Lepidothamnus_laxifolius

| | `----- Phyllocladus_aspleniifolius

`--| ,-- Saxegothaea_conspicua

`--| ,-- Microcachrys_tetragona

`--| ,-- Pherosphaera_hookeriana

`--| ,-- Acmopyle_pancheri

`--| ,-- Dacrydium_cupressinum

`--| ,-- Retrophyllum_rospigliosii

`----- Podocarpus_archboldii

Tree 74:

,-- Ginkgo_biloba

| ,-- Cordaixylon_dumusum

|--| | ,-- Emporia_lockardii

`--| | ,-- Hanskerpia_hamiltonensis

`--| | ,-- Voltzia_hexagona

`--| |	,-- Nothodacrium_warrenii

| | ,----- Rissikia_media

`--| | ,-- Telemachus_aequatus

| | | ,-- Schizolepidopsis_ediae

| | | | ,-- Schizolepidopsis_daohugouensis

`--| | ,--| ,----- Schizolepidopsis_canicularis

| | | | | ,-- Cedrus_deodara

| | | `--| | ,-- Keteleeria_davidiana

| | | | | ,----- Abies_lasiocarpa

`--| |	`--| ,--| ,-- Mataia_podocarpoides

| | | | `--| ,-- Pseudolarix_amabilis

| | | | `--| ,-- Tsuga_canadensis

| | `--| `----- Nothotsuga_longibracteata

| | | ,-- Pseudotsuga_menziesii

| | | ,----- Larix_decidua

`--|	`--| ,-- Cathaya_argyrophylla

| `--| ,-- Picea_abies

| `--| ,-- Eathiestrobus_mackenziei

| `----- Pinus_contorta

| ,-- **New_total_plant_from_Arkansas**

| ,----- Alvinia_bohemica_plant

| ,--| ,-- Himeriella_muensteri

| | `--| ,-- Kachaikestrobus_acuminatus

| | `----- Tomaxellia_biforme

| | ,-- Pararaucaria_patagonica

`--| |	,-- Sciadopitys_verticillata

| | ,--| ,-- Taxus_wallichiana

| | | `--| ,-- Cunninghamia_lanceolata

| | | `--| ,-- Taiwania_cryptomerioides

`--| |	`--| ,-- Athrotaxis_selaginoides

| | `--| ,-- Sequoia_sempervirens

| | `--| ,-- Cryptomeria_japonica

| | `--| ,-- Cupressus_funebris

`--|	`----- Callitris_muelleri

| ,-- Araucaria_mirabilis

| ,----- Araucaria_heterophylla

| ,--| ,-- Wollemia_nobilis

| | `----- Agathis_australis

| | ,-- Lagarostrobos_franklinii

`--|	,----- Prumnopitys_taxifolia

| ,--| ,-- Lepidothamnus_laxifolius

| | `----- Phyllocladus_aspleniifolius

`--| ,-- Saxegothaea_conspicua

`--| ,-- Microcachrys_tetragona

`--| ,-- Pherosphaera_hookeriana

`--| ,-- Acmopyle_pancheri

`--| ,-- Dacrydium_cupressinum

`--| ,-- Retrophyllum_rospigliosii

`----- Podocarpus_archboldii

Tree 75:

,-- Ginkgo_biloba

| ,-- Cordaixylon_dumusum

|--| | ,-- Emporia_lockardii

`--| | ,-- Hanskerpia_hamiltonensis

`--| | ,-- Voltzia_hexagona

`--| |	,-- Nothodacrium_warrenii

| | ,----- Rissikia_media

`--| | ,-- Telemachus_aequatus

| | | ,-- Schizolepidopsis_ediae

| | | | ,-- Schizolepidopsis_daohugouensis

`--| |	| ,----- Schizolepidopsis_canicularis

| | ,--| | ,-- Cedrus_deodara

| | | | | | ,-- Mataia_podocarpoides

| | | `--| | ,--| ,-- Keteleeria_davidiana

`--| |	| |	| `----- Abies_lasiocarpa

| | `--| ,--| ,-- Pseudolarix_amabilis

| | | | `--| ,-- Tsuga_canadensis

| | | | `----- Nothotsuga_longibracteata

| | `--| ,-- Pseudotsuga_menziesii

| | | ,----- Larix_decidua

| | `--| ,-- Cathaya_argyrophylla

`--|	`--| ,-- Picea_abies

| `--| ,-- Eathiestrobus_mackenziei

| `----- Pinus_contorta

| ,-- **New_total_plant_from_Arkansas**

| ,----- Alvinia_bohemica_plant

| ,--| ,-- Himeriella_muensteri

| | `--| ,-- Kachaikestrobus_acuminatus

| | `----- Tomaxellia_biforme

| | ,-- Pararaucaria_patagonica

`--| |	,-- Sciadopitys_verticillata

| | ,--| ,-- Taxus_wallichiana

| | | `--| ,-- Cunninghamia_lanceolata

| | | `--| ,-- Taiwania_cryptomerioides

`--| |	`--| ,-- Athrotaxis_selaginoides

| | `--| ,-- Sequoia_sempervirens

| | `--| ,-- Cryptomeria_japonica

| | `--| ,-- Cupressus_funebris

`--|	`----- Callitris_muelleri

| ,-- Araucaria_mirabilis

| ,----- Araucaria_heterophylla

| ,--| ,-- Wollemia_nobilis

| | `----- Agathis_australis

| | ,-- Lagarostrobos_franklinii

`--|	,----- Prumnopitys_taxifolia

| ,--| ,-- Lepidothamnus_laxifolius

| | `----- Phyllocladus_aspleniifolius

`--| ,-- Saxegothaea_conspicua

`--| ,-- Microcachrys_tetragona

`--| ,-- Pherosphaera_hookeriana

`--| ,-- Acmopyle_pancheri

`--| ,-- Dacrydium_cupressinum

`--| ,-- Retrophyllum_rospigliosii

`----- Podocarpus_archboldii

Tree 76:

,-- Ginkgo_biloba

| ,-- Cordaixylon_dumusum

|--| | ,-- Emporia_lockardii

`--| | ,-- Hanskerpia_hamiltonensis

`--| | ,-- Voltzia_hexagona

`--| |	,-- Nothodacrium_warrenii

| | ,----- Rissikia_media

`--| | ,-- Telemachus_aequatus

| | | ,-- Schizolepidopsis_ediae

| | | ,--| ,-- Schizolepidopsis_daohugouensis

`--| |	| `----- Schizolepidopsis_canicularis

| | | ,-- Cedrus_deodara

| | ,--| | ,-- Keteleeria_davidiana

| | | | | ,--| ,-- Mataia_podocarpoides

`--| | | |	| `----- Abies_lasiocarpa

| | `--| ,--| ,-- Pseudolarix_amabilis

| | | | `--| ,-- Tsuga_canadensis

| | | | `----- Nothotsuga_longibracteata

| | `--| ,-- Pseudotsuga_menziesii

| | | ,----- Larix_decidua

| | `--| ,-- Cathaya_argyrophylla

`--|	`--| ,-- Pinus_contorta

| `--| ,-- Eathiestrobus_mackenziei

| `----- Picea_abies

| ,-- **New_total_plant_from_Arkansas**

| ,----- Alvinia_bohemica_plant

| ,--| ,-- Himeriella_muensteri

| | `--| ,-- Kachaikestrobus_acuminatus

| | `----- Tomaxellia_biforme

| | ,-- Pararaucaria_patagonica

`--| |	,-- Sciadopitys_verticillata

| | ,--| ,-- Taxus_wallichiana

| | | `--| ,-- Cunninghamia_lanceolata

| | | `--| ,-- Taiwania_cryptomerioides

`--| |	`--| ,-- Athrotaxis_selaginoides

| | `--| ,-- Sequoia_sempervirens

| | `--| ,-- Cryptomeria_japonica

| | `--| ,-- Cupressus_funebris

`--|	`----- Callitris_muelleri

| ,-- Araucaria_mirabilis

| ,----- Araucaria_heterophylla

| ,--| ,-- Wollemia_nobilis

| | `----- Agathis_australis

| | ,-- Lagarostrobos_franklinii

`--|	,----- Prumnopitys_taxifolia

| ,--| ,-- Lepidothamnus_laxifolius

| | `----- Phyllocladus_aspleniifolius

`--| ,-- Saxegothaea_conspicua

`--| ,-- Microcachrys_tetragona

`--| ,-- Pherosphaera_hookeriana

`--| ,-- Acmopyle_pancheri

`--| ,-- Dacrydium_cupressinum

`--| ,-- Retrophyllum_rospigliosii

`----- Podocarpus_archboldii

Tree 77:

,-- Ginkgo_biloba

| ,-- Cordaixylon_dumusum

|--| | ,-- Emporia_lockardii

`--| | ,-- Hanskerpia_hamiltonensis

`--| | ,-- Voltzia_hexagona

`--| |	,-- Nothodacrium_warrenii

| | ,----- Rissikia_media

`--| | ,-- Telemachus_aequatus

| | | ,-- Schizolepidopsis_ediae

| | | ,--| ,-- Schizolepidopsis_daohugouensis

`--| |	| `----- Schizolepidopsis_canicularis

| | | ,-- Cedrus_deodara

| | ,--| | ,-- Keteleeria_davidiana

| | | | | ,----- Abies_lasiocarpa

`--| | | |	|	,-- Mataia_podocarpoides

| | `--| ,--| ,----- Pseudolarix_amabilis

| | | | `--| ,-- Tsuga_canadensis

| | | | `----- Nothotsuga_longibracteata

| | `--| ,-- Pseudotsuga_menziesii

| | | ,----- Larix_decidua

| | `--| ,-- Cathaya_argyrophylla

`--|	`--| ,-- Pinus_contorta

| `--| ,-- Eathiestrobus_mackenziei

| `----- Picea_abies

| ,-- **New_total_plant_from_Arkansas**

| ,----- Alvinia_bohemica_plant

| ,--| ,-- Himeriella_muensteri

| | `--| ,-- Kachaikestrobus_acuminatus

| | `----- Tomaxellia_biforme

| | ,-- Pararaucaria_patagonica

`--| |	,-- Sciadopitys_verticillata

| | ,--| ,-- Taxus_wallichiana

| | | `--| ,-- Cunninghamia_lanceolata

| | | `--| ,-- Taiwania_cryptomerioides

`--| |	`--| ,-- Athrotaxis_selaginoides

| | `--| ,-- Sequoia_sempervirens

| | `--| ,-- Cryptomeria_japonica

| | `--| ,-- Cupressus_funebris

`--|	`----- Callitris_muelleri

| ,-- Araucaria_mirabilis

| ,----- Araucaria_heterophylla

| ,--| ,-- Wollemia_nobilis

| | `----- Agathis_australis

| | ,-- Lagarostrobos_franklinii

`--|	,----- Prumnopitys_taxifolia

| ,--| ,-- Lepidothamnus_laxifolius

| | `----- Phyllocladus_aspleniifolius

`--| ,-- Saxegothaea_conspicua

`--| ,-- Microcachrys_tetragona

`--| ,-- Pherosphaera_hookeriana

`--| ,-- Acmopyle_pancheri

`--| ,-- Dacrydium_cupressinum

`--| ,-- Retrophyllum_rospigliosii

`----- Podocarpus_archboldii

Tree 78:

,-- Ginkgo_biloba

| ,-- Cordaixylon_dumusum

|--| | ,-- Emporia_lockardii

`--| | ,-- Hanskerpia_hamiltonensis

`--| | ,-- Voltzia_hexagona

`--| |	,-- Nothodacrium_warrenii

| | ,----- Rissikia_media

`--| | ,-- Telemachus_aequatus

| | | ,-- Schizolepidopsis_ediae

| | | ,--| ,-- Schizolepidopsis_daohugouensis

`--| |	| `----- Schizolepidopsis_canicularis

| | | ,-- Cedrus_deodara

| | ,--| | ,-- Keteleeria_davidiana

| | | | | ,----- Abies_lasiocarpa

`--| | `--| ,--| ,-- Pseudolarix_amabilis

| | | | `--| ,-- Mataia_podocarpoides

| | | | `--| ,-- Tsuga_canadensis

| | `--| `----- Nothotsuga_longibracteata

| | | ,-- Pseudotsuga_menziesii

| | | ,----- Larix_decidua

| | `--| ,-- Cathaya_argyrophylla

`--|	`--| ,-- Pinus_contorta

| `--| ,-- Eathiestrobus_mackenziei

| `----- Picea_abies

| ,-- **New_total_plant_from_Arkansas**

| ,----- Alvinia_bohemica_plant

| ,--| ,-- Himeriella_muensteri

| | `--| ,-- Kachaikestrobus_acuminatus

| | `----- Tomaxellia_biforme

| | ,-- Pararaucaria_patagonica

`--| |	,-- Sciadopitys_verticillata

| | ,--| ,-- Taxus_wallichiana

| | | `--| ,-- Cunninghamia_lanceolata

| | | `--| ,-- Taiwania_cryptomerioides

`--| |	`--| ,-- Athrotaxis_selaginoides

| | `--| ,-- Sequoia_sempervirens

| | `--| ,-- Cryptomeria_japonica

| | `--| ,-- Cupressus_funebris

`--|	`----- Callitris_muelleri

| ,-- Araucaria_mirabilis

| ,----- Araucaria_heterophylla

| ,--| ,-- Wollemia_nobilis

| | `----- Agathis_australis

| | ,-- Lagarostrobos_franklinii

`--|	,----- Prumnopitys_taxifolia

| ,--| ,-- Lepidothamnus_laxifolius

| | `----- Phyllocladus_aspleniifolius

`--| ,-- Saxegothaea_conspicua

`--| ,-- Microcachrys_tetragona

`--| ,-- Pherosphaera_hookeriana

`--| ,-- Acmopyle_pancheri

`--| ,-- Dacrydium_cupressinum

`--| ,-- Retrophyllum_rospigliosii

`----- Podocarpus_archboldii

Tree 79:

,-- Ginkgo_biloba

| ,-- Cordaixylon_dumusum

|--| | ,-- Emporia_lockardii

`--| | ,-- Hanskerpia_hamiltonensis

`--| | ,-- Voltzia_hexagona

`--| |	,-- Nothodacrium_warrenii

| | ,----- Rissikia_media

`--| | ,-- Telemachus_aequatus

| | | ,-- Schizolepidopsis_ediae

| | | ,--| ,-- Schizolepidopsis_daohugouensis

`--| |	| `----- Schizolepidopsis_canicularis

| | | ,-- Cedrus_deodara

| | ,--| | ,-- Keteleeria_davidiana

| | | | | ,----- Abies_lasiocarpa

`--| | `--| ,--| ,-- Pseudolarix_amabilis

| | | | `--| ,-- Tsuga_canadensis

| | | | `--| ,-- Mataia_podocarpoides

| | `--| `----- Nothotsuga_longibracteata

| | | ,-- Pseudotsuga_menziesii

| | | ,----- Larix_decidua

| | `--| ,-- Cathaya_argyrophylla

`--|	`--| ,-- Pinus_contorta

| `--| ,-- Eathiestrobus_mackenziei

| `----- Picea_abies

| ,-- **New_total_plant_from_Arkansas**

| ,----- Alvinia_bohemica_plant

| ,--| ,-- Himeriella_muensteri

| | `--| ,-- Kachaikestrobus_acuminatus

| | `----- Tomaxellia_biforme

| | ,-- Pararaucaria_patagonica

`--| |	,-- Sciadopitys_verticillata

| | ,--| ,-- Taxus_wallichiana

| | | `--| ,-- Cunninghamia_lanceolata

| | | `--| ,-- Taiwania_cryptomerioides

`--| |	`--| ,-- Athrotaxis_selaginoides

| | `--| ,-- Sequoia_sempervirens

| | `--| ,-- Cryptomeria_japonica

| | `--| ,-- Cupressus_funebris

`--|	`----- Callitris_muelleri

| ,-- Araucaria_mirabilis

| ,----- Araucaria_heterophylla

| ,--| ,-- Wollemia_nobilis

| | `----- Agathis_australis

| | ,-- Lagarostrobos_franklinii

`--|	,----- Prumnopitys_taxifolia

| ,--| ,-- Lepidothamnus_laxifolius

| | `----- Phyllocladus_aspleniifolius

`--| ,-- Saxegothaea_conspicua

`--| ,-- Microcachrys_tetragona

`--| ,-- Pherosphaera_hookeriana

`--| ,-- Acmopyle_pancheri

`--| ,-- Dacrydium_cupressinum

`--| ,-- Retrophyllum_rospigliosii

`----- Podocarpus_archboldii

Tree 80:

,-- Ginkgo_biloba

| ,-- Cordaixylon_dumusum

|--| | ,-- Emporia_lockardii

`--| | ,-- Hanskerpia_hamiltonensis

`--| | ,-- Voltzia_hexagona

`--| |	,-- Nothodacrium_warrenii

| | ,----- Rissikia_media

`--| | ,-- Telemachus_aequatus

| | | ,-- Schizolepidopsis_ediae

| | | ,--| ,-- Schizolepidopsis_daohugouensis

`--| |	| `----- Schizolepidopsis_canicularis

| | | ,-- Cedrus_deodara

| | ,--| | ,-- Keteleeria_davidiana

| | | | | ,----- Abies_lasiocarpa

`--| | `--| ,--| ,-- Mataia_podocarpoides

| | | | `--| ,-- Pseudolarix_amabilis

| | | | `--| ,-- Tsuga_canadensis

| | `--| `----- Nothotsuga_longibracteata

| | | ,-- Pseudotsuga_menziesii

| | | ,----- Larix_decidua

| | `--| ,-- Cathaya_argyrophylla

`--|	`--| ,-- Pinus_contorta

| `--| ,-- Eathiestrobus_mackenziei

| `----- Picea_abies

| ,-- **New_total_plant_from_Arkansas**

| ,----- Alvinia_bohemica_plant

| ,--| ,-- Himeriella_muensteri

| | `--| ,-- Kachaikestrobus_acuminatus

| | `----- Tomaxellia_biforme

| | ,-- Pararaucaria_patagonica

`--| |	,-- Sciadopitys_verticillata

| | ,--| ,-- Taxus_wallichiana

| | | `--| ,-- Cunninghamia_lanceolata

| | | `--| ,-- Taiwania_cryptomerioides

`--| |	`--| ,-- Athrotaxis_selaginoides

| | `--| ,-- Sequoia_sempervirens

| | `--| ,-- Cryptomeria_japonica

| | `--| ,-- Cupressus_funebris

`--|	`----- Callitris_muelleri

| ,-- Araucaria_mirabilis

| ,----- Araucaria_heterophylla

| ,--| ,-- Wollemia_nobilis

| | `----- Agathis_australis

| | ,-- Lagarostrobos_franklinii

`--|	,----- Prumnopitys_taxifolia

| ,--| ,-- Lepidothamnus_laxifolius

| | `----- Phyllocladus_aspleniifolius

`--| ,-- Saxegothaea_conspicua

`--| ,-- Microcachrys_tetragona

`--| ,-- Pherosphaera_hookeriana

`--| ,-- Acmopyle_pancheri

`--| ,-- Dacrydium_cupressinum

`--| ,-- Retrophyllum_rospigliosii

`----- Podocarpus_archboldii

Tree 81:

,-- Ginkgo_biloba

| ,-- Cordaixylon_dumusum

|--| | ,-- Emporia_lockardii

`--| | ,-- Hanskerpia_hamiltonensis

`--| | ,-- Voltzia_hexagona

`--| |	,-- Nothodacrium_warrenii

| | ,----- Rissikia_media

`--| | ,-- Telemachus_aequatus

| | | ,-- Schizolepidopsis_ediae

| | | ,--| ,-- Schizolepidopsis_daohugouensis

`--| |	| `----- Schizolepidopsis_canicularis

| | | ,-- Cedrus_deodara

| | ,--| | ,-- Mataia_podocarpoides

| | | | | ,--| ,-- Keteleeria_davidiana

`--| | | |	| `----- Abies_lasiocarpa

| | `--| ,--| ,-- Pseudolarix_amabilis

| | | | `--| ,-- Tsuga_canadensis

| | | | `----- Nothotsuga_longibracteata

| | `--| ,-- Pseudotsuga_menziesii

| | | ,----- Larix_decidua

| | `--| ,-- Cathaya_argyrophylla

`--|	`--| ,-- Pinus_contorta

| `--| ,-- Eathiestrobus_mackenziei

| `----- Picea_abies

| ,-- **New_total_plant_from_Arkansas**

| ,----- Alvinia_bohemica_plant

| ,--| ,-- Himeriella_muensteri

| | `--| ,-- Kachaikestrobus_acuminatus

| | `----- Tomaxellia_biforme

| | ,-- Pararaucaria_patagonica

`--| |	,-- Sciadopitys_verticillata

| | ,--| ,-- Taxus_wallichiana

| | | `--| ,-- Cunninghamia_lanceolata

| | | `--| ,-- Taiwania_cryptomerioides

`--| |	`--| ,-- Athrotaxis_selaginoides

| | `--| ,-- Sequoia_sempervirens

| | `--| ,-- Cryptomeria_japonica

| | `--| ,-- Cupressus_funebris

`--|	`----- Callitris_muelleri

| ,-- Araucaria_mirabilis

| ,----- Araucaria_heterophylla

| ,--| ,-- Wollemia_nobilis

| | `----- Agathis_australis

| | ,-- Lagarostrobos_franklinii

`--|	,----- Prumnopitys_taxifolia

| ,--| ,-- Lepidothamnus_laxifolius

| | `----- Phyllocladus_aspleniifolius

`--| ,-- Saxegothaea_conspicua

`--| ,-- Microcachrys_tetragona

`--| ,-- Pherosphaera_hookeriana

`--| ,-- Acmopyle_pancheri

`--| ,-- Dacrydium_cupressinum

`--| ,-- Retrophyllum_rospigliosii

`----- Podocarpus_archboldii
