# Supplementary material for: Revisiting the enigmatic Cheirolepidiaceae: origins, phylogenetic relationships, and a new whole-plant concept
Source: Ann Bot. 2026 Mar 30;137(6):1876–901. doi: 10.1093/aob/mcag069 (PMC13275020; doi:10.1093/aob/mcag069)
Supplement: mcag069_Supplementary_Data [file mcag069_supplementary_data.zip › AppendixS3-Bayesian.zip!/AppendixS3-Bayesian/Appendix3A-FigureBayesian.pdf]

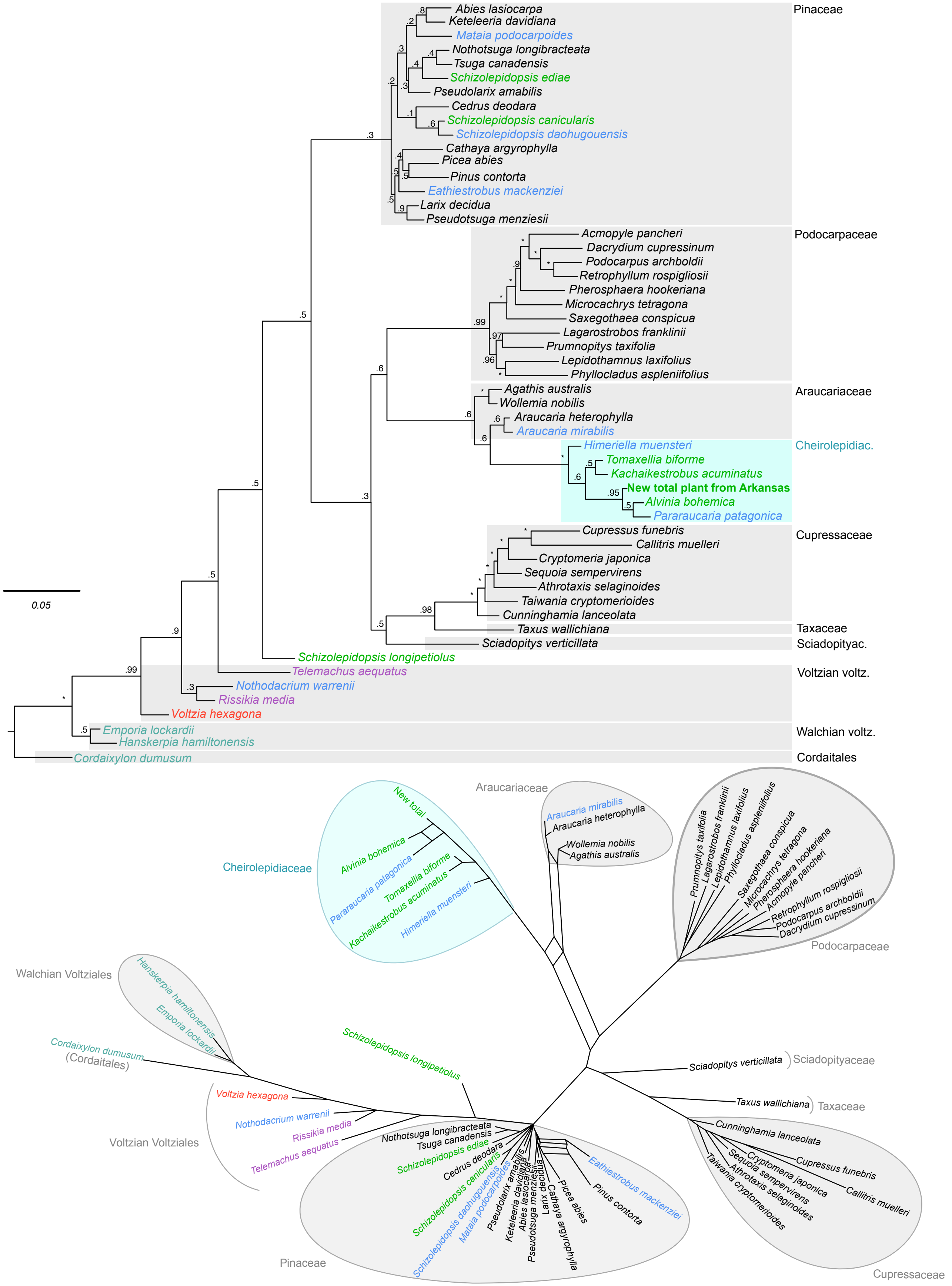
Pinaceae
Abies lasiocarpa
Keteleeria davidiana
Mataia podocarpoides
Nothotsuga longibracteata
Tsuga canadensis
Schizolepidopsis ediae
Pseudolarix amabilis
Cedrus deodara
Schizolepidopsis canicularis
Schizolepidopsis daohugouensis
Cathaya argyrophylla
Picea abies
Pinus contorta
Eathiestrobus mackenziei
Larix decidua
Pseudotsuga menziesii
Podocarpaceae
Acmopyle pancheri
Dacrydium cupressinum
Podocarpus archboldii
Retrophyllum rospigliosii
Pherosphaera hookeriana
Microcachrys tetragona
Saxegothaea conspicua
Lagarostrobos franklinii
Prumnopitys taxifolia
Lepidothamnus laxifolius
Phyllocladus aspleniifolius
Araucariaceae
Agathis australis
Wollemia nobilis
Araucaria heterophylla
Araucaria mirabilis
Cheirolepidiac.
Himeriella muensteri
Tomaxellia biforme
Kachaikestrobus acuminatus
New total plant from Arkansas
Alvinia bohemica
Pararaucaria patagonica
Cupressaceae
Cupressus funebris
Callitris muelleri
Cryptomeria japonica
Sequoia sempervirens
Athrotaxis selaginoides
Taiwania cryptomerioides
Cunninghamia lanceolata
Taxaceae
Taxus wallichiana
Sciadopityac.
Sciadopitys verticillata
Schizolepidopsis longipetiolus
Voltzian voltz.
Telemachus aequatus
Nothodacrium warrenii
Rissikia media
Voltzia hexagona
Walchian voltz.
Emporia lockardii
Hanskerpia hamiltonensis
Cordaitales
Cordaixylon dumusum
0.05
Cheirolepidiaceae
New total
Walchian Voltziales
(Cordaitales)
Voltzian Voltziales
Sciadopityaceae
