# Supplementary material for: Revisiting the enigmatic Cheirolepidiaceae: origins, phylogenetic relationships, and a new whole-plant concept
Source: Ann Bot. 2026 Mar 30;137(6):1876–901. doi: 10.1093/aob/mcag069 (PMC13275020; doi:10.1093/aob/mcag069)
Supplement: mcag069_Supplementary_Data [file mcag069_supplementary_data.zip › AppendixS4-FBDAnalyses.zip!/AppendixS4-FBDAnalyses/AppendixS4D-PosteriorAges.docx]

| **Group** | **Mean age (millions of years)** | **Age 95% HPD (millions of years)** |
| --- | --- | --- |
| Base of the tree (Cordaitales + conifers) | 342.91 | 347.10–331.59 |
| Voltziales | 331.47 | 345.18–315.83 |
| Crown+Cheirolepidiaceae | 256.98 | 289.35–225.11 |
| Cheirolepidiaceae | 202.65 | 231.94–178.13 |
| Crown conifers | 236.61 | 268.67–206.51 |

| **Group** | **Mean age (millions of years)** | **Age 95% HPD (millions of years)** |
| --- | --- | --- |
| Crown group Pinaceae | 74.89 | 97.23–55.05 |
| Crown group Cupressales + Araucariales | 200.85 | 229.69–175.8 |
| Crown group Cupressales | 162.17 | 198.54–129.27 |
| Crown group Araucariales | 182.01 | 206.2–164.64 |
| Crown Araucariaceae | 58.55 | 80.93–39.59 |
| Crown Podocarpaceae | 95.60 | 119.34–73.05 |
