# Supplementary material for: Revisiting the enigmatic Cheirolepidiaceae: origins, phylogenetic relationships, and a new whole-plant concept
Source: Ann Bot. 2026 Mar 30;137(6):1876–901. doi: 10.1093/aob/mcag069 (PMC13275020; doi:10.1093/aob/mcag069)
Supplement: mcag069_Supplementary_Data [file mcag069_supplementary_data.zip › AppendixS4-FBDAnalyses.zip!/AppendixS4-FBDAnalyses/AppendixS4C-FigureMaking/Rplot.pdf]

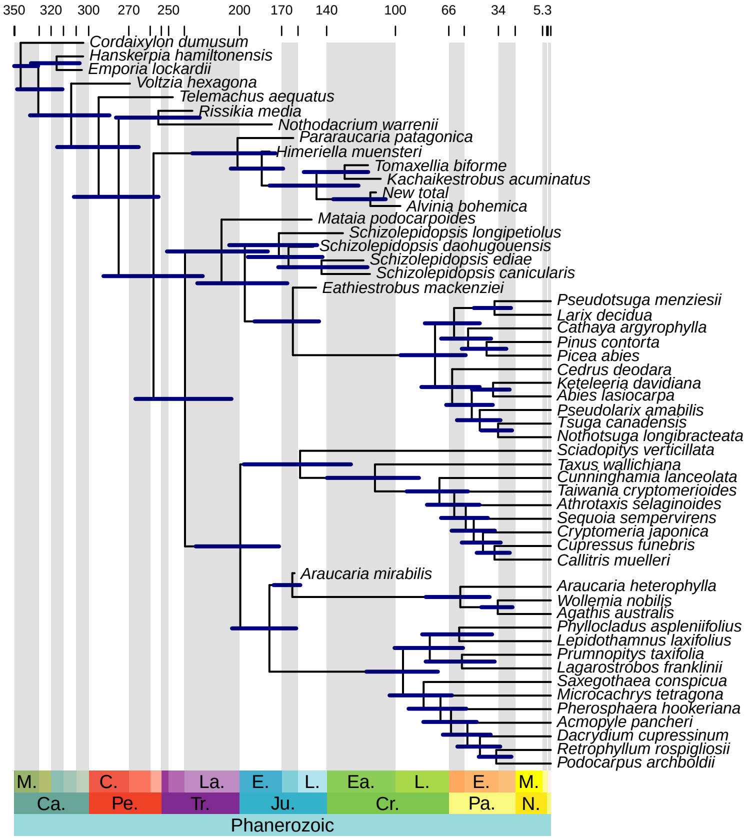

350
320
300
270
250
200
170
140
100
66
34
5.3
Cordaixylon dumusum
Hanskerpia hamiltonensis
Emporia lockardii
Voltzia hexagona
Telemachus aequatus
Rissikia media
Nothodacrium warrenii
Pararaucaria patagonica
Himeriella muensteri
Tomaxellia biforme
Kachaikestrobus acuminatus
New total
Alvinia bohemica
Mataia podocarpoides
Schizolepidopsis longipetiolus
Schizolepidopsis daohugouensis
Schizolepidopsis ediae
Schizolepidopsis canicularis
Eathiestrobus mackenziei
Pseudotsuga menziesii
Larix decidua
Cathaya argyrophylla
Pinus contorta
Picea abies
Cedrus deodara
Keteleeria davidiana
Abies lasiocarpa
Pseudolarix amabilis
Tsuga canadensis
Nothotsuga longibracteata
Sciadopitys verticillata
Taxus wallichiana
Cunninghamia lanceolata
Taiwania cryptomerioides
Athrotaxis selaginoides
Sequoia sempervirens
Cryptomeria japonica
Cupressus funebris
Callitris muelleri
Araucaria mirabilis
Araucaria heterophylla
Wollemia nobilis
Agathis australis
Phyllocladus aspleniifolius
Lepidothamnus laxifolius
Prumnopitys taxifolia
Lagarostrobos franklinii
Saxegothaea conspicua
Microcachrys tetragona
Pherosphaera hookeriana
Acmopyle pancheri
Dacrydium cupressinum
Retrophyllum rospigliosii
Podocarpus archboldii
M.
La.
E.
L.
Ea.
L.
E.
M.
Ca.
Pe.
Tr.
Ju.
Cr.
Pa.
N.
Phanerozoic
