# Supplementary material for: Revisiting the enigmatic Cheirolepidiaceae: origins, phylogenetic relationships, and a new whole-plant concept
Source: Ann Bot. 2026 Mar 30;137(6):1876–901. doi: 10.1093/aob/mcag069 (PMC13275020; doi:10.1093/aob/mcag069)
Supplement: mcag069_Supplementary_Data [file mcag069_supplementary_data.zip › AppendixS5-PlaceMyFossil_Analysis.zip!/AppendixS5-PlaceMyFossil_Analysis/FigureAppendixS5.pdf]

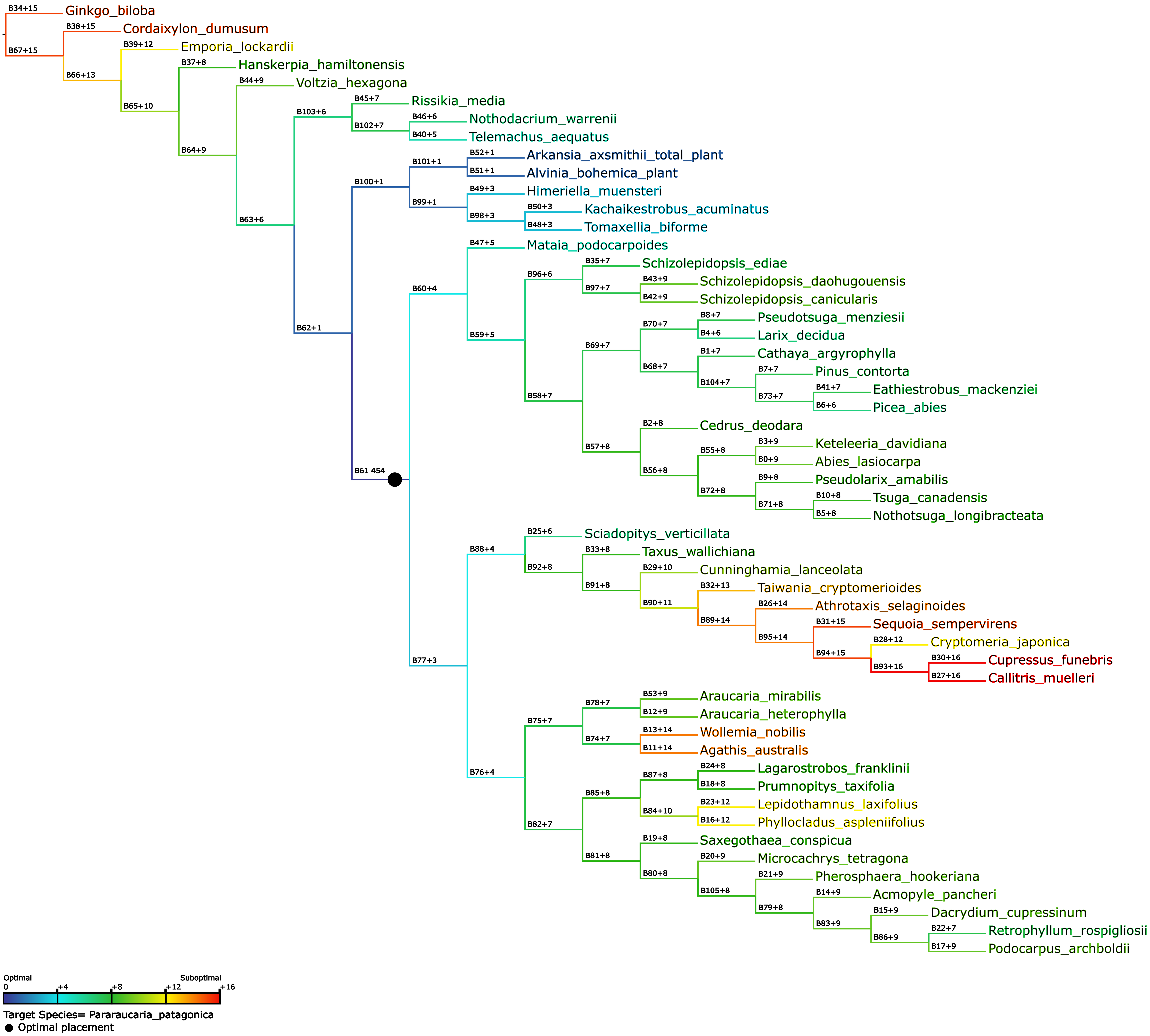

Figure Appendix S5. Output from the TNT script *PlaceMyFossil* (Catalano et al., 2024) showing optimal placement of *Pararaucaria patagonica* (black filled circle) and all its alternative placements in the tree using a map of color that ranges from blue (most optimal) to red (less optimal). Over the branches, the B indicates the branch number; the number next to B61 (optimal placement) indicates the length of the optimal tree (for the morphological matrix only); the number nex to all other B numbers indicates the additional number of steps in the tree implied by that positioning of *Pararaucaria patagonica*.
